# Supplementary material for: All-round catalytic and atroposelective strategy via dynamic kinetic resolution for N-/2-/3-arylindoles
Source: Nat Commun. 2023 Sep 7;14:5502. doi: 10.1038/s41467-023-41299-2 (PMC10485016; doi:10.1038/s41467-023-41299-2)
Supplement: Supplementary file 1 — Supplementary Information [file 41467_2023_41299_MOESM1_ESM.pdf]

Supplementary Information

All-round catalytic and atroposelective strategy via dynamic kinetic resolution for *N*-/2-/3-arylindoles

Ahreum Kim,[1] Chanhee Lee,[1] Jayoung Song,[2] Sang Kook Lee,[2] Yongseok Kwon[1,*]

[1]School of Pharmacy, Sungkyunkwan University, Suwon 16419, Republic of Korea

[2]College of Pharmacy, Seoul National University, Seoul 08826, Republic of Korea

correspondence to:

y.kwon@skku.edu

1 Materials and Methods S2
2 Synthesis of **1** S4
2.1. Synthesis of Substituted *N*-Arylindole Intermediates S4
2.2. Synthesis of **1** S18
3 Synthesis of **4** S42
3.1. Synthesis of Substituted 2-Nitroarylindoles S42
3.2. Synthesis of Substituted 2-Aminoarylindole Intermediates S49
3.3. Synthesis of **4** S59
4 Synthesis of **6** S71
4.1. Synthesis of Substituted 3-Arylindole Intermediates S71
4.2. Synthesis of **6** S82
5 Reaction Optimizations for **3**, **5** and **7** S95
5.1. Reaction Optimizations for **3** S95
5.2. Reaction Optimizations for **5** S99
5.3. Reaction Optimizations for **7** S102
6 Reaction Procedures for **3**, **5** and **7** S104
7 Characterization and Spectra of **3** S105
7.1. NMR Spectra of **3** S115
7.2. HPLC Traces of **3** S142
8 Characterization and Spectra of **5** S161
8.1. NMR Spectra of **5** S174
8.2. HPLC Traces of **5** S188
9 Characterization and Spectra of **7** S198
9.1. NMR Spectra of **7** S208
9.2. HPLC Traces of **7** S218
10 Chemical Modifications of **3aa** S228
10.1. Reaction Procedures for Modifications of **3aa** S228
10.2. Characterization and Spectra of **8** and **9** S229
11 Experiment for X-ray Crystallography S235
11.1. Experiment for X-ray Crystallography of **3ba** S235
11.2. Experiment for X-ray Crystallography of **3ld** S243
11.3. Experiment for X-ray Crystallography of **7da** S250
12 Computational Details S259
12.1. Computational Studies to Determine Rotational Barriers S259
12.2. Computational Studies for ECD Spectra S265
12.3. Computational Studies to Investigate the Origin of Enantioselectivity ... S267
13 Biological Evaluation S285
14 References S286

1 Materials and Methods

Room temperature is defined as 21–23 °C. All reagents were purchased from commercial suppliers and used without further purification, unless otherwise noted. All solvents were purchased from commercial suppliers and used without further purification, unless otherwise noted.

Routine ^{1}H NMR spectra were recorded on Bruker 400 MHz spectrometers at ambient temperature unless otherwise stated. All NMR solvents were purchased from Cambridge Isotope Laboratories and used without further purification. Chloroform-*d* was stored at ambient temperature. Spectra were processed using MestReNova 14.1 using the automatic phasing and polynomial baseline correction capabilities. Splitting was determined using the automatic multiplet analysis function with manual intervention as necessary. Spectral data are reported as follows: chemical shift (multiplicity [singlet (s), broad singlet (brs), doublet (d), triplet (t), quartet (q), pentet (p), multiplet (m), doublet of doublets (dd), doublet of doublet of doublets (ddd), doublet of triplet of doublets (dtd), doublet of doublet of doublet of doublets (dddd), doublet of triplets (dt), triplet of doublets (td), etc.], coupling constant, integration). Chemical shifts are reported in ppm (δ), and coupling constants are reported in Hz. ^{1}H Resonances are referenced to solvent residual peaks for $CDCl_3$ (7.26 ppm).[1] Routine ^{13}C NMR spectra were recorded on Bruker 400 MHz or 700 MHz spectrometers with protons fully decoupled. ^{13}C Resonances are reported in ppm relative to solvent residual peaks for $CDCl_3$ (77.16 ppm).[1] Note: Small deviations in chemical shifts may be observed depending on the concentration of NMR samples.

ECD spectra were recorded on a Jasco J-1500 CD Spectrometer. Infrared spectra were recorded on a Jasco FT/IR-4600 spectrometer, and ν_{max} are partially reported in cm^{-1}. Samples for high-resolution mass spectrometry were submitted to the SM Lab at Suwon, Republic of Korea. Data was acquired on a JEOL JMS-700 instrument equipped with an EI detector. Analytical thin-layer chromatography was performed using 60 Å Silica Gel F_{254} pre-coated plates (0.25 mm thickness). TLC plates were visualized by irradiation with a UV lamp. Normal-phase column chromatography was performed using 60 Å Silica Gel (32–62 micron) with an appropriate mobile phase composition and gradient. Optical rotations were recorded on a Jasco P-2000 polarimeter at the sodium D-line (589 nm) using a cell of 1 dm path length. Concentration values are reported in units of g/100 mL. Normal-phase high-performance liquid chromatography was performed using an Agilent 1260 series instrument equipped with a diode array detector or multiple wavelength detector and columns (chiral supports) from Daicel Chemical Industries.

Abbreviation

CAN	Ceric ammonium nitrate
DME	1,2-Dimethoxyethane
DMF	*N*,*N*-Dimethylformamide
EI	Electron ionization
EtOAc	Ethyl acetate
HPLC	High-performance liquid chromatography
HRMS	High-resolution mass spectrometry
Hx	Hexanes
NMR	Nuclear magnetic resonance
QM	Quantum-mechanical
rt	Room temperature
M.S.	Molecular sieves
SE	Semiempirical
SRB	Sulforhodamine B
TBAB	Tetrabutylammonium bromide
THF	Tetrahydrofuran
TLC	Thin-layer chromatography
ZPVE	Zero-point vibrational energy

2 Synthesis of 1

2.1 Synthesis of Substituted *N*-Arylindole Intermediates (S1–S12)

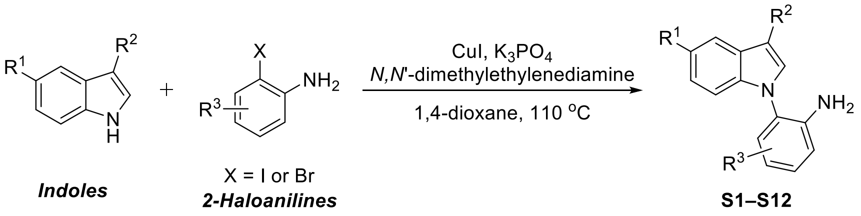

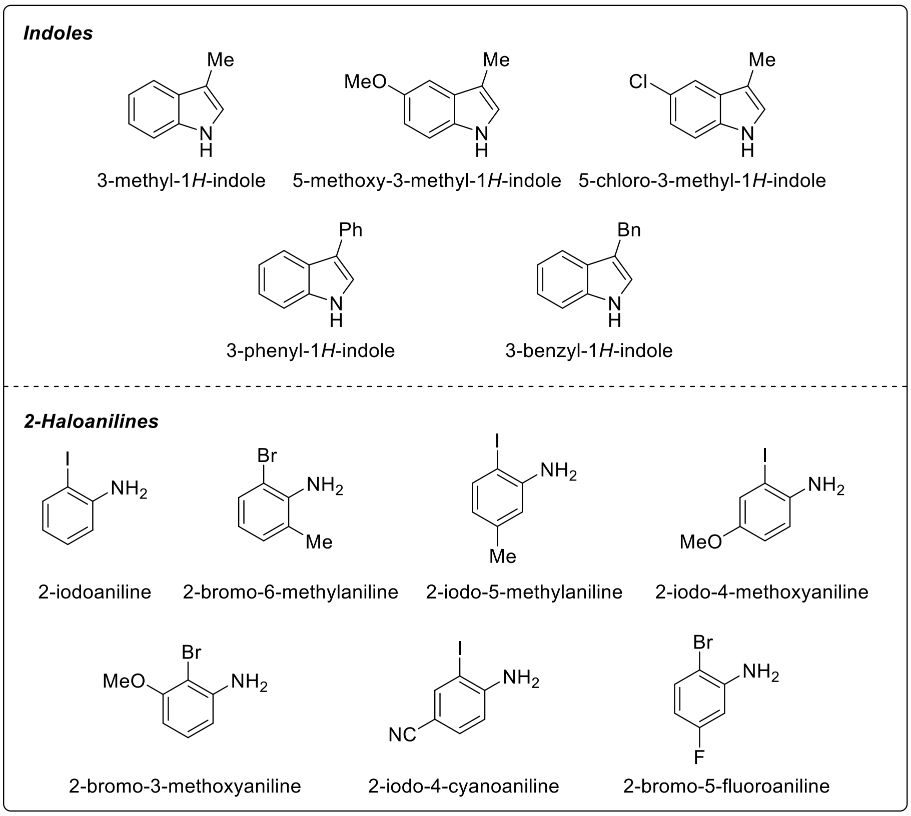

Procedure 1: To a reaction tube equipped with a magnetic stirring bar, 3-methylindole (1.0 equiv), 2-haloaniline (1.4 equiv), copper iodide (0.3 equiv), *N,N'*-dimethylethylenediamine (1.4 equiv), and potassium phosphate (2.0 equiv) were added and subsequently dissolved in 1,4-dioxane (1.0 M). The reaction was heated to 110 °C and allowed to stir for overnight. The progress of the reaction was monitored by TLC. The reaction was diluted with EtOAc, transferred to a separatory funnel, and quenched with a saturated aqueous NH_4Cl. The organic layer was separated, and the aqueous layer was extracted an additional two times with EtOAc. The combined organic layers were then rinsed with water, dried with anhydrous $MgSO_4$, and concentrated *in vacuo*. The crude material was then purified by flash chromatography to afford the desired material **S1**–**S12**.

Characterization and Spectra of Substituted *N*-Arylindole Intermediates (S1–S12)

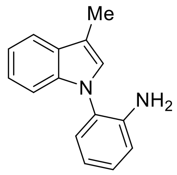

2-(3-Methyl-1*H*-indol-1-yl)aniline (S1) was synthesized by following Procedure 1. The crude material was purified by normal-phase column chromatography using an eluent of 9% EtOAc/Hx to provide **S1** (2.09 g, 94%).

^{1}H NMR (400 MHz, $CDCl_3$) δ 7.77–7.72 (m, 1H), 7.33–7.19 (m, 5H), 7.06 (q, *J* = 1.1 Hz, 1H), 6.95–6.85 (m, 2H), 3.60 (s, 2H), 2.49 (s, 3H).
^{13}C NMR (100 MHz, $CDCl_3$) δ 143.2, 137.0, 129.0, 128.9, 128.6, 126.2, 125.1, 122.2, 119.6, 119.1, 118.5, 116.2, 112.4, 110.7, 9.7.

The spectral data were identical with those previously reported.[2]

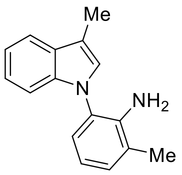

2-Methyl-6-(3-methyl-1*H*-indol-1-yl)aniline (S2) was synthesized by following Procedure 1. The crude material was purified by normal-phase column chromatography using an eluent of 13% EtOAc/Hx to provide **S2** (514 mg, 73%).

^{1}H NMR (400 MHz, $CDCl_3$) δ 7.78–7.60 (m, 1H), 7.33–7.05 (m, 5H), 7.03 (q, *J* = 1.1 Hz, 1H), 6.81 (t, *J* = 7.6 Hz, 1H), 3.56 (brs, 2H), 2.44 (d, *J* = 1.1 Hz, 3H), 2.29 (s, 3H).
^{13}C NMR (100 MHz, $CDCl_3$) δ 141.6, 136.9, 130.0, 129.1, 126.4, 126.3, 125.0, 123.6, 122.2, 119.6, 119.1, 117.9, 112.4, 110.8, 17.8, 9.8.
IR (FT-ATR, cm^{-1}, $CHCl_3$) ν_{max} 3969, 3930, 3903, 2880, 3842, 3780, 3757, 3734, 3677, 3652, 3629, 3595, 3475, 3383, 3201, 3047, 2970, 2916, 2858, 2738, 2623, 2565, 2526, 2461, 2403, 2360, 2333, 2318, 2214, 2117, 2006, 1894, 1851, 1801, 1774, 1689, 1616, 1589, 1558, 1481, 1454, 1365, 1308, 1281, 1250, 1227, 1176, 1146, 1122, 1072, 1034, 1011, 957, 926, 845, 779, 733, 667.
HRMS (EI) *m*/*z*: $[M]^+$ Calcd for $C_{16}H_{16}N_2$ 236.1314; found 236.1311.

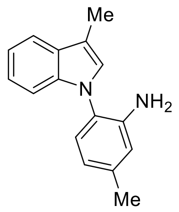

5-Methyl-2-(3-methyl-1*H*-indol-1-yl)aniline (S3) was synthesized by following Procedure 1. The crude material was purified by normal-phase column chromatography using an eluent of 13% EtOAc/Hx to provide **S3** (536 mg, 99%).

^{1}H NMR (400 MHz, $CDCl_3$) δ 7.73–7.65 (m, 1H), 7.27–7.14 (m, 3H), 7.13–7.07 (m, 1H), 7.01 (q, J = 1.1 Hz, 1H), 6.73–6.64 (m, 2H), 3.53 (s, 2H), 2.45 (d, J = 1.3 Hz, 3H), 2.40 (s, 3H).
^{13}C NMR (100 MHz, $CDCl_3$) δ 142.9, 139.0, 136.8, 129.0, 128.4, 126.4, 122.7, 122.2, 119.5, 119.4, 119.1, 116.8, 112.2, 110.7, 21.4, 9.8.

The spectral data were identical with those previously reported.[2]

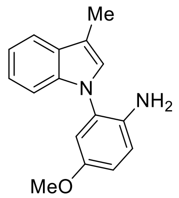

4-Methoxy-2-(3-methyl-1*H*-indol-1-yl)aniline (S4) was synthesized by following Procedure 1. The crude material was purified by normal-phase column chromatography using an eluent of 75% CH_2Cl_2/Hx to provide **S4** (513 mg, 89%).

^{1}H NMR (400 MHz, $CDCl_3$) δ 7.70–7.62 (m, 1H), 7.25–7.12 (m, 3H), 6.99 (d, J = 1.3 Hz, 1H), 6.84 (dd, J = 8.7, 2.9 Hz, 1H), 6.79 (d, J = 2.9 Hz, 1H), 6.72 (d, J = 8.7 Hz, 1H), 3.72 (s, 3H), 3.29 (s, 2H), 2.42 (d, J = 1.4 Hz, 3H).
^{13}C NMR (100 MHz, $CDCl_3$) δ 152.4, 136.7, 136.6, 128.9, 126.0, 125.7, 122.3, 119.6, 119.1, 117.4, 115.2, 113.4, 112.4, 110.6, 55.7, 9.6.

The spectral data were identical with those previously reported.[2]

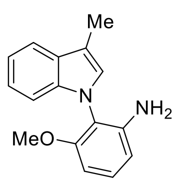

3-Methoxy-2-(3-methyl-1*H*-indol-1-yl)aniline (S5) was synthesized by following Procedure 1. The crude material was purified by normal-phase column chromatography using an eluent of 75% CH_2Cl_2/Hx to provide **S5** (328 mg, 22%).

^{1}H NMR (400 MHz, $CDCl_3$) δ 7.74–7.57 (m, 1H), 7.19 (m, 3H), 7.09–6.98 (m, 1H), 6.93 (d, J = 1.3 Hz, 1H), 6.47 (ddd, J = 13.3, 8.2, 1.2 Hz, 2H), 3.68 (s, 3H), 3.52 (brs, 2H), 2.44 (d, J = 1.2 Hz, 3H).

^{13}C NMR (100 MHz, $CDCl_3$) δ 157.3, 145.4, 136.6, 129.4, 128.9, 126.5, 122.0, 119.4, 119.0, 113.6, 112.1, 110.5, 108.6, 101.4, 55.9, 9.9.
IR (FT-ATR, cm^{-1}, $CHCl_3$) ν_{max} 3977, 3953, 3934, 3907, 3884, 3845, 3807, 3980, 3757, 3722, 3683, 3656, 3629, 3591, 3572, 3548, 3483, 3390, 3302, 3209, 3012, 2935, 2862, 2839, 2596, 2526, 2403, 2372, 2345, 2314, 2063, 1994, 1917, 1840, 1797, 0689, 1616, 1508, 1477, 1457, 1389, 1369, 1327, 1308, 1265, 1215, 1130, 1095, 1068, 1045, 1011, 930, 841, 741, 667.
HRMS (EI) *m*/*z*: $[M]^+$ Calcd for $C_{16}H_{16}N_2O$ 252.1263; found 252.1263.

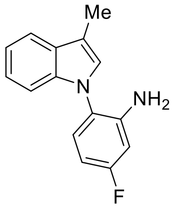

5-Fluoro-2-(3-methyl-1*H*-indol-1-yl)aniline (S6) was synthesized by following Procedure 1. The crude material was purified by normal-phase column chromatography using an eluent of 10% EtOAc/Hx to provide **S6** (300 mg, 55%).

^{1}H NMR (400 MHz, $CDCl_3$) δ 7.88–7.82 (m, 1H), 7.43–7.35 (m, 2H), 7.32–7.24 (m, 2H), 7.11 (d, *J* = 1.2 Hz, 1H), 6.74–6.62 (m, 2H), 3.79 (s, 2H), 2.60 (d, *J* = 1.3 Hz, 3H).
^{13}C NMR (100 MHz, $CDCl_3$) δ 163.0 (d, *J* = 243.0 Hz, 1C), 144.9 (d, *J* = 12.0 Hz, 1C), 136.9, 130.0 (d, *J* = 8.0 Hz, 1C), 129.0, 126.2, 122.4, 121.0 (d, *J* = 3.0 Hz, 1C), 119.7, 119.2, 112.6, 110.5, 105.0 (d, *J* = 23.0 Hz, 1C), 102.5 (d, *J* = 26.0 Hz, 1C), 9.7.

The spectral data were identical with those previously reported.[2]

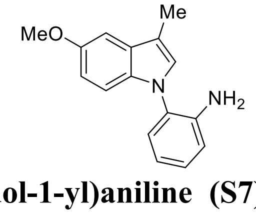

2-(5-Methoxy-3-methyl-1*H*-indol-1-yl)aniline (S7) was synthesized by following Procedure 1. The crude material was purified by normal-phase column chromatography using an eluent of 25% EtOAc/Hx to provide **S7** (672 mg, 89%).

^{1}H NMR (400 MHz, $CDCl_3$) δ 7.26–7.21 (m, 1H), 7.19 (dd, *J* = 7.7, 1.5 Hz, 1H), 7.10 (d, *J* = 2.5 Hz, 1H), 7.04 (d, *J* = 8.8 Hz, 1H), 7.02–6.96 (m, 1H), 6.92–6.80 (m, 3H), 3.92 (s, 3H), 3.62 (s, 2H), 2.39 (s, 3H).
^{13}C NMR (100 MHz, $CDCl_3$) δ 154.4, 143.2, 132.0, 129.4, 128.9, 128.6, 126.9, 125.4, 118.6, 116.3, 112.3, 112.1, 111.6, 101.0, 56.1, 9.8.
IR (FT-ATR, cm^{-1}, $CHCl_3$) ν_{max} 3907, 3880, 3607, 3757, 3734, 3656, 3629, 3591, 3467, 3371, 3205, 3005, 2931, 2862, 2831, 2738, 2692, 2623, 2580, 2461, 2430, 2372, 2349, 2314, 2229, 2087, 2060, 1928, 1898, 1859, 1793, 1689, 1616, 1504, 1481, 1454, 1388, 1346, 1308, 1284, 1238, 1215, 1173, 1149, 1122, 1061, 1029, 945, 876, 833, 744, 690, 660.
HRMS (EI) *m*/*z*: $[M]^+$ Calcd for $C_{16}H_{16}N_2O$ 252.1263; found 252.1263.

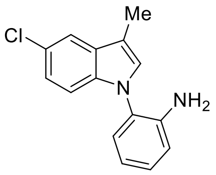

2-(5-Chloro-3-methyl-1*H*-indol-1-yl)aniline (S8) was synthesized by following Procedure 1. The crude material was purified by normal-phase column chromatography using an eluent of 25% EtOAc/Hx to provide **S8** (746 mg, 97%).

^{1}H NMR (400 MHz, $CDCl_3$) δ 7.61 (d, *J* = 2.0 Hz, 1H), 7.25 (td, *J* = 7.7, 1.6 Hz, 1H), 7.20–7.09 (m, 2H), 7.05 (s, 1H), 7.05–6.95 (m, 2H), 6.94–6.77 (m, 2H), 3.58 (s, 2H), 2.37 (d, *J* = 1.2 Hz, 3H).
^{13}C NMR (100 MHz, $CDCl_3$) δ 143.1, 135.2, 130.1, 129.3, 128.6, 127.6, 125.5, 124.7, 122.5, 118.7 (2C), 116.4, 112.2, 111.8, 9.66.
IR (FT-ATR, cm^{-1}, $CHCl_3$) ν_{max} 3950, 3930, 3907, 3884, 3856, 3826, 3807, 3780, 3757, 3718, 3680, 3656, 3629, 3591, 3568, 3471, 3379, 3201, 3062, 3039, 2920, 2862, 2773, 2738, 2684, 2619, 2565, 2488, 2445, 2372, 2349, 2310, 2201, 2148, 2110, 2087, 2021, 1986, 1925, 1902, 1863, 1793, 1693, 1616, 1504, 1450, 1369, 1338, 1308, 1269, 1219, 1148, 1092, 1049, 995, 968, 937, 860, 837, 791, 744, 714.
HRMS (EI) *m*/*z*: $[M]^+$ Calcd for $C_{15}H_{13}ClN_2$ 256.0767; found 256.0763.

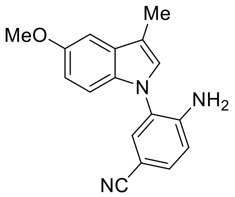

4-Amino-3-(5-methoxy-3-methyl-1*H*-indol-1-yl)benzonitrile (S9) was synthesized by following Procedure 1. The crude material was purified by normal-phase column chromatography using an eluent of 25% EtOAc/Hx to provide **S9** as a white solid (184 mg, 22%).

^{1}H NMR (400 MHz, $CDCl_3$) δ 7.50–7.41 (m, 2H), 7.06 (d, *J* = 2.4 Hz, 1H), 6.96 (d, *J* = 8.8 Hz, 1H), 6.91 (d, *J* = 0.8 Hz, 1H), 6.89–6.81 (m, 2H), 4.17 (s, 1H), 3.89 (s, 3H), 2.35 (d, *J* = 1.1 Hz, 3H).
^{13}C NMR (100 MHz, $CDCl_3$) δ 154.7, 147.4, 132.9, 132.7, 131.6, 129.8, 126.1, 124.9, 119.3, 115.9, 113.4, 112.8, 111.2, 101.4, 100.4, 56.1, 9.8.
IR (FT-ATR, cm^{-1}, $CHCl_3$) ν_{max} 3946, 3926, 3899, 3865, 3838, 3799, 3780, 3730, 3703, 3626, 3599, 3475, 3359, 3217, 3163, 3059, 3008, 2931, 2862, 2835, 2739, 2696, 2646, 2596, 2503, 2457, 2360, 2333, 2299, 2268, 2218, 2063, 1967, 1944, 1898, 1867, 1797, 1747, 1712, 1620, 1574, 1512, 1481, 1454, 1373, 1346, 1319, 1281, 1242, 1215, 1176, 1122, 1061, 1030, 972, 933, 837, 791, 752, 679.
HRMS (EI) *m*/*z*: $[M]^+$ Calcd for $C_{17}H_{15}N_3O$ 277.1215; found 277.1215.

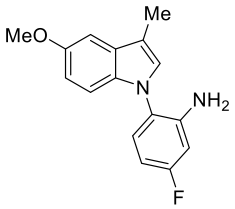

5-Fluoro-2-(5-methoxy-3-methyl-1*H*-indol-1-yl)aniline (S10) was synthesized by following Procedure 1. The crude material was purified by normal-phase column chromatography using an eluent of 13% EtOAc/Hx to provide **S10** (273 mg, 34%).

^{1}H NMR (400 MHz, $CDCl_3$) δ 7.10 (dd, *J* = 8.5, 5.9 Hz, 1H), 7.08 (d, *J* = 2.5 Hz, 1H), 6.99 (d, *J* = 8.8 Hz, 1H), 6.92 (d, *J* = 1.2 Hz, 1H), 6.86 (dd, *J* = 8.9, 2.5 Hz, 1H), 6.64–6.41 (m, 2H), 3.90 (s, 3H), 2.37 (d, *J* = 1.1 Hz, 3H).
^{13}C NMR (100 MHz, $CDCl_3$) δ 163.0 (d, *J* = 244.0 Hz, 1C), 154.4, 144.9 (d, *J* = 12.0 Hz, 1C), 132.2, 130.0 (d, *J* = 10.0 Hz, 1C), 129.4, 126.9, 121.3 (d, *J* = 2.0 Hz, 1C), 112.4, 112.3, 111.3, 105.1 (d, *J* = 22.0 Hz, 1C), 102.6 (d, *J* = 25.0 Hz, 1C), 101.1, 56.09, 9.80.
IR (FT-ATR, cm^{-1}, $CHCl_3$) ν_{max} 3988, 3945, 3926, 3899, 3865, 3838, 3822, 3799, 3780, 3730, 3699, 3626, 3599, 3475, 3371, 3213, 3078, 3001, 2924, 2858, 2835, 2742, 2603, 2488, 2461, 2422, 2360, 2333, 2295, 2268, 2087, 1894, 1863, 1797, 1620, 1512, 1481, 1450, 1392, 1346, 1284, 1238, 1165, 1119, 1061, 1026, 972, 933, 837, 791, 752, 679.
HRMS (EI) *m*/*z*: $[M]^+$ Calcd for $C_{16}H_{15}FN_2O$ 270.1168; found 270.1165.

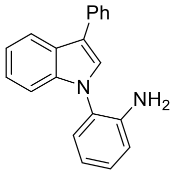

2-(3-Phenyl-1*H*-indol-1-yl)aniline (S11) was synthesized by following Procedure 1. The crude material was purified by normal-phase column chromatography using an eluent of 9% EtOAc/Hx to provide **S11** (146 mg, 44%).

^{1}H NMR (400 MHz, $CDCl_3$) δ 8.05–7.99 (m, 1H), 7.76–7.70 (m, 2H), 7.64 (dd, *J* = 7.9, 1.5 Hz, 1H), 7.51–7.44 (m, 2H), 7.42 (s, 1H), 7.35–7.17 (m, 2H), 7.14 (ddd, *J* = 8.0, 7.3, 1.4 Hz, 1H), 6.94–6.85 (m, 2H), 6.75 (dd, *J* = 8.0, 1.5 Hz, 1H), 6.48 (ddd, *J* = 7.9, 7.3, 1.5 Hz, 1H), 3.67 (s, 2H).
^{13}C NMR (100 MHz, $CDCl_3$) δ 143.2, 139.1, 137.3, 135.3, 129.5, 129.0 (2C), 128.8, 127.6 (2C), 126.29, 126.27, 122.8, 120.8, 120.2, 120.1, 118.8, 116.5, 114.9, 111.3.

The spectral data were identical with those previously reported.[3]

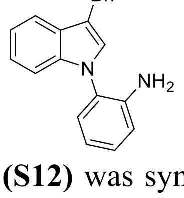

2-(3-Benzyl-1*H*-indol-1-yl)aniline (S12) was synthesized by following Procedure 1. The crude material was purified by normal-phase column chromatography using an eluent of 13% EtOAc/Hx to provide **S12** (600 mg, 67%).

^{1}H NMR (400 MHz, $CDCl_3$) δ 7.68–7.62 (m, 1H), 7.42–7.37 (m, 2H), 7.37–7.30 (m, 2H), 7.29–7.14 (m, 6H), 6.98 (s, 1H), 6.90–6.83 (m, 2H), 4.22 (s, 2H), 3.60 (s, 2H).
^{13}C NMR (100 MHz, $CDCl_3$) δ 143.2, 141.0, 137.0, 129.1, 128.9 (2C), 128.7, 128.5 (2C), 128.2, 126.8, 126.1, 125.0, 122.4, 119.8, 119.5, 118.6, 116.7, 116.3, 110.9, 31.7.
IR (FT-ATR, cm^{-1}, $CHCl_3$) ν_{max} 3924, 3904, 3874, 3857, 3838, 3802, 3753, 3727, 3705, 3650, 3624, 3599, 3545, 3467, 3368, 3209, 3058, 3028, 2907, 2845, 2359, 2349, 2337, 2327, 2296, 1945, 1684, 1618, 1551, 1507, 1464, 1455, 1431, 1374, 1310, 1264, 1221, 1200, 1157, 1137, 1075, 1029, 1011, 932, 848, 768, 743, 700, 669.
HRMS (EI) *m*/*z*: $[M]^+$ Calcd for $C_{21}H_{18}N_2$ 298.1470; found 298.1468.

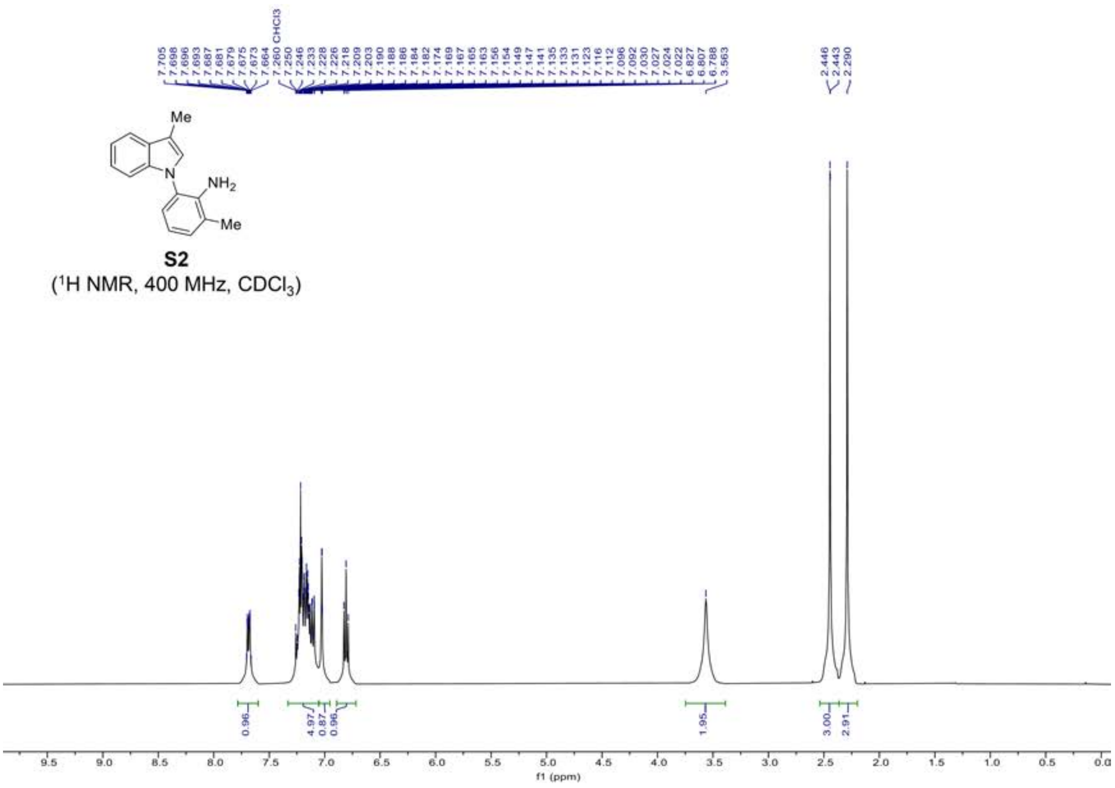
Me
NH2
Me
S2
(1H NMR, 400 MHz, CDCl3)
f1 (ppm)

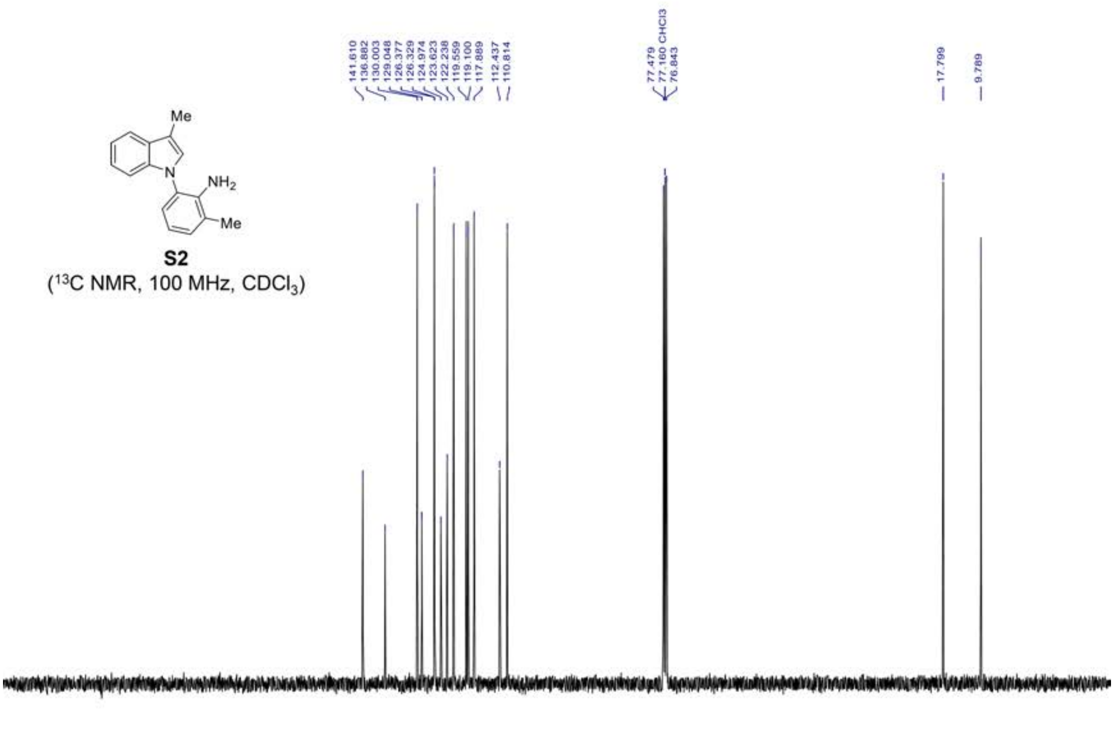
Me
NH2
Me
S2
(13C NMR, 100 MHz, CDCl3)
f1 (ppm)

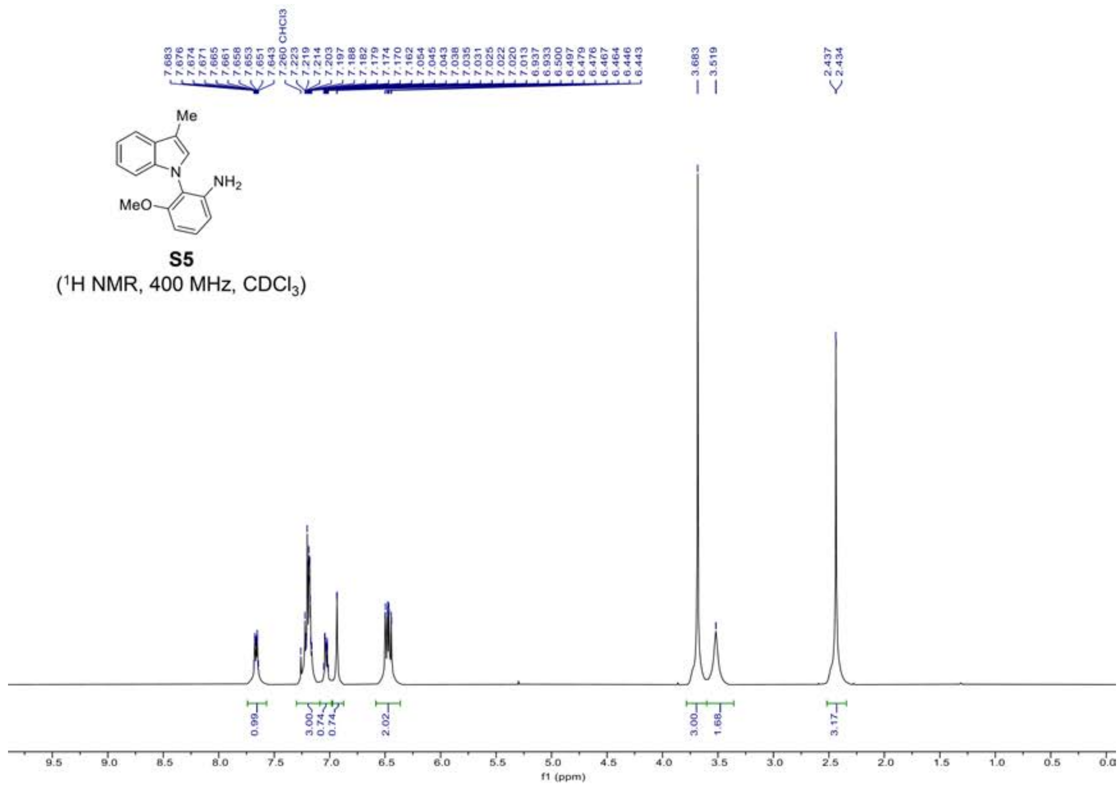
7.683
7.676
7.674
7.671
7.665
7.661
7.658
7.653
7.651
7.643
7.260 CHCl3
7.223
7.219
7.214
7.203
7.197
7.188
7.182
7.179
7.174
7.170
7.162
7.054
7.045
7.043
7.038
7.035
7.031
7.025
7.022
7.020
7.013
6.937
6.933
6.500
6.497
6.479
6.476
6.467
6.464
6.446
6.443
3.683
3.519
2.437
2.434
Me
N
NH2
MeO
S5
(1H NMR, 400 MHz, CDCl3)
0.99
3.00
0.74
0.74
2.02
3.00
1.68
3.17
9.5 9.0 8.5 8.0 7.5 7.0 6.5 6.0 5.5 5.0 4.5 4.0 3.5 3.0 2.5 2.0 1.5 1.0 0.5 0.0
f1 (ppm)

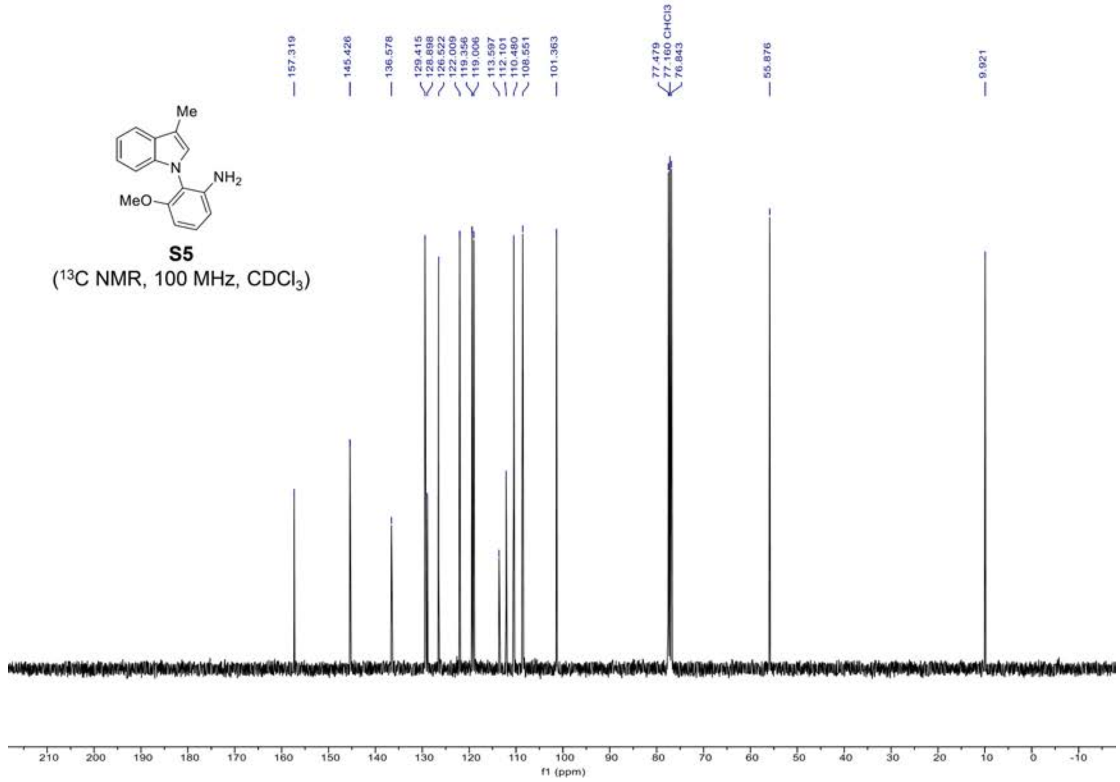
157.319
145.426
136.578
129.415
128.898
126.522
122.009
119.356
119.006
113.597
112.101
110.480
108.551
101.363
77.479
77.160 CHCl3
76.843
55.876
9.921
Me
N
NH2
MeO
S5
(13C NMR, 100 MHz, CDCl3)
210 200 190 180 170 160 150 140 130 120 110 100 90 80 70 60 50 40 30 20 10 0 -10
f1 (ppm)

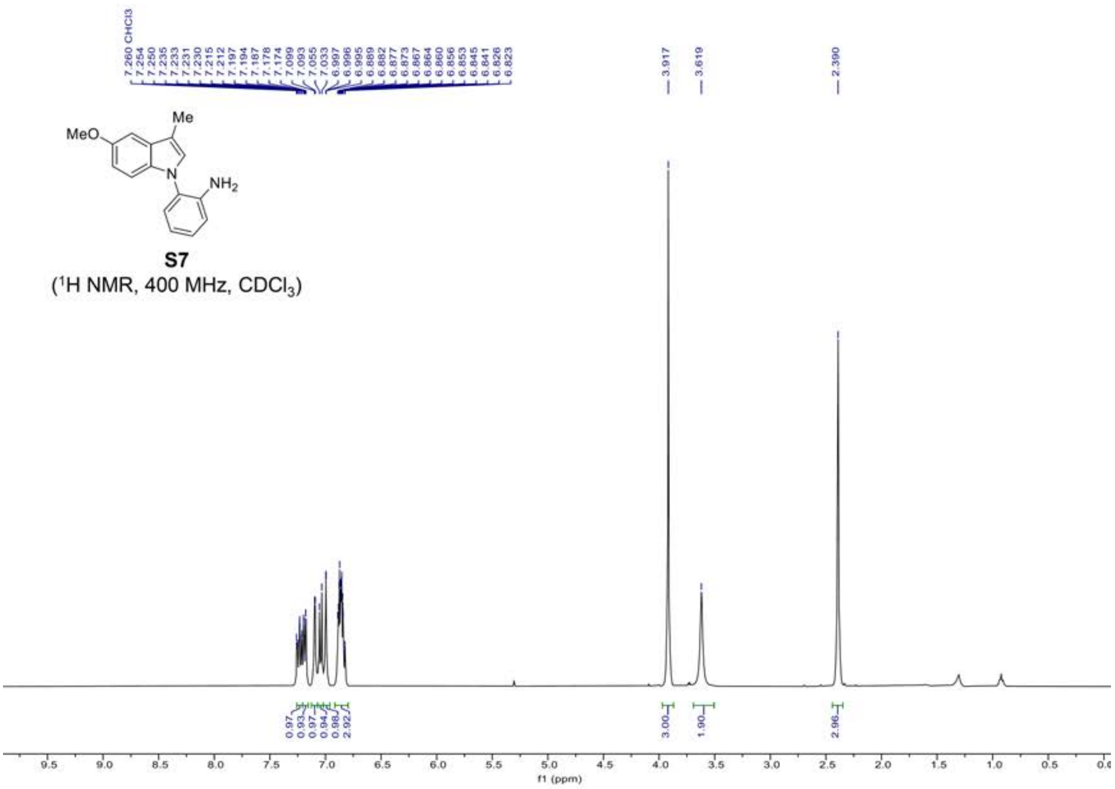
7.260 CHCl3
7.254
7.250
7.235
7.233
7.231
7.230
7.215
7.212
7.197
7.194
7.187
7.178
7.174
7.099
7.093
7.055
7.033
6.997
6.996
6.995
6.889
6.882
6.877
6.873
6.867
6.864
6.860
6.856
6.853
6.845
6.841
6.826
6.823
3.917
3.619
2.390
MeO
Me
N
NH2
S7
(1H NMR, 400 MHz, CDCl3)
0.97
0.93
0.97
0.94
0.98
2.92
3.00
1.90
2.96
f1 (ppm)

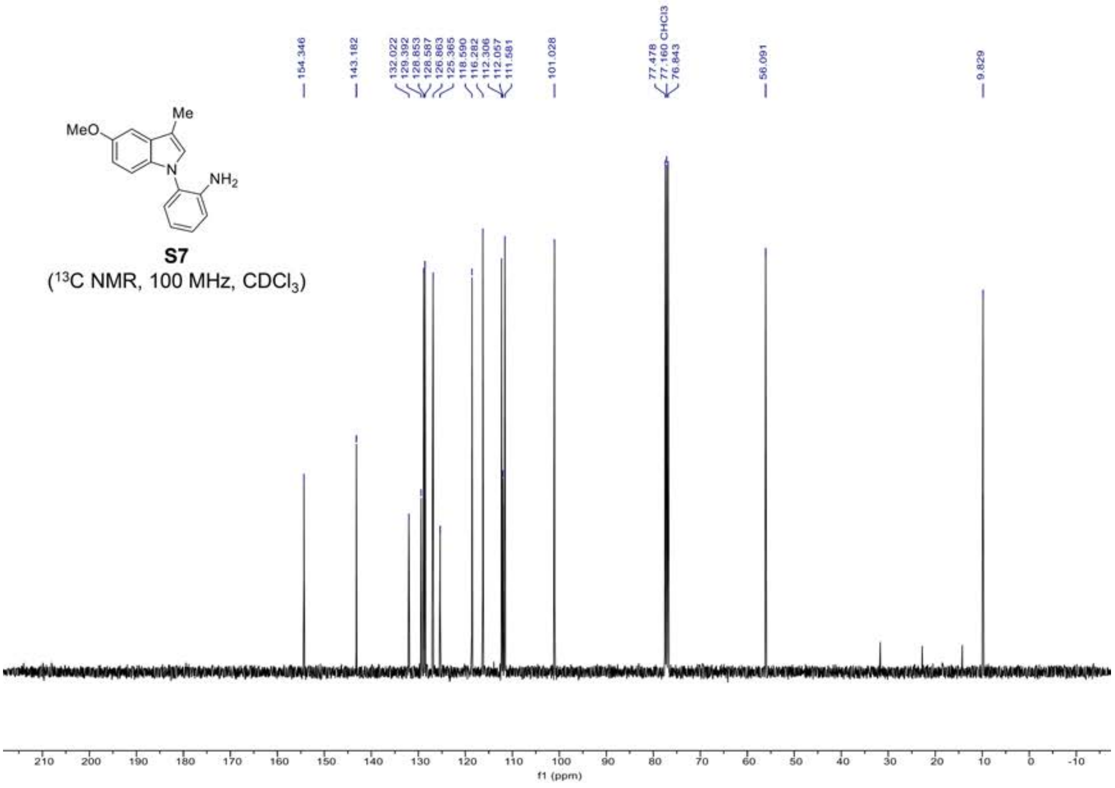
154.346
143.182
132.022
129.392
128.853
128.587
126.863
125.365
118.590
116.282
112.306
112.057
111.581
101.028
77.478
77.160 CHCl3
76.843
56.091
9.829
MeO
Me
N
NH2
S7
(13C NMR, 100 MHz, CDCl3)
f1 (ppm)

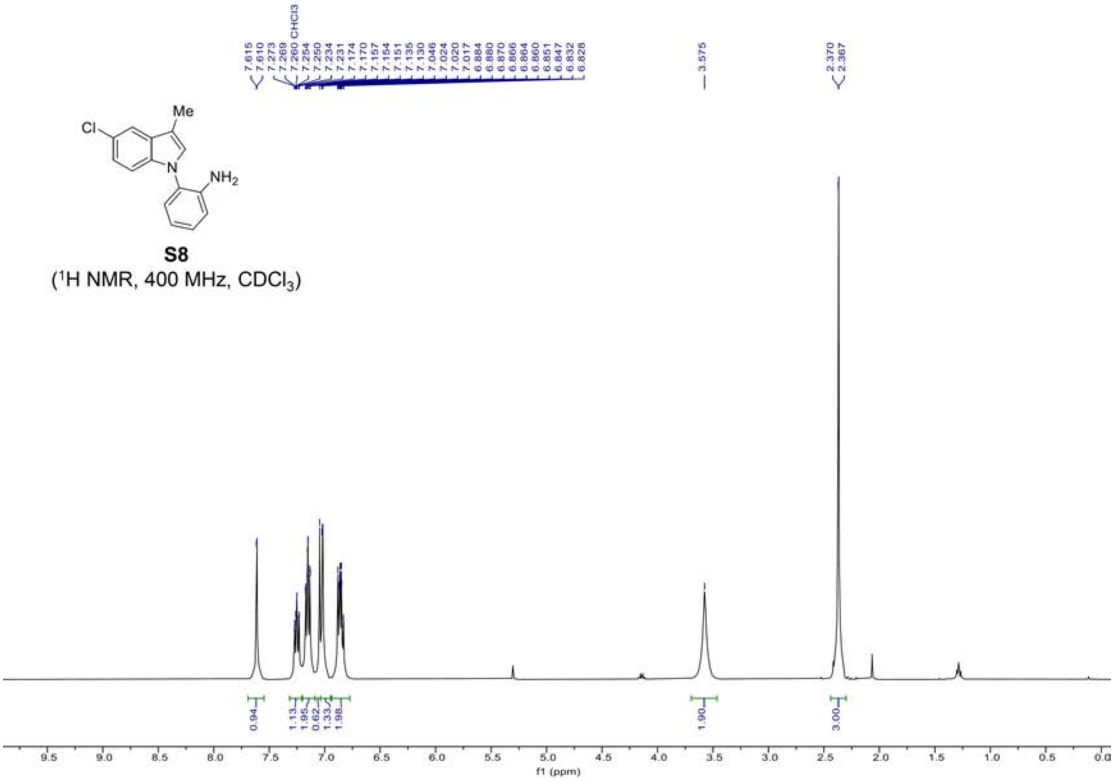

S8
(^{1}H NMR, 400 MHz, $CDCl_3$)

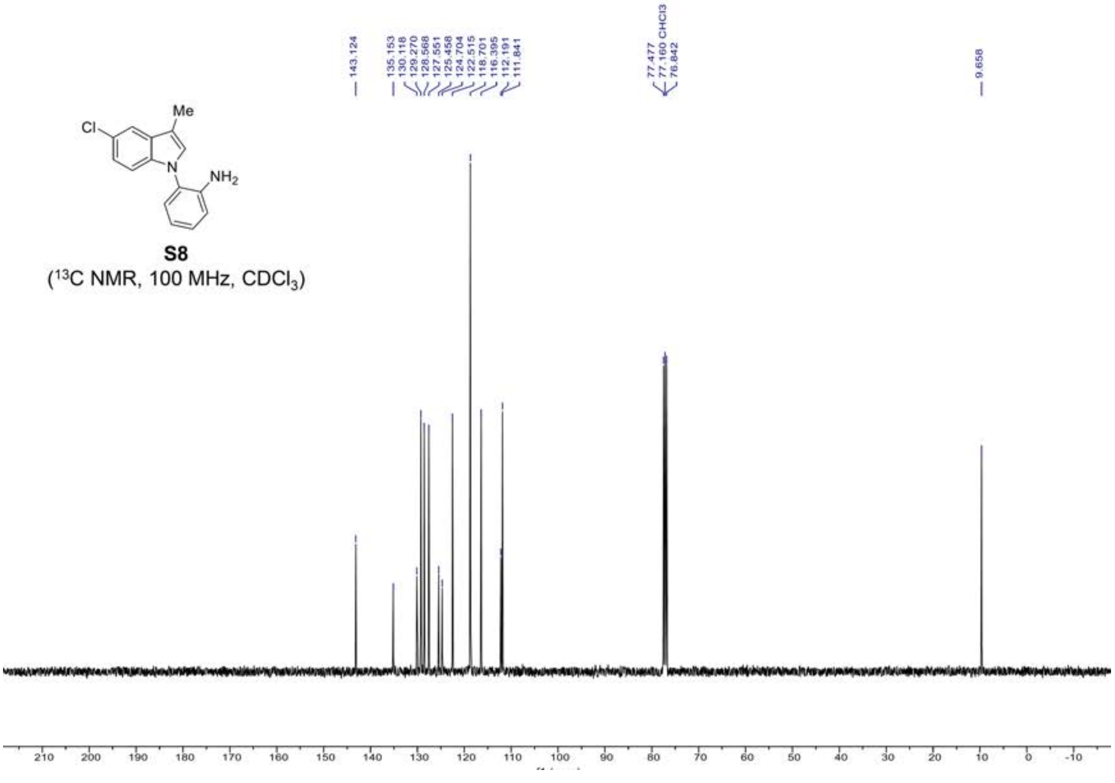

S8
(^{13}C NMR, 100 MHz, $CDCl_3$)

S9
(^{1}H NMR, 400 MHz, $CDCl_3$)

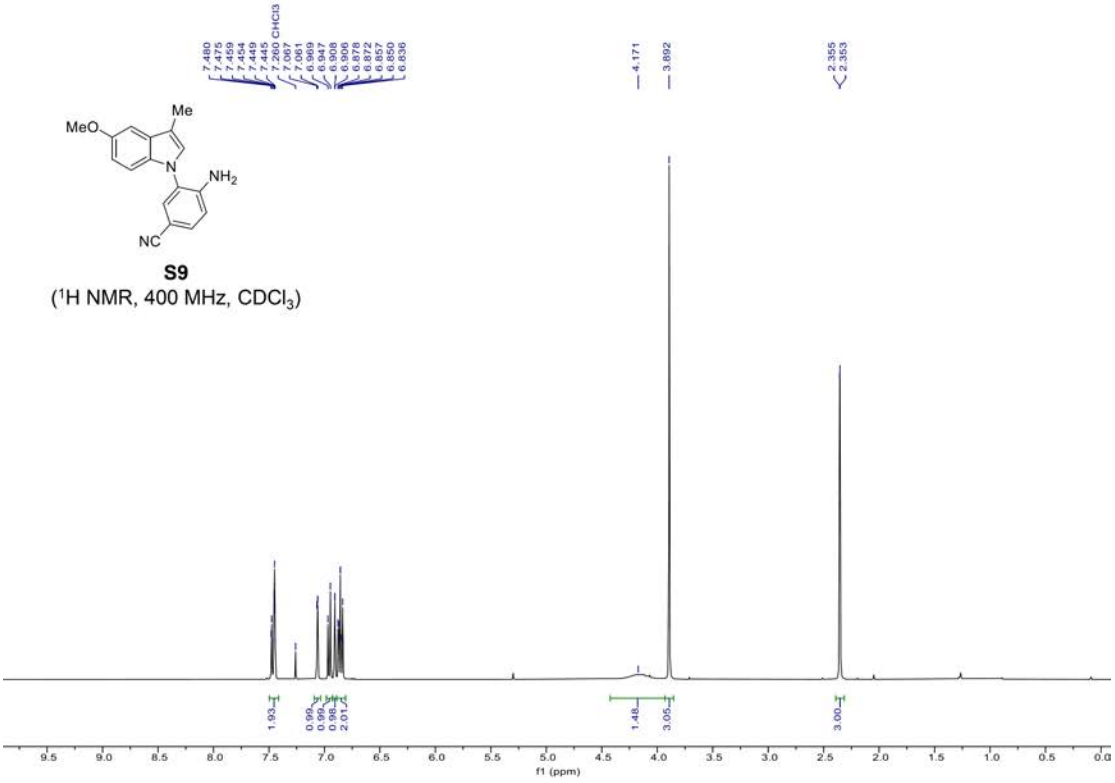

S9
(^{13}C NMR, 100 MHz, $CDCl_3$)

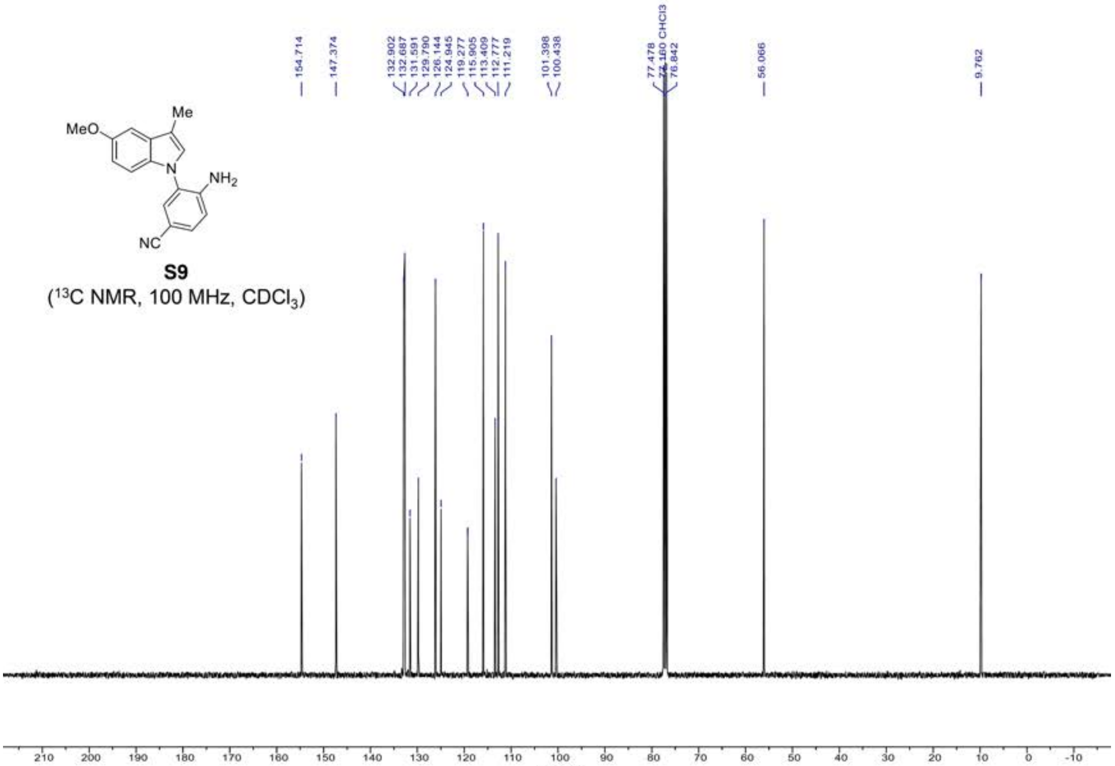

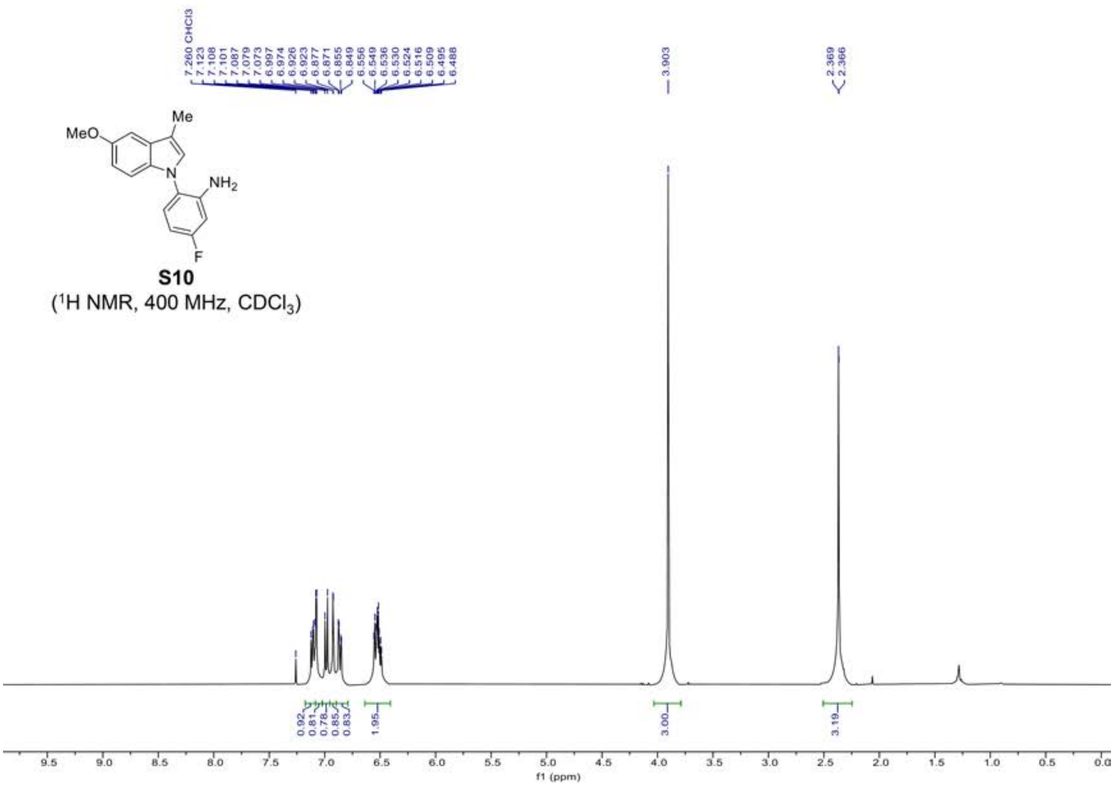

S10
(^{1}H NMR, 400 MHz, $CDCl_3$)

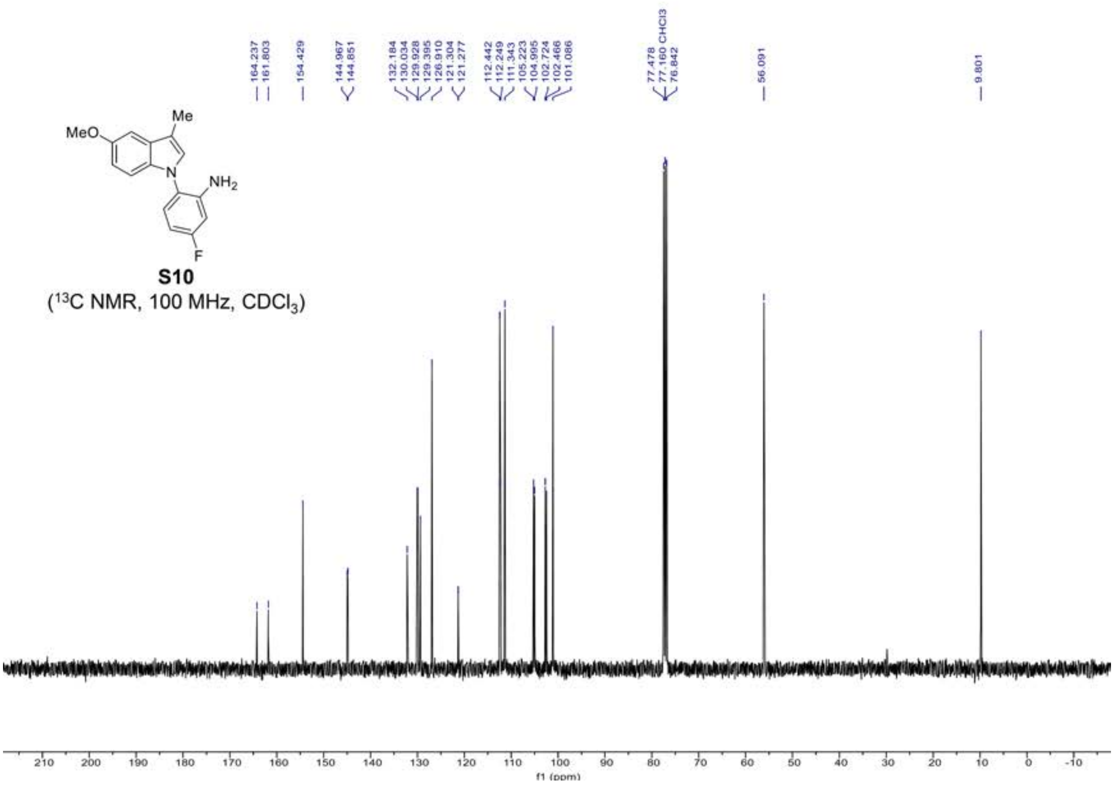

S10
(^{13}C NMR, 100 MHz, $CDCl_3$)

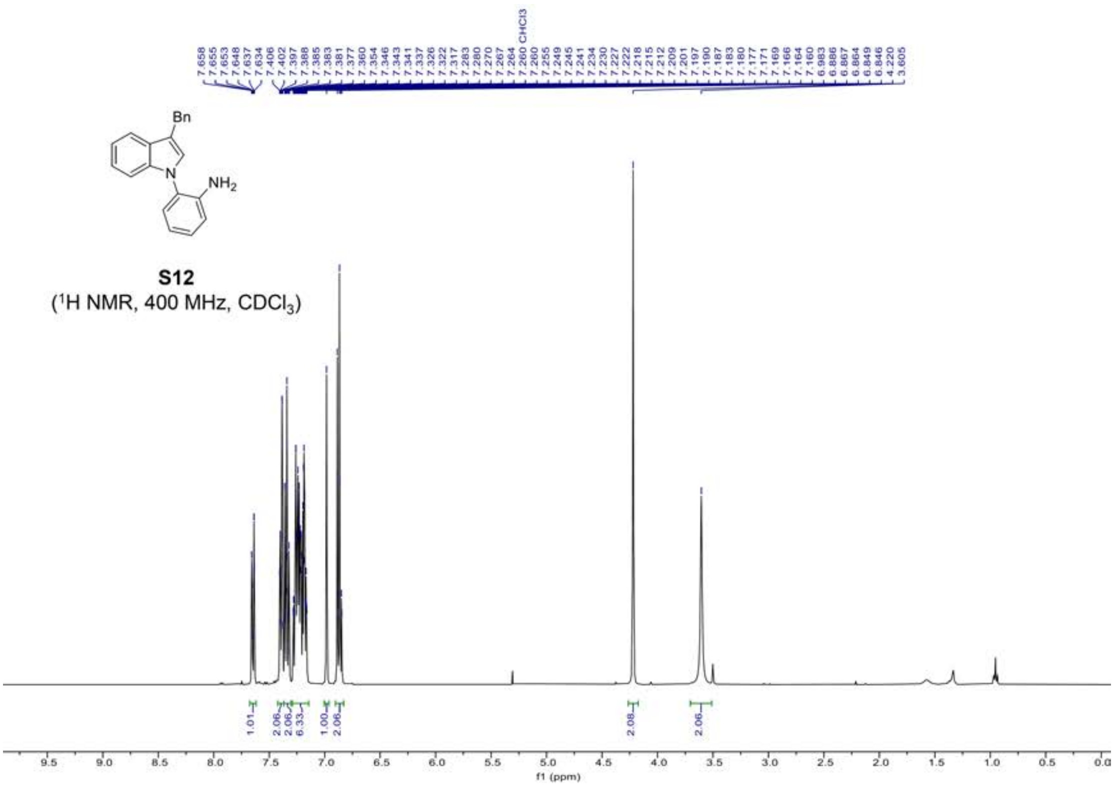

S12
(^{1}H NMR, 400 MHz, $CDCl_3$)

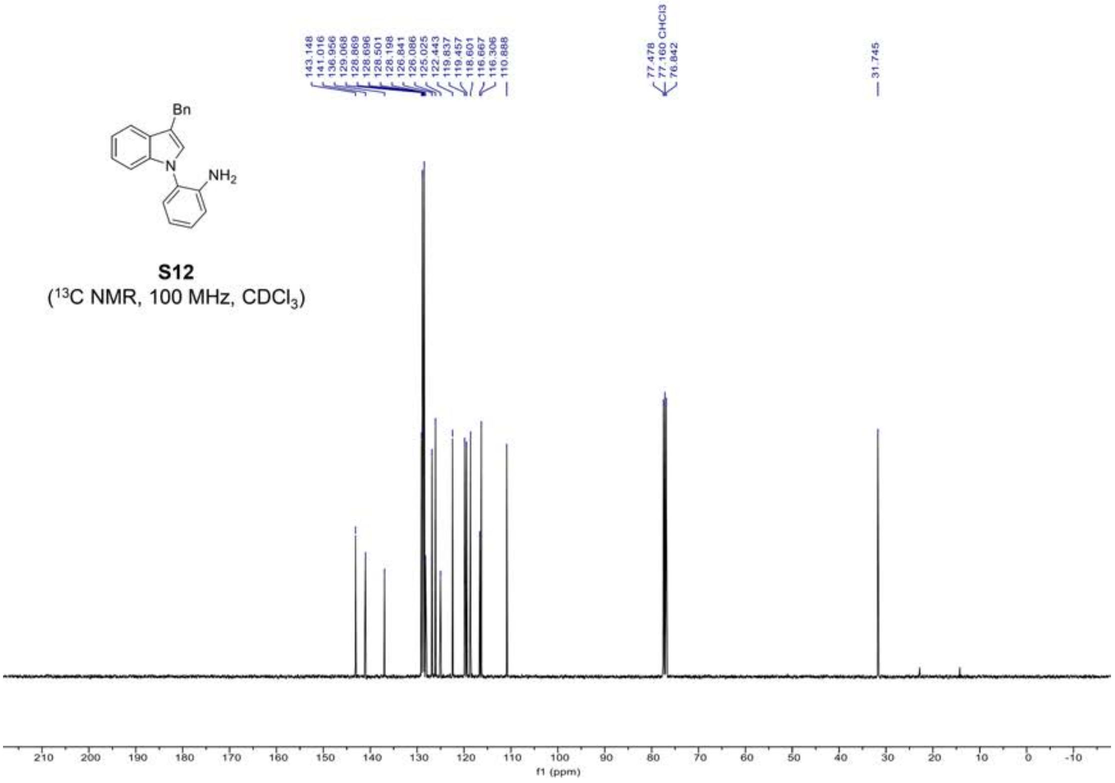

S12
(^{13}C NMR, 100 MHz, $CDCl_3$)

2.2 Synthesis of 1

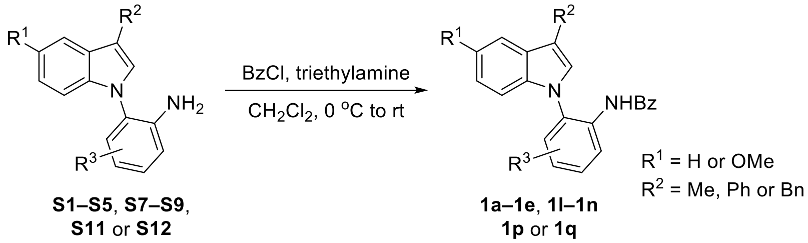

S1–S5, **S7–S9**, **S11** or **S12** → **1a–1e**, **1l–1n**, **1p** or **1q**

Procedure 2: To a round-bottom flask equipped with a magnetic stirring bar, **S1–S5**, **S7–S9**, **S11** or **S12**, (1.0 equiv), and triethylamine (2.0 equiv) were added and subsequently dissolved in CH_2Cl_2 (0.4 M). Then, benzoyl chloride (1.2 equiv) was added dropwise and allowed to stir for overnight at rt. The progress of the reaction was monitored by TLC. The reaction was diluted with CH_2Cl_2, transferred to a separatory funnel, and quenched with a saturated aqueous NH_4Cl. The organic layer was separated, and the aqueous layer was extracted an additional two times with CH_2Cl_2. The combined organic layers were then rinsed with water, dried with anhydrous $MgSO_4$, and concentrated *in vacuo*. The crude material was then purified by flash chromatography to afford the desired material **1a**–**1e**, **1l**–**1n**, **1p** or **1q**.

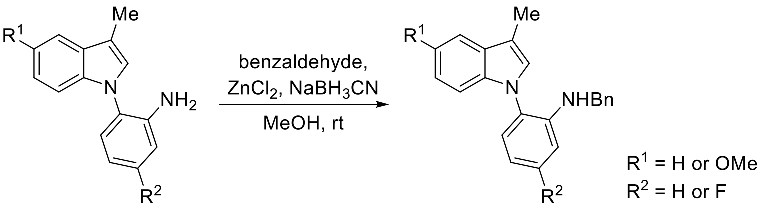

S1, S6 or **S10** → **1f, 1h** or **1o**

Procedure 3: To a round-bottom flask equipped with a magnetic stirring bar, benzaldehyde (1.0 equiv), $ZnCl_2$ (1.2 equiv) and **S1**, **S6** or **S10** (1.2 equiv) were added and subsequently dissolved in MeOH (0.25 M). Then $NaBH_3CN$ (1.1 equiv) was added, and the reaction mixture was stirred at rt for overnight. The progress of the reaction was monitored by TLC. The reaction mixture was quenched with 1 N aqueous NaOH and extracted three times with EtOAc. The combined organic layers were washed with brine, dried over $MgSO_4$, and concentrated *in vacuo*. The crude material was then purified by flash chromatography to afford the desired material **1f**, **1h** or **1o**.

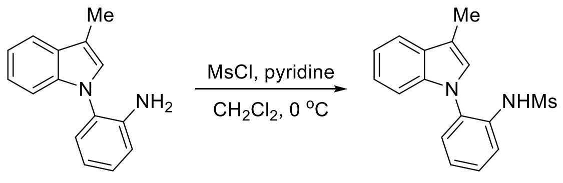

S1 → **1g**

Procedure 4: To a round-bottom flask equipped with a magnetic stirring bar, add methanesulfonyl chloride (1.2 equiv) to a cooled solution of **S1** (1.0 equiv) and pyridine (3.0 equiv) in anhydrous CH_2Cl_2 (0.5 M) and the reaction mixture was allowed to stir for 1 h at 0 °C. The progress of the reaction was monitored by TLC. Then, the reaction mixture was quenched with water and was extracted three times with CH_2Cl_2. The combined organic layers were washed with brine, dried with anhydrous $MgSO_4$, filtered

with CH_2Cl_2, and concentrated *in vacuo*. The crude material was then purified by flash chromatography to afford the desired material **1g**.

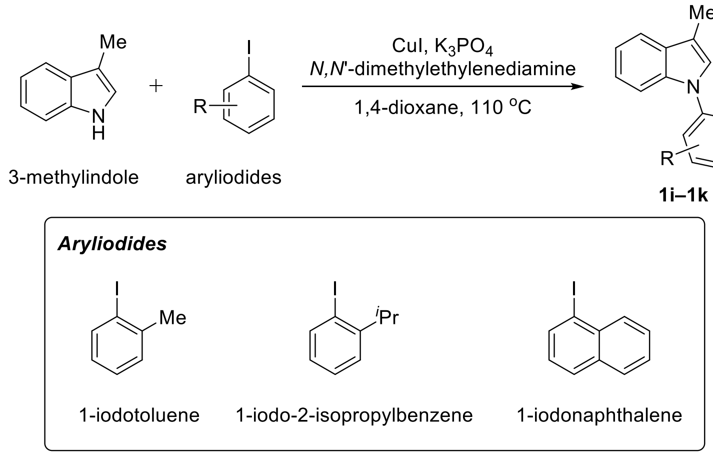

Procedure 5: To a reaction tube equipped with a magnetic stirring bar, 3-methylindole (1.0 equiv), aryliodides (1.4 equiv), copper iodide (0.3 equiv), *N,N'*-dimethylethylenediamine (1.4 equiv), and potassium phosphate (2.0 equiv) were added and subsequently dissolved in 1,4-dioxane (1.0 M). The reaction was heated to 110 °C and allowed to stir for overnight. The progress of the reaction was monitored by TLC. The reaction was diluted with EtOAc, transferred to a separatory funnel, and quenched with a saturated aqueous NH_4Cl. The organic layer was separated, and the aqueous layer was extracted an additional two times with EtOAc. The combined organic layers were then rinsed with water, dried with anhydrous $MgSO_4$, and concentrated *in vacuo*. The crude material was then purified by flash chromatography to afford the desired material **1i–1k**.

Characterization and Spectra of 1

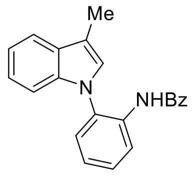

***N*-(2-(3-Methyl-1*H*-indol-1-yl)phenyl)benzamide (1a)** was synthesized by following Procedure 2 from **S1**. The crude material was purified by normal-phase column chromatography using an eluent of 50% CH_2Cl_2/Hx to provide **1a** (964 mg, 66%).

^{1}H NMR (400 MHz, $CDCl_3$) δ 8.64 (dd, *J* = 8.3, 1.4 Hz, 1H), 7.72–7.53 (m, 2H), 7.44 (td, *J* = 7.9, 1.6 Hz, 1H), 7.39–7.22 (m, 4H), 7.22–7.11 (m, 5H), 7.11–7.02 (m, 1H), 6.94 (s, 1H), 2.36 (d, *J* = 1.1 Hz, 3H).
^{13}C NMR (100 MHz, $CDCl_3$) δ 165.1, 136.9, 134.8, 134.3, 131.9, 129.3, 129.0, 128.8, 128.7 (2C), 128.0, 126.8 (2C), 126.1, 124.6, 123.2, 121.5, 120.4, 119.6, 113.9, 110.4, 9.7.
IR (FT-ATR, cm^{-1}, $CHCl_3$) ν_{max} 3984, 3950, 3926, 3903, 3865, 3838, 3799, 3776, 3730, 3699, 3413, 3317, 3059, 3016, 2920, 2889, 2858, 2704, 2650, 2584, 2441, 2360, 2337, 2295, 2214, 2137, 2075, 1955, 1921, 1894, 1801, 1770, 1674, 1593, 1520, 1454, 1365, 1304, 1250, 1223, 1161, 1126, 1072, 1014, 933, 891, 845, 741, 706, 683.
HRMS (EI) *m*/*z*: $[M]^+$ Calcd for $C_{22}H_{18}N_2O$ 326.1419; found 326.1420.

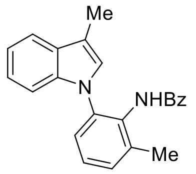

***N*-(2-Methyl-6-(3-methyl-1*H*-indol-1-yl)phenyl)benzamide (1b)** was synthesized by following Procedure 2 from **S2**. The crude material was purified by normal-phase column chromatography using an eluent of 20% EtOAc/Hx to provide **1b** (376 mg, 74%).

^{1}H NMR (400 MHz, $CDCl_3$) δ 7.76–7.66 (m, 1H), 7.57–7.33 (m, 9H), 7.31–7.22 (m, 3H), 7.06 (s, 1H), 2.49 (s, 3H), 2.39 (d, *J* = 1.2 Hz, 3H).
^{13}C NMR (100 MHz, $CDCl_3$) δ 166.8, 138.4, 137.1, 135.8, 134.3, 132.3, 131.6, 130.3, 129.2, 128.5 (2C), 127.8, 127.1 (2C), 126.5, 125.4, 122.6, 119.8, 119.3, 112.7, 110.0, 18.9, 9.6.
IR (FT-ATR, cm^{-1}, $CHCl_3$) ν_{max} 3988, 3926, 3899, 3838, 3799, 3776, 3730, 3699, 3626, 3599, 3545, 3525, 3275, 3124, 3055, 3012, 2920, 2885, 2862, 2738, 2650, 2600, 2465, 2403, 2360, 2333, 2295, 2268, 1959, 1894, 1770, 1647, 1585, 1512, 1481, 1365, 1304, 1223, 1165, 1142, 1095, 1076, 1022, 972, 922, 849, 787, 744, 717, 663.
HRMS (EI) *m*/*z*: $[M]^+$ Calcd for $C_{23}H_{20}N_2O$ 340.1576; found 340.1573.

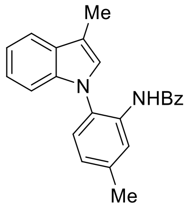

***N*-(5-Methyl-2-(3-methyl-1*H*-indol-1-yl)phenyl)benzamide (1c)** was synthesized by following Procedure 2 from **S3**. The crude material was purified by normal-phase column chromatography using an eluent of 20% EtOAc/Hx to provide **1c** (372 mg, 65%).

^{1}H NMR (400 MHz, $CDCl_3$) δ 8.54 (s, 1H), 7.75–7.65 (m, 1H), 7.58 (s, 1H), 7.41 (tt, *J* = 6.8, 1.8 Hz, 1H), 7.32–7.24 (m, 5H), 7.25–7.18 (m, 4H), 7.17–7.09 (m, 1H), 7.07 (d, *J* = 7.7 Hz, 1H), 6.99 (s, 1H), 2.50 (s, 3H), 2.42 (s, 3H).
^{13}C NMR (100 MHz, $CDCl_3$) δ 165.2, 139.5, 137.1, 134.6, 134.4, 131.9, 129.3, 128.8 (2C), 127.8, 126.9 (2C), 126.3, 126.3, 125.4, 123.1, 121.9, 120.3, 119.6, 113.8, 110.5, 21.8, 9.8.
IR (FT-ATR, cm^{-1}, $CHCl_3$) ν_{max} 3969, 3926, 3899, 3838, 3799, 3776, 3730, 3703, 3626, 3599, 3545, 3525, 3417, 3317, 3055, 2920, 2858, 2738, 2603, 2360, 2333, 2295, 2268, 1967, 1894, 1782, 1716, 1678, 1585, 1531, 1504, 1462, 1369, 1296, 1264, 1227, 1173, 1122, 1095, 1072, 1014, 930, 883, 818, 798, 744, 717, 706, 679.
HRMS (EI) *m*/*z*: $[M]^+$ Calcd for $C_{23}H_{20}N_2O$ 340.1576; found 340.1577.

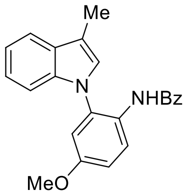

***N*-(4-Methoxy-2-(3-methyl-1*H*-indol-1-yl)phenyl)benzamide (1d)** was synthesized by following Procedure 2 from **S4**. The crude material was purified by normal-phase column chromatography using an eluent of 5% EtOAc/Hx to provide **1d** (523 mg, 92%).

^{1}H NMR (400 MHz, $CDCl_3$) δ 8.52 (d, *J* = 9.1 Hz, 1H), 7.76–7.66 (m, 1H), 7.52 (s, 1H), 7.45–7.37 (m, 1H), 7.37–7.31 (m, 2H), 7.31–7.22 (m, 4H), 7.22–7.15 (m, 1H), 7.08 (dd, *J* = 9.1, 2.9 Hz, 1H), 7.04 (s, 1H), 6.97 (d, *J* = 2.9 Hz, 1H), 3.86 (s, 3H), 2.50–2.43 (s, 3H).
^{13}C NMR (100 MHz, $CDCl_3$) δ 165.2, 156.6, 136.8, 134.5, 131.8, 130.4, 129.4, 128.7 (2C), 127.8, 126.9 (2C), 126.1, 123.4, 123.2, 120.4, 119.6, 114.2, 113.9, 113.4, 110.5, 55.8, 9.7.
IR (FT-ATR, cm^{-1}, $CHCl_3$) ν_{max} 3969, 3950, 3930, 3907, 3880, 3841, 3822, 3803, 3780, 3757, 3718, 3676, 3652, 3629, 3614, 3591, 3568, 3545, 3417, 3305, 3055, 3008, 2935, 2862, 2835, 2750, 2627, 2576, 2472, 2372, 2345, 2318, 2252, 2210, 2125, 2063, 2017, 1991, 1963, 1917, 1894, 1747, 1666, 1601, 1516, 1458, 1362, 1304, 1273, 1254, 1211, 1176, 1134, 1099, 1065, 1034, 968, 930, 895, 852, 810, 741, 717, 706, 663.
HRMS (EI) *m*/*z*: $[M]^+$ Calcd for $C_{23}H_{20}N_2O_2$ 356.1525; found 356.1524.

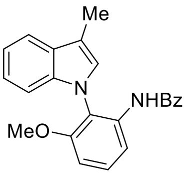

N-(3-Methoxy-2-(3-methyl-1H-indol-1-yl)phenyl)benzamide (1e) was synthesized by following Procedure 2 from **S5**. The crude material was purified by normal-phase column chromatography using an eluent of 5% EtOAc/Hx to provide **1e** (396 mg, 97%).

^{1}H NMR (400 MHz, $CDCl_3$) δ 8.35 (dd, J = 8.4, 1.2 Hz, 1H), 7.81–7.63 (m, 1H), 7.60–7.44 (m, 2H), 7.39 (ddt, J = 7.4, 6.5, 1.8 Hz, 1H), 7.32–7.12 (m, 6H), 7.04 (ddd, J = 7.9, 3.0, 1.6 Hz, 1H), 6.96 (q, J = 1.2 Hz, 1H), 6.88 (dd, J = 8.5, 1.2 Hz, 1H), 3.76 (s, 3H), 2.44 (d, J = 1.2 Hz, 3H).
^{13}C NMR (100 MHz, $CDCl_3$) δ 165.1, 156.4, 137.0, 136.8, 134.4, 131.9, 129.9, 129.1, 128.7 (2C), 126.8, 126.4, 123.0, 120.2, 119.5, 117.1, 113.5, 113.0, 110.4, 107.4, 56.1, 9.9.
IR (FT-ATR, cm^{-1}, $CHCl_3$) ν_{max} 3980, 3907, 3884, 3845, 3807, 3780, 3757, 3718, 3676, 3656, 3629, 3595, 3572, 3417, 3190, 3055, 3012, 2962, 2935, 2862, 2839, 2750, 2549, 2438, 2372, 2345, 2318, 2237, 2133, 1990, 1963, 1925, 1894, 1801, 1774, 1682, 1597, 1531, 1462, 1389, 1362, 1292, 1261, 1223, 1184, 1126, 1068, 1011, 968, 930, 887, 845, 741, 706, 663.
HRMS (EI) m/z: $[M]^+$ Calcd for $C_{23}H_{20}N_2O_2$ 356.1525; found 356.1522.

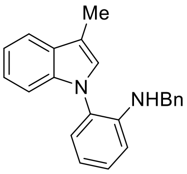

N-Benzyl-2-(3-methyl-1H-indol-1-yl)aniline (1f) was synthesized by following Procedure 3 from **S1**. The crude material was purified by normal-phase column chromatography using an eluent of 20% EtOAc/Hx to provide **1f** (649 mg, 69%).

^{1}H NMR (400 MHz, $CDCl_3$) δ 7.79–7.50 (m, 1H), 7.49–7.09 (m, 10H), 7.03 (q, J = 1.2 Hz, 1H), 6.92–6.62 (m, 2H), 4.33 (s, 2H), 4.19 (s, 1H), 2.44 (d, J = 1.1 Hz, 3H).
^{13}C NMR (100 MHz, $CDCl_3$) δ 144.5, 139.2, 137.0, 129.2, 129.1, 128.7 (2C), 128.6, 127.2, 127.0 (2C), 126.4, 125.0, 122.3, 119.7, 119.2, 117.0, 112.7, 111.8, 110.8, 47.6, 9.8.
IR (FT-ATR, cm^{-1}, $CHCl_3$) ν_{max} 3894, 3907, 3884, 3842, 3818, 3784, 3757, 3734, 3699, 3656, 3633, 3595, 3421, 3182, 3055, 3032, 2916, 2885, 2858, 2623, 2526, 2461, 2418, 2360, 2337, 2133, 1925, 1886, 1801, 1778, 1689, 1651, 1601, 1516, 1454, 1365, 1304, 1227, 1161, 1130, 1068, 1014, 991, 930, 845, 795, 737, 698.
HRMS (EI) m/z: $[M]^+$ Calcd for $C_{22}H_{20}N_2$ 312.1627; found 312.1625.

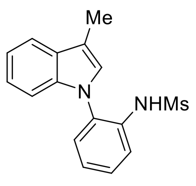

***N*-(2-(3-Methyl-1*H*-indol-1-yl)phenyl)methanesulfonamide (1g)** was synthesized by following Procedure 4 from **S1**. The crude material was purified by normal-phase column chromatography using an eluent of 25% EtOAc/Hx to provide **1g** (259 mg, 58%).

^{1}H NMR (400 MHz, $CDCl_3$) δ 7.80 (dd, *J* = 8.2, 1.4 Hz, 1H), 7.73–7.59 (m, 1H), 7.48 (ddd, *J* = 8.4, 7.3, 1.9 Hz, 1H), 7.41–7.24 (m, 2H), 7.27–7.10 (m, 2H), 7.09–6.96 (m, 1H), 6.93 (q, *J* = 1.2 Hz, 1H), 6.31 (s, 1H), 2.80 (s, 3H), 2.41 (d, *J* = 1.2 Hz, 3H).
^{13}C NMR (100 MHz, $CDCl_3$) δ 137.2, 134.1, 129.7, 129.6, 129.4, 129.2, 125.54, 125.51, 123.3, 120.7, 120.5, 119.8, 114.5, 109.8, 39.8, 9.8.
IR (FT-ATR, cm^{-1}, $CHCl_3$) ν_{max} 3977, 3930, 3907, 3884, 3842, 3826, 3807, 3780, 3757, 3714, 3680, 3653, 3629, 3595, 3572, 3552, 3529, 3506, 3487, 3448, 3336, 3278, 3024, 2924, 2889, 2862, 2742, 2565, 2480, 2372, 2349, 2310, 2245, 2091, 1925, 1894, 1845, 1801, 1778, 1693, 1655, 1593, 1504, 1454, 1392, 1369, 1331, 1281, 1231, 1153, 1068, 1040, 1011, 968, 903, 814, 737, 714, 663.
HRMS (EI) *m*/*z*: $[M]^+$ Calcd for $C_{16}H_{16}N_2O_2S$ 300.0933; found 300.0930.

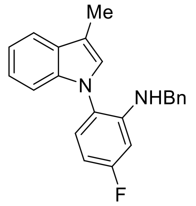

***N*-Benzyl-5-fluoro-2-(3-methyl-1*H*-indol-1-yl)aniline (1h)** was synthesized by following Procedure 3 from **S6**. The crude material was purified by normal-phase column chromatography using an eluent of 1% EtOAc/Hx to provide **1h** (130 mg, 23%).

^{1}H NMR (400 MHz, $CDCl_3$) δ 7.64 (dd, *J* = 6.5, 1.4 Hz, 1H), 7.34–7.27 (m, 2H), 7.25–7.15 (m, 5H), 7.14–7.06 (m, 2H), 6.95 (d, *J* = 1.3 Hz, 1H), 6.48–6.38 (m, 2H), 4.27 (s, 2H), 2.39 (d, *J* = 1.2 Hz, 3H).
^{13}C NMR (100 MHz, $CDCl_3$) δ 163.6 (d, *J* = 242.0 Hz, 1C), 146.2 (d, *J* = 12.0 Hz, 1C), 138.4, 137.2, 129.8 (d, *J* = 11.0 Hz, 1C), 129.2, 128.8 (2C), 127.5, 127.0 (2C), 126.4, 122.5, 120.9 (d, *J* = 2.0 Hz, 1C), 119.8, 119.2, 112.9, 110.6, 103.2 (d, *J* = 23.0 Hz, 1C), 98.9 (d, *J* = 28.0 Hz, 1C), 47.5, 9.8.
IR (FT-ATR, cm^{-1}, $CHCl_3$) ν_{max} 3946, 3926, 3899, 3838, 3799, 3780, 3730, 3703, 3626, 3599, 3545, 3525, 3506, 3421, 3224, 3182, 3059, 3032, 2920, 2889, 2858, 2360, 2333, 2299, 2268, 2017, 1990, 1944, 1921, 1890, 1867, 1801, 1774, 1747, 1716, 1678, 1616, 1523, 1458, 1304, 1230, 1169, 1119, 1072, 1011, 957, 926, 829, 795, 744, 698.
HRMS (EI) *m*/*z*: $[M]^+$ Calcd for $C_{22}H_{19}FN_2$ 330.1532; found 330.1533.

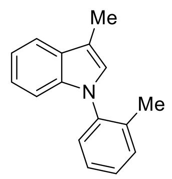

3-Methyl-1-(*o*-tolyl)-1*H*-indole (1i) was synthesized by following Procedure 5. The crude material was purified by normal-phase column chromatography using an eluent of 5% EtOAc/Hx to provide **1i** (1.92 g, 87%).

^{1}H NMR (400 MHz, $CDCl_3$) δ 7.93–7.87 (m, 1H), 7.62–7.50 (m, 4H), 7.44–7.38 (m, 2H), 7.29–7.24 (m, 1H), 7.18 (q, *J* = 1.1 Hz, 1H), 2.66 (s, 3H), 2.33 (s, 3H).
^{13}C NMR (100 MHz, $CDCl_3$) δ 138.5, 137.3, 135.8, 131.3, 128.8, 128.2, 128.0, 126.8, 126.4, 122.1, 119.3, 119.1, 111.7, 110.5, 17.9, 9.8.

The spectral data were identical with those previously reported.[4]

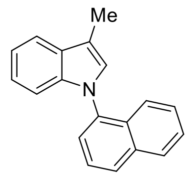

3-Methyl-1-(naphthalen-1-yl)-1*H*-indole (1j) was synthesized by following Procedure 5. The crude material was purified by normal-phase column chromatography using an eluent of 5% EtOAc/Hx to provide **1j** (221 mg, 57%).

^{1}H NMR (400 MHz, $CDCl_3$) δ 7.95–7.86 (m, 2H), 7.72 (dt, *J* = 7.9, 1.0 Hz, 1H), 7.58–7.46 (m, 4H), 7.36 (ddd, *J* = 8.3, 6.8, 1.3 Hz, 1H), 7.21 (ddd, *J* = 7.9, 7.0, 1.2 Hz, 1H), 7.17–7.10 (m, 2H), 7.03 (dt, *J* = 8.1, 1.0 Hz, 1H), 2.47 (d, *J* = 1.3 Hz, 3H).
^{13}C NMR (100 MHz, $CDCl_3$) δ 138.3, 136.3, 134.6, 130.6, 129.0, 128.3, 128.2, 127.5, 126.9, 126.6, 125.6, 125.0, 123.7, 122.2, 119.6, 119.1, 112.2, 110.8, 9.8.

The spectral data were identical with those previously reported.[5]

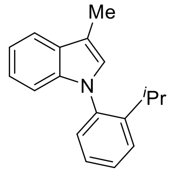

1-(2-Isopropylphenyl)-3-methyl-1*H*-indole (1k) was synthesized by following Procedure 5. The crude material was purified by normal-phase column chromatography using an eluent of 5% EtOAc/Hx to provide **1k** (428 mg, 57%).

^{1}H NMR (400 MHz, $CDCl_3$) δ 7.74–7.68 (m, 1H), 7.57–7.47 (m, 2H), 7.35 (td, *J* = 7.4, 1.7 Hz, 1H), 7.29 (dd, *J* = 7.9, 1.6 Hz, 1H), 7.25–7.19 (m, 2H), 7.10–7.05 (m, 1H), 7.01 (q, *J* = 1.1 Hz, 1H), 2.81 (hept, *J* = 6.9 Hz, 1H), 2.49 (d, *J* = 1.2 Hz, 3H), 1.21 (d, *J* = 6.9 Hz, 3H), 1.16 (d, *J* = 6.9 Hz, 3H).
^{13}C NMR (100 MHz, $CDCl_3$) δ 147.1, 138.3, 137.1, 128.9, 128.8, 128.6, 127.0, 126.8, 126.6, 122.1, 119.2, 119.0, 111.6, 110.4, 27.8, 24.8, 23.7, 9.8.

The spectral data were identical with those previously reported.[5]

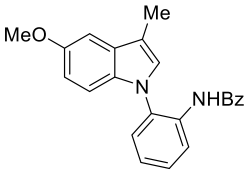

***N*-(2-(5-Methoxy-3-methyl-1*H*-indol-1-yl)phenyl)benzamide (1l)** was synthesized by following Procedure 2 from **S7**. The crude material was purified by normal-phase column chromatography using an eluent of 13% EtOAc/Hx to provide **1l** (461 mg, 86%).

^{1}H NMR (400 MHz, $CDCl_3$) δ 8.70 (dd, *J* = 8.3, 1.4 Hz, 1H), 7.70 (s, 1H), 7.60–7.47 (m, 1H), 7.47–7.40 (m, 1H), 7.37 (ddd, *J* = 8.6, 4.9, 1.6 Hz, 3H), 7.33–7.20 (m, 3H), 7.14 (d, *J* = 2.5 Hz, 1H), 7.10–6.95 (m, 2H), 6.89 (d d, *J* = 8.8, 2.5 Hz, 1H), 3.92 (s, 3H), 2.40 (d, *J* = 1.1 Hz, 3H).
^{13}C NMR (100 MHz, $CDCl_3$) δ 165.2, 154.8, 134.9, 134.4, 132.2, 131.9, 129.9, 129.02, 128.97, 128.82, 128.77, 128.0, 126.91, 126.89, 124.6, 121.5, 113.6, 113.1, 111.3, 101.6, 56.1, 9.8.
IR (FT-ATR, cm^{-1}, $CHCl_3$) ν_{max} 3973, 3953, 3930, 3907, 3888, 3807, 3757, 3718, 3680, 3656, 3629, 3595, 3572, 3548, 3525, 3502, 3413, 3340, 3186, 3062, 3012, 2935, 2831, 2738, 2573, 2434, 2372, 2349, 2314, 2249, 2210, 2087, 1955, 1921, 1840, 1790, 1678, 1589, 1520, 1454, 1392, 1373, 1308, 1250, 1215, 1176, 1149, 1119, 1061, 1030, 945, 875, 833, 748, 706, 663.
HRMS (EI) *m*/*z*: $[M]^+$ Calcd for $C_{23}H_{20}N_2O_2$ 356.1525; found 356.1522.

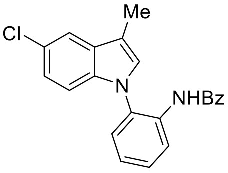

***N*-(2-(5-Chloro-3-methyl-1*H*-indol-1-yl)phenyl)benzamide (1m)** was synthesized by following Procedure 2 from **S8**. The crude material was purified by normal-phase column chromatography using an eluent of 20% EtOAc/Hx to provide **1m** (364.7 mg, 67%).

^{1}H NMR (400 MHz, $CDCl_3$) δ 8.67 (dd, *J* = 8.3, 1.4 Hz, 1H), 7.66 (d, *J* = 2.0 Hz, 1H), 7.62–7.48 (m, 2H), 7.48–7.40 (m, 1H), 7.40–7.22 (m, 6H), 7.17 (dd, *J* = 8.7, 2.0 Hz, 1H), 7.10–6.96 (m, 2H), 2.38 (d, *J* = 1.2 Hz, 3H).
^{13}C NMR (100 MHz, $CDCl_3$) δ 165.2, 135.5, 134.9, 134.3, 132.1, 130.4, 129.5, 128.9 (2C), 128.4, 128.0, 127.5, 126.9, 126.3, 124.8, 123.4, 121.8, 119.2, 113.6, 111.6, 9.65.
IR (FT-ATR, cm^{-1}, $CHCl_3$) ν_{max} 3950, 3930, 3903, 3884, 3845, 3803, 3780, 3757, 3718, 3676, 3653, 3633, 3614, 3691, 3568, 3421, 3305, 3062, 3016, 2974, 2920, 2862, 2731, 2742, 2607, 2561, 2488, 2430, 2372, 2345, 2318, 2206, 2141, 2079, 1959, 1917, 1847, 1805, 1743, 1674, 1593, 1520, 1450, 1369, 1304, 1246, 1219, 1149, 1126, 1095, 1049, 999, 937, 891, 864, 837, 791, 748, 717, 706.
HRMS (EI) *m*/*z*: $[M]^+$ Calcd for $C_{22}H_{17}ClN_2O$ 360.1029; found 360.1029.

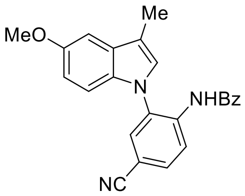

***N*-(4-Cyano-2-(5-methoxy-3-methyl-1*H*-indol-1-yl)phenyl)benzamide (1n)** was synthesized by following Procedure 2 from **S9**. The crude material was purified by normal-phase column chromatography using an eluent of 13% EtOAc/Hx to provide **1n** (148 mg, 47%).

^{1}H NMR (400 MHz, $CDCl_3$) δ 8.91 (d, *J* = 8.7 Hz, 1H), 7.89 (s, 1H), 7.77 (dd, *J* = 8.6, 2.0 Hz, 1H), 7.66 (d, *J* = 2.0 Hz, 1H), 7.46 (ddt, *J* = 8.7, 6.7, 2.0 Hz, 1H), 7.37–7.27 (m, 4H), 7.13 (d, *J* = 2.4 Hz, 1H), 6.98 (d, *J* = 8.9 Hz, 1H), 6.96 (d, *J* = 1.3 Hz, 1H), 6.91 (dd, *J* = 8.9, 2.4 Hz, 1H), 3.91 (s, 3H), 2.39 (d, *J* = 1.1 Hz, 3H).
^{13}C NMR (100 MHz, $CDCl_3$) δ 165.3, 155.3, 139.3, 133.5, 133.1, 132.6, 131.9, 131.8, 130.4, 129.1, 129.0 (2C), 127.0 (2C), 126.3, 121.3, 118.1, 115.2, 113.7, 111.1, 107.5, 102.0, 56.1, 9.8.
IR (FT-ATR, cm^{-1}, $CHCl_3$) ν_{max} 3988, 3946, 3926, 3899, 3880, 3838, 3799, 3780, 3730, 3703, 3626, 3599, 3545, 3529, 3406, 3186, 3062, 3012, 2935, 2862, 2835, 2580, 2441, 2360, 2333, 2295, 2268, 2229, 2179, 2060, 1967, 1917, 1867, 1774, 1685, 1581, 1512, 1473, 1365, 1308, 1254, 1176, 1146, 1119, 1061, 1030, 972, 895, 837, 795, 756, 706, 671.
HRMS (EI) *m/z*: $[M]^+$ Calcd for $C_{24}H_{19}N_3O_2$ 381.1477; found 381.1474.

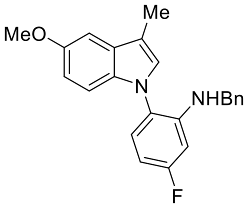

***N*-Benzyl-5-fluoro-2-(5-methoxy-3-methyl-1*H*-indol-1-yl)aniline (1o)** was synthesized by following Procedure 3 from **S10**. The crude material was purified by normal-phase column chromatography using an eluent of 1% EtOAc/Hx to provide **1o** (140 mg, 42%).

^{1}H NMR (400 MHz, $CDCl_3$) δ 7.29–7.21 (m, 2H), 7.21–7.10 (m, 3H), 7.07–7.69 (m, 2H), 6.95 (d, *J* = 8.8 Hz, 1H), 6.87 (d, *J* = 1.3 Hz, 1H), 6.83 (dd, *J* = 8.8, 2.5 Hz, 1H), 6.37 (ddd, *J* = 10.5, 6.0, 2.3 Hz, 2H), 4.18 (s, 2H), 3.84 (s, 3H), 2.32 (d, *J* = 1.1 Hz, 3H).
^{13}C NMR (100 MHz, $CDCl_3$) δ 163.5 (d, *J* = 242.0 Hz, 1C), 154.5, 146.2 (d, *J* = 12.0 Hz, 1C), 138.4, 132.4, 129.7 (d, *J* = 11.0 Hz, 1C), 129.5, 128.8 (2C), 127.4, 127.03, 126.97 (2C), 121.0 (d, *J* = 2.0 Hz, 1C), 112.5, 112.4, 111.4, 103.1 (d, *J* = 23.0 Hz, 1C), 101.1, 98.9 (d, *J* = 27.0 Hz, 1H), 56.0, 47.4, 9.8.
IR (FT-ATR, cm^{-1}, $CHCl_3$) ν_{max} 3965, 3926, 3899, 3880, 3865, 3842, 3799, 3776, 3730, 3699, 3626, 3599, 3545, 3421, 3062, 3008, 2927, 2858, 2835, 2738, 2600, 2461, 2360, 2333, 2299, 2268, 2087, 1959, 1863, 1801, 1697, 1616, 1520, 1477, 1450, 1354, 1304, 1242, 1215, 1169, 1115, 1061, 1026, 991, 957, 933, 864, 829, 791, 748, 694, 667.
HRMS (EI) *m/z*: $[M]^+$ Calcd for $C_{23}H_{21}FN_2O$ 360.1638; found 360.1637.

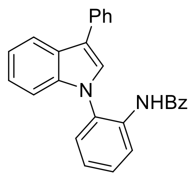

***N*-(2-(3-Phenyl-1*H*-indol-1-yl)phenyl)benzamide (1p)** was synthesized by following Procedure 2 from **S11**. The crude material was purified by normal-phase column chromatography using an eluent of 50% CH_2Cl_2/Hx to provide **1p** (128 mg, 64%).

^{1}H NMR (400 MHz, $CDCl_3$) δ 8.73 (dd, *J* = 8.4, 1.4 Hz, 1H), 8.08 (ddt, *J* = 7.0, 3.3, 1.7 Hz, 1H), 7.76–7.69 (m, 2H), 7.66 (s, 1H), 7.58 (ddd, *J* = 8.7, 7.6, 1.6 Hz, 1H), 7.49 (ddd, *J* = 7.8, 4.6, 3.0 Hz, 3H), 7.43 (s, 1H), 7.43–7.38 (m, 1H), 7.33 (tdd, *J* = 9.5, 7.5, 4.7 Hz, 6H), 7.29–7.19 (m, 3H).
^{13}C NMR (100 MHz, $CDCl_3$) δ 165.3, 137.5, 134.9, 134.7, 134.2, 132.1, 129.7, 129.1 (2C), 128.8 (2C), 128.4, 128.2, 127.7 (2C), 126.9 (2C), 126.72, 126.70, 126.0, 124.8, 123.7, 121.8, 121.6, 120.7, 120.2, 111.0.
IR (FT-ATR, cm^{-1}, $CHCl_3$) ν_{max} 3983, 3950, 3904, 3858, 3839, 3822, 3802, 3771, 3752, 3727, 3704, 3623, 3600, 3568, 3545, 3420, 3305, 3115, 3059, 3026, 2921, 2850, 2655, 2597, 2400, 2359, 2349, 2338, 2327, 2295, 1955, 1924, 1895, 1810, 1773, 1670, 1594, 1550, 1523, 1496, 1457, 1376, 1306, 1279, 1254, 1239, 1212, 1173, 1159, 1141, 1121, 1101, 1072, 1028, 1016, 1001, 975, 946, 927, 909, 891, 846, 812, 794, 741, 696, 664.
HRMS (EI) *m*/*z*: $[M]^+$ Calcd for $C_{27}H_{20}N_2O$ 388.1576; found 388.1573.

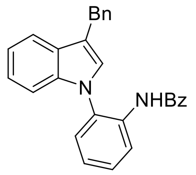

***N*-(2-(3-Benzyl-1*H*-indol-1-yl)phenyl)benzamide (1q)** was synthesized by following Procedure 2 from **S12**. The crude material was purified by normal-phase column chromatography using an eluent of 13% EtOAc/Hx to provide **1q** (155 mg, 19%).

^{1}H NMR (400 MHz, $CDCl_3$) δ 8.69 (dd, *J* = 8.2, 1.4 Hz, 1H), 7.70–7.60 (m, 2H), 7.52 (ddd, *J* = 8.7, 7.6, 1.6 Hz, 1H), 7.48–7.38 (m, 2H), 7.36–7.13 (m, 13H), 6.96 (s, 1H), 4.20 (s, 2H).
^{13}C NMR (100 MHz, $CDCl_3$) δ 165.2, 140.5, 137.3, 134.9, 134.3, 132.0, 129.3, 128.80 (2C), 128.75 (2C), 128.62, 128.60 (2C), 128.4, 128.1, 126.9 (2C), 126.6, 126.3, 124.6, 123.4, 121.6, 120.7, 119.9, 118.2, 110.6, 31.7.
IR (FT-ATR, cm^{-1}, $CHCl_3$) ν_{max} 3983, 3950, 3933, 3924, 3904, 3856, 3839, 3822, 3802, 3783, 3771, 3752, 3727, 3704, 3692, 3677, 3650, 3630, 3623, 3600, 3569, 3545, 3420, 3305, 3115, 3059, 3026, 2921, 2850, 2655, 2597, 2400, 2359, 2349, 2338, 2327, 2295, 1955, 1924, 1895, 1810, 1773, 1680, 1670, 1594, 1550, 1523, 1496, 1457, 1376, 1306, 1279, 1254, 1239, 1212, 1173, 1159, 1141, 1121, 1101, 1072, 1028, 1016, 1001, 975, 946, 927, 909, 891, 846, 812, 794, 741, 696, 664.
HRMS (EI) *m*/*z*: $[M]^+$ Calcd for $C_{28}H_{22}N_2O$ 402.1732; found 402.1732.

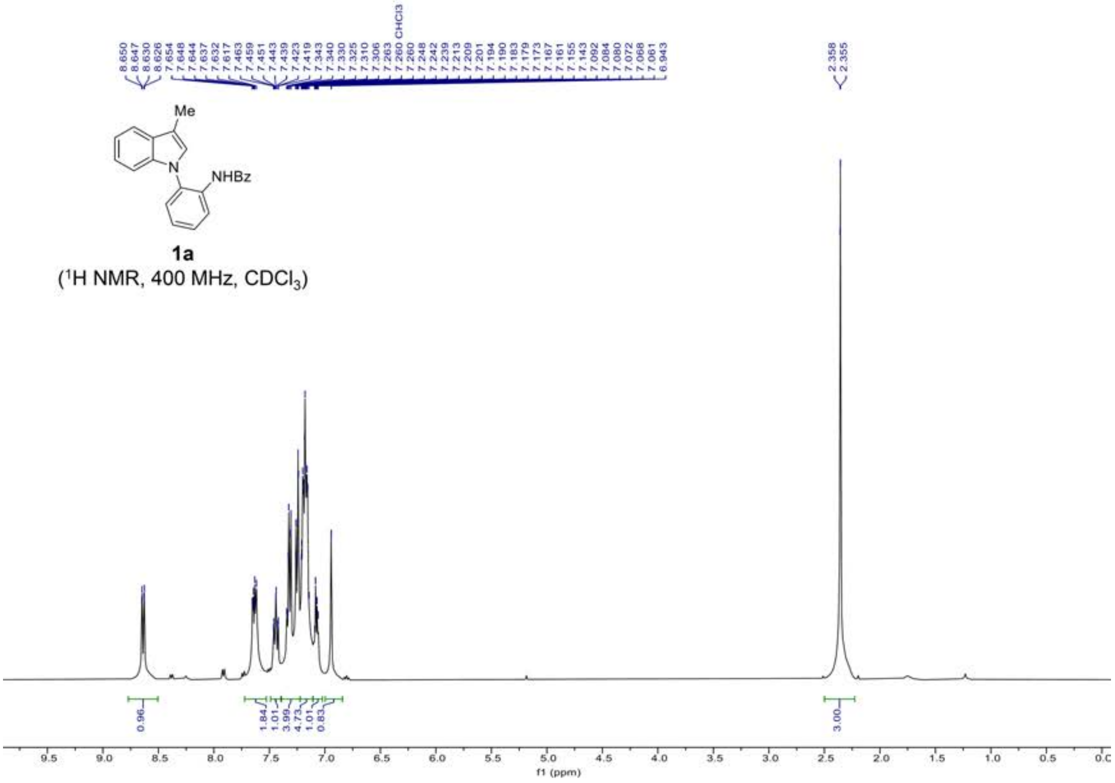
8.650
8.647
8.630
8.626
7.654
7.648
7.644
7.637
7.632
7.617
7.463
7.459
7.451
7.443
7.439
7.423
7.419
7.343
7.340
7.330
7.325
7.310
7.306
7.263
7.260 CHCl3
7.260
7.248
7.242
7.239
7.213
7.209
7.201
7.194
7.190
7.183
7.179
7.173
7.167
7.161
7.155
7.143
7.092
7.084
7.080
7.072
7.068
7.061
6.943
2.358
2.355
Me
N
NHBz
1a
(¹H NMR, 400 MHz, CDCl₃)
0.96
1.84
1.01
3.99
4.73
1.01
0.83
3.00
f1 (ppm)

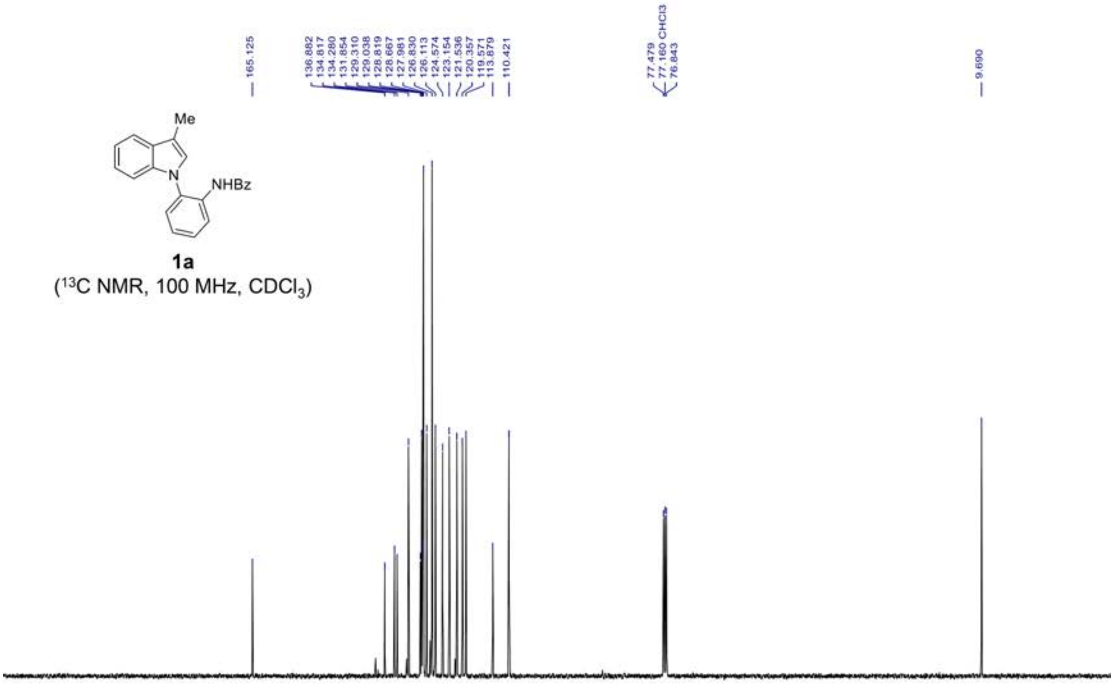
165.125
136.682
134.817
134.280
131.854
129.310
129.038
128.819
128.667
127.981
126.830
126.113
124.574
123.154
121.536
120.357
119.571
113.879
110.421
77.479
77.160 CHCl3
76.843
9.690
Me
N
NHBz
1a
(¹³C NMR, 100 MHz, CDCl₃)
f1 (ppm)

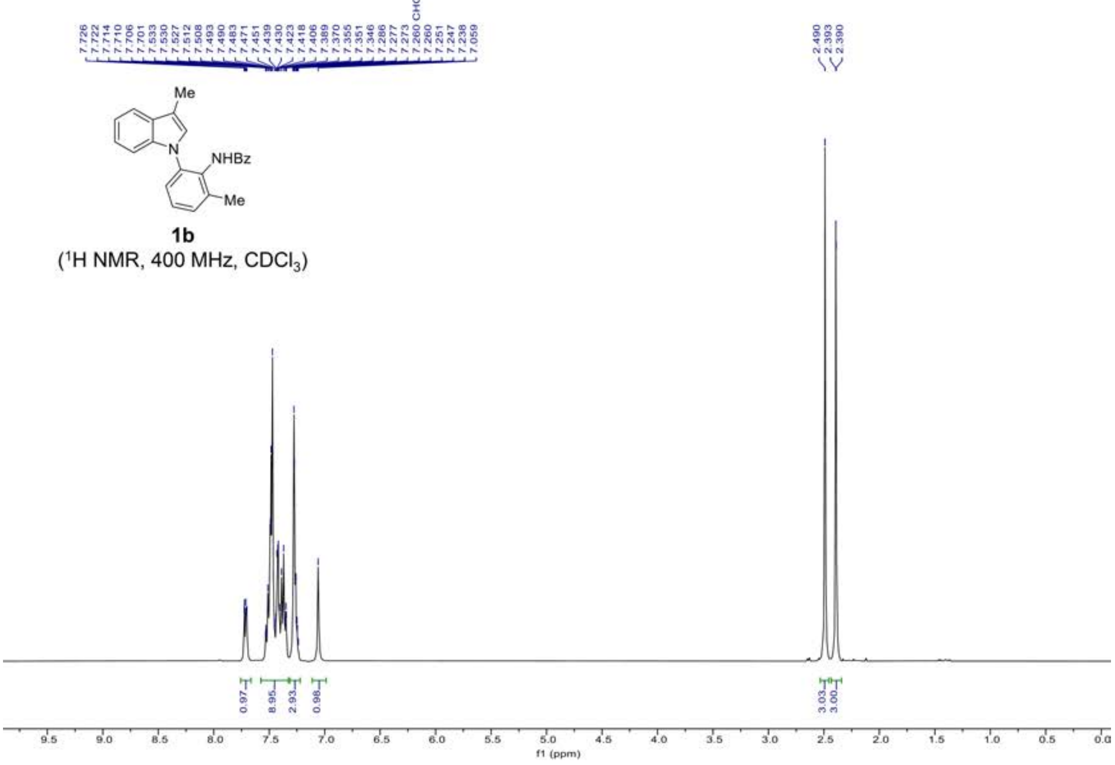
Me
NHBz
Me
1b
(1H NMR, 400 MHz, CDCl3)
f1 (ppm)

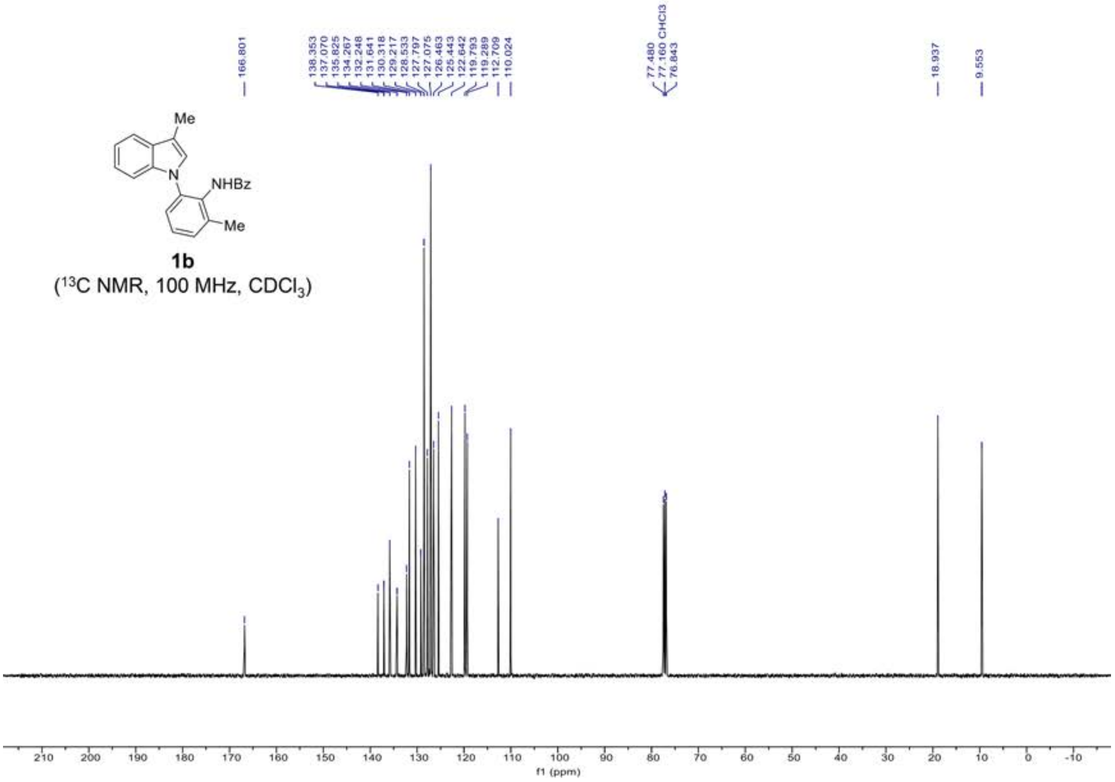
Me
NHBz
Me
1b
(13C NMR, 100 MHz, CDCl3)
f1 (ppm)

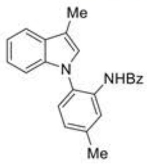

1c
(^{1}H NMR, 400 MHz, $CDCl_3$)

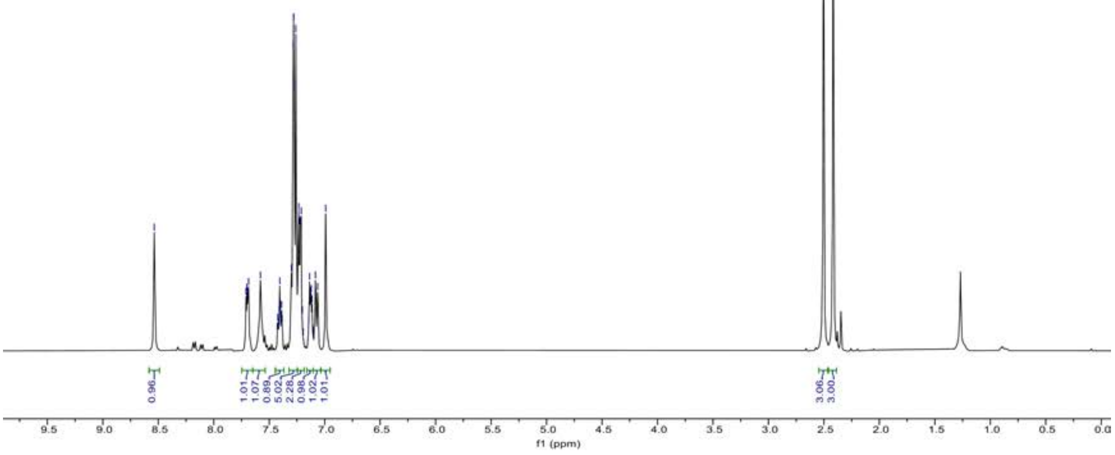

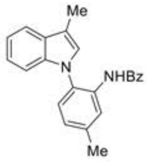

1c
(^{13}C NMR, 100 MHz, $CDCl_3$)

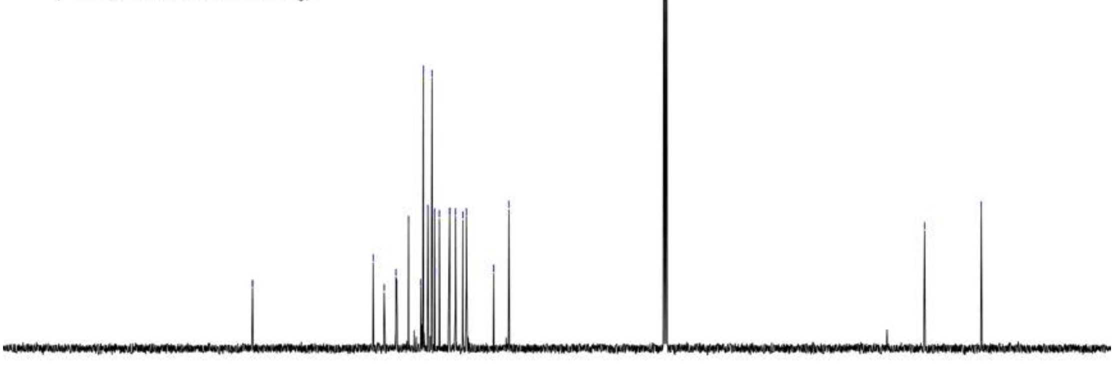

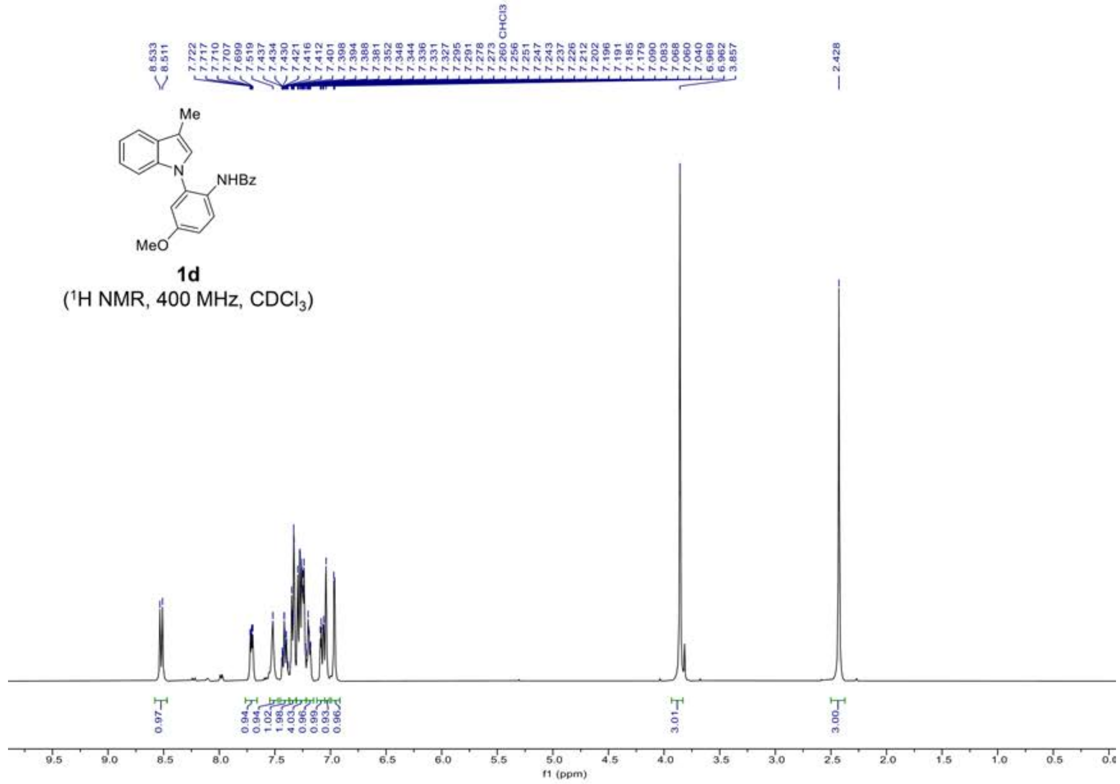

1d
(^1H NMR, 400 MHz, $CDCl_3$)

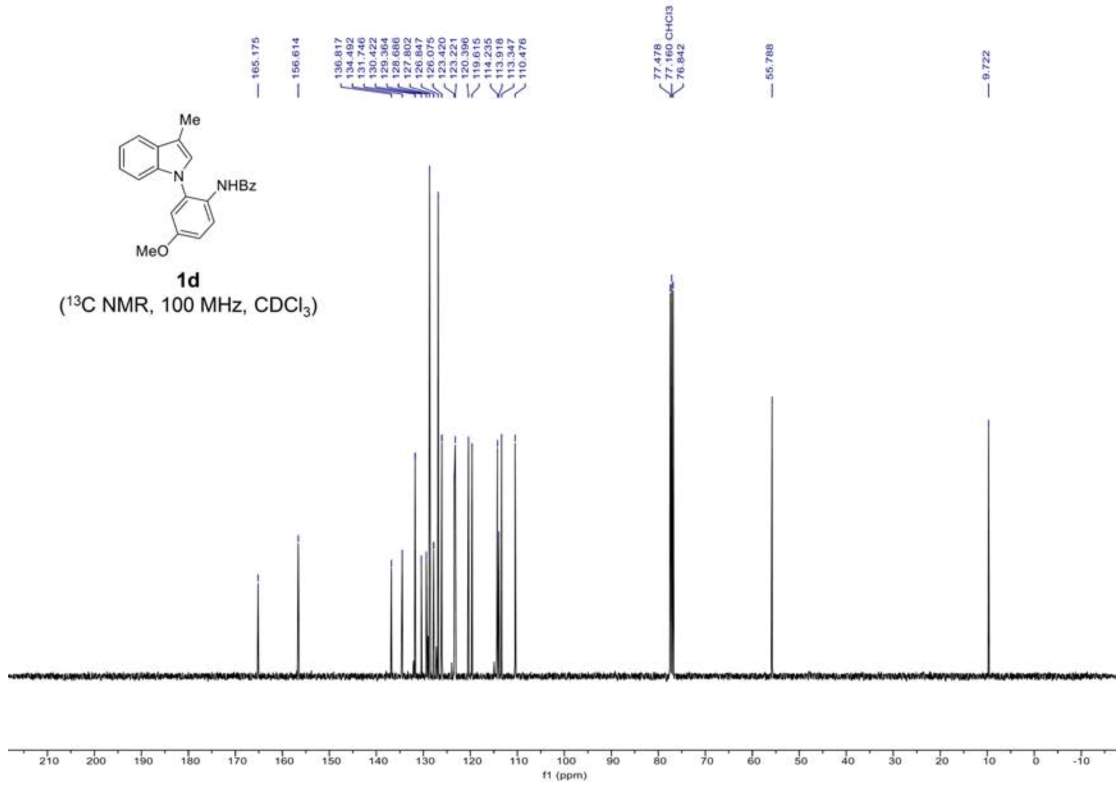

1d
(^{13}C NMR, 100 MHz, $CDCl_3$)

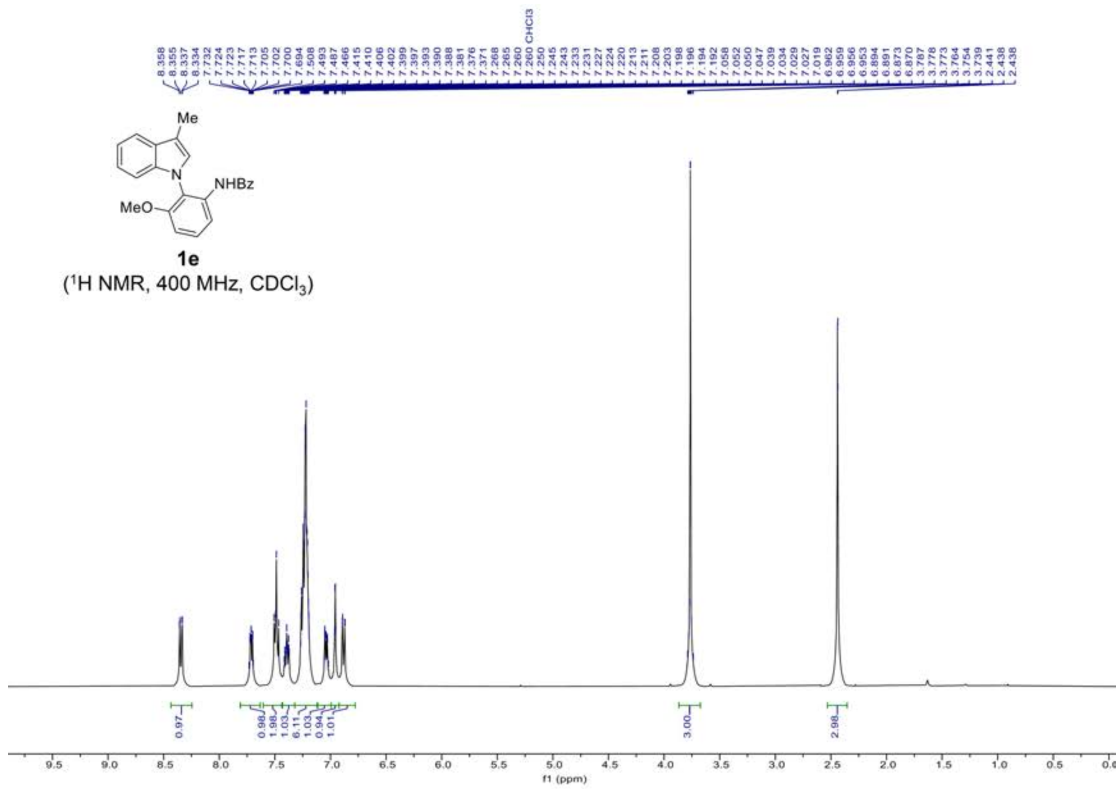

1e
(1H NMR, 400 MHz, $CDCl_3$)

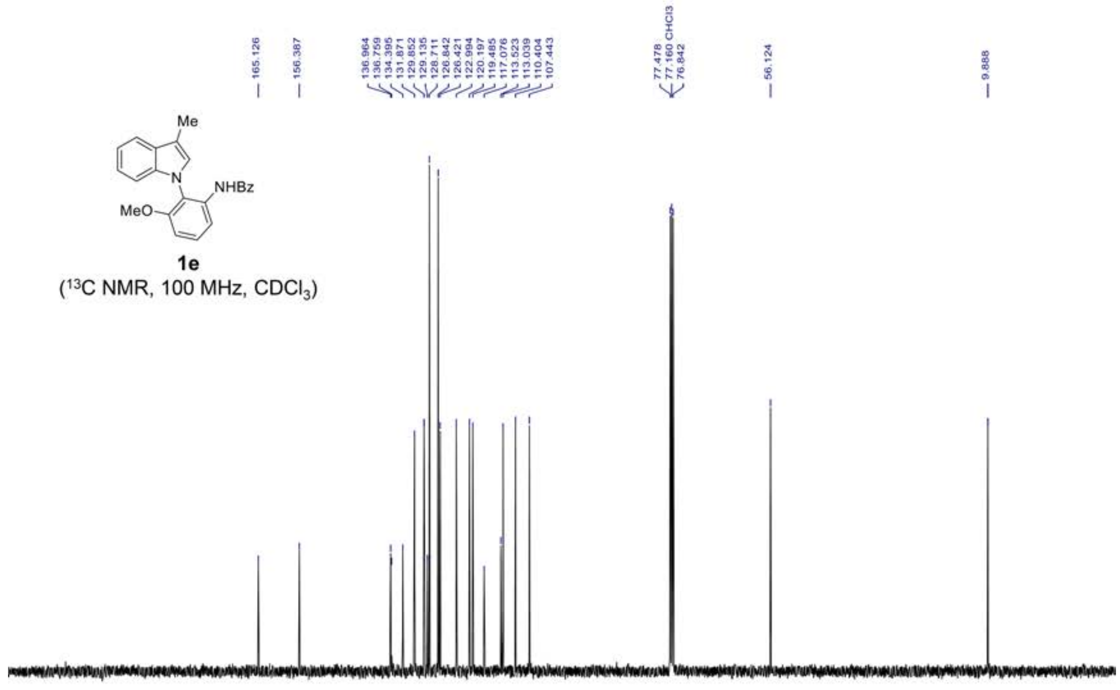

1e
(13C NMR, 100 MHz, $CDCl_3$)

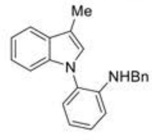

1f
(^{1}H NMR, 400 MHz, $CDCl_3$)

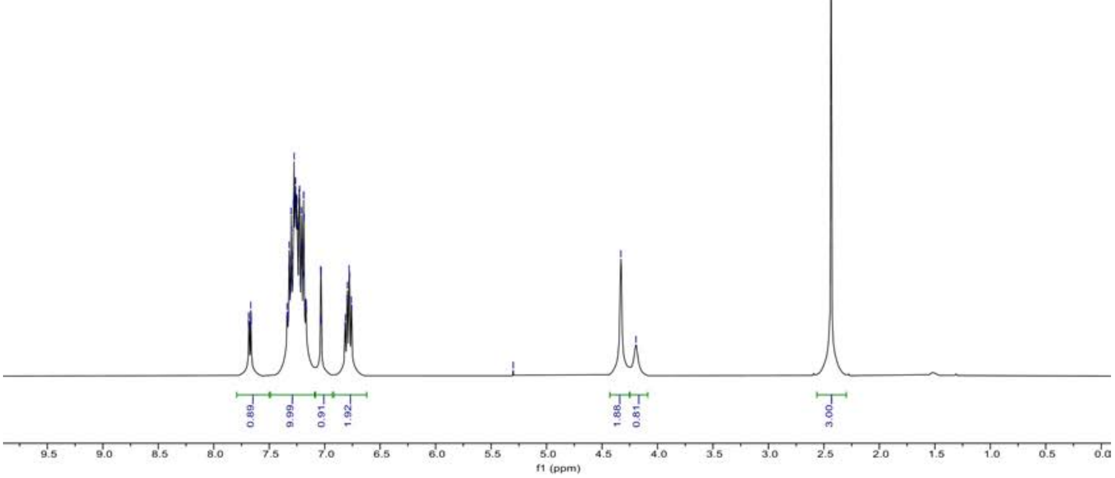

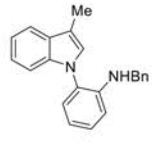

1f
(^{13}C NMR, 100 MHz, $CDCl_3$)

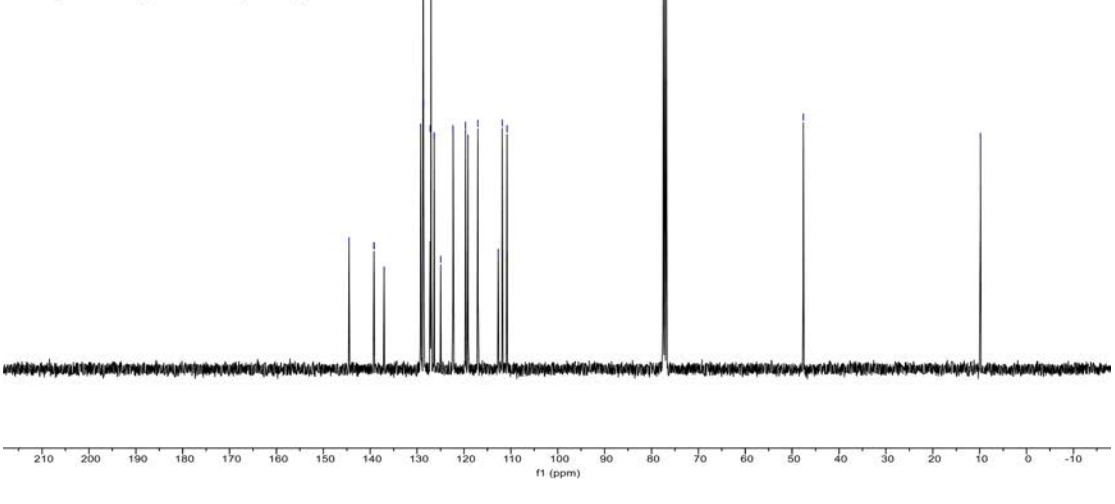

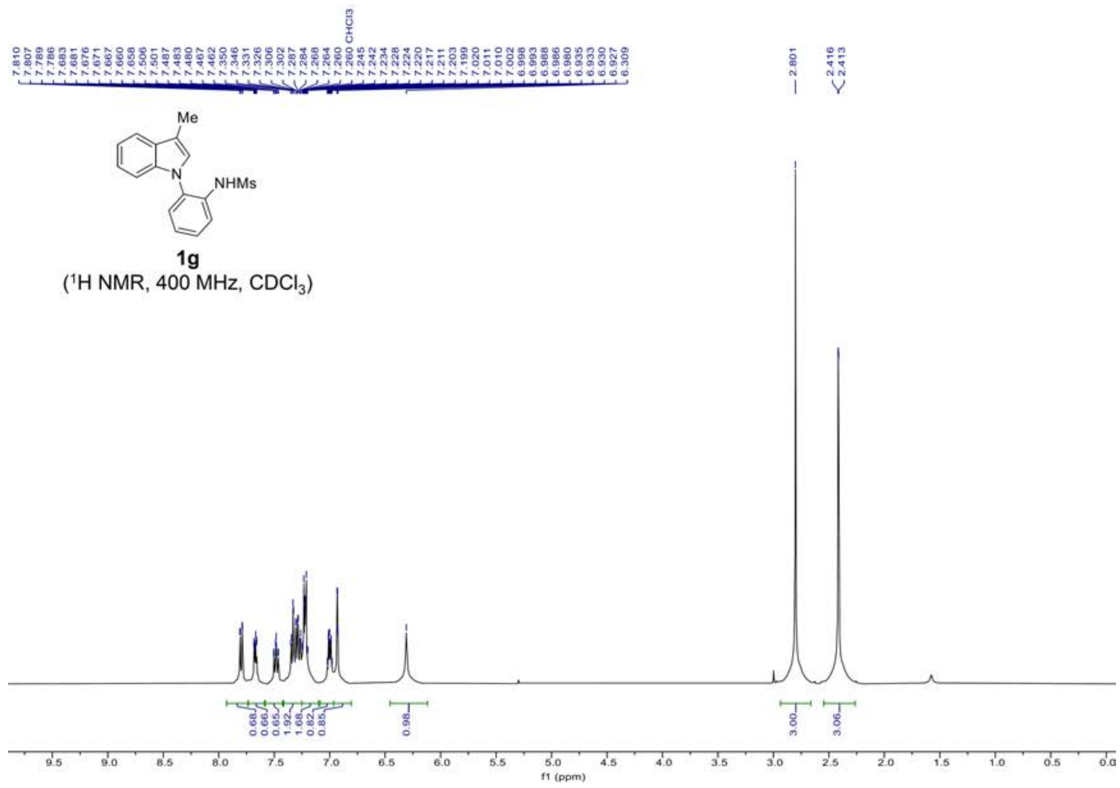

1g
(^{1}H NMR, 400 MHz, $CDCl_3$)

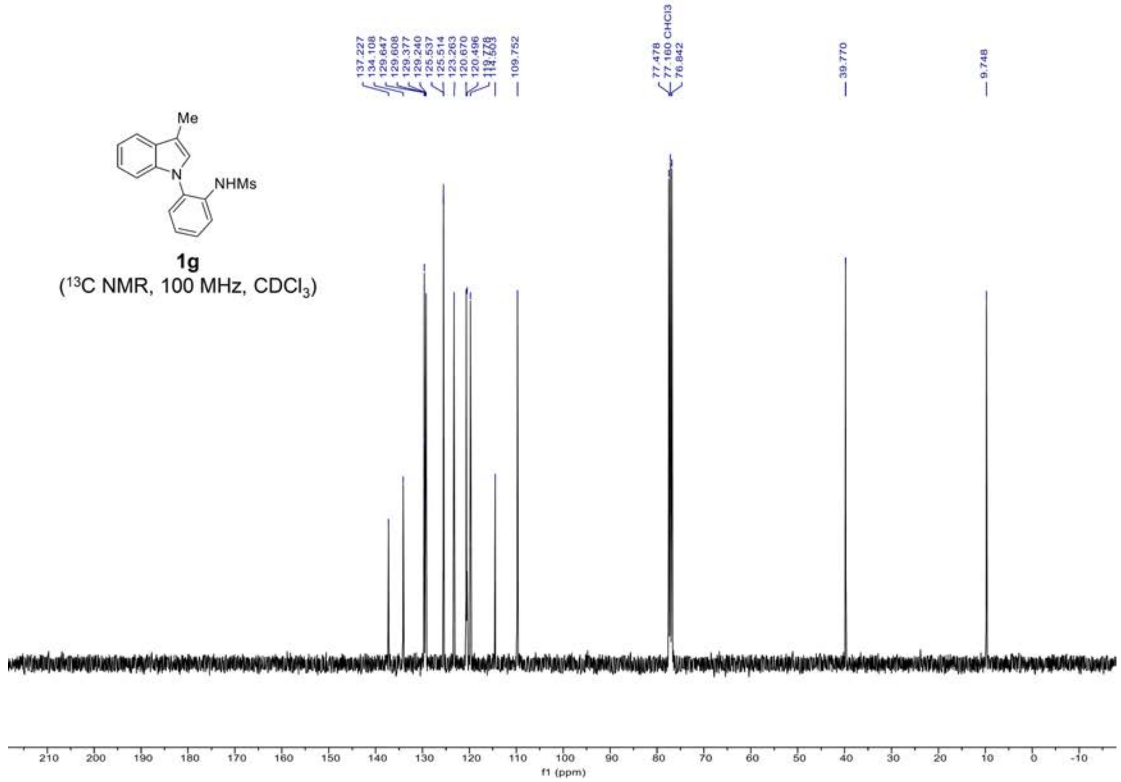

1g
(^{13}C NMR, 100 MHz, $CDCl_3$)

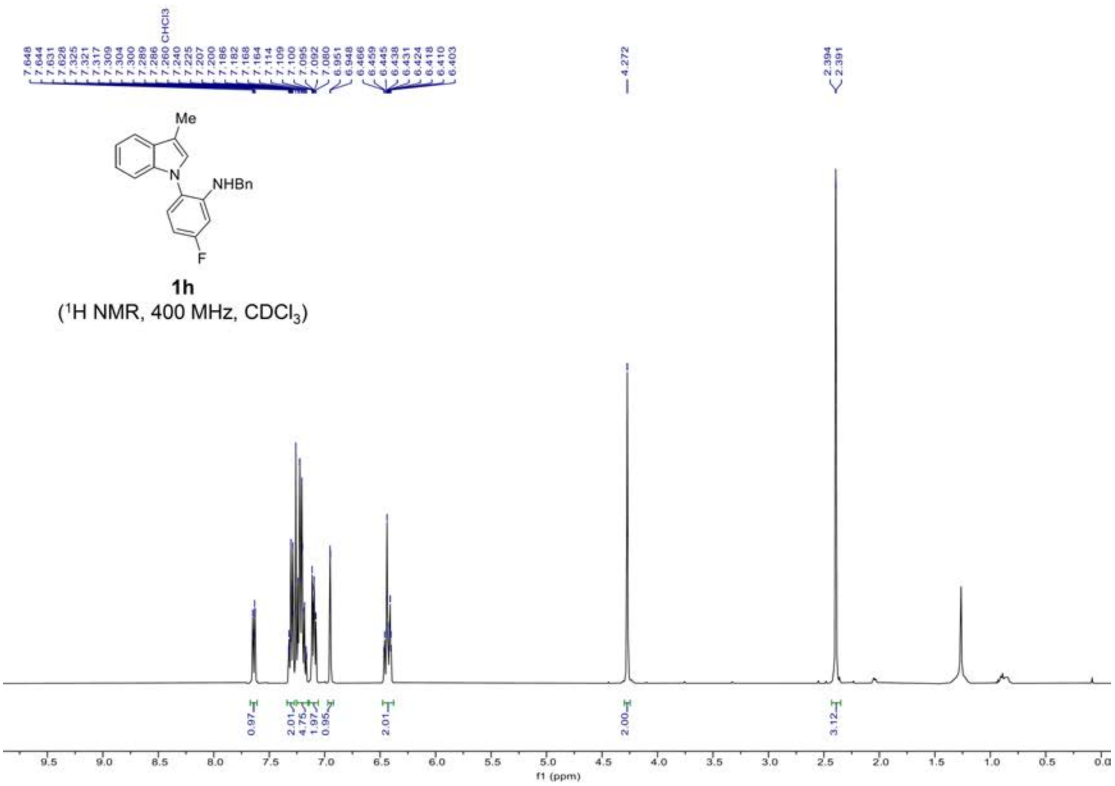
7.648 7.644 7.631 7.628 7.325 7.321 7.317 7.309 7.304 7.300 7.289 7.286 7.260 CHCl3 7.240 7.225 7.207 7.200 7.186 7.182 7.168 7.164 7.114 7.109 7.100 7.095 7.092 7.080 6.951 6.948 6.466 6.459 6.445 6.438 6.431 6.424 6.418 6.410 6.403
4.272
2.394 2.391
Me
N
NHBn
F
1h
(1H NMR, 400 MHz, CDCl3)
0.97
2.01
4.75
1.97
0.95
2.01
2.00
3.12
f1 (ppm)

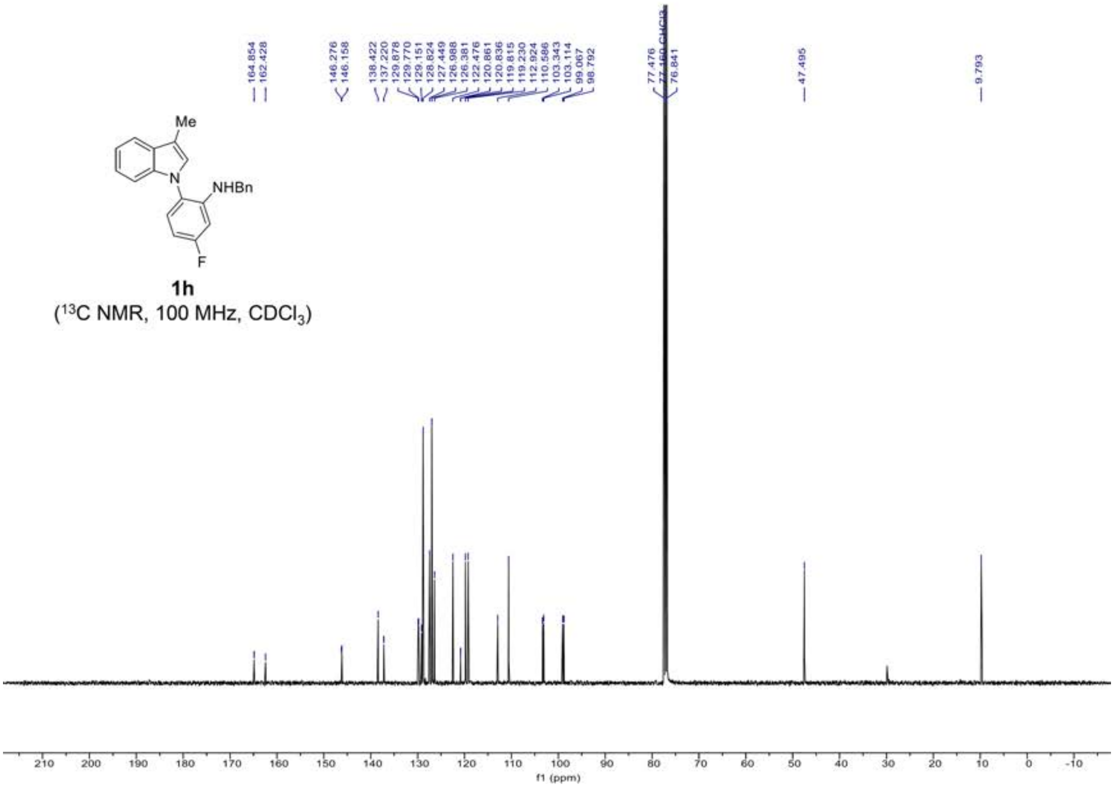
164.854 162.428
146.276 146.158
138.422 137.220 129.878 129.770 129.151 128.824 127.449 126.988 126.381 122.476 120.861 120.836 119.815 119.230 112.924 110.586 103.343 103.114 99.067 98.792
77.476 77.160 CHCl3 76.841
47.495
9.793
Me
N
NHBn
F
1h
(13C NMR, 100 MHz, CDCl3)
f1 (ppm)

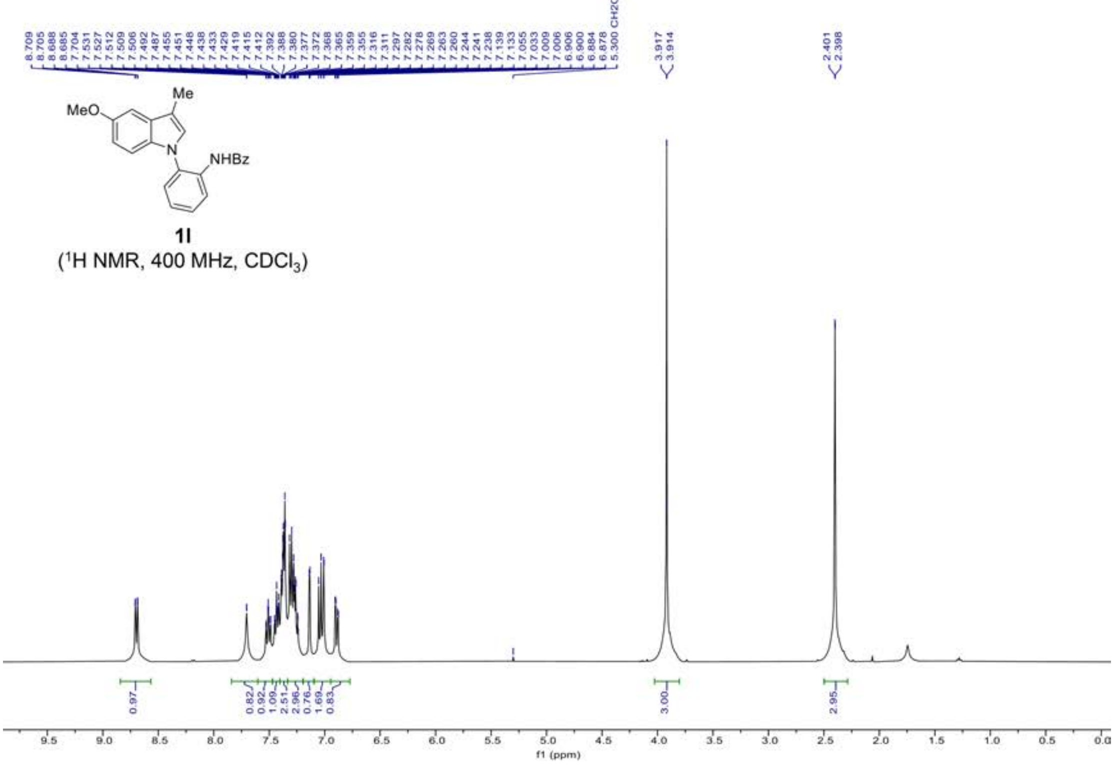

1l
(^{1}H NMR, 400 MHz, $CDCl_3$)

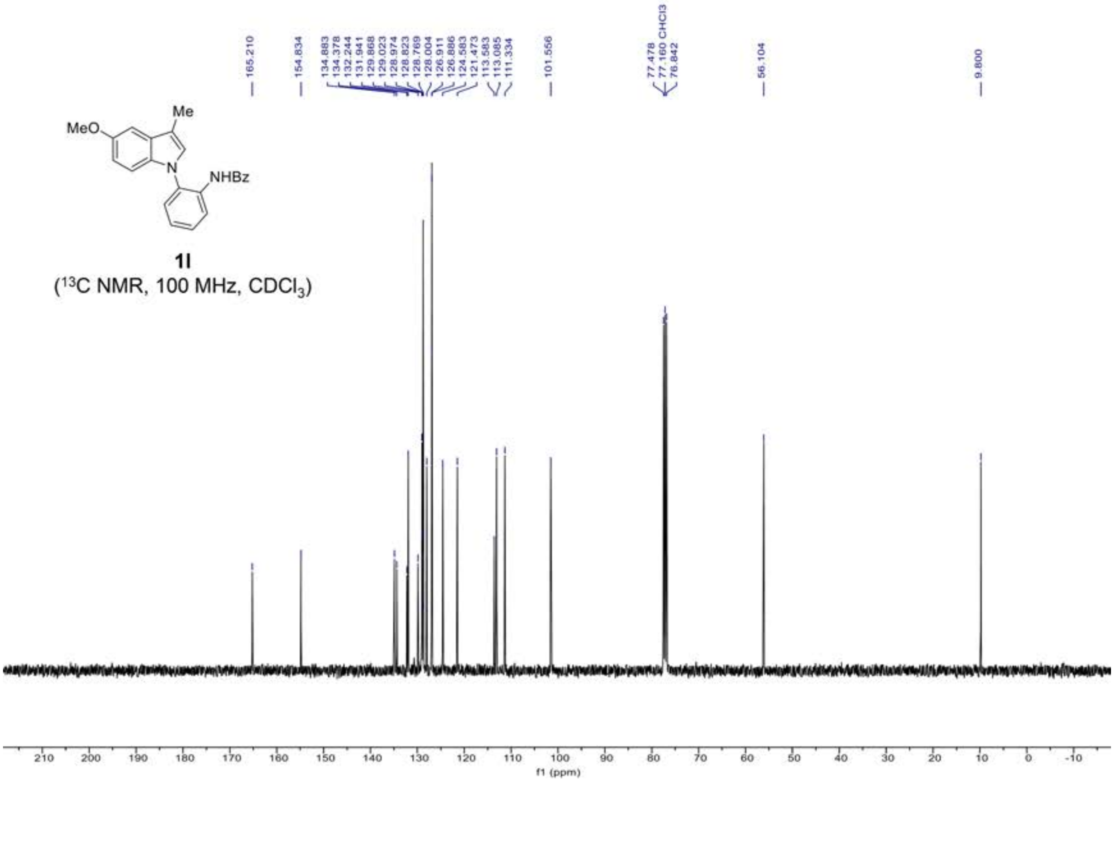

1l
(^{13}C NMR, 100 MHz, $CDCl_3$)

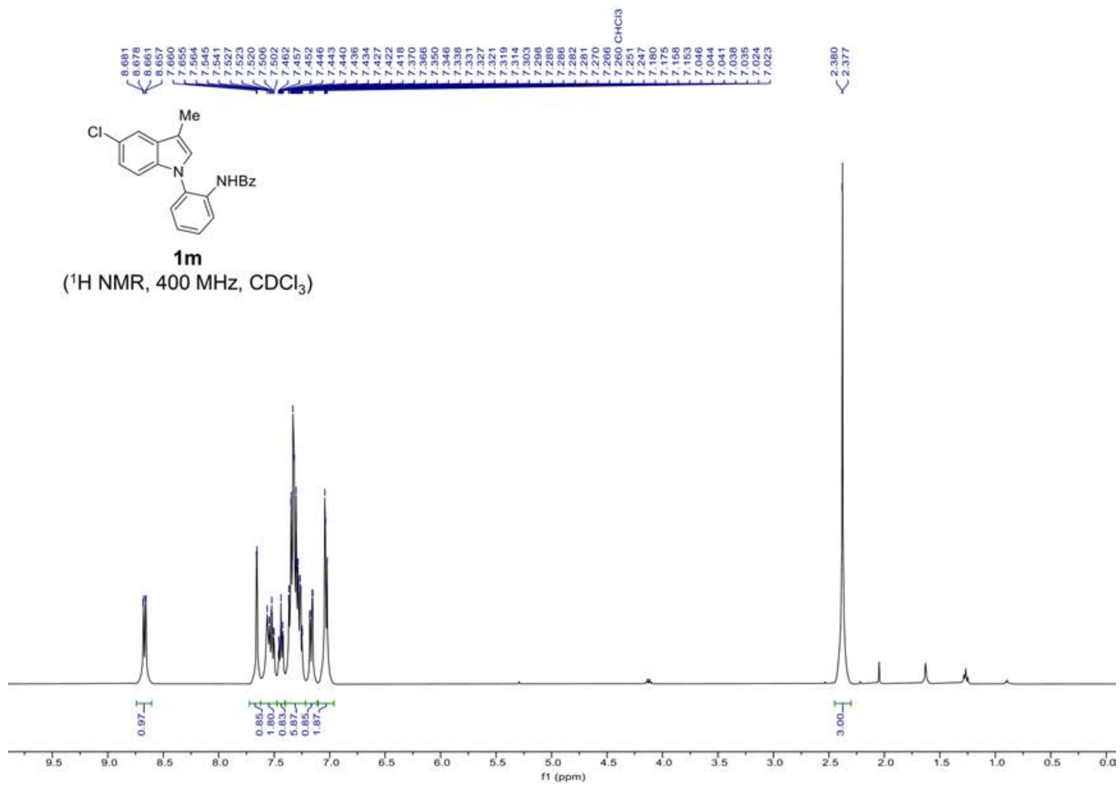

1m
(^{1}H NMR, 400 MHz, $CDCl_3$)

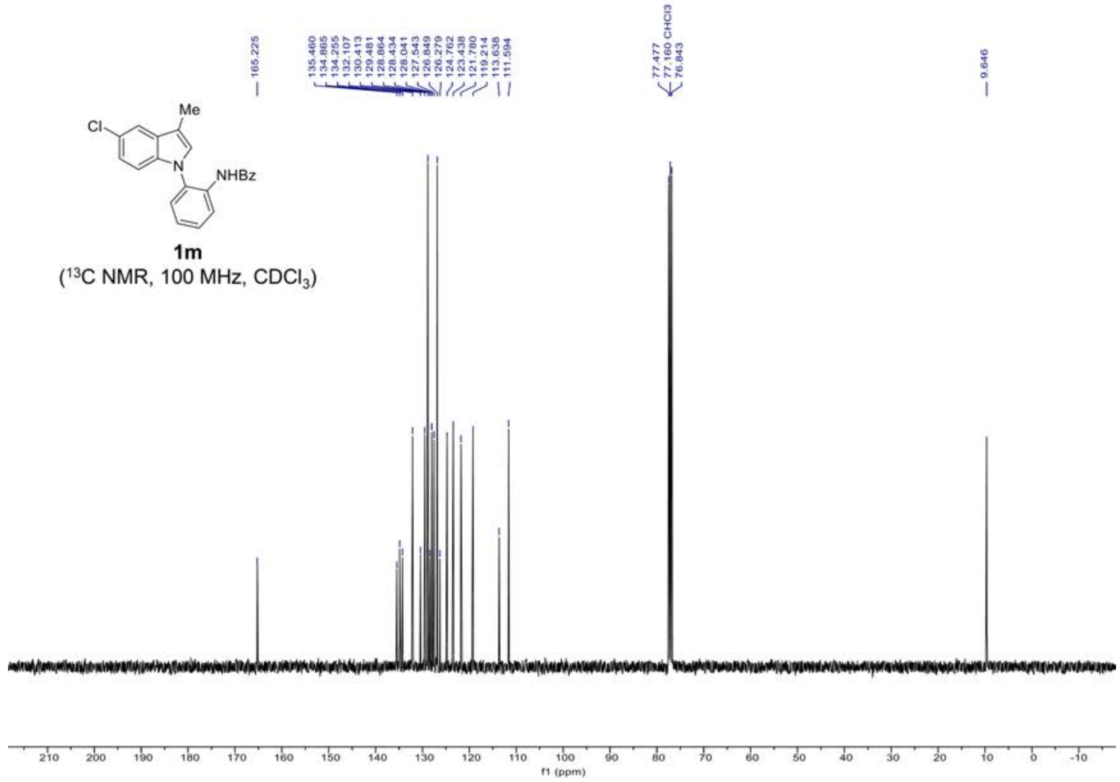

1m
(^{13}C NMR, 100 MHz, $CDCl_3$)

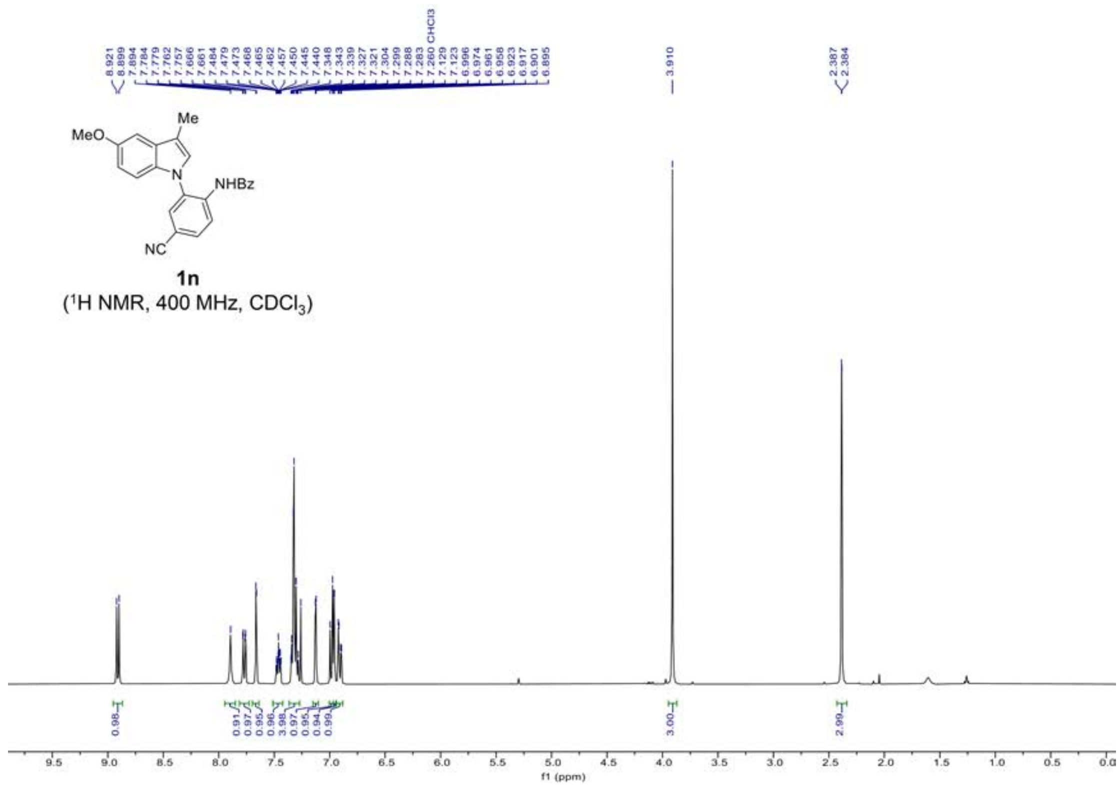

1n
(^{1}H NMR, 400 MHz, $CDCl_3$)

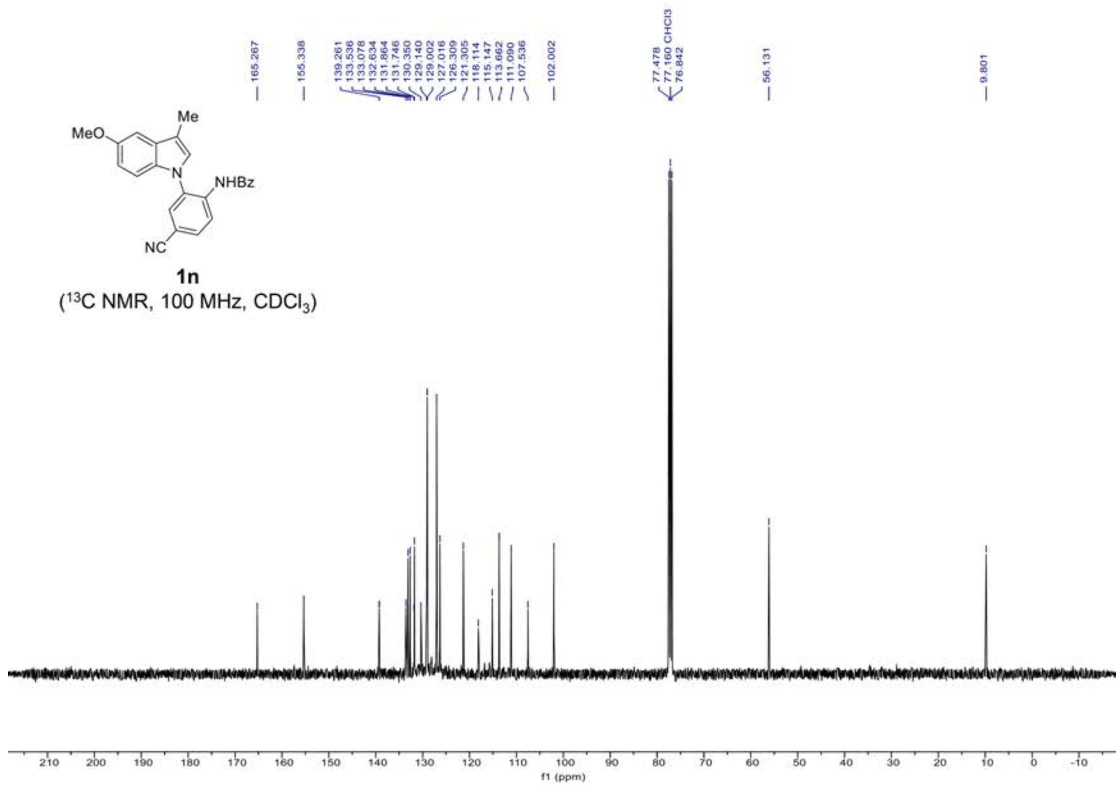

1n
(^{13}C NMR, 100 MHz, $CDCl_3$)

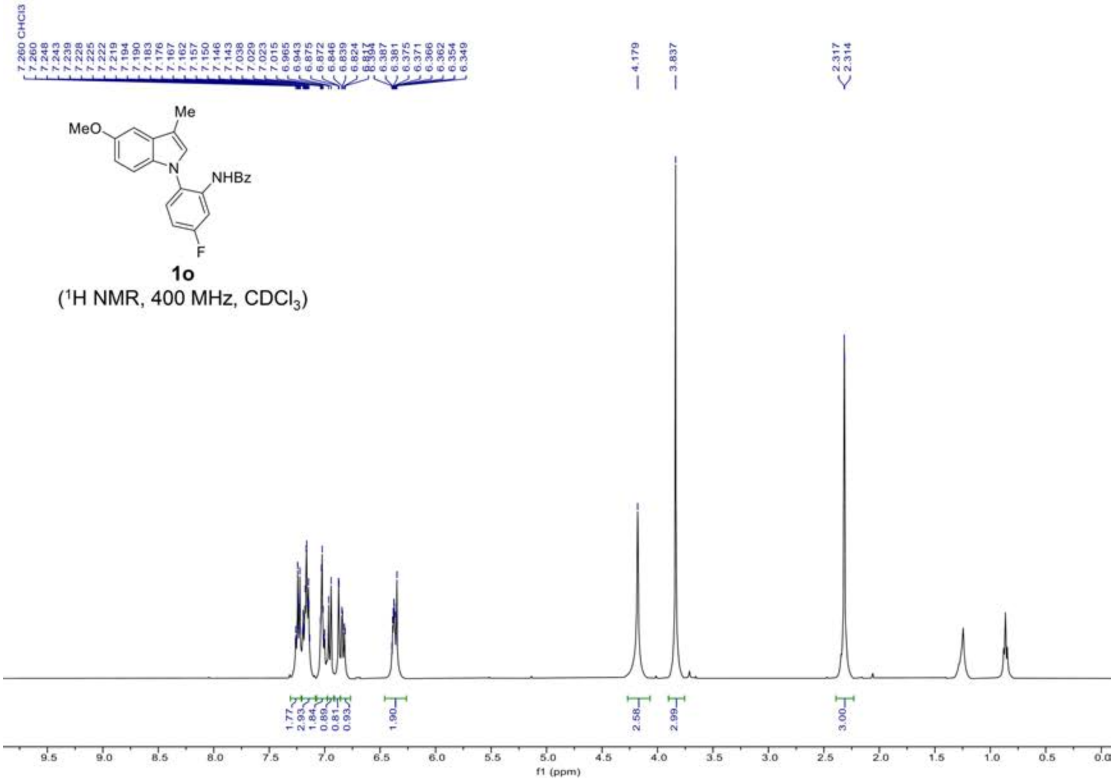
7.260 CHCl3
7.260
7.248
7.243
7.239
7.228
7.225
7.222
7.219
7.194
7.190
7.183
7.176
7.167
7.162
7.157
7.150
7.146
7.143
7.038
7.029
7.023
7.015
6.965
6.943
6.875
6.872
6.846
6.839
6.824
6.387
6.381
6.375
6.371
6.366
6.362
6.354
6.349
4.179
3.837
2.317
2.314
MeO
Me
NHBz
F
1o
(1H NMR, 400 MHz, CDCl3)
1.77
2.93
1.84
0.89
0.81
0.93
1.90
2.58
2.99
3.00
f1 (ppm)

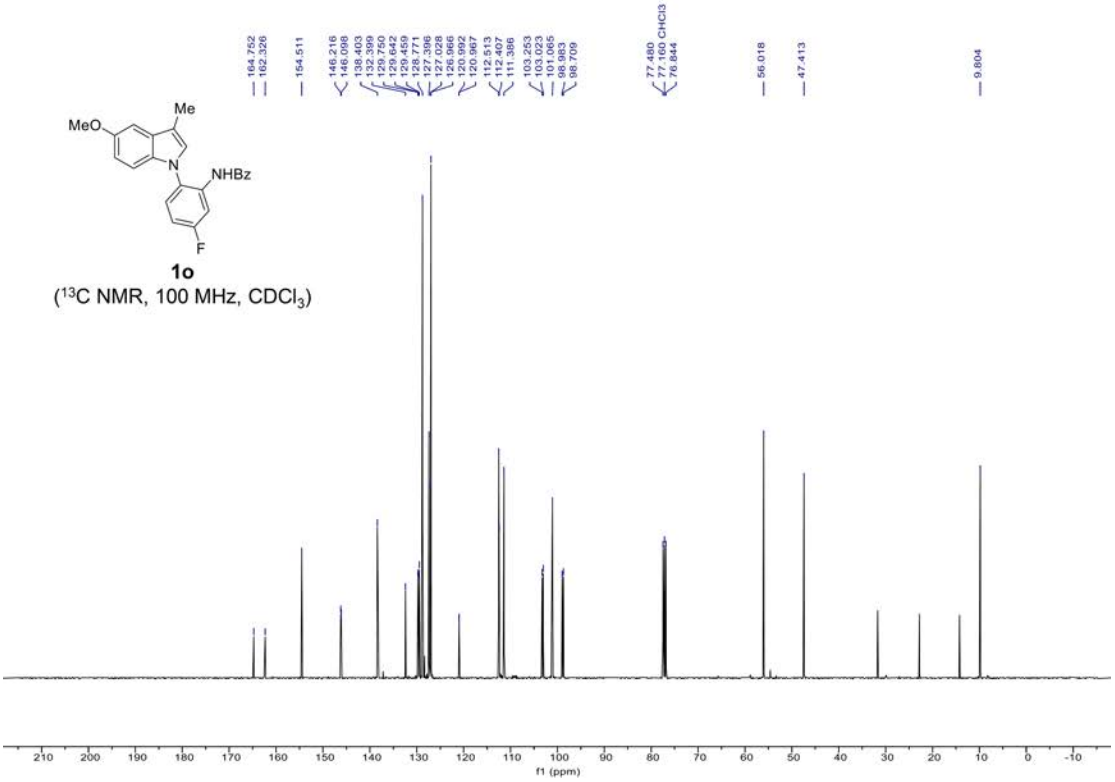
164.752
162.326
154.511
146.216
146.098
138.403
132.399
129.750
129.642
129.459
128.771
127.396
127.028
126.966
120.992
120.967
112.513
112.407
111.386
103.253
103.023
101.065
98.983
98.709
77.480
77.160 CHCl3
76.844
56.018
47.413
9.804
MeO
Me
NHBz
F
1o
(13C NMR, 100 MHz, CDCl3)
f1 (ppm)

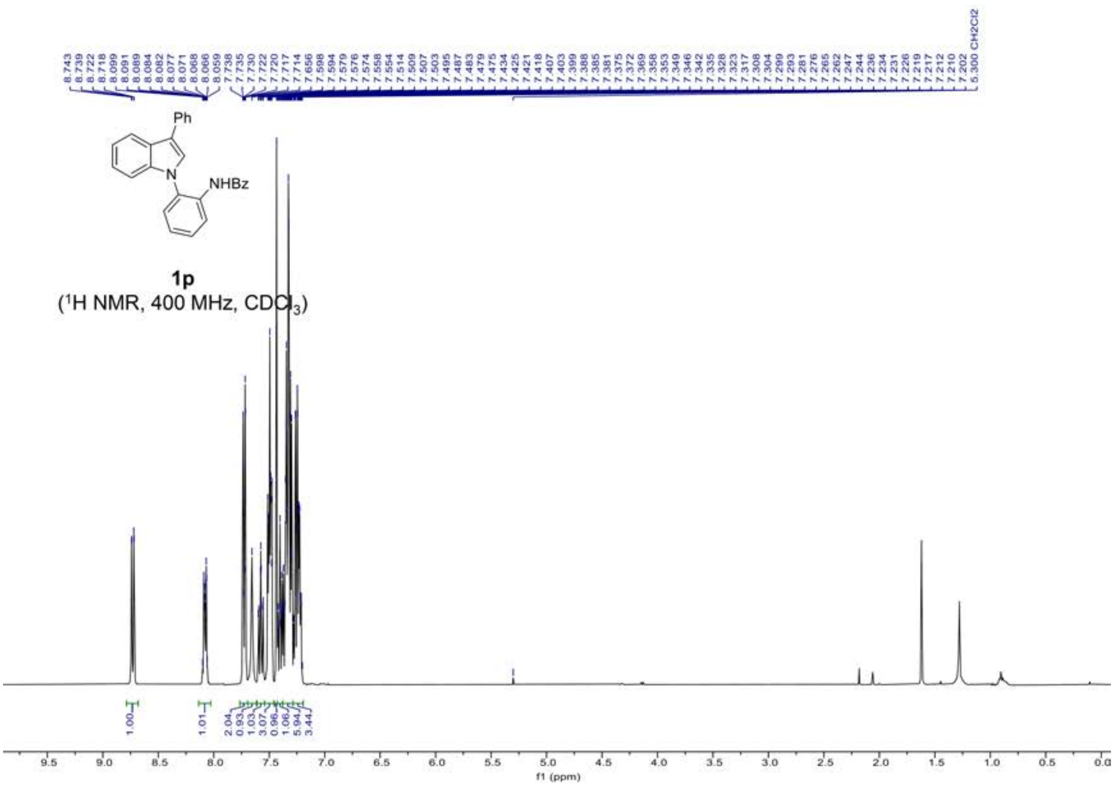
8.743
8.739
8.722
8.718
8.099
8.091
8.089
8.084
8.082
8.077
8.071
8.068
8.066
8.059
7.738
7.735
7.730
7.722
7.720
7.717
7.714
7.598
7.594
7.579
7.576
7.574
7.558
7.554
7.514
7.509
7.507
7.503
7.495
7.487
7.483
7.479
7.475
7.434
7.425
7.421
7.418
7.407
7.403
7.399
7.388
7.385
7.381
7.375
7.372
7.369
7.358
7.353
7.349
7.346
7.342
7.335
7.328
7.323
7.317
7.308
7.304
7.299
7.293
7.281
7.276
7.265
7.262
7.247
7.244
7.236
7.234
7.231
7.226
7.219
7.217
7.212
7.210
7.202
5.300 CH2Cl2
Ph
N
NHBz
1p
(1H NMR, 400 MHz, CDCl3)
1.00
1.01
2.04
0.93
1.03
3.07
0.96
1.06
5.94
3.44
9.5
9.0
8.5
8.0
7.5
7.0
6.5
6.0
5.5
5.0
4.5
4.0
3.5
3.0
2.5
2.0
1.5
1.0
0.5
0.0
f1 (ppm)

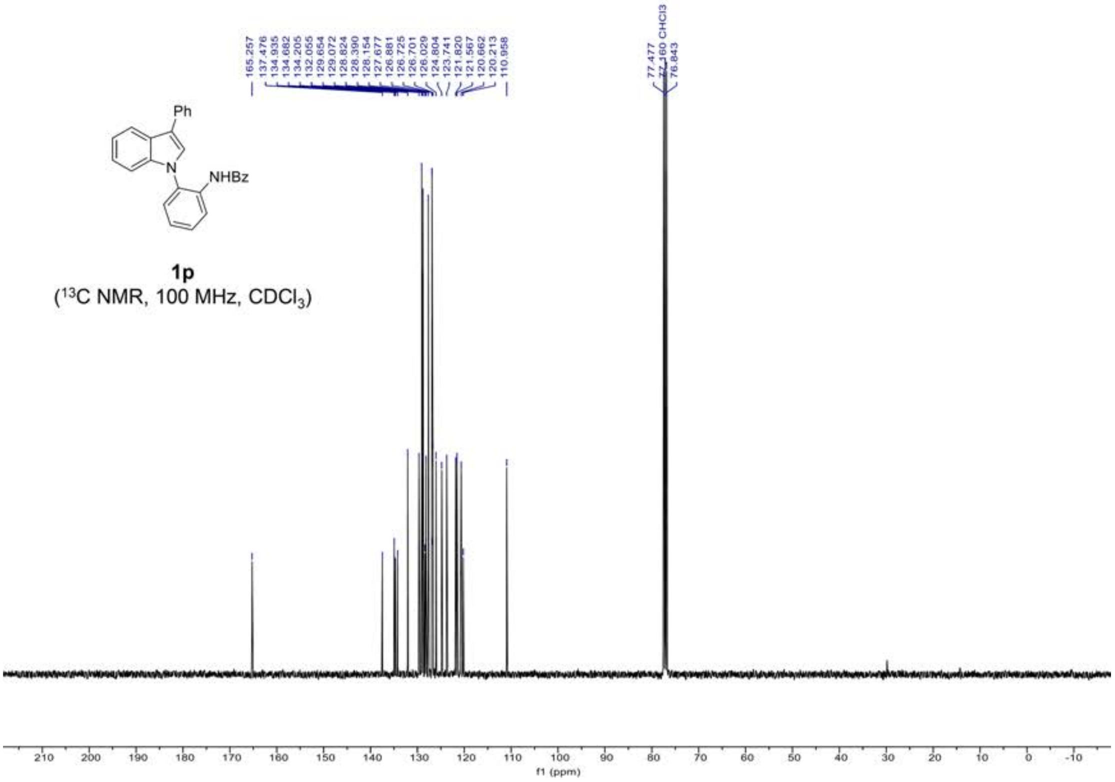
165.257
137.476
134.935
134.682
134.205
132.065
129.654
129.072
128.824
128.390
128.154
127.677
126.881
126.725
126.701
126.029
124.804
123.741
121.820
121.567
120.662
120.213
110.958
77.477
77.160 CHCl3
76.843
Ph
N
NHBz
1p
(13C NMR, 100 MHz, CDCl3)
210
200
190
180
170
160
150
140
130
120
110
100
90
80
70
60
50
40
30
20
10
0
-10
f1 (ppm)

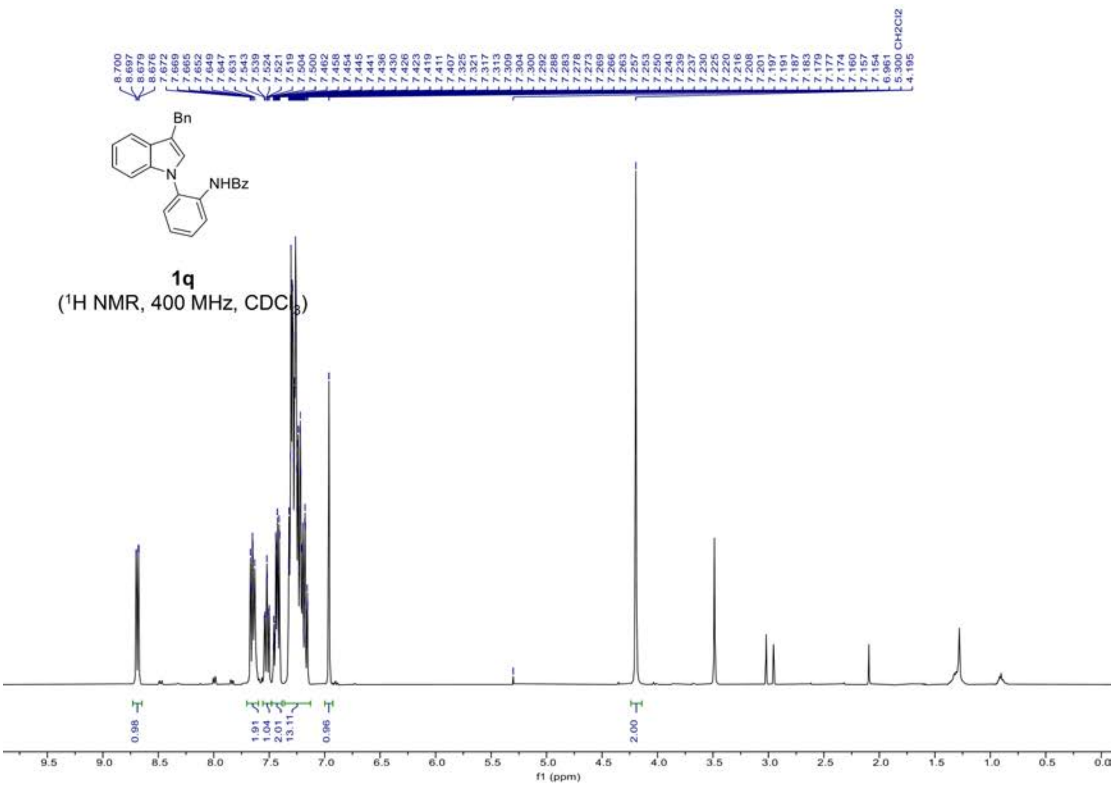
8.700 8.697 8.679 8.676 7.672 7.669 7.665 7.652 7.649 7.647 7.631 7.543 7.539 7.524 7.521 7.519 7.504 7.500 7.462 7.458 7.454 7.445 7.441 7.436 7.430 7.426 7.423 7.419 7.411 7.407 7.325 7.321 7.317 7.313 7.309 7.304 7.300 7.292 7.288 7.283 7.278 7.273 7.269 7.266 7.263 7.257 7.253 7.250 7.243 7.239 7.237 7.230 7.225 7.220 7.216 7.208 7.201 7.197 7.191 7.187 7.183 7.179 7.177 7.174 7.160 7.157 7.154 6.961 5.300 CH2Cl2 4.195
Bn
NHBz
1q
(1H NMR, 400 MHz, CDCl3)
0.98
1.91
1.04
2.01
13.11
0.96
2.00
f1 (ppm)

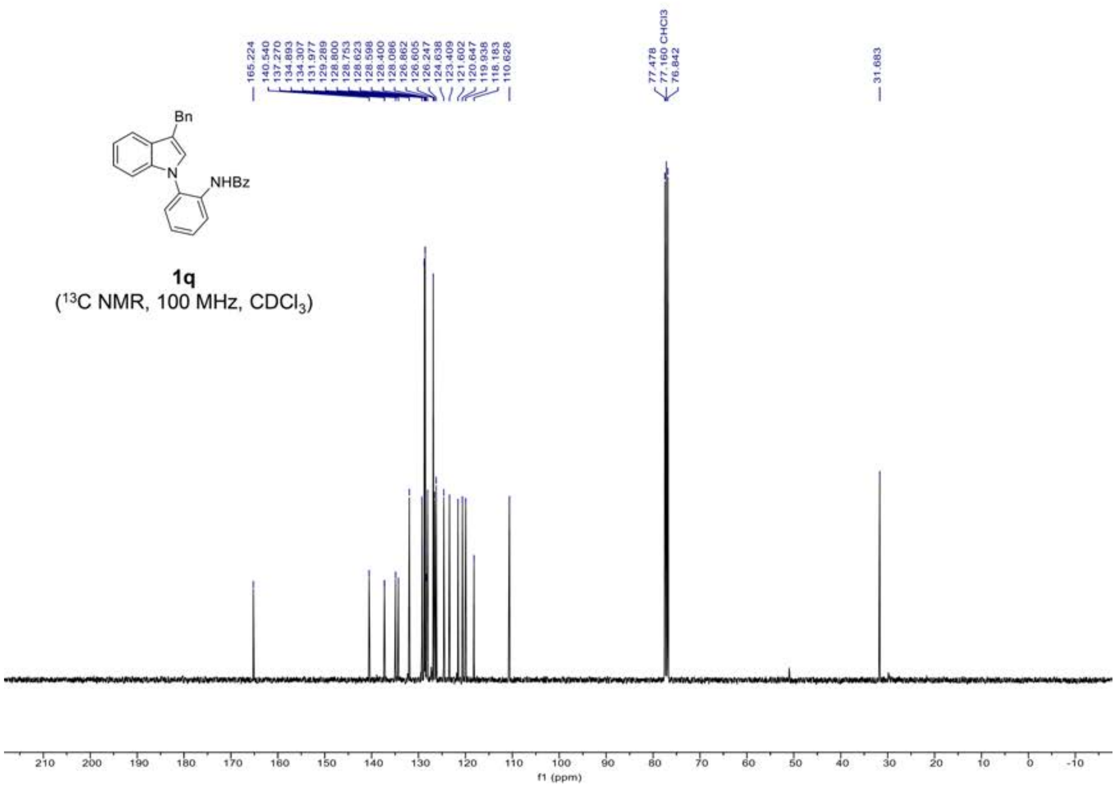
165.224 140.540 137.270 134.893 134.307 131.977 129.289 128.800 128.753 128.623 128.598 128.400 126.866 126.862 126.605 126.247 124.638 123.409 121.602 120.647 119.938 118.183 110.628
77.478 77.160 CHCl3 76.842
31.683
Bn
NHBz
1q
(13C NMR, 100 MHz, CDCl3)
f1 (ppm)

3 Synthesis of 4

3.1 Synthesis of Substituted 2-Nitroarylindoles (S13−S19)

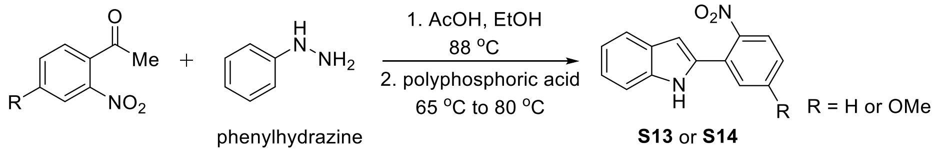

S13 or **S14**

Procedure 6: To a reaction tube equipped with a magnetic stirring bar, a solution of 2-nitroacetophenone or 4-methoxy-2-nitroacetophenone (1.0 equiv) in EtOH (2.5 M) was added. Then, phenylhydrazine (1.4 equiv) and glacial acetic acid (0.35 equiv) were added. After completion of addition, the reaction mixture was heated to 88 ºC in oil bath for 6 h. The progress of the reaction was monitored by TLC. The reaction mixture was cooled to rt, stored in the refrigerator overnight. The formed crystals were filtered with diethyl ether, washed with ethanol, and concentrated *in vacuo.* Corresponding phenylhydrazone was directly employed in the next step without further purification.
To a reaction tube equipped with a magnetic stirring bar, the above phenylhydrazone was pulverized and mixed with polyphosphoric acid (PPA, 0.83 M). The mixture was heated to 65 ºC, then, the temperature was gradually raised to 80 ºC and the mixture was held at this temperature for 30 min. The reaction mixture was cooled to rt and poured into ice-water. The aqueous phase was extracted with Et_2O three times. The combined organic phase was washed with water, dried with anhydrous Na_2SO_4, and concentrated *in vacuo*. The crude material was recrystallized from EtOH to afford the desired material **S13** or **S14**.

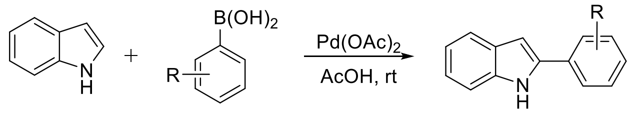

S15–S18

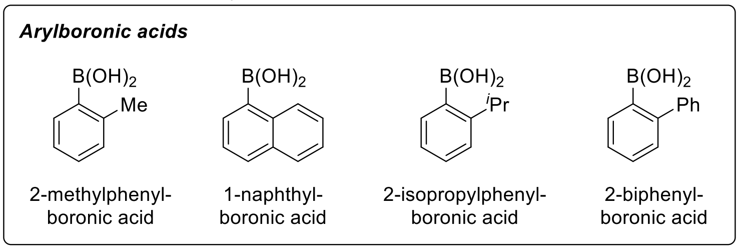

Procedure 7: To a reaction tube equipped with a magnetic stirring bar, indole (1.0 equiv), substituted phenylboronic acids (1.5 equiv), palladium acetate (0.05 equiv) and subsequently dissolved in AcOH (0.1 M). The reaction was stirred for 2–4 days at rt. The progress of the reaction was monitored by TLC. AcOH was removed under reduced pressure, the residue was dissolved in CH_2Cl_2 and quenched with aqueous $NaHCO_3$. The combined organic layers were dried with anhydrous $MgSO_4$ and concentrated *in vacuo*. The crude material was then purified by flash chromatography to afford the desired material **S15**–**S18**.

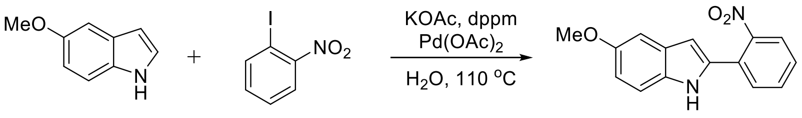

5-methoxyindole 1-Iodo-2-nitrobenzene **S19**

Procedure 8: To a sealed round-bottom flask equipped with a magnetic stirring bar, 5-methoxyindole (1.0 equiv), 1-iodo-2-nitrobenzene (1.2 equiv), potassium acetate (3.0 equiv), bis-(diphenylphosphino)methane (dppm, 0.06 equiv), and palladium acetate (0.06 equiv) were added and subsequently dissolved in water (0.33 M). The reaction was heated to 110 °C and allowed to stir for two-days. The progress of the reaction was monitored by TLC. After cooled to rt, the aqueous phase was extracted with three times. The combined organic layers were dried with anhydrous $MgSO_4$ and concentrated *in vacuo*. The crude material was then purified by flash chromatography to afford the desired material **S19**.

Characterization and Spectra of Substituted 2-Nitroarylindoles (S13–S19)

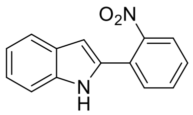

2-(2-Nitrophenyl)-1*H*-indole (S13) was synthesized by following Procedure 6. The crude material was purified by normal-phase column chromatography using an eluent of 20% EtOAc/Hx to provide **S13** (3.71 g, 62%).

^{1}H NMR (400 MHz, $CDCl_3$) δ 8.51 (brs, 1H), 7.81 (dd, J = 8.1, 1.3 Hz, 1H), 7.73–7.59 (m, 3H), 7.50 (ddd, J = 8.1, 7.4, 1.5 Hz, 1H), 7.41 (dq, J = 8.2, 1.0 Hz, 1H), 7.28–7.21 (m, 1H), 7.15 (ddd, J = 8.1, 7.0, 1.1 Hz, 1H), 6.72 (dd, J = 2.2, 1.0 Hz, 1H).
^{13}C NMR (100 MHz, $CDCl_3$) δ 149.0, 137.1, 132.5 (2C), 131.8, 128.8, 128.4, 127.1, 124.4, 123.4, 121.1, 120.6, 111.4, 104.6.

The spectral data were identical with those previously reported.[6]

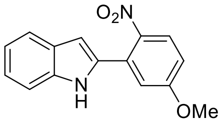

2-(5-Methoxy-2-nitrophenyl)-1*H*-indole (S14) was synthesized by following Procedure 6. The crude material was purified by normal-phase column chromatography using an eluent of 25% EtOAc/Hx to provide **S14** (820 mg, 60%).

^{1}H NMR (400 MHz, $CDCl_3$) δ 7.99 (d, J = 9.0 Hz, 1H), 7.65 (dd, J = 7.9, 1.0 Hz, 1H), 7.41 (dd, J = 8.2, 1.0 Hz, 1H), 7.27–7.22 (m, 1H), 7.17–7.11 (m, 2H), 6.96 (dd, J = 9.1, 2.8 Hz, 1H), 6.71 (dd, J = 2.2, 1.0 Hz, 1H), 3.93 (s, 3H).
^{13}C NMR (100 MHz, $CDCl_3$) δ 162.7, 141.6, 136.9, 133.3, 130.2, 128.2, 127.4, 123.1, 120.9, 120.4, 117.2, 113.8, 111.4, 104.2, 56.0.
IR (FT-ATR, cm^{-1}, $CHCl_3$) ν_{max} 3946, 3926, 3899, 3838, 3799, 3780, 3730, 3703, 3626, 3599, 3545, 3398, 3190, 3059, 3016, 2974, 2939, 2843, 2642, 2507, 2360, 2333, 2295, 2268, 2052, 1917, 1894, 1774, 1743, 1705, 1674, 1608, 1581, 1516, 1454, 1338, 1300, 1277, 1238, 1169, 1103, 1049, 1026, 957, 872, 829, 798, 752, 679.
HRMS (EI) m/z: $[M]^+$ Calcd for $C_{15}H_{12}N_2O_3$ 268.0848; found 268.0845.

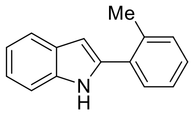

2-(*o*-Tolyl)-1*H*-indole (S15) was synthesized by following Procedure 7. The crude material was purified by normal-phase column chromatography using an eluent of 13% EtOAc/Hx to provide **S15** (1.26 g, 48%).

^{1}H NMR (400 MHz, $CDCl_3$) δ 8.10 (s, 1H), 7.70 (ddd, J = 7.8, 1.4, 0.8 Hz, 1H), 7.52–7.46 (m, 1H), 7.42 (dq, J = 8.0, 1.0 Hz, 1H), 7.34 (tdd, J = 8.7, 4.3, 2.8 Hz, 3H), 7.25 (ddd, J = 8.2, 7.0, 1.3 Hz, 1H), 7.19 (ddd, J = 8.1, 7.1, 1.1 Hz, 1H), 6.65 (dd, J = 2.2, 1.0 Hz, 1H), 2.53 (s, 3H).

^{13}C NMR (100 MHz, $CDCl_3$) δ 137.5, 136.3, 136.2, 132.7, 131.2, 129.1, 128.9, 128.1, 126.2, 122.2, 120.6, 120.2, 110.9, 103.1, 21.2.

The spectral data were identical with those previously reported.[7]

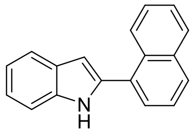

2-(Naphthalen-1-yl)-1*H*-indole (S16) was synthesized by following Procedure 7. The crude material was purified by normal-phase column chromatography using an eluent of 13% EtOAc/Hx to provide **S16** (275 mg, 22%).

^{1}H NMR (400 MHz, $CDCl_3$) δ 8.36–8.29 (m, 1H), 8.22 (s, 1H), 7.99–7.84 (m, 2H), 7.78–7.68 (m, 1H), 7.61 (dd, *J* = 7.1, 1.3 Hz, 1H), 7.53 (dqd, *J* = 8.5, 6.7, 1.6 Hz, 3H), 7.42 (dq, *J* = 8.0, 1.0 Hz, 1H), 7.31–7.16 (m, 2H), 6.81 (dd, *J* = 2.1, 1.0 Hz, 1H).
^{13}C NMR (100 MHz, $CDCl_3$) δ 136.8, 136.4, 134.0, 131.6, 131.2, 129.0, 128.7, 128.6, 127.3, 126.8, 126.3, 125.8, 125.5, 122.3, 120.7, 120.3, 111.0, 103.8.

The spectral data were identical with those previously reported.[8]

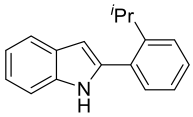

2-(2-Isopropylphenyl)-1*H*-indole (S17) was synthesized by following Procedure 7. The crude material was purified by normal-phase column chromatography using an eluent of 9% EtOAc/Hx to provide **S17** (873 mg, 43%).

^{1}H NMR (400 MHz, $CDCl_3$) δ 7.89 (s, 1H), 7.57 (dd, *J* = 7.7, 1.3 Hz, 1H), 7.41–7.21 (m, 4H), 7.21–7.00 (m, 3H), 6.46 (dd, *J* = 2.1, 0.9 Hz, 1H), 3.31 (hept, *J* = 6.8 Hz, 1H), 1.15 (d, *J* = 7.0 Hz, 6H).
^{13}C NMR (100 MHz, $CDCl_3$) δ 147.9, 137.5, 136.1, 132.0, 130.2, 128.9, 128.8, 126.1, 125.7, 122.0, 120.5, 120.2, 110.9, 102.9, 29.6, 24.6 (2C).
IR (FT-ATR, cm^{-1}, $CHCl_3$) ν_{max} 3934, 3907, 3884, 3842, 3822, 3807, 3784, 3757, 3718, 3680, 3656, 3633, 3595, 3568, 3406, 3224, 3194, 3055, 2962, 2927, 2870, 2723, 2634, 2600, 2546, 2507, 2372, 2349, 2314, 2249, 2198, 2160, 2094, 2067, 1921, 1882, 1844, 1805, 1774, 1693, 1651, 1612, 1577, 1550, 1512, 1481, 1450, 1411, 1342, 1296, 1231, 1149, 1088, 1030, 1011, 957, 926, 872, 845, 795, 744, 679.
HRMS (EI) *m/z*: $[M]^+$ Calcd for $C_{17}H_{17}N$ 235.1361; found 235.1359.

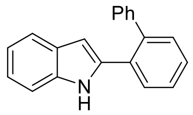

2-([1,1'-Biphenyl]-2-yl)-1*H*-indole (S18) was synthesized by following Procedure 7. The crude material was purified by normal-phase column chromatography using an eluent of 9% EtOAc/Hx to provide **S18** (205 mg, 18%).

^{1}**H NMR** (400 MHz, $CDCl_3$) δ 7.74–7.69 (m, 1H), 7.60–7.56 (m, 1H), 7.51 (s, 1H), 7.45–7.39 (m, 3H), 7.36–7.28 (m, 5H), 7.13– 7.03 (m, 3H), 6.58 (d, *J* = 2.3 Hz, 1H).
^{13}**C NMR** (100 MHz, $CDCl_3$) δ 141.2, 139.7, 137.8, 136.4, 131.2, 131.1, 130.0, 129.4, 128.6, 128.4, 128.0, 127.9, 127.5, 122.0, 120.5, 119.9, 110.8, 102.5.

The spectral data were identical with those previously reported.[9]

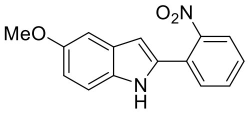

5-Methoxy-2-(2-nitrophenyl)-1*H*-indole (S19) was synthesized by following Procedure 8. The crude material was purified by normal-phase column chromatography using an eluent of 25% EtOAc/Hx to provide **S19** (1.50 g, 56%).

^{1}**H NMR** (400 MHz, $CDCl_3$) δ 8.50 (s, 1H), 7.76 (d, *J* = 8.1 Hz, 1H), 7.67–7.49 (m, 2H), 7.47–7.37 (m, 1H), 7.23 (dd, *J* = 9.0, 2.4 Hz, 1H), 7.10 (d, *J* = 2.4 Hz, 1H), 6.91 (dd, *J* = 8.9, 2.6 Hz, 1H), 6.64 (d, *J* = 2.2 Hz, 1H), 3.87 (s, 3H).
^{13}**C NMR** (100 MHz, $CDCl_3$) δ 154.5, 148.7, 133.0, 132.4, 132.3, 131.3, 128.8, 128.5, 126.8, 124.1, 113.8, 112.2, 103.9, 102.2, 55.8.

The spectral data were identical with those previously reported.[10]

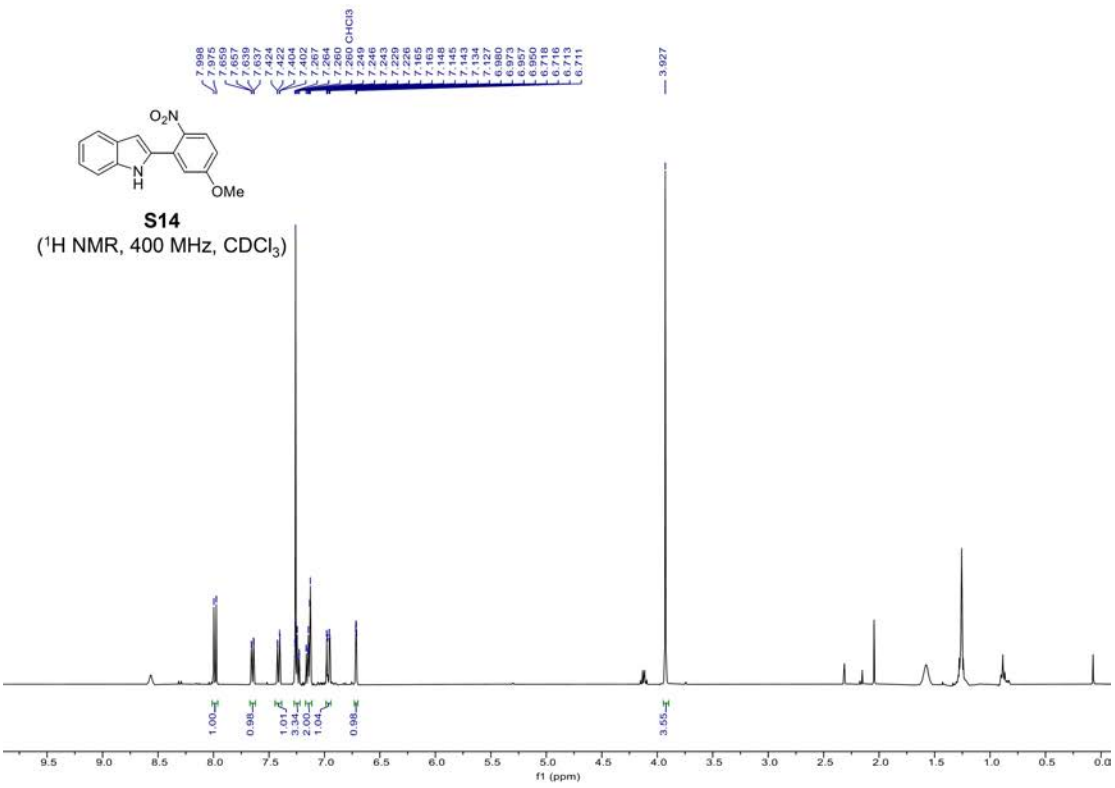

S14
(^{1}H NMR, 400 MHz, $CDCl_3$)

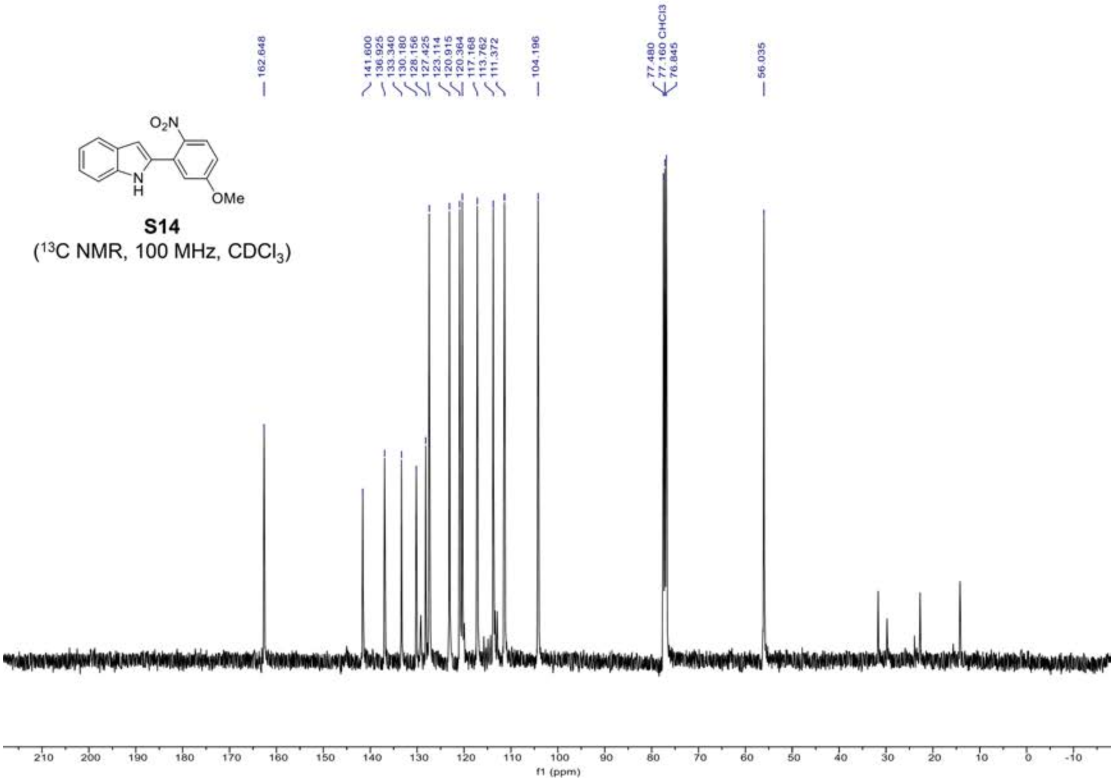

S14
(^{13}C NMR, 100 MHz, $CDCl_3$)

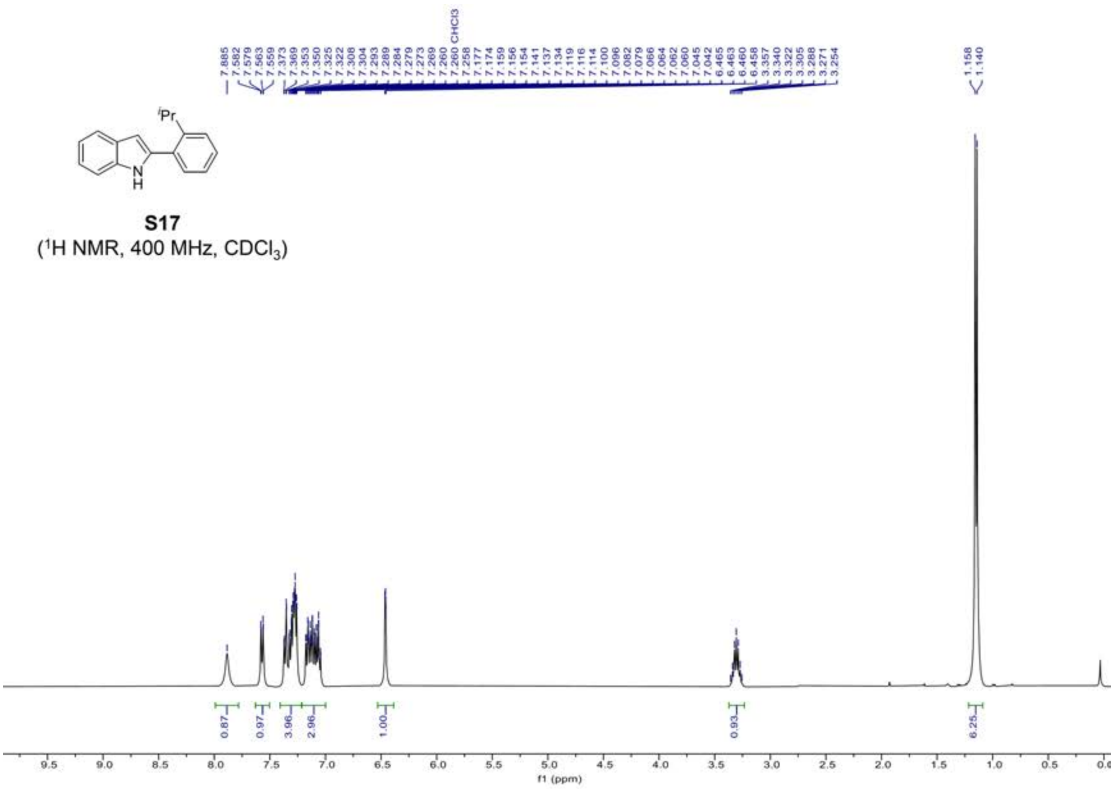

S17
(^{1}H NMR, 400 MHz, $CDCl_3$)

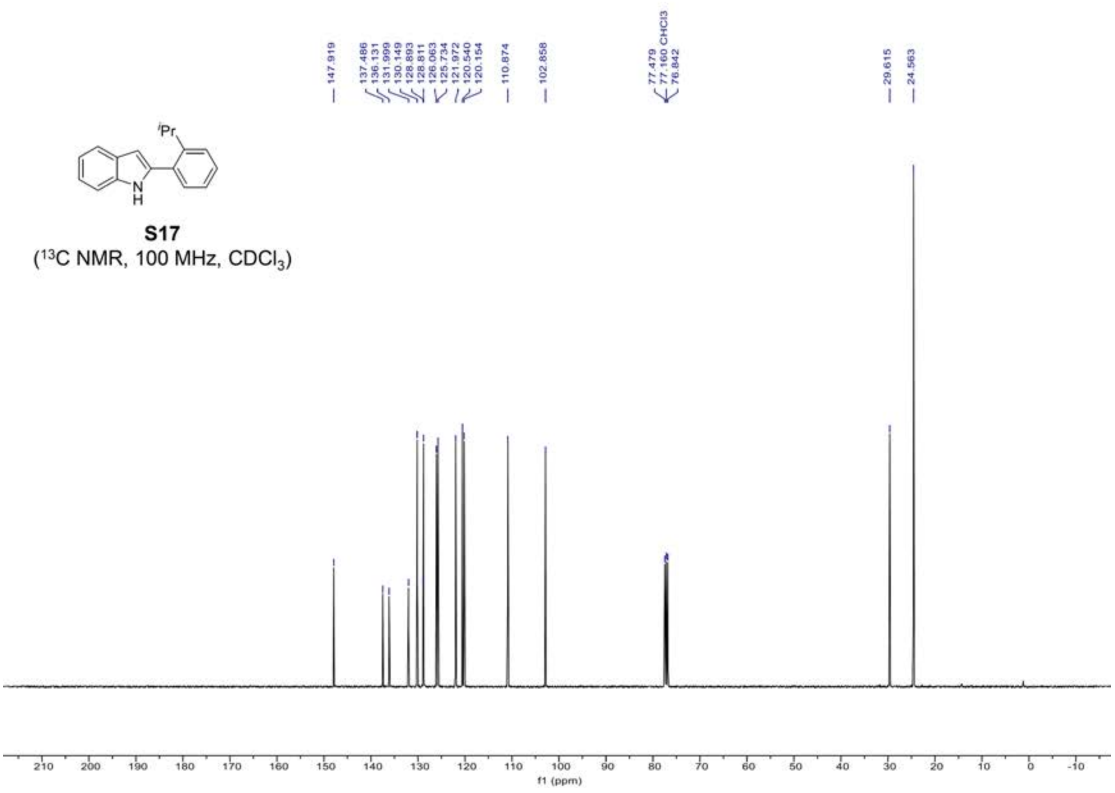

S17
(^{13}C NMR, 100 MHz, $CDCl_3$)

3.2 Synthesis of Substituted 2-Aminoarylindole Intermediates (S20–S27)

3.2.1 Synthesis of *N*-Substituted 2-Nitroarylindoles (S20–S23)

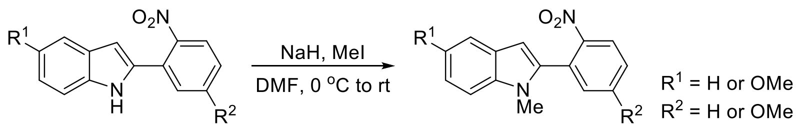

S13, **S14** or **S19** **S20–S22**

Procedure 9: To a round-bottom flask equipped with a magnetic stirring bar, **S13**, **S14** or **S19** (1.0 equiv) was dissolved in DMF (0.2 M) and sodium hydride (1.5 equiv) was slowly added at 0 °C and the resulting mixture was stirred for 30 min. Iodomethane (1.1 equiv) was added and the reaction mixture was warmed to rt and allowed to stir for overnight. Then, the reaction mixture was quenched with water and was extracted three times with EtOAc. The combined organic layer was washed with brine, dried with anhydrous $MgSO_4$ and concentrated *in vacuo*. The crude material was then purified by flash chromatography to afford the desired material **S20**–**S22**.

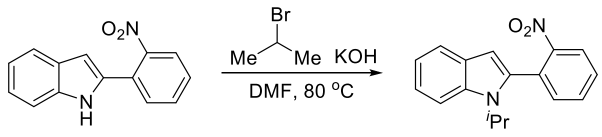

S13 **S23**

Procedure 10: To a round-bottom flask equipped with a magnetic stirring bar, **S13** (1.0 equiv), 2-bromopropane (3.0 equiv), and KOH (3.0 equiv) were added and subsequently dissolved in DMF (0.5 M). The reaction was heated to 80 °C and allowed to stir 1 h. The progress of the reaction was monitored by TLC. After cooled to rt, the reaction mixture was diluted with EtOAc, washed with brine, and the aqueous phase was extracted with EtOAc. The combined organic layers were dried with anhydrous $MgSO_4$ and concentrated *in vacuo*. The crude material was then purified by flash chromatography to afford the desired material **S23**.

Characterization and Spectra of *N*-Substituted 2-Nitroarylindoles (S20–S23)

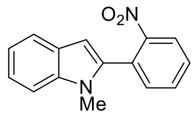

1-Methyl-2-(2-nitrophenyl)-1*H*-indole (S20) was synthesized by following Procedure 9. The crude material was purified by normal-phase column chromatography using an eluent of 20% EtOAc/Hx to provide **S20** (461 mg, 87%).

^{1}H NMR (400 MHz, $CDCl_3$) δ 8.08 (dd, *J* = 8.1, 1.4 Hz, 1H), 7.73–7.66 (m, 2H), 7.65–7.53 (m, 2H), 7.42 (dd, *J* = 8.3, 1.0 Hz, 1H), 7.34 (ddd, *J* = 8.2, 7.0, 1.2 Hz, 1H), 7.22 (ddd, *J* = 8.0, 6.9, 1.1 Hz, 1H), 6.57 (d, *J* = 0.9 Hz, 1H), 3.60 (s, 3H).
^{13}C NMR (100 MHz, $CDCl_3$) δ 149.7, 137.8, 135.8, 133.6, 132.7, 129.7, 127.7, 127.6, 124.3, 122.2, 120.8, 120.0, 109.6, 102.4, 30.7.

The spectral data were identical with those previously reported.[11]

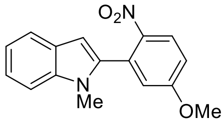

2-(5-Methoxy-2-nitrophenyl)-1-methyl-1*H*-indole (S21) was synthesized by following Procedure 9. The crude material was purified by normal-phase column chromatography using an eluent of 25% EtOAc/Hx to provide **S21** (269 mg, 64%).

^{1}H NMR (400 MHz, $CDCl_3$) δ 8.19 (d, *J* = 9.1 Hz, 1H), 7.65 (d, *J* = 7.8 Hz, 1H), 7.37 (d, *J* = 8.3 Hz, 1H), 7.28 (ddd, *J* = 8.8, 7.6, 1.2 Hz, 1H), 7.16 (ddd, *J* = 7.9, 7.0, 1.1 Hz, 1H), 7.06 (dd, *J* = 9.1, 2.8 Hz, 1H), 6.98 (d, *J* = 2.8 Hz, 1H), 6.51 (d, *J* = 0.9 Hz, 1H), 3.92 (s, 3H), 3.54 (s, 3H).
^{13}C NMR (100 MHz, $CDCl_3$) δ 162.9, 142.4, 137.8, 136.7, 130.6, 127.7, 127.3, 122.2, 120.7, 120.0, 118.7, 114.7, 109.6, 101.8, 56.2, 30.6.
IR (FT-ATR, cm^{-1}, $CHCl_3$) ν_{max} 3950, 3926, 3903, 3880, 3838, 3799, 3776, 1730, 3703, 3626, 3599, 3548, 3390, 3348, 3055, 3016, 2939, 2843, 2727, 2642, 2603, 2506, 2445, 2360, 2337, 2299, 2268, 2191, 2125, 2048, 1917, 1844, 1797, 1770, 1712, 1674, 1608, 1577, 1512, 1462, 1435, 1335, 1281, 1238, 1203, 1173, 1095, 1018, 960, 906, 876, 833, 783, 748, 702, 671.
HRMS (EI) *m*/*z*: $[M]^+$ Calcd for $C_{16}H_{14}N_2O_3$ 282.1004; found 282.1003.

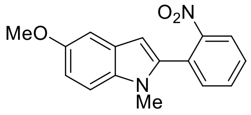

5-Methoxy-1-methyl-2-(2-nitrophenyl)-1*H*-indole (S22) was synthesized by following Procedure 9. The crude material was purified by normal-phase column chromatography using an eluent of 25% EtOAc/Hx to provide **S22** (800 mg, 95%).

^{1}H NMR (400 MHz, $CDCl_3$) δ 8.04 (dd, *J* = 8.1, 1.4 Hz, 1H), 7.69 (td, *J* = 7.5, 1.4 Hz, 1H), 7.65–7.57 (m, 1H), 7.54 (dd, *J* = 7.6, 1.6 Hz, 1H), 7.26 (d, *J* = 8.7 Hz, 1H), 7.10

(d, *J* = 2.4 Hz, 1H), 6.95 (dd, *J* = 8.9, 2.5 Hz, 1H), 6.44 (d, *J* = 0.9 Hz, 1H), 3.87 (s, 3H), 3.54 (s, 3H).
^{13}C NMR (100 MHz, $CDCl_3$) δ 154.5, 149.8, 136.2, 133.6, 133.3, 132.7, 129.7, 128.1, 127.7, 124.4, 112.7, 110.4, 102.4, 102.1, 56.0, 30.9.

The spectral data were identical with those previously reported.[12]

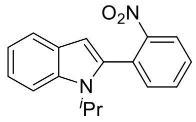

1-Isopropyl-2-(2-nitrophenyl)-1*H*-indole (S23) was synthesized by following Procedure 10. The crude material was purified by normal-phase column chromatography using an eluent of 17% CH_2Cl_2/Hx to provide **S23** (353 mg, 63%).

^{1}H NMR (400 MHz, $CDCl_3$) δ 8.01 (dd, *J* = 8.0, 1.5 Hz, 1H), 7.83–7.53 (m, 4H), 7.49 (dd, *J* = 7.5, 1.6 Hz, 1H), 7.22 (ddd, *J* = 8.5, 7.0, 1.3 Hz, 1H), 7.13 (ddd, *J* = 7.9, 7.1, 0.9 Hz, 1H), 6.39 (s, 1H), 4.28 (hept, *J* = 6.9 Hz, 1H), 1.65 (d, *J* = 6.6 Hz, 3H), 1.56 (d, *J* = 8.0 Hz, 3H).
^{13}C NMR (100 MHz, $CDCl_3$) δ 150.1, 135.3, 135.2, 133.4, 132.4, 129.7, 129.1, 128.6, 124.2, 121.7, 121.3, 119.7, 112.3, 102.4, 49.0, 21.9, 21.2.
IR (FT-ATR, cm^{-1}, $CHCl_3$) ν_{max} 3907, 3884, 3861, 3842, 3818, 3757, 3737, 3680, 3656, 3633, 3614, 3595, 3575, 3552, 3491, 3467, 3402, 3367, 3344, 3302, 3209, 3055, 2978, 2935, 2873, 2769, 2708, 2669, 2607, 2507, 2438, 2376, 2357, 2337, 2318, 2252, 2071, 1986, 1921, 1874, 1840, 1801, 1678, 1612, 1574, 1527, 1454, 1408, 1346, 1304, 1261, 1215, 1176, 1138, 1107, 1084, 1014, 957, 927, 883, 852, 783, 744, 706, 675.
HRMS (EI) *m*/*z*: $[M]^+$ Calcd for $C_{17}H_{16}N_2O_2$ 280.1212; found 280.1209.

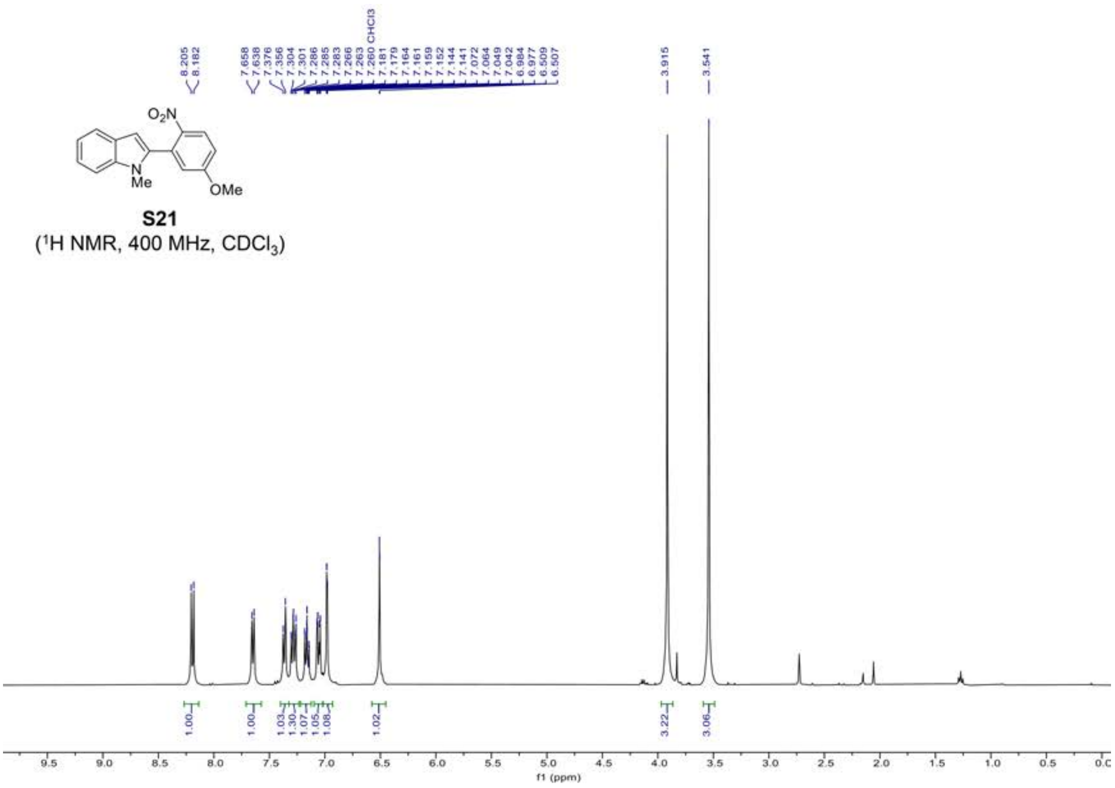

O2N
N
Me
OMe
S21
(1H NMR, 400 MHz, CDCl3)
f1 (ppm)

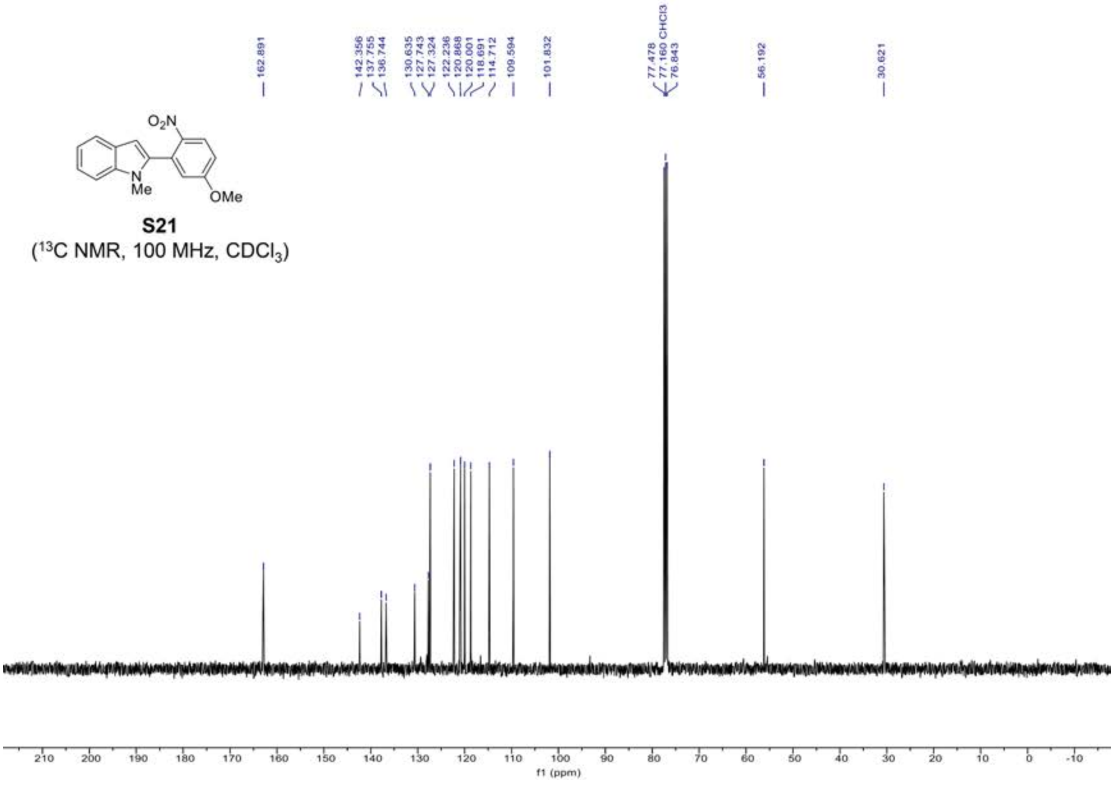

O2N
N
Me
OMe
S21
(13C NMR, 100 MHz, CDCl3)
f1 (ppm)

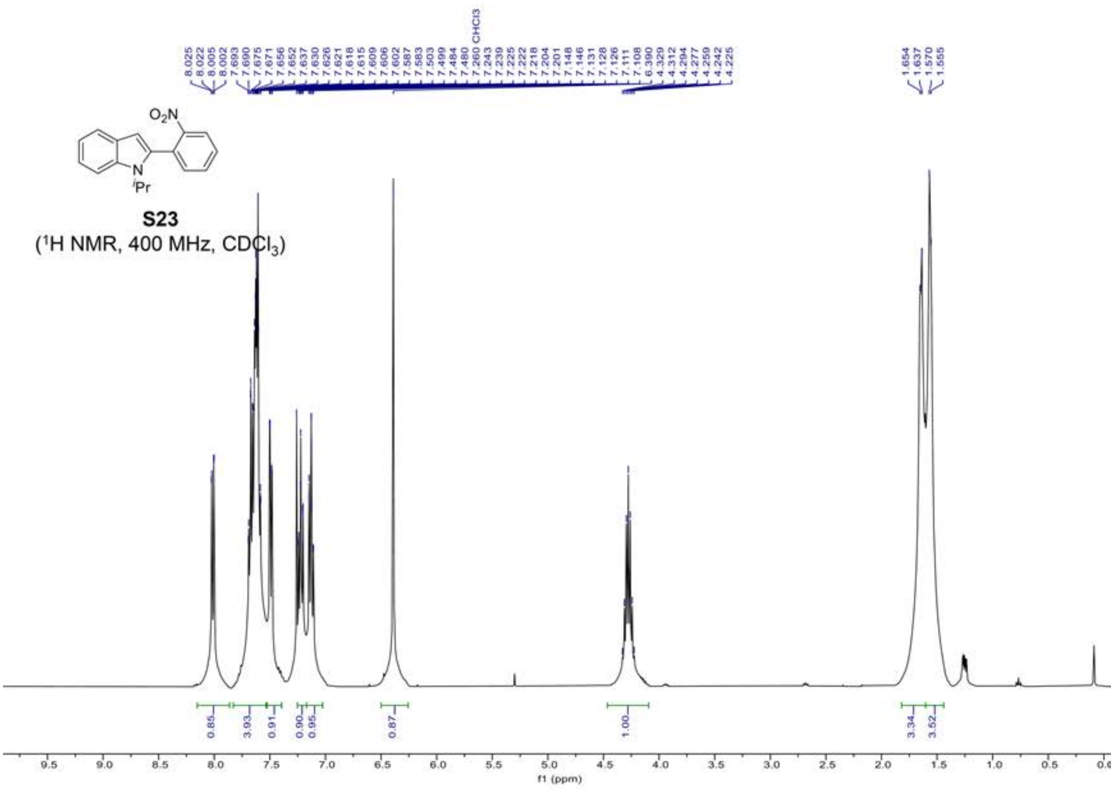

S23
(^{1}H NMR, 400 MHz, $CDCl_3$)

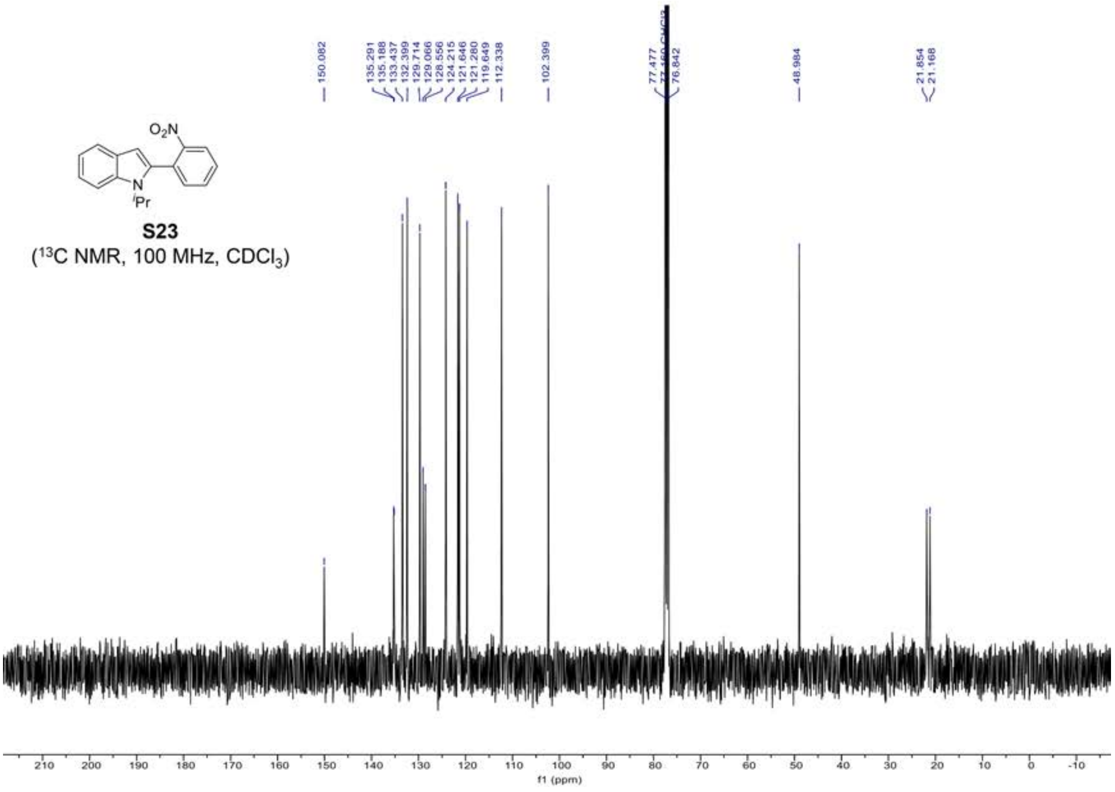

S23
(^{13}C NMR, 100 MHz, $CDCl_3$)

3.2.2 Synthesis of Substituted 2-Aminoarylindole Intermediates (S24–S27)

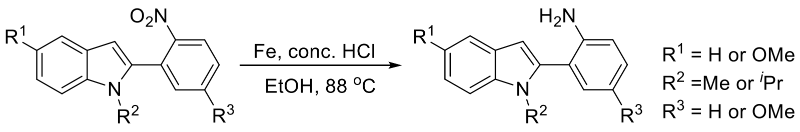

S20–S23 **S24–S27**

Procedure 11: To a reaction tube equipped with a magnetic stirring bar, **S20**–**S23** (1.0 equiv) and iron powder (5.0 equiv) were added and subsequently dissolved in EtOH (0.25 M). Then, the concentrated hydrogen chloride (12.0 equiv) was added under nitrogen atmosphere. The reaction was heated to 88 °C and allowed to stir for 4 h. The progress of the reaction was monitored by TLC. After cooled to rt, the reaction mixture was neutralized by 1 M aqueous NaOH and extracted with EtOAc three times. The combined organic layer was washed with brine, dried with anhydrous $MgSO_4$, and concentrated *in vacuo*. The crude material was then purified by flash chromatography to afford the desired material **S24**–**S27**.

Characterization and Spectra of Substituted 2-Aminoarylindole Intermediates (S24–S27)

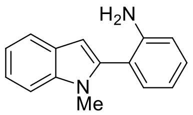

2-(1-Methyl-1*H*-indol-2-yl)aniline (S24) was synthesized by following Procedure 11. The crude material was purified by normal-phase column chromatography using an eluent of 20% EtOAc/Hx to provide **S24** (290 mg, 81%).

^{1}H NMR (400 MHz, $CDCl_3$) δ 7.68 (dt, *J* = 7.8, 1.1 Hz, 1H), 7.40 (dq, *J* = 8.2, 1.0 Hz, 1H), 7.32–7.23 (m, 2H), 7.23–7.13 (m, 2H), 6.92–6.79 (m, 2H), 6.57 (d, *J* = 0.8 Hz, 1H), 3.84 (s, 2H), 3.62 (s, 3H).
^{13}C NMR (100 MHz, $CDCl_3$) δ 145.7, 138.2, 137.8, 131.7, 129.9, 128.2, 121.7, 120.6, 119.9, 118.1, 118.0, 115.4, 109.7, 101.9, 30.6.

The spectral data were identical with those previously reported.[13]

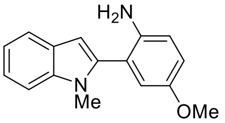

4-Methoxy-2-(1-methyl-1*H*-indol-2-yl)aniline (S25) was synthesized by following Procedure 11. The crude material was purified by normal-phase column chromatography using an eluent of 50% CH_2Cl_2/Hx to provide **S25** (73 mg, 42%).

^{1}H NMR (400 MHz, $CDCl_3$) δ 7.66 (dt, *J* = 7.9, 1.0 Hz, 1H), 7.39 (d, *J* = 8.2 Hz, 1H), 7.33–7.23 (m, 1H), 7.17 (td, *J* = 7.4, 1.1 Hz, 1H), 6.88 (dd, *J* = 8.7, 3.0 Hz, 1H), 6.84–6.65 (m, 2H), 6.56 (s, 1H), 3.79 (s, 3H), 3.63 (s, 3H).
^{13}C NMR (100 MHz, $CDCl_3$) δ 152.3, 139.4, 138.1, 137.9, 128.2, 121.8, 120.6, 119.9, 119.1, 116.8, 116.7, 116.2, 109.7, 102.0, 56.0, 30.7.
IR (FT-ATR, cm^{-1}, $CHCl_3$) ν_{max} 3926, 3899, 3838, 3799, 3780, 3730, 3703, 3626, 3599, 3545, 3525, 3437, 3356, 3209, 3163, 3051, 2997, 2939, 2831, 2711, 2360, 2333, 2299, 2268, 2071, 1921, 1890, 1797, 1770, 1743, 1604, 1547, 1496, 1466, 1435, 1362, 1335, 1311, 1277, 1230, 1196, 1165, 1099, 1038, 960, 906, 876, 814, 748, 671.
HRMS (EI) *m*/*z*: $[M]^+$ Calcd for $C_{16}H_{16}N_2O$ 252.1263; found 252.1261.

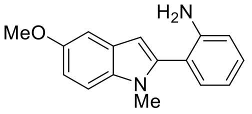

2-(5-Methoxy-1-methyl-1*H*-indol-2-yl)aniline (S26) was synthesized by following Procedure 11. The crude material was purified by normal-phase column chromatography using an eluent of 25% EtOAc/Hx to provide **S26** (470 mg, 64%).

^{1}H NMR (400 MHz, $CDCl_3$) δ 7.32–7.22 (m, 2H), 7.20 (dd, *J* = 7.6, 1.6 Hz, 1H), 7.14 (d, *J* = 2.5 Hz, 1H), 6.94 (dd, *J* = 8.9, 2.5 Hz, 1H), 6.89–6.77 (m, 2H), 6.49 (d, *J* = 0.9 Hz, 1H), 3.90 (s, 3H), 3.84 (s, 2H), 3.59 (s, 3H).

^{13}C NMR (100 MHz, $CDCl_3$) δ 154.4, 145.7, 138.7, 133.2, 131.7, 129.9, 128.5, 118.1, 118.0, 115.3, 111.9, 110.4, 102.3, 101.5, 56.1, 30.7.

The spectral data were identical with those previously reported.[12]

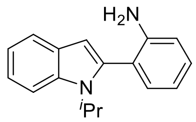

2-(1-Isopropyl-1*H*-indol-2-yl)aniline (S27) was synthesized by following Procedure 11. The crude material was purified by normal-phase column chromatography using an eluent of 40% EtOAc/Hx to provide **S27** (280 mg, 88%).

^{1}H NMR (400 MHz, $CDCl_3$) δ 7.65 (t, *J* = 8.2 Hz, 2H), 7.38–7.02 (m, 4H), 6.96–6.71 (m, 2H), 6.48 (s, 1H), 4.42 (hept, *J* = 7.0 Hz, 1H), 3.56 (brs, 2H), 1.64 (d, *J* = 7.0 Hz, 3H), 1.53 (d, *J* = 7.0 Hz, 3H).
^{13}C NMR (100 MHz, $CDCl_3$) δ 145.9, 137.6, 135.3, 131.7, 129.9, 129.3, 121.1, 120.9, 119.4, 118.8, 118.1, 115.2, 112.3, 102.2, 48.2, 21.8, 21.6.
IR (FT-ATR, cm^{-1}, $CHCl_3$) ν_{max} 3946, 3926, 3903, 3880, 3842, 3822, 3803, 3780, 3757, 3718, 3680, 3653, 3629, 3614, 3591, 3568, 3545, 3464, 3371, 3205, 3051, 2974, 2935, 2877, 2808, 2734, 2704, 2654, 2611, 2507, 2403, 2372, 2341, 2314, 2252, 2210, 2148, 2102, 2009, 1925, 1894, 1797, 1739, 1720, 1682, 1612, 1574, 1543, 1489, 1454, 1404, 1342, 1300, 1254, 1219, 1176, 1157, 1134, 1107, 1014, 968, 933, 883, 845, 787, 741, 675.
HRMS (EI) *m*/*z*: $[M]^+$ Calcd for $C_{17}H_{18}N_2$ 250.1470; found 250.1470.

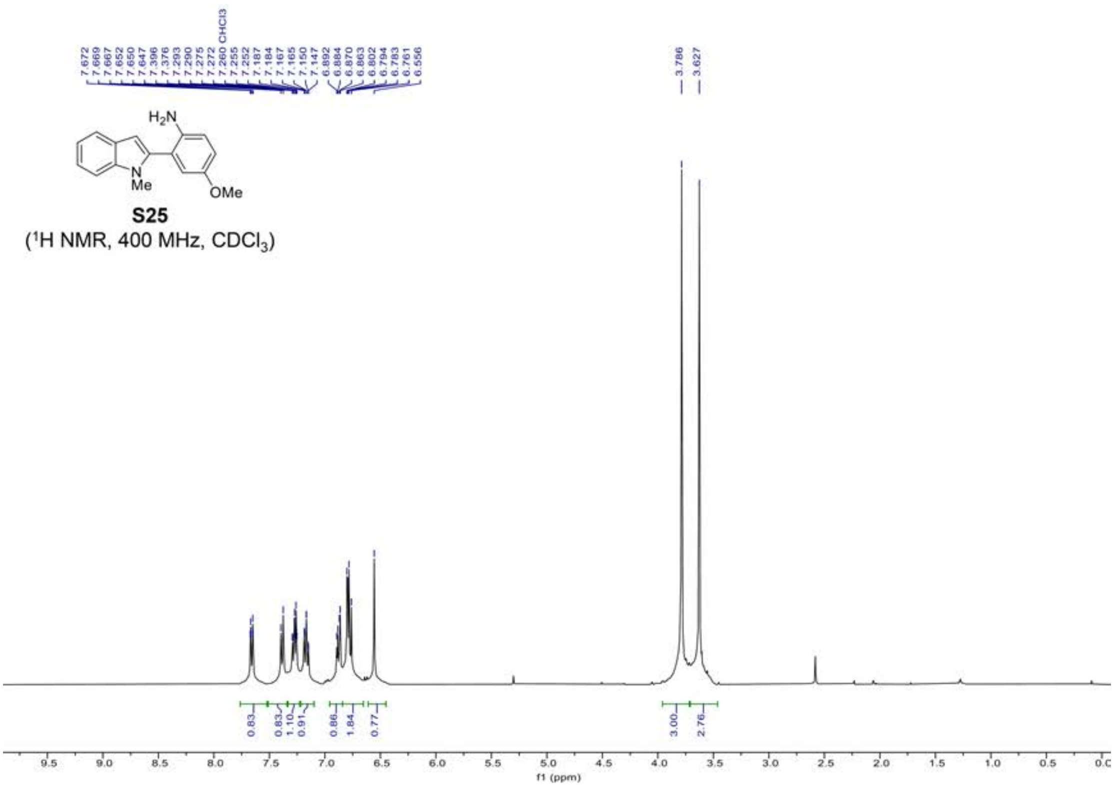

S25
(^{1}H NMR, 400 MHz, $CDCl_3$)

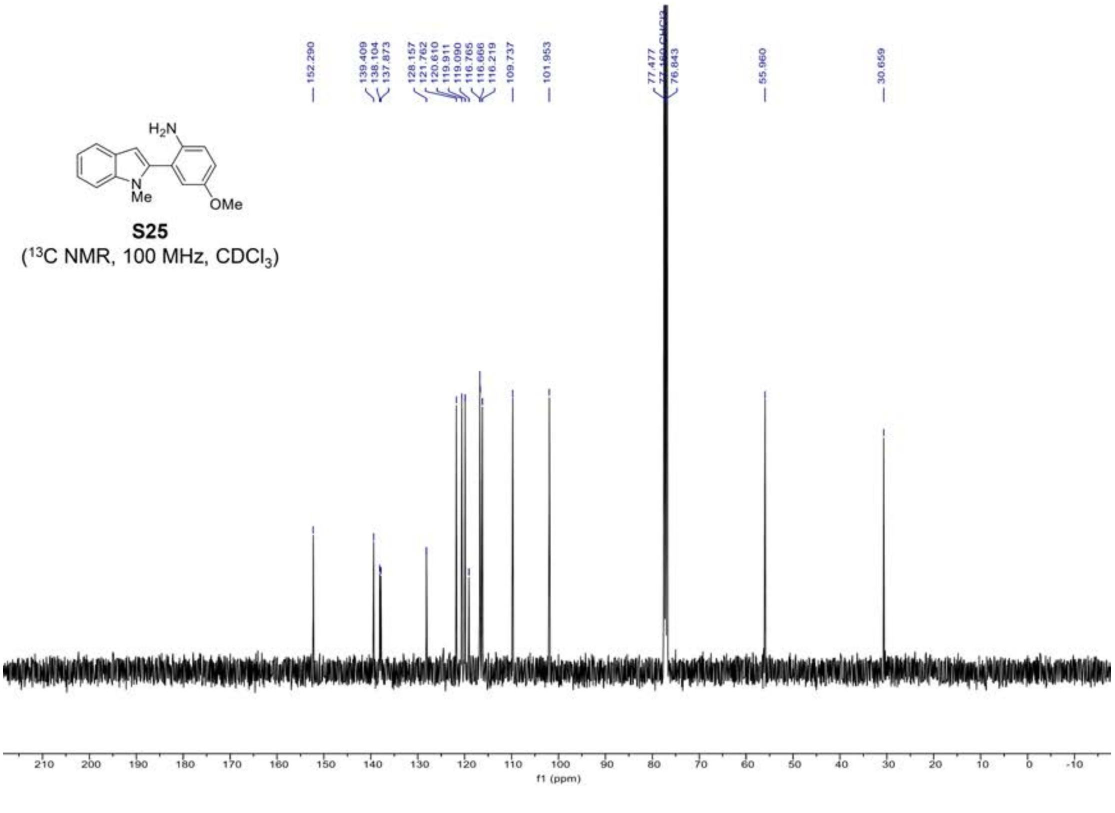

S25
(^{13}C NMR, 100 MHz, $CDCl_3$)

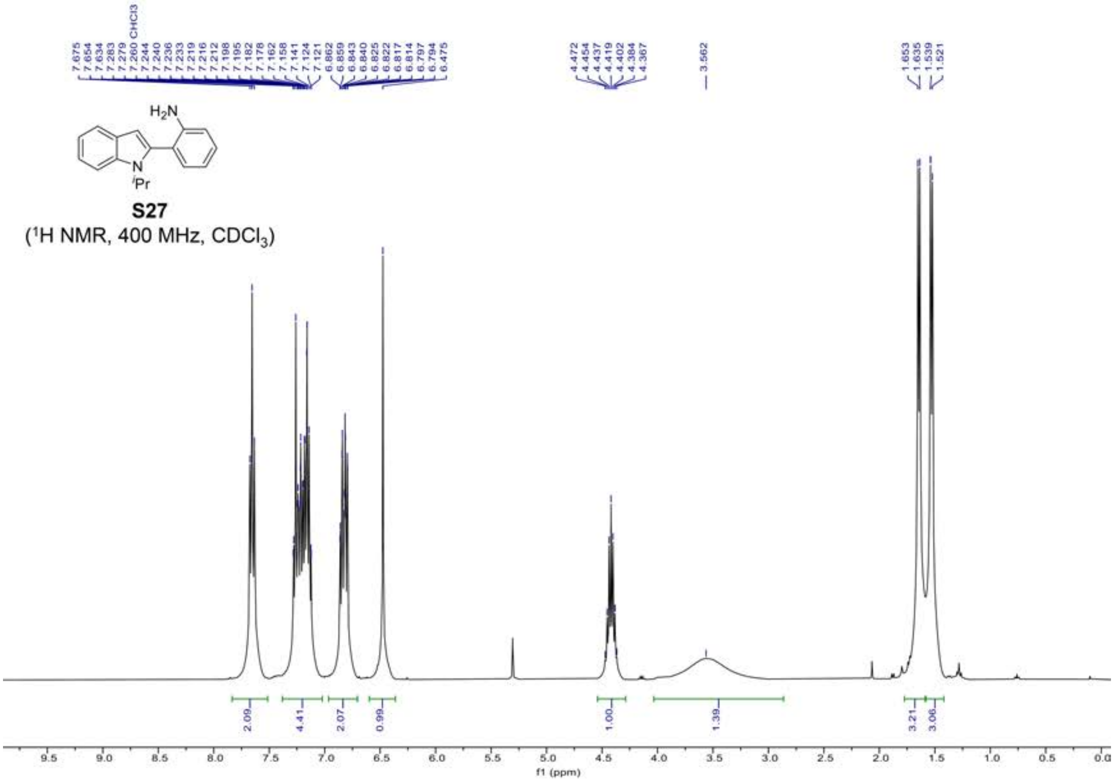
H2N
N
iPr
S27
(1H NMR, 400 MHz, CDCl3)
f1 (ppm)

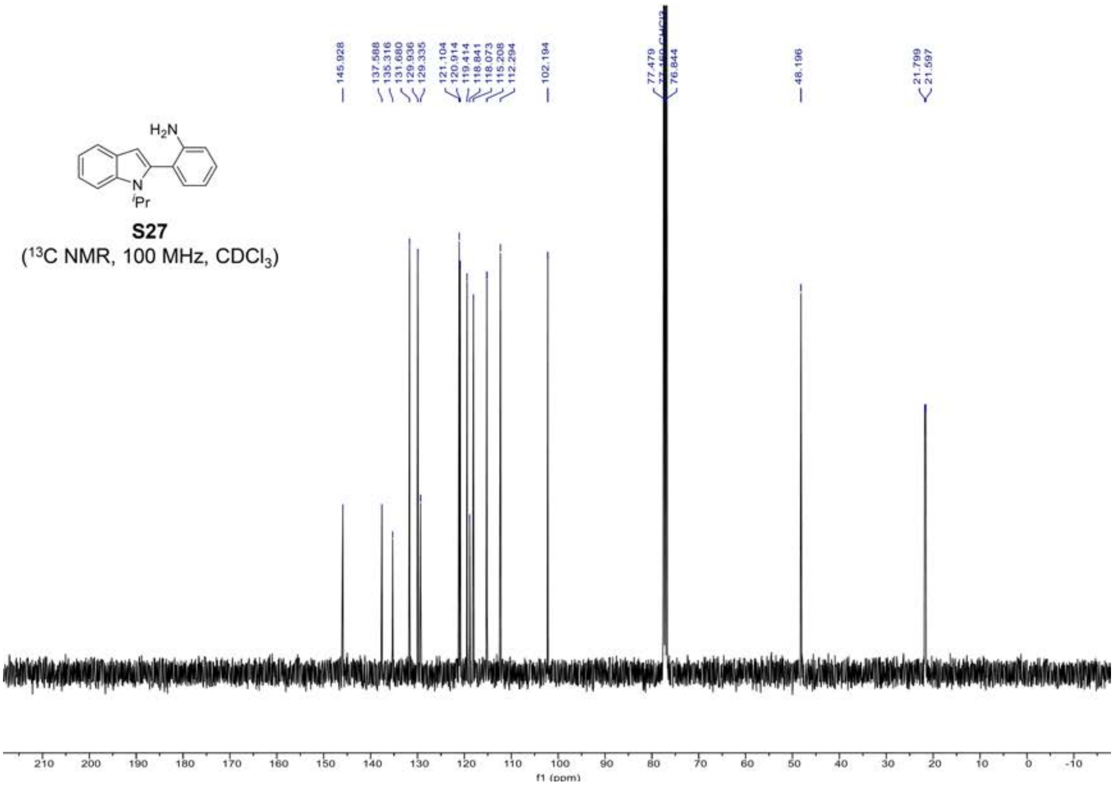
H2N
N
iPr
S27
(13C NMR, 100 MHz, CDCl3)
f1 (ppm)

3.3 Synthesis of 4

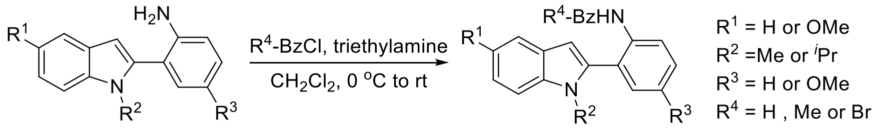

S24–S27 **4a–4d**, **4j** or **4k**

Procedure 12: To a round-bottom flask equipped with a magnetic stirring bar, **S24–S27** (1.0 equiv), and triethylamine (2.0 equiv) were added and subsequently dissolved in CH_2Cl_2 (0.4 M). Then, benzoyl chloride (1.2 equiv) was added dropwise and allowed to stir for overnight at rt. The reaction was diluted with CH_2Cl_2, transferred to a separatory funnel, and quenched with a saturated aqueous NH_4Cl. The organic layer was separated, and the aqueous layer was extracted an additional two times with CH_2Cl_2. The combined organic layers were then rinsed with water, dried with anhydrous $MgSO_4$, and concentrated *in vacuo*. The crude material was then purified by flash chromatography to afford the desired material **4a**–**4d**, **4j** and **4k**.

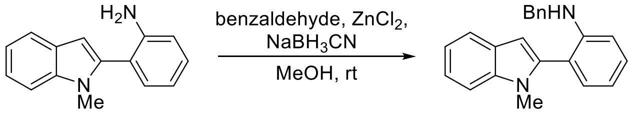

S24 **4e**

Procedure 13: To a round-bottom flask equipped with a magnetic stirring bar, **S24** (1.0 equiv), $ZnCl_2$ (1.2 equiv) and benzaldehyde (1.2 equiv) were added and subsequently dissolved in MeOH (0.25 M). Then, $NaBH_3CN$ (1.1 equiv) was added and the reaction mixture was stirred for 5 h at rt. The reaction mixture was quenched with 1 N aqueous NaOH and extracted three times with EtOAc. The combined organic layer was washed with brine, dried over $MgSO_4$, and concentrated *in vacuo*. The crude material was then purified by flash chromatography to afford the desired material **4e**.

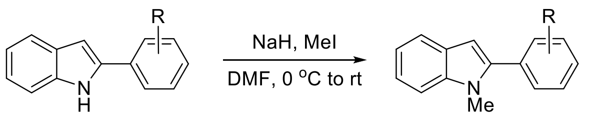

S15–S18 **4f–4i**

Procedure 14: To a round-bottom flask equipped with a magnetic stirring bar, NaH (1.0 equiv) and THF (0.1 M) were added. **S15**–**S18** (1.0 equiv) was added at 0 ºC and the mixture was stirred for 30 min. Iodomethane (1.5 equiv) was added and the reaction mixture was warmed to room temperature and allowed to stir for three days. Then, the reaction mixture was quenched with water and was extracted three times with EtOAc. The combined organic layers were washed with brine, dried with anhydrous MgSO4, filtered with EtOAc, and concentrated *in vacuo*. The crude material was then purified by flash chromatography to afford the desired material **4f**–**4i**.

Characterization and Spectra of 4

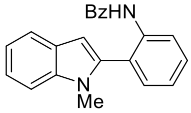

***N*-(2-(1-Methyl-1*H*-indol-2-yl)phenyl)benzamide (4a)** was synthesized by following Procedure 12 from **S22**. The crude material was purified by normal-phase column chromatography using an eluent of 67% CH_2Cl_2/Hx to provide **4a** (610 mg, 88%).

^{1}H NMR (400 MHz, $CDCl_3$) δ 8.70 (dd, *J* = 8.3, 1.2 Hz, 1H), 8.50 (s, 1H), 7.72 (dt, *J* = 7.9, 1.0 Hz, 1H), 7.65–7.59 (m, 2H), 7.54 (ddd, *J* = 8.6, 7.4, 1.7 Hz, 1H), 7.49–7.43 (m, 1H), 7.43–7.30 (m, 5H), 7.27 (td, *J* = 7.5, 1.2 Hz, 1H), 7.23 (ddd, *J* = 8.0, 7.0, 1.1 Hz, 1H), 6.69 (d, *J* = 0.9 Hz, 1H), 3.61 (s, 3H).
^{13}C NMR (100 MHz, $CDCl_3$) δ 165.2, 138.3, 137.1, 136.0, 134.5, 132.0, 131.2, 130.0, 128.9 (2C), 128.0, 127.0 (2C), 123.9, 122.5, 122.2, 120.9, 120.4, 109.9, 102.8, 30.8.

The spectral data were identical with those previously reported.[14]

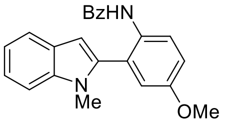

***N*-(4-Methoxy-2-(1-methyl-1*H*-indol-2-yl)phenyl)benzamide (4b)** was synthesized by following Procedure 12 from **S23**. The crude material was purified by normal-phase column chromatography using an eluent of 25% EtOAc/Hx to provide **4b** (75 mg, 81%).

^{1}H NMR (400 MHz, $CDCl_3$) δ 8.52 (d, *J* = 9.1 Hz, 1H), 8.20 (s, 1H), 7.70 (d, *J* = 7.9 Hz, 1H), 7.65–7.50 (m, 2H), 7.50–7.27 (m, 5H), 7.21 (ddd, *J* = 7.9, 7.0, 1.1 Hz, 1H), 7.08 (dd, *J* = 9.1, 3.0 Hz, 1H), 6.92 (d, *J* = 3.0 Hz, 1H), 6.66 (s, 1H), 3.86 (s, 3H), 3.60 (s, 3H).
^{13}C NMR (100 MHz, $CDCl_3$) δ 165.0, 155.9, 138.3, 136.1, 134.7, 131.8, 130.3, 128.9 (2C), 127.9, 126.9 (2C), 124.1, 122.9, 122.5, 120.9, 120.4, 116.7, 115.0, 109.9, 102.8, 55.7, 30.9.
IR (FT-ATR, cm^{-1}, $CHCl_3$) ν_{max} 3950, 3930, 3907, 3884, 3826, 3803, 3780, 3757, 3717, 3676, 3653, 3629, 3614, 3591, 3568, 3545, 3525, 3502, 3394, 3336, 3055, 3008, 2943, 2835, 2603, 2515, 2372, 2349, 2314, 2249, 2195, 2129, 2063, 1963, 1917, 1894, 1847, 1747, 1666, 1608, 1512, 1466, 1439, 1408, 1381, 1362, 1335, 1308, 1277, 1215, 1165, 1134, 1099, 1034, 903, 876, 814, 791, 744, 706, 663.
HRMS (EI) *m*/*z*: $[M]^+$ Calcd for $C_{23}H_{20}N_2O_2$ 356.1525; found 356.1524.

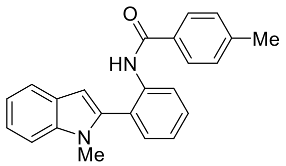

4-Methyl-*N*-(2-(1-methyl-1*H*-indol-2-yl)phenyl)benzamide (4c) was synthesized by following Procedure 12 from **S22**. The crude material was purified by normal-phase column chromatography using an eluent of 25% CH_2Cl_2/Hx to provide **4c** (150 mg, 98%).

^{1}H NMR (400 MHz, $CDCl_3$) δ 8.59 (d, *J* = 8.3 Hz, 1H), 8.33 (brs, 1H), 7.61 (d, *J* = 7.9 Hz, 1H), 7.50–7.35 (m, 3H), 7.35–7.19 (m, 3H), 7.16–7.09 (m, 2H), 7.05 (s, 1H), 7.03 (s, 1 H), 6.58 (s, 1H), 3.49 (s, 3H), 2.24 (s, 3H).
^{13}C NMR (100 MHz, $CDCl_3$) δ 165.1, 142.6, 138.3, 137.2, 136.1, 131.7, 131.2, 130.0, 129.6 (2C), 128.0, 127.0 (2C), 123.8, 122.5, 122.2, 120.9, 120.8, 120.4, 109.9, 102.8, 30.8, 21.5.
IR (FT-ATR, cm^{-1}, $CHCl_3$) ν_{max} 3950, 3907, 3842, 3807, 3780, 3757, 3718, 3676, 3653, 3629, 3614, 3591, 3518, 3545, 3525, 3502, 3390, 3167, 3051, 3012, 2943, 2839, 2731, 2584, 2507, 2434, 2403, 2372, 2345, 2314, 2206, 2144, 1921, 1890, 1851, 1801, 1743, 1674, 1612, 1581, 1504, 1441, 1377, 1335, 1304, 1250, 1215, 1188, 1119, 1095, 1041, 1011, 949, 922, 895, 841, 791, 741, 667.
HRMS (EI) *m*/*z*: $[M]^+$ Calcd for $C_{23}H_{20}N_2O$ 340.1576; found 340.1574.

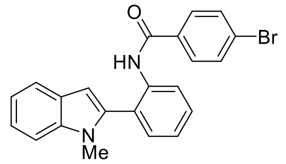

4-Bromo-*N*-(2-(1-methyl-1*H*-indol-2-yl)phenyl)benzamide (4d) was synthesized by following Procedure 12 from **S22**. The crude material was purified by normal-phase column chromatography using an eluent of 50% EtOAc/Hx to provide **4d** (177 mg, 97%).

^{1}H NMR (400 MHz, $CDCl_3$) δ 8.64 (d, *J* = 8.1 Hz, 1H), 8.43 (brs, 1H), 7.70 (d, *J* = 7.9 Hz, 1H), 7.60–7.12 (m, 10H), 6.66 (s, 1H), 3.59 (s, 3H).
^{13}C NMR (100 MHz, $CDCl_3$) δ 164.2, 138.3, 136.9, 135.8, 133.4, 132.2 (2C) 131.2, 130.1, 128.6 (2C), 127.9, 126.8, 124.2, 122.7, 122.3, 120.9, 120.9, 120.6, 110.0, 102.8, 30.9.
IR (FT-ATR, cm^{-1}, $CHCl_3$) ν_{max} 3950, 3930, 3907, 3880, 3826, 3807, 3780, 3753, 3718, 3676, 3652, 3629, 3614, 3591, 3568, 3545, 3525, 3502, 3383, 3055, 3016, 2935, 2843, 2719, 2576, 2507, 2434, 2372, 2349, 2314, 2206, 2161, 2044, 1959, 1925, 1851, 1674, 1612, 1581, 1516, 1481, 1442, 1381, 1338, 1308, 1250, 1165, 1095, 1072, 1045, 1007, 949, 895, 845, 795, 741, 714, 687.
HRMS (EI) *m*/*z*: $[M]^+$ Calcd for $C_{22}H_{17}BrN_2O$ 404.0524; found 404.0524.

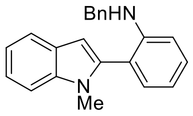

***N*-Benzyl-2-(1-methyl-1*H*-indol-2-yl)aniline (4e)** was synthesized by following Procedure 13 from **S22**. The crude material was purified by normal-phase column chromatography using an eluent of 20% CH_2Cl_2/Hx to provide **4e** (220 mg, 52%).

^{1}H NMR (400 MHz, $CDCl_3$) δ 7.71 (d, J = 7.8 Hz, 1H), 7.43 (d, J = 8.2 Hz, 1H), 7.40–7.16 (m, 9H), 6.85 (td, J = 7.4, 1.1 Hz, 1H), 6.74 (d, J = 8.2 Hz, 1H), 6.64 (s, 1H), 4.57 (brs, 1H), 4.38 (s, 2H), 3.66 (s, 3H).
^{13}C NMR (100 MHz, $CDCl_3$) δ 146.9, 139.4, 138.0, 137.9, 131.7, 130.2, 128.7 (2C), 128.2, 127.2, 127.1 (2C), 121.7, 120.6, 119.9, 117.8, 116.8, 110.9, 109.7, 102.2, 47.9, 30.6.
IR (FT-ATR, cm^{-1}, $CHCl_3$) ν_{max} 3950, 3907, 3880, 3842, 3807, 3780, 3757, 3718, 3676, 3653, 3629, 3591, 3568, 3548, 3525, 3413, 3213, 3056, 3012, 2939, 2843, 2711, 2607, 2507, 2403, 2372, 2345, 2318, 2245, 2133, 2060, 1921, 1890, 1743, 1678, 1651, 1604, 1577, 1543, 1504, 1458, 1362, 1311, 1215, 1165, 1126, 1099, 1065, 1026, 1003, 926, 845, 741, 698, 667.
HRMS (EI) *m*/*z*: [M]$^+$ Calcd for $C_{22}H_{20}N_2$ 312.1627; found 312.1626.

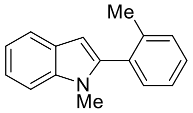

1-Methyl-2-(*o*-tolyl)-1*H*-indole (4f) was synthesized by following Procedure 14 from **S13**. The crude material was purified by normal-phase column chromatography using an eluent of 9% EtOAc/Hx to provide **4f** (158 mg, 86%).

^{1}H NMR (400 MHz, $CDCl_3$) δ 7.79 (dt, J = 7.8, 1.1 Hz, 1H), 7.53–7.36 (m, 6H), 7.30 (ddd, J = 8.0, 7.0, 1.1 Hz, 1H), 6.59 (d, J = 0.9 Hz, 1H), 3.63 (s, 3H), 2.34 (s, 3H).
^{13}C NMR (100 MHz, $CDCl_3$) δ 140.6, 138.1, 137.4, 132.6, 131.2, 130.2, 128.8, 128.1, 125.7, 121.4, 120.5, 119.8, 109.5, 101.6, 30.4, 20.2.

The spectral data were identical with those previously reported.[7]

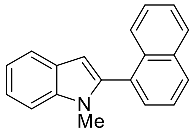

1-Methyl-2-(naphthalen-1-yl)-1*H*-indole (4g) was synthesized by following Procedure 14 from **S14**. The crude material was purified by normal-phase column chromatography using an eluent of 17% CH_2Cl_2/Hx to provide **4g** (78 mg, 95%).

^{1}H NMR (400 MHz, $CDCl_3$) δ 8.02–7.92 (m, 2H), 7.80–7.71 (m, 2H), 7.63–7.51 (m, 3H), 7.51–7.42 (m, 2H), 7.34 (ddd, J = 8.2, 7.1, 1.2 Hz, 1H), 7.28–7.20 (m, 1H), 6.68 (d, J = 0.9 Hz, 1H), 3.53 (s, 3H).
^{13}C NMR (100 MHz, $CDCl_3$) δ 139.5, 137.8, 133.6, 133.1, 130.7, 129.1, 129.1, 128.4, 128.2, 126.8, 126.2 (2C), 125.3, 121.7, 120.7, 119.9, 109.6, 103.2, 30.9.

The spectral data were identical with those previously reported.[15]

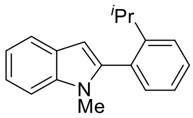

2-(2-Isopropylphenyl)-1-methyl-1*H*-indole (4h) was synthesized by following Procedure 14 from **S15**. The crude material was purified by normal-phase column chromatography using an eluent of 9% EtOAc/Hx to provide **4h** (150 mg, 71%).

^{1}H NMR (400 MHz, $CDCl_3$) δ 7.82 (dt, J = 7.8, 1.0 Hz, 1H), 7.63–7.57 (m, 2H), 7.52 (dq, J = 8.2, 1.0 Hz, 1H), 7.46–7.38 (m, 3H), 7.33 (ddd, J = 8.0, 7.0, 1.1 Hz, 1H), 6.63 (d, J = 0.9 Hz, 1H), 3.65 (s, 3H), 3.08 (hept, J = 6.9 Hz, 1H), 1.32 (s, 6H).
^{13}C NMR (100 MHz, $CDCl_3$) δ 149.2, 140.2, 137.2, 131.33, 131.32, 129.3, 128.1, 125.6, 125.5, 121.3, 120.5, 119.8, 109.5, 102.0, 30.5, 30.2, 25.1, 23.3.

The spectral data were identical with those previously reported.[16]

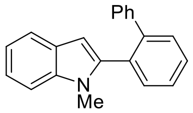

2-([1,1'-Biphenyl]-2-yl)-1-methyl-1*H*-indole (4i) was synthesized by following Procedure 14 from **S16**. The crude material was purified by normal-phase column chromatography using an eluent of 17% CH_2Cl_2/Hx to provide **4i** (203 mg, 94%).

^{1}H NMR (400 MHz, $CDCl_3$) δ 7.81–7.75 (m, 1H), 7.72–7.62 (m, 3H), 7.56 (td, J = 7.3, 1.7 Hz, 1H), 7.40–7.23 (m, 8H), 6.69 (d, J = 0.8 Hz, 1H), 3.18 (s, 3H).
^{13}C NMR (100 MHz, $CDCl_3$) δ 141.6, 141.0, 140.9, 137.3, 132.6, 131.2, 130.2, 129.1, 128.9 (2C), 128.3 (2C), 128.1, 127.4, 127.0, 121.3, 120.4, 119.6, 109.5, 102.6, 30.4.

The spectral data were identical with those previously reported.[17]

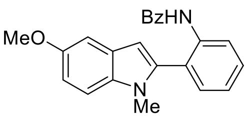

***N*-(2-(5-Methoxy-1-methyl-1*H*-indol-2-yl)phenyl)benzamide (4j)** was synthesized by following Procedure 12 from **S24**. The crude material was purified by normal-phase column chromatography using an eluent of 25% EtOAc/Hx to provide **4j** (509 mg, 81%).

^{1}H NMR (400 MHz, $CDCl_3$) δ 8.68 (d, J = 8.3 Hz, 1H), 8.48 (brs, 1H), 7.61 (dt, J = 7.0, 1.4 Hz, 2H), 7.52 (ddd, J = 8.6, 7.4, 1.7 Hz, 1H), 7.49–7.42 (m, 1H), 7.41–7.32 (m, 3H), 7.29 (d, J = 8.9 Hz, 1H), 7.25 (td, J = 7.5, 1.2 Hz, 1H), 7.16 (d, J = 2.5 Hz, 1H), 6.98 (dd, J = 8.9, 2.4 Hz, 1H), 6.59 (s, 1H), 3.90 (s, 3H), 3.56 (s, 3H).
^{13}C NMR (100 MHz, $CDCl_3$) δ 165.1, 154.7, 137.1, 136.5, 134.6, 133.7, 132.0, 131.1, 130.0, 128.9 (2C), 128.4, 127.0 (2C), 123.9, 122.3, 120.9, 112.7, 110.7, 102.5, 102.4, 56.0, 30.9.
IR (FT-ATR, cm^{-1}, $CHCl_3$) ν_{max} 3973, 3953, 3930, 3907, 3872, 3842, 3826, 3807, 3757, 3714, 3676, 3653, 3614, 3591, 3568, 3545, 3525, 3502, 3383, 3062, 3008, 2943, 2831, 2583, 2430, 2372, 2345, 2322, 2245, 2164, 2056, 1963, 1921, 1813, 1674, 1616, 1577,

1516, 1481, 1450, 1461, 1385, 1342, 1300, 1238, 1211, 1176, 1138, 1099, 1076, 1030, 945, 895, 841, 795, 748, 717, 706, 663.
HRMS (EI) *m*/*z*: $[M]^+$ Calcd for $C_{23}H_{20}N_2O_2$ 356.1525; found 356.1522.

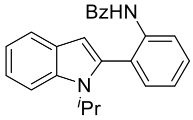

***N*-(2-(1-Isopropyl-1*H*-indol-2-yl)phenyl)benzamide (4k)** was synthesized by following Procedure 12 from **S25**. The crude material was purified by normal-phase column chromatography using an eluent of 67% EtOAc/Hx to provide **4k** (230 mg, 65%).

^{1}H NMR (400 MHz, $CDCl_3$) δ 8.66 (d, *J* = 8.4 Hz, 1H), 8.35 (brs, 1H), 7.71 (d, *J* = 7.1 Hz, 1H), 7.63 (d, *J* = 8.3 Hz, 1H), 7.60–7.49 (m, 3H), 7.49–7.40 (m, 1H), 7.40–7.29 (m, 3H), 7.29–7.22 (m, 2H), 7.18 (t, *J* = 7.1 Hz, 1H), 6.60 (s, 1H), 4.39 (hept, *J* = 6.9 Hz, 1H), 1.54 (d, *J* = 6.9 Hz, 3H), 1.46 (d, *J* = 7.1 Hz, 3H).
^{13}C NMR (100 MHz, $CDCl_3$) δ 165.1, 137.4, 135.7, 135.5, 134.5, 132.0, 131.2, 130.1, 129.04, 128.96 (2C), 126.9 (2C), 123.9, 123.3, 121.9, 121.3, 120.7, 120.0, 112.5, 103.1, 48.3, 21.8, 21.6.
IR (FT-ATR, cm^{-1}, $CHCl_3$) ν_{max} 3969, 3926, 3907, 3842, 3822, 3803, 3757, 3718, 3676, 3653, 3629, 3595, 3578, 3545, 3390, 3217, 3059, 3012, 2978, 2935, 2877, 2762, 2700, 2657, 2603, 2511, 2445, 2403, 2364, 2326, 2252, 2206, 2140, 2087, 1959, 1921, 1894, 1805, 1674, 1608, 1581, 1516, 1439, 1408, 1342, 1304, 1250, 1219, 1176, 1130, 1103, 1076, 1026, 949, 926, 895, 845, 791, 744, 706, 667.
HRMS (EI) *m*/*z*: $[M]^+$ Calcd for $C_{24}H_{22}N_2O$ 354.1732; found 354.1733.

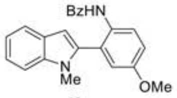

4b

(^{1}H NMR, 400 MHz, $CDCl_3$)

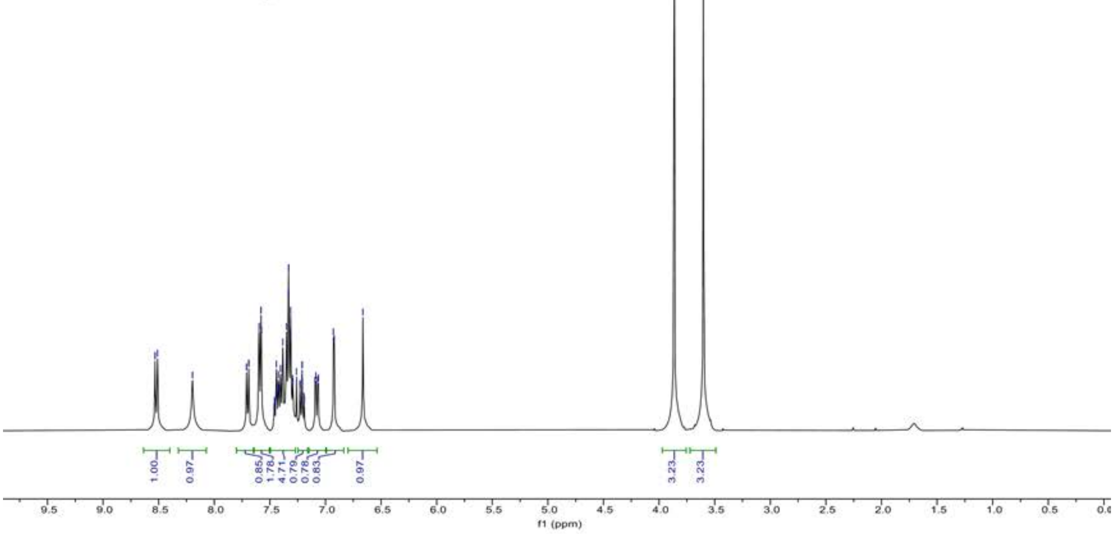

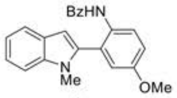

4b

(^{13}C NMR, 100 MHz, $CDCl_3$)

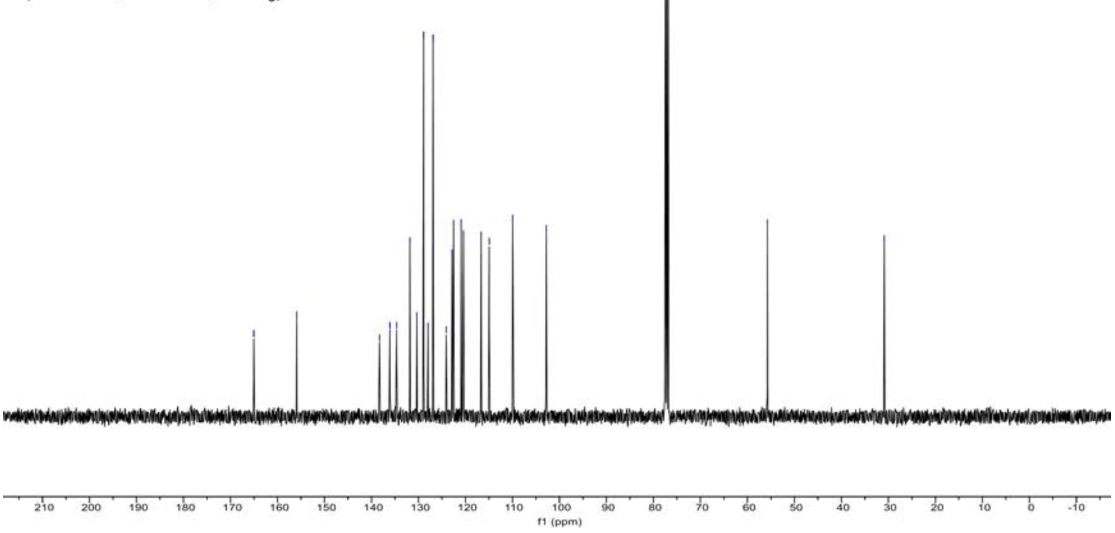

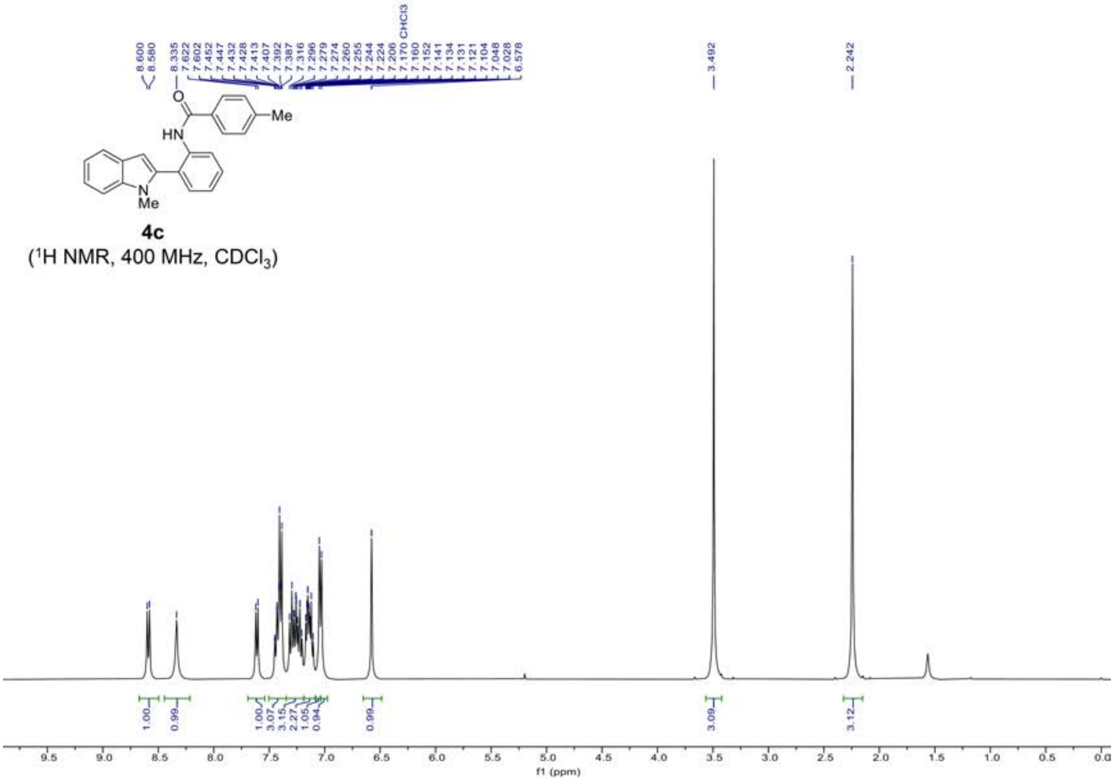
4c
(¹H NMR, 400 MHz, CDCl3)
f1 (ppm)

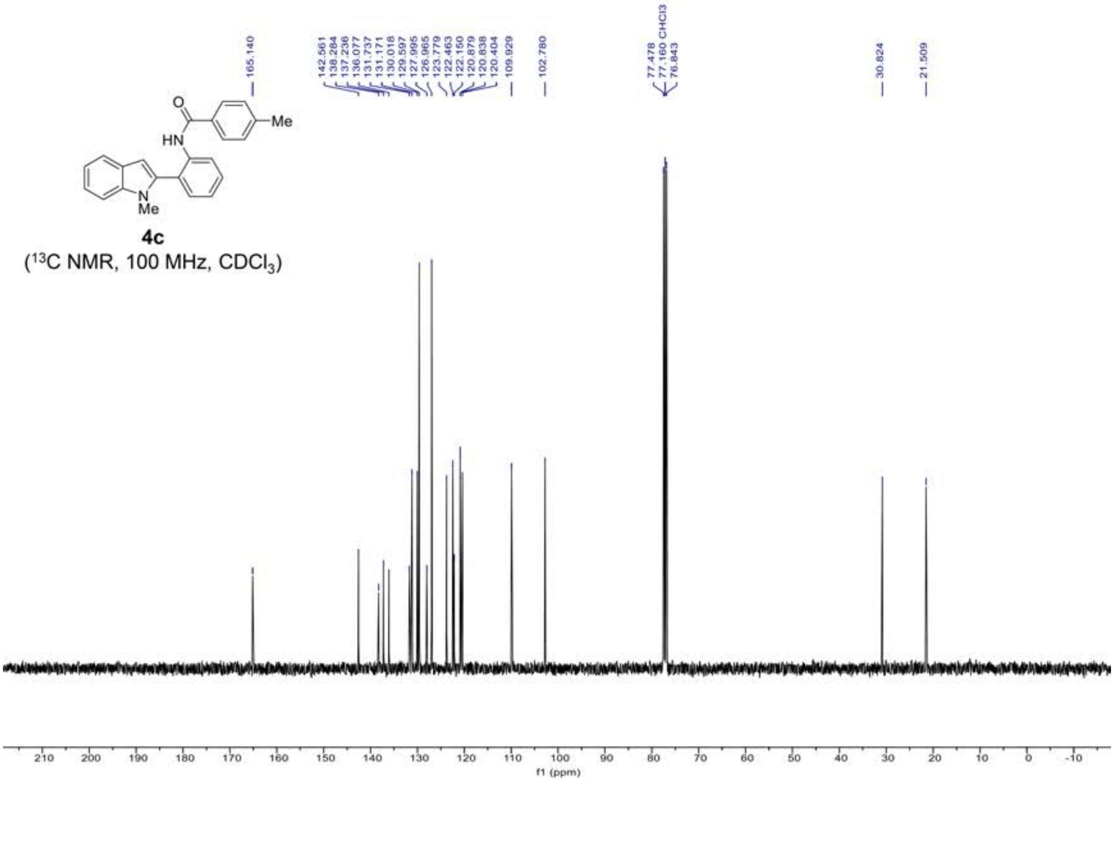
4c
(¹³C NMR, 100 MHz, CDCl3)
f1 (ppm)

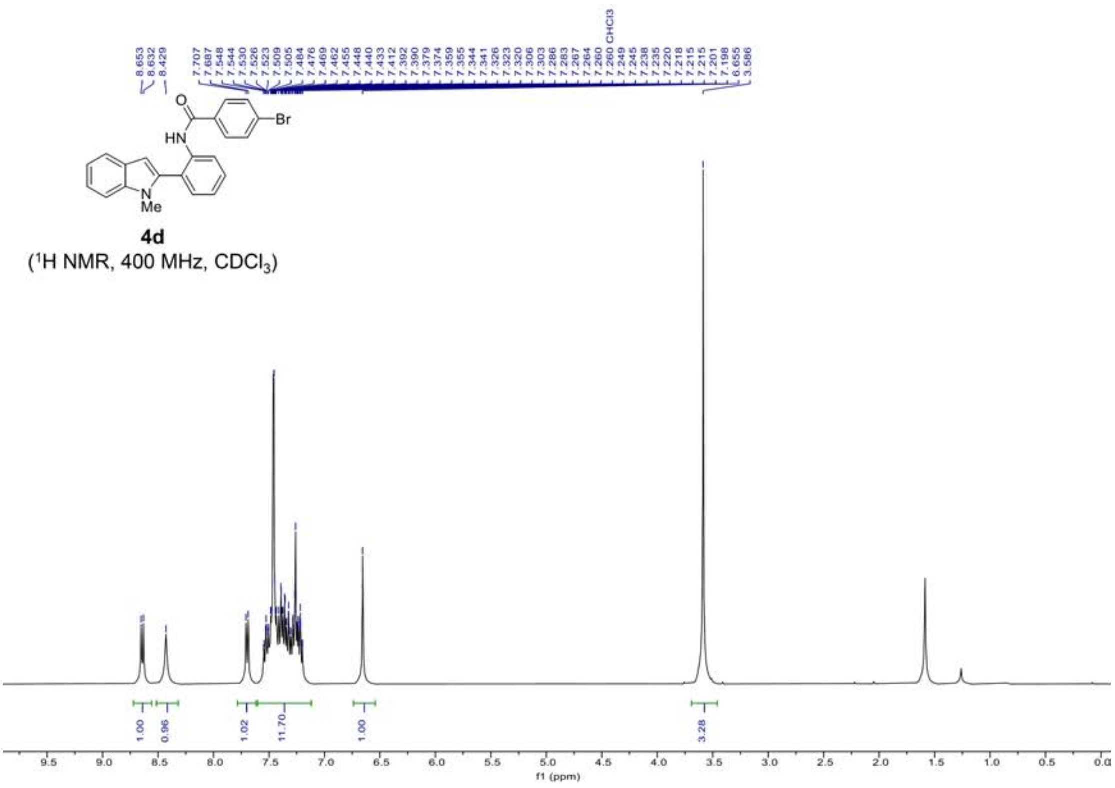

4d
(^{1}H NMR, 400 MHz, $CDCl_3$)

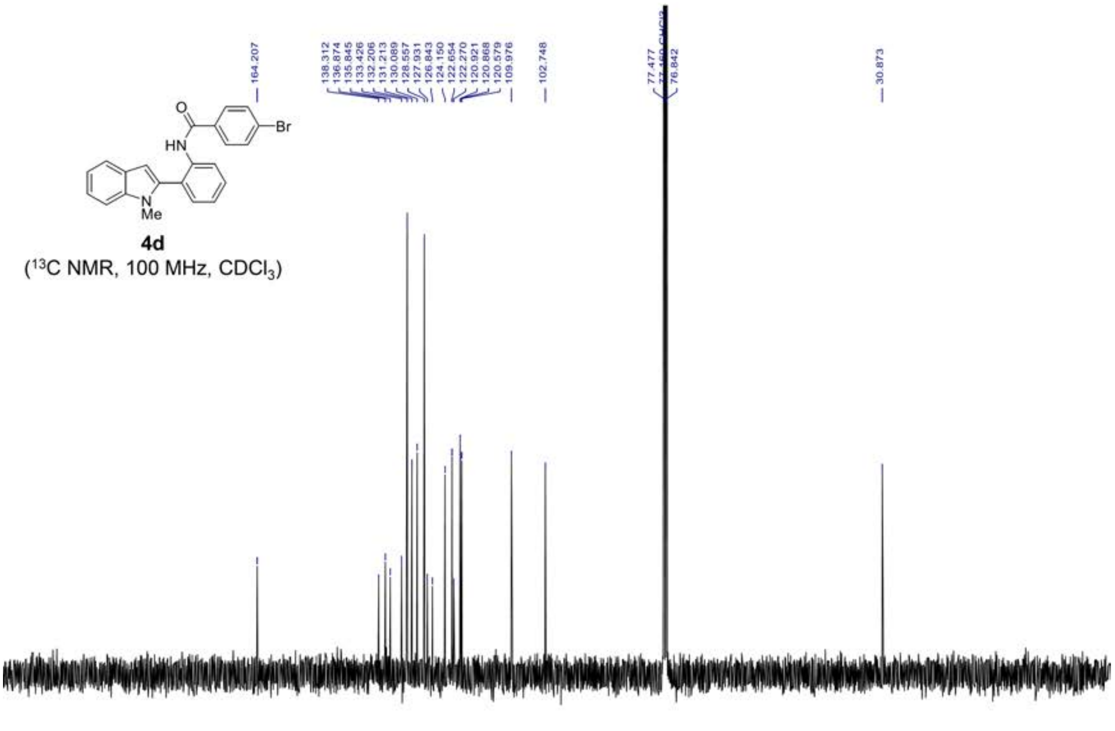

4d
(^{13}C NMR, 100 MHz, $CDCl_3$)

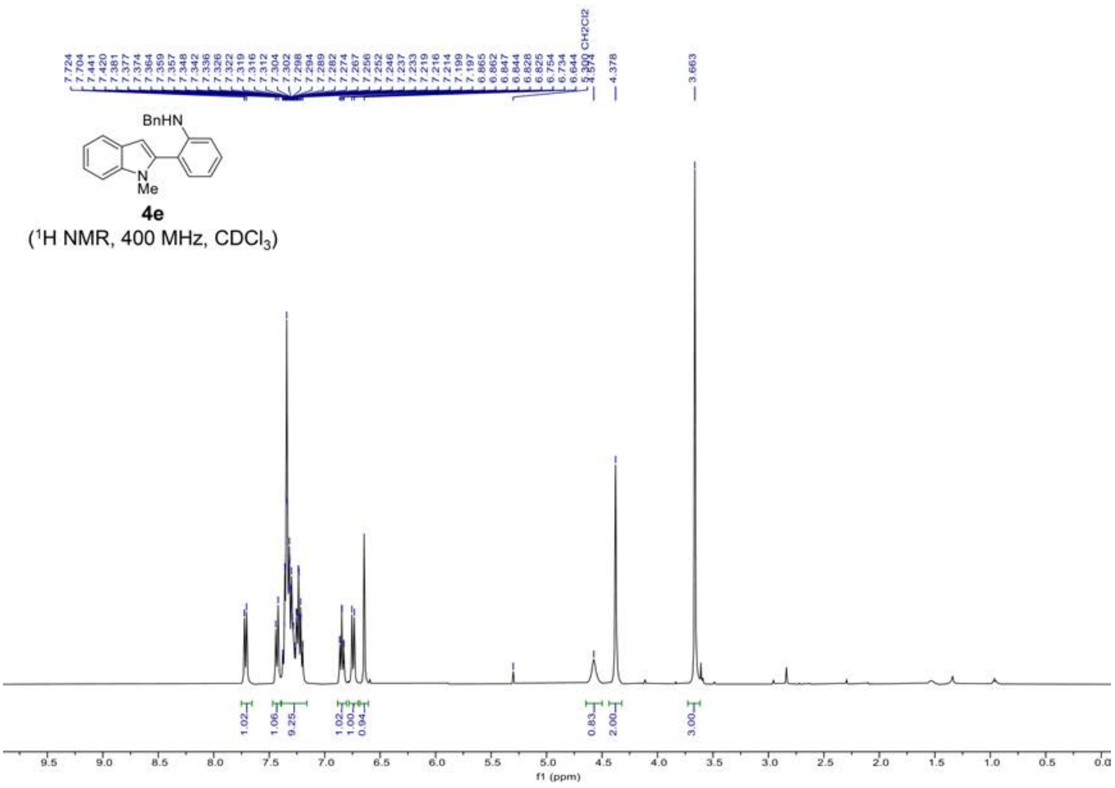
BnHN
N
Me
4e
(1H NMR, 400 MHz, CDCl3)
f1 (ppm)

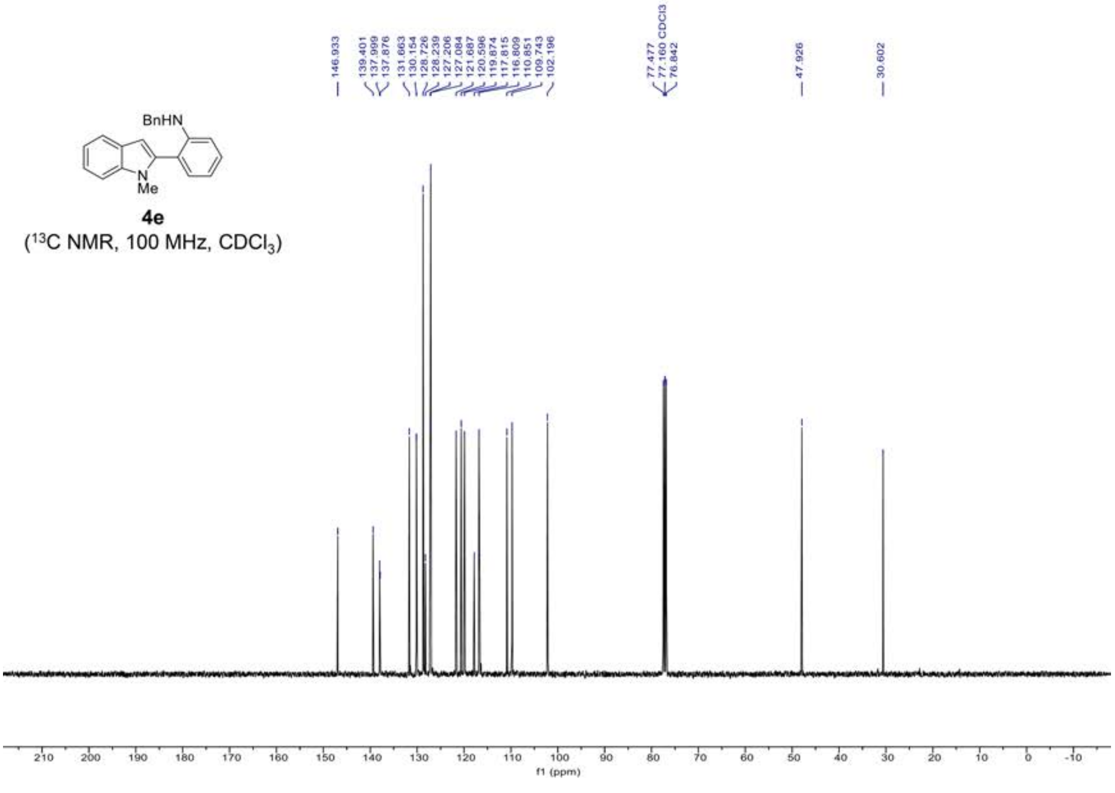
BnHN
N
Me
4e
(13C NMR, 100 MHz, CDCl3)
f1 (ppm)

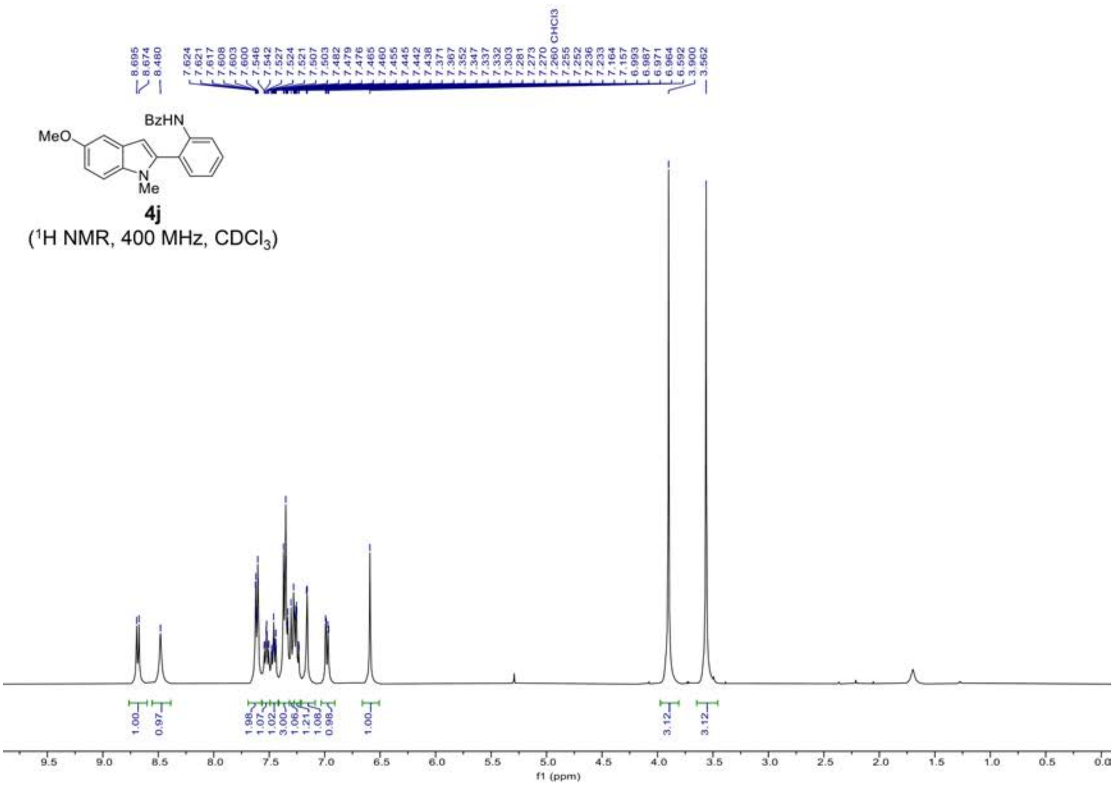
MeO
BzHN
N
Me
4j
(^{1}H NMR, 400 MHz, $CDCl_3$)
f1 (ppm)

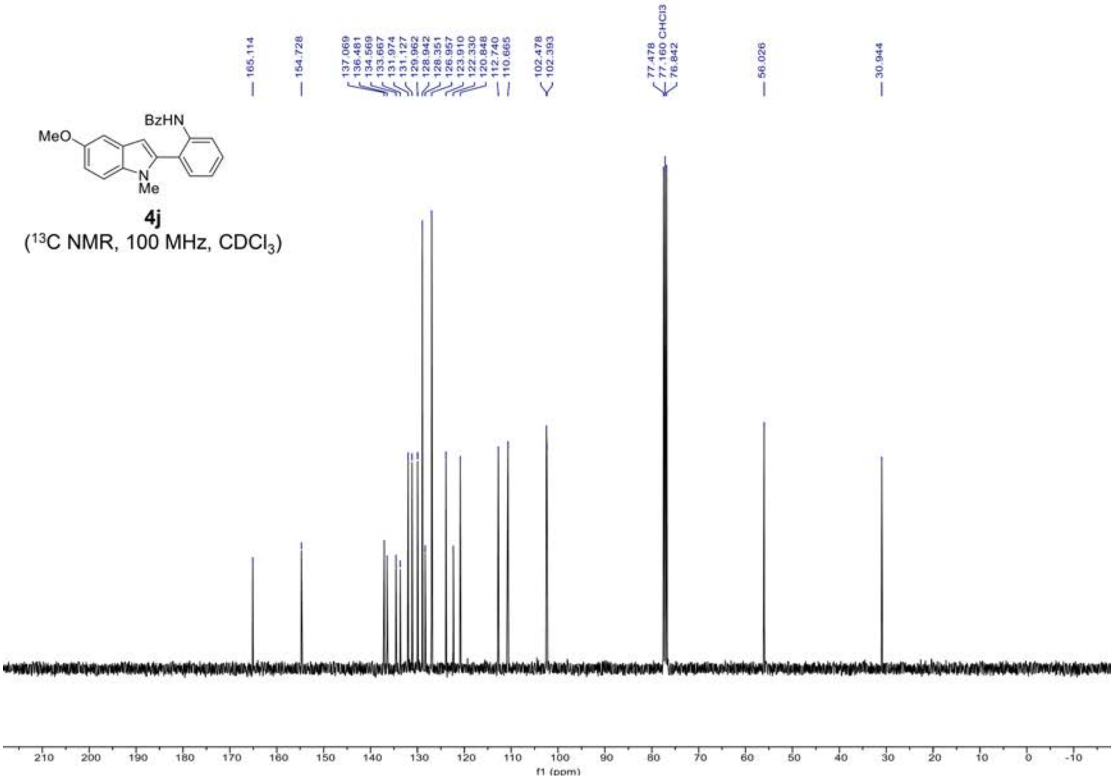
MeO
BzHN
N
Me
4j
(^{13}C NMR, 100 MHz, $CDCl_3$)
f1 (ppm)

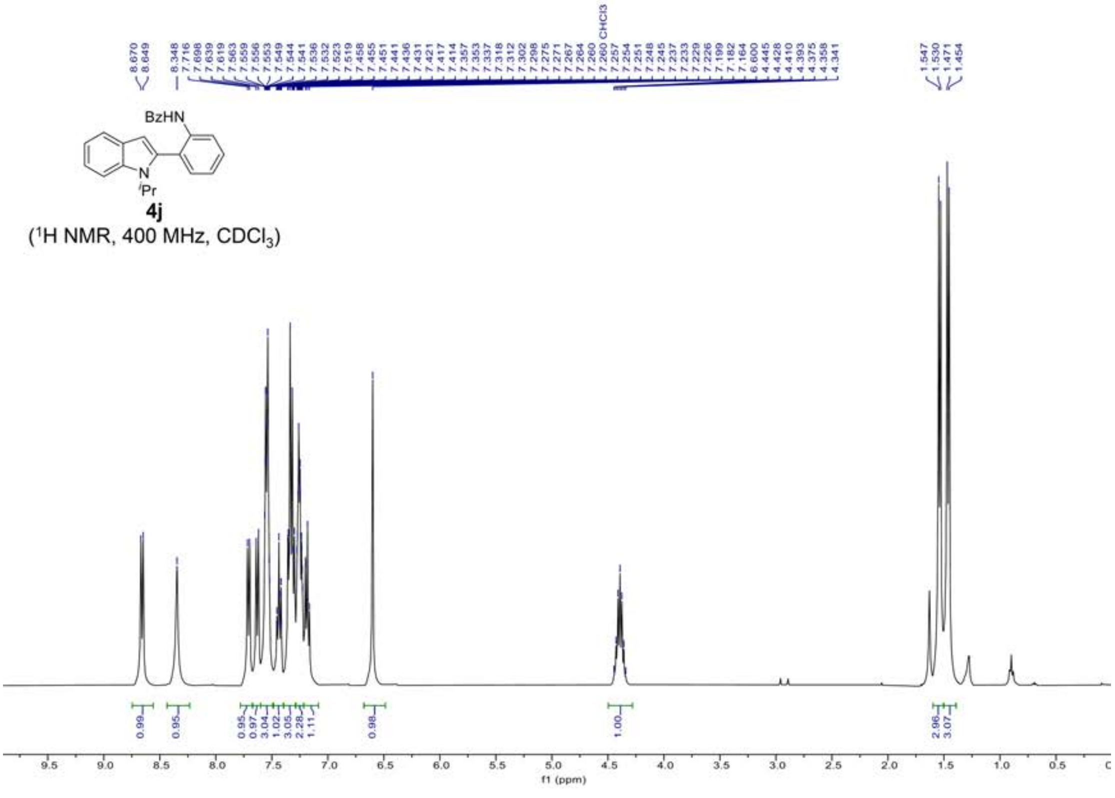
8.670
8.649
8.348
7.716
7.698
7.639
7.619
7.563
7.559
7.556
7.553
7.549
7.544
7.541
7.536
7.532
7.523
7.519
7.458
7.455
7.451
7.441
7.436
7.421
7.417
7.414
7.357
7.353
7.337
7.318
7.312
7.302
7.298
7.275
7.271
7.267
7.264
7.260
7.260 CHCl3
7.257
7.254
7.251
7.248
7.245
7.237
7.233
7.229
7.226
7.199
7.182
7.164
6.600
4.445
4.428
4.410
4.393
4.375
4.358
4.341
1.547
1.530
1.471
1.454
BzHN
N
iPr
4j
(1H NMR, 400 MHz, CDCl3)
0.99
0.95
0.95
0.97
3.04
1.02
3.05
2.28
1.11
0.98
1.00
2.96
3.07
f1 (ppm)

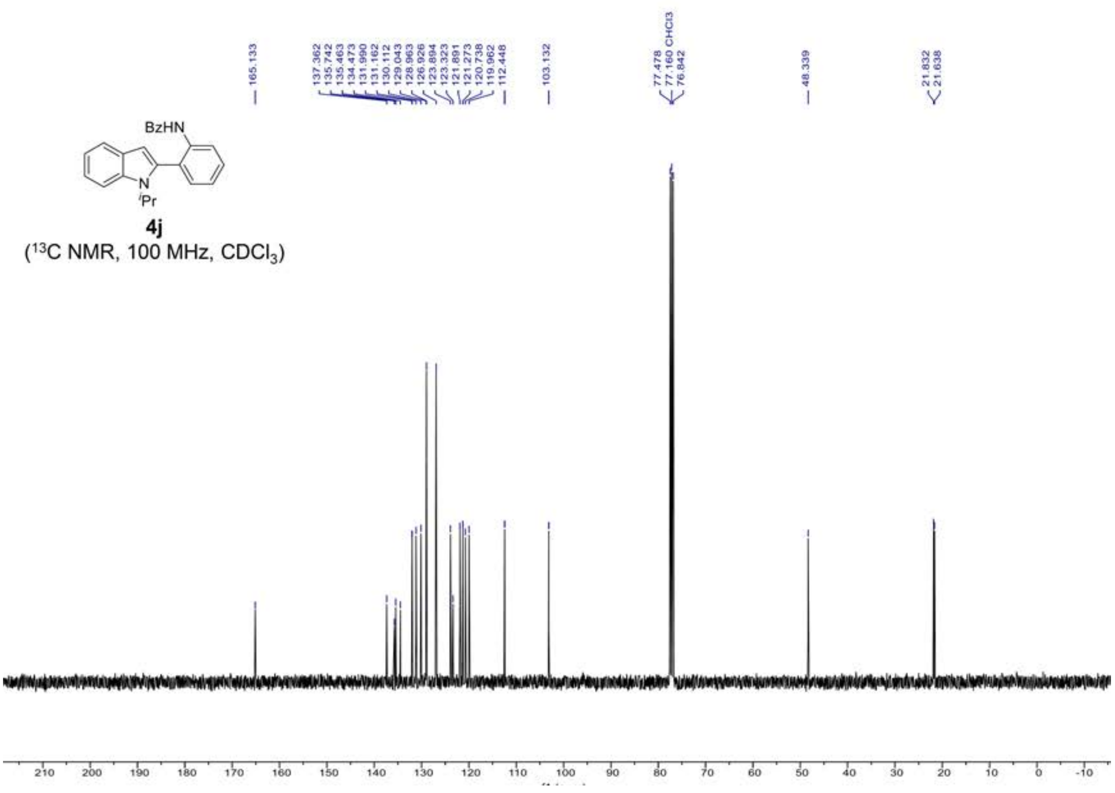
165.133
137.362
135.742
135.463
134.473
131.990
131.162
130.112
129.043
128.963
126.926
123.894
123.323
121.891
121.273
120.738
119.962
112.448
103.132
77.478 CHCl3
77.160
76.842
48.339
21.832
21.638
BzHN
N
iPr
4j
(13C NMR, 100 MHz, CDCl3)

4 Synthesis of 6

4.1 Synthesis of Substituted 3-Arylindole Intermediates (S28–S38)

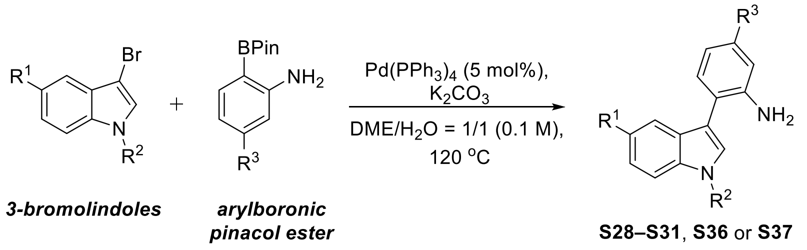

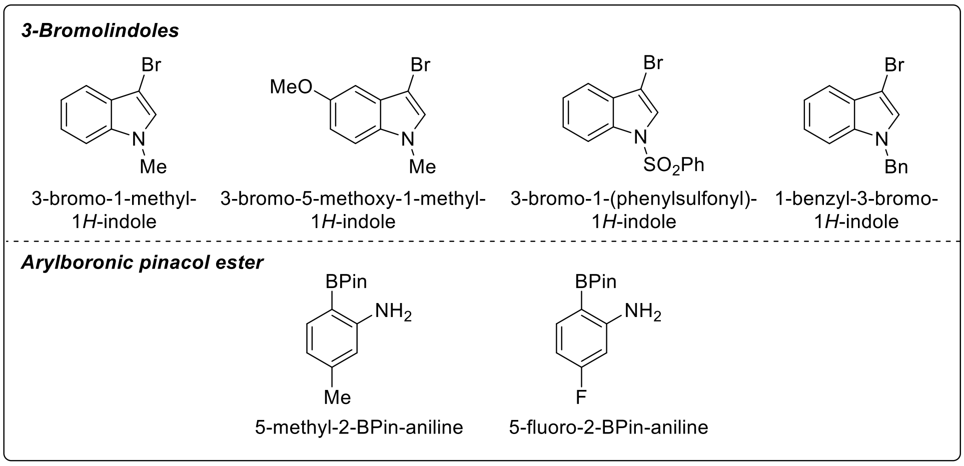

Procedure 15: An oven-dried round-bottom flask equipped with a magnetic stir bar, a solution of 3-bromolindoles (1.0 equiv) and arylboronic pinacol ester (1.0 equiv) in 1,2-dimethoxyethane mixed with water (DME/water = 1/1, 0.10 M) was added. Then, $Pd(PPh_3)_4$ (5 mol%) and K_2CO_3 (4.0 equiv) were added to flask. The reaction mixture was heated for overnight at 120 ºC in a heating block. The progress of the reaction was monitored by TLC. The reaction mixture was cooled to rt, diluted with CH_2Cl_2 and washed with brine. The organic layer was extracted with CH_2Cl_2 three times, dried with anhydrous $MgSO_4$, filtered, and concentrated *in vacuo*. The crude material was then purified by flash chromatography to afford the desired material **S28**–**S31**, **S36** or **S37**.

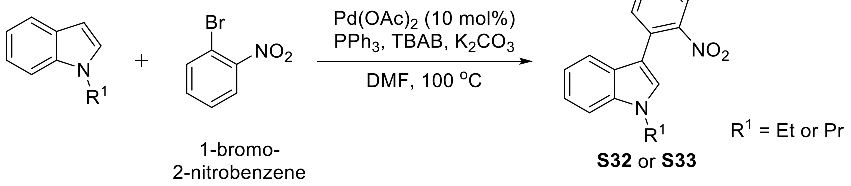

Procedure 16: An oven-dried round-bottom flask equipped with a magnetic stir bar, *N*-alkylated indoles (1.0 equiv), 2-bromo-1-nitrobenzene (1.2 equiv), $Pd(OAc)_2$ (10 mol%), PPh_3 (10 mol%), K_2CO_3 (3.0 equiv) and tetrabutylammonium bromide (TBAB, 20 mol%) were added and subsequently dissolved in DMF (0.1 M). The reaction mixture was heated for overnight at 110 ºC in an oil bath. The progress of the reaction was monitored by TLC. The reaction was cooled to rt, diluted with EtOAc and washed with brine. The organic layer was extracted with EtOAc three times, dried with

anhydrous $MgSO_4$, filtered, and concentrated *in vacuo*. The crude material was then purified by flash chromatography to afford the desired material **S32** or **S33**.

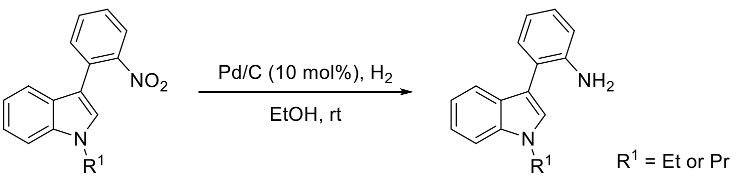

S32 or **S33** **S34** or **S35**

Procedure 17: To an oven-dried round-bottom flask equipped with a magnetic stir bar, a solution of **S32** or **S33** (1.0 equiv) in EtOH (0.5 M) was added. Then, subsequently added Pd/C (10 mol%). The reaction mixture was allowed to stir at rt with hydrogen pressure using hydrogen balloon. The progress of the reaction was monitored by TLC. The reaction mixture was filtered over celite, rinsed with CH_2Cl_2 and concentrated *in vacuo*. The crude material was then purified by flash chromatography to afford the desired material **S34** or **S35**.

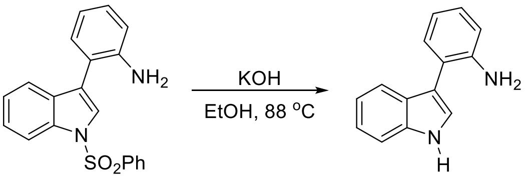

S37 **S38**

Procedure 18: To a reaction tube equipped with a magnetic stirring bar, **S37** (1.0 equiv) and KOH (12.0 equiv) were added and subsequently dissolved in EtOH (0.25M). The reaction was heated to 85 °C and allowed to stir for overnight. The progress of the reaction was monitored by TLC. The reaction mixture was cooled to rt, diluted with CH_2Cl_2 and washed with brine. The organic layer was extracted with CH_2Cl_2 three times, dried with anhydrous $MgSO_4$, filtered, and concentrated *in vacuo*. The crude material was then purified by flash chromatography to afford the desired material **S38**.

Characterization and Spectra of 3-Arylindole Intermediates (S28–S38)

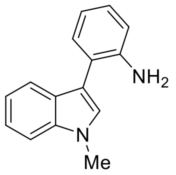

2-(1-Methyl-1*H*-indol-3-yl)aniline (S28) was synthesized by following Procedure 15. The crude material was purified by normal-phase column chromatography using an eluent of 25% EtOAc/Hx to provide **S28** (482 mg, 52%).

^{1}H NMR (400 MHz, $CDCl_3$) δ 7.66 (dt, *J* = 7.9, 1.0 Hz, 1H), 7.41 (dt, *J* = 8.3, 1.0 Hz, 1H), 7.32 (dddd, *J* = 9.4, 6.9, 4.0, 1.8 Hz, 2H), 7.22–7.13 (m, 3H), 6.90–6.82 (m, 2H), 4.00–3.60 (m, 5H).
^{13}C NMR (100 MHz, $CDCl_3$) δ 144.8, 137.1, 131.3, 127.9, 127.7, 127.0, 122.1, 120.7, 120.5, 119.7, 118.4, 115.4, 113.3, 109.6, 33.0.

The spectral data were identical with those previously reported.[18]

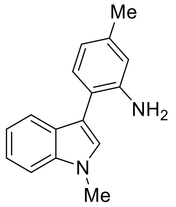

5-Methyl-2-(1-methyl-1*H*-indol-3-yl)aniline (S29) was synthesized by following Procedure 15. The crude material was purified by normal-phase column chromatography using an eluent of 25% EtOAc/Hx to provide **S29** (237 mg, 40%).

^{1}H NMR (400 MHz, $CDCl_3$) δ 7.61 (d, *J* = 8.0 Hz, 1H), 7.45–7.31 (m, 1H), 7.26 (ddd, *J* = 8.2, 6.9, 1.2 Hz, 1H), 7.23–7.02 (m, 3H), 6.75–6.54 (m, 2H), 3.81 (s, 3H), 3.59 (brs, 2H), 2.32 (s, 3H).
^{13}C NMR (100 MHz, $CDCl_3$) δ 144.6, 137.7, 137.1, 131.2, 127.7, 127.1, 122.0, 120.6, 119.6, 119.4, 117.9, 116.2, 113.3, 109.5, 32.9, 21.4.
IR (FT-ATR, cm^{-1}, $CHCl_3$) ν_{max} 3980, 3903, 3861, 3811, 3784, 3757, 3656, 3633, 3460, 3367, 3205, 3047, 3008, 2916, 2827, 2731, 2696, 2603, 2565, 2480, 2403, 2376, 2353, 2314, 2245, 2171, 2021, 1990, 1917, 1882, 1801, 1689, 1608, 1551, 1466, 1423, 1373, 1327, 1230, 1257, 1219, 1157, 1134, 1088, 1038, 1011, 953, 922, 860, 802, 737, 663.
HRMS (EI) *m*/*z*: $[M]^+$ Calcd for $C_{16}H_{16}N_2$ 236.1314; found 236.1313.

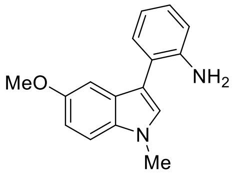

2-(5-Methoxy-1-methyl-1*H*-indol-3-yl)aniline (S30) was synthesized by following Procedure 15. The crude material was purified by normal-phase column chromatography using an eluent of 20% EtOAc/Hx to provide **S30** (310 mg, 60%).

^{1}H NMR (400 MHz, $CDCl_3$) δ 7.66 (dt, *J* = 8.0, 1.0 Hz, 1H), 7.41 (dt, *J* = 8.2, 0.9 Hz, 1H), 7.31 (ddd, *J* = 8.2, 6.9, 1.2 Hz, 1H), 7.25–7.11 (m, 3H), 6.70 (d, *J* = 7.7 Hz, 2H), 3.86 (s, 3H), 2.37 (s, 3H).
^{13}C NMR (100 MHz, $CDCl_3$) δ 144.6, 137.7, 137.1, 131.2, 127.7, 127.1, 122.0, 120.6, 119.6, 119.4, 117.9, 116.2, 113.3, 109.5, 32.9, 21.4.

The spectral data were identical with those previously reported.[18]

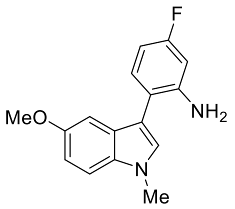

5-Fluoro-2-(5-methoxy-1-methyl-1*H*-indol-3-yl)aniline (S31) was synthesized by following Procedure 15. The crude material was purified by normal-phase column chromatography using an eluent of 9% EtOAc/Hx to provide **S31** (76.3 mg, 14%).

^{1}H NMR (400 MHz, $CDCl_3$) δ 7.28 (d, *J* = 8.9 Hz, 1H), 7.23–7.16 (m, 1H), 7.10 (s, 1H), 6.99 (d, *J* = 2.4 Hz, 1H), 6.95 (dd, *J* = 8.8, 2.5 Hz, 1H), 6.60–6.43 (m, 2H), 3.94 (brs, 2H), 3.82 (d, *J* = 2.4 Hz, 6H).
^{13}C NMR (100 MHz, $CDCl_3$) δ 163.0 (d, *J* = 241.0 Hz, 1C), 154.4, 146.4 (d, *J* = 11.0 Hz, 1C), 132.5, 132.3 (d, *J* = 10.0 Hz, 1C), 128.3, 127.3, 116.5 (d, *J* = 3.0 Hz, 1C), 112.7, 111.9, 110.5, 104.8 (d, *J* = 21.0 Hz, 1C), 101.9 (d, *J* = 24.0 Hz, 1C), 101.7, 56.1, 33.1.
IR (FT-ATR, cm^{-1}, $CHCl_3$) ν_{max} 3965, 3930, 3907, 3880, 3865, 3841, 3807, 3980, 3757, 3737, 3718, 3680, 3656, 3618, 3595, 3572, 3471, 3375, 3201, 3066, 3005, 3939, 2831, 2738, 2665, 2592, 2414, 2372, 2341, 2318, 2171, 2113, 2052, 1944, 1859, 1736, 1612, 1570, 1547, 1485, 1454, 1427, 1377, 1342, 1281, 1261, 1211, 1161, 1130, 1088, 1026, 976, 953, 841, 791, 748, 714, 671.
HRMS (EI) *m*/*z*: $[M]^+$ Calcd for $C_{16}H_{15}FN_2O$ 270.1168; found 270.1164.

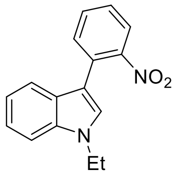

1-Ethyl-3-(2-nitrophenyl)-1*H*-indole (S32) was synthesized by following Procedure 16. The crude material was purified by normal-phase column chromatography using an eluent of 13% EtOAc/Hx to provide **S32** (783 mg, 59%).

^{1}H NMR (400 MHz, $CDCl_3$) δ 7.85 (dd, *J* = 8.1, 1.4 Hz, 1H), 7.70 (dd, *J* = 7.8, 1.5 Hz, 1H), 7.65–7.57 (m, 2H), 7.49–7.38 (m, 2H), 7.37–7.28 (m, 2H), 7.21 (ddd, *J* = 8.1, 7.0, 1.1 Hz, 1H), 4.23 (q, *J* = 7.3 Hz, 2H), 1.53 (t, *J* = 7.3 Hz, 3H).
^{13}C NMR (100 MHz, $CDCl_3$) δ 149.7, 136.0, 132.5, 131.9, 129.3, 126.8, 126.4, 124.1, 122.3, 120.3, 119.1, 110.9, 109.9, 102.7, 41.2, 15.4.

The spectral data were identical with those previously reported.[18]

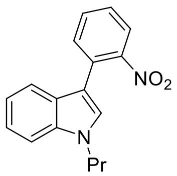

1-Propyl-3-(2-nitrophenyl)-1*H*-indole (S33) was synthesized by following Procedure 16. The crude material was purified by normal-phase column chromatography using an eluent of 13% EtOAc/Hx to provide **S33** (819 mg, 58%).

^{1}H NMR (400 MHz, $CDCl_3$) δ 7.82 (dd, *J* = 8.1, 1.4 Hz, 1H), 7.68 (dd, *J* = 7.9, 1.4 Hz, 1H), 7.60 (td, *J* = 7.6, 1.3 Hz, 1H), 7.55 (dt, *J* = 8.0, 1.0 Hz, 1H), 7.44–7.38 (m, 2H), 7.31–7.24 (m, 2H), 7.16 (ddd, *J* = 8.0, 7.0, 1.0 Hz, 1H), 4.14 (t, *J* = 7.1 Hz, 2H), 1.92 (h, *J* = 7.3 Hz, 2H), 0.97 (t, *J* = 7.4 Hz, 3H).
^{13}C NMR (100 MHz, $CDCl_3$) δ 149.9, 136.3, 132.5, 131.9, 129.3, 127.3, 126.9, 126.9, 124.1, 122.3, 120.4, 119.2, 110.7, 110.1, 48.4, 23.5, 11.6.

The spectral data were identical with those previously reported.[18]

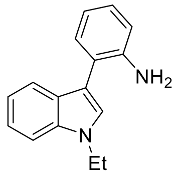

2-(1-Ethyl-1*H*-indol-3-yl)aniline (S34) was synthesized by following Procedure 17. The crude material was purified by normal-phase column chromatography using an eluent of 25% EtOAc/Hx to provide **S34** (493 mg, 79%).

^{1}H NMR (400 MHz, $CDCl_3$) δ 7.83 (dt, *J* = 7.9, 1.0 Hz, 1H), 7.55 (dt, *J* = 8.2, 1.0 Hz, 1H), 7.50 (dd, *J* = 7.5, 1.6 Hz, 1H), 7.43 (ddd, *J* = 8.2, 7.0, 1.2 Hz, 1H), 7.37 (s, 1H), 7.32 (ddd, *J* = 8.8, 7.4, 1.5 Hz, 2H), 7.01 (td, *J* = 7.4, 1.2 Hz, 1H), 6.92 (dd, *J* = 8.0, 1.3 Hz, 1H), 4.29 (q, *J* = 7.3 Hz, 2H), 3.95 (s, 2H), 1.61 (t, *J* = 7.3 Hz, 3H).

^{13}C NMR (100 MHz, $CDCl_3$) δ 144.7, 136.0, 131.1, 127.7, 127.0, 125.8, 121.8, 120.6, 120.5, 119.5, 118.2, 115.3, 113.2, 109.5, 40.9, 15.4.

The spectral data were identical with those previously reported.[18]

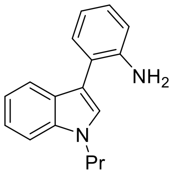

2-(1-Propyl-1*H*-indol-3-yl)aniline (S35) was synthesized by following Procedure 17. The crude material was purified by normal-phase column chromatography using an eluent of 25% EtOAc/Hx to provide **S35** (272 mg, 76%).

^{1}H NMR (400 MHz, $CDCl_3$) δ 7.72 (dt, *J* = 8.0, 1.0 Hz, 1H), 7.47 (dt, *J* = 8.3, 1.0 Hz, 1H), 7.40 (dd, *J* = 7.5, 1.6 Hz, 1H), 7.34 (ddd, *J* = 8.3, 7.0, 1.2 Hz, 1H), 7.30 (s, 1H), 7.26–7.18 (m, 2H), 6.92 (td, *J* = 7.4, 1.2 Hz, 1H), 6.87 (dd, *J* = 7.9, 1.2 Hz, 1H), 4.18 (t, *J* = 7.1 Hz, 2H), 3.90 (s, 2H), 1.97 (h, *J* = 7.3 Hz, 2H), 1.04 (t, *J* = 7.4 Hz, 3H).
^{13}C NMR (100 MHz, $CDCl_3$) δ 144.7, 136.4, 131.2, 127.7, 127.0, 126.8, 121.9, 120.7, 120.6, 119.5, 118.3, 115.4, 113.1, 109.7, 48.1, 23.6, 11.7.

The spectral data were identical with those previously reported.[18]

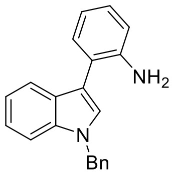

2-(1-Benzyl-1*H*-indol-3-yl)aniline (S36) was synthesized by following Procedure 15. The crude material was purified by normal-phase column chromatography using an eluent of 13% EtOAc/Hx to provide **S36** (341 mg, 38%).

^{1}H NMR (400 MHz, $CDCl_3$) δ 7.71 (d, *J* = 8.0 Hz, 1H), 7.46–7.12 (m, 11H), 6.94–6.68 (m, 2H), 5.38 (s, 2H), 3.82 (brs, 2H).
^{13}C NMR (100 MHz, $CDCl_3$) δ 144.7, 137.4, 136.8, 131.3, 129.4, 128.9 (2C), 127.94, 127.85, 127.1 (2C), 122.3, 120.7, 120.6, 119.9, 118.5, 115.5, 115.3, 114.0, 110.1, 50.3.
IR (FT-ATR, cm^{-1}, $CHCl_3$) ν_{max} 3926, 3865, 3838, 3803, 3734, 3714, 3672, 3652, 3568, 3467, 3375, 3194, 3055, 3012, 2924, 2866, 2808, 2719, 2607, 2557, 2403, 2322, 2114, 1925, 1890, 1801, 1770, 1682, 1612, 1547, 1520, 1493, 1454, 1354, 1335, 1296, 1254, 1215, 1173, 1076, 1022, 964, 937, 845, 813, 737, 694, 663.
HRMS (EI) *m*/*z*: $[M]^+$ Calcd for $C_{21}H_{18}N_2$ 298.1470; found 298.1471.

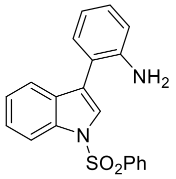

2-(1-(Phenylsulfonyl)-1*H*-indol-3-yl)aniline (S37) was synthesized by following Procedure 15. The crude material was purified by normal-phase column chromatography using an eluent of 33% EtOAc/Hx to provide **S37** (1.29 g, 93%).

^{1}H NMR (400 MHz, $CDCl_3$) δ 8.14 (dd, *J* = 8.4, 0.9 Hz, 1H), 7.98 (dd, *J* = 8.5, 1.3 Hz, 2H), 7.74 (s, 1H), 7.65–7.52 (m, 2H), 7.52–7.45 (m, 2H), 7.41 (ddd, *J* = 8.5, 7.2, 1.3 Hz, 1H), 7.35–7.13 (m, 3H), 6.96–6.69 (m, 2H), 3.76 (brs, 2H).
^{13}C NMR (100 MHz, $CDCl_3$) δ 144.7, 138.1, 135.2, 134.0, 131.0, 129.9, 129.4 (2C), 129.2, 126.9 (2C), 125.2, 124.2, 123.6, 121.3, 121.2, 118.4, 117.6, 115.7, 113.8.
IR (FT-ATR, cm^{-1}, $CHCl_3$) ν_{max} 3930, 3869, 3838, 3803, 3780, 3734, 3680, 3653, 3591, 3568, 3471, 3386, 3128, 3062, 3020, 2893, 2754, 2669, 2619, 2573, 2403, 2368, 2322, 2268, 2110, 1944, 1901, 1805, 1743, 1678, 1616, 1562, 1493, 1446, 1369, 1338, 1304, 1215, 1176, 1126, 1092, 1014, 933, 856, 810, 741, 683.
HRMS (EI) *m*/*z*: $[M]^+$ Calcd for $C_{20}H_{16}N_2O_2S$ 348.0933; found 348.0929.

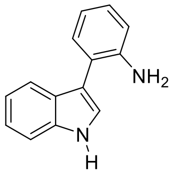

2-(1*H*-Indol-3-yl)aniline (S38) was synthesized by following Procedure 18. The crude material was purified by normal-phase column chromatography using an eluent of 33% EtOAc/Hx to provide **S38** (631 mg, 89%).

^{1}H NMR (400 MHz, $CDCl_3$) δ 8.31 (s, 1H), 7.78 (dd, *J* = 7.9, 1.1 Hz, 1H), 7.48 (dd, *J* = 7.5, 1.6 Hz, 1H), 7.41–7.26 (m, 4H), 7.17 (d, *J* = 2.5 Hz, 1H), 7.03 (td, *J* = 7.4, 1.3 Hz, 1H), 6.93 (dd, *J* = 7.9, 1.2 Hz, 1H), 3.91 (s, 2H).
^{13}C NMR (100 MHz, $CDCl_3$) δ 144.5, 136.1, 131.3, 128.0, 126.3, 123.2, 122.3, 120.7, 120.1, 120.0, 118.5, 115.6, 114.2, 111.5.

The spectral data were identical with those previously reported.[19]

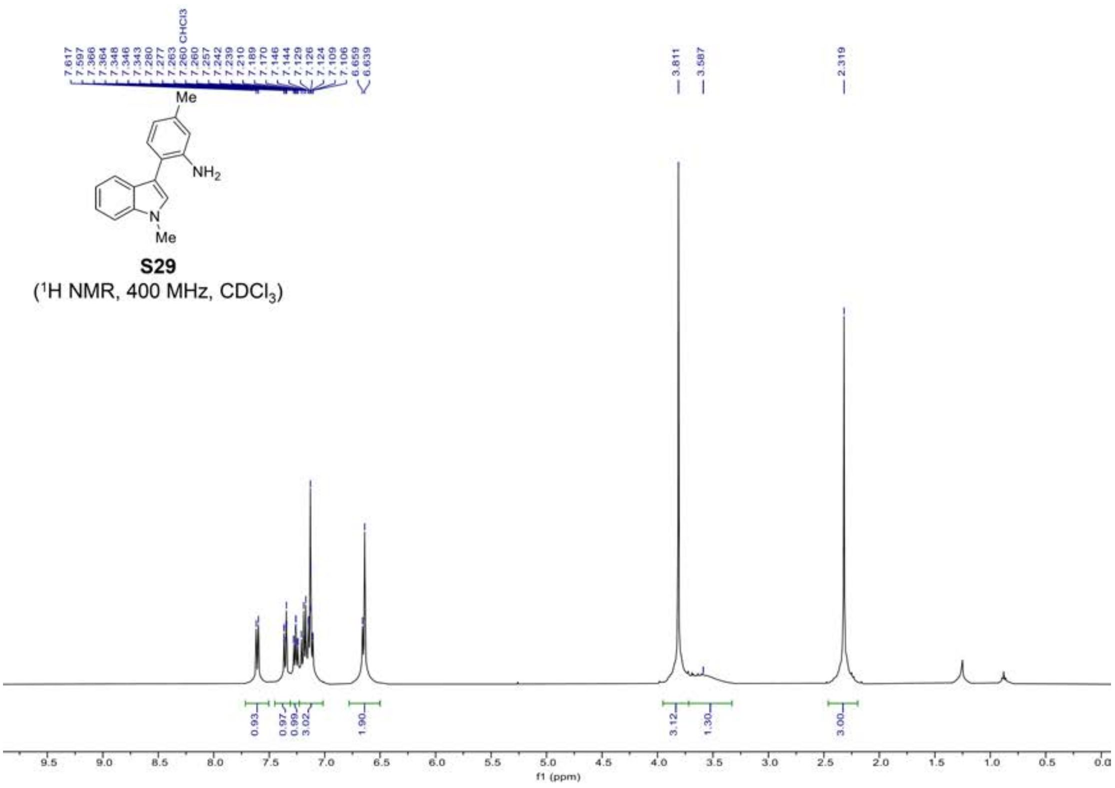

S29
(^{1}H NMR, 400 MHz, $CDCl_3$)

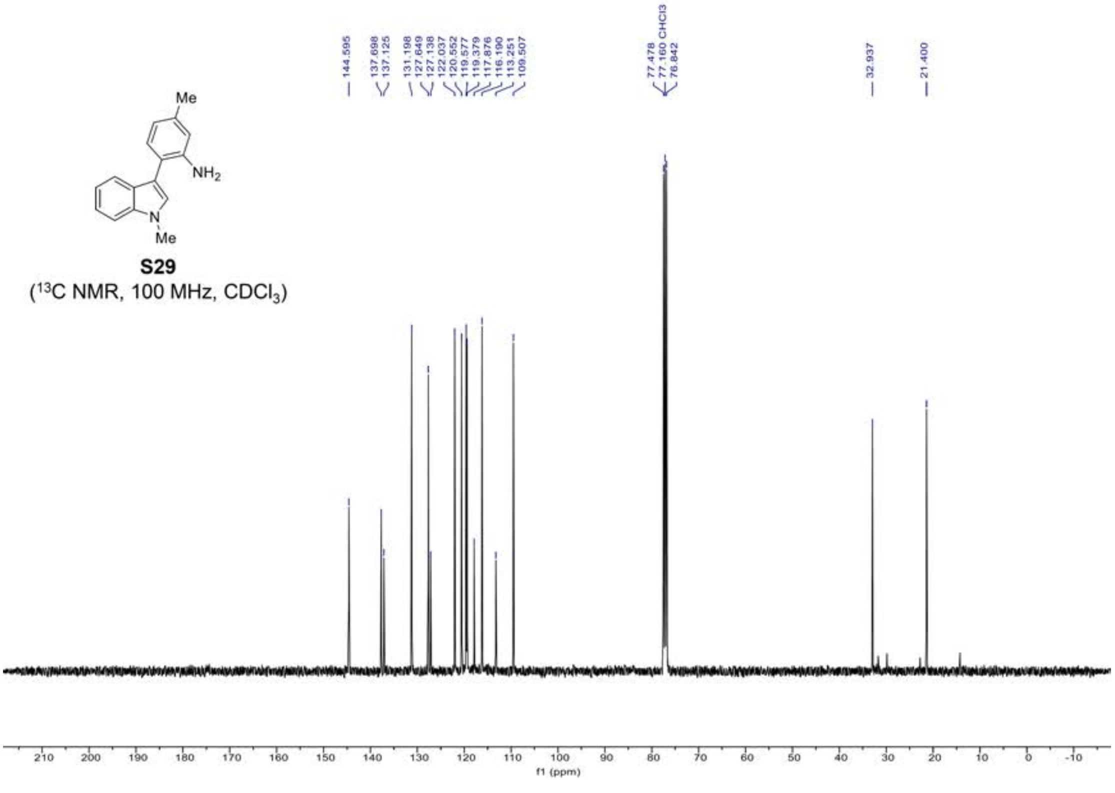

S29
(^{13}C NMR, 100 MHz, $CDCl_3$)

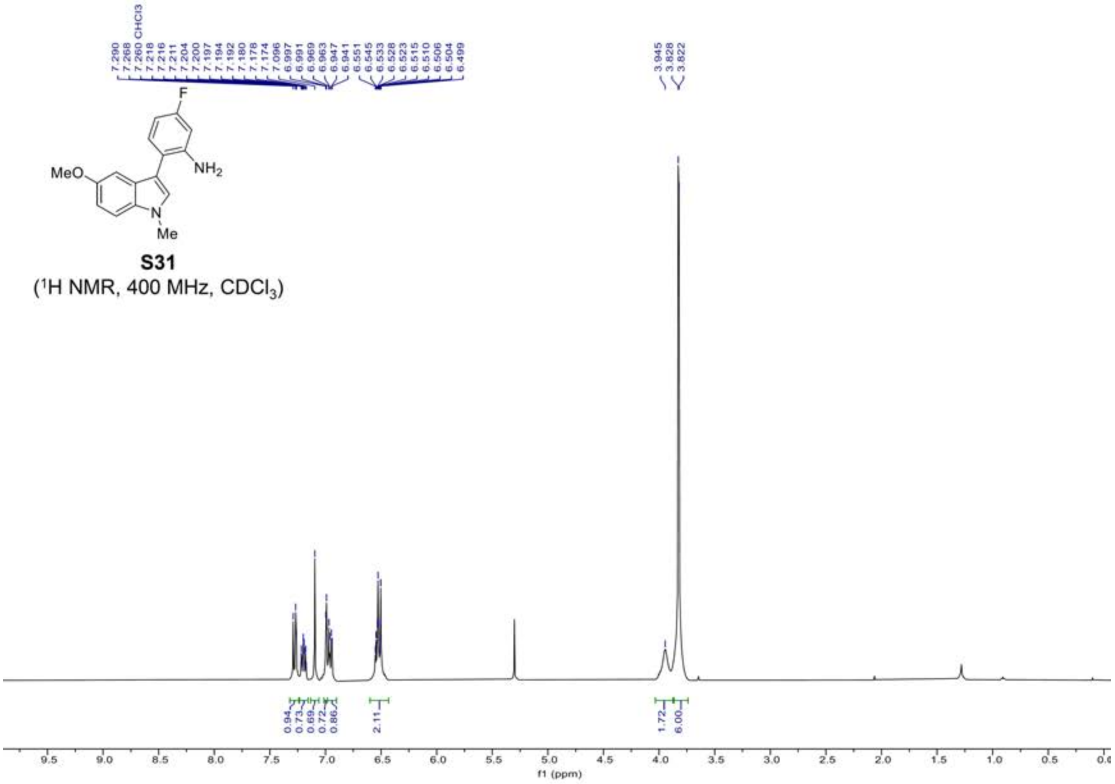
F
MeO
NH2
N
Me
S31
(1H NMR, 400 MHz, CDCl3)
7.290
7.268
7.260 CHCl3
7.218
7.216
7.211
7.204
7.200
7.197
7.194
7.192
7.180
7.178
7.174
7.096
6.997
6.991
6.969
6.963
6.947
6.941
6.551
6.545
6.533
6.528
6.523
6.515
6.510
6.506
6.504
6.499
3.945
3.828
3.822
0.94
0.73
0.69
0.72
0.86
2.11
1.72
6.00
f1 (ppm)

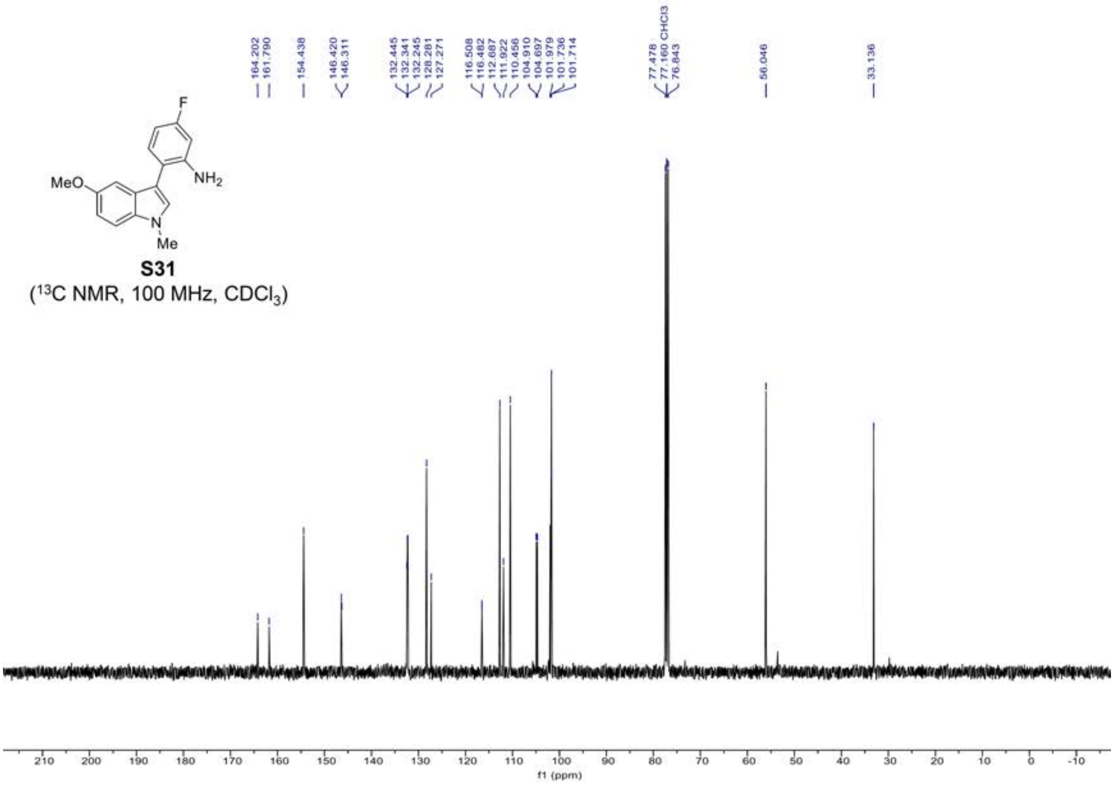
F
MeO
NH2
N
Me
S31
(13C NMR, 100 MHz, CDCl3)
164.202
161.790
154.438
146.420
146.311
132.445
132.341
132.245
128.281
127.271
116.508
116.482
112.687
111.922
110.456
104.910
104.697
101.979
101.736
101.714
77.478
77.160 CHCl3
76.843
56.046
33.136
f1 (ppm)

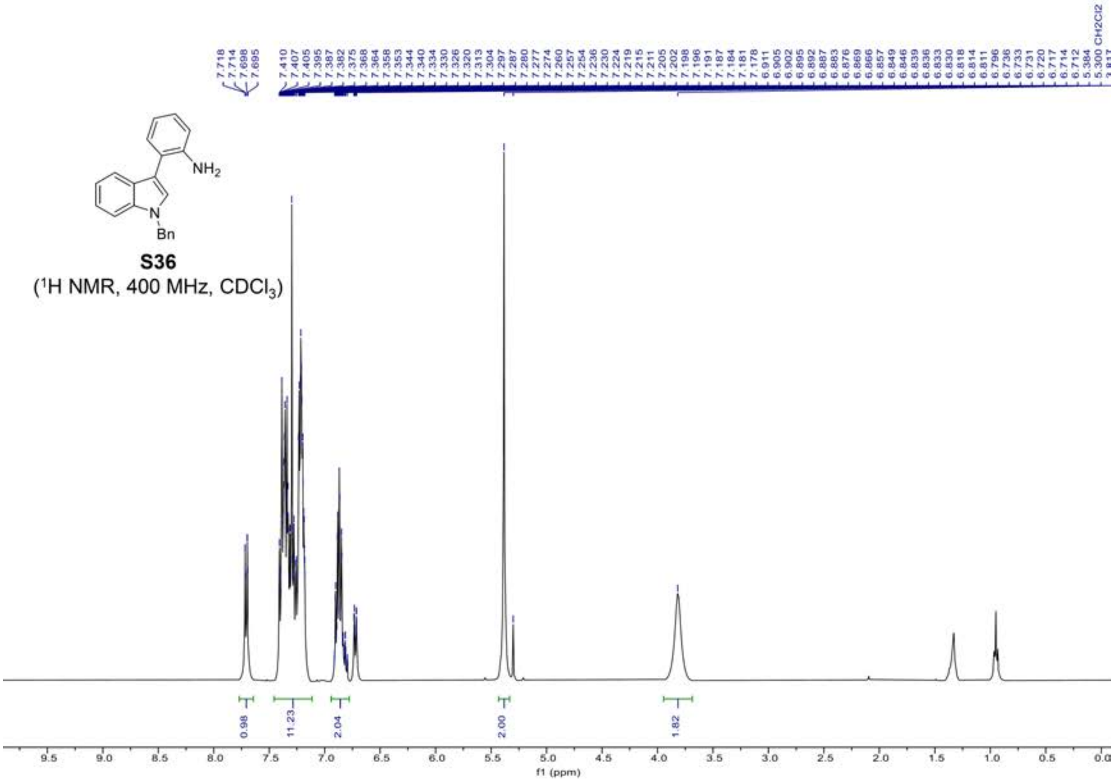

S36
(^{1}H NMR, 400 MHz, $CDCl_3$)

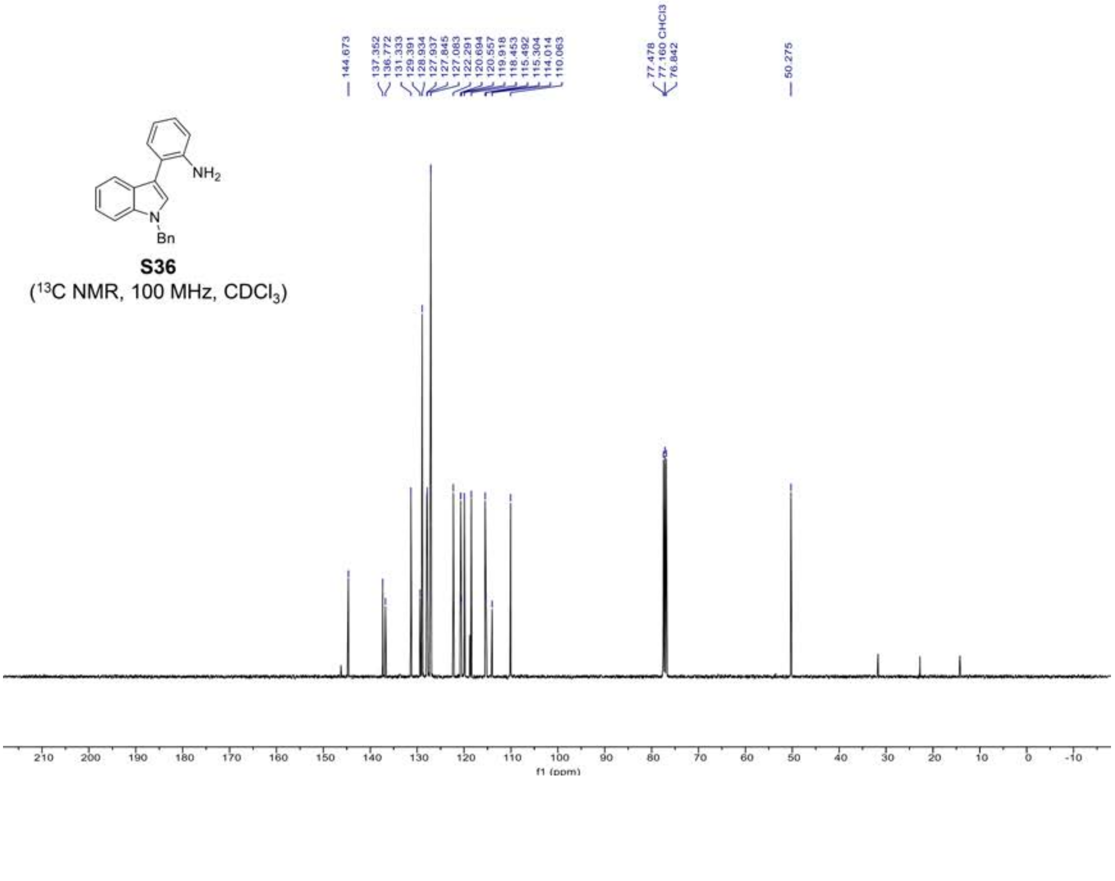

S36
(^{13}C NMR, 100 MHz, $CDCl_3$)

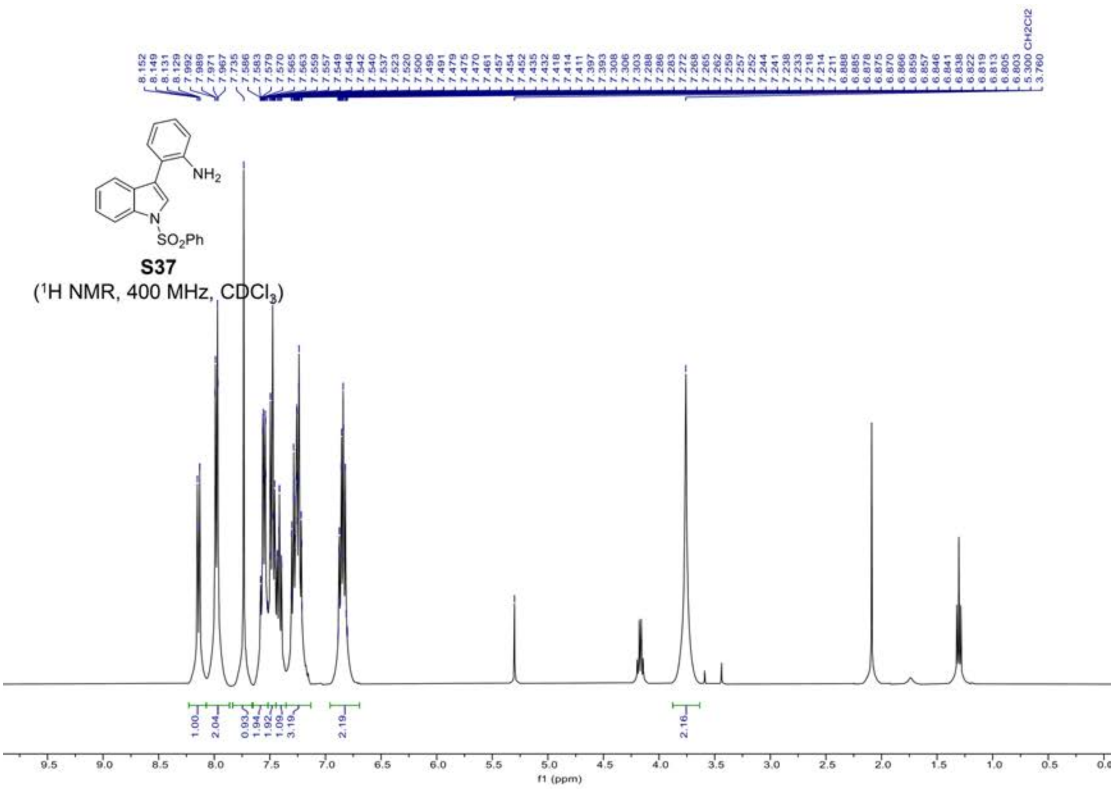

S37
(^{1}H NMR, 400 MHz, $CDCl_3$)

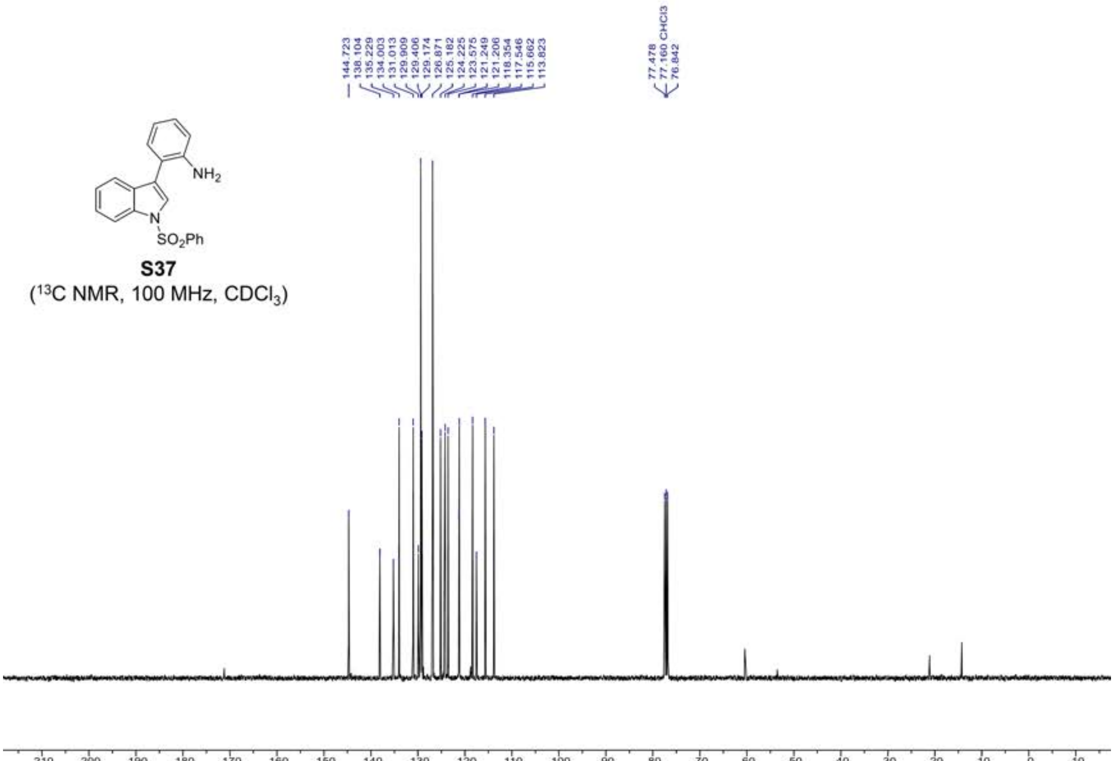

S37
(^{13}C NMR, 100 MHz, $CDCl_3$)

4.2 Synthesis of 6

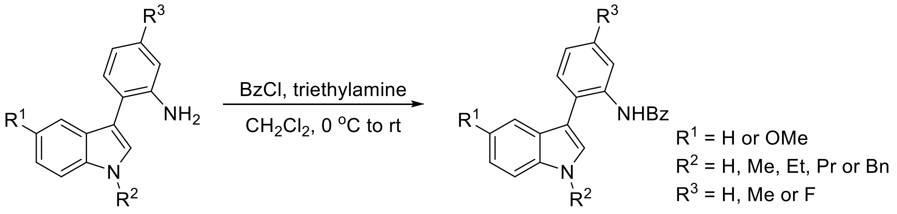

S28–S31 or **S34–S36**, **S38** **6a, 6b, 6d–6i**

Procedure 19: To a round-bottom flask equipped with a magnetic stirring bar, **S28**–**S31** or **S34**–**S36**, **S38** (1.0 equiv), and triethylamine (2.0 equiv) were added and subsequently dissolved in CH_2Cl_2 (0.4 M). Then, benzoyl chloride (1.2 equiv) was added dropwise and allowed to stir for overnight at rt. The reaction was diluted with CH_2Cl_2, transferred to a separatory funnel, and quenched with a saturated aqueous NH_4Cl. The organic layer was separated, and the aqueous layer was extracted an additional two times with CH_2Cl_2. The combined organic layers were then rinsed with water, dried with anhydrous $MgSO_4$, and concentrated *in vacuo*. The crude material was then purified by flash chromatography to afford the desired material **6a**, **6b**, **6d**–**6i**.

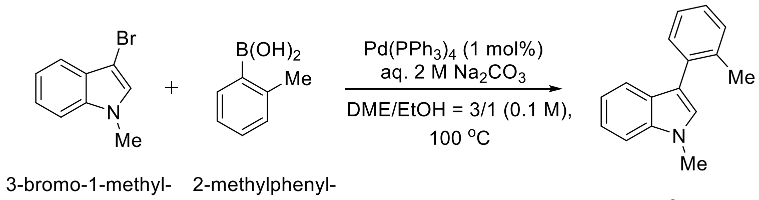

3-bromo-1-methyl-1*H*-indole 2-methylphenylboronic acid **6c**

Procedure 20: An oven-dried round-bottom flask equipped with a magnetic stir bar, a solution of *N*-methyl-3-bromoindole (1.0 equiv) in 1,2-dimethoxyethane was added. Then, $Pd(PPh_3)_4$ (1 mol%) was added to flask and solution of 2-methylphenylboronic acid (1.5 equiv) in EtOH was added. The reaction mixture was heated 4 h at 100 °C in an oil bath after addition of aqueous 2.0 M of Na_2CO_3 solution. The progress of the reaction was monitored by TLC. The reaction mixture was cooled to rt, diluted with CH_2Cl_2 and washed with brine. The organic layer was extracted with CH_2Cl_2 three times, dried with anhydrous $MgSO_4$, filtered, and concentrated *in vacuo*. The crude material was then purified by flash chromatography to afford the desired material **6c**.

Characterization and Spectra of 6

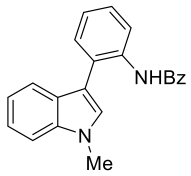

***N*-(2-(1-Methyl-1*H*-indol-3-yl)phenyl)benzamide (6a)** was synthesized by following Procedure 17 from **S28**. The crude material was purified by normal-phase column chromatography using an eluent of 75% CH_2Cl_2/Hx to provide **6a** (418 mg, 64%).

^{1}H NMR (400 MHz, $CDCl_3$) δ 8.69 (d, *J* = 8.1 Hz, 1H), 8.39 (s, 1H), 7.61 (d, *J* = 8.0 Hz, 1H), 7.56–7.33 (m, 7H), 7.33–7.10 (m, 5H), 3.90 (s, 3H).
^{13}C NMR (100 MHz, $CDCl_3$) δ 165.1, 137.3, 136.3, 135.0, 131.6, 131.1, 128.6 (2C), 128.2, 128.1, 126.9 (2C), 126.5, 125.0, 124.1, 122.7, 120.6, 120.5, 120.0, 112.0, 110.0, 33.1.
IR (FT-ATR, cm^{-1}, $CHCl_3$) ν_{max} 3953, 3930, 3903, 3884, 3842, 3826, 3807, 3780, 3757, 3718, 3676, 3652, 3629, 3614, 3591, 3568, 3545, 3402, 3336, 3055, 3012, 2939, 2885, 2823, 2700, 2561, 2411, 2372, 2345, 2318, 2249, 2202, 2137, 2090, 1952, 1917, 1874, 1809, 1743, 1670, 1608, 1577, 1516, 1473, 1446, 1373, 1304, 1238, 1157, 1130, 1080, 1040, 1014, 972, 941, 891, 822, 795, 741, 717, 706.
HRMS (EI) *m*/*z*: $[M]^+$ Calcd for $C_{22}H_{18}N_2O$ 326.1419; found 326.1420.

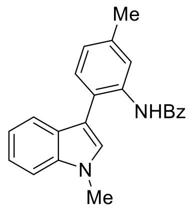

***N*-(5-Methyl-2-(1-methyl-1*H*-indol-3-yl)phenyl)benzamide (6b)** was synthesized by following Procedure 17 from **S29**. The crude material was purified by normal-phase column chromatography using an eluent of 20% EtOAc/Hx to provide **6b** (210 mg, 73%).

^{1}H NMR (400 MHz, $CDCl_3$) δ 8.52 (s, 1H), 8.32 (s, 1H), 7.56 (dt, *J* = 8.0, 1.0 Hz, 1H), 7.50–7.28 (m, 6H), 7.28–7.20 (m, 2H), 7.16 (ddd, *J* = 8.0, 6.9, 1.0 Hz, 1H), 7.12 (s, 1H), 7.03 (dd, *J* = 7.6, 0.9 Hz, 1H), 3.86 (s, 3H), 2.48 (s, 3H).
^{13}C NMR (100 MHz, $CDCl_3$) δ 165.0, 138.1, 137.3, 136.1, 135.0, 131.5, 130.8, 128.6 (2C), 128.1, 126.9 (2C), 126.6, 125.0, 122.7, 122.0, 121.2, 120.4, 120.0, 111.9, 110.0, 33.1, 21.8.
IR (FT-ATR, cm^{-1}, $CHCl_3$) ν_{max} 3988, 3953, 3907, 3884, 3826, 3807, 3780, 3757, 3714, 3680, 3656, 3629, 3591, 3572, 3402, 3336, 3167, 3055, 3012, 2920, 2827, 2735, 2700, 2600, 2549, 2407, 2372, 2349, 2314, 2249, 2175, 2137, 2094, 1917, 1894, 1836, 1797, 1774, 1670, 1604, 1574, 1551, 1520, 1461, 1419, 1373, 1331, 1296, 1242, 1219, 1184, 1157, 1131, 1076, 1018, 930, 887, 813, 741, 706, 663.
HRMS (EI) *m*/*z*: $[M]^+$ Calcd for $C_{23}H_{20}N_2O$ 340.1576; found 340.1577.

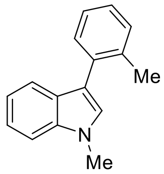

1-Methyl-3-(*o*-tolyl)-1*H*-indole (6c) was synthesized by following Procedure 18. The crude material was purified by normal-phase column chromatography using an eluent of 5% EtOAc/Hx to provide **6c** (438 mg, 99%).

^{1}H NMR (400 MHz, $CDCl_3$) δ 7.65 (dt, *J* = 8.0, 1.0 Hz, 1H), 7.57–7.51 (m, 1H), 7.46 (dt, *J* = 8.3, 0.9 Hz, 1H), 7.42 (tdd, *J* = 5.6, 3.3, 1.3 Hz, 1H), 7.40–7.32 (m, 3H), 7.24 (ddd, *J* = 8.0, 7.0, 1.1 Hz, 1H), 7.13 (s, 1H), 3.90 (s, 3H), 2.45 (s, 3H).
^{13}C NMR (100 MHz, $CDCl_3$) δ 136.79 (2C), 134.65, 130.98, 130.46, 127.63 (2C), 126.65, 125.72, 121.82, 120.34, 119.53, 115.99, 109.43, 32.89, 20.95.

The spectral data were identical with those previously reported.[20]

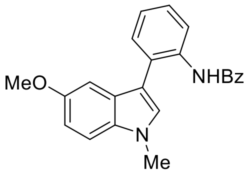

***N*-(2-(5-Methoxy-1-methyl-1*H*-indol-3-yl)phenyl)benzamide (6d)** was synthesized by following Procedure 17 from **S30**. The crude material was purified by normal-phase column chromatography using an eluent of 13% EtOAc/Hx to provide **6d** (262 mg, 74%).

^{1}H NMR (400 MHz, $CDCl_3$) δ 8.68 (d, *J* = 8.2 Hz, 1H), 8.38 (s, 1H), 7.60–7.38 (m, 5H), 7.38–7.18 (m, 4H), 7.14 (s, 1H), 7.05–6.88 (m, 2H), 3.86 (s, 3H), 3.69 (s, 3H).
^{13}C NMR (100 MHz, $CDCl_3$) δ 165.0, 155.0, 136.2, 134.9, 132.4, 131.6, 131.0, 128.7, 128.6 (2C), 128.0, 126.9 (2C), 126.6, 125.2, 124.2, 120.7, 113.5, 111.5, 111.0, 100.9, 55.9, 33.2.
IR (FT-ATR, cm^{-1}, $CHCl_3$) ν_{max} 3977, 3938, 3888, 3861, 3734, 3663, 3575, 3402, 3105, 3059, 3008, 2939, 2831, 2596, 2436, 2349, 2268, 2198, 2133, 2090, 2052, 1959, 1913, 1836, 1670, 1620, 1574, 1518, 1489, 1446, 1385, 964, 895, 864, 841, 795, 748, 717, 706, 656.
HRMS (EI) *m*/*z*: $[M]^+$ Calcd for $C_{23}H_{20}N_2O_2$ 356.1525; found 356.1523.

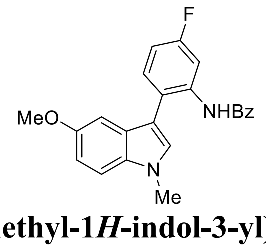

***N*-(5-Fluoro-2-(5-methoxy-1-methyl-1*H*-indol-3-yl)phenyl)benzamide (6e)** was synthesized by following Procedure 17 from **S31**. The crude material was purified by normal-phase column chromatography using an eluent of 5% EtOAc/Hx to provide **6e** (55 mg, 87%).

¹H NMR (400 MHz, $CDCl_3$) δ 8.52 (dd, *J* = 11.4, 2.7 Hz, 1H), 8.40 (brs, 1H), 7.53–7.38 (m, 3H), 7.34 (dt, *J* = 8.5, 3.0 Hz, 2H), 7.31–7.18 (m, 2H), 7.10 (s, 1H), 6.98 (dd, *J* = 8.9, 2.5 Hz, 1H), 6.95–6.78 (m, 2H), 3.86 (s, 3H), 3.67 (s, 3H).
¹³C NMR (100 MHz, $CDCl_3$) δ 165.1, 162.4 (d, *J* = 243.0 Hz, 1C), 155.1, 137.5 (d, *J* = 12.0 Hz, 1C), 134.5, 132.5, 131.92, 131.89, 131.8, 128.8 (2C), 126.9 (2C), 126.6, 120.5 (d, *J* = 3.0 Hz, 1C), 113.6, 111.1, 110.8 (d, *J* = 22.0 Hz, 1C), 110.5, 107.8 (d, *J* = 28.0 Hz, 1C), 100.8, 56.0, 33.3.
IR (FT-ATR, cm^{-1}, $CHCl_3$) ν_{max} 3965, 3946, 3926, 3899, 3880, 3865, 3838, 3822, 3799, 3776, 3730, 3699, 3626, 3595, 3548, 3529, 3398, 3182, 3109, 3062, 3005, 2927, 2835, 2603, 2438, 2360, 2337, 2299, 2268, 2056, 1967, 1894, 1867, 1844, 1824, 1797, 1674, 1597, 1547, 1516, 1489, 1462, 1427, 1381, 1342, 1288, 1250, 1215, 1157, 1134, 1092, 1076, 1030, 976, 930, 852, 798, 756, 706, 683, 656.
HRMS (EI) *m*/*z*: $[M]^+$ Calcd for $C_{23}H_{19}FN_2O_2$ 374.1431; found 374.1428.

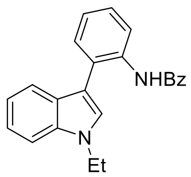

***N*-(2-(1-Ethyl-1*H*-indol-3-yl)phenyl)benzamide (6f)** was synthesized by following Procedure 17 from **S34**. The crude material was purified by normal-phase column chromatography using an eluent of 75% CH_2Cl_2/Hx to provide **6f** (306.2 mg, 90%).

¹H NMR (400 MHz, $CDCl_3$) δ 8.66 (dd, *J* = 8.3, 1.3 Hz, 1H), 8.41 (s, 1H), 7.59 (dd, *J* = 8.0, 1.1 Hz, 1H), 7.51–7.36 (m, 6H), 7.32 (ddd, *J* = 8.3, 7.0, 1.1 Hz, 1H), 7.29–7.21 (m, 4H), 7.21–7.13 (m, 1H), 4.25 (q, *J* = 7.3 Hz, 2H), 1.52 (t, *J* = 7.3 Hz, 3H).
¹³C NMR (100 MHz, $CDCl_3$) δ 165.0, 136.4, 136.2, 135.0, 131.6, 131.0, 128.6 (2C), 128.0, 127.0 (2C), 126.8, 126.3, 125.0, 124.2, 122.6, 120.6, 120.5, 120.1, 112.0, 110.0, 41.3, 15.6.
IR (FT-ATR, cm^{-1}, $CHCl_3$) ν_{max} 3820, 3726, 3624, 3400, 3057, 2977, 2933, 2371, 2339, 2328, 1811, 1772, 1668, 1606, 1579, 1543, 1511, 1493, 1470, 1441, 1389, 1370, 1354, 1337, 1306, 1255, 1243, 1223, 1204, 1159, 1136, 1099, 1085, 1074, 1041, 1027, 1013, 962, 946, 930, 892, 871, 843, 821, 795, 740, 703, 667.
HRMS (EI) *m*/*z*: $[M]^+$ Calcd for $C_{23}H_{20}N_2O$ 340.1576; found 340.1577.

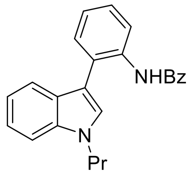

***N*-(2-(1-Propyl-1*H*-indol-3-yl)phenyl)benzamide (6g)** was synthesized by following Procedure 17 from **S35**. The crude material was purified by normal-phase column chromatography using an eluent of 75% CH_2Cl_2/Hx to provide **6g** (273 mg, 96%).

¹H NMR (400 MHz, $CDCl_3$) δ 8.64 (dd, *J* = 8.3, 1.2 Hz, 1H), 8.39 (s, 1H), 7.57 (dt, *J* = 8.0, 1.0 Hz, 1H), 7.48–7.35 (m, 6H), 7.29 (ddd, *J* = 8.3, 7.0, 1.1 Hz, 1H), 7.27–7.17 (m, 4H), 7.14 (ddd, *J* = 8.0, 7.0, 1.0 Hz, 1H), 4.14 (t, *J* = 7.0 Hz, 2H), 1.90 (h, *J* = 7.3 Hz, 2H), 0.93 (t, *J* = 7.4 Hz, 3H).

^{13}**C NMR** (100 MHz, $CDCl_3$) δ 165.0, 136.7, 136.2, 135.0, 131.6, 131.0, 128.6 (2C), 128.0, 127.1, 126.9 (2C), 126.7, 125.0, 124.1, 122.6, 120.6, 120.4, 120.0, 111.8, 110.2, 48.3, 23.7, 11.7.
IR (FT-ATR, cm^{-1}, $CHCl_3$) ν_{max} 3727, 3624, 3400, 3058, 2964, 2931, 2874, 2371, 2339, 2328, 1668, 1608, 1579, 1543, 1512, 1493, 1469, 1441, 1389, 1369, 1306, 1250, 1219, 1200, 1160, 1137, 1102, 1073, 1042, 1027, 1014, 977, 943, 892, 795, 741, 703, 666.
HRMS (EI) *m*/*z*: $[M]^+$ Calcd for $C_{24}H_{22}N_2O$ 354.1732; found 354.1730.

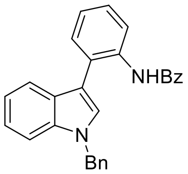

***N*-(2-(1-Benzyl-1*H*-indol-3-yl)phenyl)benzamide (6h)** was synthesized by following Procedure 17 from **S36**. The crude material was purified by normal-phase column chromatography using an eluent of 13% EtOAc/Hx to provide **6h** (239 mg, 63%).

^{1}**H NMR** (400 MHz, $CDCl_3$) δ 8.65 (d, *J* = 8.1 Hz, 1H), 8.37 (s, 1H), 7.60 (d, *J* = 7.9 Hz, 1H), 7.49–7.36 (m, 6H), 7.32–7.11 (m, 11H), 5.37 (s, 2H).
^{13}**C NMR** (100 MHz, $CDCl_3$) δ 165.0, 136.9, 136.9, 136.2, 135.0, 131.6, 131.0, 129.0 (2C), 128.7 (2C), 128.1, 128.0, 127.4, 127.02, 127.01 (2C), 126.9 (2C), 124.8, 124.2, 123.0, 120.8 (2C), 120.7, 120.1, 112.5, 110.5, 50.4.
IR (FT-ATR, cm^{-1}, $CHCl_3$) ν_{max} 3950, 3926, 3899, 3838, 3799, 3776, 3730, 3703, 3626, 3599, 3545, 3525, 3402, 3325, 3059, 3032, 2927, 2862, 2603, 2565, 2407, 2360, 2333, 2299, 2268, 2071, 2017, 1990, 1948, 1921, 1894, 1871, 1828, 1801, 1774 ,1666, 1601, 1581, 1515, 1446, 1308, 1250, 1219, 1176, 1115, 1072, 1026, 972, 941, 895, 841, 818, 744, 702, 656.
HRMS (EI) *m*/*z*: $[M]^+$ Calcd for $C_{28}H_{22}N_2O$ 402.17321; found 402.1732.

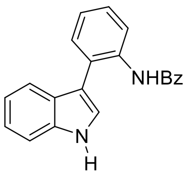

***N*-(2-(1*H*-Indol-3-yl)phenyl)benzamide (6i)** was synthesized by following Procedure 17 from **S38**. The crude material was purified by normal-phase column chromatography using an eluent of 13% EtOAc/Hx to provide **6i** (434 mg, 93%).

^{1}**H NMR** (400 MHz, $CDCl_3$) δ 8.65 (dd, *J* = 8.7, 1.3 Hz, 1H), 8.53 (d, *J* = 7.4 Hz, 1H), 8.32 (s, 1H), 7.59 (dd, *J* = 7.9, 1.0 Hz, 1H), 7.55–7.48 (m, 1H), 7.48–7.36 (m, 5H), 7.34–7.15 (m, 8H).
^{13}**C NMR** (100 MHz, $CDCl_3$) δ 165.2, 136.4, 136.3, 135.0, 131.7, 131.1, 128.7 (2C), 128.3, 127.0 (2C), 126.2, 124.9, 124.3, 123.6, 123.3, 121.0, 120.7, 119.9, 113.5, 111.9.

The spectral data were identical with those previously reported.[21]

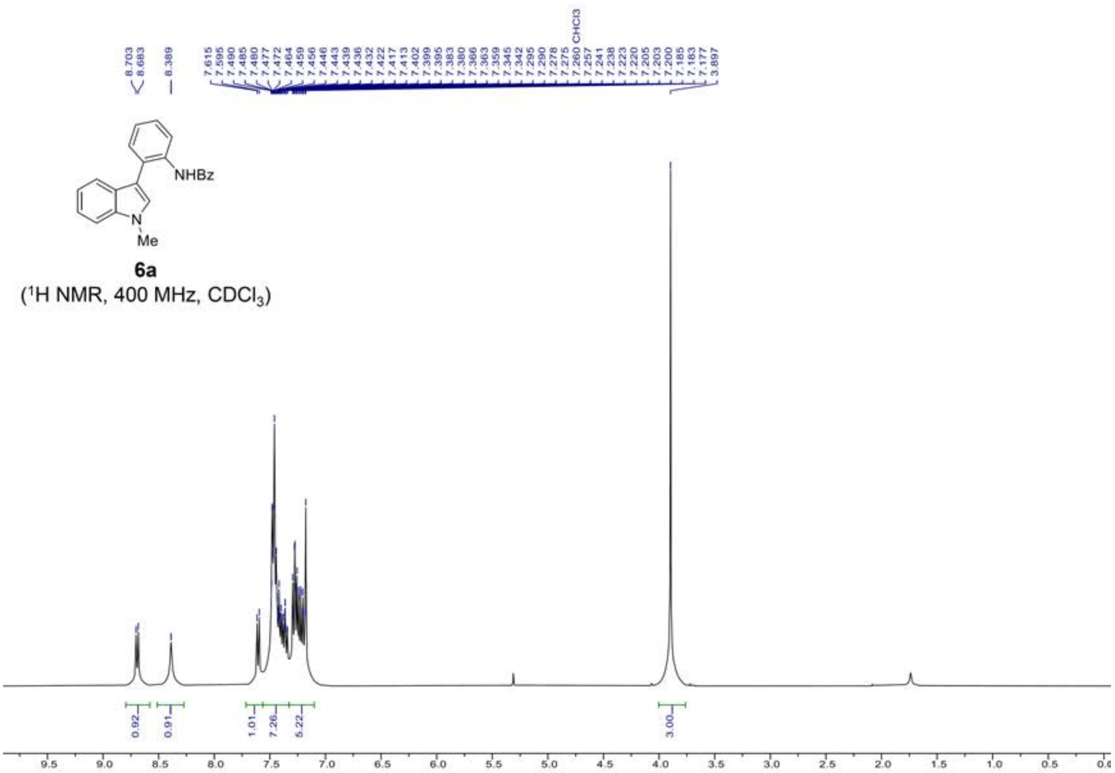

6a
(^{1}H NMR, 400 MHz, $CDCl_3$)

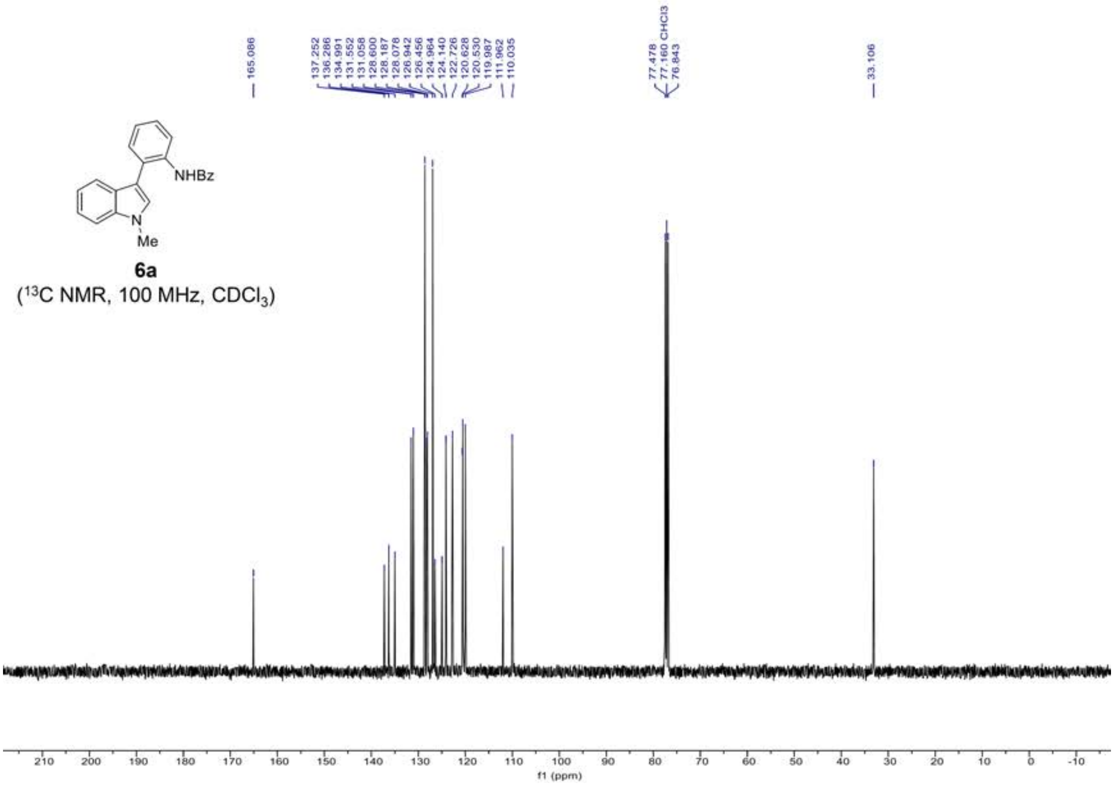

6a
(^{13}C NMR, 100 MHz, $CDCl_3$)

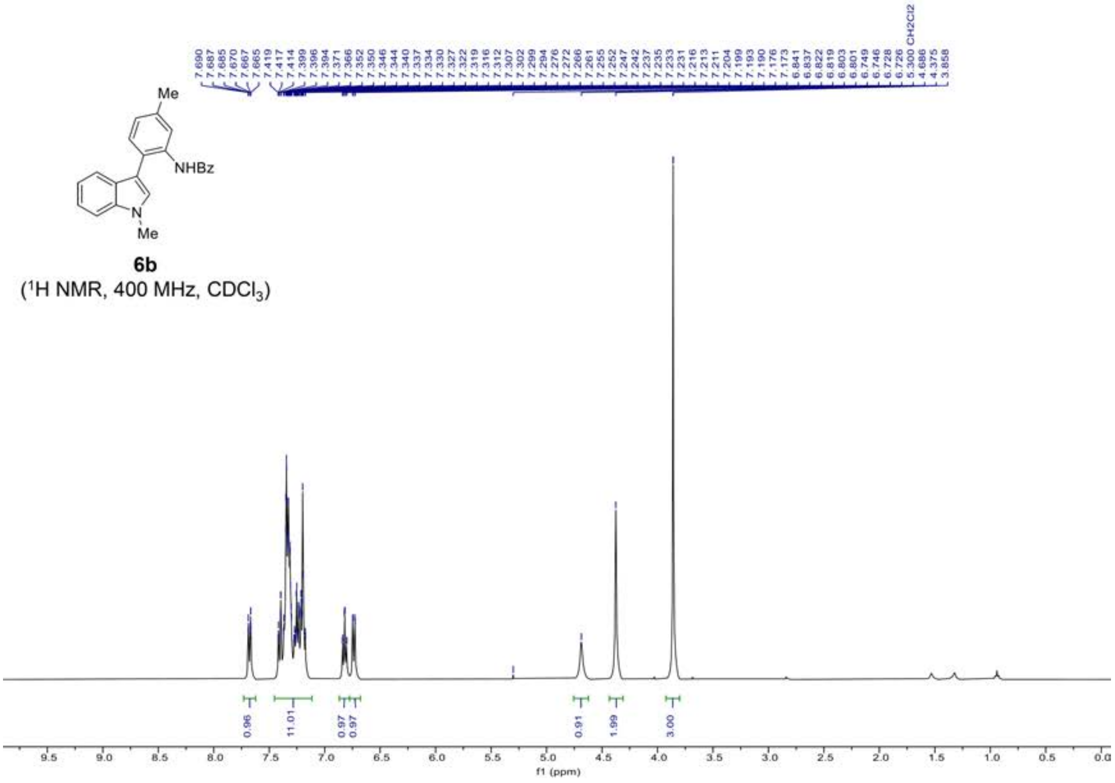

6b
(^{1}H NMR, 400 MHz, $CDCl_3$)

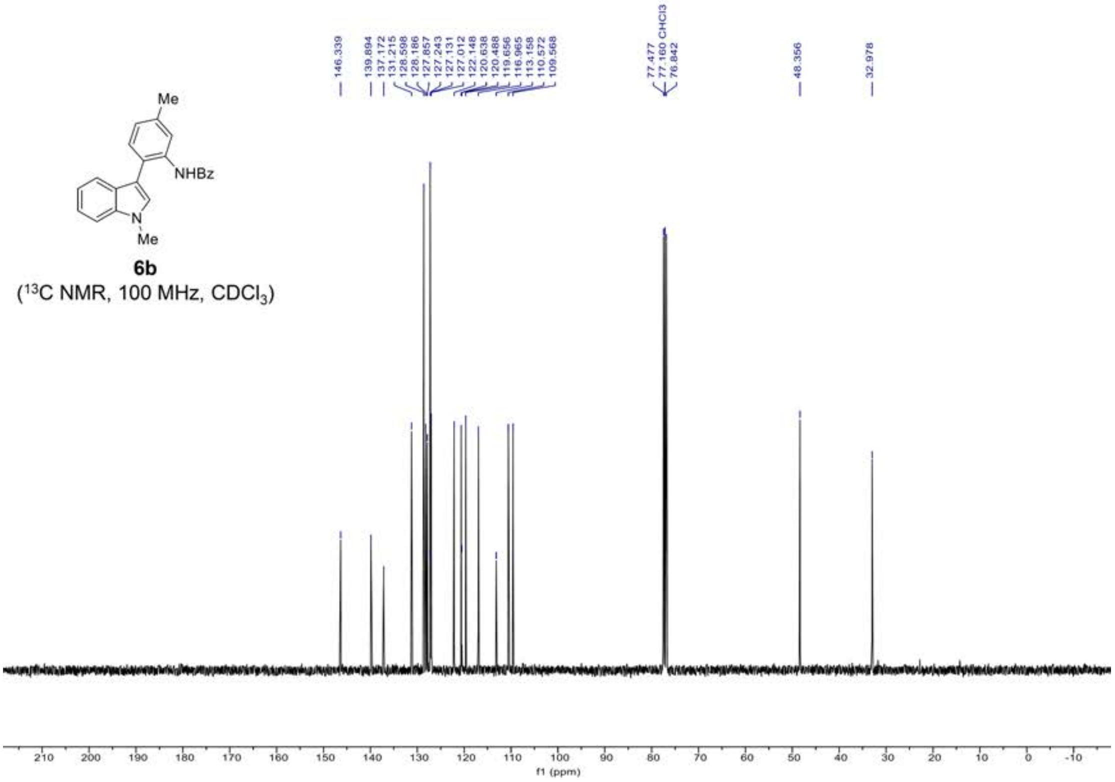

6b
(^{13}C NMR, 100 MHz, $CDCl_3$)

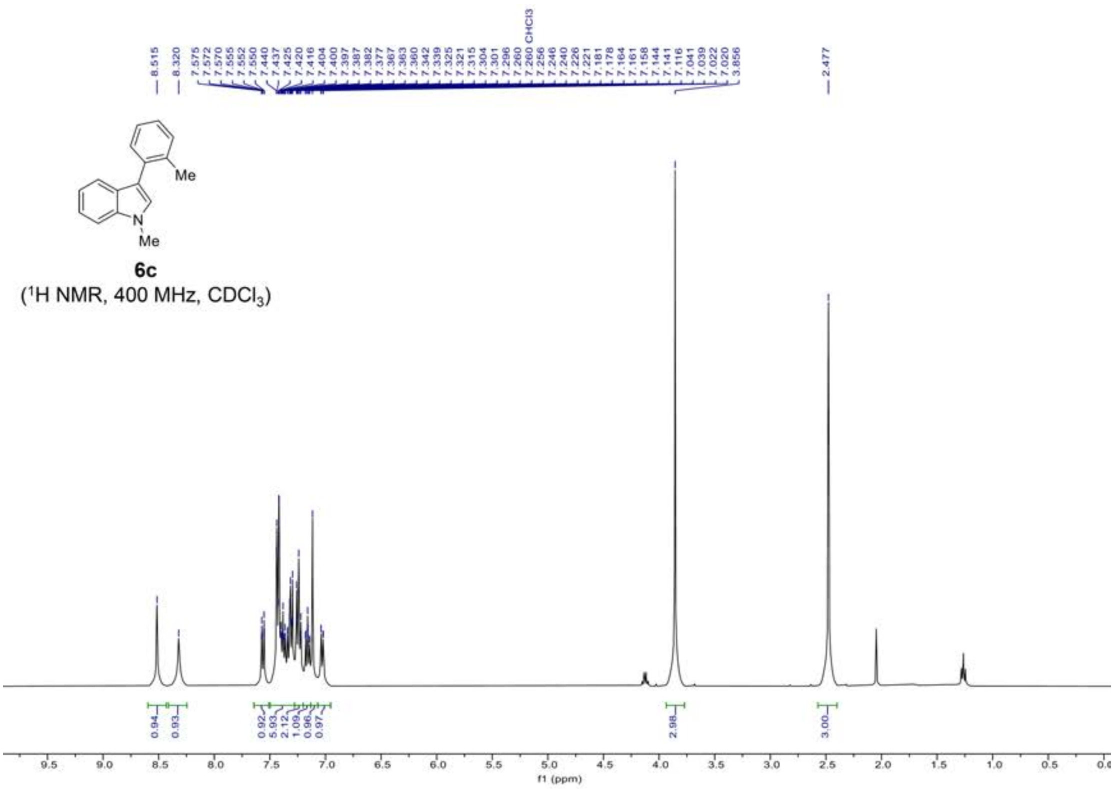

6c

(^{1}H NMR, 400 MHz, $CDCl_3$)

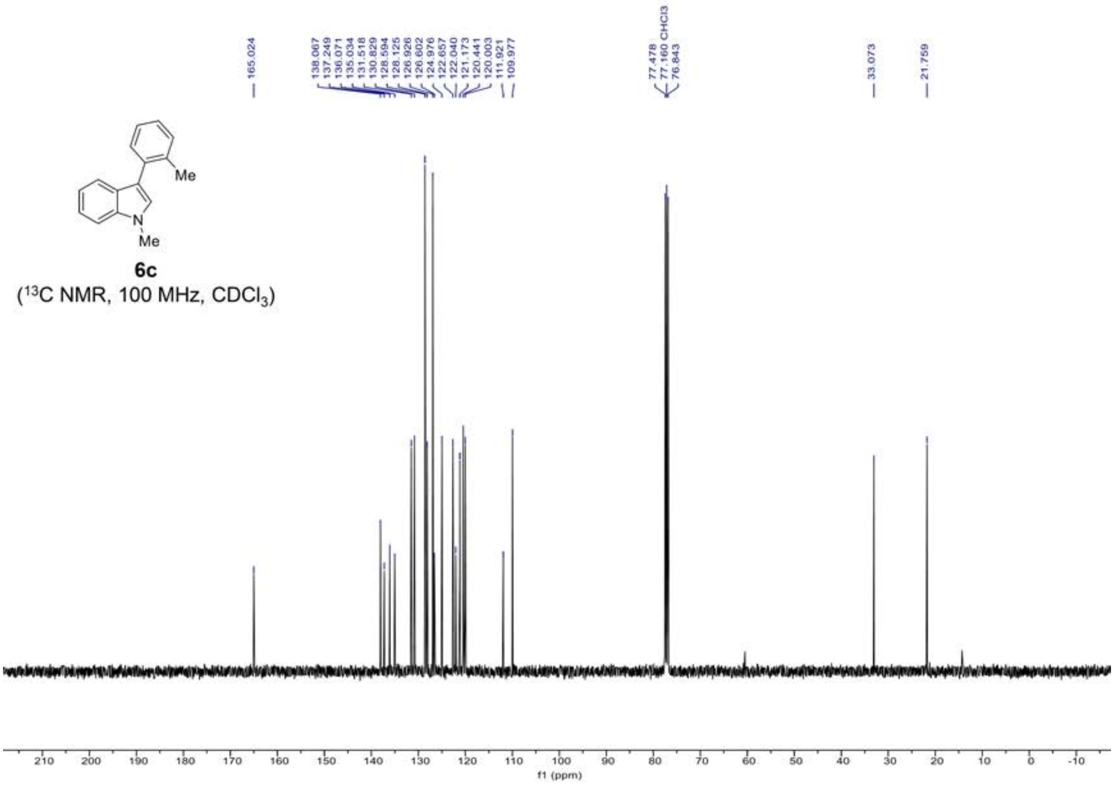

6c

(^{13}C NMR, 100 MHz, $CDCl_3$)

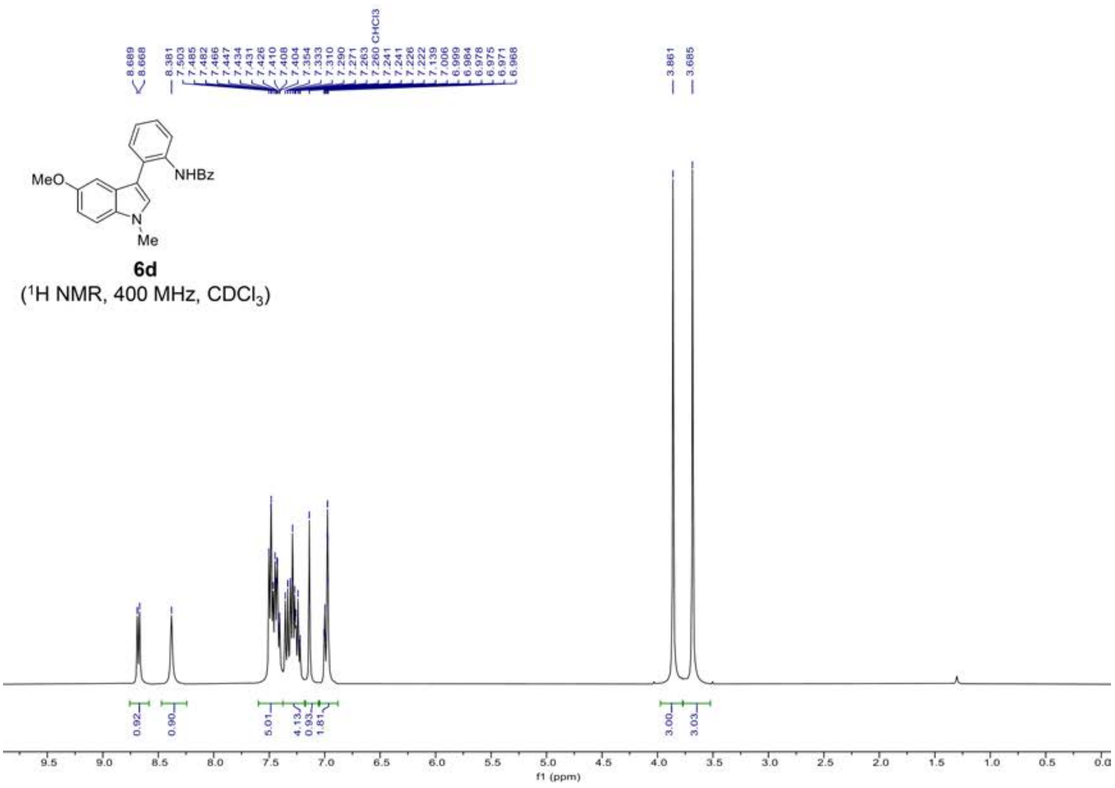

6d
(^{1}H NMR, 400 MHz, $CDCl_3$)

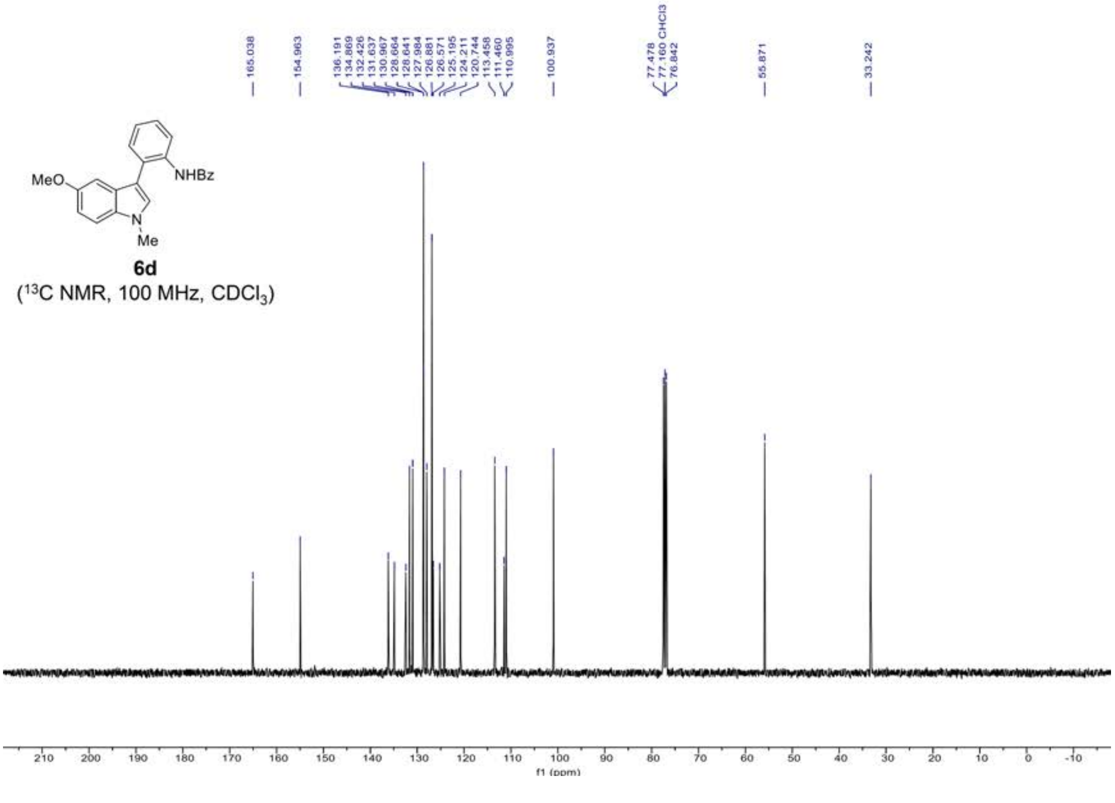

6d
(^{13}C NMR, 100 MHz, $CDCl_3$)

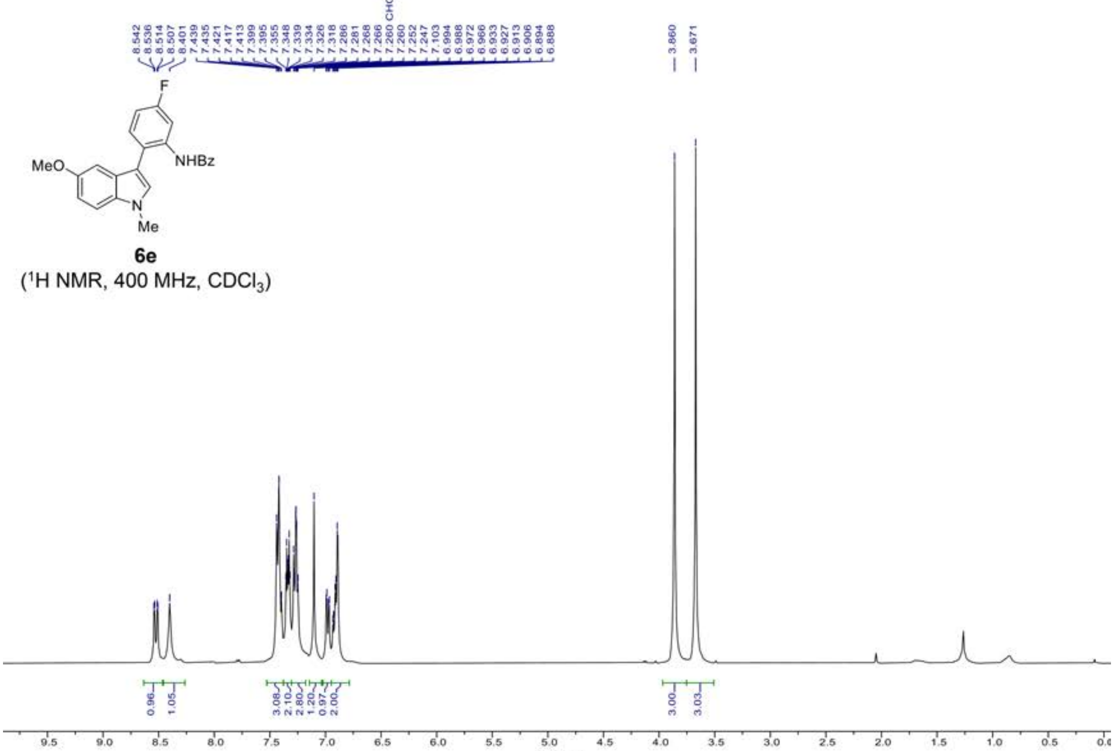

6e
(^{1}H NMR, 400 MHz, $CDCl_3$)

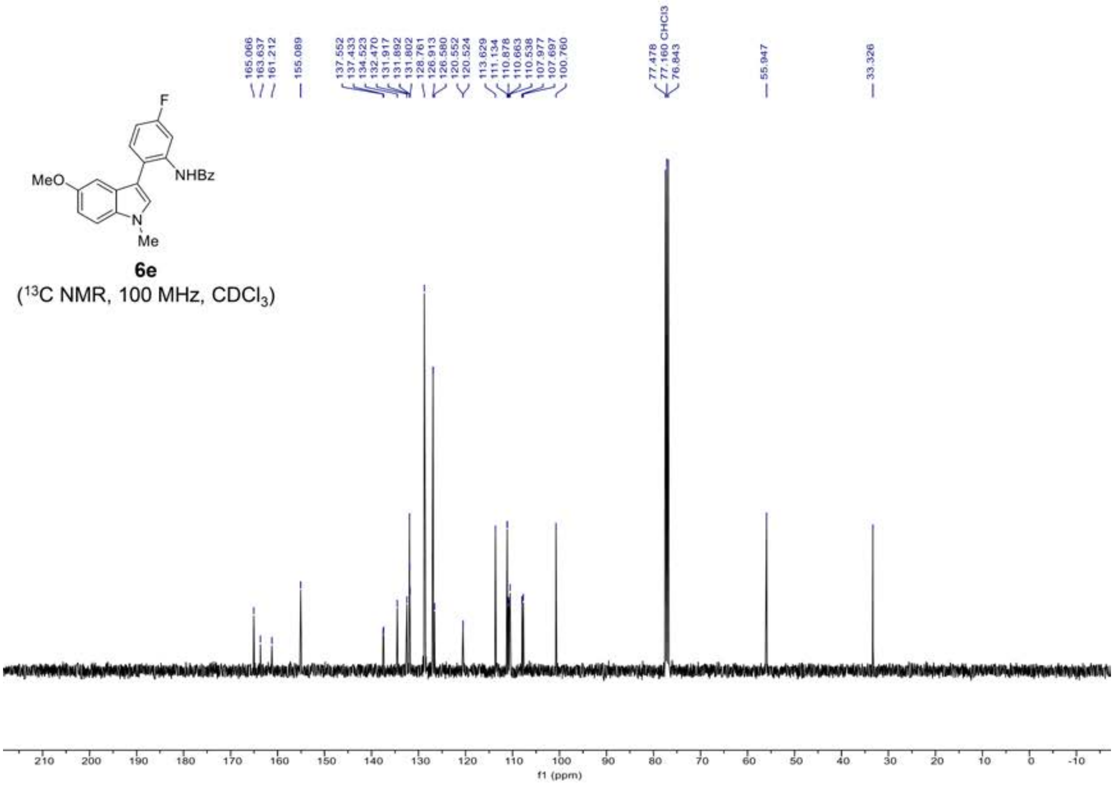

6e
(^{13}C NMR, 100 MHz, $CDCl_3$)

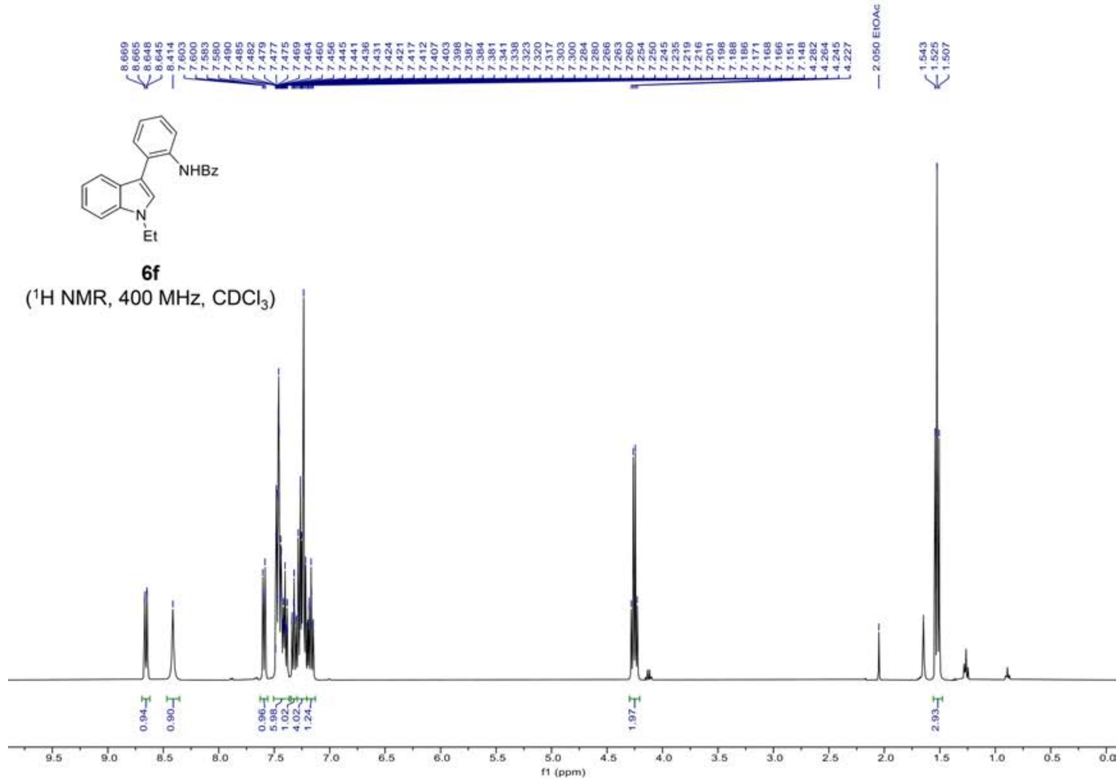

6f
(^{1}H NMR, 400 MHz, CDCl$_3$)

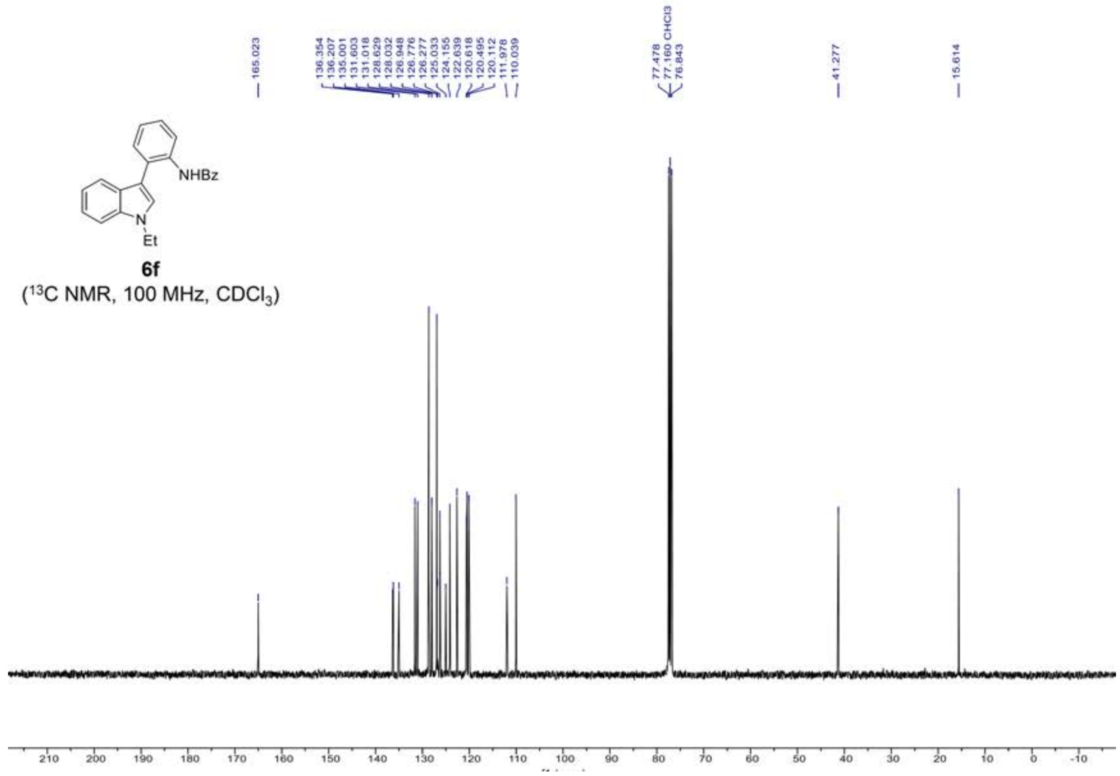

6f
(^{13}C NMR, 100 MHz, CDCl$_3$)

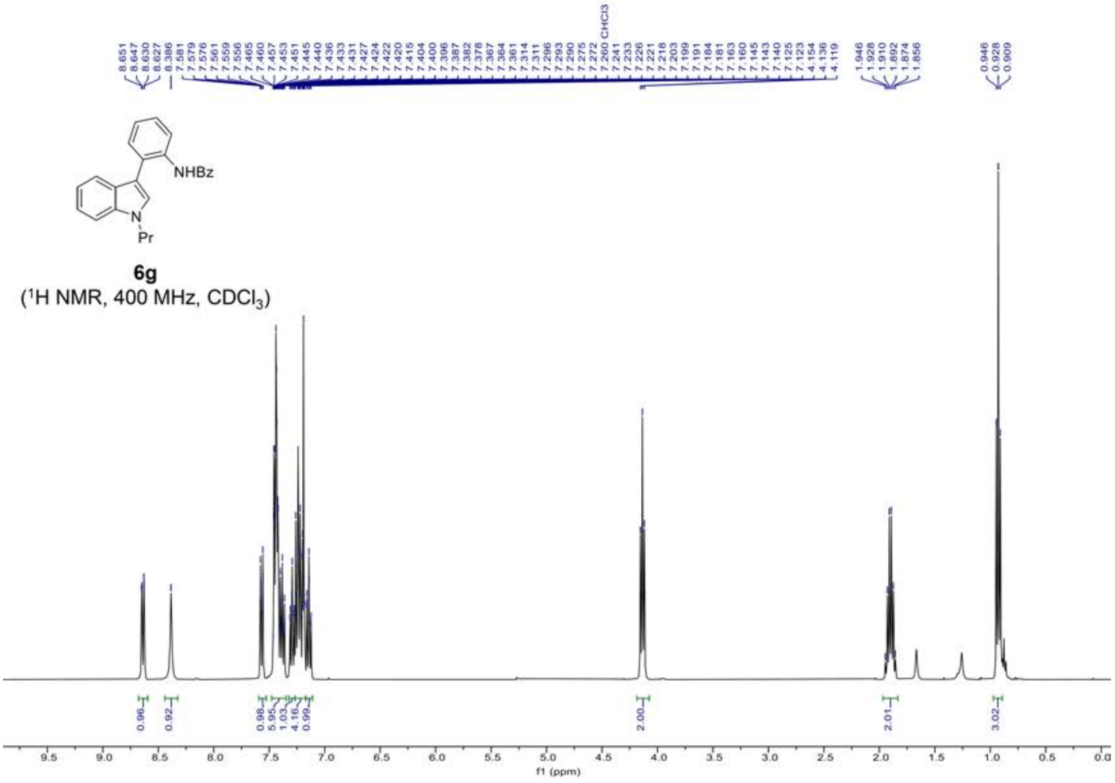

6g
(^{1}H NMR, 400 MHz, $CDCl_3$)

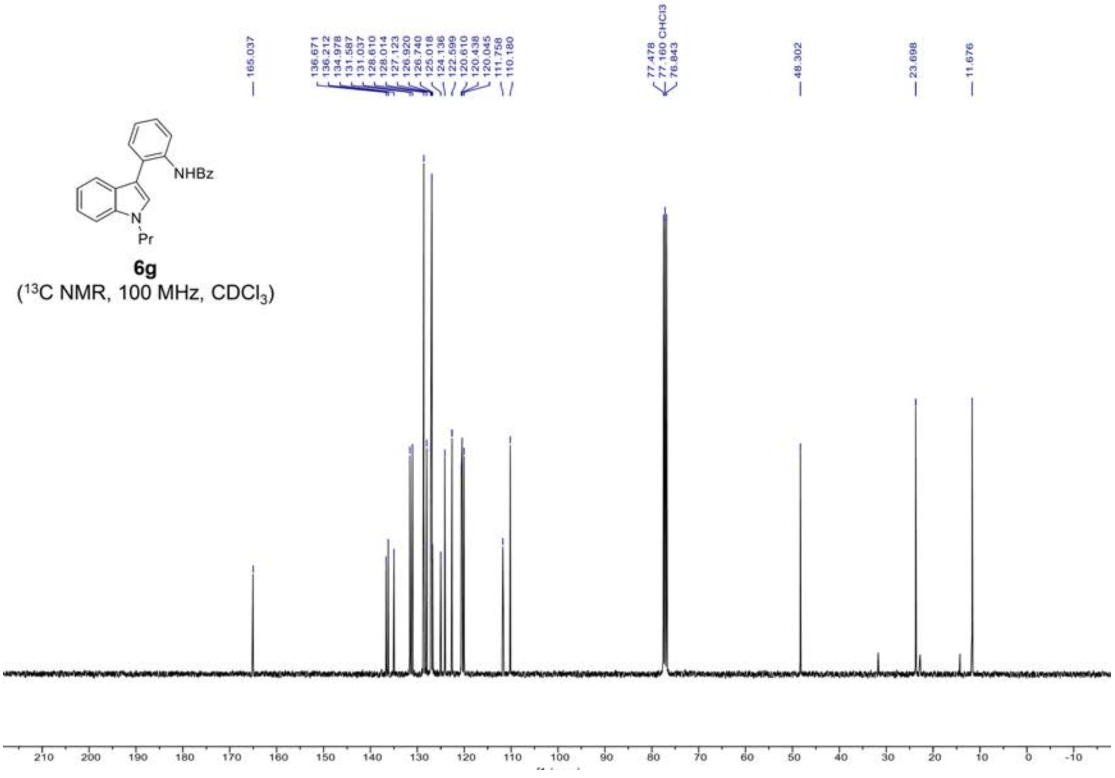

6g
(^{13}C NMR, 100 MHz, $CDCl_3$)

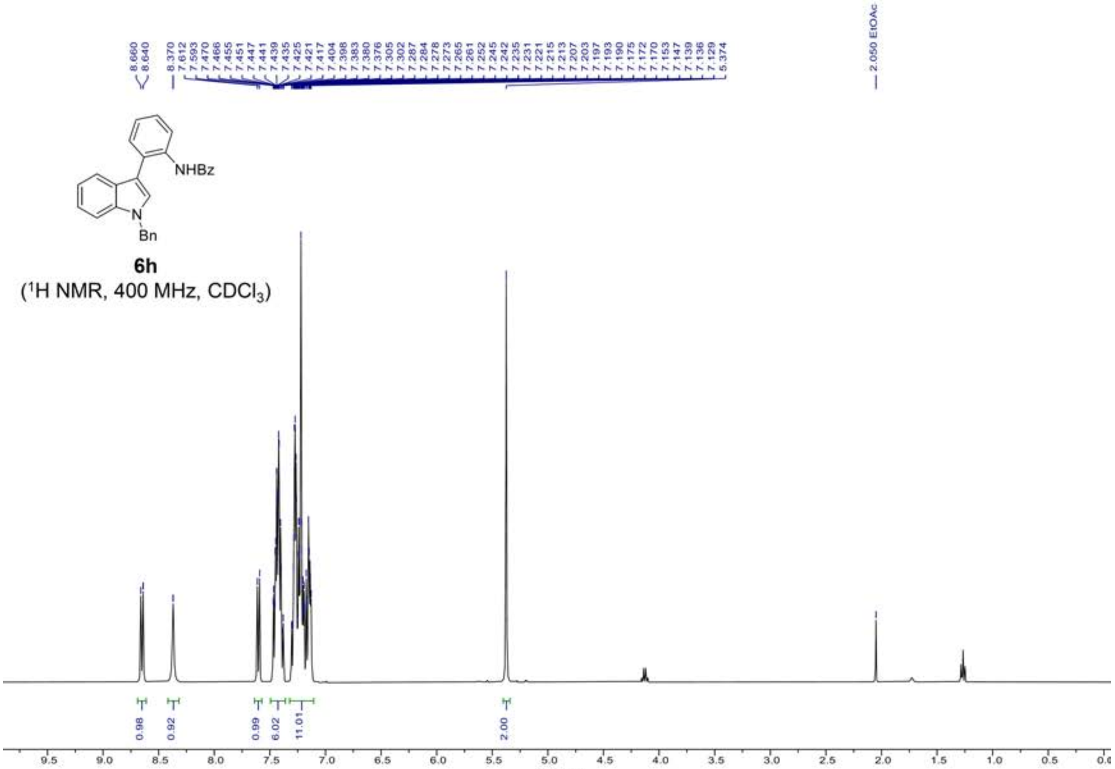

6h
(^{1}H NMR, 400 MHz, CDCl$_3$)

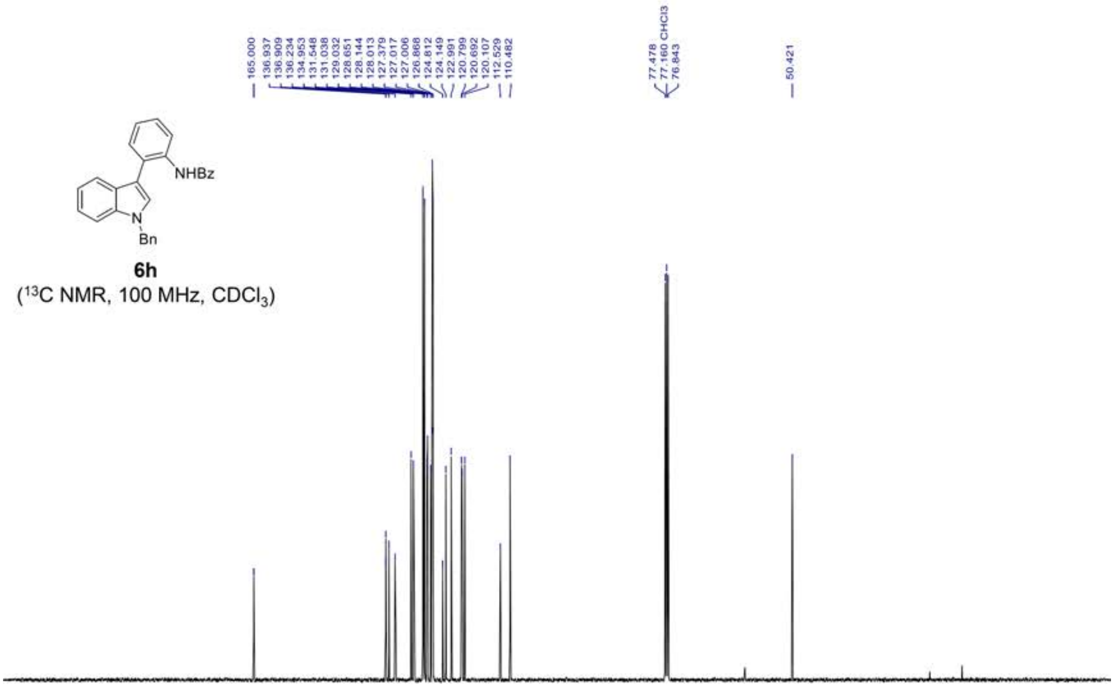

6h
(^{13}C NMR, 100 MHz, CDCl$_3$)

5 Reaction Optimizations for 3, 5 and 7

5.1 Reaction Optimizations for 3

5.1.1 Catalyst Screening

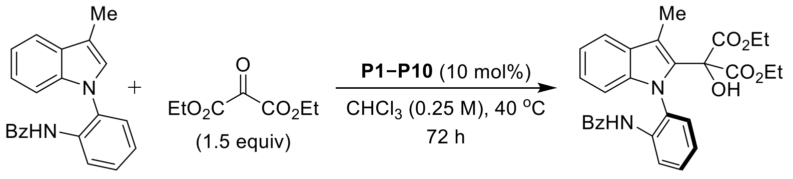

1a **2a** **3aa**

To an oven dried 4 mL vial equipped with a magnetic stir bar was added substrate **1a** (16.3 mg, 0.05 mmol, 1 equiv) and **P1**–**P10** (0.005 mmol, 0.1 equiv), a solution of diethyl ketomalonate **2a** (11.4 μL, 0.075 mmol, 1.5 equiv) in $CHCl_3$ (0.20 mL, 0.25 M) was added. The vial was sealed with a Teflon cap and further secured with Parafilm M®. The reaction mixture was left to stir at 40 °C, and then the solvent was removed *in vacuo*. The crude material was purified by flash column chromatography using an eluent of 5% EtOAc/CH_2Cl_2 to afford the desired material **3aa**. The enantioselectivity was determined by chiral HPLC.

Supplementary Table 1.

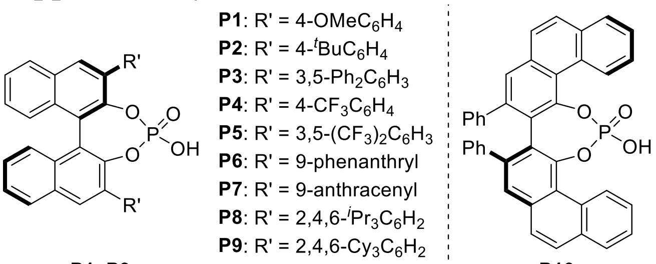

P1–P9 **P10**

Entry	Catalyst	Yield[a] (%)	e.e.[b] (%)
1	**P1**	56	0
2	**P2**	64	0
3	**P3**	69	26
4	**P4**	83	(20)[c]
5	**P5**	80	(3)[c]
6	45	55	45
7	**P7**	95	59
8	**P8**	90	69
9	**P9**	86	82
10	**P10**	46	1

[a]Isolated yields. [b]Enantiomeric excesses were determined by chiral HPLC analysis. [c]The opposite enantiomer was obtained as a major isomer.

5.1.2 Solvent Screening

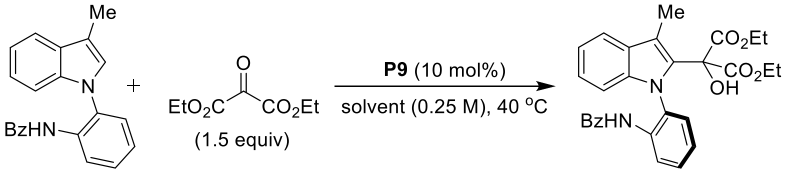

1a **2a** **3aa**

To an oven dried 4 mL vial equipped with a magnetic stir bar was added substrate **1a** (16.3 mg, 0.05 mmol, 1 equiv) and **P9** (5 mg, 0.005 mmol, 0.1 equiv). Then, the solution of diethyl ketomalonate **2a** (11.4 µL, 0.075 mmol, 1.5 equiv) in solvent (0.20 mL, 0.25 M) was added. The vial was sealed with a Teflon cap and further secured with Parafilm M®. The reaction mixture was stirred until complete consumption of **1a**. Then the solvent was removed *in vacuo*. The crude material was purified by flash column chromatography using an eluent of 5% EtOAc/CH_2Cl_2 to afford the desired material **3**. The enantioselectivity was determined by chiral HPLC.

Supplementary Table 2.

Entry	Solvent	Yield[a] (%)	e.e.[b] (%)
1	PhMe	99	89
2	MeCN	≤ 10	n.d.[c]
3	THF	≤ 10	n.d.[c]
4	CH_2Cl_2	97	90
5	$CHCl_3$	94	83
6	DMF	≤ 10	n.d.[c]
7	1,4-dioxane	≤ 10	n.d.[c]
8	cyclohexane	83	87
9	1,2-dichloroethne	76	85
10	tetrachloroethane	64	68
11	chlorobenzene	91	89

[a]Isolated yields. [b]Enantiomeric excesses were determined by chiral HPLC analysis. [c]Not determined.

5.1.3 Temperature and Additive Screening

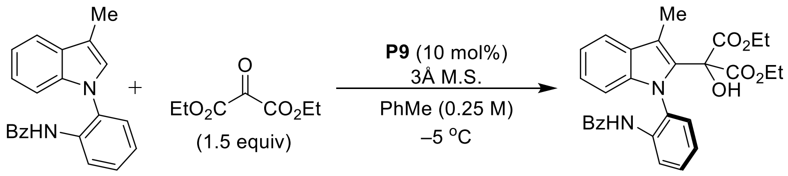

To an oven dried 4 mL vial equipped with a magnetic stir bar was added substrate **1a** (16.3 mg, 0.05 mmol, 1 equiv) and **P9** (5 mg, 0.005 mmol, 0.1 equiv). Then, the solution of diethyl ketomalonate **2a** (11.4 µL, 0.075 mmol, 1.5 equiv) in PhMe (0.2 mL, 0.25 M) was added. The vial was sealed with a Teflon cap and further secured with Parafilm M®. The reaction mixture was stirred until complete consumption of **1a**. Then the solvent was removed *in vacuo*. The crude material was purified by flash column chromatography using an eluent of 5% EtOAc/CH_2Cl_2 to afford the desired material **3**. The enantioselectivity was determined by chiral HPLC.

Supplementary Table 3.

Entry	Temp (°C)	Additive	Time (h)	Yield[a] (%)	e.e.[b] (%)
1	40	-	72	99	89
2	rt	-	72	90	90
3	–5	-	72	74	95
4	–5	3 Å M.S.[c]	72	99	96

[a]Isolated yields. [b]Enantiomeric excesses were determined by chiral HPLC analysis. [c]40 mg of 3 Å molecular sieves was added.

5.1.4 Electrophile Screening

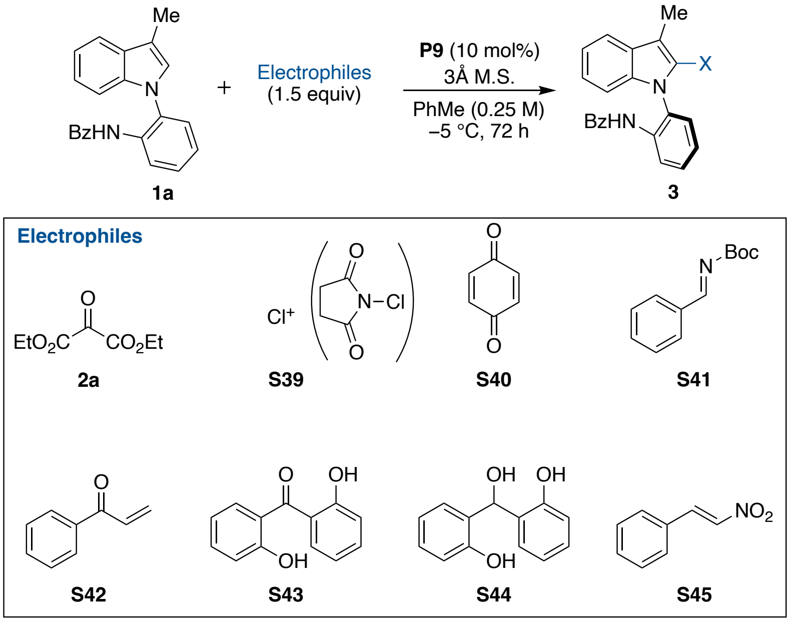

To an oven dried 4 mL vial equipped with a magnetic stir bar was added substrate **1a** (16.3 mg, 0.05 mmol, 1 equiv), **P9** (5 mg, 0.005 mmol, 0.1 equiv) and 3 Å molecular sieves (40 mg). Then, the solution of electrophile (0.075 mmol, 1.5 equiv) in PhMe (0.2 mL, 0.25 M) was added. The vial was sealed with a Teflon cap and further secured with Parafilm M®. The reaction mixture was stirred at –5 ºC for 72 h. Then the solvent was removed *in vacuo*. The crude material was purified by flash column chromatography to afford the desired material **3**. The enantioselectivity was determined by chiral HPLC.

Supplementary Table 4.

Entry	Electrophile	Time (h)	Yield[a] (%)	e.e.[b] (%)
1	**2a**	72	99	96
2	**S39**	72	32	3
3	**S40**	72	<5	-
4	**S41**	72	<5	-
5	**S42**	72	<5	-
6	**S43**	72	<5	-
7	**S44**	72	<5	-
8	**S45**	72	<5	-

[a]Isolated yields. [b]Enantiomeric excesses were determined by chiral HPLC analysis.

5.2 Reaction Optimizations for 5

5.2.1 Concentration and Additive Screening

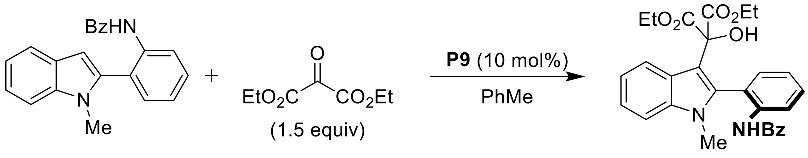

To an oven dried 20 mL vial equipped with a magnetic stir bar was added substrate **4a** (0.05 mmol, 1 equiv) and **P9** (0.005 mmol, 0.1 equiv). Then, the solution of diethyl ketomalonate **2a** (11.4 μL, 0.075 mmol, 1.5 equiv) in PhMe was added. The vial was sealed with a Teflon cap and further secured with Parafilm M®. The reaction mixture was stirred at –20 ºC until complete consumption of **4a**. Then the solvent was removed *in vacuo*. The crude material was purified by flash column chromatography using an eluent of 5% EtOAc/CH_2Cl_2 to afford the desired material **5aa**. The enantioselectivity was determined by chiral HPLC.

Supplementary Table 5.

Entry	Temp (ºC)	Conc. (M)	Additive	Time (h)	Yield[a] (%)	e.e.[b] (%)
1[c]	–5	0.25	3 Å M.S.[d]	0.2	99	66
2	–20	0.25	-	0.5	99	63
3	–20	0.05	-	6	98	74
4	–20	0.025	-	12	92	76
5	–20	0.025	3 Å M.S.[e]	12	99	76

[a]Isolated yields. [b]Enantiomeric excesses were determined by chiral HPLC analysis. [c]Optimized reaction condition of **3**. [d]3 Å molecular sieves was added 40 mg. [e]3 Å molecular sieves was added 400 mg.

5.2.2 Temperature Screening

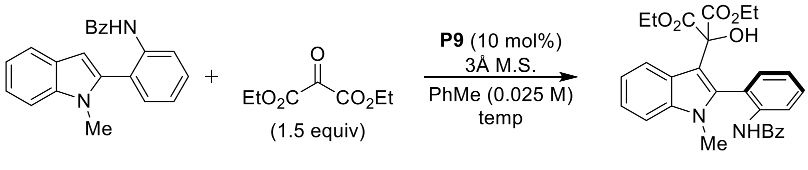

4a **2a** **5aa**

To an oven dried 20 mL vial equipped with a magnetic stir bar was added substrate **4a** (0.05 mmol, 1 equiv), **P9** (0.005 mmol, 0.1 equiv) and 3 Å molecular sieves (400 mg). Then, the solution of diethyl ketomalonate **2a** (11.4 μL, 0.075 mmol, 1.5 equiv) in PhMe (2.0 mL, 0.025 M) was added. The vial was sealed with a Teflon cap and further secured with Parafilm M®. The reaction mixture was stirred until complete consumption of **4a**. Then the solvent was removed *in vacuo*. The crude material was purified by flash column chromatography using an eluent of 5% $EtOAc/CH_2Cl_2$ to afford the desired material **5aa**. The enantioselectivity was determined by chiral HPLC.

Supplementary Table 6.

Entry	Temp (°C)	Time (h)	Yield[a] (%)	e.e.[b] (%)
1	–5	2	91	72
2	–20	12	99	76
3	–47	15	99	81
5	–78	72	99	83

[a]Isolated yields. [b]Enantiomeric excesses were determined by chiral HPLC analysis.

5.2.3 Catalyst Screening

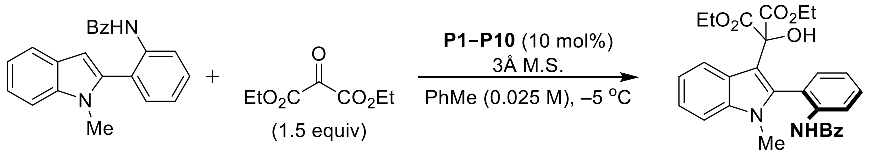

4a **2a** **5aa**

To an oven dried 20 mL vial equipped with a magnetic stir bar was added substrate **4a** (0.05 mmol, 1 equiv), **P1**–**10** (0.005 mmol, 0.1 equiv) and 3 Å molecular sieves (400 mg). Then, the solution of diethyl ketomalonate **2a** (11.4 µL, 0.075 mmol, 1.5 equiv) in PhMe (2.0 mL, 0.025 M) was added. The vial was sealed with a Teflon cap and further secured with Parafilm M®. The reaction mixture was stirred at –5 ºC until complete consumption of **4a**. Then the solvent was removed *in vacuo*. The crude material was purified by flash column chromatography using an eluent of 5% EtOAc/CH_2Cl_2 to afford the desired material **5aa**. The enantioselectivity was determined by chiral HPLC.

Supplementary Table 7.

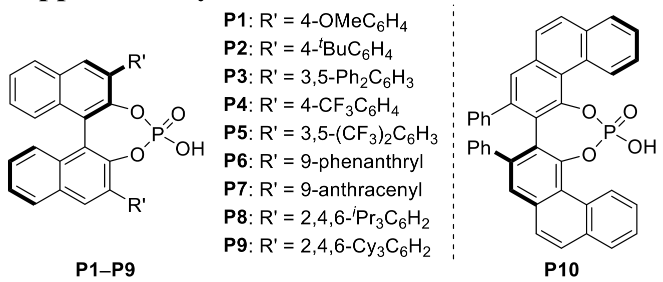

Entry	Catalyst	Time (h)	Yield[a] (%)	e.e.[b] (%)
1	**P1**	60	62	(3)[c]
2	**P2**	60	88	(13)[c]
3	**P3**	6	88	14
4	**P4**	6	97	(19)[c]
5	**P5**	24	92	19
6	**P6**	60	92	25
7	**P7**	1.5	94	31
8	**P8**	3	90	39
9	**P9**	2	91	72
10	**P10**	60	96	4

[a]Isolated yields. [b]Enantiomeric excesses were determined by chiral HPLC analysis. [c]The opposite enantiomer was obtained as a major isomer.

5.3 Reaction Optimizations for 7

5.3.1 Lewis Acid Screening

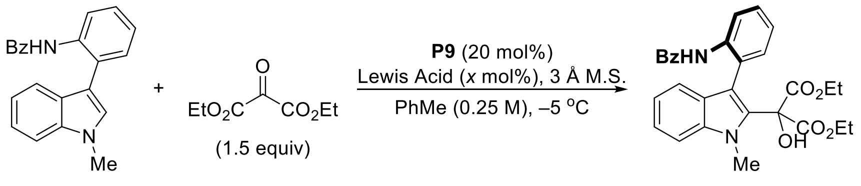

To an oven dried 4 mL vial equipped with a magnetic stir bar was added substrate **6a** (0.05 mmol, 1 equiv), **P9** (20 mol%), lewis acids (*x* mol%) and 3 Å molecular sieves (40 mg). Then, the solution of diethyl ketomalonate **2a** (*x* equiv) in PhMe (0.2 mL, 0.25 M) was added. The vial was sealed with a Teflon cap and further secured with Parafilm M®. The reaction mixture was stirred at –5 ºC until complete consumption of **6a**. Then the solvent was removed *in vacuo*. The crude material was purified by flash column chromatography using an eluent of 5% EtOAc/CH_2Cl_2 to afford the desired material **7aa**. The enantioselectivity was determined by chiral HPLC.

Supplementary Table 8.

Entry	Lewis Acid (*x* mol%)	Solvent	Yield[a] (%)	e.e.[b] (%)
1	None	PhMe	34	88
2	$AlCl_3$ (10)	PhMe	29	86
3	$CeCl_3$ (10)	PhMe	22	85
4	CuCl (10)	PhMe	7	85
5	$CuCl_2$ (10)	PhMe	58	85
6	$FeCl_3$ (10)	PhMe	37	77
7	$InCl_3$ (10)	PhMe	48	75
8	$MgCl_2$ (10)	PhMe	18	86
9	$ZnCl_2$ (10)	PhMe	39	84
10	$ZnCl_2$ (30)	PhMe	42	74

[a]Isolated yields. [b]Enantiomeric excesses were determined by chiral HPLC analysis.

5.3.2 Concentration and Additive Screening

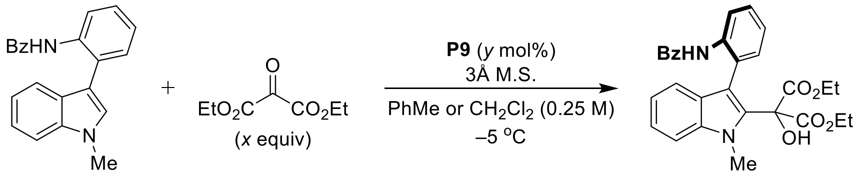

6a **2a** **7aa**

To an oven dried 4 mL vial equipped with a magnetic stir bar was added substrate **6a** (0.05 mmol, 1 equiv), **P9** (*y* mol%) and 3 Å molecular sieves (40 mg). Then, the solution of diethyl ketomalonate **2a** (*x* equiv) in PhMe or CH_2Cl_2 (0.2 mL, 0.25 M) was added. The vial was sealed with a Teflon cap and further secured with Parafilm M®. The reaction mixture was stirred at –5 ºC until complete consumption of **6a**. Then the solvent was removed *in vacuo*. The crude material was purified by flash column chromatography using an eluent of 5% EtOAc/CH_2Cl_2 to afford the desired material **7aa**. The enantioselectivity was determined by chiral HPLC.

Supplementary Table 9.

Entry	Solvent	**2a** (*x* equiv)	**P9** (*y* mol%)	Yield[a] (%)	e.e.[b] (%)
1[c]	PhMe	1.5	10	7	86
2	PhMe	1.5	20	34	88
3	CH_2Cl_2	1.5	10	15	90
4	CH_2Cl_2	5	10	8	89
5	CH_2Cl_2	10	10	10	89
6	CH_2Cl_2	15	10	39	89
7	CH_2Cl_2	15	20	45	89
8	CH_2Cl_2	15	40	65	89

[a]Isolated yields. [b]Enantiomeric excesses were determined by chiral HPLC analysis. [c]Optimized reaction condition of **3**.

6 Reaction Procedures for 3, 5 and 7

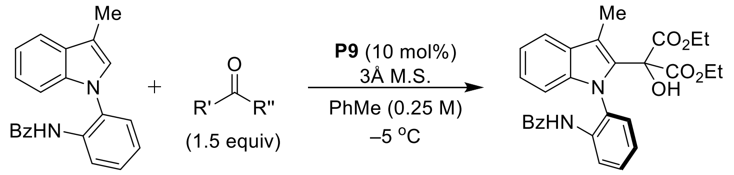

1 **2** **3**

Procedure 21: To an oven dried 4 mL vial equipped with a magnetic stir bar was added substrate **1** (0.05 mmol, 1 equiv), **P9** (0.005 mmol, 0.1 equiv) and 3 Å molecular sieves (40 mg). Then, the solution of **2** (0.075 mmol, 1.5 equiv) in PhMe (0.2 mL, 0.25 M) was added. The vial was sealed with a Teflon cap and further secured with Parafilm M®. The reaction mixture was stirred at –5 °C until complete consumption of **1**. Then the solvent was removed *in vacuo*. The crude material was purified by flash column chromatography using an eluent of 5% EtOAc/CH_2Cl_2 to afford the desired material **3**. The enantioselectivity was determined by chiral HPLC.

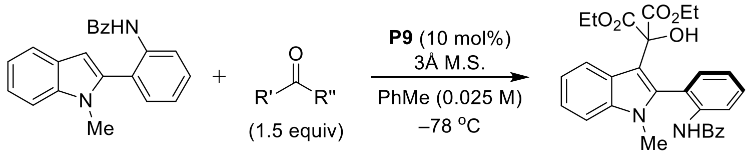

4 **2** **5**

Procedure 22: To an oven dried 20 mL vial equipped with a magnetic stir bar was added substrate **4** (0.05 mmol, 1 equiv), **P9** (0.005 mmol, 0.1 equiv) and 3 Å molecular sieves (400 mg). Then, the solution of **2** (0.075 mmol, 1.5 equiv) in PhMe (2 mL, 0.025 M) was added. The vial was sealed with a Teflon cap and further secured with Parafilm M®. The reaction mixture was stirred at –78 °C until complete consumption of **4**. Then the solvent was removed *in vacuo*. The crude material was purified by flash column chromatography using an eluent of 5% EtOAc/CH_2Cl_2 to afford the desired material **5**. The enantioselectivity was determined by chiral HPLC.

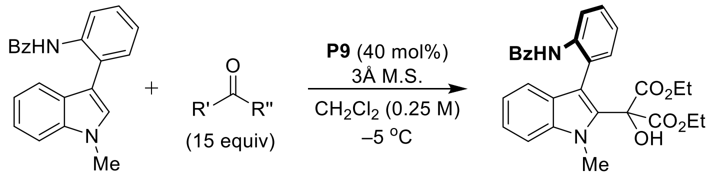

6 **2** **7**

Procedure 23: To an oven dried 4 mL vial equipped with a magnetic stir bar was added substrate **6** (0.05 mmol, 1 equiv), **P9** (0.02 mmol, 0.4 equiv) and 3 Å molecular sieves (40 mg). Then, the solution of **2** (0.75 mmol, 15 equiv) in CH_2Cl_2 (0.2 mL, 0.25 M) was added. The vial was sealed with a Teflon cap and further secured with Parafilm M®. The reaction mixture was stirred at –5 °C until complete consumption of **6**. Then the solvent was removed *in vacuo*. The crude material was purified by flash column chromatography using an eluent of 5% EtOAc/CH_2Cl_2 to afford the desired material **7**. The enantioselectivity was determined by chiral HPLC.

7 Characterization and Spectra of 3

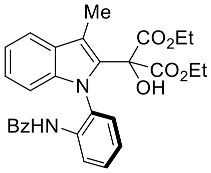

Diethyl 2-(1-(2-Benzamidophenyl)-3-methyl-1*H*-indol-2-yl)-2-hydroxymalonate (3aa) was synthesized by following Procedure 21 from **1a**. The crude material was purified by normal-phase column chromatography using an eluent of 5% EtOAc/CH_2Cl_2 to give **3aa** (99% yield).

^{1}H NMR (400 MHz, $CDCl_3$) δ 8.53 (dd, J = 8.3, 1.4 Hz, 1H), 7.92 (s, 1H), 7.64 (ddt, J = 7.2, 3.4, 1.7 Hz, 1H), 7.52 (ddd, J = 8.5, 7.6, 1.6 Hz, 1H), 7.44–7.36 (m, 3H), 7.33 (dd, J = 7.9, 1.6 Hz, 1H), 7.30–7.24 (m, 2H), 7.21 (td, J = 7.6, 1.5 Hz, 1H), 7.18–7.12 (m, 2H), 6.85–6.77 (m, 1H), 4.35–4.22 (m, 2H), 4.13 (ddq, J = 25.0, 10.8, 7.2 Hz, 2H), 3.97 (dq, J = 10.8, 7.1 Hz, 1H), 2.33 (s, 3H), 1.25 (t, J = 7.2 Hz, 3H), 1.15 (t, J = 7.2 Hz, 3H).
^{13}C NMR (100 MHz, $CDCl_3$) δ 169.4, 168.7, 165.5, 138.2, 137.0, 134.6, 131.8, 131.0, 130.9, 130.1, 128.7 (2C), 128.6, 128.0, 127.2 (2C), 124.4, 124.2, 122.1, 120.9, 119.4, 114.1, 110.9, 77.5, 63.8, 63.5, 13.9, 13.8, 9.5.
IR (FT-ATR, cm^{-1}, $CHCl_3$) ν_{max} 3946, 3926, 3899, 3865, 3838, 3799, 3776, 3730, 3699, 3626, 3595, 3413, 3163, 3059, 2985, 2931, 2873, 2627, 2441, 2360, 2333, 2299, 2268, 1967, 1894, 1867, 1809, 1739, 1674, 1593, 1523, 1450, 1362, 1304, 1257, 1215, 1103, 1026, 937, 895, 856, 748, 710.
HRMS (EI) *m/z*: $[M]^+$ Calcd for $C_{29}H_{28}N_2O_6$ 500.1947; found 500.1943.
Optical: $[a]^{20}_D$ = +55.1° (c = 2.19, $CHCl_3$, 96% e.e.)
HPLC (Chiralpak AD-H, ^{i}PrOH/*n*-hexane = 30/70, flow rate = 1.0 mL/min, λ = 254 nm) t_R = 25.1 min (major), 11.0 (minor)

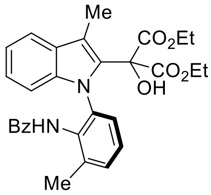

Diethyl 2-(1-(2-Benzamido-3-methylphenyl)-3-methyl-1*H*-indol-2-yl)-2-hydroxymalonate (3ba) was synthesized by following Procedure 21 from **1b**. The crude material was purified by normal-phase column chromatography using an eluent of 5% EtOAc/CH_2Cl_2 to give **3ba** (45% yield, 10 mol%; 99% yield, 20 mol%).

^{1}H NMR (400 MHz, $CDCl_3$) δ 8.26 (brs, 1H), 7.47 (dt, J = 7.7, 1.0 Hz, 1H), 7.44–7.39 (m, 3H), 7.39–7.34 (m, 1H), 7.31 (t, J = 7.7 Hz, 1H), 7.25 (ddd, J = 8.1, 6.2, 1.3 Hz, 2H), 7.20 (dd, J = 7.9, 1.6 Hz, 1H), 7.09 (ddd, J = 8.3, 7.0, 1.3 Hz, 1H), 7.04 (ddd, J = 8.0, 7.0, 1.2 Hz, 1H), 6.84 (dt, J = 8.2, 1.0 Hz, 1H), 4.33–4.14 (m, 3H), 4.02 (dq, J = 10.8, 7.2 Hz, 1H), 2.38 (s, 3H), 2.27 (s, 3H), 1.25 (q, J = 7.2 Hz, 6H).

^{13}C NMR (100 MHz, $CDCl_3$) δ 169.3, 169.1, 165.7, 138.7, 138.2, 135.9, 134.4, 134.2, 131.9, 131.4, 130.2, 128.6, 128.4 (2C), 128.0, 127.2 (2C), 126.8, 124.2, 120.3, 118.8, 113.3, 111.2, 77.7, 63.7, 63.7, 19.0, 13.9, 13.9, 9.4.
IR (FT-ATR, cm^{-1}, $CHCl_3$) ν_{max} 3950, 3926, 3899, 3838, 3799, 3780, 3730, 3703, 3626, 3599, 3545, 3336, 3163, 3059, 2985, 2927, 2873, 2360, 2333, 2299, 2268, 2017, 1990, 1967, 1894, 1871, 1743, 1666, 1585, 1512, 1489, 1466, 1365, 1265, 1223, 1146, 1099, 1026, 910, 860, 752, 710, 687.
HRMS (EI) *m/z*: $[M]^+$ Calcd for $C_{30}H_{30}N_2O_6$ 514.2104; found 514.2102.
Optical: $[a]^{20}_D$ = +44.1° (*c* = 2.52, $CHCl_3$, 96% e.e.)
HPLC (Chiralpak AD-H, ^{i}PrOH/*n*-hexane = 10/90, flow rate = 1.0 mL/min, λ = 254 nm) t_R = 35.8 min (major), 29.2 min (minor)

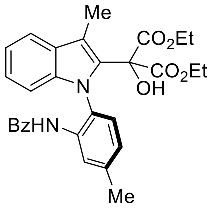

Diethyl 2-(1-(2-Benzamido-4-methylphenyl)-3-methyl-1*H*-indol-2-yl)-2-hydroxymalonate (3ca) was synthesized by following Procedure 21 from **1c**. The crude material was purified by normal-phase column chromatography using an eluent of 5% EtOAc/CH_2Cl_2 to give **3ca** (76% yield).

^{1}H NMR (400 MHz, $CDCl_3$) δ 8.35 (d, *J* = 2.0 Hz, 1H), 7.87 (brs, 1H), 7.68–7.60 (m, 1H), 7.44–7.35 (m, 3H), 7.31–7.24 (m, 2H), 7.19 (d, *J* = 8.0 Hz, 1H), 7.18–7.11 (m, 2H), 7.01 (dd, *J* = 8.0, 1.3 Hz, 1H), 6.84–6.77 (m, 1H), 4.32–4.23 (m, 2H), 4.19 (dq, *J* = 10.8, 7.1 Hz, 1H), 4.10 (dq, *J* = 10.8, 7.1 Hz, 1H), 3.99 (dq, *J* = 10.8, 7.2 Hz, 1H), 2.47 (s, 3H), 2.32 (s, 3H), 1.25 (t, *J* = 7.2 Hz, 3H), 1.16 (t, *J* = 7.1 Hz, 3H).
^{13}C NMR (100 MHz, $CDCl_3$) δ 169.5, 168.8, 165.4, 140.4, 138.3, 136.5, 134.7, 131.7, 131.1, 130.6, 128.7 (2C), 128.5, 127.1 (2C), 125.3, 125.0, 124.3, 122.6, 120.8, 119.3, 113.8, 110.9, 77.5, 63.7, 63.5, 21.9, 13.9, 13.8, 9.5.
IR (FT-ATR, cm^{-1}, $CHCl_3$) ν_{max} 3984, 3907, 3884, 3865, 3826, 3807, 3757, 3714, 3680, 3652, 3618, 3464, 3417, 3055, 2985, 2927, 2870, 2746, 2603, 2461, 2372, 2349, 2318, 2249, 2129, 1967, 1894, 1851, 1739, 1678, 1585, 1531, 1458, 1365, 1292, 1254, 1211, 1146, 1099, 1022, 930, 856, 818, 744, 717, 710, 667.
HRMS (EI) *m/z*: $[M]^+$ Calcd for $C_{30}H_{30}N_2O_6$ 514.2104; found 514.2100.
Optical: $[a]^{20}_D$ = +50.7° (*c* = 2.26, $CHCl_3$, 95% e.e.)
HPLC (Chiralpak IC, ^{i}PrOH/*n*-hexane = 30/70, flow rate = 1.0 mL/min, λ = 254 nm) t_R = 14.5 min (major), 11.2 min (minor)

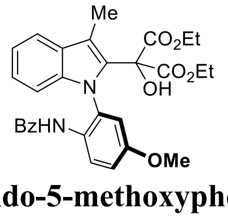

Diethyl 2-(1-(2-Benzamido-5-methoxyphenyl)-3-methyl-1*H*-indol-2-yl)-2-hydroxymalonate (3da) was synthesized by following Procedure 21 from **1d**. The

crude material was purified by normal-phase column chromatography using an eluent of 5% EtOAc/CH_2Cl_2 to give **3da** (52% yield, 10 mol%; 93% yield, 20 mol%).

^{1}H NMR (400 MHz, $CDCl_3$) δ 8.33 (d, *J* = 9.1 Hz, 1H), 7.84 (s, 1H), 7.65–7.59 (m, 1H), 7.38 (ddt, *J* = 7.1, 3.1, 1.5 Hz, 3H), 7.26 (dd, *J* = 8.9, 6.4 Hz, 2H), 7.15 (dt, *J* = 7.0, 3.5 Hz, 2H), 7.06 (dd, *J* = 9.1, 3.0 Hz, 1H), 6.91 (d, *J* = 2.9 Hz, 1H), 6.89–6.83 (m, 1H), 4.29 (dq, *J* = 10.8, 7.1 Hz, 1H), 4.14 (ddq, *J* = 25.1, 10.8, 7.1 Hz, 2H), 3.99 (dq, *J* = 10.9, 7.1 Hz, 1H), 3.80 (s, 3H), 2.32 (s, 3H), 1.25 (t, *J* = 7.2 Hz, 3H), 1.16 (t, *J* = 7.1 Hz, 3H).
^{13}C NMR (100 MHz, $CDCl_3$) δ 169.4, 168.8, 165.4, 156.3, 138.0, 134.8, 131.6, 130.9, 129.9, 129.5, 128.6 (2C), 128.5, 127.1 (2C), 124.4, 123.7, 120.8, 119.3, 116.3, 115.4, 114.1, 110.9, 77.6, 63.8, 63.6, 55.8, 13.9, 13.8, 9.5.
IR (FT-ATR, cm^{-1}, $CHCl_3$) ν_{max} 3953, 3930, 3907, 3869, 3842, 3826, 3807, 3757, 3714, 3680, 3653, 3618, 3460, 3417, 3059, 2985, 2939, 3873, 2839, 2627, 2372, 2349, 2318, 2233, 2067, 1990, 1894, 1739, 1666, 1601, 1520, 1454, 1365, 1300, 1257, 1215, 1099, 1026, 860, 814, 748, 710, 667.
HRMS (EI) *m*/*z*: $[M]^+$ Calcd for $C_{30}H_{30}N_2O_7$ 530.2053; found 530.2053.
Optical: $[a]^{20}_D$ = +46.9° (*c* = 2.29, $CHCl_3$, 99% e.e.)
HPLC (Chiralpak IC, ^{i}PrOH/*n*-hexane = 20/80, flow rate = 1.0 mL/min, λ = 254 nm) t_R = 34.5 min (major), 27.2 min (minor)

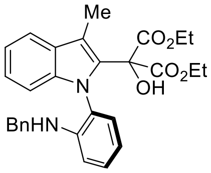

Diethyl 2-(1-(2-(Benzylamino)phenyl)-3-methyl-1*H*-indol-2-yl)-2-hydroxymalonate (3fa) was synthesized by following Procedure 21 from **1f**. The crude material was purified by normal-phase column chromatography using an eluent of 5% EtOAc/CH_2Cl_2 to give **3fa** (81% yield).

^{1}H NMR (400 MHz, $CDCl_3$) δ 7.67–7.59 (m, 1H), 7.32–7.15 (m, 8H), 7.13 (dd, *J* = 7.7, 1.6 Hz, 1H), 6.93–6.85 (m, 1H), 6.77–6.66 (m, 2H), 4.46–4.21 (m, 4H), 4.12 (ddq, *J* = 25.0, 10.8, 7.2 Hz, 2H), 3.98 (dq, *J* = 10.8, 7.1 Hz, 1H), 2.31 (s, 3H), 1.26 (t, *J* = 7.2 Hz, 3H), 1.21 (t, *J* = 7.2 Hz, 3H).
^{13}C NMR (100 MHz, $CDCl_3$) δ 169.7, 168.7, 145.9, 139.1, 137.5, 131.3, 131.2, 130.1, 128.7, 128.6 (2C), 127.3 (2C), 127.2, 123.5, 123.2, 120.3, 119.2, 116.8, 113.1, 111.9, 111.1, 77.6, 63.6, 63.2, 48.0, 14.0, 13.9, 9.6.
IR (FT-ATR, cm^{-1}, $CHCl_3$) ν_{max} 3946, 3926, 3899, 3869, 3838, 3799, 3780, 3730, 3703, 3626, 3599, 3479, 3425, 3182, 3163, 3059, 3028, 2981, 2927, 2870, 2627, 2360, 2333, 2299, 2268, 1963, 1890, 1736, 1604, 1516, 1454, 1362, 1265, 1211, 1161, 1111, 1026, 933, 860, 810, 744, 698.
HRMS (EI) *m*/*z*: $[M]^+$ Calcd for $C_{29}H_{30}N_2O_5$ 486.2155; found 486.2155.
Optical: $[a]^{20}_D$ = +16.4° (*c* = 1.22, $CHCl_3$, 99% e.e.)
HPLC (Chiralpak AD-H, ^{i}PrOH/*n*-hexane = 20/80, flow rate = 1.0 mL/min, λ = 254 nm) t_R = 9.3 min (major), 12.6 min (minor)

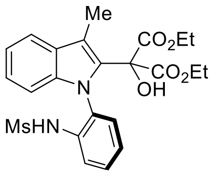

Diethyl 2-Hydroxy-2-(3-methyl-1-(2-(methylsulfonamido)phenyl)-1*H*-indol-2-yl)malonate (3ga) was synthesized by following Procedure 21 from **1g**. The crude material was purified by normal-phase column chromatography using an eluent of 5% EtOAc/CH_2Cl_2 to give **3ga** (99% yield).

^{1}H NMR (400 MHz, $CDCl_3$) δ 7.84–7.77 (m, 1H), 7.66–7.61 (m, 1H),7.51–7.43 (m, 1H), 7.24–7.20 (m, 2H), 7.20–7.13 (m, 2H), 6.85 (brs, 1H), 6.75–6.68 (m, 1H), 4.28 (s, 1H), 4.27–4.19 (m, 2H), 4.15 (dq, *J* = 10.8, 7.1 Hz, 1H), 4.06 (dq, *J* = 10.8, 7.2 Hz, 1H), 2.73 (s, 3H), 2.31 (s, 3H), 1.26 (dt, *J* = 10.0, 7.2 Hz, 6H).
^{13}C NMR (100 MHz, $CDCl_3$) δ 169.4, 168.5, 138.3, 136.2, 131.4, 130.6, 130.4, 128.72, 128.71, 124.7, 124.1, 120.9, 120.7, 119.6, 114.3, 110.4, 77.4, 63.8, 63.6, 40.1, 13.9 (2C), 9.5.
IR (FT-ATR, cm^{-1}, $CHCl_3$) ν_{max} 3934, 3907, 3880, 3842, 3826, 3807, 3780, 3757, 3718, 3680, 3656, 3629, 3595, 3456, 3275, 3113, 3051, 2981, 2927, 2858, 2480, 2372, 2349, 2310, 2083, 1728, 1608, 1504, 1462, 1373, 1335, 1273, 1230, 1153, 1103, 1030, 972, 922, 895, 864, 818, 756, 687.
HRMS (EI) *m*/*z*: $[M]^+$ Calcd for $C_{23}H_{26}N_2O_7S$ 474.1461; found 474.1460.
Optical: $[a]^{20}_D$ = +36.5° (*c* = 1.82, $CHCl_3$, 96% e.e.)
HPLC (Chiralpak AD, ^{i}PrOH/*n*-hexane = 10/90, flow rate = 1.0 mL/min, λ = 254 nm) t_R = 32.9 min (major), 46.1 min (minor)

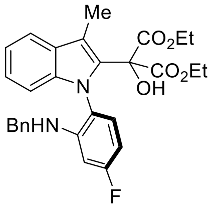

Diethyl 2-(1-(2-(Benzylamino)-4-fluorophenyl)-3-methyl-1*H*-indol-2-yl)-2-hydroxymalonate (3ha) was synthesized by following Procedure 21 from **1h**. The crude material was purified by normal-phase column chromatography using an eluent of 5% EtOAc/CH_2Cl_2 to give **3ha** (80% yield).

^{1}H NMR (400 MHz, $CDCl_3$) δ 7.62 (dd, *J* = 6.5, 1.8 Hz, 1H), 7.36–7.14 (m, 7H), 7.09 (dd, *J* = 8.5, 6.1 Hz, 1H), 6.87 (dd, *J* = 6.8, 1.6 Hz, 1H), 6.43–6.28 (m, 2H), 4.40–4.22 (m, 3H), 4.21–4.07 (m, 2H), 4.01 (dq, *J* = 10.7, 7.1 Hz, 1H), 2.30 (s, 3H), 1.28 (t, *J* = 7.1 Hz, 3H), 1.23 (t, *J* = 7.1 Hz, 3H).
^{13}C NMR (100 MHz, $CDCl_3$) δ 169.7, 168.6, 164.1 (d, *J* = 243.0 Hz, 1C), 147.7 (d, *J* = 12.0 Hz, 1C), 138.5, 137.6, 132.5 (d, *J* = 10.0 Hz, 1C), 131.2, 128.7 (2C), 128.6, 127.4, 127.2 (2C), 123.6, 120.4, 119.3, 118.9 (d, *J* = 3.0 Hz, 1C), 113.2, 110.9, 102.8 (d, *J* = 23.0 Hz, 1C), 98.6 (d, *J* = 27.0 Hz, 1C), 77.5, 63.7, 63.3, 47.8, 14.0, 13.9, 9.5.
IR (FT-ATR, cm^{-1}, $CHCl_3$) ν_{max} 3977, 3934, 3907, 3880, 3842, 3822, 3807, 3780, 3757, 3718, 3680, 3656, 3629, 3614, 3425, 3232, 3059, 3032, 2981, 2927, 2870, 2603, 2530, 2461, 2372, 2349, 2310, 2245, 2067, 1948, 1921, 1874, 1805, 1736, 1616, 1523, 1454, 1362, 1296, 1261, 1207, 1169, 1103, 1022, 957, 856, 829, 741, 698.

HRMS (EI) *m*/*z*: $[M]^+$ Calcd for $C_{29}H_{29}FN_2O_5$ 504.2061; found 504.2056.
Optical: $[\alpha]^{20}_D$ = +6.4° (*c* = 1.91, $CHCl_3$, 95% e.e.)
HPLC (Chiralpak AD-H, ^{i}PrOH/*n*-hexane = 10/90, flow rate = 1.0 mL/min, λ = 254 nm) t_R = 14.0 min (major), 12.6 min (minor)

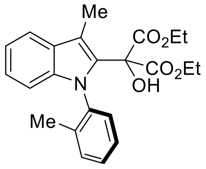

Diethyl 2-Hydroxy-2-(3-methyl-1-(*o*-tolyl)-1*H*-indol-2-yl)malonate (3ia) was synthesized by following Procedure 21 from **1i**. The crude material was purified by normal-phase column chromatography using an eluent of 5% EtOAc/CH_2Cl_2 to give **3ia** (50% yield).

^{1}H NMR (400 MHz, $CDCl_3$) δ 7.67–7.59 (m, 1H), 7.38–7.30 (m, 2H), 7.26–7.23 (m, 2H), 7.18–7.11 (m, 2H), 6.68 (ddt, *J* = 7.1, 4.3, 2.2 Hz, 1H), 4.28 (dq, *J* = 10.8, 7.2 Hz, 1H), 4.13 (ddq, *J* = 21.6, 10.8, 7.1 Hz, 2H), 4.01 (s, 1H), 3.96 (dt, *J* = 10.8, 7.2 Hz, 1H), 2.32 (s, 3H), 1.99 (s, 3H), 1.25 (dt, *J* = 17.8, 7.2 Hz, 6H).
^{13}C NMR (100 MHz, $CDCl_3$) δ 169.9, 169.0, 138.6, 137.8, 137.1, 130.7, 130.6, 130.5, 128.9, 128.2, 126.3, 123.3, 119.8, 119.1, 112.3, 110.6, 77.7, 63.5, 63.2, 17.9, 13.94, 13.90, 9.6.
IR (FT-ATR, cm^{-1}, $CHCl_3$) ν_{max} 3946, 3926, 3899, 3838, 3803, 3780, 3730, 3703, 3626, 3595, 3479, 3182, 3055, 2981, 2927, 2873, 2746, 2623, 2360, 2333, 2299, 2268, 1963, 1936, 1890, 1732, 1604, 1581, 1496, 1458, 1362, 1265, 1215, 1107, 1026, 937, 860, 810, 748, 687.
HRMS (EI) *m*/*z*: $[M]^+$ Calcd for $C_{23}H_{25}NO_5$ 395.1733; found 395.1732.
Optical: $[\alpha]^{20}_D$ = –22.9° (*c* = 0.34, $CHCl_3$, 84% e.e.)
HPLC (Chiralpak AD, ^{i}PrOH/*n*-hexane = 2/98, flow rate = 1.0 mL/min, λ = 254 nm) t_R = 26.4 min (major), 29.5 min (minor)

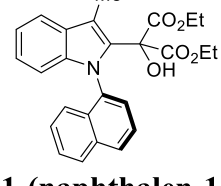

Diethyl 2-Hydroxy-2-(3-methyl-1-(naphthalen-1-yl)-1*H*-indol-2-yl)malonate (3ja) was synthesized by following Procedure 21 from **1j**. The crude material was purified by normal-phase column chromatography using an eluent of 5% EtOAc/CH_2Cl_2 to give **3ja** (71% yield).

^{1}H NMR (400 MHz, $CDCl_3$) δ 7.93 (dd, *J* = 7.8, 3.7 Hz, 2H), 7.68 (d, *J* = 7.9 Hz, 1H), 7.61 (dd, *J* = 7.3, 1.3 Hz, 1H), 7.57–7.44 (m, 2H), 7.37 (d, *J* = 3.6 Hz, 2H), 7.16 (ddd, *J* = 8.0, 6.9, 1.0 Hz, 1H), 7.06 (ddd, *J* = 8.2, 7.0, 1.2 Hz, 1H), 6.58 (d, *J* = 8.2 Hz, 1H), 4.38–4.11 (m, 2H), 4.08 (s, 1H), 3.69 (dq, *J* = 10.7, 7.1 Hz, 1H), 2.97 (dq, *J* = 10.8, 7.2 Hz, 1H), 2.37 (s, 3H), 1.24 (t, *J* = 7.1 Hz, 4H), 0.91 (t, *J* = 7.1 Hz, 3H).

^{13}C NMR (100 MHz, $CDCl_3$) δ 169.7, 168.9, 138.9, 134.6, 134.2, 131.9, 131.6, 129.2, 128.6, 128.25, 128.23, 126.8, 126.5, 125.5, 124.7, 123.4, 120.1, 119.2, 113.0, 111.3, 77.4, 63.5, 62.8, 14.0, 13.5, 9.6.
IR (FT-ATR, cm^{-1}, $CHCl_3$) ν_{max} 3977, 3930, 3907, 3865, 3842, 3807, 3757, 3737, 3680, 3656, 3618, 3475, 3232, 3055, 2981, 2930, 2873, 2750, 2611, 2530, 2418, 2372, 2345, 2318, 2129, 1932, 1886, 1736, 1597, 1577, 1512, 1458, 1400, 1365, 1265, 1211, 1412, 1099, 1018, 930, 860, 802, 771, 744, 714, 667.
HRMS (EI) *m*/*z*: $[M]^+$ Calcd for $C_{26}H_{25}NO_5$ 431.1733; found 431.1735.
Optical: $[a]^{20}_D$ = –28.0° (*c* = 0.21, $CHCl_3$, 27% e.e.)
HPLC (Chiralpak IC, ^{i}PrOH/*n*-hexane = 20/80, flow rate = 1.0 mL/min, λ = 254 nm) t_R = 20.4 min (major), 12.5 min (minor)

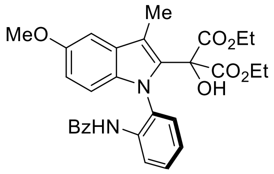

Diethyl 2-(1-(2-Benzamidophenyl)-5-methoxy-3-methyl-1*H*-indol-2-yl)-2-hydroxymalonate (3la) was synthesized by following Procedure 21 from **1l**. The crude material was purified by normal-phase column chromatography using an eluent of 5% EtOAc/CH_2Cl_2 to give **3la** (93% yield).

^{1}H NMR (400 MHz, $CDCl_3$) δ 8.52 (dd, *J* = 8.2, 1.4 Hz, 1H), 7.94 (brs, 1H), 7.50 (ddd, *J* = 8.6, 7.4, 1.6 Hz, 1H), 7.47–7.37 (m, 3H), 7.33–7.26 (m, 3H), 7.19 (td, *J* = 7.6, 1.4 Hz, 1H), 7.04 (d, *J* = 2.4 Hz, 1H), 6.80 (dd, *J* = 8.9, 2.4 Hz, 1H), 6.70 (d, *J* = 8.9 Hz, 1H), 4.25 (dq, *J* = 10.8, 7.3 Hz, 2H), 4.12 (ddq, *J* = 25.1, 10.8, 7.2 Hz, 2H), 3.96 (dq, *J* = 10.8, 7.1 Hz, 1H), 3.85 (s, 3H), 2.29 (s, 3H), 1.24 (t, *J* = 7.1 Hz, 3H), 1.15 (t, *J* = 7.2 Hz, 3H).
^{13}C NMR (100 MHz, $CDCl_3$) δ 169.4, 168.7, 165.5, 155.1, 137.0, 134.6, 133.5, 131.8, 131.6, 130.8, 130.0, 129.0, 128.7 (2C), 128.1, 127.2 (2C), 124.2, 122.0, 114.4, 113.8, 111.7, 101.1, 77.6, 63.7, 63.5, 56.1, 13.9, 13.8, 9.6.
IR (FT-ATR, cm^{-1}, $CHCl_3$) ν_{max} 3946, 3926, 3899, 3865, 3838, 3799, 3776, 3730, 3703, 3626, 3599, 3413, 3163, 3062, 2985, 2935, 2835, 2603, 2360, 2333, 2299, 2268, 1967, 1894, 1867, 1739, 1674, 1593, 1523, 1450, 1369, 1342, 1304, 1254, 1223, 1120, 1173, 1107, 1026, 949, 856, 837, 802, 756, 710, 663.
HRMS (EI) *m*/*z*: $[M]^+$ Calcd for $C_{30}H_{30}N_2O_7$ 530.2053; found 530.2054.
Optical: $[a]^{20}_D$ = +64.9° (*c* = 1.53, $CHCl_3$, 95% e.e.)
HPLC (Chiralpak AD-H, ^{i}PrOH/*n*-hexane = 20/80, flow rate = 1.0 mL/min, λ = 254 nm) t_R = 28.3 min (major), 22.3 min (minor)

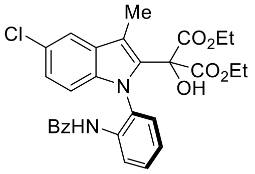

Diethyl 2-(1-(2-Benzamidophenyl)-5-chloro-3-methyl-1*H*-indol-2-yl)-2-hydroxymalonate (3ma) was synthesized by following Procedure 21 from **1m**. The

crude material was purified by normal-phase column chromatography using an eluent of 5% EtOAc/CH_2Cl_2 to give **3ma** (34% yield, 10 mol%; 70% yield, 20 mol%).

^{1}H NMR (400 MHz, $CDCl_3$) δ 8.47 (dd, J = 8.3, 1.4 Hz, 1H), 7.92 (s, 1H), 7.59 (d, J = 1.9 Hz, 1H), 7.53 (ddd, J = 8.5, 7.3, 1.7 Hz, 1H), 7.48–7.40 (m, 3H), 7.35–7.26 (m, 3H), 7.22 (td, J = 7.6, 1.4 Hz, 1H), 7.09 (dd, J = 8.7, 2.0 Hz, 1H), 6.71 (d, J = 8.7 Hz, 1H), 4.26 (dq, J = 10.8, 7.1 Hz, 2H), 4.13 (ddq, J = 27.0, 10.8, 7.1 Hz, 2H), 4.00 (dq, J = 10.8, 7.2 Hz, 1H), 2.28 (s, 3H), 1.25 (t, J = 7.2 Hz, 3H), 1.17 (t, J = 7.1 Hz, 3H).
^{13}C NMR (100 MHz, $CDCl_3$) δ 169.2, 168.5, 165.5, 136.9, 136.6, 134.5, 132.4, 131.9, 130.8, 130.3, 130.0, 128.8 (2C), 127.8, 127.2 (2C), 126.7, 124.7, 124.4, 122.6, 118.9, 113.6, 112.0, 77.5, 63.9, 63.7, 13.91, 13.86, 9.5.
IR (FT-ATR, cm^{-1}, $CHCl_3$) ν_{max} 3977, 3953, 3930, 3907, 3884, 3841, 3826, 3807, 3780, 3727, 3718, 3680, 3656, 3629, 3460, 3082, 2985, 2924, 2854, 2468, 2372, 2345, 2310, 2241, 1971, 1739, 1678, 1597, 1523, 1454, 1373, 1300, 1227, 1091, 1018, 856, 795, 717.
HRMS (EI) m/z: $[M]^+$ Calcd for $C_{29}H_{27}ClN_2O_6$ 534.1558; found 534.1560.
Optical: $[a]^{20}_D$ = +75.3° (c = 1.39, $CHCl_3$, 97% e.e.)
HPLC (Chiralpak AD-H, ^{i}PrOH/n-hexane = 10/90, flow rate = 1.0 mL/min, λ = 254 nm) t_R = 22..3 min (major), 15.7 min (minor)

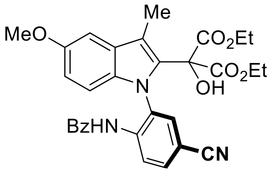

Diethyl 2-(1-(2-Benzamido-5-cyanophenyl)-5-methoxy-3-methyl-1*H*-indol-2-yl)-2-hydroxymalonate (3na) was synthesized by following Procedure 21 from **1n**. The crude material was purified by normal-phase column chromatography using an eluent of 5% EtOAc/CH_2Cl_2 to give **3na** (40% yield, 10 mol%; 99% yield, 20 mol%).

^{1}H NMR (400 MHz, $CDCl_3$) δ 8.77 (d, J = 8.7 Hz, 1H), 8.03 (brs, 1H), 7.77 (dd, J = 8.6, 2.0 Hz, 1H), 7.56 (d, J = 2.0 Hz, 1H), 7.49–7.41 (m, 3H), 7.36–7.28 (m, 2H), 7.06 (d, J = 2.4 Hz, 1H), 6.84 (dd, J = 8.9, 2.4 Hz, 1H), 6.65 (d, J = 8.9 Hz, 1H), 4.31 (dq, J = 10.8, 7.1 Hz, 1H), 4.25–4.10 (m, 3H), 4.05 (dq, J = 10.8, 7.1 Hz, 1H), 3.86 (s, 3H), 2.29 (s, 3H), 1.28 (t, J = 7.1 Hz, 3H), 1.18 (t, J = 7.2 Hz, 3H).
^{13}C NMR (100 MHz, $CDCl_3$) δ 169.3, 168.5, 165.6, 155.5, 141.4, 134.4, 133.83, 133.82, 133.3, 132.5, 131.3, 129.5, 128.9 (2C), 128.6, 127.3 (2C), 121.5, 118.0, 115.0, 114.9, 111.3, 107.0, 101.5, 77.5, 64.0, 63.6, 56.1, 14.0, 13.9, 9.6.
IR (FT-ATR, cm^{-1}, $CHCl_3$) ν_{max} 3957, 3930, 3907, 3869, 3842, 3826, 3807, 3780, 3757, 3714, 3680, 3653, 3618, 3410, 3062, 2981, 2931, 2584, 2372, 2349, 2318, 2229, 2060, 1971, 1851, 1739, 1685, 1581, 1512, 1466, 1365, 1304, 1250, 1227, 1211, 1165, 1103, 1026, 903, 837, 802, 756, 710, 663.
HRMS (EI) m/z: $[M]^+$ Calcd for $C_{31}H_{29}N_3O_7$ 555.2006; found 555.2004.
Optical: $[a]^{20}_D$ = +22.9° (c = 1.59, $CHCl_3$, 99% e.e.)
HPLC (Chiralpak IC, ^{i}PrOH/n-hexane = 40/60, flow rate = 1.0 mL/min, λ = 254 nm) t_R = 13.2 min (major), 26.6 min (minor)

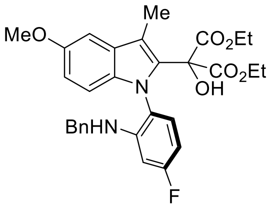

Diethyl 2-(1-(2-(Benzylamino)-4-fluorophenyl)-5-methoxy-3-methyl-1*H*-indol-2-yl)-2-hydroxymalonate (3oa) was synthesized by following Procedure 21 from **1o**. The crude material was purified by normal-phase column chromatography using an eluent of 5% EtOAc/CH_2Cl_2 to give **3oa** (83% yield).

^{1}H NMR (400 MHz, $CDCl_3$) δ 7.35–7.21 (m, 5H), 7.08 (dd, *J* = 8.5, 6.1 Hz, 1H), 7.04 (d, *J* = 2.4 Hz, 1H), 6.88 (dd, *J* = 8.9, 2.4 Hz, 1H), 6.78 (d, *J* = 8.9 Hz, 1H), 6.43–6.31 (m, 2H), 4.41 (brs, 1H), 4.37–4.23 (m, 3H), 4.16 (ddq, *J* = 20.4, 10.7, 7.1 Hz, 2H), 4.00 (dq, *J* = 10.8, 7.2 Hz, 1H), 3.89 (s, 3H), 2.27 (s, 3H), 1.29 (t, *J* = 7.2 Hz, 3H), 1.23 (t, *J* = 7.1 Hz, 3H).
^{13}C NMR (100 MHz, $CDCl_3$) δ 169.7, 168.6, 164.0 (d, *J* = 243.0 Hz, 1C), 154.8, 147.7 (d, *J* = 12.0 Hz, 1C), 138.5, 132.8, 132.4 (d, *J* = 11.0 Hz, 1C), 131.7, 128.9, 128.7 (2C), 127.4, 127.2 (2C), 119.0 (d, *J* = 2.0 Hz, 1C), 113.9, 112.8, 111.9, 102.8 (d, *J* = 23.0 Hz, 1C), 100.9, 98.5 (d, *J* = 27.0 Hz, 1C), 77.5, 63.7, 63.2, 56.1, 47.8, 14.0, 13.9, 9.7.
IR (FT-ATR, cm^{-1}, $CHCl_3$) ν_{max} 3953, 3930, 3907, 3884, 3845, 3826, 3807, 3780, 3761, 3718, 3683, 3656, 3629, 3695, 3572, 3464, 3398, 3367, 3302, 3271, 3232, 3066, 2954, 2927, 2862, 2372, 2345, 2314, 2060, 1952, 1739, 1624, 1593, 1512, 1454, 1369, 1234, 1173, 1119, 1065, 1034, 960, 856, 833, 791, 744, 706.
HRMS (EI) *m/z*: $[M]^+$ Calcd for $C_{30}H_{31}FN_2O_3$ 534.2166; found 534.2168.
Optical: $[a]^{20}_{D}$ = +7.3° (*c* = 2.37, $CHCl_3$, 93% e.e.)
HPLC (Chiralpak AD-H, ^{i}PrOH/*n*-hexane = 10/90, flow rate = 1.0 mL/min, λ = 254 nm) t_R = 21.0 min (major), 31.2 min (minor)

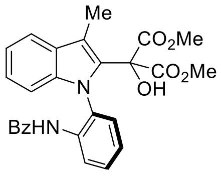

Dimethyl 2-(1-(2-Benzamidophenyl)-3-methyl-1*H*-indol-2-yl)-2-hydroxymalonate (3ab) was synthesized by following Procedure 21 from **1a**. The crude material was purified by normal-phase column chromatography using an eluent of 5% EtOAc/CH_2Cl_2 to give **3ab** (73% yield).

^{1}H NMR (400 MHz, $CDCl_3$) δ 8.56 (dd, *J* = 8.3, 1.4 Hz, 1H), 7.84 (brs, 1H), 7.70–7.61 (m, 1H), 7.53 (ddd, *J* = 8.6, 7.5, 1.6 Hz, 1H), 7.49–7.32 (m, 4H), 7.32–7.05 (m, 5H), 6.87–6.74 (m, 1H), 3.77 (s, 3H), 3.61 (s, 3H), 2.31 (s, 3H).
^{13}C NMR (100 MHz, $CDCl_3$) δ 169.8, 169.2, 165.5, 138.0, 136.9, 134.6, 131.9, 131.0, 130.8, 130.2, 128.7 (2C), 128.5, 127.5, 127.1 (2C), 124.6, 124.3, 122.0, 121.0, 119.5, 114.1, 110.8, 77.3, 54.3, 54.2, 9.3.
IR (FT-ATR, cm^{-1}, $CHCl_3$) ν_{max} 3946, 3926, 3899, 3865, 3838, 3803, 3780, 3730, 3699, 3626, 3599, 3568, 3460, 3413, 3163, 3116, 3059, 3016, 2951, 2927, 2850, 2360, 2337, 2295, 2268, 1967, 1894, 1867, 1743, 1670, 1593, 1523, 1450, 1358, 1304, 1254, 1223, 1115, 1034, 937, 829, 795, 748, 710.

HRMS (EI) *m/z*: $[M]^+$ Calcd for $C_{27}H_{24}N_2O_6$ 472.1634; found 472.1630.
Optical: $[a]^{20}_D$ = +62.8° (*c* = 1.81, $CHCl_3$, 91% e.e.)
HPLC (Chiralpak AD, ^{i}PrOH/*n*-hexane = 30/70, flow rate = 1.0 mL/min, λ = 254 nm) t_R = 14.3 min (major), 9.7 min (minor)

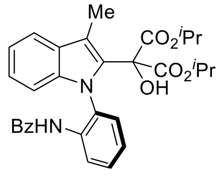

Diisopropyl 2-(1-(2-Benzamidophenyl)-3-methyl-1*H*-indol-2-yl)-2-hydroxymalonate (3ac) was synthesized by following Procedure 21 from **1a**. The crude material was purified by normal-phase column chromatography using an eluent of 5% EtOAc/CH_2Cl_2 to give **3ac** (98% yield).

^{1}H NMR (400 MHz, $CDCl_3$) δ 8.49 (dd, *J* = 8.3, 1.4 Hz, 1H), 8.02 (brs, 1H), 7.65–7.59 (m, 1H), 7.54–7.44 (m, 3H), 7.43–7.36 (m, 1H), 7.33–7.22 (m, 3H), 7.21–7.09 (m, 3H), 6.82–6.75 (m, 1H), 5.01 (h, *J* = 6.3 Hz, 1H), 4.92 (h, *J* = 6.3 Hz, 1H), 4.18 (s, 1H), 2.35 (s, 3H), 1.26 (d, *J* = 6.3 Hz, 3H), 1.23 (d, *J* = 6.2 Hz, 3H), 1.20 (d, *J* = 6.3 Hz, 3H), 1.13 (d, *J* = 6.2 Hz, 3H).
^{13}C NMR (100 MHz, $CDCl_3$) δ 169.2, 168.1, 165.6, 138.6, 137.0, 134.8, 131.7, 131.2, 130.5, 129.8, 128.9, 128.8, 128.6 (2C), 127.2 (2C), 124.20, 124.15, 122.2, 120.7, 119.2, 113.9, 110.9, 77.9, 72.2, 71.6, 21.6, 21.48 (2C), 21.45, 9.8.
IR (FT-ATR, cm^{-1}, $CHCl_3$) ν_{max} 3946, 3926, 3899, 3838, 3799, 3776, 3730, 3703, 3626, 3595, 3568, 3413, 3163, 3059, 2981, 2931, 2877, 2360, 2333, 2299, 2268, 1967, 1921, 1894, 1867, 1813, 1732, 1674, 1593, 1523, 1454, 1534, 1308, 1261, 1219, 1138, 1099, 1026, 991, 933, 906, 829, 748, 710.
HRMS (EI) *m/z*: $[M]^+$ Calcd for $C_{31}H_{32}N_2O_6$ 528.2260; found 528.2258.
Optical: $[a]^{20}_D$ = +56.5° (*c* = 2.37, $CHCl_3$, 88% e.e.)
HPLC (Chiralpak AD, ^{i}PrOH/*n*-hexane = 30/70, flow rate = 1.0 mL/min, λ = 254 nm) t_R = 11.3 min (major), 7.4 min (minor)

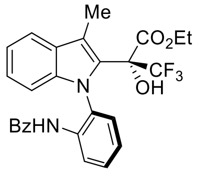

Ethyl 2-(1-(2-Benzamidophenyl)-3-methyl-1*H*-indol-2-yl)-3,3,3-trifluoro-2-hydroxypropanoate (3ad) was synthesized by following Procedure 21 from **1a**. The crude material was purified by normal-phase column chromatography using an eluent of 5% EtOAc/CH_2Cl_2 to give **3ad** (17% yield, >20:1 d.r., 10 mol%; 50% yield, >20:1 d.r., 20 mol%, rt).

^{1}H NMR (400 MHz, $CDCl_3$) Major diastereomer: δ 8.52 (dd, *J* = 8.3, 1.4 Hz, 1H), 7.75–7.69 (m, 1H), 7.54 (td, *J* = 7.8, 1.6 Hz, 1H), 7.45–7.38 (m, 2H), 7.35 (brs, 1H), 7.31–7.16 (m, 7H), 6.80–6.74 (m, 1H), 4.32 (s, 1H), 4.03 (dq, *J* = 10.8, 7.1 Hz, 1H), 3.71 (dq, *J* = 10.8, 7.1 Hz, 1H), 2.50 (d, *J* = 1.5 Hz, 3H), 0.99 (t, *J* = 7.2 Hz, 3H).
^{13}C NMR (100 MHz, $CDCl_3$) Major diastereomer: δ 167.2, 165.7, 154.7, 138.6, 136.2, 134.2, 132.1, 131.0, 130.4, 128.9, 128.8 (2C), 127.4, 126.9 (2C), 125.1, 125.0, 123.0

(q, J = 335.8 Hz, 1C), 121.9, 121.3, 119.9, 116.2, 110.7, 77.1 (q, J = 31.4 Hz, 1C), 64.3, 13.5, 9.8 (q, J = 4.3 Hz, 1C).
IR (FT-ATR, cm^{-1}, $CHCl_3$) ν_{max} 3950, 3926, 3899, 3865, 3838, 3803, 3780, 3730, 3699, 3626, 3595, 3568, 3545, 3525, 3410, 3232, 3163, 3066, 3012, 2924, 2873, 2360, 2333, 2299, 2268, 2017, 1990, 1967, 1921, 1894, 1871, 1747, 1658, 1597, 1527, 1465, 1354, 1308, 1281, 1250, 1184, 1138, 1072, 1030, 968, 899, 856, 752, 717, 683.
HRMS (EI) *m*/*z*: $[M]^+$ Calcd for $C_{27}H_{23}F_3N_2O_4$ 496.1610; found 496.1608.
Optical: $[a]^{20}_D$ = +91.1° (c = 1.13, $CHCl_3$, >20:1 d.r., 99% e.e.)
HPLC (Chiralpak AD-H, ^{i}PrOH/*n*-hexane = 5/95, flow rate = 1.0 mL/min, λ = 254 nm) t_R = 34.3 min (major diastereomer), 22.8 min (minor enantiomer)

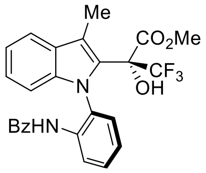

Methyl 2-(1-(2-Benzamidophenyl)-3-methyl-1*H*-indol-2-yl)-3,3,3-trifluoro-2-hydroxypropanoate (3ae) was synthesized by following Procedure 21 from **1a**. The crude material was purified by normal-phase column chromatography using an eluent of 5% EtOAc/CH_2Cl_2 to give **3ae** (6% yield, >20:1 d.r., 10 mol%; 49% yield, >20:1 d.r., 20 mol%, rt).

^{1}H NMR (400 MHz, $CDCl_3$) Major diastereomer: δ 8.47 (dd, J = 8.3, 1.4 Hz, 1H), 7.71 (ddt, J = 7.2, 3.6, 1.8 Hz, 1H), 7.58–7.49 (m, 1H), 7.42 (ddt, J = 8.6, 7.0, 1.6 Hz, 2H), 7.36 (s, 1H), 7.32–7.16 (m, 7H), 6.80–6.73 (m, 1H), 4.48 (s, 1H), 3.43 (s, 3H), 2.46 (d, J = 1.4 Hz, 3H).
^{13}C NMR (100 MHz, $CDCl_3$) Major diastereomer: δ 167.6, 165.9, 138.6, 136.1, 134.0, 132.2, 130.9, 130.3, 129.1, 128.8 (3C), 127.3, 126.9 (2C), 125.2, 125.1, 123.2 (q, J = 284.0 Hz, 1C), 122.2, 121.3, 119.9, 116.1, 110.7, 77.1 (q, J = 32.0 Hz, 1C), 54.3, 9.7 (q, J = 3.5 Hz, 1C).
IR (FT-ATR, cm^{-1}, $CHCl_3$) ν_{max} 3727, 3624, 3400, 3058, 2964, 2931, 2874, 2371, 2339, 2328, 1668, 1608, 1579, 1543, 1512, 1493, 1469, 1441, 1389, 1369, 1306, 1250, 1218, 1200, 1159, 1137, 1102, 1073, 1042, 1027, 1014, 977, 943, 892, 795, 741, 703, 666.
HRMS (EI) *m*/*z*: $[M]^+$ Calcd for $C_{26}H_{21}F_3N_2O_4$ 482.1453; found 482.1449.
Optical: $[a]^{20}_D$ = +98.8° (c = 1.22, $CHCl_3$, >20:1 d.r., 86% e.e.)
HPLC (Chiralpak OD-H, ^{i}PrOH/*n*-hexane = 3/97, flow rate = 1.0 mL/min, λ = 254 nm) t_R = 36.8 min (major diastereomer), 17.8 min (minor enantiomer)

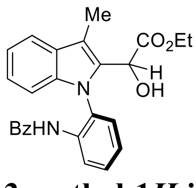

Ethyl 2-(1-(2-Benzamidophenyl)-3-methyl-1*H*-indol-2-yl)-2-hydroxyacetate (3af) was synthesized by following Procedure 21 from **1a**. The crude material was purified by normal-phase column chromatography using an eluent of 5% EtOAc/CH_2Cl_2 to give **3af** (13% yield, 1.2:1 d.r., 10 mol%; 41% yield, 1.3:1 d.r., 20 mol%, rt).

^{1}H NMR (400 MHz, $CDCl_3$) Major diastereomer: δ 8.63 (dd, *J* = 8.3, 1.4 Hz, 1H), 7.68 (tdd, *J* = 7.6, 4.1, 2.4 Hz, 1H), 7.57 (ddd, *J* = 8.5, 7.5, 1.7 Hz, 1H), 7.52 (dd, *J* = 7.9, 1.6 Hz, 1H), 7.46 (s, 1H), 7.43–7.33 (m, 2H), 7.32–7.11 (m, 6H), 6.99–6.92 (m, 1H), 5.23 (d, *J* = 3.2 Hz, 1H), 4.13 (dq, *J* = 10.8, 7.2 Hz, 1H), 4.08–3.86 (m, 1H), 3.50–3.35 (m, 1H), 2.42 (s, 3H), 1.07 (t, *J* = 7.1 Hz, 3H). Minor diastereomer: δ 8.57 (dd, *J* = 8.3, 1.4 Hz, 1H), 8.00 (s, 1H), 7.68 (tdd, *J* = 7.6, 4.1, 2.4 Hz, 1H), 7.57 (ddd, *J* = 8.5, 7.4, 1.7 Hz, 1H), 7.43–7.33 (m, 2H), 7.32–7.11 (m, 7H), 6.91–6.83 (m, 1H), 5.30 (d, *J* = 2.5 Hz, 1H), 4.08–3.86 (m, 2H), 3.50–3.35 (m, 1H), 2.44 (s, 3H), 1.04 (t, *J* = 7.2 Hz, 3H).
^{13}C NMR (100 MHz, $CDCl_3$) Major diastereomer: δ 172.3, 165.3, 137.3, 136.6, 134.3, 132.4, 131.9, 130.7, 130.4, 128.7 (2C), 128.6, 127.0 (2C), 126.5, 124.6, 124.1, 121.7, 120.8, 119.6, 113.3, 110.7, 65.2, 63.0, 14.0, 8.8. Minor diastereomer: δ 172.5, 165.4, 138.3, 137.1, 134.5, 132.1, 131.9, 130.5, 130.2, 128.7 (2C), 128.5, 127.4, 127.1 (2C), 124.6, 124.0, 122.6, 120.7, 119.4, 113.7, 110.7, 65.2, 62.9, 14.0, 8.9.
IR (FT-ATR, cm^{-1}, $CHCl_3$) ν_{max} 3412, 2370, 2357, 2338, 2329, 2296, 1735, 1668, 1592, 1523, 1497, 1456, 1364, 1306, 1216, 1159, 1135, 1088, 1025, 957, 894, 863, 795, 774, 755, 741, 707, 691, 667.
HRMS (EI) *m*/*z*: $[M]^+$ Calcd for $C_{26}H_{24}N_2O_4$ 428.1736; found 428.1736.
Optical: $[a]^{20}_D$ = +11.8° (*c* = 0.86, $CHCl_3$, 1.3:1 d.r., 72% e.e.)
HPLC (Chiralpak AD-H, ^{i}PrOH/*n*-hexane = 20/80, flow rate = 1.0 mL/min, λ = 254 nm) t_R = 12.4 min (major diastereomer), 16.7 min (minor enantiomer)

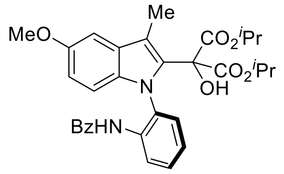

Diisopropyl 2-(1-(2-Benzamidophenyl)-5-methoxy-3-methyl-1*H*-indol-2-yl)-2-hydroxymalonate (3lc) was synthesized by following Procedure 21 from **1l**. The crude material was purified by normal-phase column chromatography using an eluent of 5% EtOAc/CH_2Cl_2 to give **3lc** (97% yield).

^{1}H NMR (400 MHz, $CDCl_3$) δ 8.48 (dd, *J* = 8.2, 1.5 Hz, 1H), 8.04 (s, 1H), 7.55–7.45 (m, 3H), 7.44–7.37 (m, 1H), 7.30 (dd, *J* = 8.3, 7.0 Hz, 2H), 7.22 (dd, *J* = 7.9, 1.7 Hz, 1H), 7.16 (td, *J* = 7.6, 1.4 Hz, 1H), 7.02 (d, *J* = 2.4 Hz, 1H), 6.77 (dd, *J* = 8.9, 2.4 Hz, 1H), 6.68 (d, *J* = 8.9 Hz, 1H), 4.96 (dp, *J* = 27.9, 6.3 Hz, 2H), 4.16 (s, 1H), 3.84 (s, 3H), 2.31 (s, 3H), 1.30 (d, *J* = 6.3 Hz, 3H), 1.28–1.23 (m, 3H), 1.23–1.17 (m, 3H), 1.13 (d, *J* = 6.2 Hz, 3H).
^{13}C NMR (100 MHz, $CDCl_3$) δ 169.2, 168.2, 165.6, 155.0, 136.9, 134.8, 133.9, 131.8, 131.7, 130.4, 129.7, 129.12, 129.06, 128.6 (2C), 127.3 (2C), 124.2, 122.1, 114.1, 113.6, 111.8, 101.1, 77.9, 72.2, 71.5, 56.0, 21.60, 21.55, 21.48, 21.45, 9.9.
IR (FT-ATR, cm^{-1}, $CHCl_3$) ν_{max} 3946, 3926, 3899, 3865, 3838, 3803, 3780, 3730, 3699, 3626, 3595, 3568, 3460, 3413, 3163, 3116, 3066, 2981, 2931, 2360, 2333, 2295, 2268, 1967, 1894, 1867, 1847, 1732, 1674, 1593, 1527, 1454, 1377, 1342, 1261, 1230, 1200, 1176, 1134, 1099, 1030, 995, 945, 906, 829, 802, 756, 710, 683.
HRMS (EI) *m*/*z*: $[M]^+$ Calcd for $C_{32}H_{34}N_2O_7$ 558.2366; found 558.2366.
Optical: $[a]^{20}_D$ = +43.5° (*c* = 2.37, $CHCl_3$, 75% e.e.)
HPLC (Chiralpak AD-H, ^{i}PrOH/*n*-hexane = 20/80, flow rate = 1.0 mL/min, λ = 254 nm) t_R = 18.7 min (major), 13.8 min (minor)

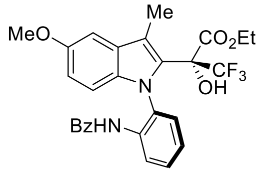

Ethyl 2-(1-(2-Benzamidophenyl)-5-methoxy-3-methyl-1*H*-indol-2-yl)-3,3,3-trifluoro-2-hydroxypropanoate (3ld) was synthesized by following Procedure 21 from **1l**. The crude material was purified by normal-phase column chromatography using an eluent of 5% EtOAc/CH_2Cl_2 to give **3ld** (97% yield, >20:1 d.r.).

^{1}H NMR (400 MHz, $CDCl_3$) Major diastereomer: δ 8.50 (dd, J = 8.3, 1.4 Hz, 1H), 7.51 (td, J = 7.8, 1.7 Hz, 1H), 7.48–7.38 (m, 2H), 7.35 (dd, J = 7.9, 1.6 Hz, 1H), 7.32–7.20 (m, 5H), 7.10 (d, J = 2.5 Hz, 1H), 6.85 (dd, J = 8.9, 2.5 Hz, 1H), 6.66 (d, J = 8.8 Hz, 1H), 4.01 (dq, J = 10.7, 7.2 Hz, 1H), 3.87 (s, 3H), 3.69 (dq, J = 10.7, 7.2 Hz, 1H), 2.46 (s, 3H), 0.99 (t, J = 7.1 Hz, 3H).
^{13}C NMR (100 MHz, $CDCl_3$) Major diastereomer: δ 167.2, 165.7, 136.2, 134.2, 134.0, 132.1, 130.7, 130.2, 129.3, 129.1, 128.8 (2C), 127.9, 126.9 (2C), 124.9, 123.3 (q, J = 286.0 Hz, 1C), 121.8, 116.0, 115.3, 111.7, 101.3, 77.20 (q, J = 32.0 Hz, 1C), 64.2, 56.1, 13.5, 9.91 (q, J = 4.0 Hz, 1C).
IR (FT-ATR, cm^{-1}, $CHCl_3$) ν_{max} 3950, 3926, 3899, 3869, 3838, 3803, 3780, 3730, 3699, 3626, 3595, 3545, 3525, 3406, 3244, 3163, 3070, 3012, 2927, 2850, 2360, 2333, 2295, 2268, 1990, 1967, 1917, 1867, 1747, 1658, 1597, 1527, 1454, 1365, 1308, 1281, 1227, 1173, 1138, 1068, 1026, 968, 856, 827, 802, 748, 710, 663.
HRMS (EI) m/z: $[M]^+$ Calcd for $C_{28}H_{25}F_3N_2O_5$ 526.1716; found 526.1714.
Optical: $[a]^{20}_D$ = +97.9° (c = 2.08, $CHCl_3$, >20:1 d.r., 95% e.e.)
HPLC (Chiralpak OD-H, ^{i}PrOH/n-hexane = 1/99, flow rate = 1.0 mL/min, λ = 254 nm) t_R = 150.9 min (major diastereomer), 67.2 min (minor enantiomer)

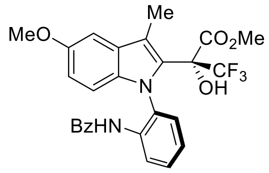

Methyl 2-(1-(2-Benzamidophenyl)-5-methoxy-3-methyl-1*H*-indol-2-yl)-3,3,3-trifluoro-2-hydroxypropanoate (3le) was synthesized by following Procedure 21 from **1l**. The crude material was purified by normal-phase column chromatography using an eluent of 5% EtOAc/CH_2Cl_2 to give **3le** (37% yield, >20:1 d.r., 10 mol%; 99% yield, >20:1 d.r., 20 mol%, rt).

^{1}H NMR (400 MHz, $CDCl_3$) Major diastereomer: δ 8.42 (dd, J = 8.2, 1.4 Hz, 1H), 7.47 (ddd, J = 8.6, 7.4, 1.6 Hz, 1H), 7.43 (s, 1H), 7.42–7.35 (m, 1H), 7.31 (dd, J = 7.9, 1.6 Hz, 1H), 7.28–7.19 (m, 5H), 7.05 (d, J = 2.4 Hz, 1H), 6.81 (dd, J = 8.9, 2.4 Hz, 1H), 6.62 (d, J = 8.9 Hz, 1H), 4.50 (s, 1H), 3.83 (s, 3H), 3.38 (s, 3H), 2.39 (s, 3H).
^{13}C NMR (100 MHz, $CDCl_3$) Major diastereomer: δ 167.6, 165.9, 155.3, 136.1, 134.0, 134.0, 132.1, 130.6, 130.2, 129.4, 129.2, 128.8 (2C), 127.8, 126.9 (2C), 125.1, 123.2 (q, J = 284.0 Hz, 1C), 122.1, 115.9, 115.3, 111.7, 101.2, 77.2 (q, J = 31.5 Hz, 1C), 56.1, 54.2, 9.8 (q, J = 3.5 Hz, 1C).
IR (FT-ATR, cm^{-1}, $CHCl_3$) ν_{max} 3753, 3713, 3651, 3612, 3411, 2953, 2377, 2359, 2352, 2331, 2320, 1752, 1656, 1593, 1523, 1497, 1477, 1454, 1361, 1308, 1285, 1225, 1171,

1136, 1103, 1075, 1028, 1001, 980, 949, 930, 907, 881, 834, 800,768, 754, 745, 707, 691, 666.
HRMS (EI) *m/z*: $[M]^+$ Calcd for $C_{27}H_{23}F_3N_2O_5$ 512.1559; found 512.1558.
Optical: $[a]^{20}_D$ = +90.2° (*c* = 2.49, $CHCl_3$, >20:1 d.r., 87% e.e.)
HPLC (Chiralpak OD-H, ^{i}PrOH/*n*-hexane = 3/97, flow rate = 1.0 mL/min, λ = 254 nm) t_R = 60.0 min (major diastereomer), 26.7 min (minor enantiomer)

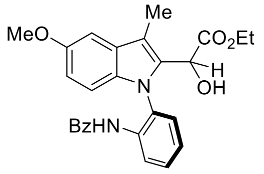

Ethyl 2-(1-(2-Benzamidophenyl)-5-methoxy-3-methyl-1*H*-indol-2-yl)-2-hydroxyacetate (3lf) was synthesized by following Procedure 21 from **1l**. The crude material was purified by normal-phase column chromatography using an eluent of 5% EtOAc/CH_2Cl_2 to give **3lf** (34% yield, 1.1:1 d.r., 10 mol%; 79% yield, 1.2:1 d.r., 20 mol%, rt).

^{1}H NMR (400 MHz, $CDCl_3$) Major diastereomer: δ 8.62 (dd, *J* = 8.3, 1.4 Hz, 1H), 7.55 (ddd, *J* = 8.5, 7.5, 1.7 Hz, 1H), 7.52–7.46 (m, 2H), 7.44–7.36 (m, 2H), 7.32–7.15 (m, 4H), 7.10–7.05 (m, 1H), 6.87–6.75 (m, 2H), 5.19 (d, *J* = 2.4 Hz, 1H), 4.13 (dq, *J* = 10.8, 7.1 Hz, 1H), 4.06–3.90 (m, 1H), 3.88 (s, 3H), 3.44 (d, *J* = 3.2 Hz, 1H), 2.38 (s, 3H), 1.08 (t, *J* = 7.1 Hz, 3H). Minor diastereomer: δ 8.56 (dd, *J* = 8.3, 1.3 Hz, 1H), 8.01 (s, 1H), 7.55 (ddd, *J* = 8.5, 7.5, 1.7 Hz, 1H), 7.44–7.36 (m, 2H), 7.33–7.14 (m, 6H), 7.11–7.04 (m, 1H), 6.88–6.74 (m, 2H), 5.25 (d, *J* = 1.9 Hz, 1H), 4.06–3.89 (m, 2H), 3.87 (s, 3H), 3.44 (d, *J* = 3.2 Hz, 1H), 2.40 (s, 3H), 1.03 (t, *J* = 7.1 Hz, 3H).
^{13}C NMR (100 MHz, $CDCl_3$) Major diastereomer: δ 172.3, 165.4, 155.1, 136.7, 134.3, 133.1, 132.5, 131.9, 130.7, 130.3, 129.0, 128.7 (2C), 127.0 (2C), 126.7, 124.6, 121.7, 114.1, 112.8, 111.6, 101.3, 65.2, 62.9, 56.1, 14.0, 8.9. Minor diastereomer: δ 172.5, 165.3, 155.0, 137.1, 134.5, 133.6, 132.8, 131.9, 130.4, 130.2, 128.9, 128.7 (2C), 127.4, 127.1 (2C), 124.6, 122.2, 114.0, 113.2, 111.6, 101.2, 65.3, 62.9, 56.1, 14.0, 9.0.
IR (FT-ATR, cm^{-1}, $CHCl_3$) ν_{max} 3753, 3713, 3651, 3612, 3411, 2953, 2377, 2359, 2352, 2331, 2321, 1752, 1656, 1593, 1523, 1497, 1477, 1454, 1361, 1308, 1285, 1225, 1171, 1136, 1103, 1075, 1028, 1001, 980, 949, 930, 907, 881, 834, 800, 768, 754, 745, 707, 691, 666.
HRMS (EI) *m/z*: $[M]^+$ Calcd for $C_{27}H_{26}N_2O_5$ 458.1842; found 458.1838.
Optical: $[a]^{20}_D$ = +4.50° (*c* = 1.16, $CHCl_3$, 1.2:1 d.r., 74% e.e.)
HPLC (Chiralpak AD-H, ^{i}PrOH/*n*-hexane = 20/80, flow rate = 1.0 mL/min, λ = 254 nm) t_R = 12.8 min (major diastereomer), 41.3 min (minor enantiomer)

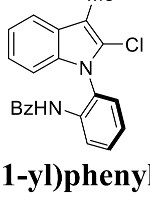

***N*-(2-(2-Chloro-3-methyl-1*H*-indol-1-yl)phenyl)benzamide (3aS39)** was synthesized by following Procedure 21 from **1a**. The crude material was purified by normal-phase column chromatography using an eluent of 25% EtOAc/Hx to give **3aS39** (32% yield, 3% e.e.).

^{1}H NMR (400 MHz, $CDCl_3$) δ 8.69 (dd, *J* = 8.3, 1.4 Hz, 1H), 7.62–7.52 (m, 2H), 7.39 (tt, *J* = 5.9, 2.8 Hz, 2H), 7.33–7.24 (m, 5H), 7.24–7.14 (m, 3H), 6.99–6.93 (m, 1H), 2.36 (s, 3H).
^{13}C NMR (100 MHz, $CDCl_3$) δ 165.3, 136.31, 136.25, 134.5, 132.0, 130.3, 129.8, 128.8 (2C), 128.1, 126.9 (2C), 125.7, 124.6, 123.8, 123.5, 121.5, 121.3, 118.9, 110.5, 110.2, 9.0.
IR (FT-ATR, cm^{-1}, $CHCl_3$) ν_{max} 3753, 3713, 3651, 3612, 3411, 2953, 2377, 2359, 2352, 2331, 2321, 1752, 1656, 1593, 1523, 1497, 1477, 1454, 1361, 1308, 1285, 1225, 1171, 1136, 1103, 1075, 1028, 1001, 980, 949, 930, 907, 880, 834, 800, 768, 754, 745, 707, 691, 666.
HRMS (EI) *m*/*z*: $[M]^+$ Calcd for $C_{22}H_{17}ClN_2O$ 360.1029; found 360.1030.
HPLC (Chiralpak AD-H, ^{i}PrOH/*n*-hexane = 10/90, flow rate = 1.0 mL/min, λ = 254 nm) t_R = 7.5 min (major), 8.6 min (minor)

7.1 NMR Spectra of 3

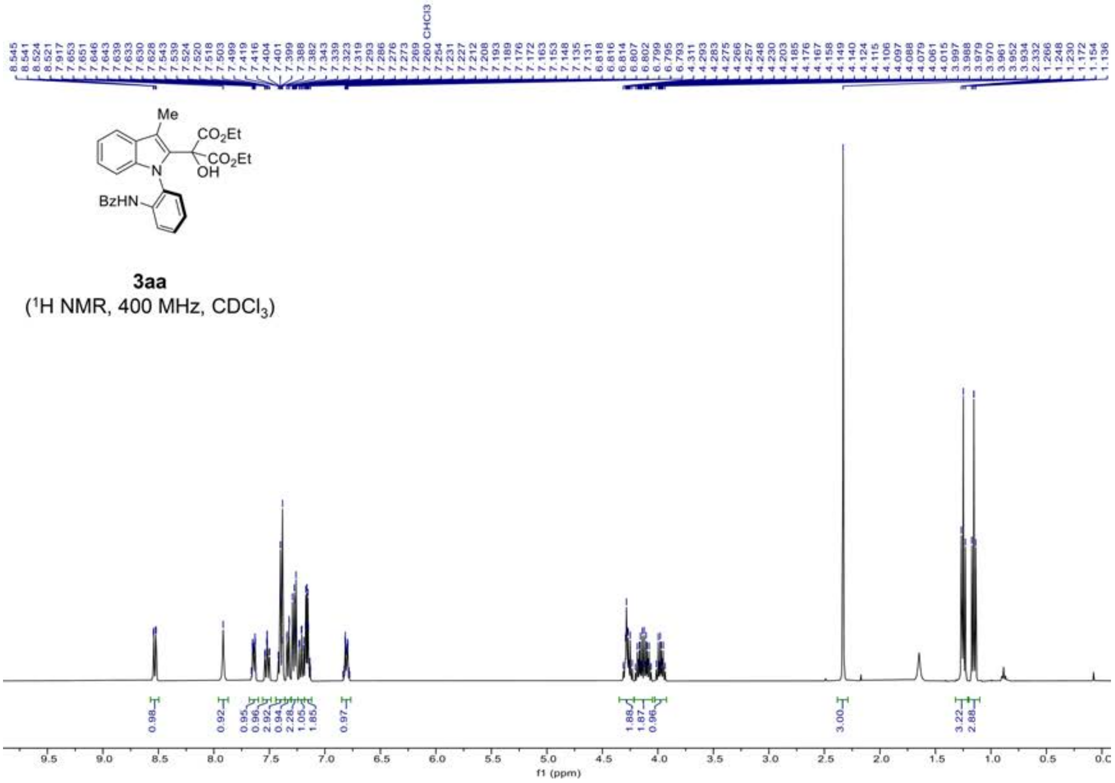

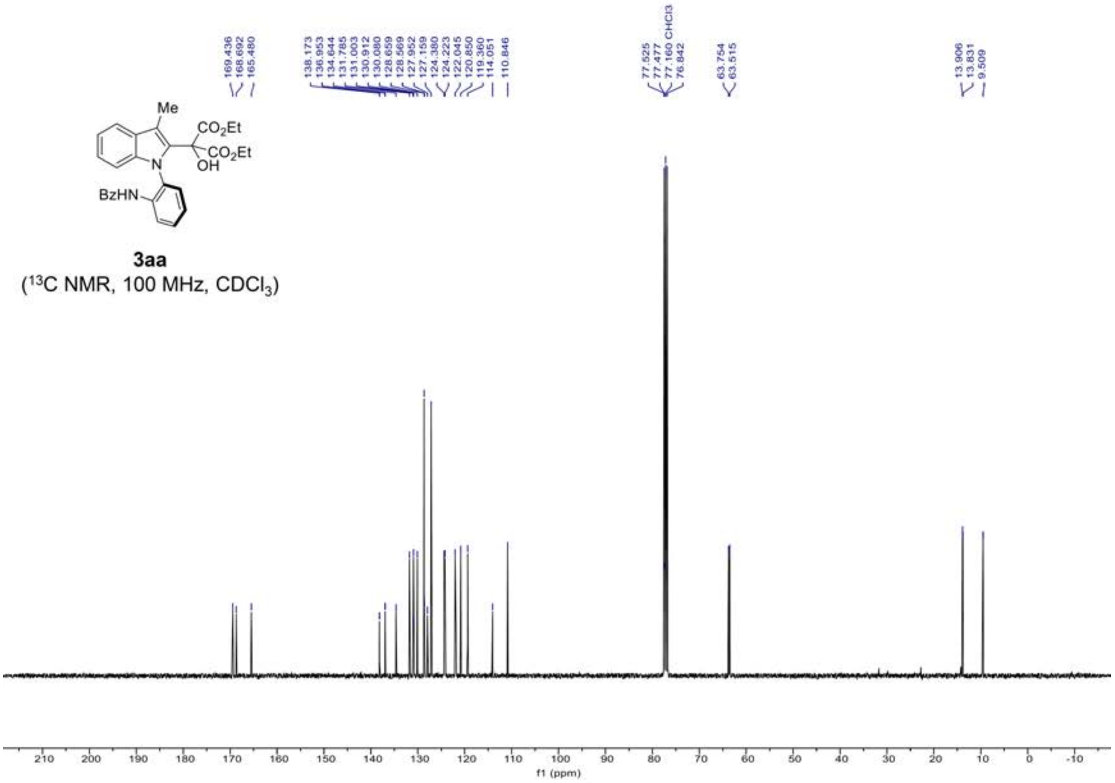

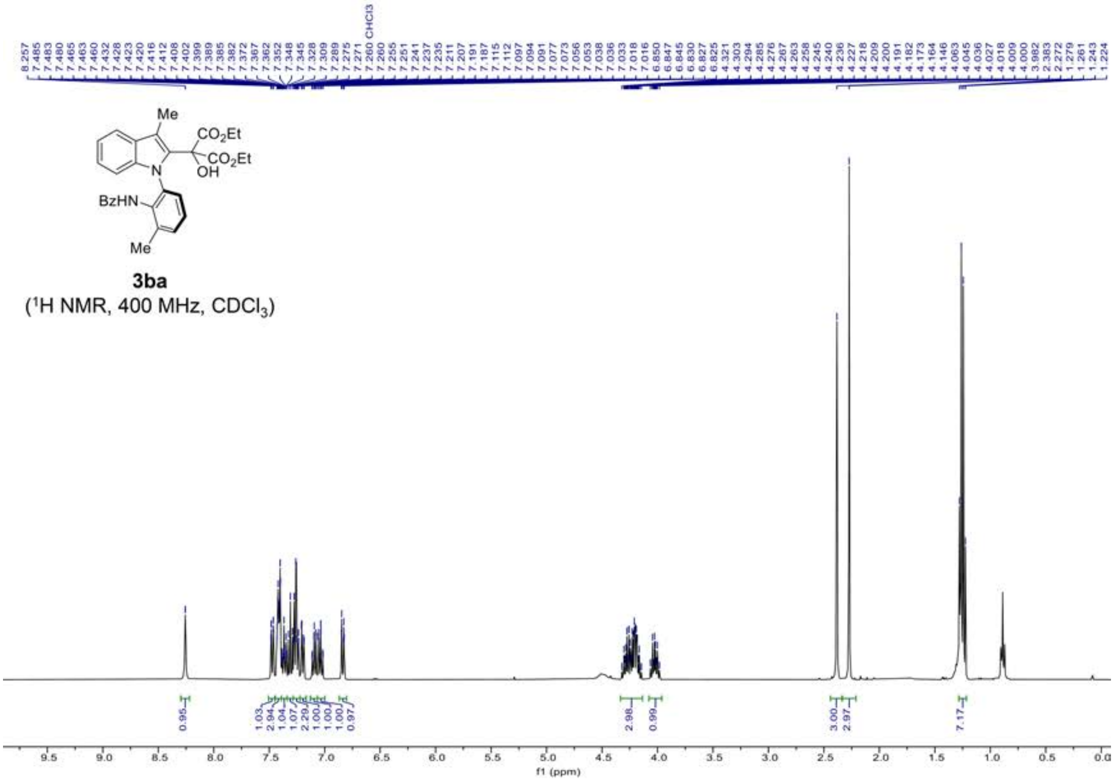
8.257 7.485 7.483 7.480 7.465 7.463 7.460 7.432 7.428 7.423 7.420 7.416 7.412 7.408 7.402 7.399 7.389 7.385 7.382 7.372 7.367 7.362 7.352 7.348 7.345 7.328 7.309 7.289 7.275 7.271 7.260 CHCl3 7.260 7.255 7.251 7.241 7.237 7.235 7.211 7.207 7.191 7.187 7.115 7.112 7.097 7.094 7.091 7.077 7.073 7.056 7.053 7.038 7.036 7.033 7.018 7.016 6.850 6.847 6.845 6.830 6.827 6.825 4.321 4.303 4.294 4.285 4.276 4.267 4.263 4.258 4.245 4.240 4.236 4.227 4.218 4.209 4.200 4.191 4.182 4.173 4.164 4.146 4.063 4.045 4.036 4.027 4.018 4.009 4.000 3.982 2.383 2.272 1.279 1.261 1.243 1.224
Me
CO2Et
CO2Et
OH
N
BzHN
Me
3ba
(1H NMR, 400 MHz, CDCl3)
0.95 1.03 2.94 1.04 1.07 2.29 1.00 1.00 1.00 0.97 2.98 0.99 3.00 2.97 7.17
9.5 9.0 8.5 8.0 7.5 7.0 6.5 6.0 5.5 5.0 4.5 4.0 3.5 3.0 2.5 2.0 1.5 1.0 0.5 0.0
f1 (ppm)

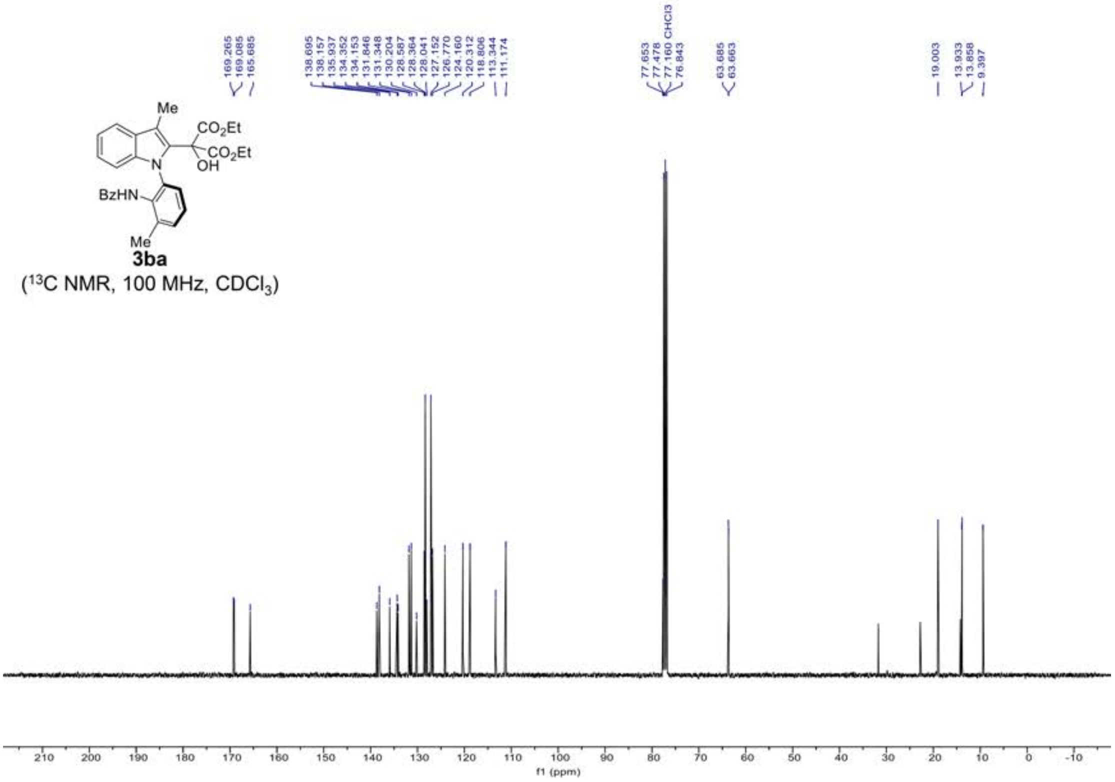
169.265 169.085 165.685 138.695 138.157 135.937 134.352 134.153 131.846 131.348 130.204 128.587 128.364 128.041 127.152 126.770 124.160 120.312 118.806 113.344 111.174 77.653 77.478 77.160 CHCl3 76.843 63.685 63.663 19.003 13.933 13.858 9.397
Me
CO2Et
CO2Et
OH
N
BzHN
Me
3ba
(13C NMR, 100 MHz, CDCl3)
210 200 190 180 170 160 150 140 130 120 110 100 90 80 70 60 50 40 30 20 10 0 -10
f1 (ppm)

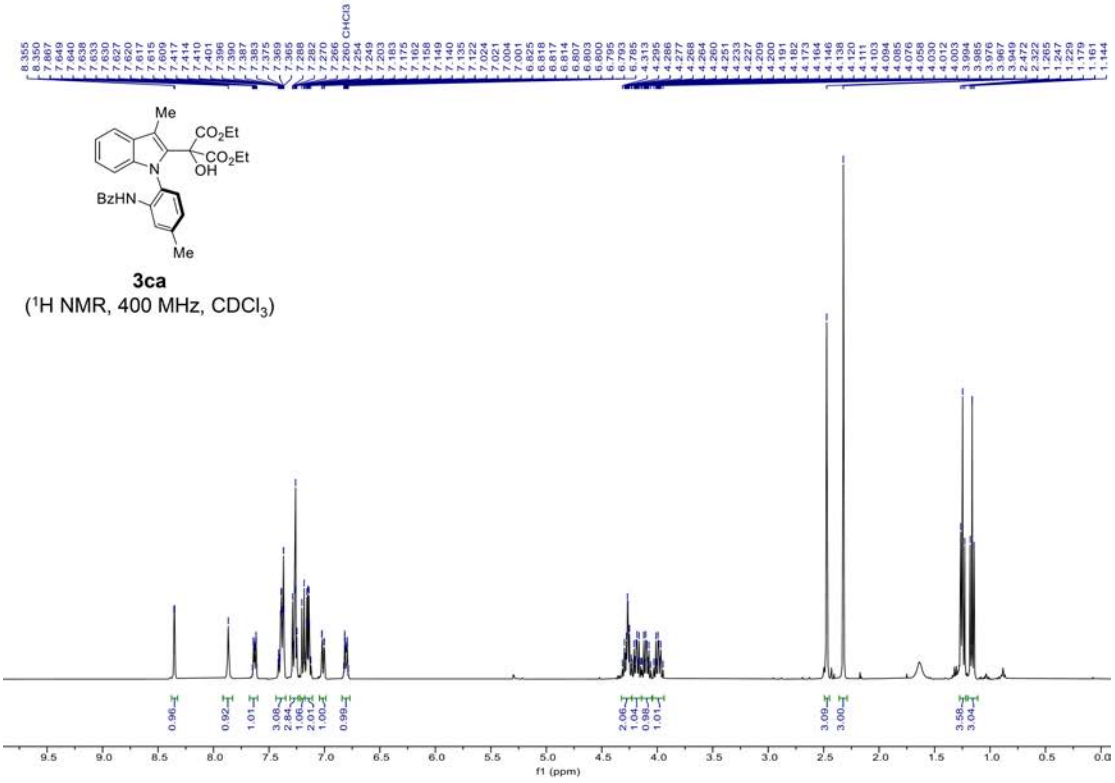
8.355 8.350 7.867 7.649 7.640 7.638 7.633 7.630 7.627 7.620 7.617 7.615 7.609 7.417 7.414 7.410 7.401 7.396 7.390 7.387 7.383 7.375 7.369 7.365 7.288 7.282 7.270 7.266 7.260 CHCl3 7.254 7.249 7.203 7.183 7.175 7.162 7.158 7.149 7.140 7.135 7.122 7.024 7.021 7.004 7.001 6.825 6.818 6.817 6.814 6.807 6.803 6.800 6.795 6.793 6.785 4.313 4.295 4.286 4.277 4.268 4.264 4.260 4.251 4.233 4.227 4.209 4.200 4.191 4.182 4.173 4.164 4.146 4.138 4.120 4.111 4.103 4.094 4.085 4.076 4.058 4.030 4.012 4.003 3.994 3.985 3.976 3.967 3.949 2.472 2.322 1.265 1.247 1.229 1.179 1.161 1.144
Me
CO_2Et
CO_2Et
OH
N
BzHN
Me
3ca
(^{1}H NMR, 400 MHz, $CDCl_3$)
0.96 0.92 1.01 3.08 2.84 1.06 2.01 1.00 0.99 2.06 1.04 0.98 1.01 3.09 3.00 3.58 3.04
9.5 9.0 8.5 8.0 7.5 7.0 6.5 6.0 5.5 5.0 4.5 4.0 3.5 3.0 2.5 2.0 1.5 1.0 0.5 0.0
f1 (ppm)

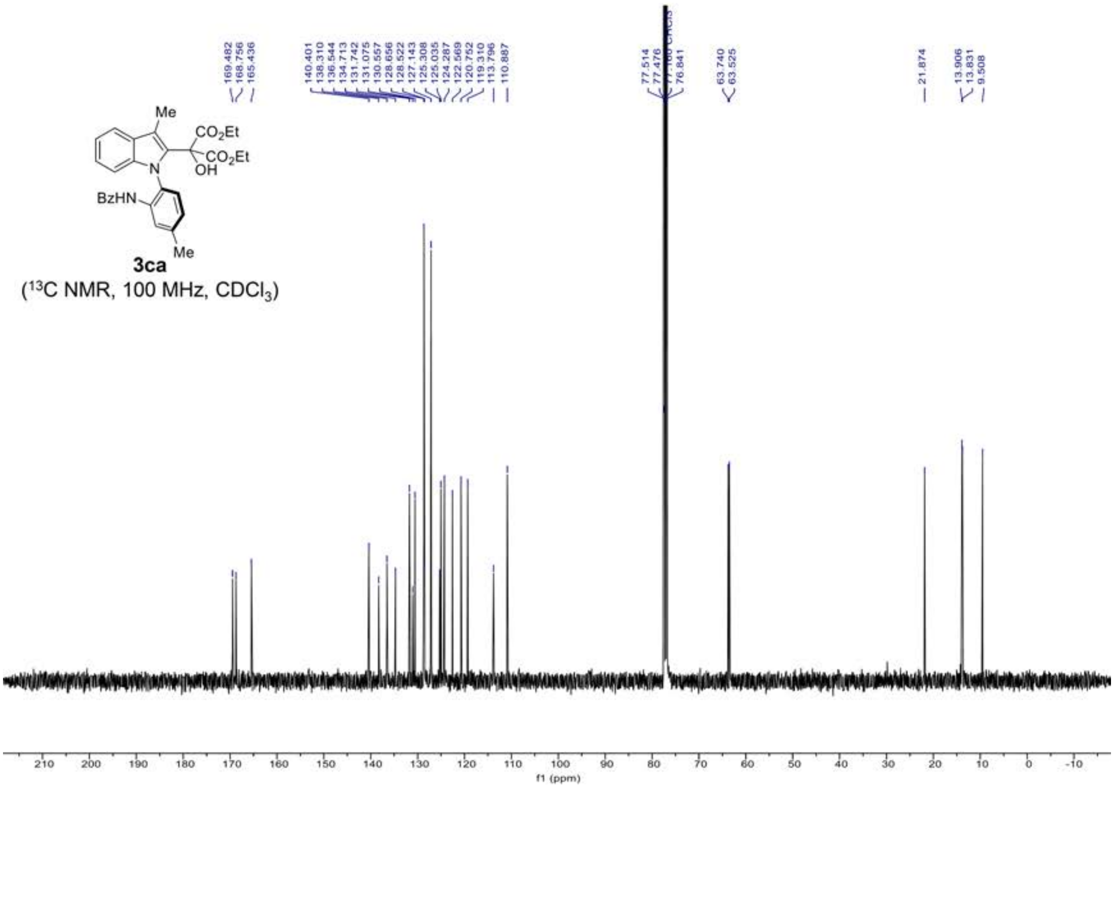
169.482 168.756 165.436
140.401 138.310 136.544 134.713 131.742 131.075 130.657 128.656 128.522 127.143 125.308 125.035 124.287 122.469 120.752 119.310 113.796 110.887
77.514 77.476 77.160 CHCl3 76.841
63.740 63.525
21.874
13.906 13.831 9.508
Me
CO_2Et
CO_2Et
OH
N
BzHN
Me
3ca
(^{13}C NMR, 100 MHz, $CDCl_3$)
210 200 190 180 170 160 150 140 130 120 110 100 90 80 70 60 50 40 30 20 10 0 -10
f1 (ppm)

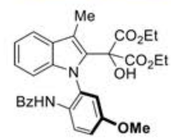

3da
(^{1}H NMR, 400 MHz, $CDCl_3$)

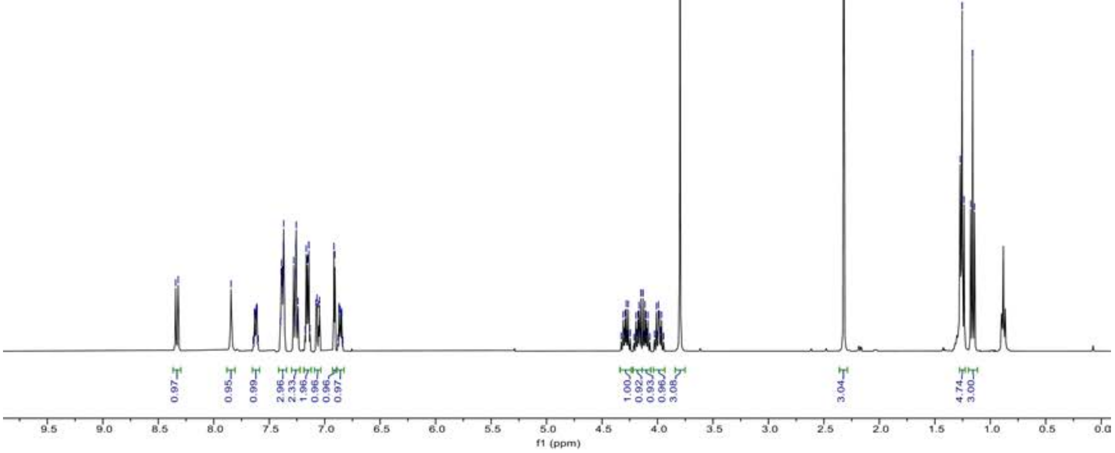

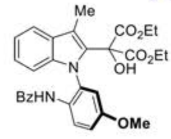

3da
(^{13}C NMR, 100 MHz, $CDCl_3$)

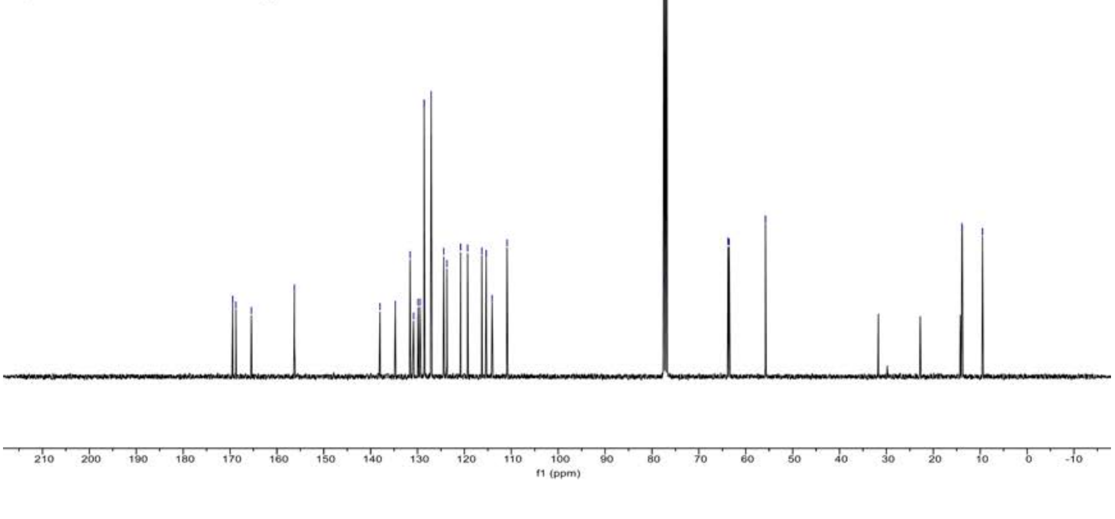

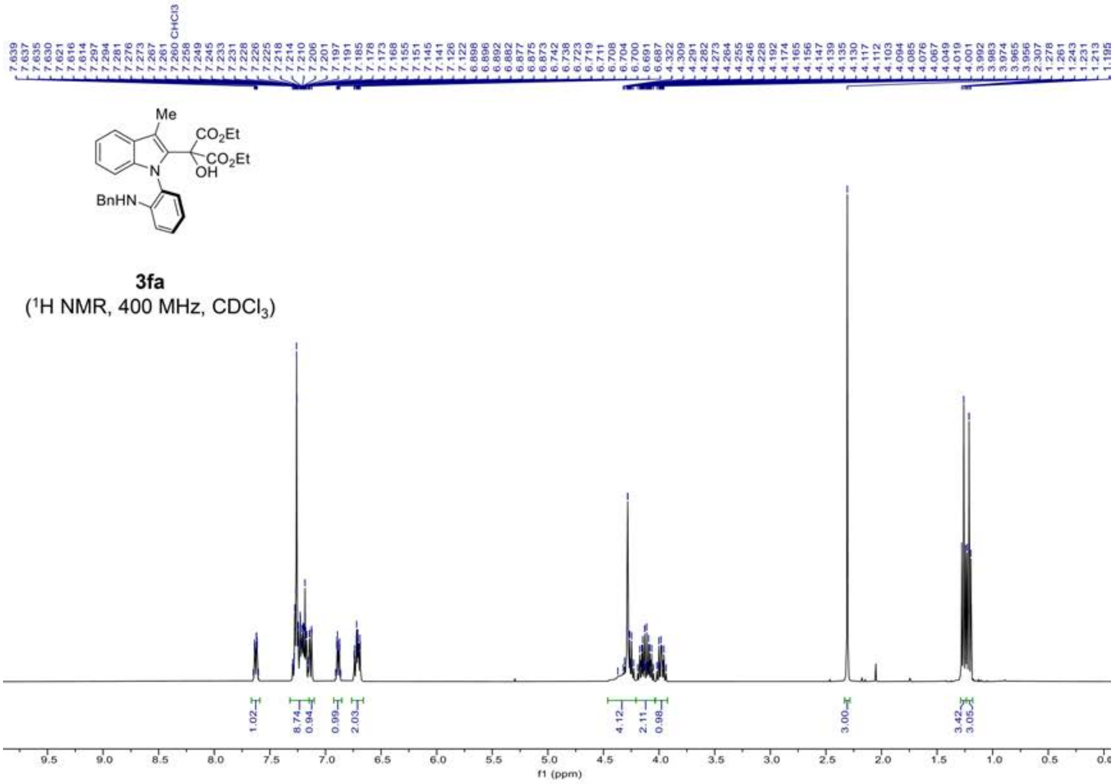

3fa
(^1H NMR, 400 MHz, $CDCl_3$)

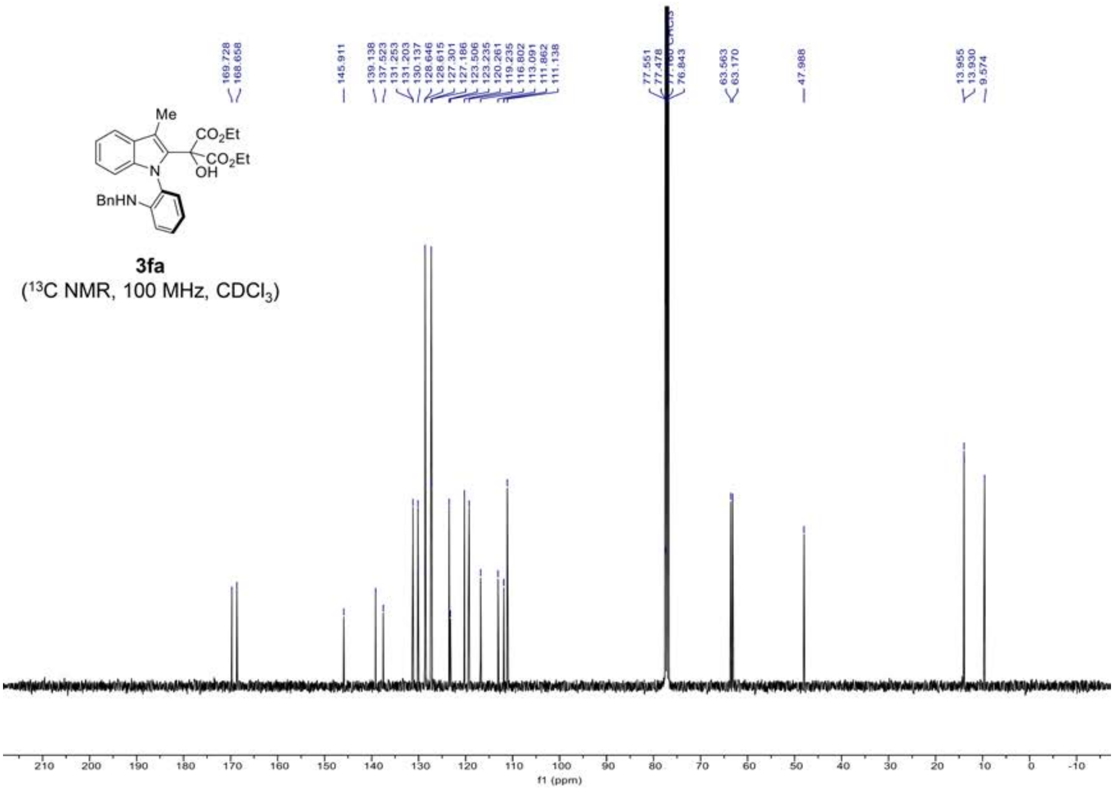

3fa
(^{13}C NMR, 100 MHz, $CDCl_3$)

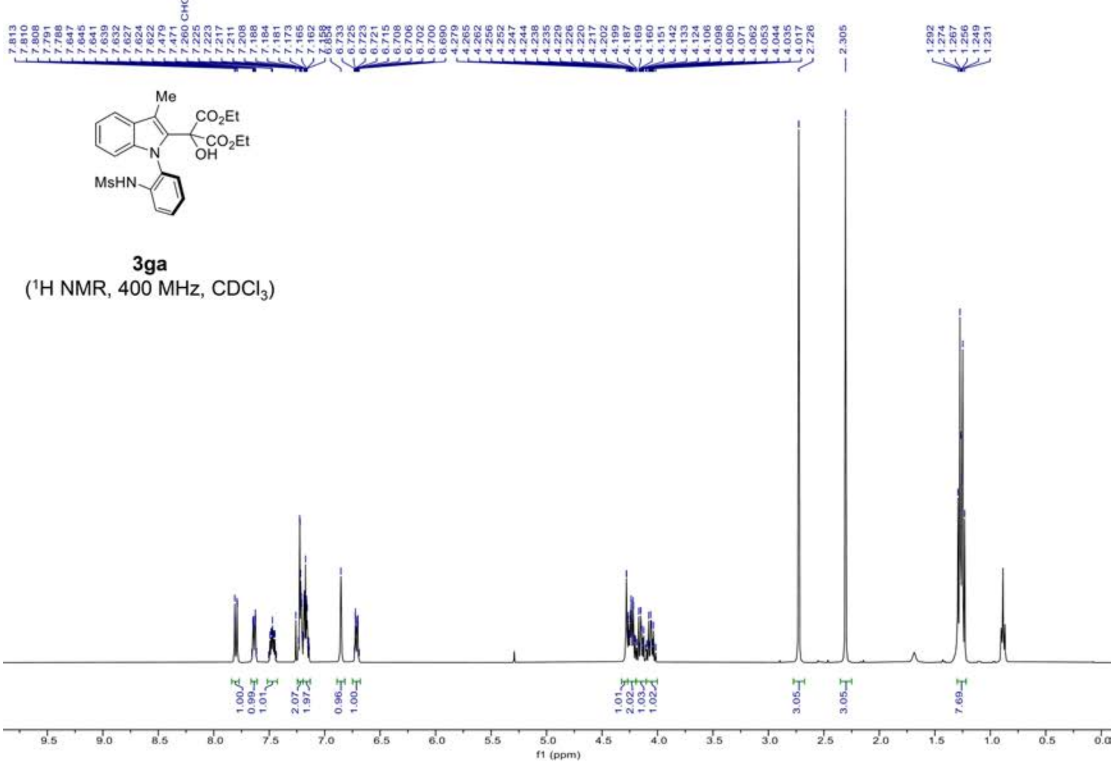
7.813 7.810 7.791 7.788 7.647 7.645 7.641 7.639 7.632 7.627 7.624 7.622 7.479 7.471 7.260 CHCl3 7.225 7.223 7.217 7.211 7.208 7.188 7.184 7.181 7.173 7.165 7.162 6.733 6.725 6.723 6.721 6.715 6.708 6.706 6.702 6.700 6.690 4.279 4.265 4.262 4.256 4.252 4.247 4.244 4.238 4.235 4.229 4.226 4.220 4.217 4.202 4.199 4.187 4.169 4.160 4.151 4.142 4.133 4.124 4.106 4.098 4.080 4.071 4.062 4.053 4.044 4.035 4.017 2.726
2.305
1.292 1.274 1.267 1.256 1.249 1.231
Me
CO2Et
CO2Et
OH
N
MsHN
3ga
(1H NMR, 400 MHz, CDCl3)
1.00 0.99 1.01 2.07 1.97 0.96 1.00 1.01 2.02 1.03 1.02 3.05 3.05 7.69
9.5 9.0 8.5 8.0 7.5 7.0 6.5 6.0 5.5 5.0 4.5 4.0 3.5 3.0 2.5 2.0 1.5 1.0 0.5 0.0
f1 (ppm)

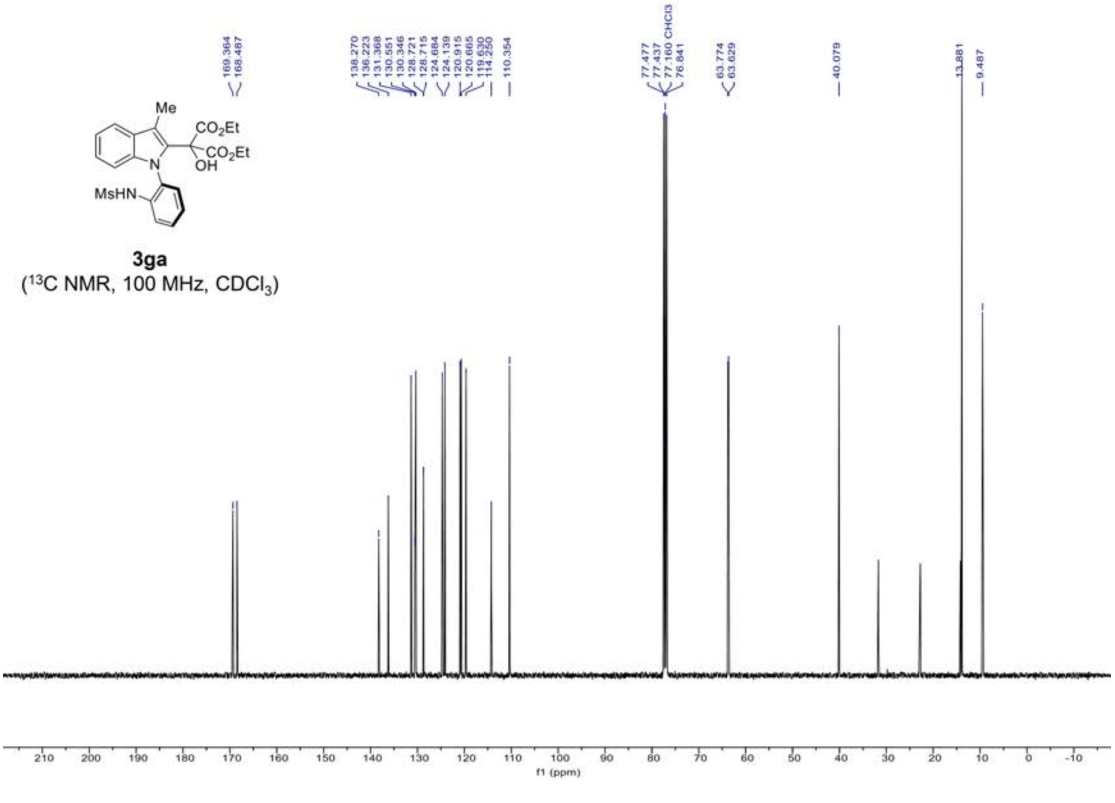
169.364 168.487
138.270 136.223 131.368 130.551 130.346 128.721 128.715 124.684 124.139 120.915 120.665 119.630 114.250 110.354
77.477 77.437 77.160 CHCl3 76.841
63.774 63.629
40.079
13.881
9.487
Me
CO2Et
CO2Et
OH
N
MsHN
3ga
(13C NMR, 100 MHz, CDCl3)
210 200 190 180 170 160 150 140 130 120 110 100 90 80 70 60 50 40 30 20 10 0 -10
f1 (ppm)

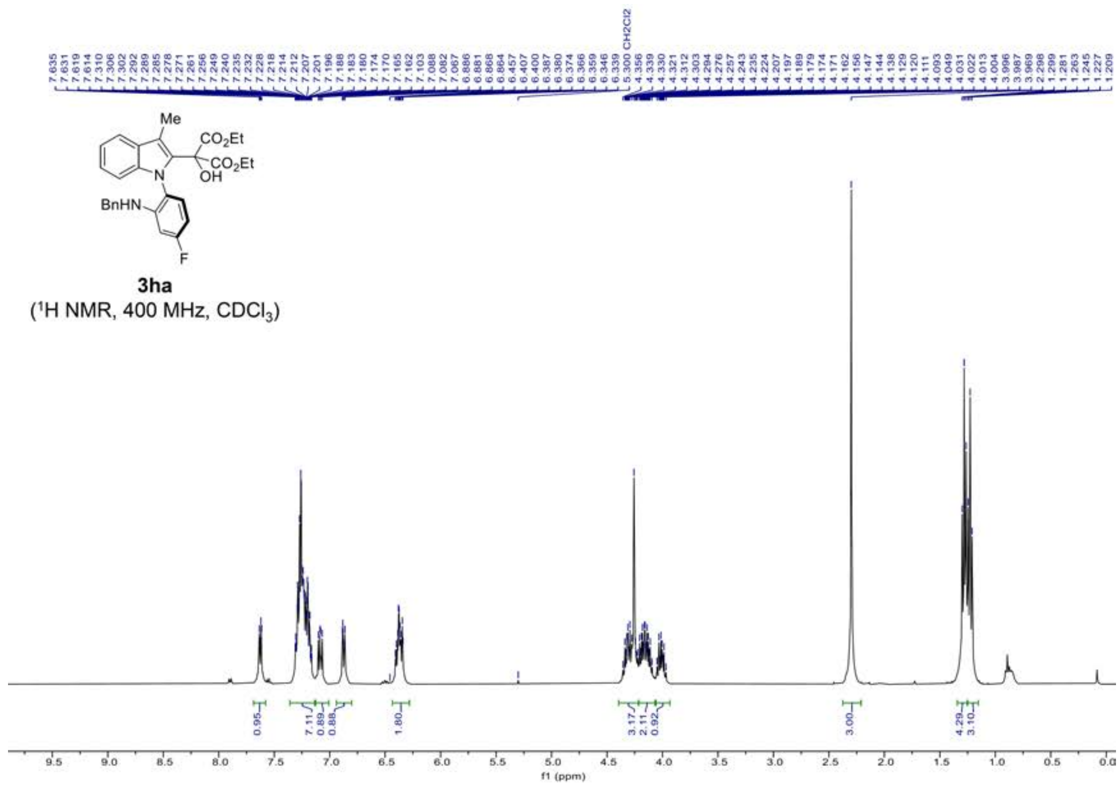
Me
CO2Et
CO2Et
OH
N
BnHN
F
3ha
(1H NMR, 400 MHz, CDCl3)
f1 (ppm)

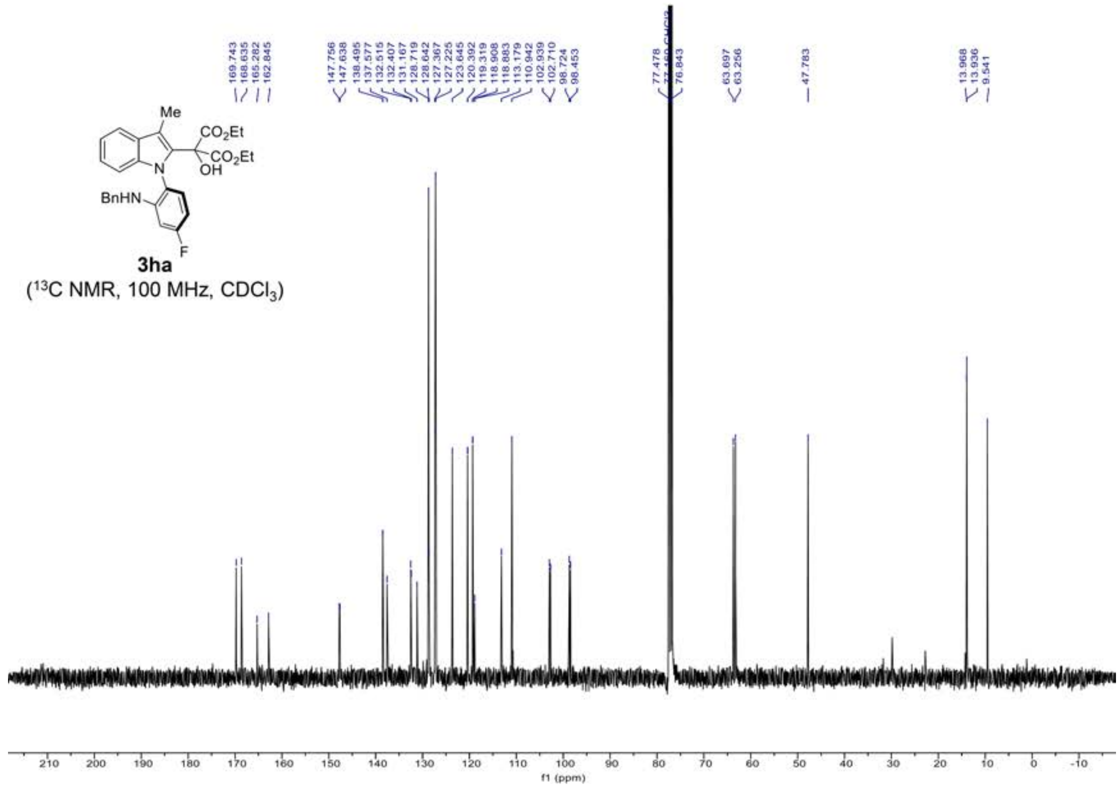
Me
CO2Et
CO2Et
OH
N
BnHN
F
3ha
(13C NMR, 100 MHz, CDCl3)
f1 (ppm)

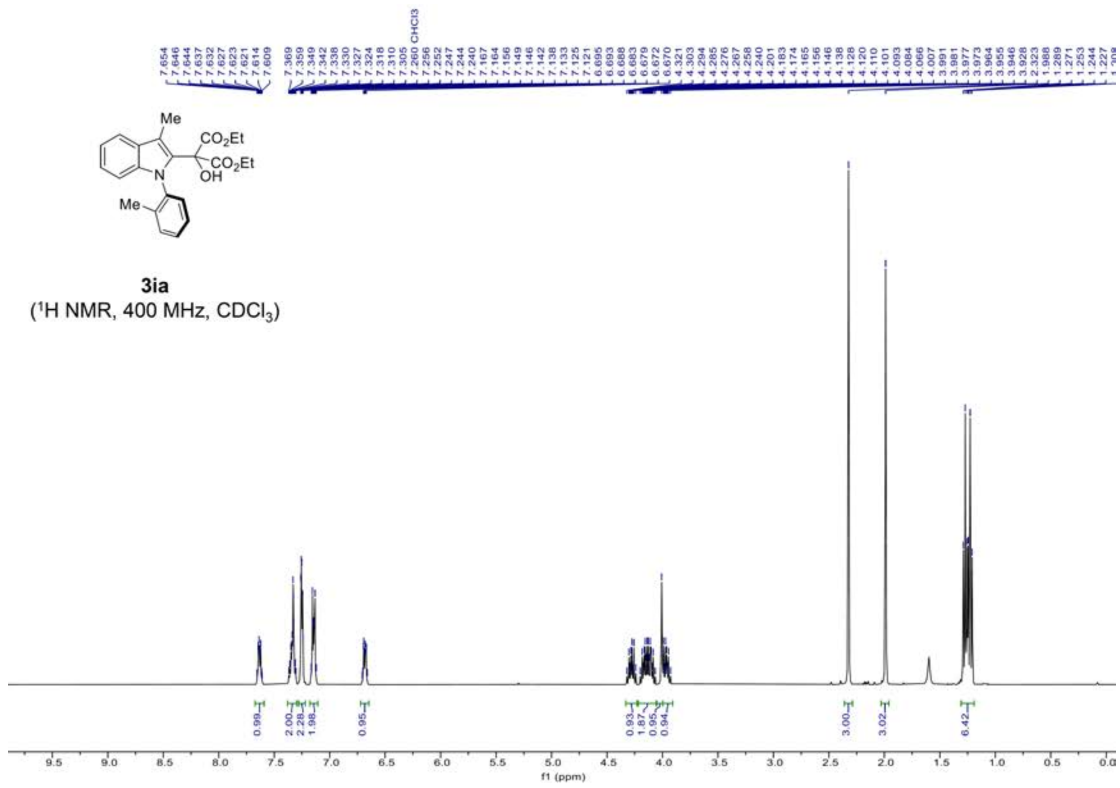

3ia
(^{1}H NMR, 400 MHz, $CDCl_3$)

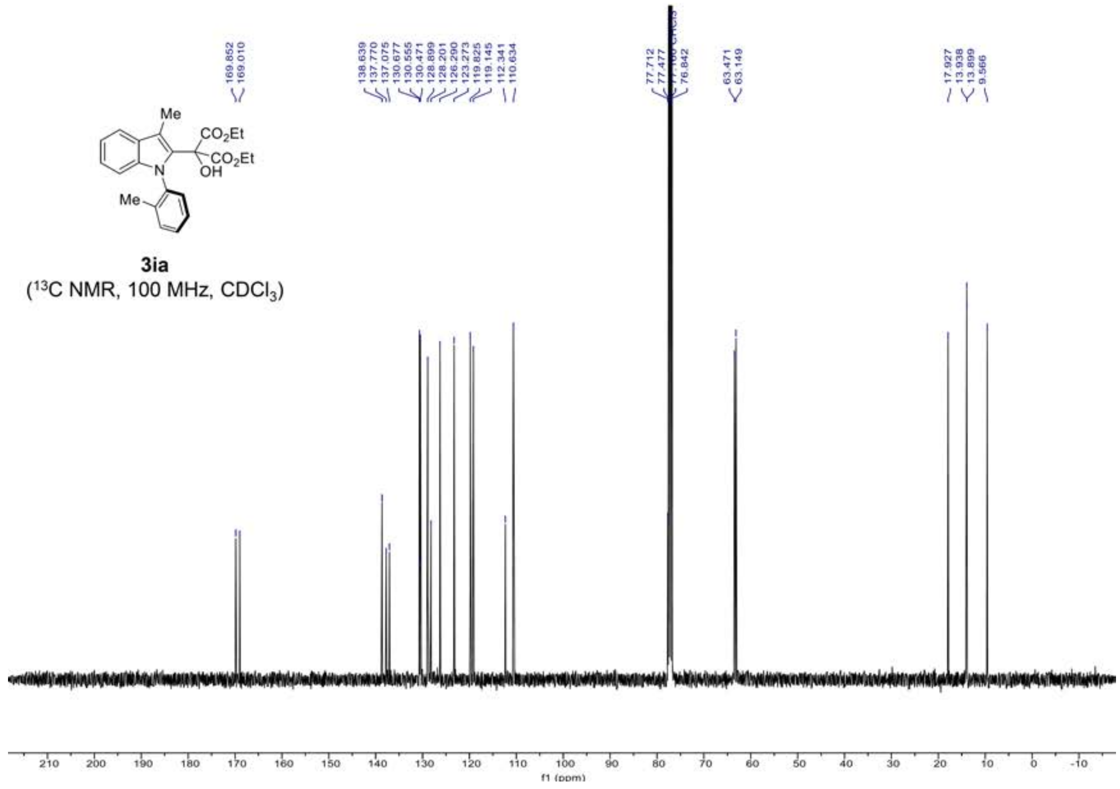

3ia
(^{13}C NMR, 100 MHz, $CDCl_3$)

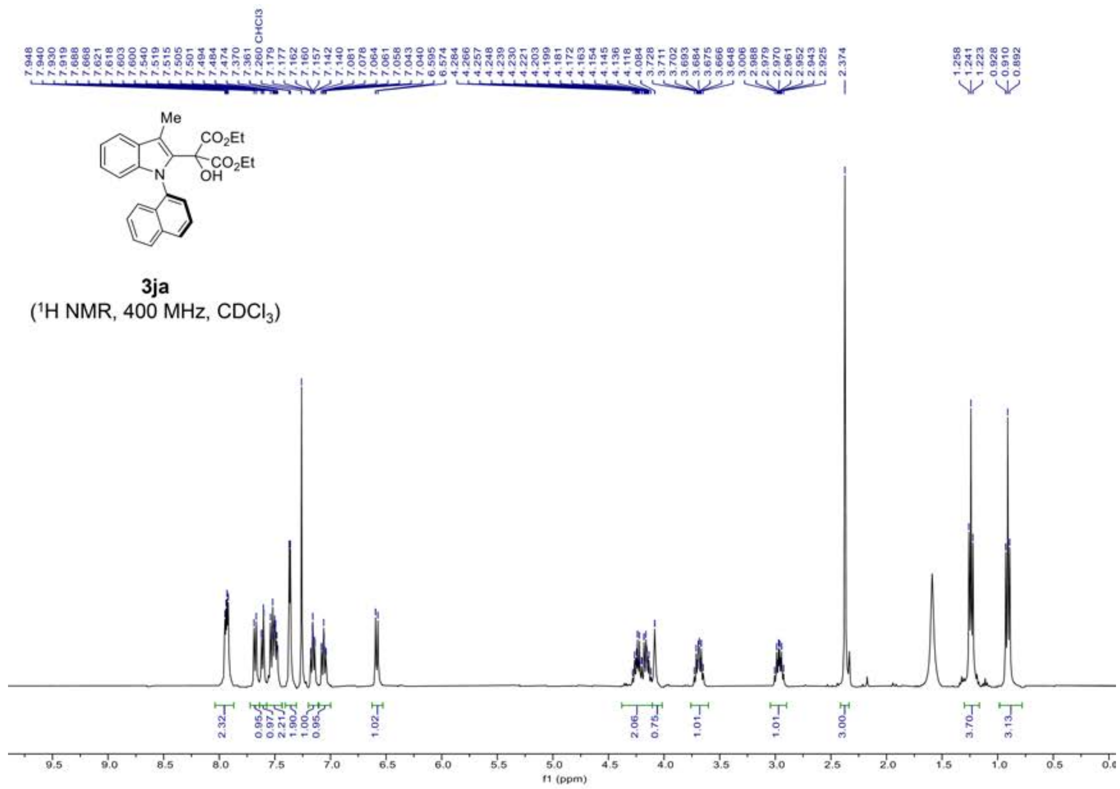

3ja
(^{1}H NMR, 400 MHz, $CDCl_3$)

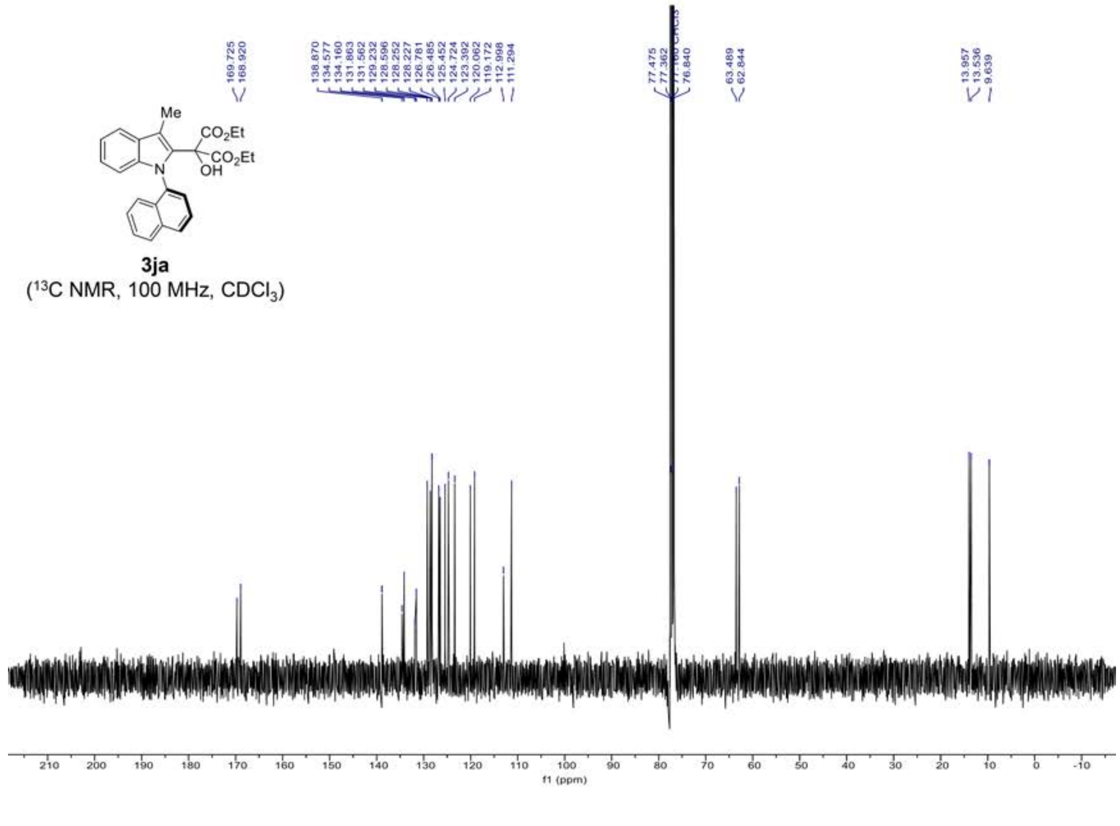

3ja
(^{13}C NMR, 100 MHz, $CDCl_3$)

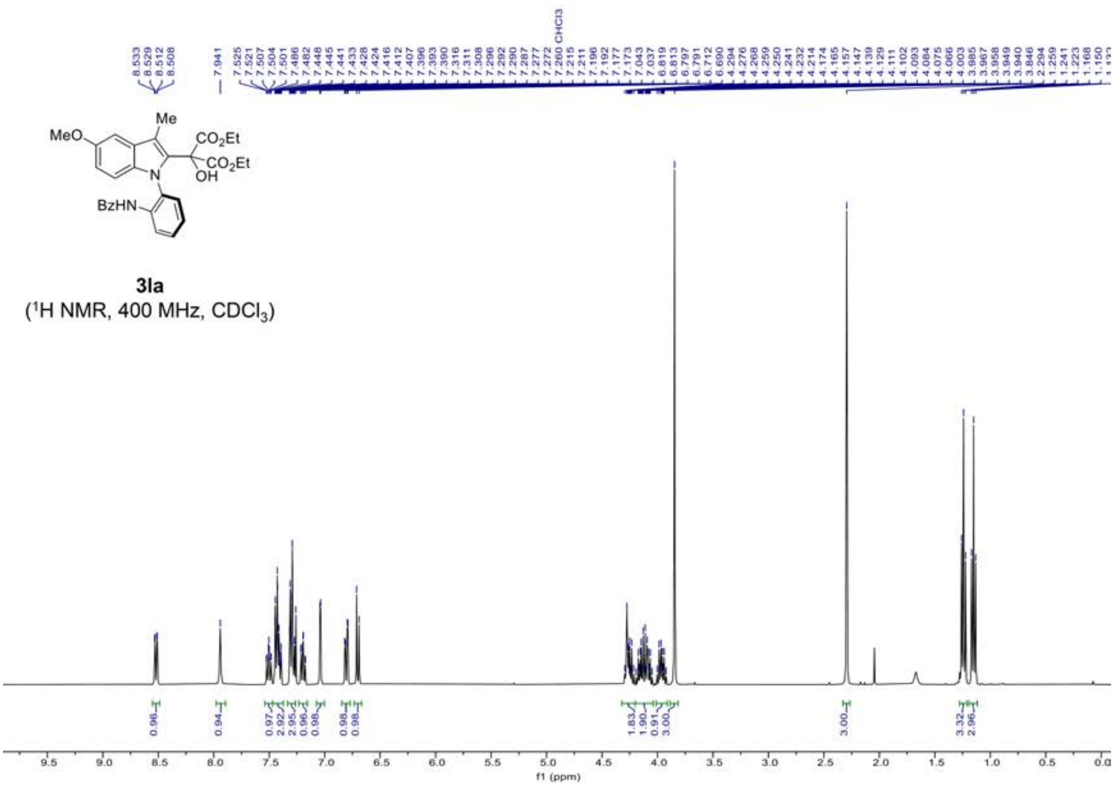

3la
(^{1}H NMR, 400 MHz, $CDCl_3$)

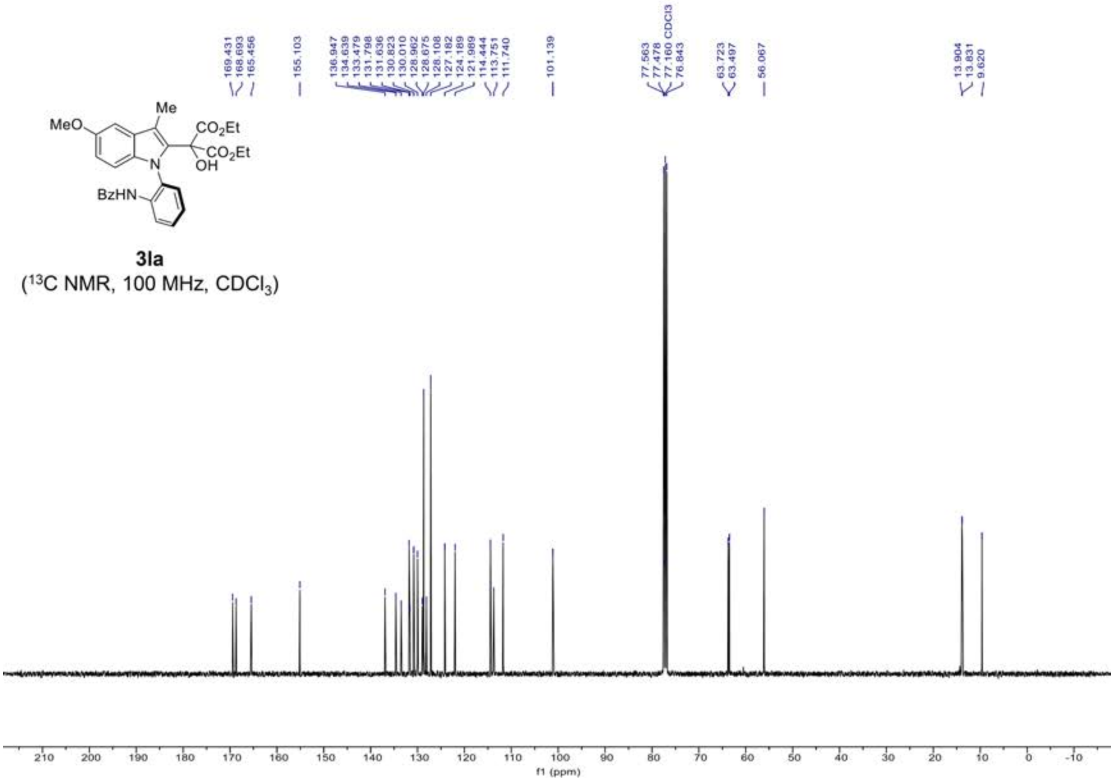

3la
(^{13}C NMR, 100 MHz, $CDCl_3$)

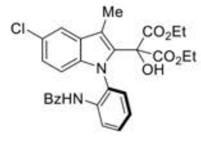

3ma
(^{1}H NMR, 400 MHz, $CDCl_3$)

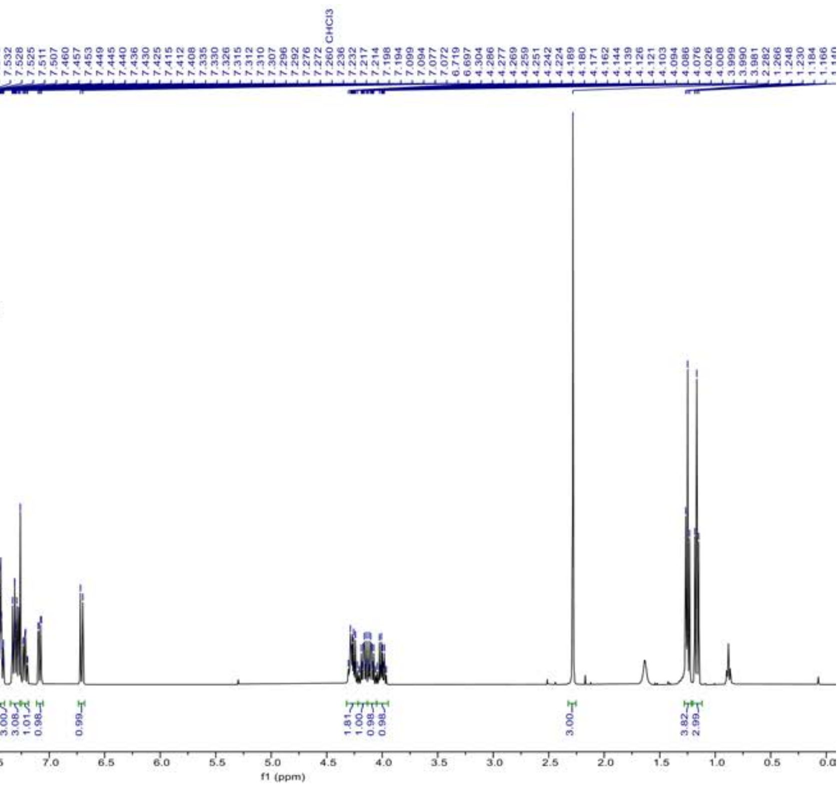

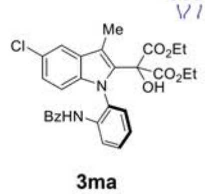

3ma
(^{13}C NMR, 100 MHz, $CDCl_3$)

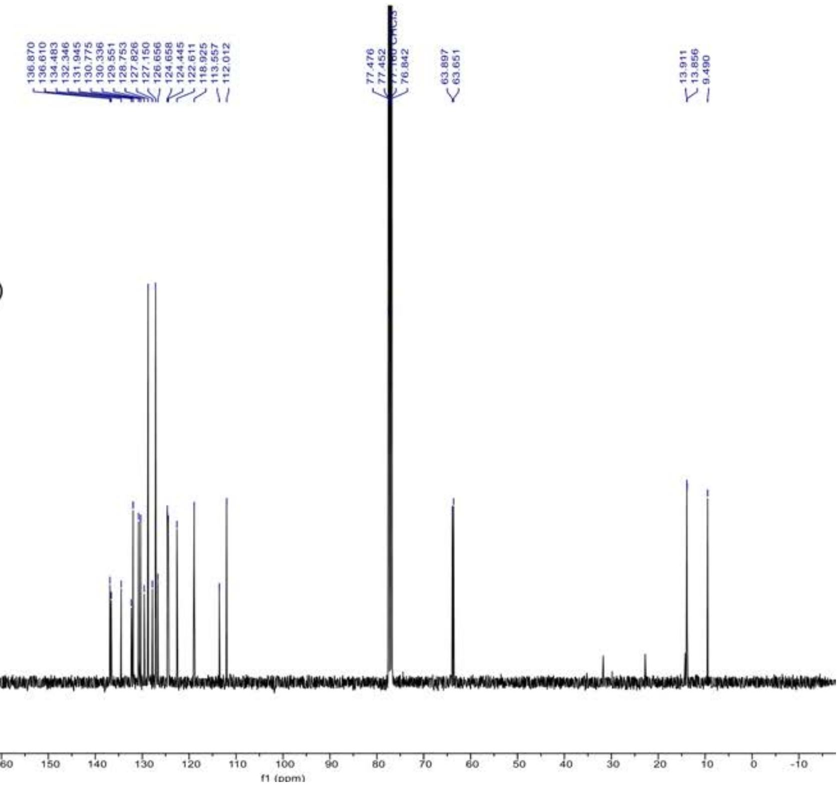

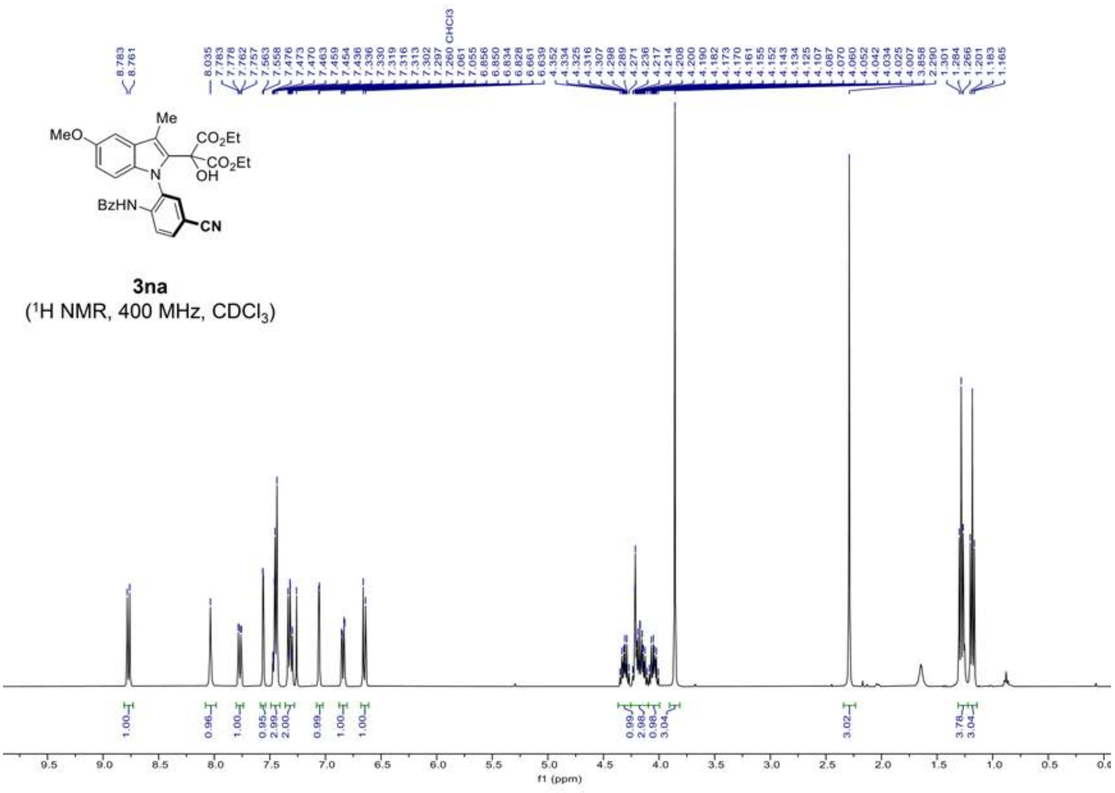
8.783
8.761
8.035
7.783
7.778
7.762
7.757
7.563
7.558
7.476
7.473
7.470
7.463
7.459
7.454
7.436
7.336
7.330
7.319
7.316
7.313
7.302
7.297
7.260 CHCl3
7.061
7.055
6.856
6.850
6.834
6.828
6.661
6.639
4.352
4.334
4.325
4.316
4.307
4.298
4.289
4.271
4.236
4.217
4.214
4.208
4.200
4.190
4.182
4.173
4.170
4.161
4.155
4.152
4.143
4.134
4.125
4.107
4.087
4.070
4.060
4.052
4.042
4.034
4.025
4.007
3.858
2.290
1.301
1.284
1.266
1.201
1.183
1.165
MeO
Me
CO2Et
CO2Et
OH
N
BzHN
CN
3na
(1H NMR, 400 MHz, CDCl3)
1.00
0.96
1.00
0.95
2.99
2.00
0.99
1.00
1.00
0.99
2.98
0.98
3.04
3.02
3.78
3.04
9.5
9.0
8.5
8.0
7.5
7.0
6.5
6.0
5.5
5.0
4.5
4.0
3.5
3.0
2.5
2.0
1.5
1.0
0.5
0.0
f1 (ppm)

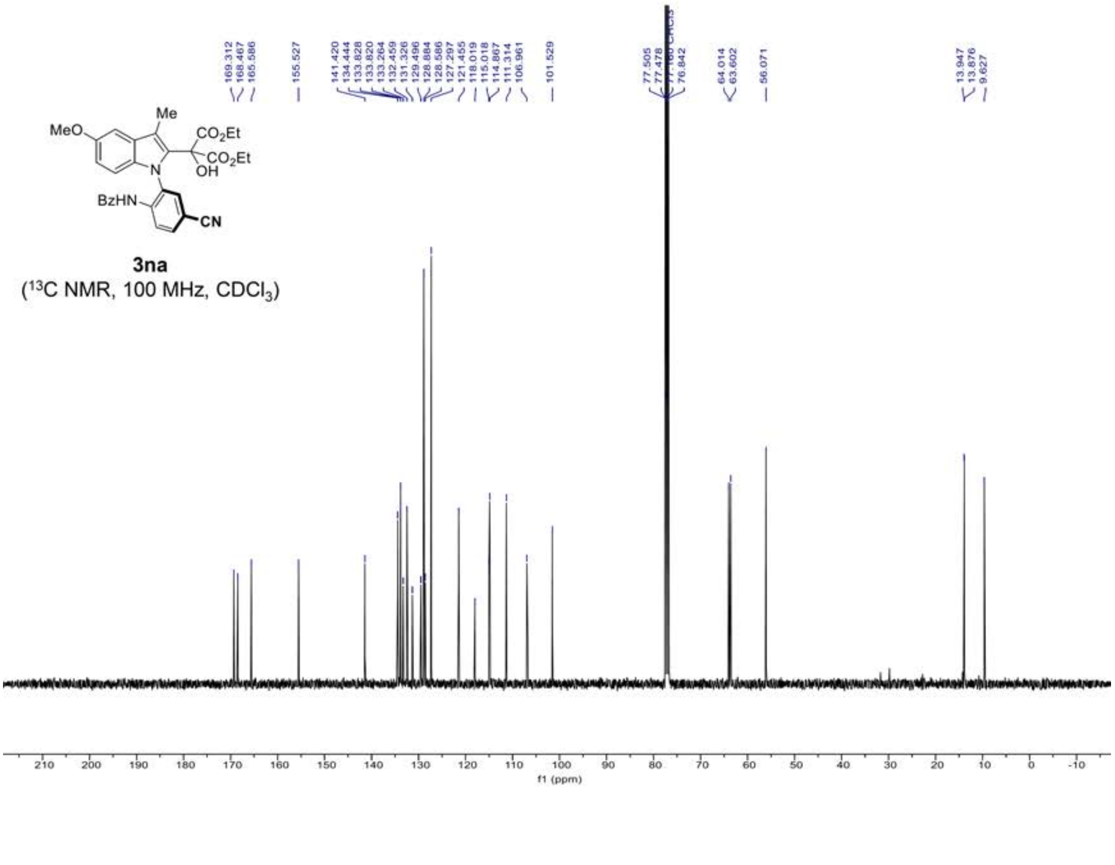
169.312
168.467
165.586
155.527
141.420
134.444
133.828
133.820
133.264
132.459
131.326
129.496
128.884
128.586
127.297
121.455
118.019
115.018
114.867
111.314
106.961
101.529
77.505
77.478
77.160 CHCl3
76.842
64.014
63.602
56.071
13.947
13.876
9.627
MeO
Me
CO2Et
CO2Et
OH
N
BzHN
CN
3na
(13C NMR, 100 MHz, CDCl3)
210
200
190
180
170
160
150
140
130
120
110
100
90
80
70
60
50
40
30
20
10
0
-10
f1 (ppm)

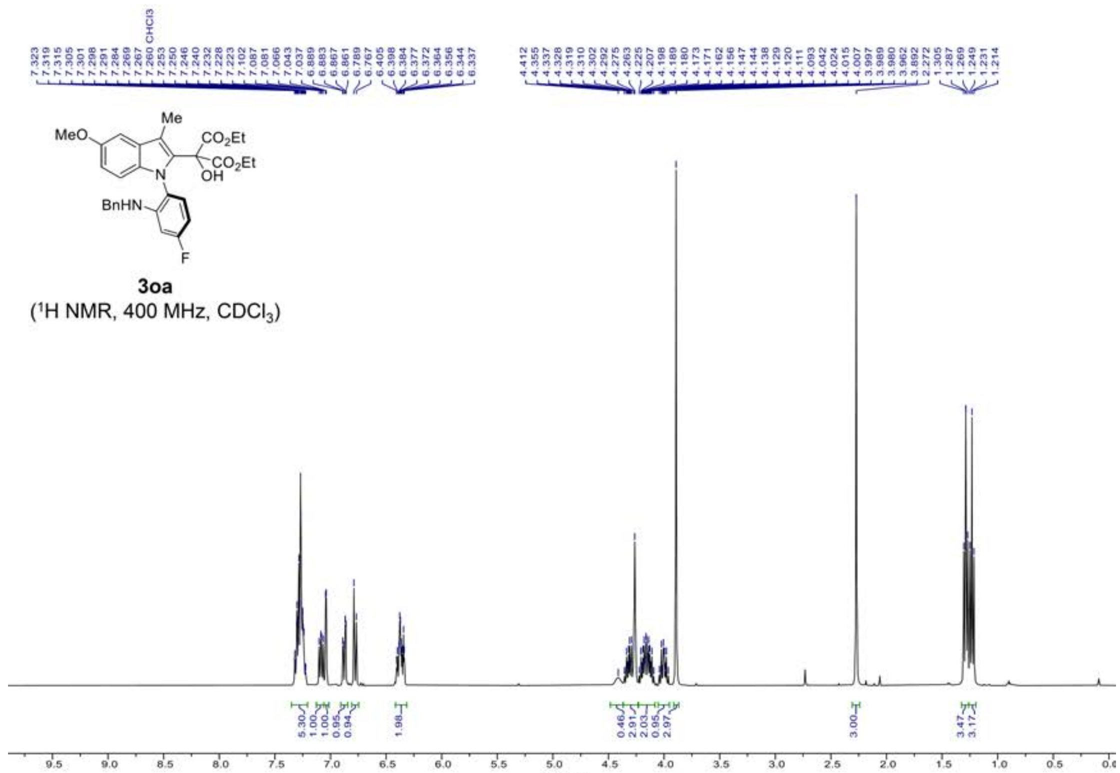

3oa
(^{1}H NMR, 400 MHz, $CDCl_3$)

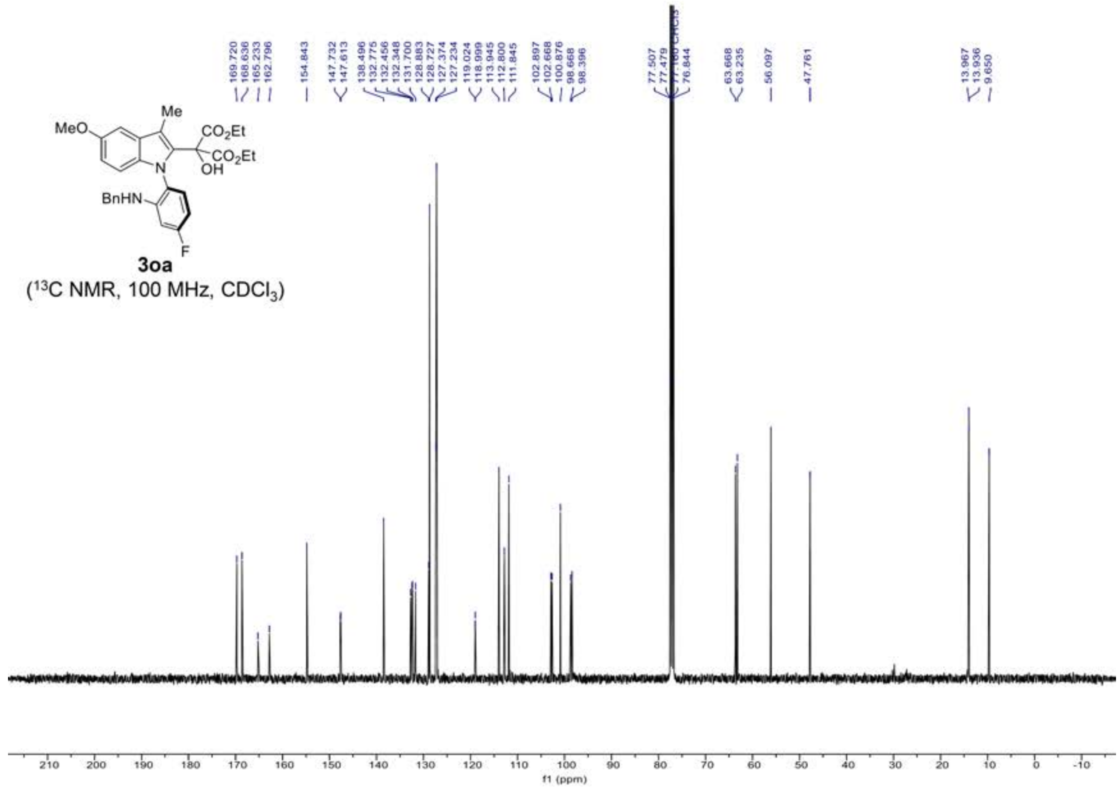

3oa
(^{13}C NMR, 100 MHz, $CDCl_3$)

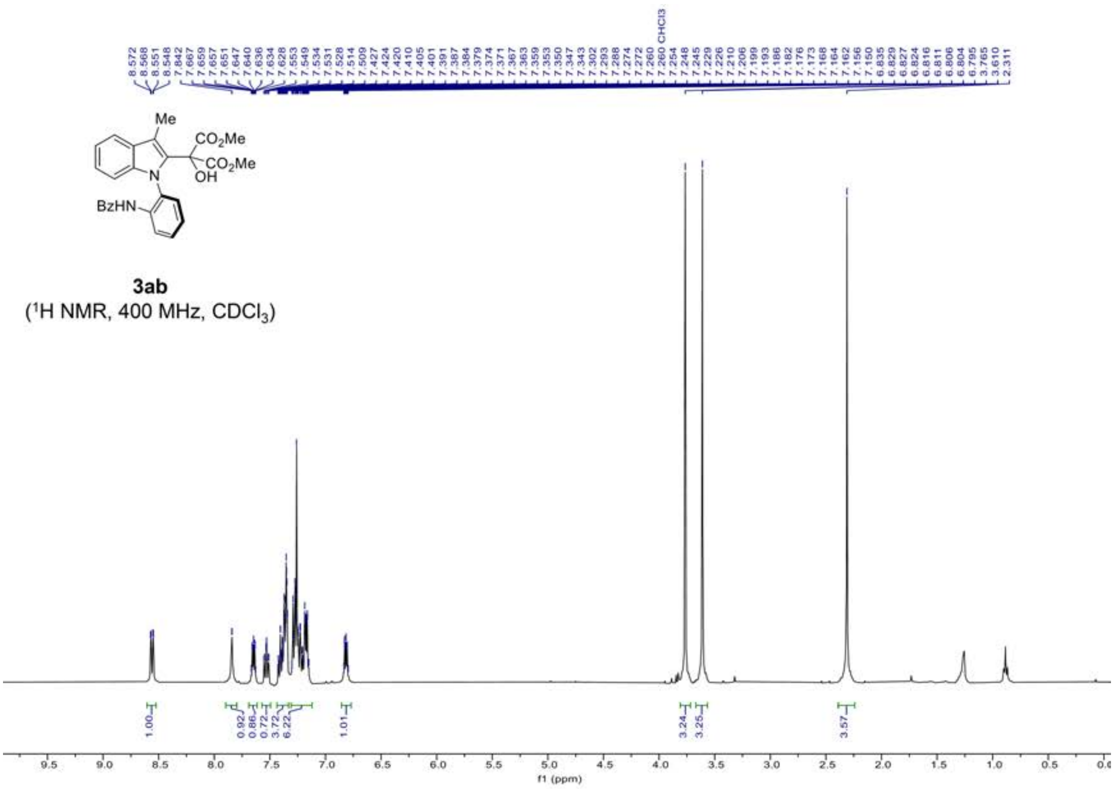

3ab
(^{1}H NMR, 400 MHz, $CDCl_3$)

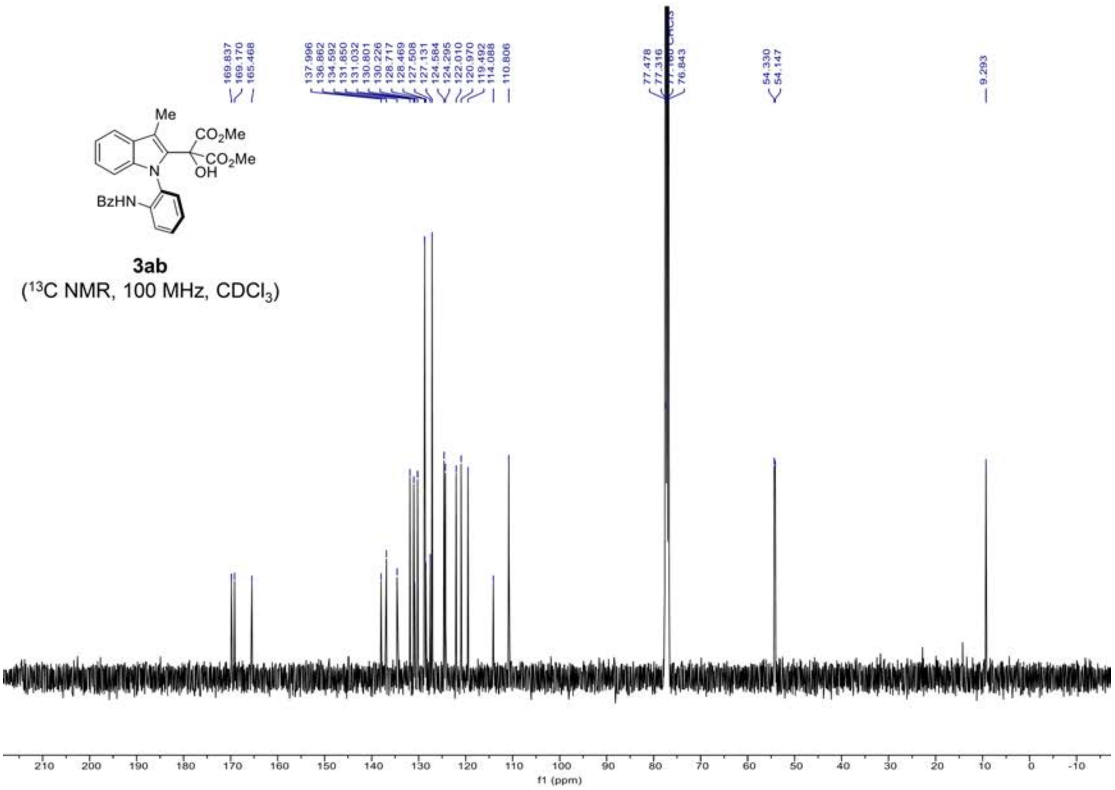

3ab
(^{13}C NMR, 100 MHz, $CDCl_3$)

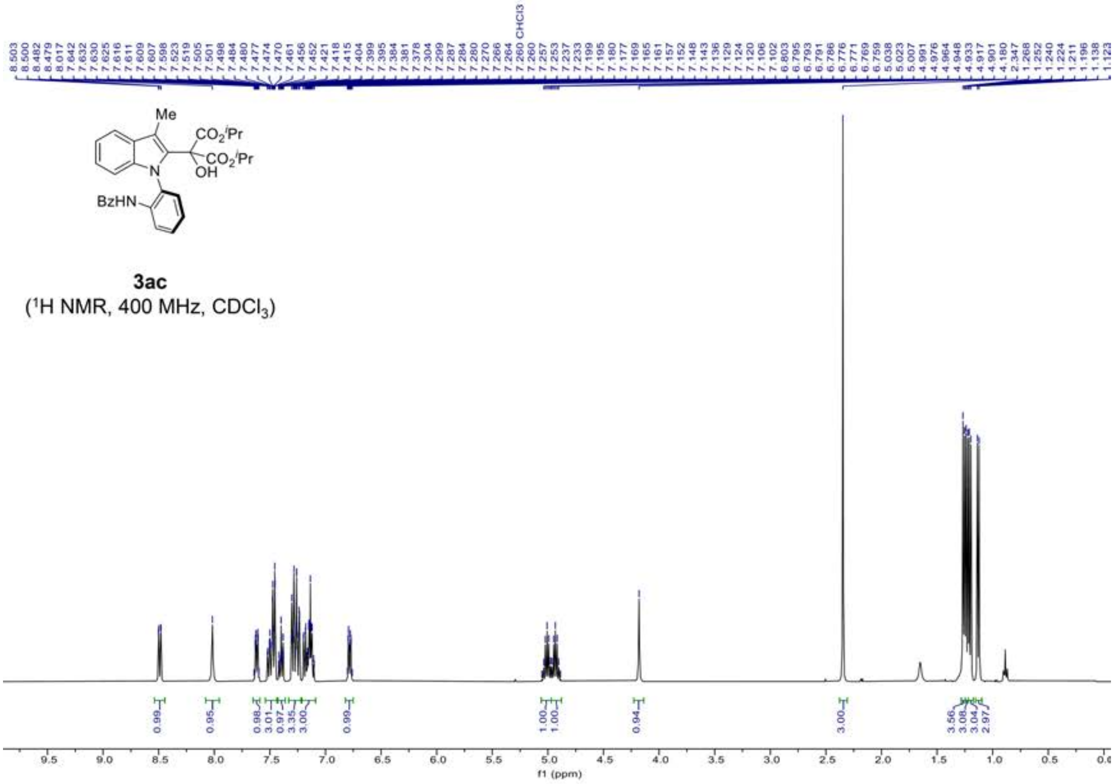

3ac
(^{1}H NMR, 400 MHz, $CDCl_3$)

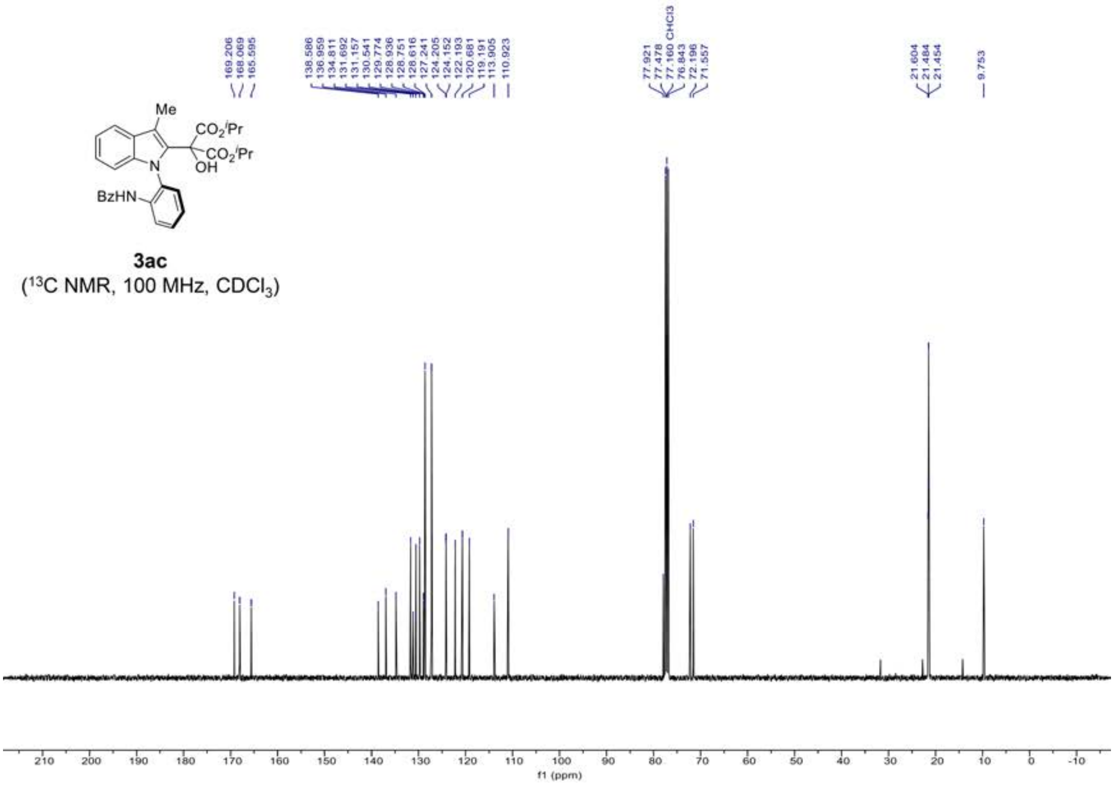

3ac
(^{13}C NMR, 100 MHz, $CDCl_3$)

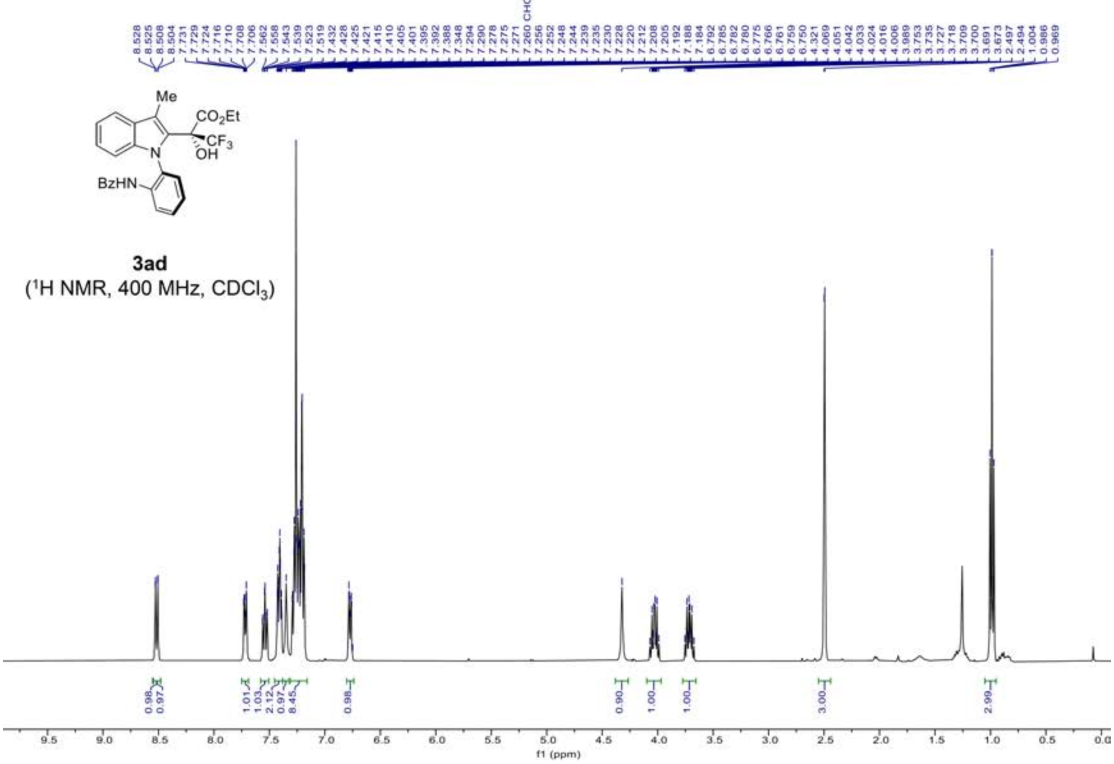
Me
CO2Et
CF3
OH
N
BzHN
3ad
(1H NMR, 400 MHz, CDCl3)
f1 (ppm)

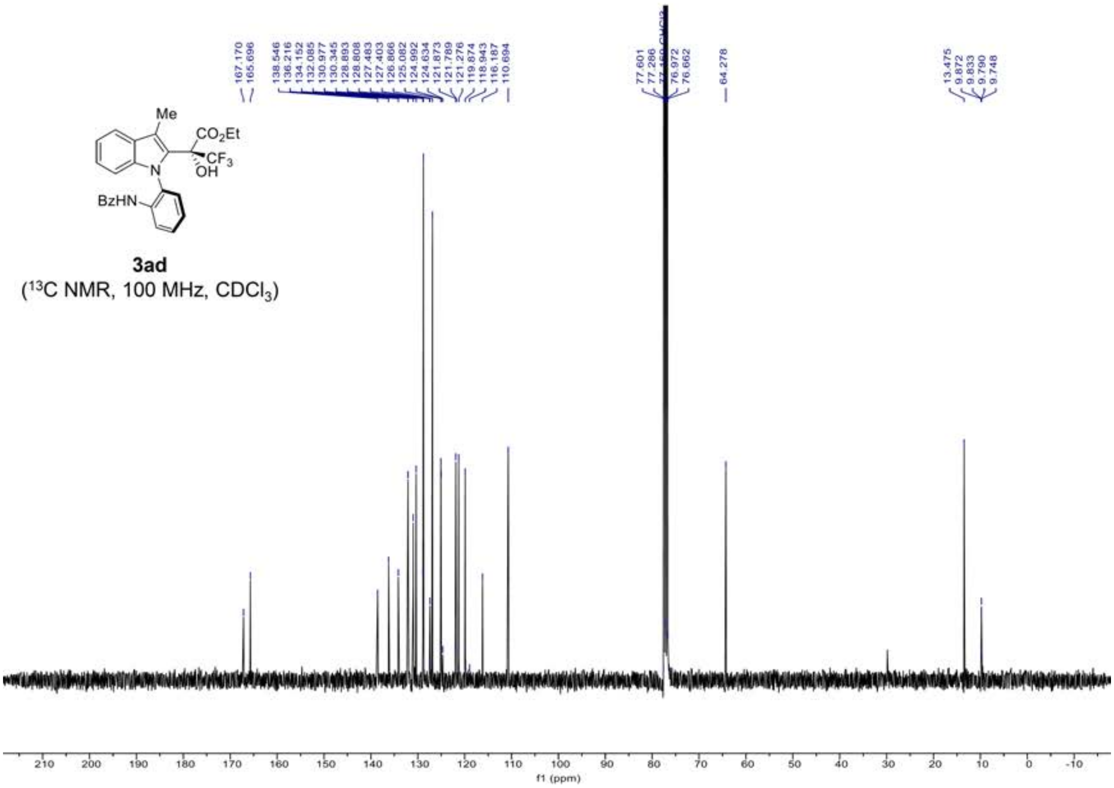
Me
CO2Et
CF3
OH
N
BzHN
3ad
(13C NMR, 100 MHz, CDCl3)
f1 (ppm)

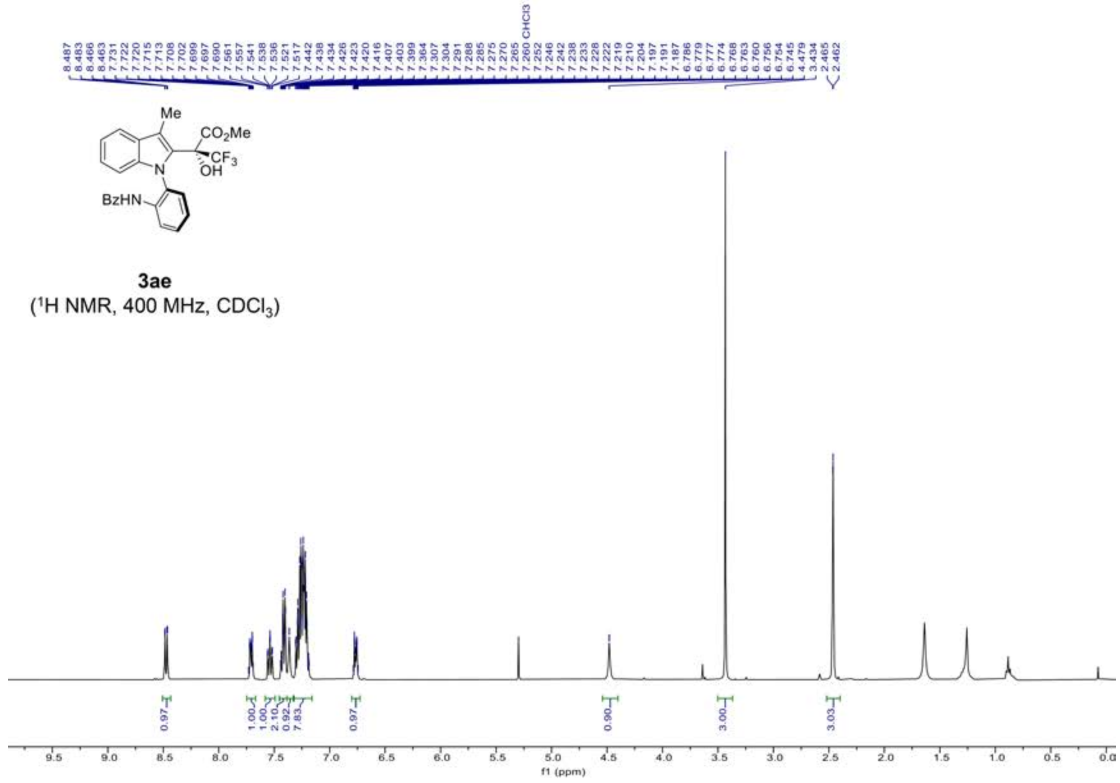

3ae
(^{1}H NMR, 400 MHz, $CDCl_3$)

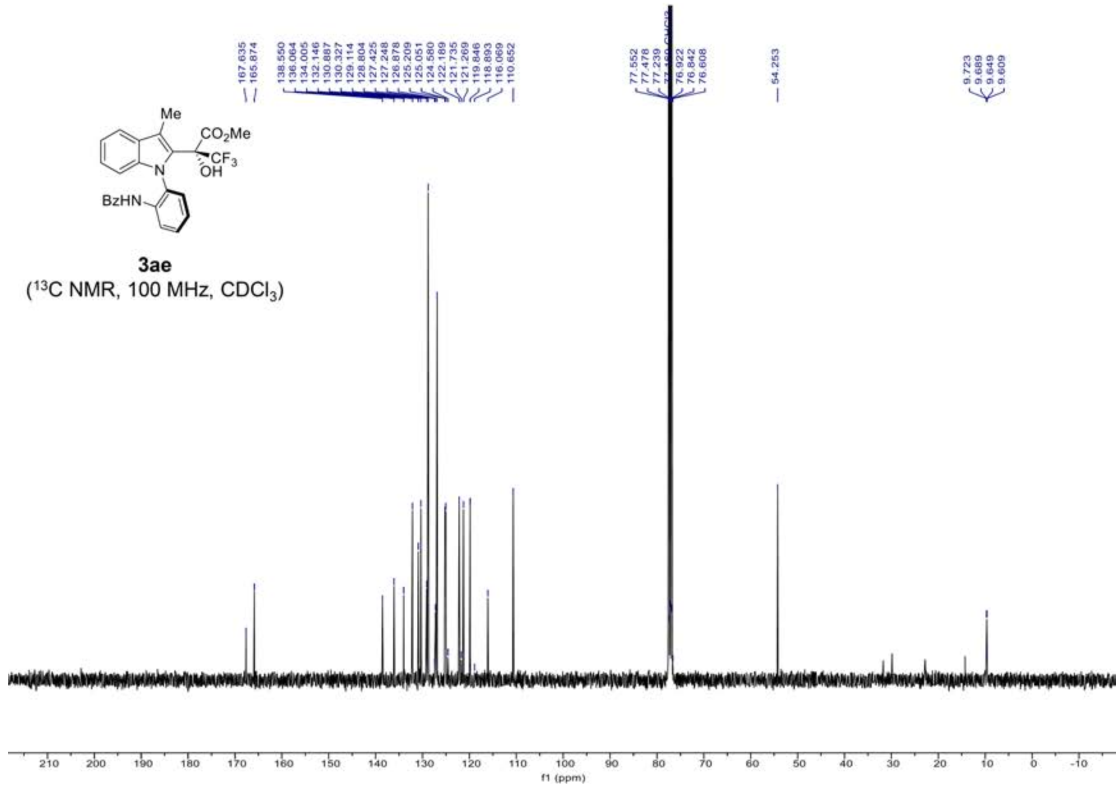

3ae
(^{13}C NMR, 100 MHz, $CDCl_3$)

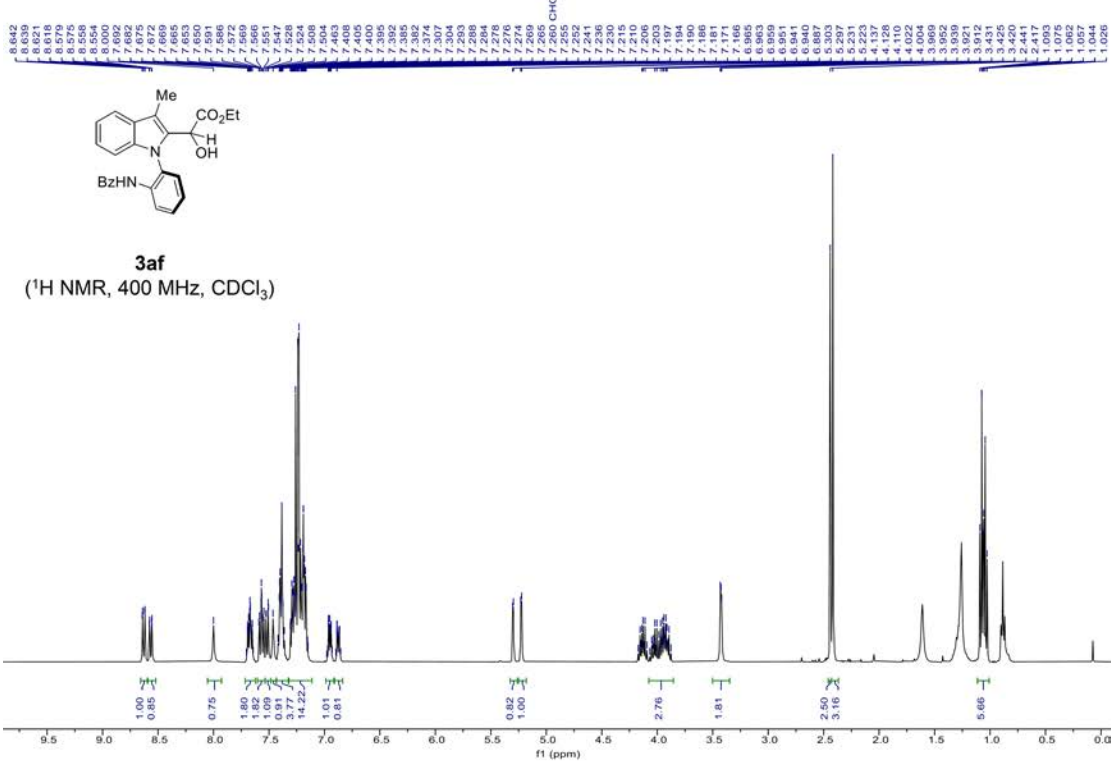
Me
CO2Et
H
OH
N
BzHN
3af
(1H NMR, 400 MHz, CDCl3)
f1 (ppm)

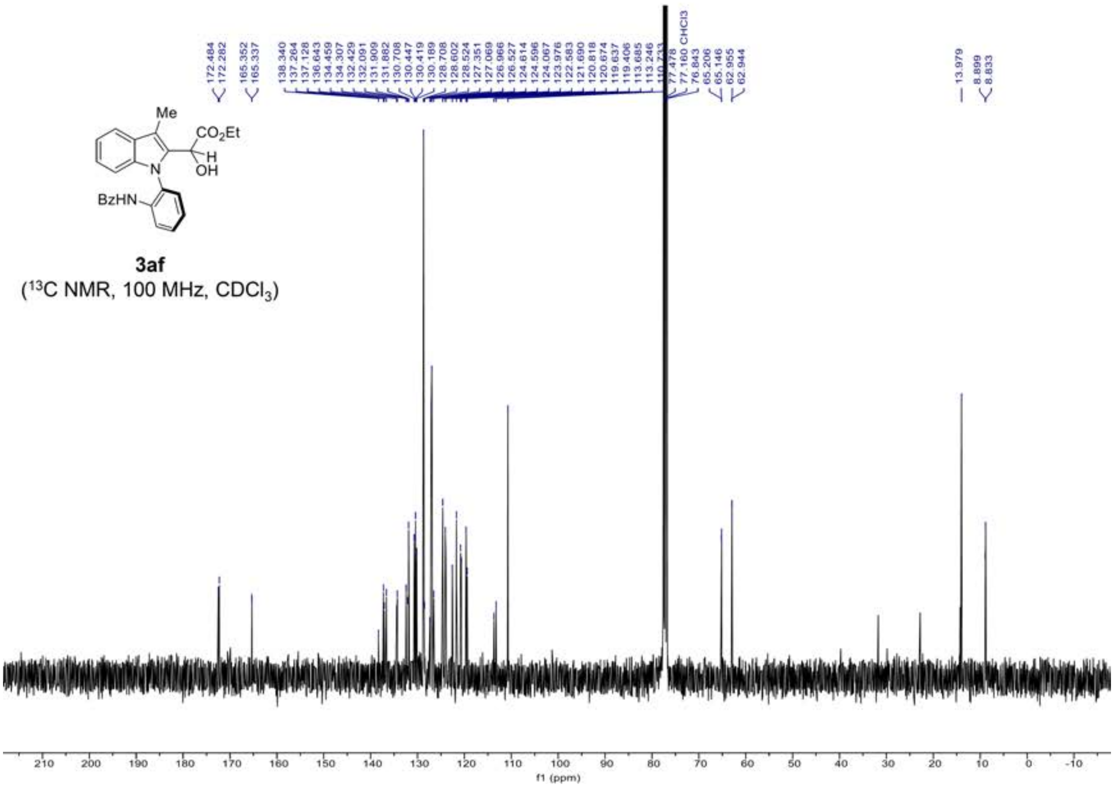
Me
CO2Et
H
OH
N
BzHN
3af
(13C NMR, 100 MHz, CDCl3)
f1 (ppm)

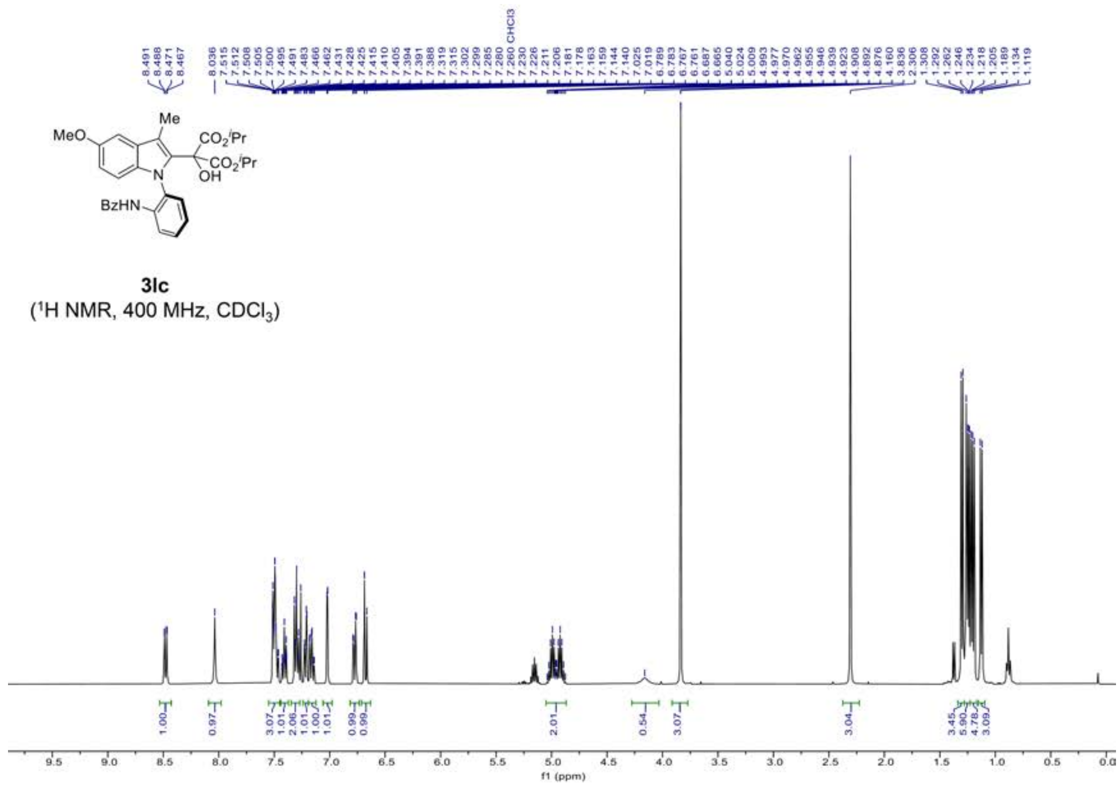

3lc
(^1H NMR, 400 MHz, $CDCl_3$)

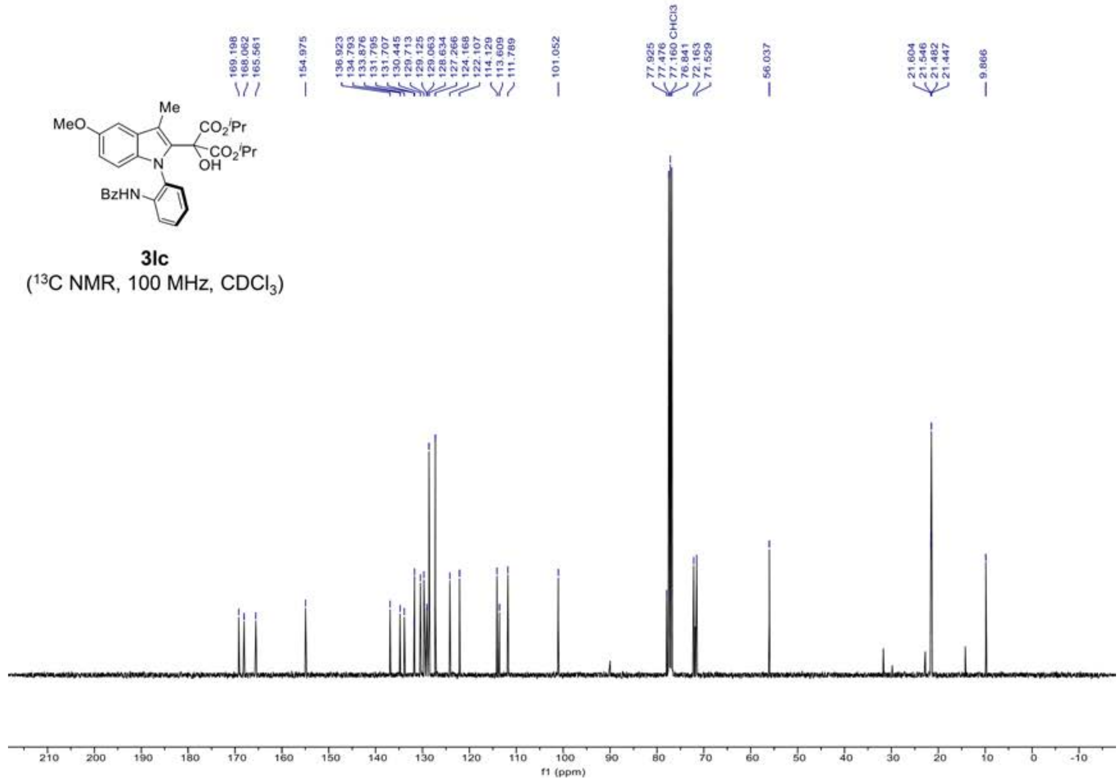

3lc
(^{13}C NMR, 100 MHz, $CDCl_3$)

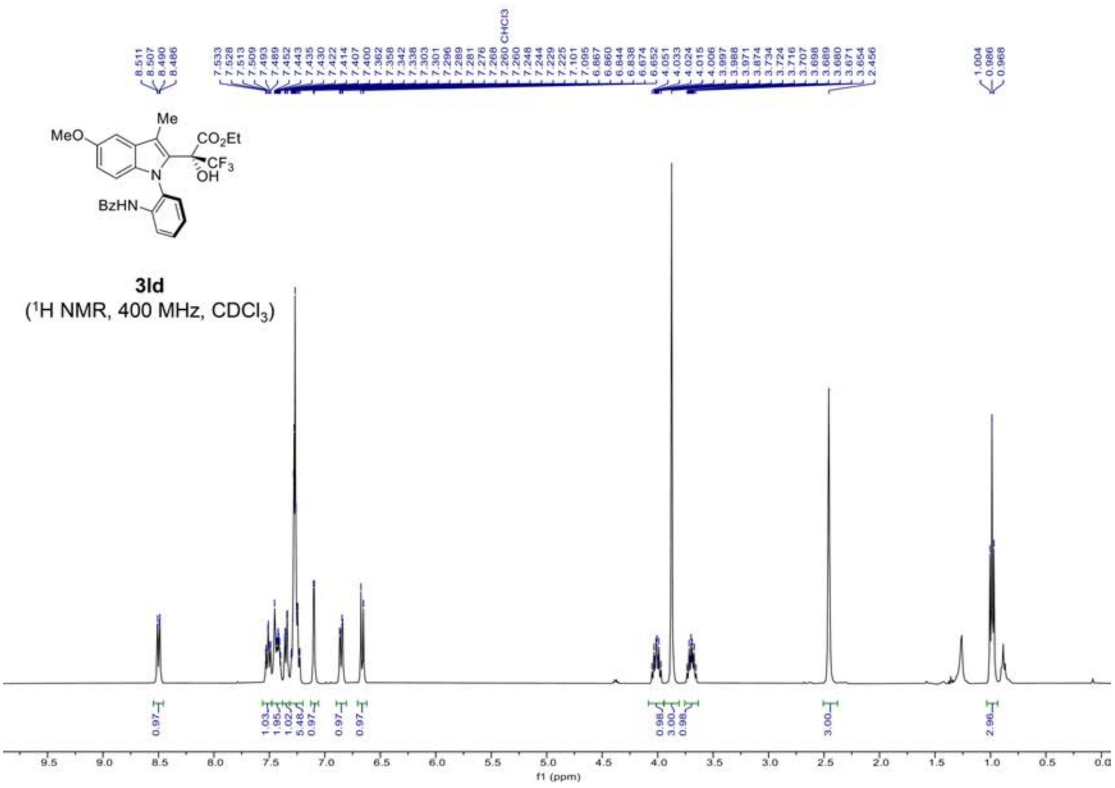

3ld
(^{1}H NMR, 400 MHz, $CDCl_3$)

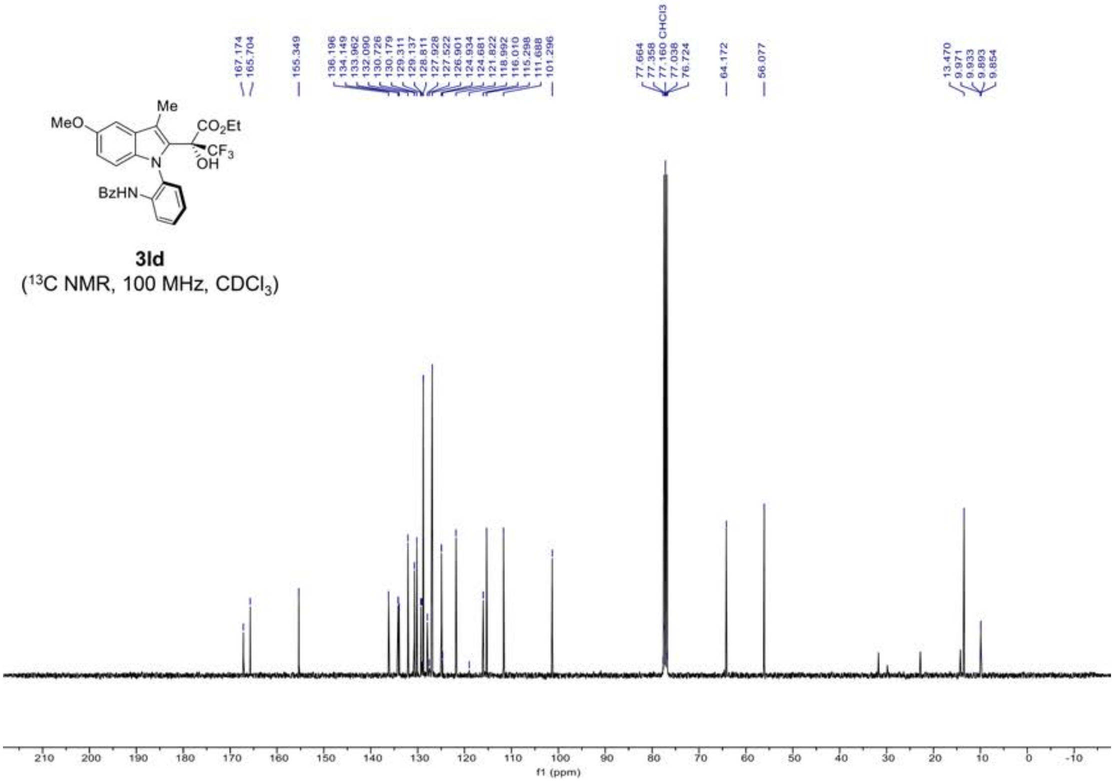

3ld
(^{13}C NMR, 100 MHz, $CDCl_3$)

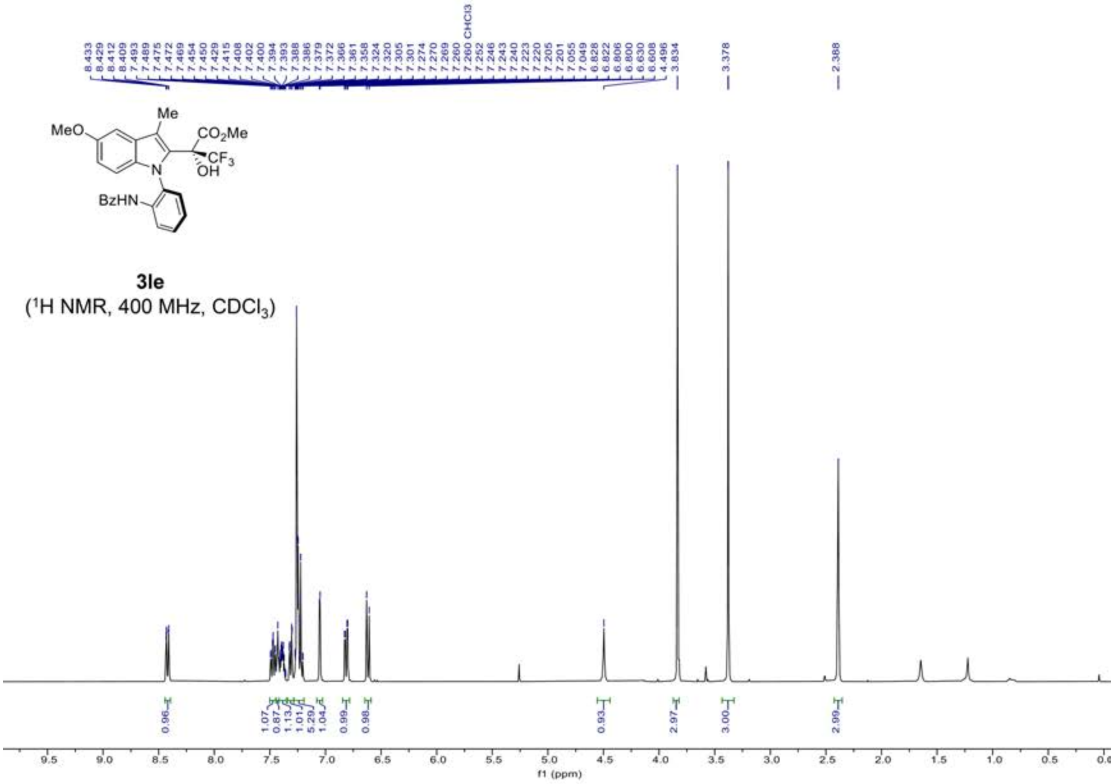

3le
(^{1}H NMR, 400 MHz, $CDCl_3$)

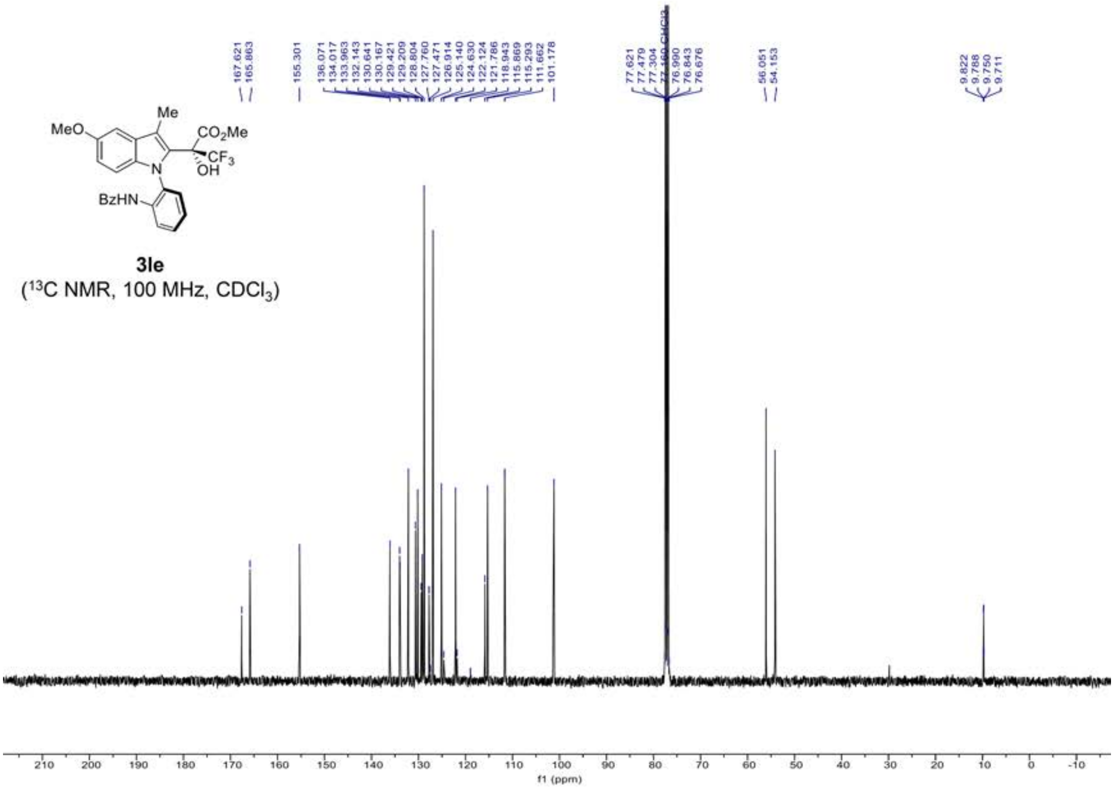

3le
(^{13}C NMR, 100 MHz, $CDCl_3$)

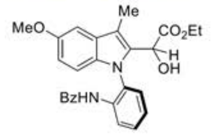

3lf
(^{1}H NMR, 400 MHz, $CDCl_3$)

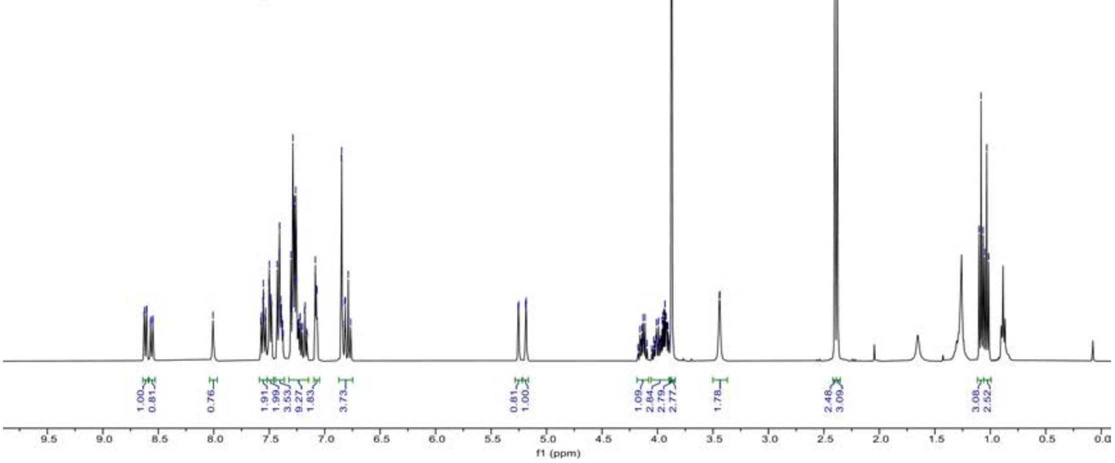

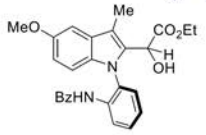

3lf
(^{13}C NMR, 100 MHz, $CDCl_3$)

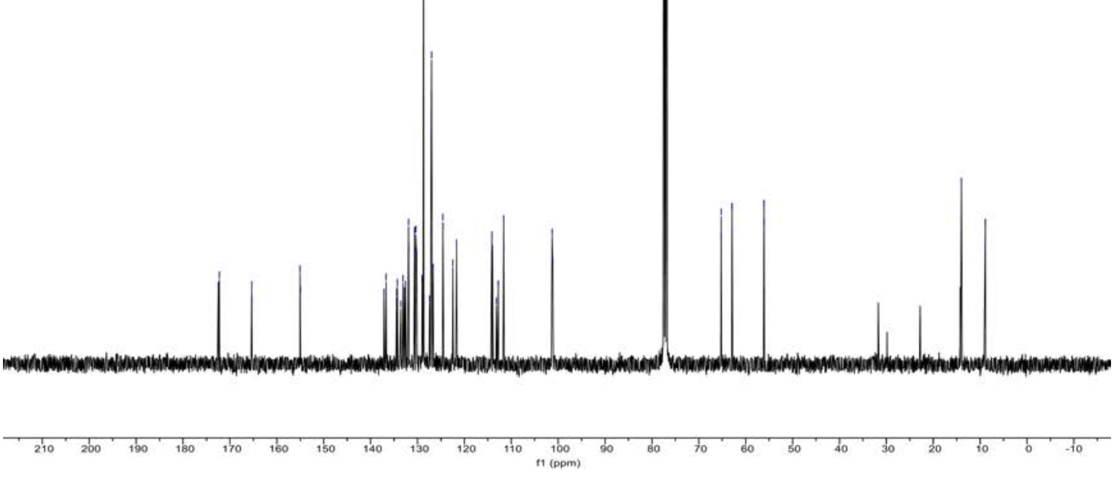

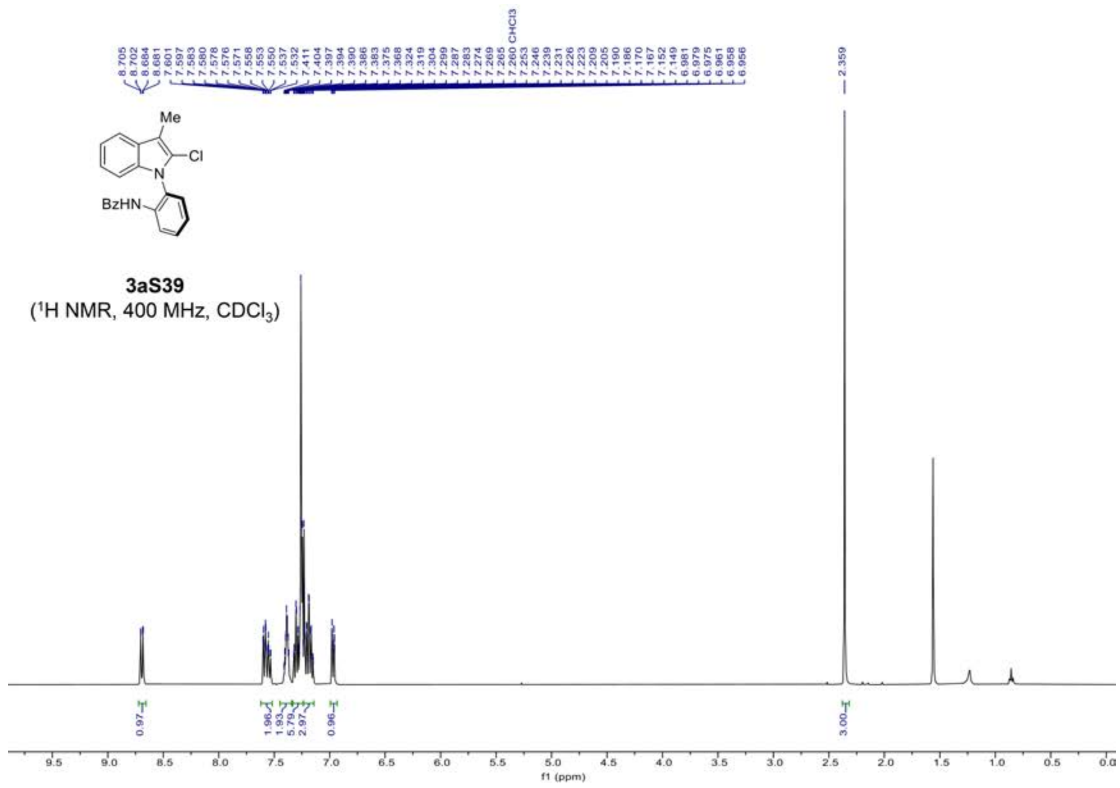
Me
Cl
N
BzHN
3aS39
(1H NMR, 400 MHz, CDCl3)
f1 (ppm)

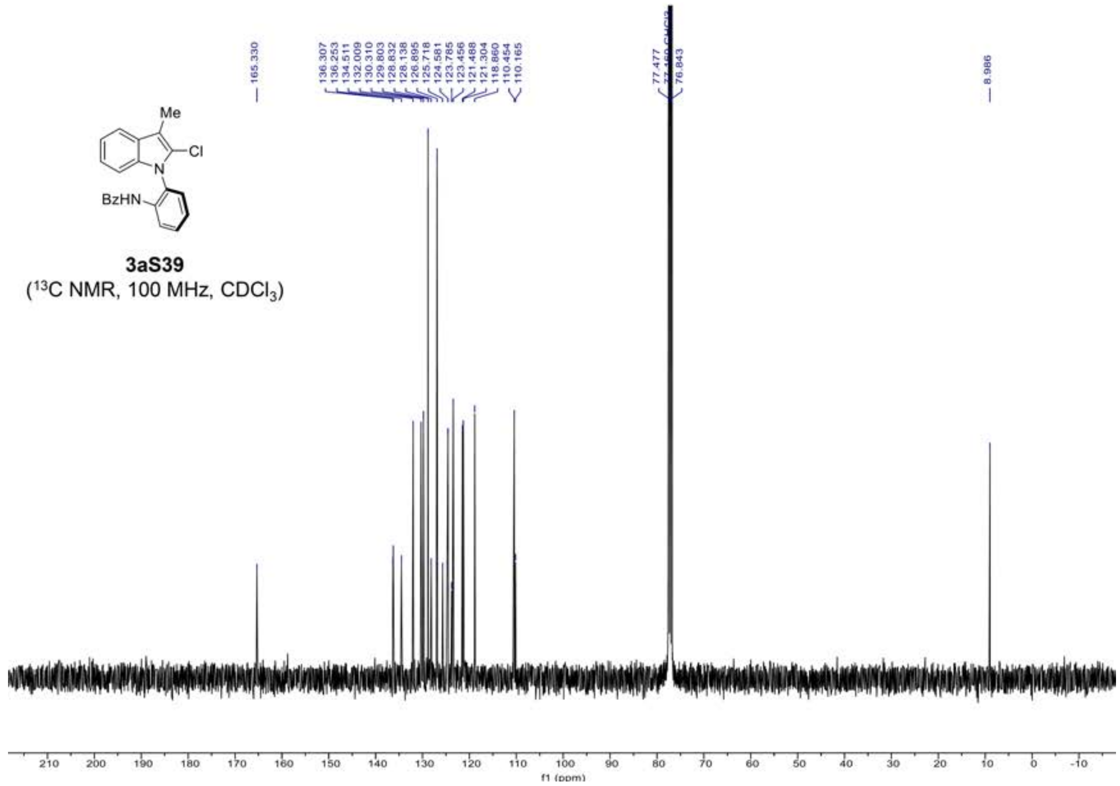
Me
Cl
N
BzHN
3aS39
(13C NMR, 100 MHz, CDCl3)
f1 (ppm)

7.2 HPLC Traces of 3

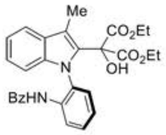

3aa

HPLC conditions:
Chiralpak AD-H, 30% ^{i}PrOH/*n*-Hexane eluent
1.0 mL/min, 254 nm

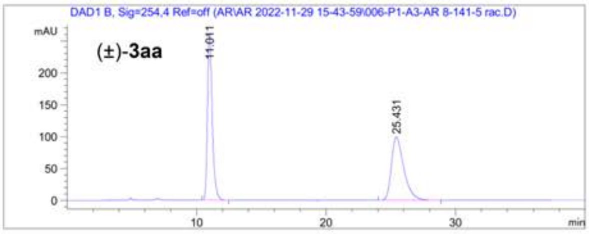

Peak #	RetTime [min]	Type	Width [min]	Area [mAU*s]	Height [mAU]	Area %
1	11.011	BB	0.3843	6632.09033	261.14130	49.9833
2	25.431	BB	1.0051	6636.52295	99.12162	50.0167

Totals : 1.32686e4 360.26292

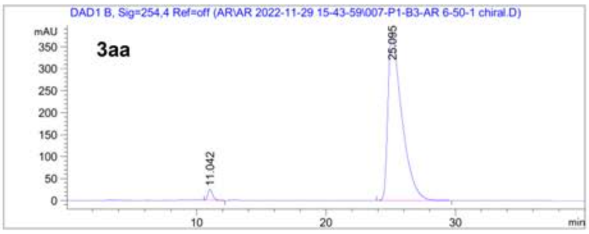

Peak #	RetTime [min]	Type	Width [min]	Area [mAU*s]	Height [mAU]	Area %
1	11.042	BB	0.3824	622.66846	24.84797	2.1468
2	25.095	BB	1.0720	2.83818e4	379.70905	97.8532

Totals : 2.90045e4 404.55702

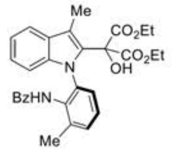

3ba

HPLC conditions:
Chiralpak AD-H, 10% ^{i}PrOH/n-Hexane eluent
1.0 mL/min, 254 nm

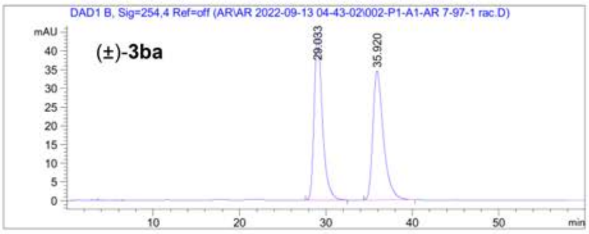

Peak #	RetTime [min]	Type	Width [min]	Area [mAU*s]	Height [mAU]	Area %
1	29.033	BB	0.9861	2930.37012	44.39140	49.9888
2	35.920	BB	1.2501	2931.68018	34.47462	50.0112
Totals :				5862.05029	78.86602	

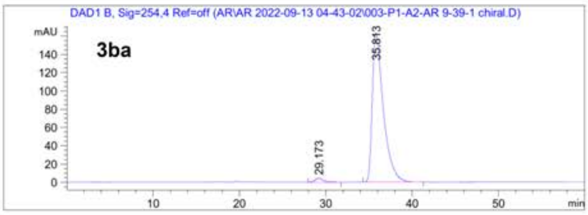

Peak #	RetTime [min]	Type	Width [min]	Area [mAU*s]	Height [mAU]	Area %
1	29.173	BB	0.8272	284.94257	4.36710	1.9218
2	35.813	BB	1.2832	1.45416e4	163.25168	98.0782
Totals :				1.48265e4	167.61878	

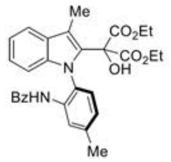

3ca

HPLC conditions:
Chiralpak IC, 30% ^{i}PrOH/n-Hexane eluent
1.0 mL/min, 254 nm

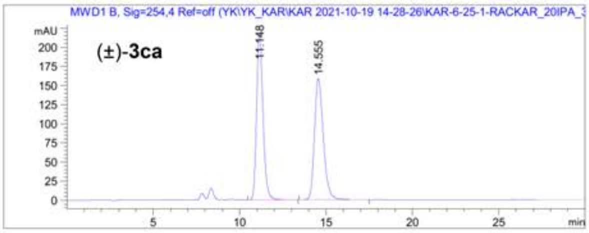

Peak #	RetTime [min]	Type	Width [min]	Area [mAU*s]	Height [mAU]	Area %
1	11.148	BB	0.3980	5647.59277	218.17801	49.8725
2	14.555	BB	0.5489	5676.47266	158.86308	50.1275
Totals :				1.13241e4	377.04109	

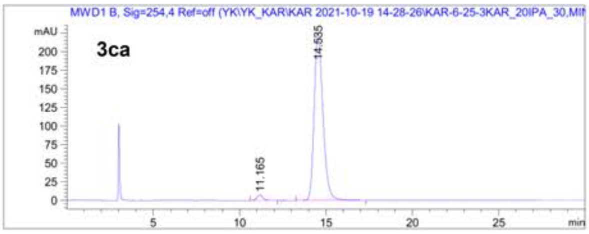

Peak #	RetTime [min]	Type	Width [min]	Area [mAU*s]	Height [mAU]	Area %
1	11.165	BB	0.3946	187.20245	7.17045	2.2912
2	14.535	BB	0.5451	7983.44629	224.35445	97.7088
Totals :				8170.64874	231.52490	

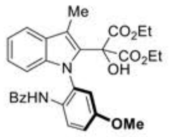

3da

HPLC conditions:
Chiralpak IC, 20% ^{i}PrOH/n-Hexane eluent
1.0 mL/min, 254 nm

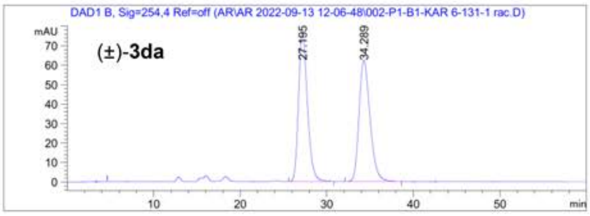

Peak #	RetTime [min]	Type	Width [min]	Area [mAU*s]	Height [mAU]	Area %
1	27.195	BB	1.0988	5460.91406	76.32214	49.9254
2	34.289	BB	1.3379	5477.24219	62.28801	50.0746
Totals :				1.09382e4	138.61014	

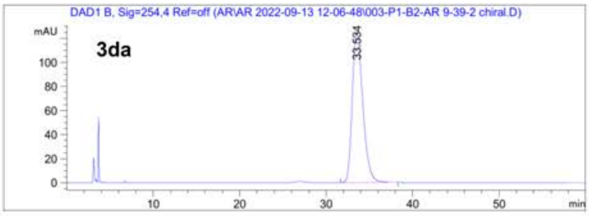

Peak #	RetTime [min]	Type	Width [min]	Area [mAU*s]	Height [mAU]	Area %
1	33.534	BB	1.3125	1.06453e4	124.63554	100.0000
Totals :				1.06453e4	124.63554	

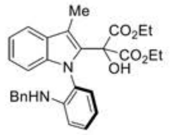

3fa

HPLC conditions:
Chiralpak AD-H, 20% ^{i}PrOH/n-Hexane eluent
1.0 mL/min, 254 nm

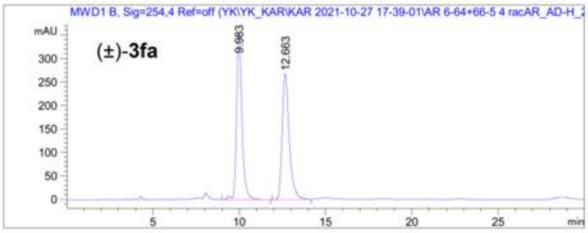

Peak #	RetTime [min]	Type	Width [min]	Area [mAU*s]	Height [mAU]	Area %
1	9.983	VB R	0.3335	7803.21094	354.18506	50.7463
2	12.663	BB	0.4268	7573.69141	267.12903	49.2537

Totals : 1.53769e4 621.31409

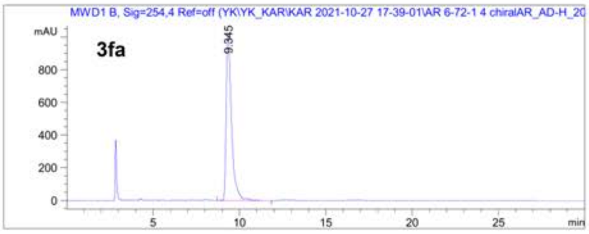

Peak #	RetTime [min]	Type	Width [min]	Area [mAU*s]	Height [mAU]	Area %
1	9.345	BV R	0.3178	2.16740e4	1019.32281	100.0000

Totals : 2.16740e4 1019.32281

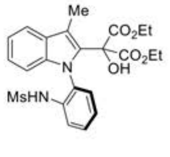

3ga

HPLC conditions:
Chiralpak IC, 20% ^{i}PrOH/n-Hexane eluent
1.0 mL/min, 254 nm

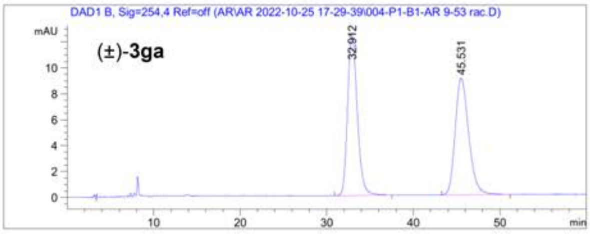

Peak #	RetTime [min]	Type	Width [min]	Area [mAU*s]	Height [mAU]	Area %
1	32.912	BB	1.2040	974.58075	12.52187	49.9694
2	45.531	BB	1.6231	975.77570	9.01258	50.0306
Totals :				1950.35645	21.53445	

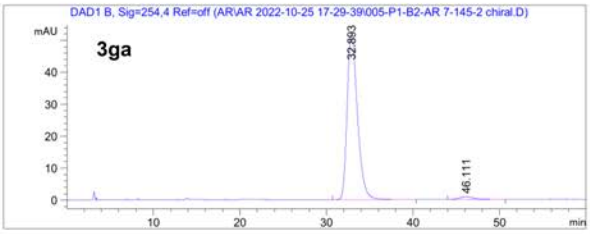

Peak #	RetTime [min]	Type	Width [min]	Area [mAU*s]	Height [mAU]	Area %
1	32.893	BB	1.2561	4318.63525	52.04596	97.7973
2	46.111	BB	1.4601	97.27001	8.57073e-1	2.2027
Totals :				4415.90527	52.90303	

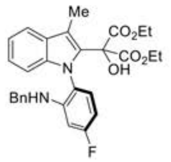

3ha

HPLC conditions:
Chiralpak AD-H, 10% ^{i}PrOH/n-Hexane eluent
1.0 mL/min, 254 nm

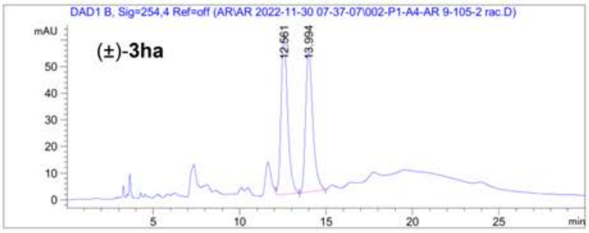

Peak #	RetTime [min]	Type	Width [min]	Area [mAU*s]	Height [mAU]	Area %
1	12.561	VB	0.3979	1588.89197	60.60601	50.7420
2	13.994	BB	0.4332	1542.42249	53.99923	49.2580
Totals :				3131.31445	114.60524	

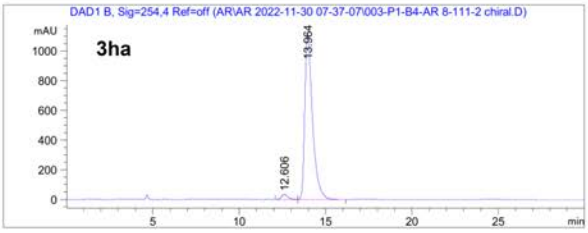

Peak #	RetTime [min]	Type	Width [min]	Area [mAU*s]	Height [mAU]	Area %
1	12.606	BV	0.3908	922.07214	35.53242	2.5939
2	13.964	VB	0.4581	3.46251e4	1121.66321	97.4061
Totals :				3.55472e4	1157.19563	

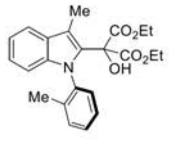

3ia

HPLC conditions:
Chiralpak AD, 2% ^{i}PrOH/n-Hexane eluent
1.0 mL/min, 254 nm

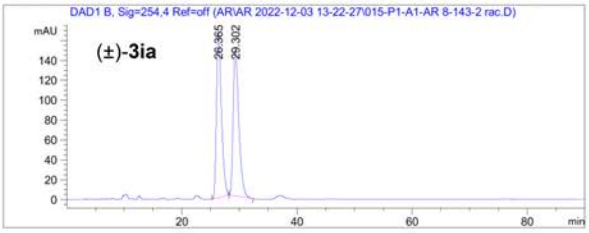

Peak #	RetTime [min]	Type	Width [min]	Area [mAU*s]	Height [mAU]	Area %
1	26.365	BB	0.9176	1.00387e4	165.50928	50.0216
2	29.302	BB	1.0357	1.00300e4	146.33395	49.9784
Totals :				2.00686e4	311.84323	

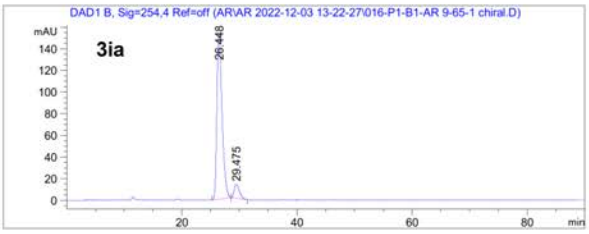

Peak #	RetTime [min]	Type	Width [min]	Area [mAU*s]	Height [mAU]	Area %
1	26.448	BB	0.9420	9545.89453	152.98906	92.0696
2	29.475	BB	0.8932	822.23853	13.01308	7.9304
Totals :				1.03681e4	166.00214	

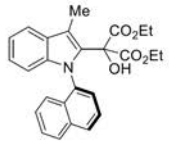

3ja

HPLC conditions:
Chiralpak IC, 20% ^{i}PrOH/n-Hexane eluent
1.0 mL/min, 254 nm

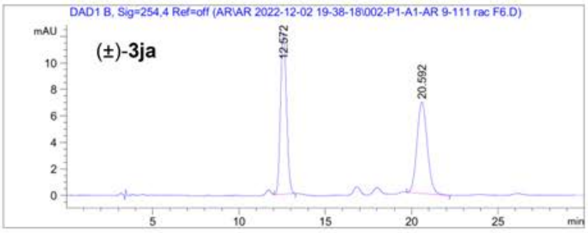

Peak #	RetTime [min]	Type	Width [min]	Area [mAU*s]	Height [mAU]	Area %
1	12.572	BB	0.3791	294.84323	12.15013	50.9172
2	20.592	BB	0.6281	284.22049	6.87803	49.0828
Totals :				579.06372	19.02817	

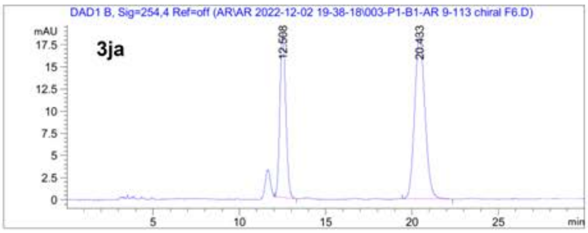

Peak #	RetTime [min]	Type	Width [min]	Area [mAU*s]	Height [mAU]	Area %
1	12.508	BB	0.3668	439.05841	18.50582	36.3721
2	20.433	BB	0.6441	768.07043	18.43517	63.6279
Totals :				1207.12885	36.94099	

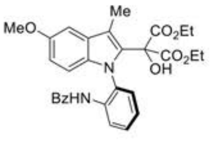

3la

HPLC conditions:
Chiralpak AD-H, 20% ^{i}PrOH/n-Hexane eluent
1.0 mL/min, 254 nm

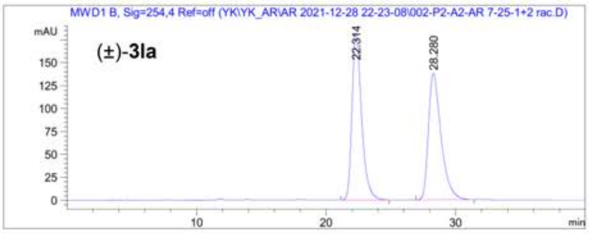

Peak #	RetTime [min]	Type	Width [min]	Area [mAU*s]	Height [mAU]	Area %
1	22.314	BB	0.7841	9356.90625	181.30293	49.9621
2	28.280	BB	1.0236	9371.09570	137.76915	50.0379
Totals :				1.87280e4	319.07208	

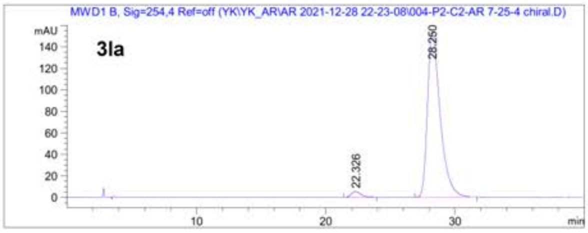

Peak #	RetTime [min]	Type	Width [min]	Area [mAU*s]	Height [mAU]	Area %
1	22.326	BB	0.6102	273.39172	5.36789	2.5477
2	28.250	BB	1.0028	1.04574e4	152.72368	97.4523
Totals :				1.07308e4	158.09157	

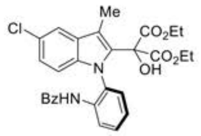

3ma

HPLC conditions:
Chiralpak AD-H, 20% ^{i}PrOH/*n*-Hexane eluent
1.0 mL/min, 254 nm

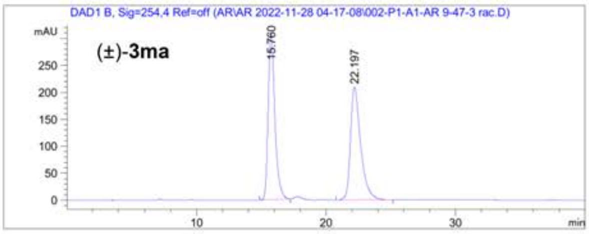

Peak #	RetTime [min]	Type	Width [min]	Area [mAU*s]	Height [mAU]	Area %
1	15.760	BB	0.5442	1.10303e4	307.71103	49.0807
2	22.197	BB	0.8140	1.14435e4	209.25150	50.9193
Totals :				2.24738e4	516.96252	

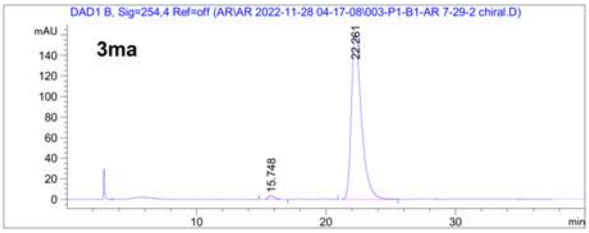

Peak #	RetTime [min]	Type	Width [min]	Area [mAU*s]	Height [mAU]	Area %
1	15.748	VV R	0.5092	130.49812	3.61082	1.4770
2	22.261	BV R	0.8085	8704.57324	161.57310	98.5230
Totals :				8835.07137	165.18392	

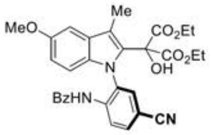

3na

HPLC conditions:
Chiralpak IC, 40% ^{i}PrOH/n-Hexane eluent
1.0 mL/min, 254 nm

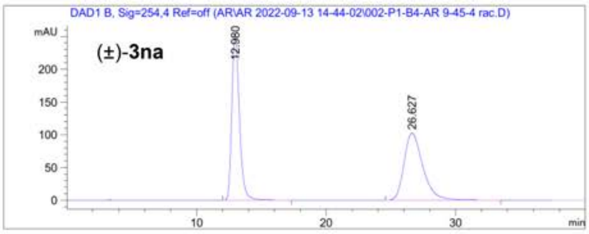

Peak #	RetTime [min]	Type	Width [min]	Area [mAU*s]	Height [mAU]	Area %
1	12.980	BB	0.6213	1.02926e4	253.68626	50.0594
2	26.627	BB	1.4922	1.02682e4	102.36964	49.9406
Totals :				2.05608e4	356.05591	

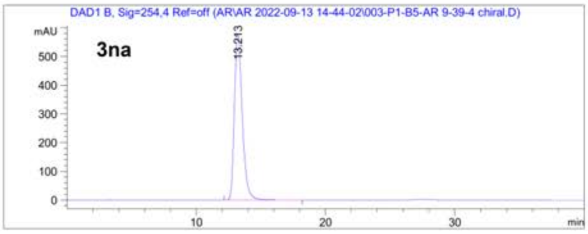

Peak #	RetTime [min]	Type	Width [min]	Area [mAU*s]	Height [mAU]	Area %
1	13.213	BB	0.6489	2.45817e4	579.52771	100.0000
Totals :				2.45817e4	579.52771	

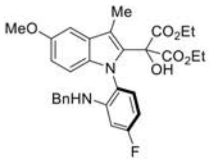

3oa

HPLC conditions:
Chiralpak AD-H, 10% ^{i}PrOH/n-Hexane eluent
1.0 mL/min, 254 nm

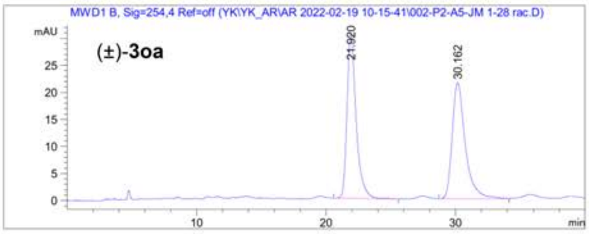

Peak #	RetTime [min]	Type	Width [min]	Area [mAU*s]	Height [mAU]	Area %
1	21.920	BB	0.7492	1489.26758	30.31375	49.7805
2	30.162	BB	1.0568	1502.40356	21.45957	50.2195
Totals :				2991.67114	51.77332	

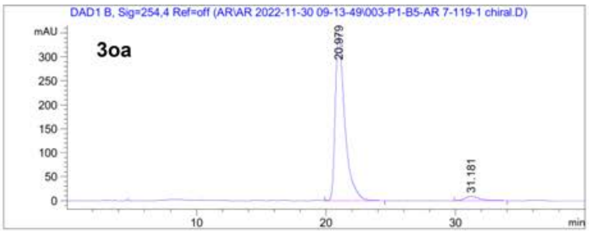

Peak #	RetTime [min]	Type	Width [min]	Area [mAU*s]	Height [mAU]	Area %
1	20.979	BB	0.7575	1.78712e4	348.94617	96.4665
2	31.181	BB	0.9773	654.60822	8.86699	3.5335
Totals :				1.85259e4	357.81315	

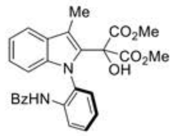

3ab

HPLC conditions:
Chiralpak AD, 30% ^{i}PrOH/*n*-Hexane eluent
1.0 mL/min, 254 nm

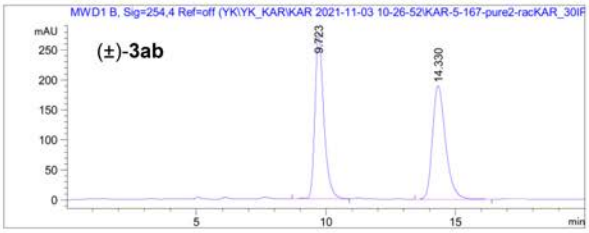

Peak #	RetTime [min]	Type	Width [min]	Area [mAU*s]	Height [mAU]	Area %
1	9.723	VB R	0.3612	6603.34668	275.88583	49.9752
2	14.330	BB	0.5318	6609.90527	189.09416	50.0248

Totals : 1.32133e4 464.98000

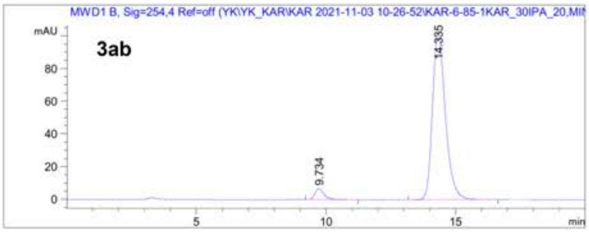

Peak #	RetTime [min]	Type	Width [min]	Area [mAU*s]	Height [mAU]	Area %
1	9.734	BB	0.3792	162.81795	6.70816	4.3978
2	14.335	BB	0.5494	3539.42944	101.85734	95.6022

Totals : 3702.24739 108.56550

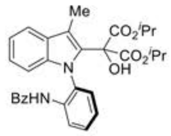

3ac

HPLC conditions:
Chiralpak AD, 30% ^{i}PrOH/*n*-Hexane eluent
1.0 mL/min, 254 nm

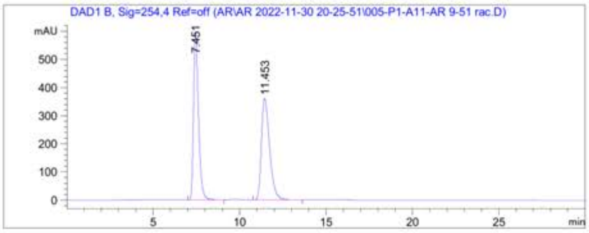

Peak #	RetTime [min]	Type	Width [min]	Area [mAU*s]	Height [mAU]	Area %
1	7.451	BB	0.3029	1.18445e4	593.28436	50.0316
2	11.453	BB	0.5002	1.18295e4	361.12067	49.9684
Totals :				2.36740e4	954.40503	

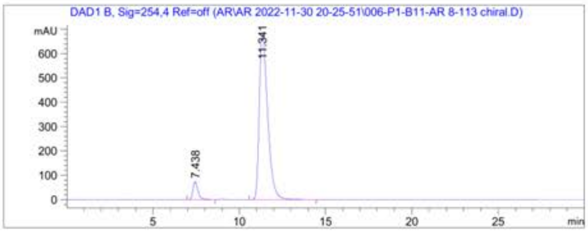

Peak #	RetTime [min]	Type	Width [min]	Area [mAU*s]	Height [mAU]	Area %
1	7.438	BB	0.2968	1460.02209	74.46333	5.9328
2	11.341	BB	0.5169	2.31495e4	683.98108	94.0672
Totals :				2.46095e4	758.44441	

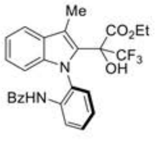

3ad

HPLC conditions:
Chiralpak AD-H, 5% ^{i}PrOH/*n*-Hexane eluent
1.0 mL/min, 254 nm

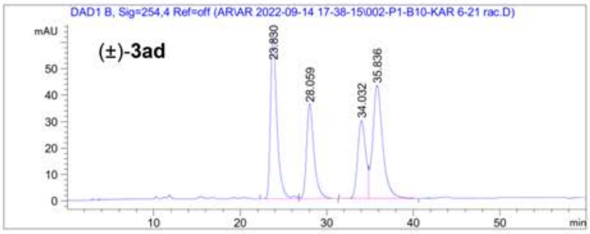

Peak #	RetTime [min]	Type	Width [min]	Area [mAU*s]	Height [mAU]	Area %
1	23.830	BV	0.8033	3229.38501	61.63403	30.1339
2	28.059	VB	0.8999	2142.92383	35.31472	19.9960
3	34.032	BV	1.0149	1902.48877	29.24721	17.7524
4	35.836	VB	1.2026	3441.97363	42.42124	32.1176

Totals : 1.07168e4 168.61720

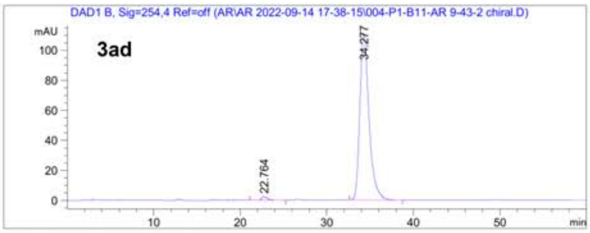

Peak #	RetTime [min]	Type	Width [min]	Area [mAU*s]	Height [mAU]	Area %
1	22.764	BB	0.7279	114.03420	2.37607	1.3737
2	34.277	BB	1.1173	8186.98535	110.88969	98.6263

Totals : 8301.01955 113.26575

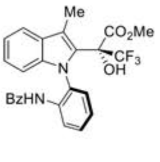

3ae

HPLC conditions:
Chiralpak OD-H, 3% ^{i}PrOH/n-Hexane eluent
1.0 mL/min, 254 nm

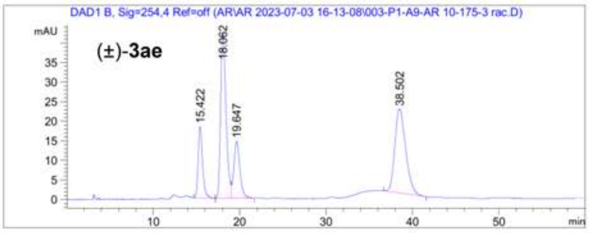

Peak #	RetTime [min]	Type	Width [min]	Area [mAU*s]	Height [mAU]	Area %
1	15.422	BB	0.5431	648.82928	18.23561	12.7698
2	18.062	BV	0.6789	1844.80725	42.12495	36.3082
3	19.647	VB	0.7376	711.49384	14.57362	14.0031
4	38.502	BB	1.3316	1875.83032	21.46410	36.9188
Totals :				5080.96069	96.39827	

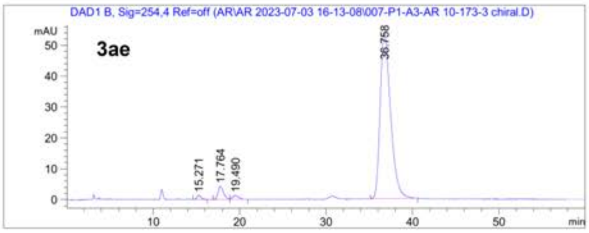

Peak #	RetTime [min]	Type	Width [min]	Area [mAU*s]	Height [mAU]	Area %
1	15.271	BV R	0.4490	45.22667	1.28995	0.9423
2	17.764	BV	0.5737	191.34348	4.27911	3.9868
3	19.490	VV R	0.5711	64.95226	1.35047	1.3533
4	36.758	VV R	1.1901	4497.90088	53.53845	93.7175
Totals :				4799.42329	60.45798	

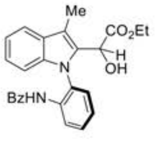

3af

HPLC conditions:
Chiralpak AD-H, 20% ^{i}PrOH/n-Hexane eluent
1.0 mL/min, 254 nm

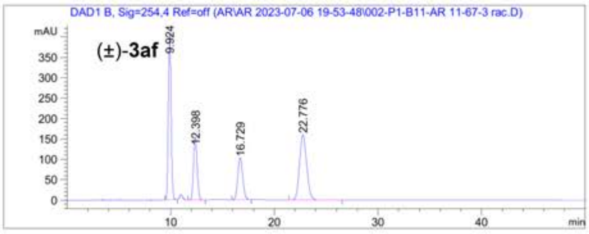

Peak #	RetTime [min]	Type	Width [min]	Area [mAU*s]	Height [mAU]	Area %
1	9.924	BB	0.4375	7589.41553	360.67975	33.8907
2	12.398	BB	0.4717	3747.17798	127.19206	16.7331
3	16.729	BB	0.5997	3424.36035	97.99822	15.2916
4	22.776	BB	0.7609	7632.81348	156.53947	34.0845
Totals :				2.23938e4	742.40951	

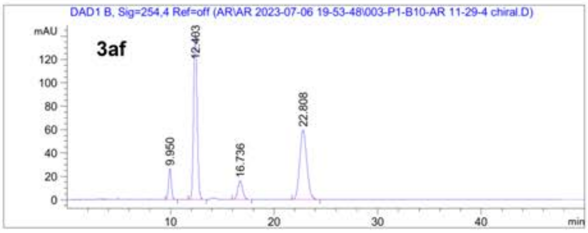

Peak #	RetTime [min]	Type	Width [min]	Area [mAU*s]	Height [mAU]	Area %
1	9.950	BB	0.2874	493.10550	26.46116	6.8124
2	12.403	BB	0.3664	3373.61914	142.37306	46.6072
3	16.736	BB	0.5181	528.27051	15.79828	7.2982
4	22.808	BB	0.7450	2843.40601	59.55651	39.2822
Totals :				7238.40115	244.18902	

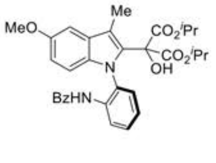

3lc

HPLC conditions:
Chiralpak AD-H, 20% ^{i}PrOH/n-Hexane eluent
1.0 mL/min, 254 nm

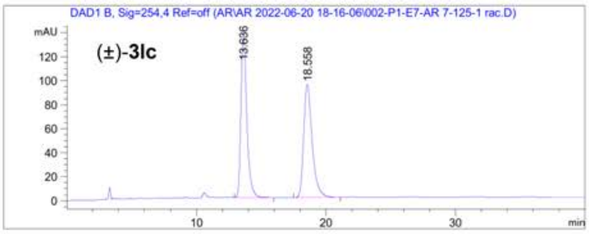

Peak #	RetTime [min]	Type	Width [min]	Area [mAU*s]	Height [mAU]	Area %
1	13.636	BB	0.4772	4293.63330	136.40483	49.9876
2	18.558	BB	0.6898	4295.76807	94.24238	50.0124
Totals :				8589.40137	230.64721	

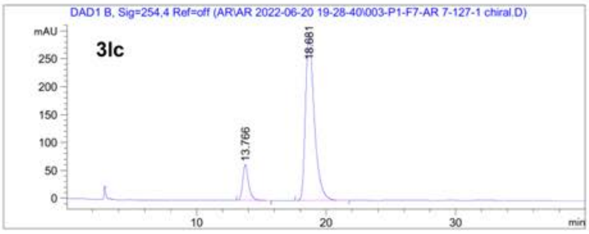

Peak #	RetTime [min]	Type	Width [min]	Area [mAU*s]	Height [mAU]	Area %
1	13.766	BB	0.4775	2000.75671	63.51570	12.4052
2	18.681	BB	0.7109	1.41276e4	299.20087	87.5948
Totals :				1.61283e4	362.71657	

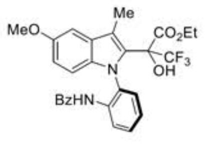

3ld

HPLC conditions:
Chiralpak OD-H, 1% ^{i}PrOH/*n*-Hexane eluent
1.0 mL/min, 254 nm

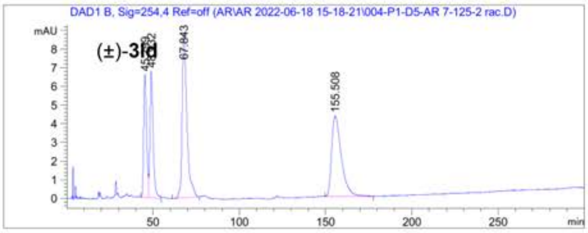

Peak #	RetTime [min]	Type	Width [min]	Area [mAU*s]	Height [mAU]	Area %
1	45.299	BV	1.7647	771.88422	6.59885	15.3361
2	48.932	VB	1.9252	871.43567	6.80874	17.3141
3	67.843	BB	2.8154	1704.98425	8.81953	33.8754
4	155.508	BB	4.6690	1684.80676	4.32861	33.4745
Totals :				5033.11090	26.55572	

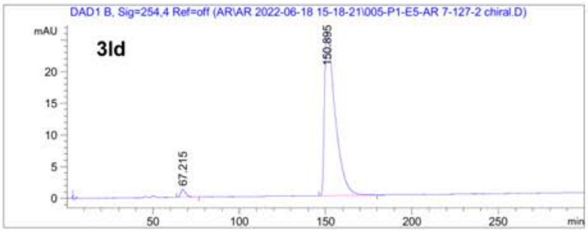

Peak #	RetTime [min]	Type	Width [min]	Area [mAU*s]	Height [mAU]	Area %
1	67.215	BB	2.7641	249.09753	1.27234	2.1959
2	150.895	BB	5.1264	1.10947e4	25.64961	97.8041
Totals :				1.13438e4	26.92195	

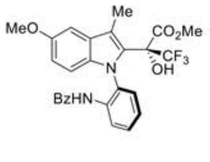

3le

HPLC conditions:
Chiralpak OD-H, 3% ^{i}PrOH/n-Hexane eluent
1.0 mL/min, 254 nm

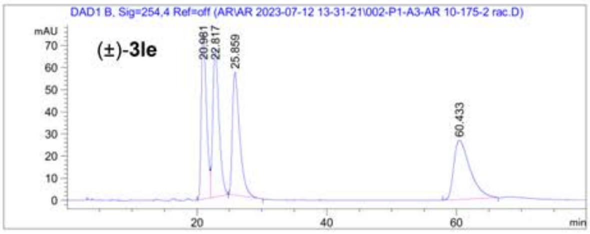

Peak #	RetTime [min]	Type	Width [min]	Area [mAU*s]	Height [mAU]	Area %
1	20.981	BV	0.8445	4168.39502	74.99114	23.6308
2	22.817	VB	0.9938	4497.41162	67.11131	25.4961
3	25.859	BB	1.1471	4334.50586	55.62824	24.5725
4	60.433	BB	2.0385	4639.32324	26.73566	26.3006
Totals :				1.76396e4	224.46635	

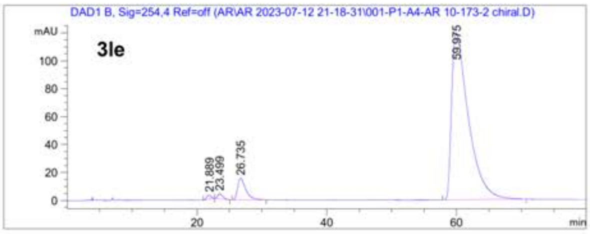

Peak #	RetTime [min]	Type	Width [min]	Area [mAU*s]	Height [mAU]	Area %
1	21.889	BB	0.5983	143.04572	2.88573	0.6151
2	23.499	BB	0.6797	206.50272	3.61918	0.8879
3	26.735	BB	1.0472	1364.93640	15.44211	5.8689
4	59.975	BB	2.4287	2.15427e4	119.30090	92.6281
Totals :				2.32572e4	141.24792	

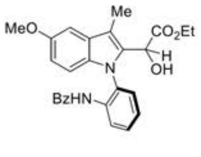

3lf

HPLC conditions:
Chiralpak AD-H, 20% ^{i}PrOH/n-Hexane eluent
1.0 mL/min, 254 nm

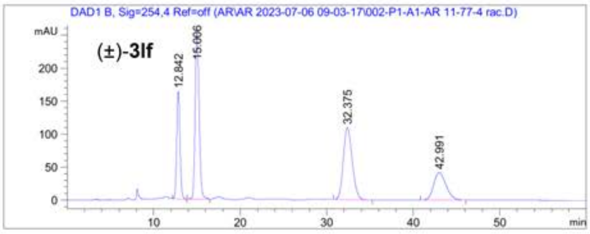

Peak #	RetTime [min]	Type	Width [min]	Area [mAU*s]	Height [mAU]	Area %
1	12.842	BB	0.3992	4198.03271	162.62170	17.3184
2	15.006	BB	0.4922	8008.94238	251.01585	33.0397
3	32.375	BB	1.0969	7851.12891	109.18524	32.3887
4	42.991	BB	1.3906	4182.24219	42.11515	17.2532
Totals :				2.42403e4	564.93795	

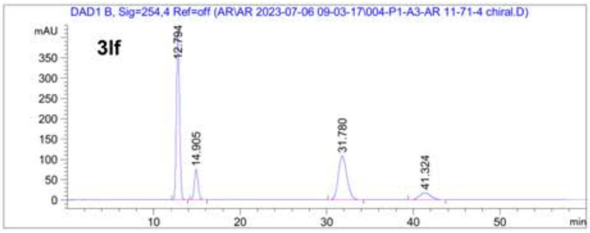

Peak #	RetTime [min]	Type	Width [min]	Area [mAU*s]	Height [mAU]	Area %
1	12.794	BB	0.3886	1.03092e4	411.01089	47.9142
2	14.905	BB	0.4650	2233.69360	74.24036	10.3816
3	31.780	BB	1.0688	7428.59912	108.22347	34.5260
4	41.324	BB	1.1960	1544.45630	17.09507	7.1782
Totals :				2.15159e4	610.56979	

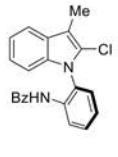

3aS39

HPLC conditions:
Chiralpak AD-H, 10% ^{i}PrOH/n-Hexane eluent
1.0 mL/min, 254 nm

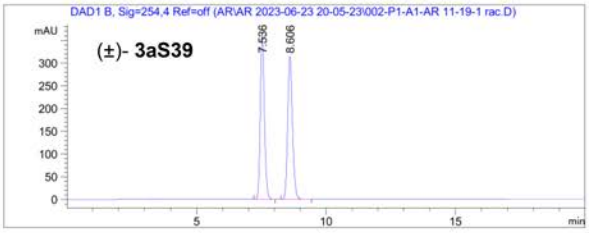

Peak #	RetTime [min]	Type	Width [min]	Area [mAU*s]	Height [mAU]	Area %
1	7.536	BB	0.1717	4073.14380	366.34973	49.9816
2	8.606	BB	0.1994	4076.15063	314.08765	50.0184
Totals :				8149.29443	680.43738	

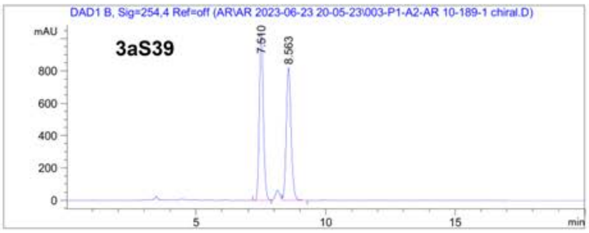

Peak #	RetTime [min]	Type	Width [min]	Area [mAU*s]	Height [mAU]	Area %
1	7.510	BV	0.1687	1.13842e4	1032.29614	51.6732
2	8.563	VB	0.1995	1.06470e4	819.81012	48.3268
Totals :				2.20312e4	1852.10626	

8 Characterization and Spectra of 5

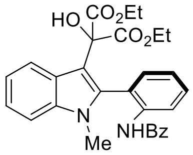

Diethyl 2-(2-(2-Benzamidophenyl)-1-methyl-1*H*-indol-3-yl)-2-hydroxymalonate (5aa) was synthesized by following Procedure 22 from **4a**. The crude material was purified by normal-phase column chromatography using an eluent of 10% EtOAc/CH_2Cl_2 to give **5aa** (99% yield).

^{1}H NMR (400 MHz, $CDCl_3$) δ 8.40–8.26 (m, 2H), 7.64 (dt, J = 8.0, 1.0 Hz, 1H), 7.62–7.58 (m, 2H), 7.55 (ddd, J = 8.5, 7.3, 1.8 Hz, 1H), 7.44–7.36 (m, 1H), 7.36–7.30 (m, 2H), 7.30–7.22 (m, 4H), 7.17 (ddd, J = 8.0, 6.7, 1.3 Hz, 1H), 4.20 (s, 1H), 4.04 (ddq, J = 12.0, 10.8, 7.1 Hz, 2H), 3.83 (dq, J = 10.8, 7.1 Hz, 1H), 3.67 (dq, J = 10.8, 7.1 Hz, 1H), 3.33 (s, 3H), 1.11 (dt, J = 9.6, 7.2 Hz, 6H).
^{13}C NMR (100 MHz, $CDCl_3$) δ 170.4, 170.0, 165.7, 138.4, 137.1, 134.3, 134.1, 132.1, 131.9, 130.7, 128.7 (2C), 127.4 (2C), 125.9, 124.3, 123.1, 122.93, 122.86, 120.51, 120.45, 111.1, 109.9, 78.3, 63.02, 62.99, 30.2, 13.88, 13.85.
IR (FT-ATR, cm^{-1}, $CHCl_3$) ν_{max} 3930, 3861, 3757, 3737, 3680, 3656, 3483, 3305, 3016, 2985, 2931, 2607, 2403, 2376, 2353, 2318, 1955, 1886, 1855, 1813, 1728, 1612, 1574, 1531, 1466, 1350, 1219, 1161, 1134, 1095, 1061, 1038, 926, 860, 744, 667.
HRMS (EI) m/z: $[M]^+$ Calcd for $C_{29}H_{28}N_2O_6$ 500.1947; found 500.1946.
Optical: $[a]^{20}_D$ = –20.1° (c = 1.02, $CHCl_3$, 83% e.e.)
HPLC (Chiralpak AD-H, ^{i}PrOH/n-hexane = 20/80, flow rate = 1.0 mL/min, λ = 254 nm) t_R = 23.6 min (major), 20.8 min (minor)

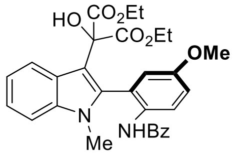

Diethyl 2-(2-(2-Benzamido-5-methoxyphenyl)-1-methyl-1*H*-indol-3-yl)-2-hydroxymalonate (5ba) was synthesized by following Procedure 22 from **4b**. The crude material was purified by normal-phase column chromatography using an eluent of 10% EtOAc/CH_2Cl_2 to give **5ba** (95% yield).

^{1}H NMR (400 MHz, $CDCl_3$) δ 8.21 (s, 1H), 8.12 (d, J = 9.0 Hz, 1H), 7.63 (d, J = 8.1 Hz, 1H), 7.60–7.54 (m, 2H), 7.43–7.36 (m, 1H), 7.34–7.22 (m, 6H), 7.20–7.13 (m, 2H), 7.09 (dd, J = 9.0, 3.0 Hz, 1H), 6.88 (d, J = 3.0 Hz, 1H), 4.08 (ddq, J = 19.1, 10.7, 7.1 Hz, 2H), 3.92–3.81 (m, 4H), 3.76 (dq, J = 10.8, 7.2 Hz, 1H), 3.36 (s, 3H), 1.13 (dt, J = 10.4, 7.1 Hz, 6H).
^{13}C NMR (100 MHz, $CDCl_3$) δ 170.4, 170.1, 165.8, 156.4, 137.1, 134.4, 134.3, 131.7, 131.4, 129.2, 128.7 (2C), 128.4, 127.3 (2C), 125.9, 125.2, 125.0, 122.8, 120.5, 120.4, 117.4, 115.9, 110.8, 109.9, 78.4, 63.1, 63.0, 55.8, 30.3, 13.88 (2C).
IR (FT-ATR, cm^{-1}, $CHCl_3$) ν_{max} 3950, 3903, 3865, 3845, 3807, 3780, 3730, 3699, 3626, 3595, 3456, 3374, 3163, 3059, 2985, 2939, 2839, 2630, 2360, 2337, 2295, 2268, 2133,

2067, 1990, 1898, 1809, 1736, 1662, 1612, 1516, 1466, 1442, 1415, 1369, 1300, 1277, 1246, 1215, 1099, 1065, 1034, 921, 807, 864, 822, 748, 710, 671.
HRMS (EI) *m*/*z*: $[M]^+$ Calcd for $C_{30}H_{30}N_2O_7$ 530.2053; found 530.2053.
Optical: $[a]^{20}_D$ = –31.8º (*c* = 2.04, $CHCl_3$, 96% e.e.)
HPLC (Chiralpak AD-H, ^{i}PrOH/*n*-hexane = 10/90, flow rate = 1.0 mL/min, λ = 254 nm) t_R = 19.4 min (major), 26.2 min (minor)

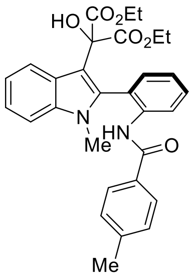

Diethyl 2-Hydroxy-2-(1-methyl-2-(2-(4-methylbenzamido)phenyl)-1*H*-indol-3-yl)malonate (5ca) was synthesized by following Procedure 22 from **4c**. The crude material was purified by normal-phase column chromatography using an eluent of 10% EtOAc/CH_2Cl_2 to give **5ca** (99% yield).

^{1}H NMR (400 MHz, $CDCl_3$) δ 8.34 (d, *J* = 8.3 Hz, 1H), 8.24 (s, 1H), 7.64 (d, *J* = 8.0 Hz, 1H), 7.54 (td, *J* = 7.9, 1.8 Hz, 1H), 7.51–7.42 (m, 2H), 7.38–7.20 (m, 4H), 7.17 (ddd, *J* = 8.1, 6.6, 1.5 Hz, 1H), 7.07 (d, *J* = 7.9 Hz, 2H), 4.04 (ddq, *J* = 14.2, 10.8, 7.2 Hz, 2H), 3.85 (dq, *J* = 10.7, 7.1 Hz, 1H), 3.65 (dq, *J* = 10.7, 7.1 Hz, 1H), 3.32 (s, 3H), 2.30 (s, 3H), 1.11 (td, *J* = 7.1, 5.5 Hz, 6H).
^{13}C NMR (100 MHz, $CDCl_3$) δ 170.4, 170.1, 165.7, 142.5, 138.5, 137.1, 134.1, 132.1, 131.5, 130.7, 129.4 (2C), 127.4 (2C), 126.0, 124.2, 122.91, 122.86, 122.75, 120.51, 120.48, 111.2, 109.9, 78.3, 63.02, 62.98, 30.2, 21.5, 13.90, 13.86.
IR (FT-ATR, cm^{-1}, $CHCl_3$) ν_{max} 3926, 3899, 3869, 3838, 3780, 3730, 3703, 3626, 3599, 3471, 3394, 3163, 3055, 2981, 2935, 2623, 2407, 2360, 2333, 2299, 2268, 1932, 1739, 1670, 1612, 1581, 1527, 1504, 1450, 1369, 1300, 1250, 1219, 1161, 1099, 1065, 1038, 922, 860, 795, 748, 663.
HRMS (EI) *m*/*z*: $[M]^+$ Calcd for $C_{30}H_{30}N_2O_6$ 514.2104; found 514.2100.
Optical: $[a]^{20}_D$ = –21.4º (*c* = 2.04, $CHCl_3$, 87% e.e.)
HPLC (Chiralpak AD-H, ^{i}PrOH/*n*-hexane = 20/80, flow rate = 1.0 mL/min, λ = 254 nm) t_R = 69.1 min (major), 33.8 min (minor)

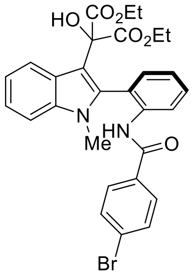

Diethyl 2-(2-(2-(4-Bromobenzamido)phenyl)-1-methyl-1*H*-indol-3-yl)-2-hydroxymalonate (5da) was synthesized by following Procedure 22 from **4d**. The

crude material was purified by normal-phase column chromatography using an eluent of 10% EtOAc/CH_2Cl_2 to give **5da** (99% yield).

^{1}H NMR (400 MHz, $CDCl_3$) δ 8.39 (s, 1H), 8.26 (dd, *J* = 8.3, 1.1 Hz, 1H), 7.63 (dt, *J* = 8.1, 1.0 Hz, 1H), 7.55 (ddd, *J* = 8.5, 7.1, 2.0 Hz, 1H), 7.51–7.45 (m, 2H), 7.44–7.37 (m, 2H), 7.36–7.27 (m, 4H), 7.18 (ddd, *J* = 8.1, 6.6, 1.6 Hz, 1H), 4.13–3.99 (m, 2H), 3.86 (dq, *J* = 10.8, 7.2 Hz, 1H), 3.71 (dq, *J* = 10.8, 7.2 Hz, 1H), 3.31 (s, 3H), 1.14 (td, *J* = 7.1, 1.5 Hz, 6H).
^{13}C NMR (100 MHz, $CDCl_3$) δ 170.3, 170.0, 164.9, 138.2, 137.1, 134.1, 133.1, 132.1, 131.9 (2C), 130.7, 129.1 (2C), 126.7, 125.8, 124.5, 123.4, 123.2, 123.0, 120.6, 120.5, 111.1, 109.9, 78.3, 63.11, 63.08, 30.3, 13.92, 13.89.
IR (FT-ATR, cm^{-1}, $CHCl_3$) ν_{max} 3988, 3926, 2899, 3865, 3842, 3822, 3799, 3780, 3730, 3703, 3626, 3595, 3456, 3390, 3163, 3055, 2981, 2931, 2854, 2607, 2407, 2360, 2337, 2299, 2268, 1932, 1894, 1855, 1736, 1674, 1612, 1585, 1520, 1454, 1369, 1304, 1254, 1219, 1161, 1099, 1068, 1038, 1014, 922, 899, 852, 798, 748, 710, 660.
HRMS (EI) *m*/*z*: $[M]^+$ Calcd for $C_{29}H_{27}BrN_2O_6$ 578.1053; found 578.1055.
Optical: $[a]^{20}_D$ = –19.9° (*c* = 0.96, $CHCl_3$, 82% e.e.)
HPLC (Chiralpak AD-H, ^{i}PrOH/*n*-hexane = 20/80, flow rate = 1.0 mL/min, λ = 254 nm) t_R = 19.3 min (major), 29.9 min (minor)

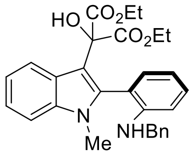

Diethyl 2-(2-(2-(Benzylamino)phenyl)-1-methyl-1*H*-indol-3-yl)-2-hydroxymalonate (5ea) was synthesized by following Procedure 22 from **4e**. The crude material was purified by normal-phase column chromatography using an eluent of 10% EtOAc/CH_2Cl_2 to give **5ea** (99% yield).

^{1}H NMR (400 MHz, $CDCl_3$) δ 7.63 (d, *J* = 8.0 Hz, 1H), 7.45–7.18 (m, 8H), 7.14 (ddd, *J* = 7.5, 5.7, 1.5 Hz, 2H), 6.75 (t, *J* = 7.4 Hz, 1H), 6.62 (d, *J* = 8.2 Hz, 1H), 4.30 (s, 2H), 4.00 (dp, *J* = 10.5, 7.3 Hz, 2H), 3.80 (dq, *J* = 10.7, 7.2 Hz, 1H), 3.66 (dq, *J* = 10.7, 7.2 Hz, 1H), 3.43 (s, 3H), 1.13 (td, *J* = 7.1, 1.4 Hz, 6H).
^{13}C NMR (100 MHz, $CDCl_3$) δ 170.32, 170.26, 148.1, 139.5, 137.1, 135.5, 132.6, 130.8, 128.6 (2C), 127.2 (2C), 127.1, 126.3, 122.3, 120.5, 120.1, 116.6, 116.0, 110.7, 110.6, 109.7, 78.3, 62.6, 62.6, 48.1, 30.1, 13.9.
IR (FT-ATR, cm^{-1}, $CHCl_3$) ν_{max} 3907, 3880, 3807, 3780, 3757, 3737, 3680, 3652, 3621, 3487, 3406, 3055, 3024, 2981, 2935, 2904, 2611, 2411, 2372, 2314, 1921, 1878, 1813, 1728, 1605, 1581, 1504, 1462, 1365, 1323, 1254, 1211, 1161, 1130, 1092, 1065, 1030, 922, 860, 741, 702, 660.
HRMS (EI) *m*/*z*: $[M]^+$ Calcd for $C_{29}H_{30}N_2O_5$ 486.2155; found 486.2157.
Optical: $[a]^{20}_D$ = –61.5° (*c* = 2.37, $CHCl_3$, 90% e.e.)
HPLC (Chiralpak AD-H, ^{i}PrOH/*n*-hexane = 20/80, flow rate = 1.0 mL/min, λ = 254 nm) t_R = 13.5 min (major), 18.2 min (minor)

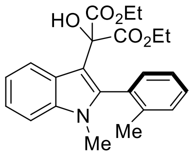

Diethyl 2-Hydroxy-2-(1-methyl-2-(*o*-tolyl)-1*H*-indol-3-yl)malonate (5fa) was synthesized by following Procedure 22 from **4f**. The crude material was purified by normal-phase column chromatography using an eluent of 10% EtOAc/CH_2Cl_2 to give **5fa** (96% yield).

^{1}H NMR (400 MHz, $CDCl_3$) δ 7.64 (d, *J* = 8.0 Hz, 1H), 7.44–7.20 (m, 6H), 7.15 (ddd, *J* = 8.0, 7.0, 1.1 Hz, 1H), 4.12–3.91 (m, 3H), 3.79 (dq, *J* = 10.8, 7.1 Hz, 1H), 3.61 (dq, *J* = 10.8, 7.1 Hz, 1H), 3.33 (s, 3H), 2.14 (s, 3H), 1.15 (t, *J* = 7.1 Hz, 6H).
^{13}C NMR (100 MHz, $CDCl_3$) δ 170.4, 170.3, 139.6, 137.9, 136.7, 132.1, 130.9, 129.7, 129.4, 126.4, 125.4, 122.1, 120.6, 120.1, 109.5, 109.1, 78.2, 62.51, 62.45, 29.9, 20.0, 13.86, 13.84.
IR (FT-ATR, cm^{-1}, $CHCl_3$) ν_{max} 3988, 3823, 3899, 3838, 3807, 3780, 3730, 3699, 3626, 3487, 3217, 3055, 2981, 2927, 2738, 2607, 2480, 2407, 2360, 2333, 2299, 2268, 1928, 1886, 1728, 1608, 1554, 1466, 1365, 1215, 1161, 1130, 1095, 1068, 1034, 922, 860, 798, 740, 706, 660.
HRMS (EI) *m*/*z*: [M]$^+$ Calcd for C23H25NO5 395.1733; found 395.1733.
Optical: $[a]^{20}_D$ = +10.0° (*c* = 2.03, $CHCl_3$, 87% e.e.)
HPLC (Chiralpak AD-H, ^{i}PrOH/*n*-hexane = 20/80, flow rate = 1.0 mL/min, λ = 254 nm) t_R = 47.1 min (major), 36.8 min (minor)

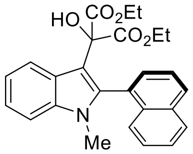

Diethyl 2-Hydroxy-2-(1-methyl-2-(naphthalen-1-yl)-1*H*-indol-3-yl)malonate (5ga) was synthesized by following Procedure 22 from **4g**. The crude material was purified by normal-phase column chromatography using an eluent of 10% EtOAc/CH_2Cl_2 to give **5ga** (40% yield).

^{1}H NMR (400 MHz, $CDCl_3$) δ 7.98–7.90 (m, 2H), 7.68 (dt, *J* = 8.1, 1.0 Hz, 1H), 7.61–7.49 (m, 4H), 7.45 (ddd, *J* = 8.3, 6.9, 1.4 Hz, 1H), 7.38 (dt, *J* = 8.2, 1.0 Hz, 1H), 7.31 (ddd, *J* = 8.2, 7.0, 1.2 Hz, 1H), 7.19 (ddd, *J* = 8.1, 7.0, 1.2 Hz, 1H), 4.13 (brs, 1H), 3.80 (ddq, *J* = 12.4, 10.7, 7.1 Hz, 2H), 3.70 (dq, *J* = 10.7, 7.1 Hz, 1H), 3.27 (s, 3H), 2.95 (dq, *J* = 10.7, 7.1 Hz, 1H), 0.96 (t, *J* = 7.1 Hz, 3H), 0.75 (t, *J* = 7.2 Hz, 3H).
^{13}C NMR (100 MHz, $CDCl_3$) δ 170.4, 170.3, 137.1, 137.0, 133.4, 133.3, 130.8, 129.7, 128.7, 128.3, 127.0, 126.6, 126.5, 126.3, 125.2, 122.4, 120.6, 120.3, 110.8, 109.5, 78.3, 62.41, 62.39, 30.5, 13.7, 13.3.
IR (FT-ATR, cm^{-1}, $CHCl_3$) ν_{max} 3946, 3926, 3899, 3865, 3842, 3803, 3780, 3730, 3699, 3626, 3595, 3568, 3483, 3302, 3244, 3051, 2981, 2935, 2407, 2360, 2333, 2299, 2268, 1963, 1944, 1894, 1845, 1728, 1601, 1550, 1504, 1466, 1369, 1261, 1215, 1161, 1134, 1095, 1065, 1034, 976, 722, 864, 744, 671.
HRMS (EI) *m*/*z*: [M]$^+$ Calcd for $C_{26}H_{25}NO_5$ 431.1733; found 431.1734.
Optical: $[a]^{20}_D$ = +38.2° (*c* = 0.89, $CHCl_3$, 77% e.e.)

HPLC (Chiralpak AD-H, ^{i}PrOH/n-hexane = 20/80, flow rate = 1.0 mL/min, λ = 254 nm) t_R = 17.7 min (major), 23.4 min (minor)

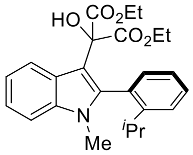

Diethyl 2-Hydroxy-2-(2-(2-isopropylphenyl)-1-methyl-1*H*-indol-3-yl)malonate (5ha) was synthesized by following Procedure 22 from **4h**. The crude material was purified by normal-phase column chromatography using an eluent of 10% EtOAc/CH_2Cl_2 to give **5ha** (59% yield).

^{1}H NMR (400 MHz, $CDCl_3$) δ 7.62 (d, J = 8.0 Hz, 1H), 7.47–7.39 (m, 2H), 7.33 (d, J = 8.1 Hz, 1H), 7.29–7.18 (m, 3H), 7.16 (ddd, J = 8.1, 6.9, 1.2 Hz, 1H), 4.06 (dqd, J = 10.6, 7.1, 4.9 Hz, 2H), 3.87–3.73 (m, 2H), 3.34 (s, 3H), 2.72 (hept, J = 6.8 Hz, 1H), 1.20 (d, J = 6.8 Hz, 3H), 1.15 (td, J = 7.1, 2.4 Hz, 7H), 1.09 (d, J = 6.9 Hz, 3H).
^{13}C NMR (100 MHz, $CDCl_3$) δ 170.5, 170.3, 150.1, 137.7, 136.8, 131.6, 129.9, 129.7, 126.4, 125.7, 125.1, 122.1, 120.8, 120.1, 109.54, 109.48, 78.3, 62.6, 62.4, 30.41, 30.38, 24.3, 23.5, 14.0, 13.9.
IR (FT-ATR, cm^{-1}, $CHCl_3$) ν_{max} 3980, 3930, 3907, 3865, 3842, 3826, 3807, 3780, 3757, 3737, 3683, 3656, 3491, 3302, 3167, 3055, 2958, 2927, 2866, 2723, 2603, 2538, 2372, 2310, 1071, 1925, 1882, 1809, 1728, 1608, 1577, 1554, 1466, 1365, 1230, 1161, 1130, 1095, 1065, 1034, 922, 860, 802, 744, 710, 660.
HRMS (EI) m/z: $[M]^+$ Calcd for $C_{25}H_{29}NO_5$ 423.2046; found 423.2042.
Optical: $[a]^{20}_{D}$ = +1.9° (c = 1.12, $CHCl_3$, 43% e.e.)
HPLC (Chiralpak AD-H, ^{i}PrOH/n-hexane = 10/90, flow rate = 1.0 mL/min, λ = 254 nm) t_R = 70.4 min (major), 31.1 min (minor)

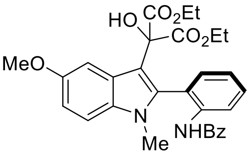

Diethyl 2-(2-(2-Benzamidophenyl)-5-methoxy-1-methyl-1*H*-indol-3-yl)-2-hydroxymalonate (5ja) was synthesized by following Procedure 22 from **1j**. The crude material was purified by normal-phase column chromatography using an eluent of 10% EtOAc/CH_2Cl_2 to give **5ja** (99% yield).

^{1}H NMR (400 MHz, $CDCl_3$) δ 8.33 (dd, J = 8.3, 1.1 Hz, 1H), 8.29 (s, 1H), 7.63–7.57 (m, 2H), 7.54 (ddd, J = 8.4, 7.3, 1.8 Hz, 1H), 7.45–7.37 (m, 1H), 7.35–7.23 (m, 4H), 7.21 (d, J = 8.9 Hz, 1H), 7.09 (d, J = 2.4 Hz, 1H), 6.94 (dd, J = 8.9, 2.4 Hz, 1H), 4.16 (s, 1H), 4.03 (ddq, J = 12.5, 10.7, 7.1 Hz, 2H), 3.90–3.77 (m, 4H), 3.62 (dq, J = 10.8, 7.1 Hz, 1H), 3.29 (s, 3H), 1.11 (dt, J = 9.9, 7.1 Hz, 6H).
^{13}C NMR (100 MHz, $CDCl_3$) δ 170.4, 170.1, 165.7, 154.7, 138.4, 134.38, 134.35, 132.5, 132.2, 131.9, 130.7, 128.7 (2C), 127.4 (2C), 126.3, 124.3, 122.9, 113.3, 110.7, 110.6, 102.3, 78.3, 63.02, 62.97, 56.1, 30.3, 13.93, 13.87.
IR (FT-ATR, cm^{-1}, $CHCl_3$) ν_{max} 3973, 3930, 3907, 3880, 3865, 3841, 3822, 3807, 3780, 3757, 3718, 3680, 3656, 3629, 3591, 3464, 3394, 3062, 2985, 2939, 2835, 2603, 2438,

2372, 2314, 2067, 1967, 1925, 1817 1732, 1674, 1616, 1577, 1516, 1485, 1450, 1389, 1373, 1300, 1254, 1219, 1203, 1169, 1130, 1095, 1061, 1030, 949, 891, 856, 798, 748, 710, 660.
HRMS (EI) *m/z*: $[M]^+$ Calcd for $C_{30}H_{30}N_2O_7$ 530.2053; found 530.2052.
Optical: $[a]^{20}_D = -56.1°$ (c = 2.43, $CHCl_3$, 94% e.e.)
HPLC (Chiralpak AD-H, ^{i}PrOH/*n*-hexane = 20/80, flow rate = 1.0 mL/min, λ = 254 nm) t_R = 36.6 min (major), 76.7 min (minor)

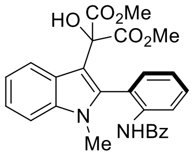

Dimethyl 2-(2-(2-Benzamidophenyl)-1-methyl-1*H*-indol-3-yl)-2-hydroxymalonate (5ab) was synthesized by following Procedure 22 from **4a**. The crude material was purified by normal-phase column chromatography using an eluent of 10% EtOAc/CH_2Cl_2 to give **5ab** (91% yield).

^{1}H NMR (400 MHz, $CDCl_3$) δ 8.34 (dd, J = 8.1, 1.1 Hz, 1H), 8.29 (s, 1H), 7.63–7.52 (m, 4H), 7.44–7.37 (m, 1H), 7.36–7.27 (m, 6H), 7.18 (ddd, J = 8.1, 6.8, 1.4 Hz, 1H), 3.49 (s, 3H), 3.47 (s, 3H), 3.34 (s, 3H).
^{13}C NMR (100 MHz, $CDCl_3$) δ 170.9, 170.5, 165.7, 138.4, 137.1, 134.4, 134.2, 132.1, 131.9, 130.7, 128.7 (2C), 127.4 (2C), 125.9, 124.4, 123.2, 123.0, 122.7, 120.8, 120.1, 111.0, 110.0, 78.2, 53.7, 53.6, 30.3.
IR (FT-ATR, cm^{-1}, $CHCl_3$) ν_{max} 3973, 3930, 3907, 3880, 3865, 3842, 3822, 3808, 3780, 3753, 3741, 3718, 3680, 3653, 3618, 3595, 3471, 3394, 3055, 3016, 2951, 2924, 2854, 2607, 2376, 2303, 2171, 2071, 1925, 1813, 1739, 1670, 1604, 1581, 1516, 1446, 1365, 1300, 1250, 1223, 1161, 1130, 1099, 1065, 1038, 968, 937, 903, 852, 798, 748, 706, 656.
HRMS (EI) *m/z*: $[M]^+$ Calcd for $C_{27}H_{24}N_2O_6$ 472.1634; found 472.1633.
Optical: $[a]^{20}_D = -7.1°$ (c = 2.11, $CHCl_3$, 14% e.e.)
HPLC (Chiralpak IC, ^{i}PrOH/*n*-hexane = 40/60, flow rate = 1.0 mL/min, λ = 254 nm) t_R = 12.6 min (major), 20.0 min (minor)

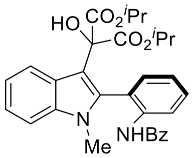

Diisopropyl 2-(2-(2-Benzamidophenyl)-1-methyl-1*H*-indol-3-yl)-2-hydroxymalonate(5ac) was synthesized by following Procedure 22 from **4a**. The crude material was purified by normal-phase column chromatography using an eluent of 10% EtOAc/CH_2Cl_2 to give **5ac** (91% yield).

^{1}H NMR (400 MHz, $CDCl_3$) δ 8.40 (s, 1H), 8.26 (d, J = 8.0 Hz, 1H), 7.70–7.59 (m, 3H), 7.54 (td, J = 7.8, 1.8 Hz, 1H), 7.46–7.36 (m, 1H), 7.36–7.21 (m, 6H), 7.15 (ddd, J = 8.2, 6.8, 1.3 Hz, 1H), 4.80 (ddt, J = 12.5, 9.3, 6.3 Hz, 2H), 4.15 (brs, 1H), 3.32 (s, 3H), 1.17 (dd, J = 6.2, 3.7 Hz, 6H), 1.12 (d, J = 6.3 Hz, 3H), 1.06 (d, J = 6.3 Hz, 3H).

^{13}C NMR (100 MHz, $CDCl_3$) δ 170.0, 169.7, 165.7, 138.2, 137.2, 134.6, 134.3, 132.2, 131.8, 130.5, 128.7 (2C), 127.4 (2C), 125.9, 124.3, 123.9, 123.4, 122.6, 120.8, 120.2, 111.0, 109.8, 78.5, 71.3, 71.2, 30.2, 21.53 (2C), 21.48, 21.46.
IR (FT-ATR, cm^{-1}, $CHCl_3$) ν_{max} 3626, 3899, 3865, 3838, 3799, 3776, 3730, 3670, 3626, 3595, 3464, 3374, 3163, 3059, 2981, 2935, 2360, 2337, 2295, 2268, 1921, 1867, 1813, 1581, 1520, 1493, 1454, 1373, 1254, 1184, 1138, 1099, 1068, 1034, 933, 906, 829, 798, 748, 710, 663.
HRMS (EI) *m*/*z*: $[M]^+$ Calcd for $C_{31}H_{32}N_2O_6$ 528.2260; found 528.2258.
Optical: $[a]^{20}_D$ = –27.8° (*c* = 2.73, $CHCl_3$, 96% e.e.)
HPLC (Chiralpak AD, ^{i}PrOH/*n*-hexane = 20/80, flow rate = 1.0 mL/min, λ = 254 nm) t_R = 15.0 min (major), 11.1 min (minor)

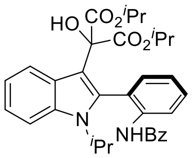

Diisopropyl 2-(2-(2-Benzamidophenyl)-1-isopropyl-1*H*-indol-3-yl)-2-hydroxymalonate (5kc) was synthesized by following Procedure 22 from **4k**. The crude material was purified by normal-phase column chromatography using an eluent of 10% EtOAc/CH_2Cl_2 to give **5kc** (99% yield).

^{1}H NMR (400 MHz, $CDCl_3$) δ 8.51 (s, 1H), 8.18 (dd, *J* = 8.3, 1.1 Hz, 1H), 7.70–7.61 (m, 3H), 7.57–7.50 (m, 2H), 7.45–7.37 (m, 1H), 7.35–7.21 (m, 4H), 7.19 (ddd, *J* = 8.4, 7.0, 1.3 Hz, 1H), 7.11 (ddd, *J* = 8.1, 7.0, 1.1 Hz, 1H), 4.82 (hept, *J* = 6.3 Hz, 1H), 4.71 (hept, *J* = 6.3 Hz, 1H), 4.11–3.98 (m, 1H), 1.41 (d, *J* = 7.0 Hz, 3H), 1.34 (d, *J* = 7.0 Hz, 3H), 1.17 (dd, *J* = 6.2, 4.3 Hz, 6H), 1.10 (dd, *J* = 10.0, 6.3 Hz, 6H).
^{13}C NMR (100 MHz, $CDCl_3$) δ 170.1, 169.6, 165.7, 138.3, 134.8, 134.3, 134.1, 132.2, 131.8, 130.4, 128.7 (2C), 127.4 (2C), 127.0, 124.6, 124.4, 123.7, 121.9, 121.1, 119.7, 112.4, 110.7, 78.7, 71.3, 71.2, 48.0, 21.52 (2C), 21.49, 21.47, 21.45, 21.35.
IR (FT-ATR, cm^{-1}, $CHCl_3$) ν_{max} 3953, 3930, 3907, 3880, 3842, 3822, 3807, 3780, 3757, 3718, 3680, 3656, 3629, 3614, 3471, 3398, 3055, 2981, 2935, 2881, 2742, 2619, 2441, 2372, 2345, 2314, 2202, 2067, 1921, 1809, 1724, 1674, 1608, 1581, 1516, 1446, 1412, 1373, 1335, 1273, 1250, 1227, 1184, 1142, 1099, 1022, 933, 906, 829, 748, 717, 710, 663.
HRMS (EI) *m*/*z*: $[M]^+$ Calcd for $C_{33}H_{36}N_2O_6$ 556.2573; found 556.2568.
Optical: $[a]^{20}_D$ = –20.4° (*c* = 2.59, $CHCl_3$, 45% e.e.)
HPLC (Chiralpak IC, ^{i}PrOH/*n*-hexane = 20/80, flow rate = 1.0 mL/min, λ = 254 nm) t_R = 10.3 min (major), 15.1 min (minor)

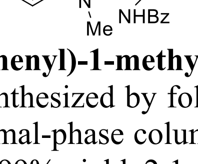

Ethyl 2-(2-(2-Benzamidophenyl)-1-methyl-1*H*-indol-3-yl)-3,3,3-trifluoro-2-hydroxypropanoate (5ad) was synthesized by following Procedure 22 from **4a**. The crude material was purified by normal-phase column chromatography using an eluent of 5% EtOAc/CH_2Cl_2 to give **5ad** (99% yield, 2:1 d.r.).

^{1}H NMR (400 MHz, $CDCl_3$) Major diastereomer: δ 8.34 (dd, J = 8.2, 1.2 Hz, 1H), 7.96 (dd, J = 8.3, 1.3 Hz, 1H), 7.70 (s, 1H), 7.52–7.45 (m, 2H), 7.44–7.39 (m, 1H), 7.37–7.30 (m, 1H), 7.28–7.08 (m, 7H), 4.03 (s, 1H), 4.00–3.87 (m, 1H), 3.33–3.23 (m, 1H), 3.20 (s, 3H), 0.99 (t, J = 7.1 Hz, 3H). Minor diastereomer: δ 8.23 (dd, J = 8.3, 1.1 Hz, 1H), 8.00 (dt, J = 8.0, 1.1 Hz, 1H), 7.71 (s, 1H), 7.52–7.44 (m, 1H), 7.44–7.38 (m, 2H), 7.37–7.30 (m, 1H), 7.28–7.07 (m, 7H), 3.94 (dtd, J = 15.5, 7.2, 3.6 Hz, 1H), 3.86 (s, 1H), 3.43 (dq, J = 10.8, 7.2 Hz, 1H), 3.22 (s, 3H), 1.03 (t, J = 7.1 Hz, 3H).
^{13}C NMR (100 MHz, $CDCl_3$) Major diastereomer: δ 168.8, 165.6, 137.8, 137.2, 134.4, 134.0, 132.2, 131.7, 130.8, 128.9 (2C), 127.0 (2C), 125.8, 124.6, 124.1 (q, J = 284.0 Hz, 1C), 123.2, 123.1, 122.4, 122.3 (q, J = 3.0 Hz, 1C), 120.9, 109.8, 107.5, 77.6 (q, J = 11.0 Hz, 1C), 63.6, 30.2, 13.6. Minor diastereomer: δ 169.8, 165.6, 137.6, 137.2, 135.2, 134.2, 132.1, 132.1, 130.8, 128.9 (2C), 127.1 (2C), 125.7, 124.7, 124.1 (q, J = 284.0 Hz, 1C), 123.1, 123.03, 122.95, 122.3 (q, J = 3.0 Hz, 1C), 120.9, 109.9, 107.9, 77.6 (q, J = 11.0 Hz, 1C), 63.8, 30.3, 13.7.
IR (FT-ATR, cm^{-1}, $CHCl_3$) ν_{max} 3950, 3933, 3925, 3905, 3853, 3840, 3822, 2801, 3752, 3737, 3727, 3692, 3677, 3650, 3630, 3622, 3600, 3568, 3553, 3400, 3058, 3018, 2997, 2980, 2937, 2401, 2385, 2369, 2359, 2348, 2338, 2328, 2295, 1898, 1736, 1663, 1611, 1580, 1522, 1493, 1466, 1449, 1430, 1368, 1303, 1270, 1235, 1218, 1182, 1135, 1096, 1065, 1043, 1026, 982, 911, 857, 822, 794, 776, 742, 709, 667.
HRMS (EI) m/z: $[M]^+$ Calcd for $C_{27}H_{23}F_3N_2O_4$ 496.1610; found 496.1609.
Optical: $[a]^{20}_D$ = +1.61° (c = 2.82, $CHCl_3$, 2:1 d.r., 34% e.e.)
HPLC (Chiralpak OD-H, ^{i}PrOH/n-hexane = 10/90, flow rate = 1.0 mL/min, λ = 254 nm) t_R = 13.7 min (major diastereomer), 9.7 min (minor enantiomer)

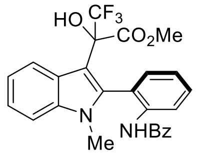

Methyl 2-(2-(2-Benzamidophenyl)-1-methyl-1*H*-indol-3-yl)-3,3,3-trifluoro-2-hydroxypropanoate (5ae) was synthesized by following Procedure 22 from **4a**. The crude material was purified by normal-phase column chromatography using an eluent of 5% EtOAc/CH_2Cl_2 to give **5ae** (91% yield, 4:1 d.r.).

^{1}H NMR (400 MHz, $CDCl_3$) Major diastereomer: δ 8.43 (d, J = 8.4 Hz, 1H), 8.03 (dq, J = 8.2, 1.2 Hz, 1H), 7.81 (s, 1H), 7.63–7.48 (m, 3H), 7.48–7.40 (m, 1H), 7.39–7.22 (m, 6H), 7.20 (dd, J = 7.6, 1.5 Hz, 1H), 4.14 (s, 1H), 3.31 (s, 3H), 3.30 (s, 3H). Minor diastereomer: δ 8.35 (d, J = 8.3 Hz, 1H), 8.10 (dd, J = 8.2, 1.2 Hz, 1H), 7.76 (s, 1H), 7.63–7.48 (m, 4H), 7.48–7.40 (m, 1H), 7.39–7.22 (m, 6H), 3.98 (s, 1H), 3.37 (s, 3H), 3.33 (d, J = 0.9 Hz, 3H).
^{13}C NMR (100 MHz, $CDCl_3$) Major diastereomer: δ 169.1, 165.7, 137.8, 137.2, 134.4, 134.0, 132.2, 131.7, 130.9, 128.9 (2C), 127.0 (2C), 125.8, 124.7, 124.1 (q, J = 281.0 Hz, 1C), 123.3, 122.9, 122.6, 122.1(q, J = 11.0 Hz, 1C), 121.0, 109.9, 107.3, 53.6, 30.2. Minor diastereomer: δ 170.2, 165.6, 137.6, 137.1, 135.0, 134.2, 132.1, 131.7, 131.0, 128.9 (2C), 127.1 (2C), 125.7, 124.7, 124.1 (q, J = 281.0 Hz, 1C), 123.1, 123.0, 122.6, 122.1 (q, J = 11.0 Hz, 1C), 121.0, 110.0, 107.8, 53.8, 30.3.
IR (FT-ATR, cm^{-1}, $CHCl_3$) ν_{max} 3950, 3933, 3925, 3905, 3882, 3874, 3855, 3840, 3822, 3801, 3752, 3737, 3727, 3692, 3677, 3651, 3630, 3622, 3600, 3568, 3553, 3399, 3058, 2953, 2930, 2852, 2385, 2369, 2359, 2348, 2338, 2328, 2295, 1750, 1655, 1610, 1580,

1513, 1493, 1450, 1363, 1302, 1248, 1180, 1135, 1100, 1065, 1044, 1023, 988, 953, 904, 872, 856, 805, 793, 744, 710, 668.

HRMS (EI) *m*/*z*: $[M]^+$ Calcd for $C_{26}H_{21}F_3N_2O_4$ 482.1453; found 482.1454.

Optical: $[a]^{20}_D$ = +4.3° (*c* = 2.19, $CHCl_3$, 4:1 d.r., 37% e.e.)

HPLC (Chiralpak OD-H, ^{i}PrOH/*n*-hexane = 10/90, flow rate = 1.0 mL/min, λ = 254 nm) t_R = 17.4 min (major diastereomer), 11.1 min (minor enantiomer)

8.1 NMR Spectra of 5

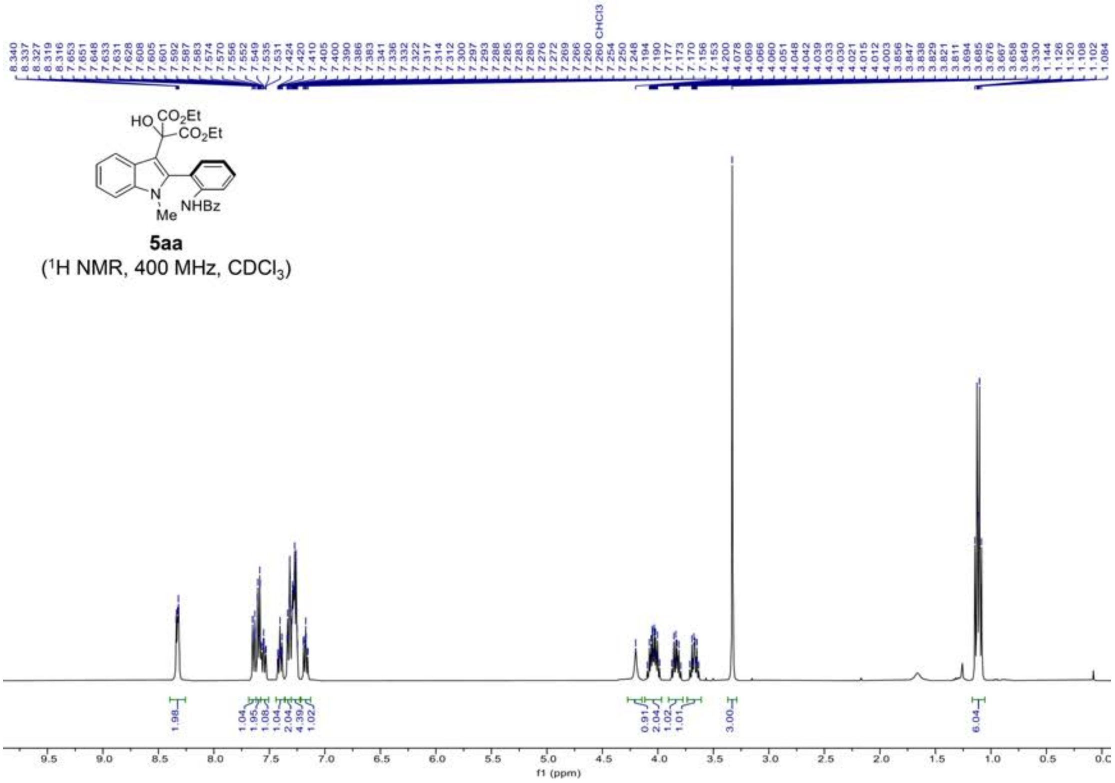

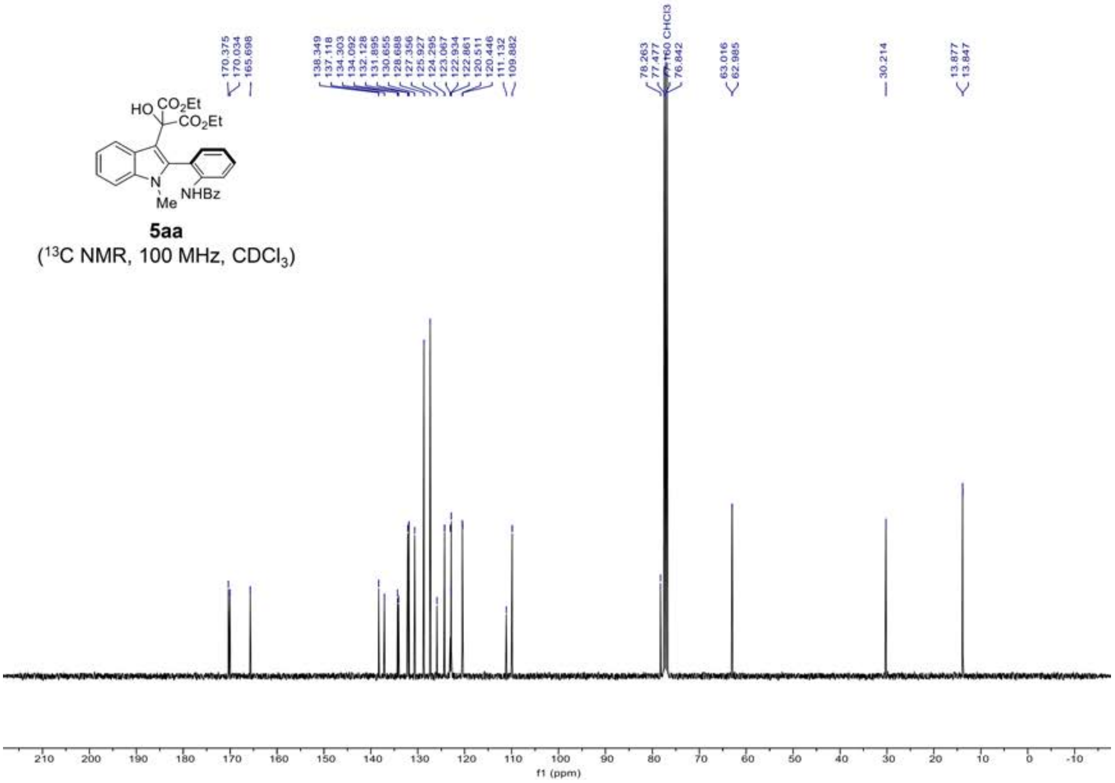

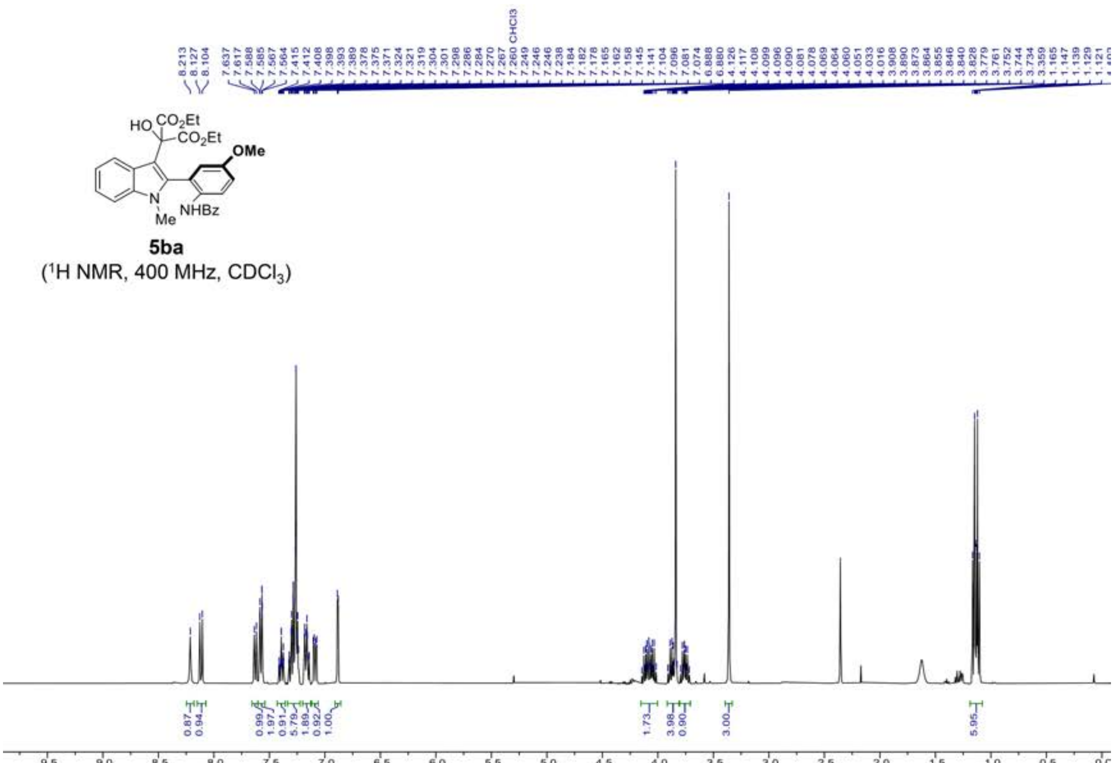

5ba
(^{1}H NMR, 400 MHz, $CDCl_3$)

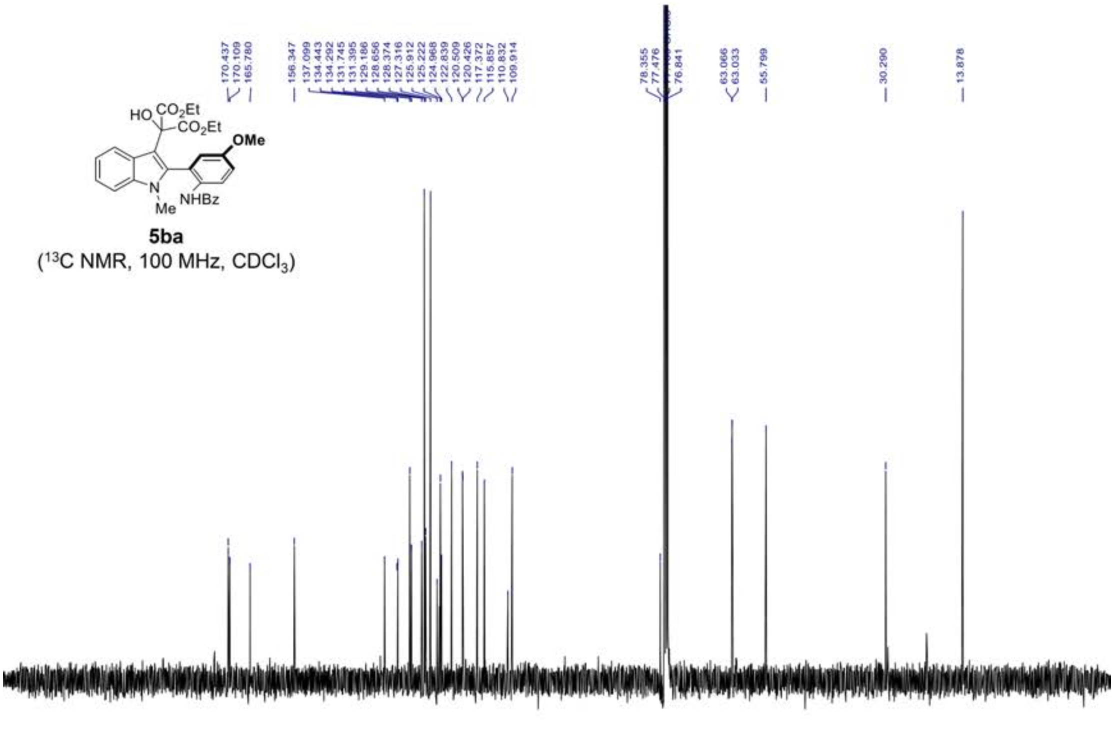

5ba
(^{13}C NMR, 100 MHz, $CDCl_3$)

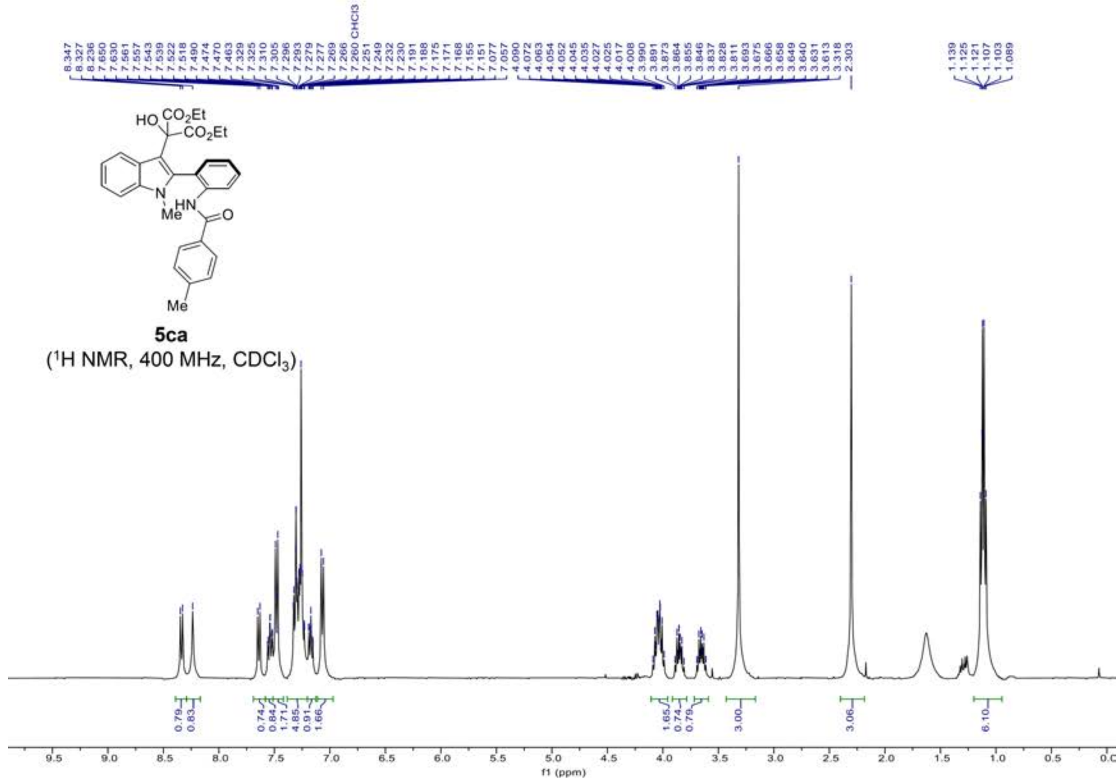

5ca
(^{1}H NMR, 400 MHz, $CDCl_3$)

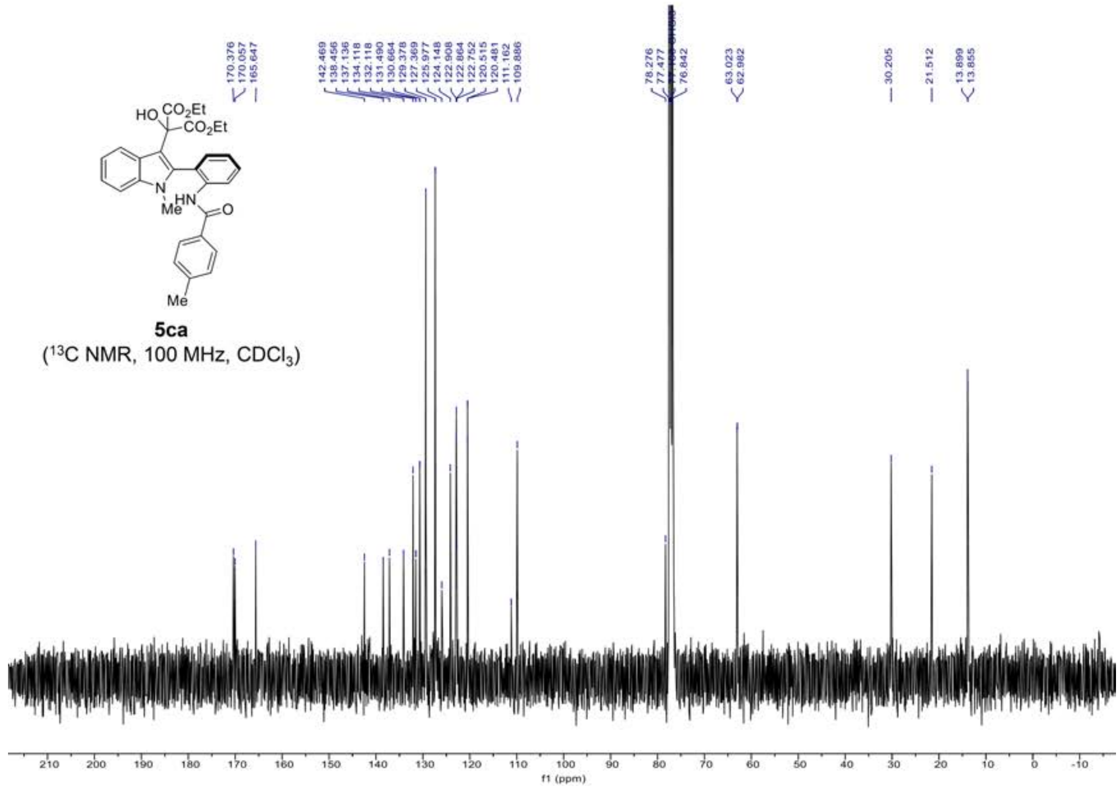

5ca
(^{13}C NMR, 100 MHz, $CDCl_3$)

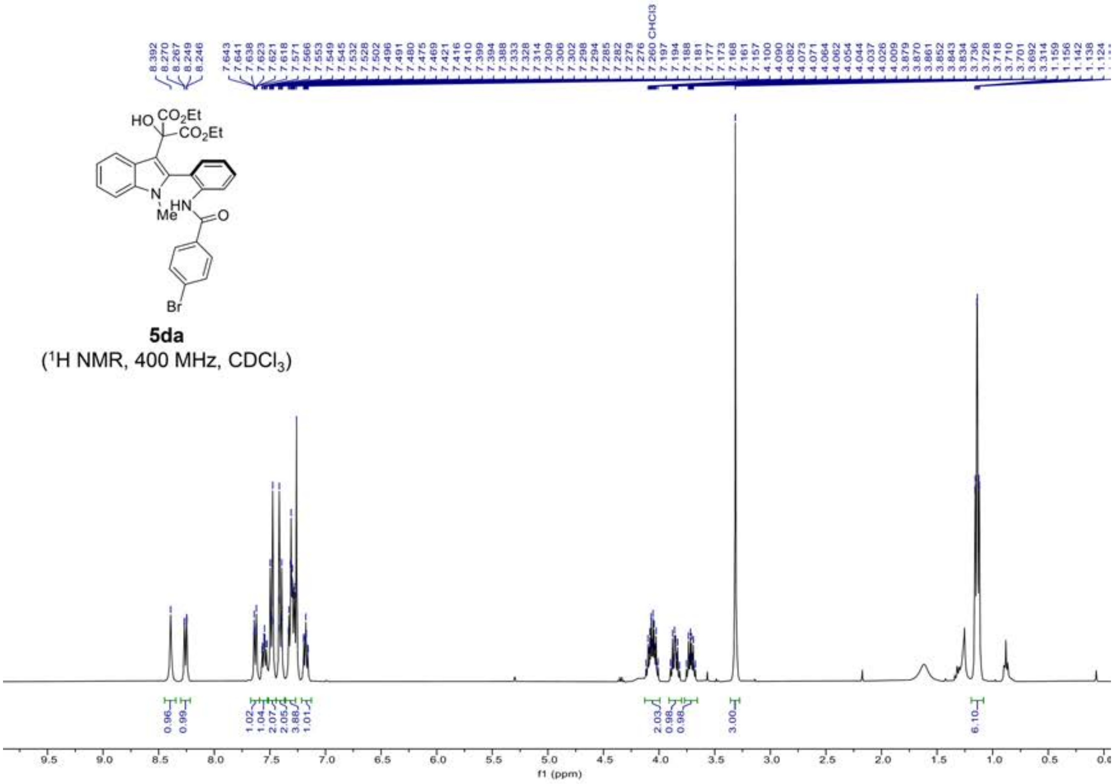

5da
(^{1}H NMR, 400 MHz, $CDCl_3$)

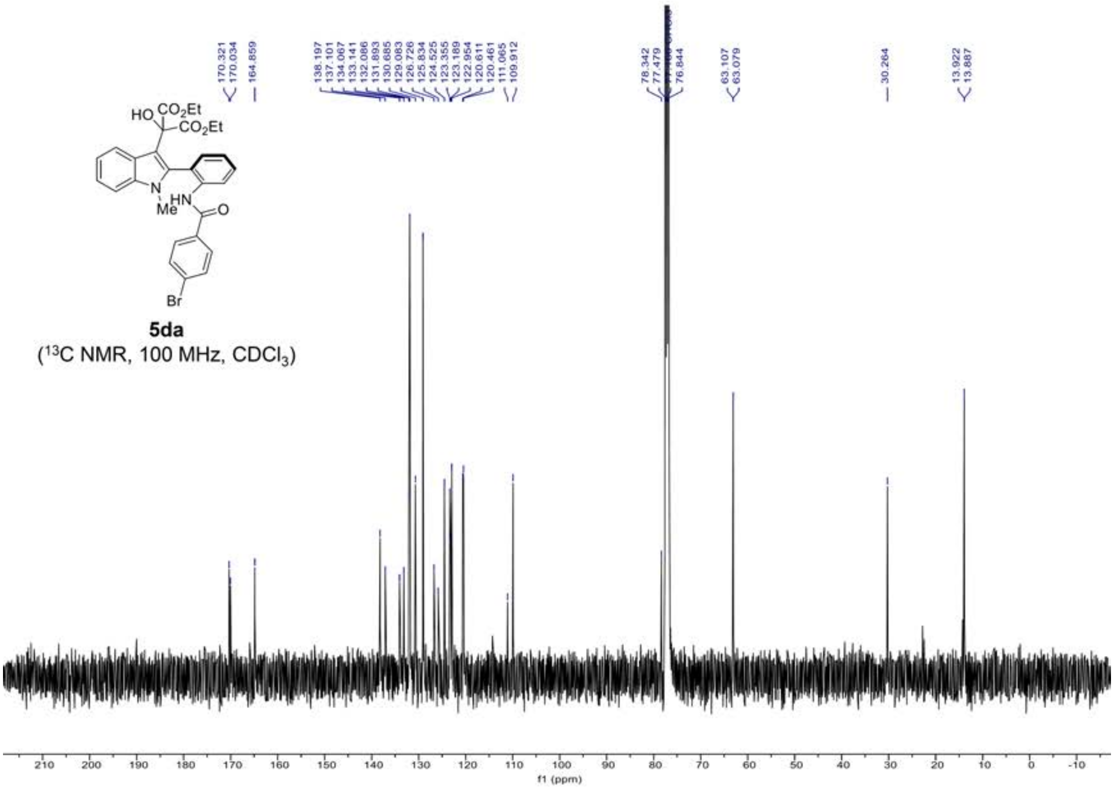

5da
(^{13}C NMR, 100 MHz, $CDCl_3$)

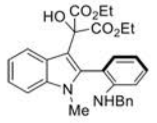

5ea

(^{1}H NMR, 400 MHz, $CDCl_3$)

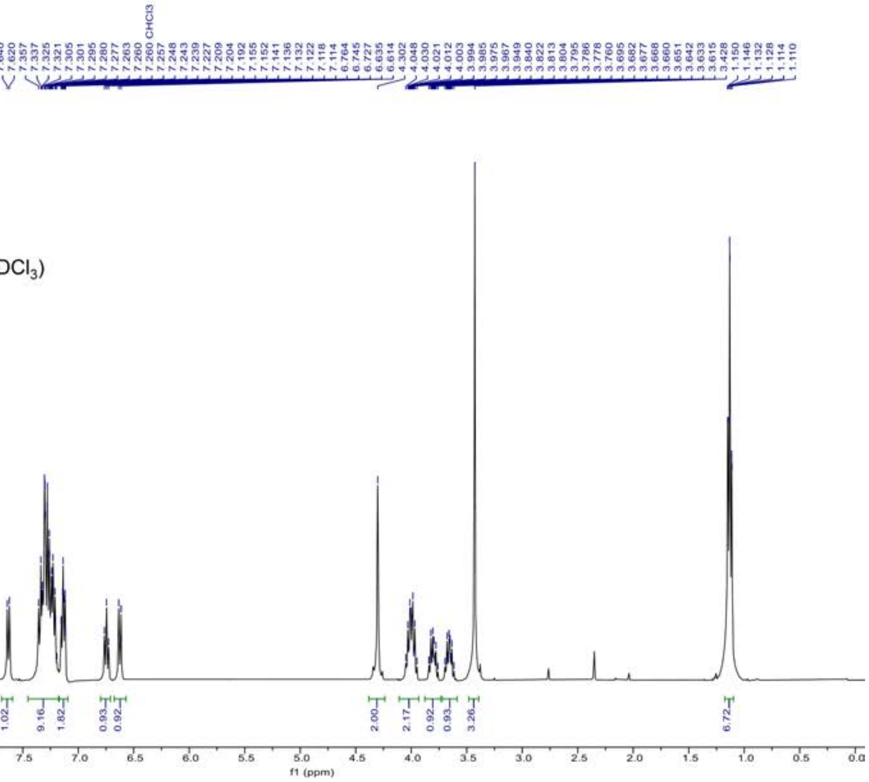

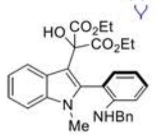

5ea

(^{13}C NMR, 100 MHz, $CDCl_3$)

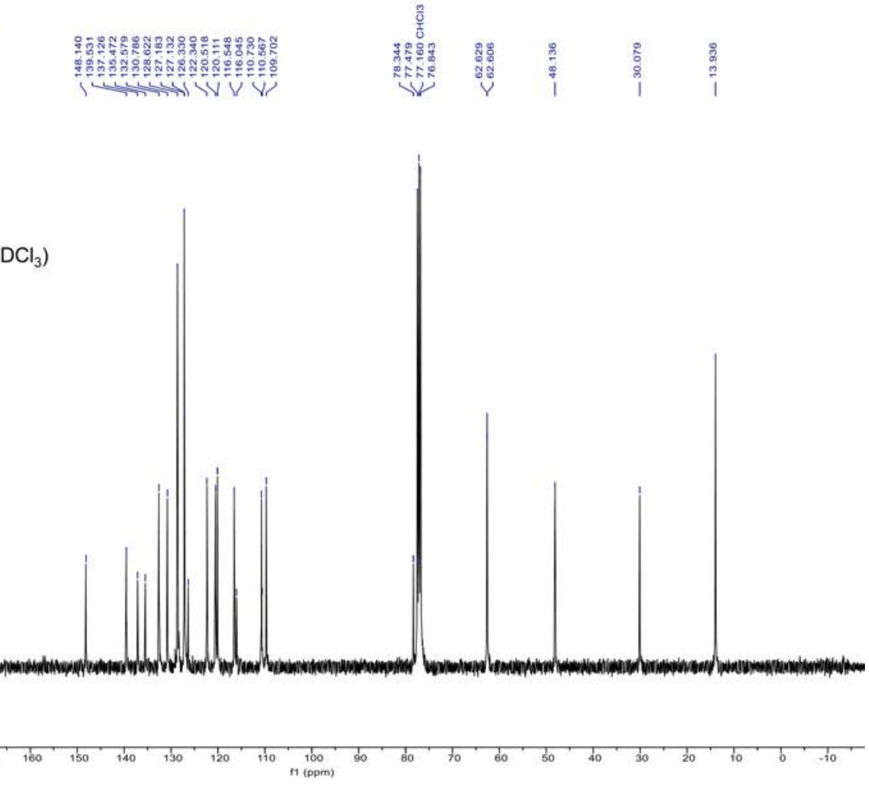

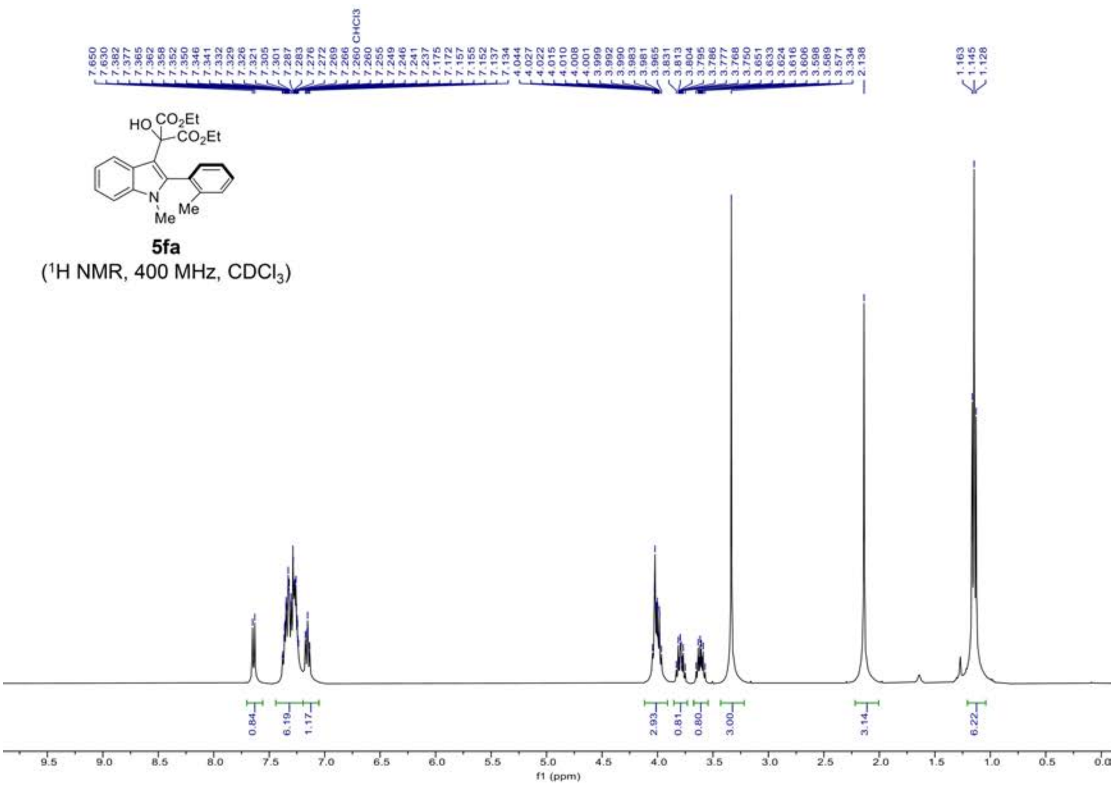

5fa
(^{1}H NMR, 400 MHz, $CDCl_3$)

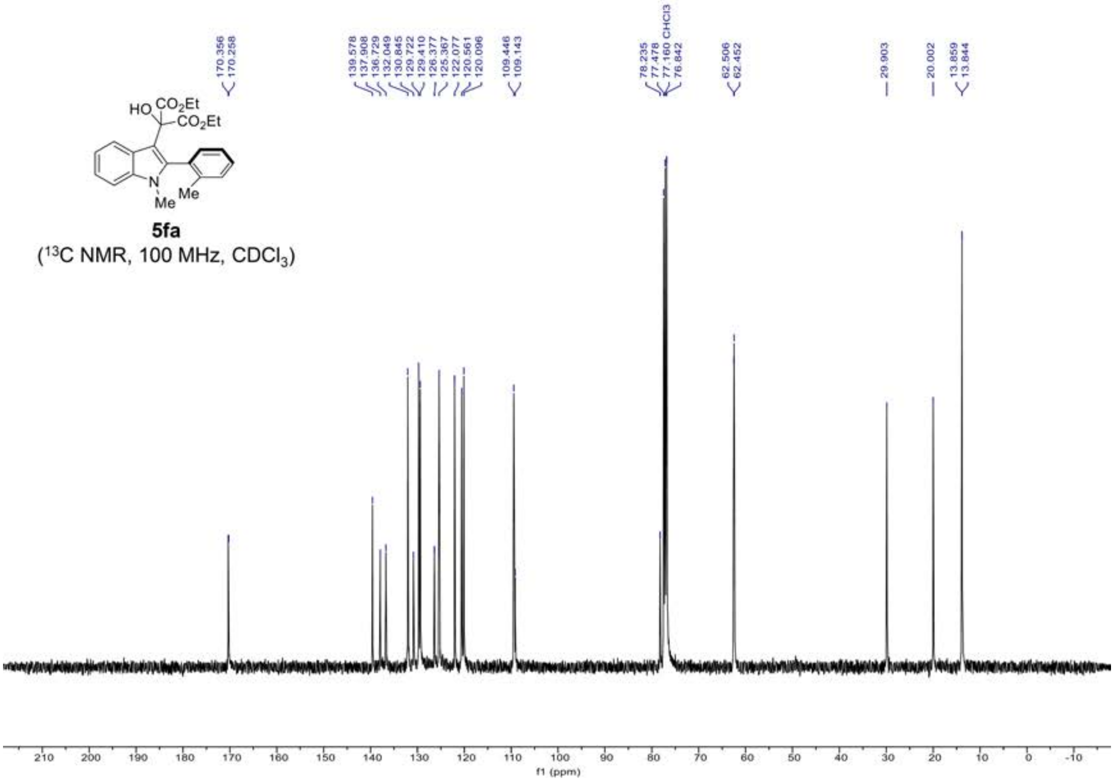

5fa
(^{13}C NMR, 100 MHz, $CDCl_3$)

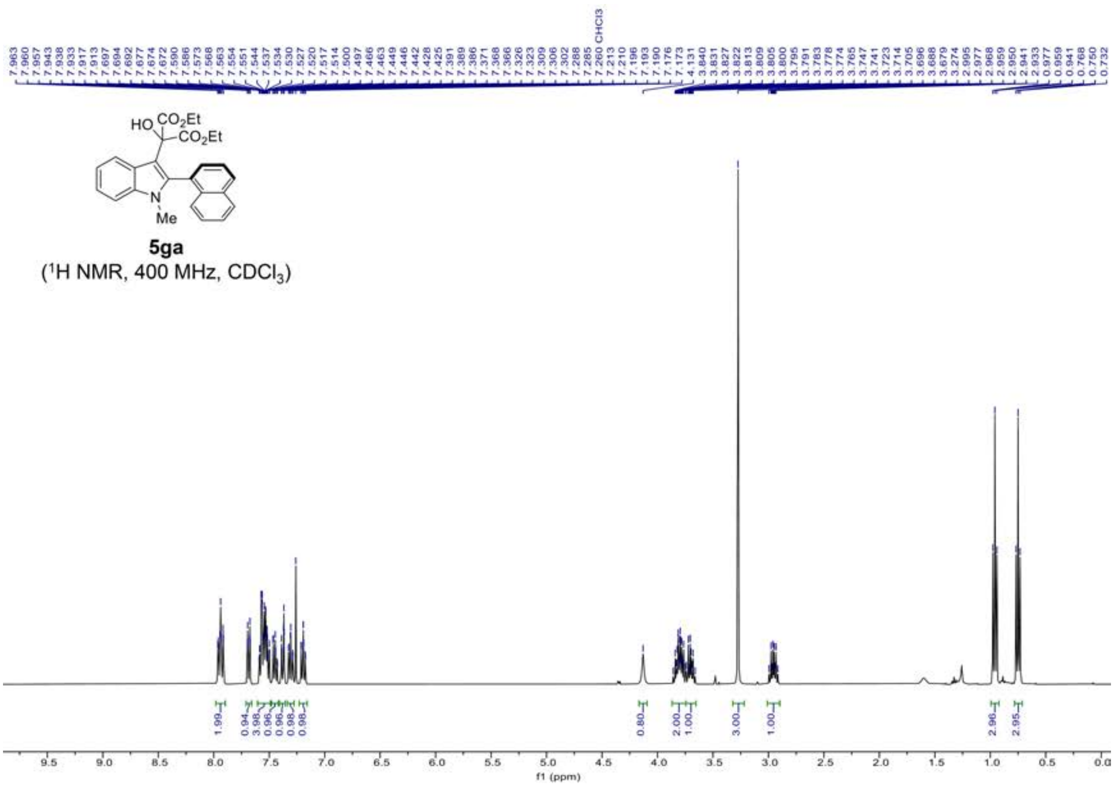

5ga
(^{1}H NMR, 400 MHz, $CDCl_3$)

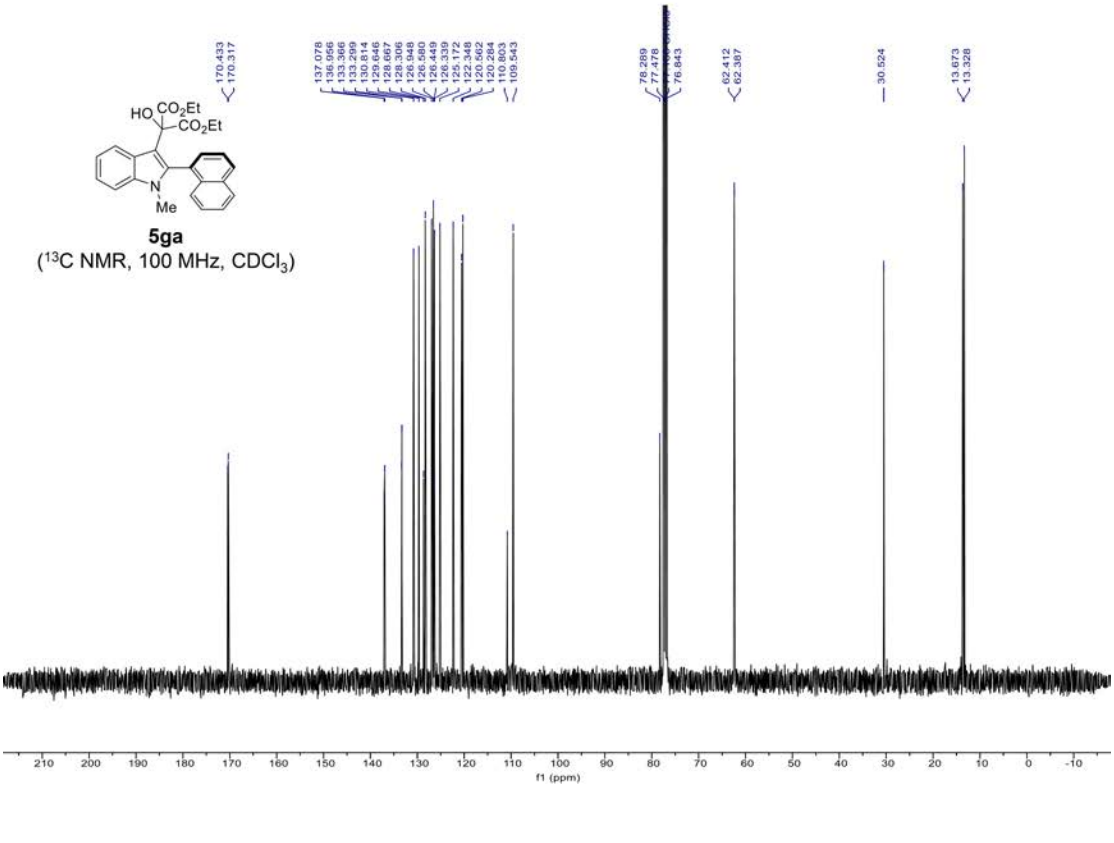

5ga
(^{13}C NMR, 100 MHz, $CDCl_3$)

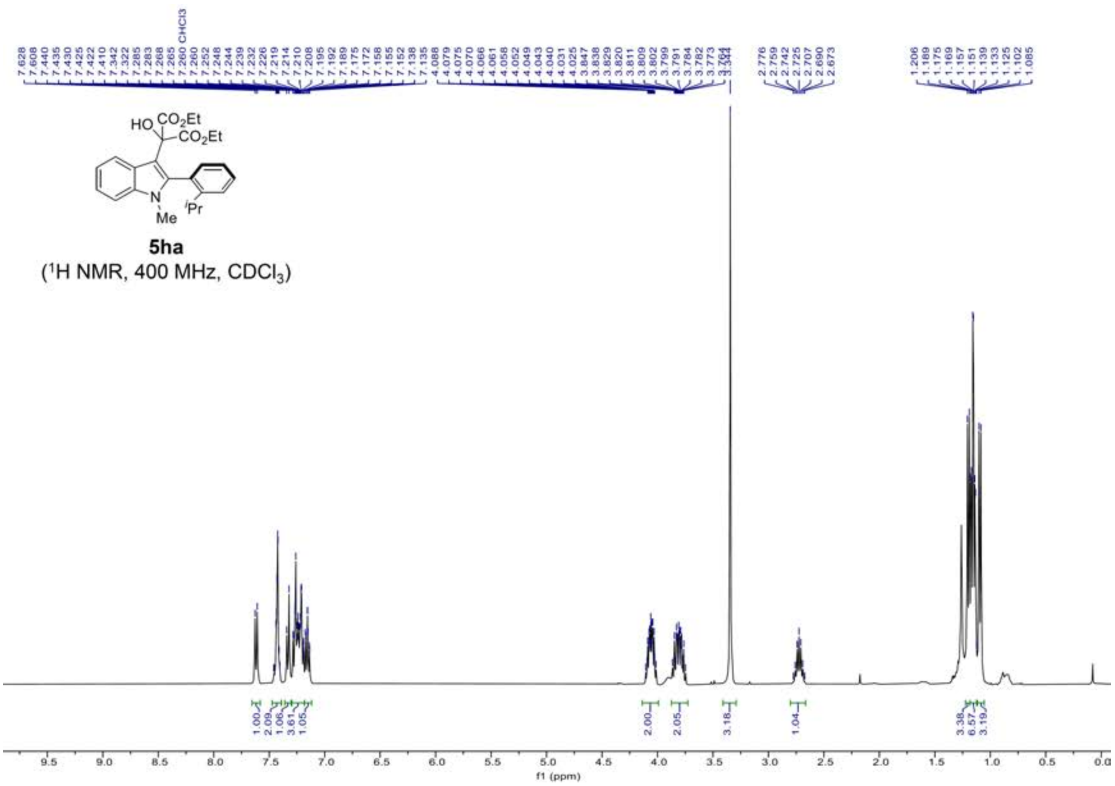

5ha
(^{1}H NMR, 400 MHz, $CDCl_3$)

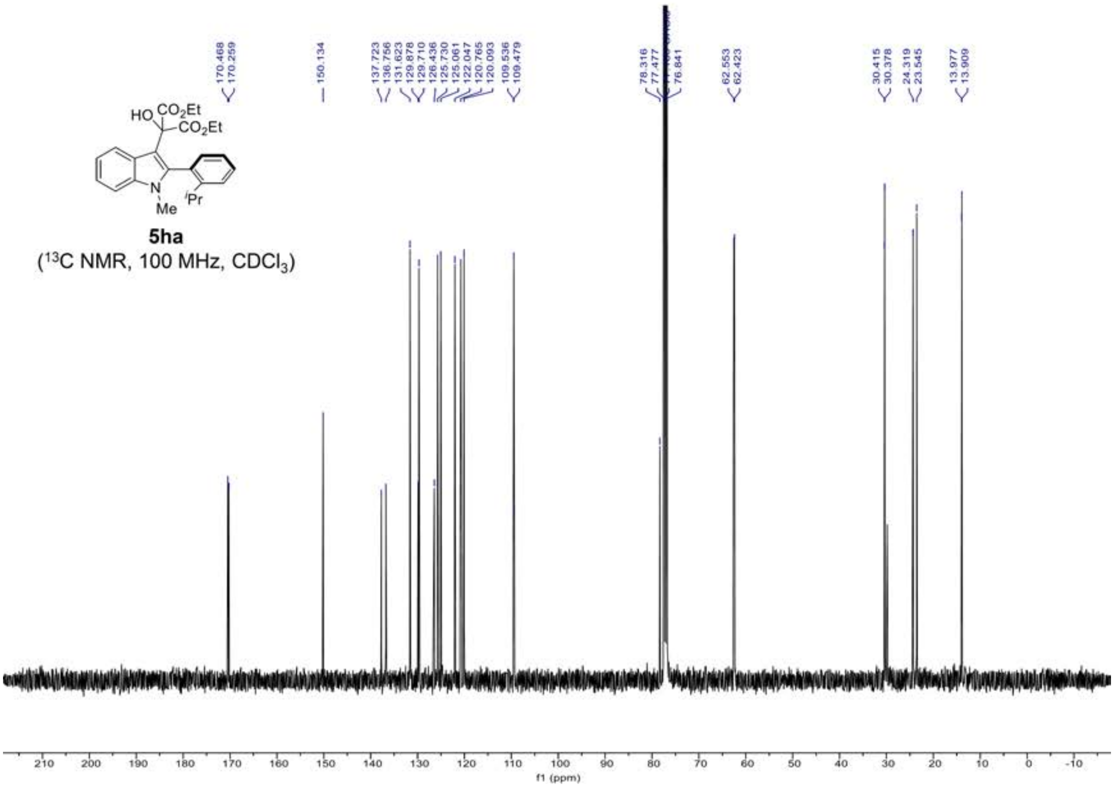

5ha
(^{13}C NMR, 100 MHz, $CDCl_3$)

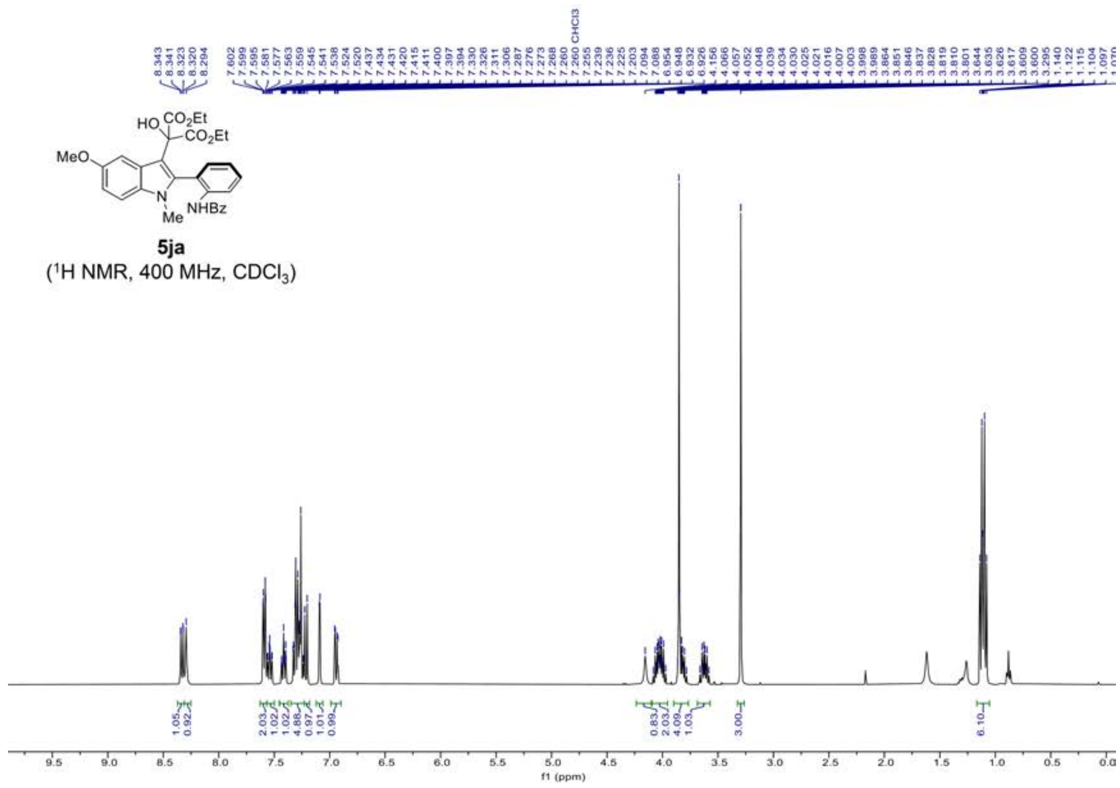
HO CO2Et
CO2Et
MeO
N
Me
NHBz
5ja
(1H NMR, 400 MHz, CDCl3)
f1 (ppm)

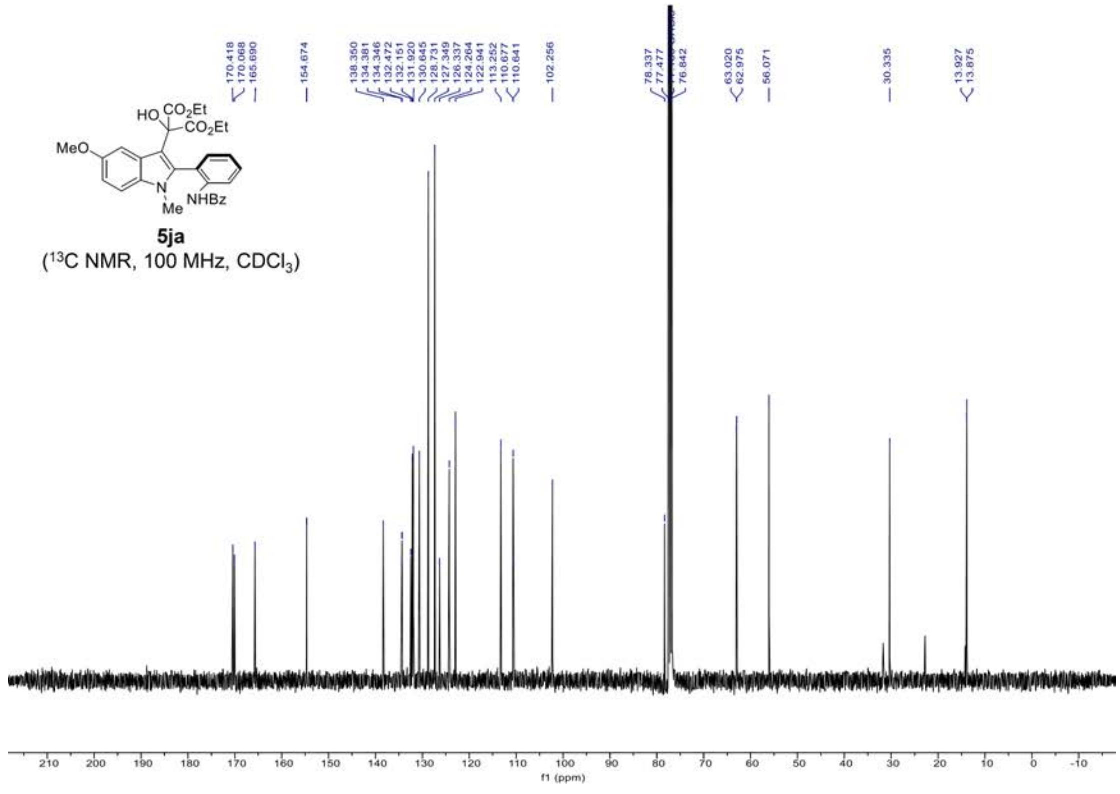
HO CO2Et
CO2Et
MeO
N
Me
NHBz
5ja
(13C NMR, 100 MHz, CDCl3)
f1 (ppm)

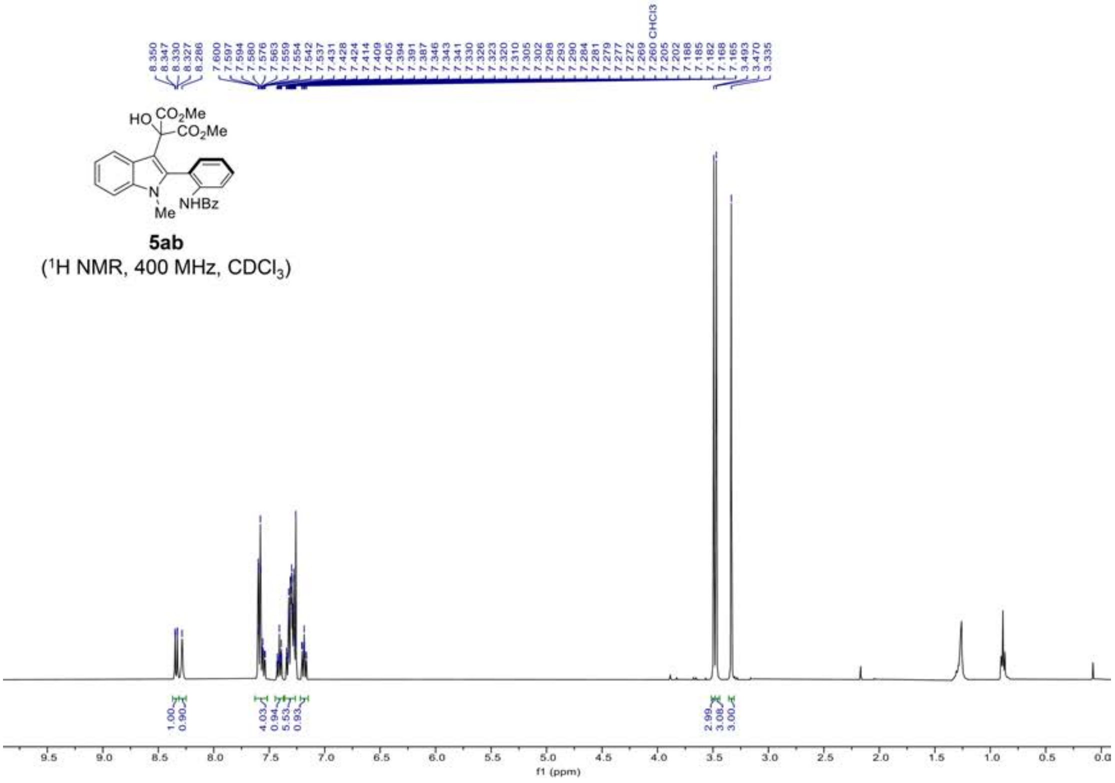

5ab
(^{1}H NMR, 400 MHz, $CDCl_3$)

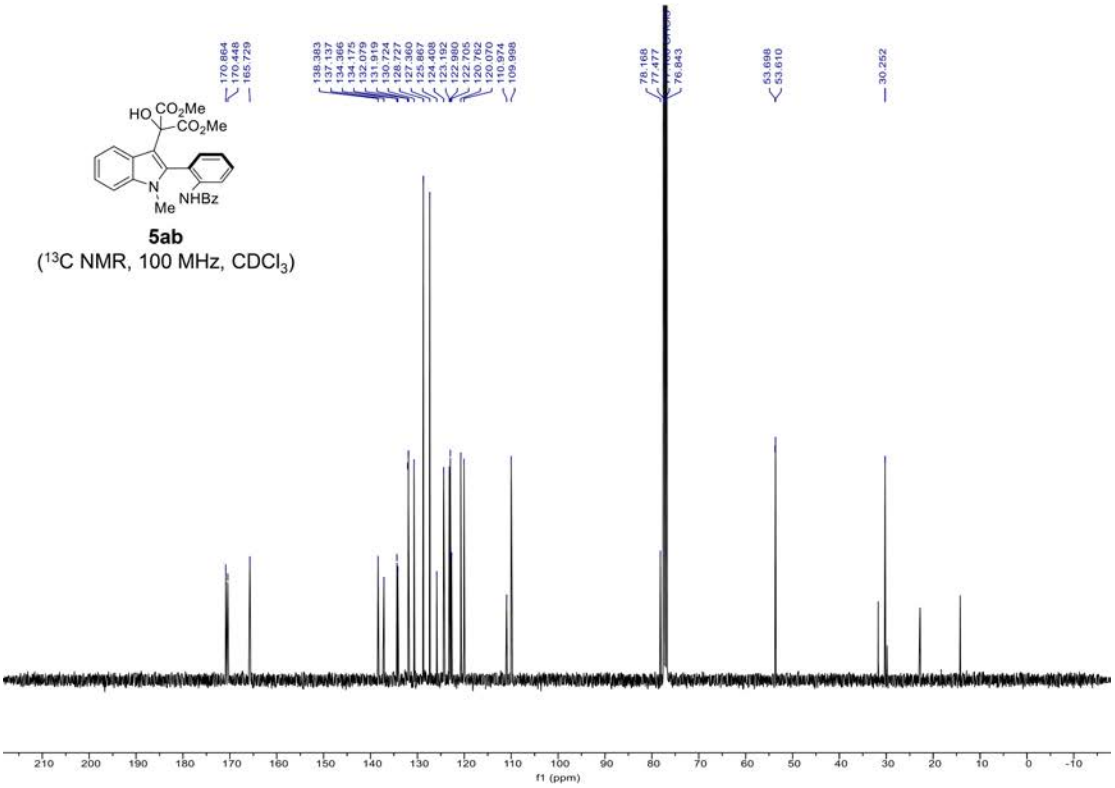

5ab
(^{13}C NMR, 100 MHz, $CDCl_3$)

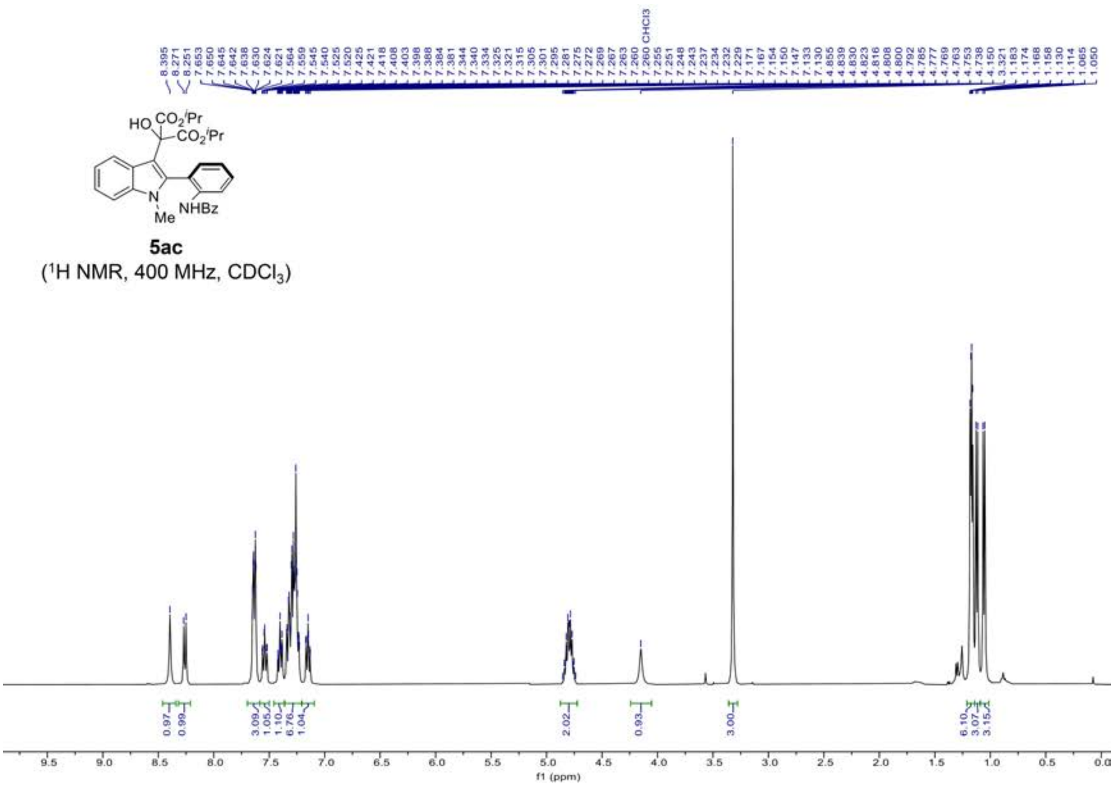

5ac
(^{1}H NMR, 400 MHz, $CDCl_3$)

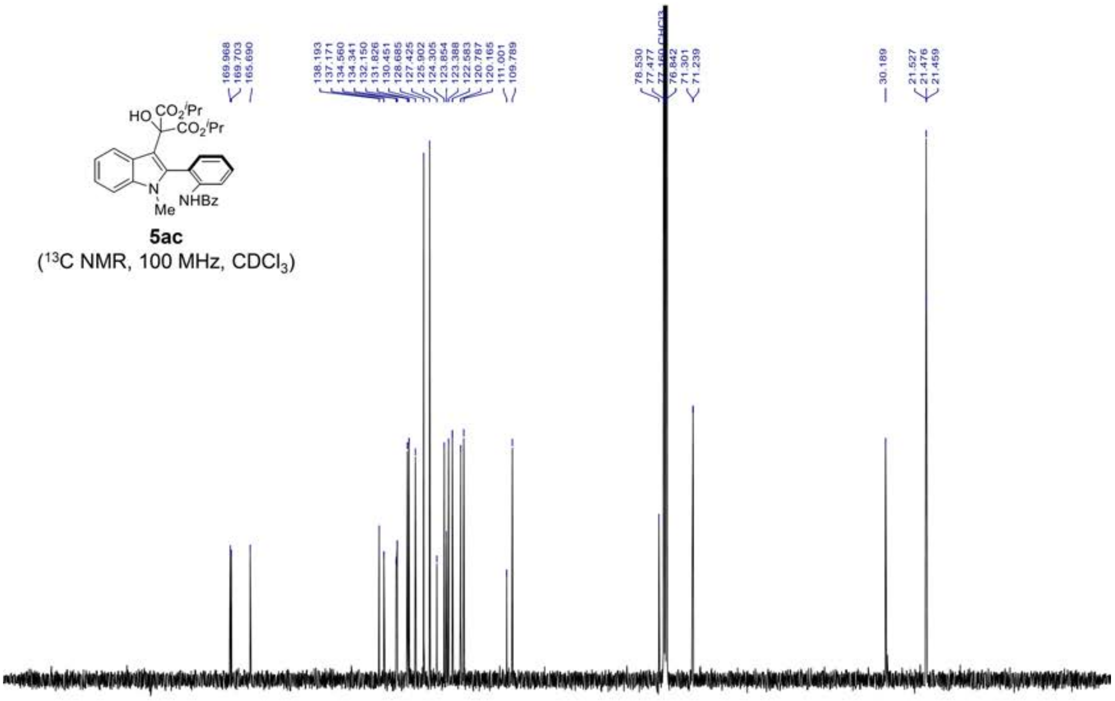

5ac
(^{13}C NMR, 100 MHz, $CDCl_3$)

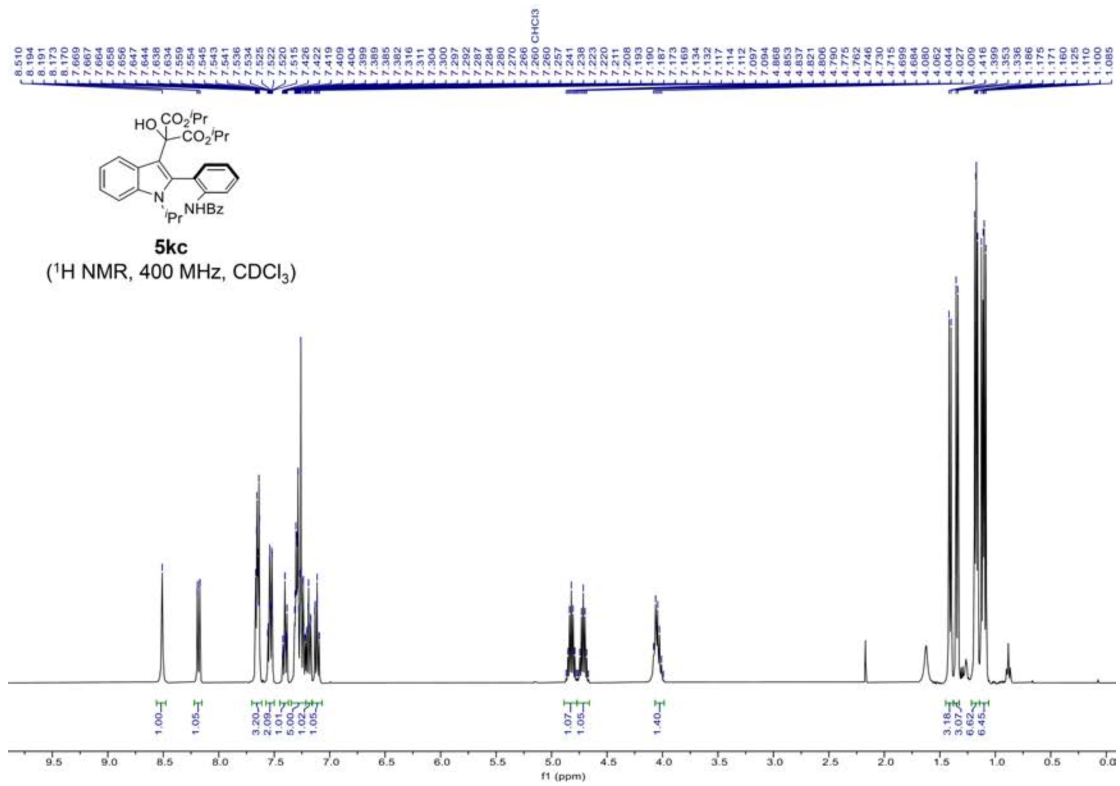

5kc
(^{1}H NMR, 400 MHz, $CDCl_3$)

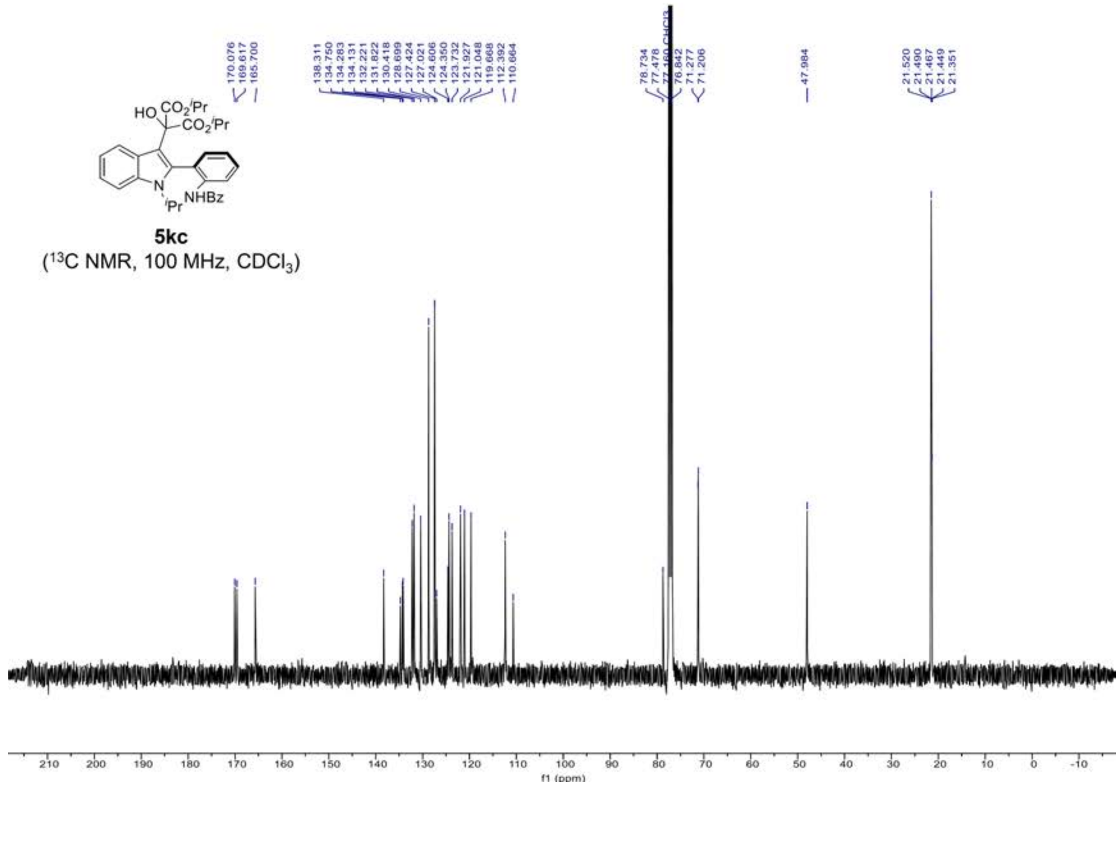

5kc
(^{13}C NMR, 100 MHz, $CDCl_3$)

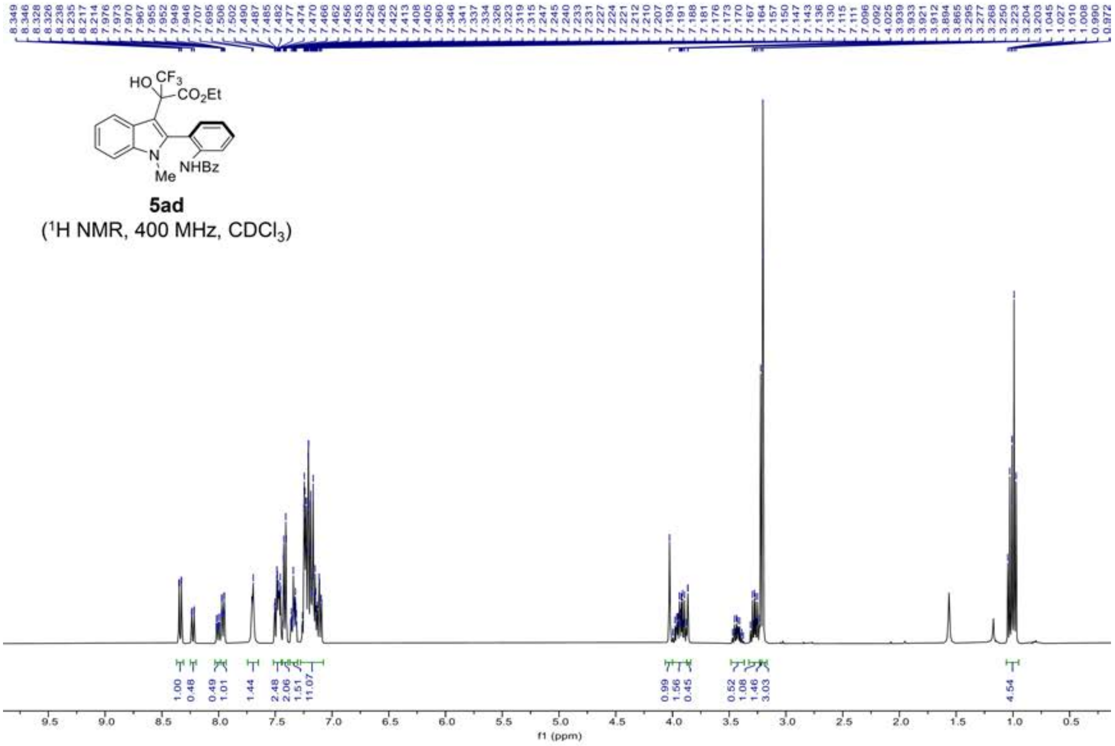
HO CF3
CO2Et
N
Me
NHBz
5ad
(1H NMR, 400 MHz, CDCl3)
f1 (ppm)

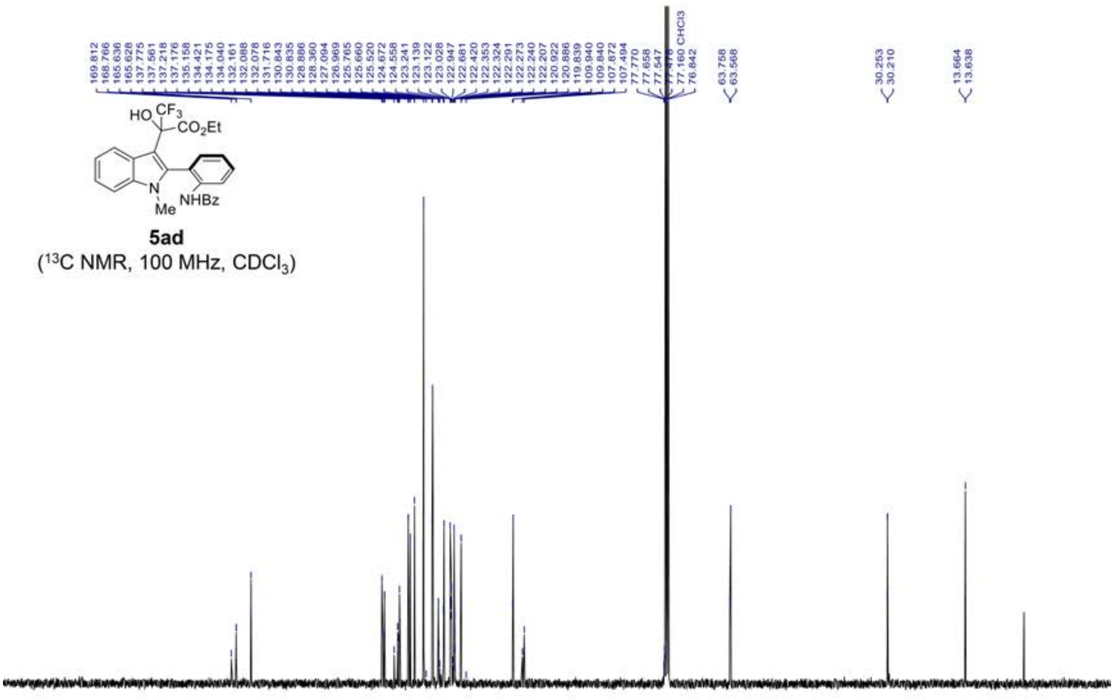
HO CF3
CO2Et
N
Me
NHBz
5ad
(13C NMR, 100 MHz, CDCl3)
f1 (ppm)

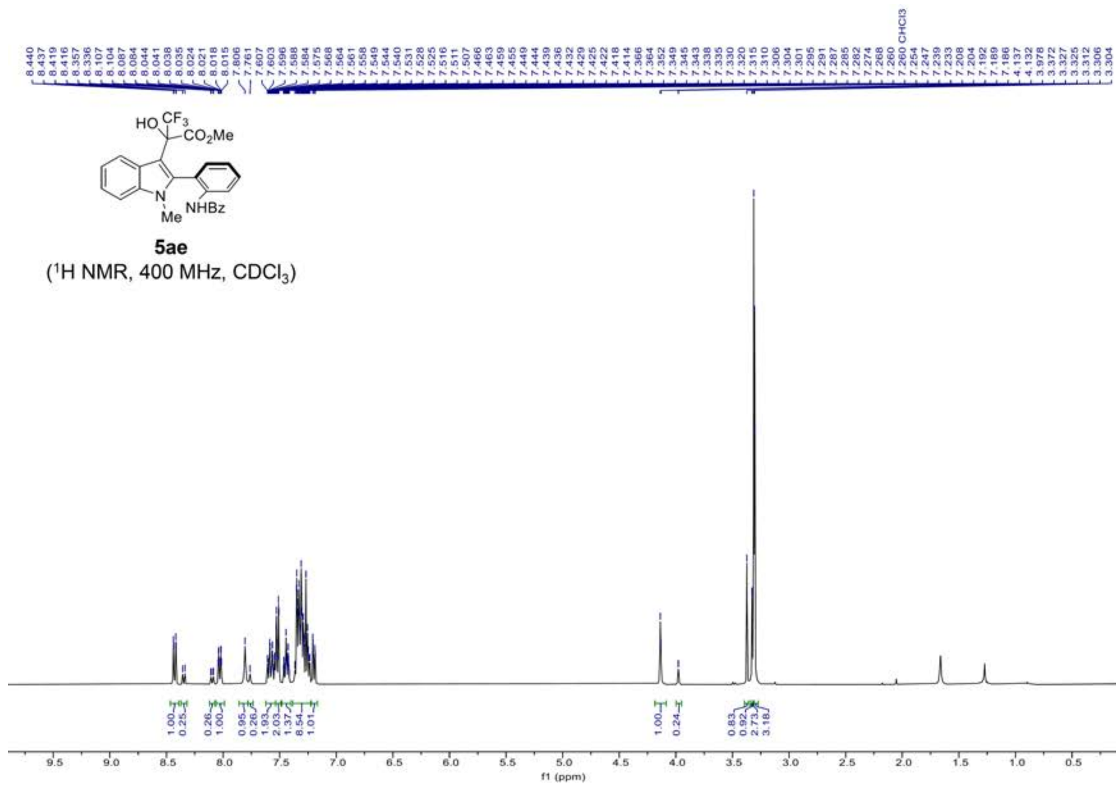
HO CF3 CO2Me
N Me NHBz
5ae
(1H NMR, 400 MHz, CDCl3)
f1 (ppm)

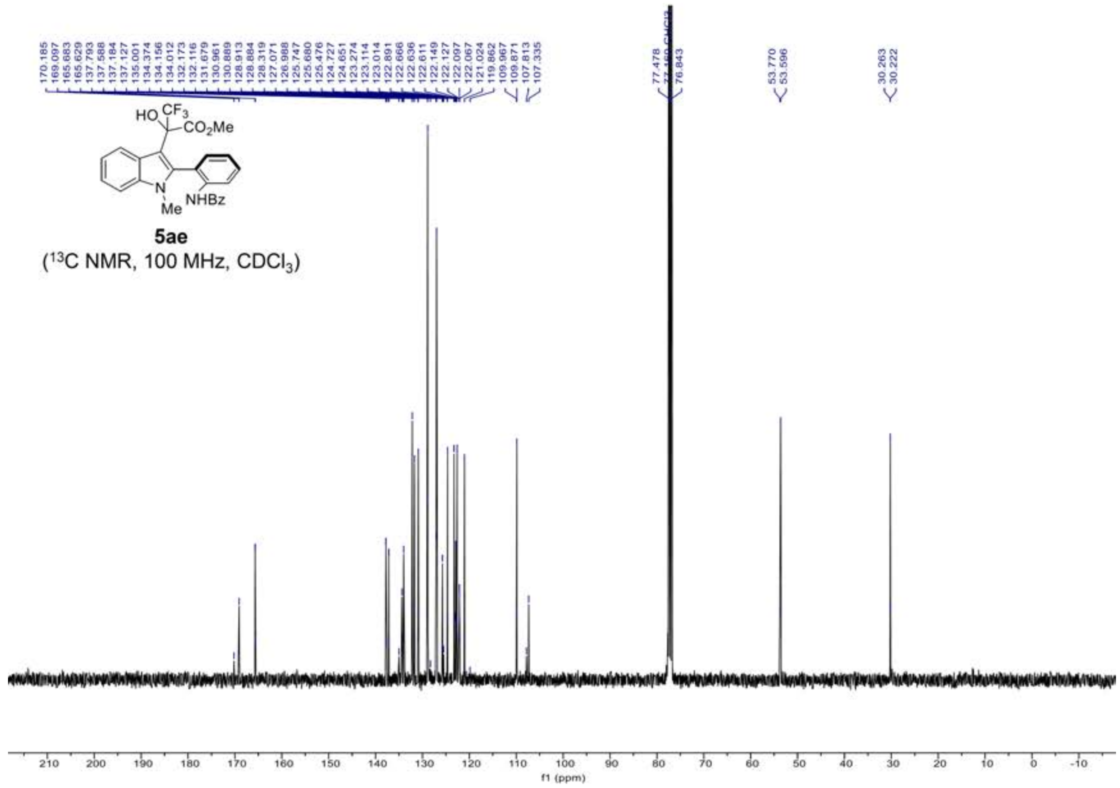
HO CF3 CO2Me
N Me NHBz
5ae
(13C NMR, 100 MHz, CDCl3)
f1 (ppm)

8.2 HPLC Traces of 5

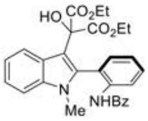

5aa

HPLC conditions:
Chiralpak AD-H, 20% ^{i}PrOH/n-Hexane eluent
1.0 mL/min, 254 nm

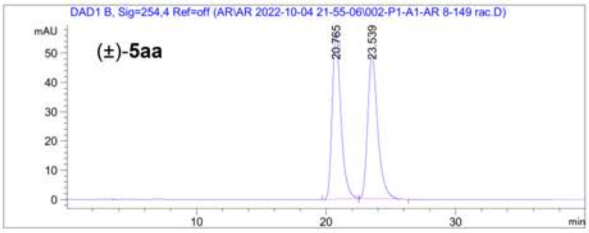

Peak #	RetTime [min]	Type	Width [min]	Area [mAU*s]	Height [mAU]	Area %
1	20.765	BB	0.7043	2645.03687	56.69687	50.0731
2	23.539	BB	0.8099	2637.31763	49.16257	49.9269
Totals :				5282.35449	105.85944	

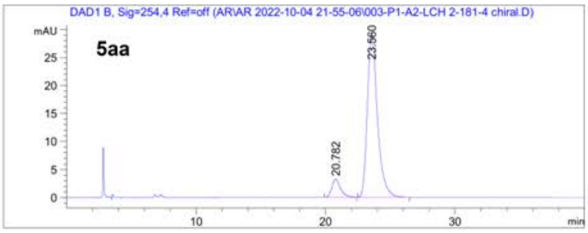

Peak #	RetTime [min]	Type	Width [min]	Area [mAU*s]	Height [mAU]	Area %
1	20.782	BB	0.6062	149.74442	3.23380	8.6461
2	23.560	BB	0.8137	1582.18311	29.31206	91.3539
Totals :				1731.92752	32.54586	

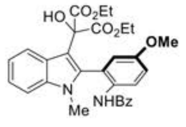

5ba

HPLC conditions:
Chiralpak AD-H, 10% ^{i}PrOH/n-Hexane eluent
1.0 mL/min, 254 nm

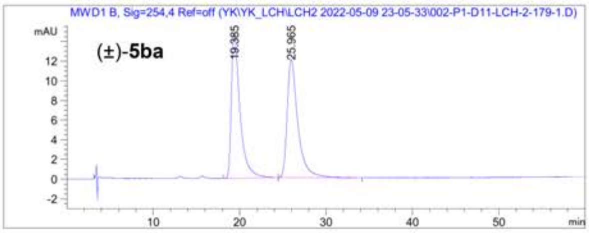

Peak #	RetTime [min]	Type	Width [min]	Area [mAU*s]	Height [mAU]	Area %
1	19.385	BB	1.0261	1033.71313	14.74067	49.8925
2	25.965	BB	1.2710	1038.16772	12.02790	50.1075
Totals :				2071.88086	26.76857	

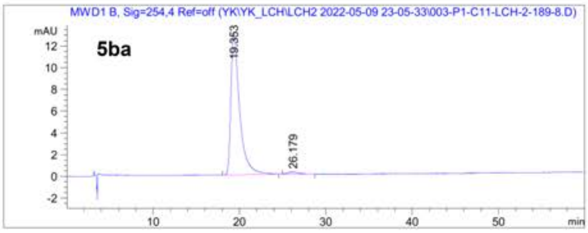

Peak #	RetTime [min]	Type	Width [min]	Area [mAU*s]	Height [mAU]	Area %
1	19.353	BB	1.0241	921.39728	13.04444	98.0830
2	26.179	BB	1.0459	18.00864	2.05605e-1	1.9170
Totals :				939.40592	13.25005	

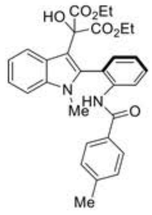

5ca

HPLC conditions:
Chiralpak AD-H, 20% ^{i}PrOH/n-Hexane eluent
1.0 mL/min, 254 nm

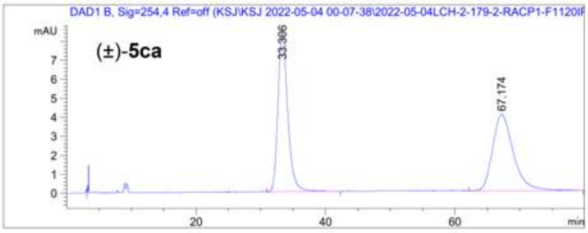

Peak #	RetTime [min]	Type	Width [min]	Area [mAU*s]	Height [mAU]	Area %
1	33.306	BB	1.6187	883.40051	8.34747	49.7604
2	67.174	BBA	3.4134	891.90863	4.00622	50.2396

Totals : 1775.30914 12.35369

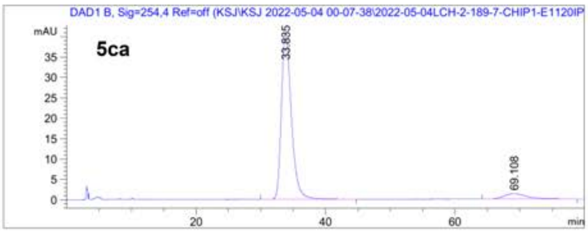

Peak #	RetTime [min]	Type	Width [min]	Area [mAU*s]	Height [mAU]	Area %
1	33.835	BV R	1.6459	4373.04102	40.68721	93.3736
2	69.108	BV R	2.9329	310.33926	1.30194	6.6264

Totals : 4683.38028 41.98915

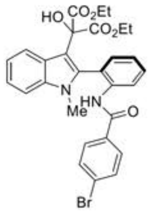

5da

HPLC conditions:
Chiralpak AD-H, 20% ^{i}PrOH/n-Hexane eluent
1.0 mL/min, 254 nm

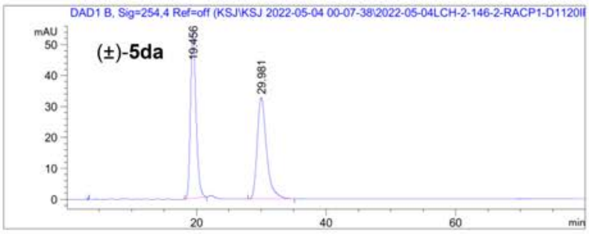

Peak #	RetTime [min]	Type	Width [min]	Area [mAU*s]	Height [mAU]	Area %
1	19.456	BB	0.9120	3168.37476	53.10804	49.0227
2	29.981	BB	1.4463	3294.69580	32.73203	50.9773
Totals :				6463.07056	85.84007	

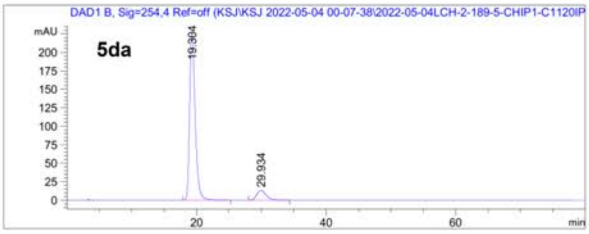

Peak #	RetTime [min]	Type	Width [min]	Area [mAU*s]	Height [mAU]	Area %
1	19.304	BB	0.9130	1.34277e4	225.39650	91.0650
2	29.934	BB	1.3496	1317.47363	13.27746	8.9350
Totals :				1.47451e4	238.67396	

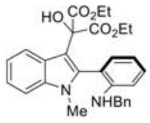

5ea

HPLC conditions:
Chiralpak AD-H, 20% ^{i}PrOH/n-Hexane eluent
1.0 mL/min, 254 nm

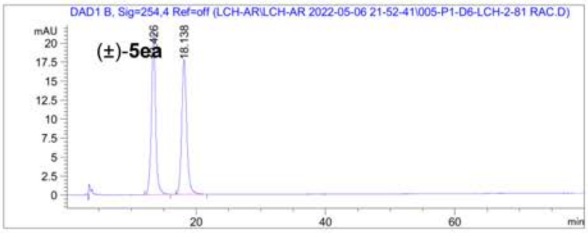

Peak #	RetTime [min]	Type	Width [min]	Area [mAU*s]	Height [mAU]	Area %
1	13.426	BB	0.6640	962.23450	21.26287	50.0947
2	18.138	BB	0.8061	958.59583	17.74806	49.9053
Totals :				1920.83032	39.01093	

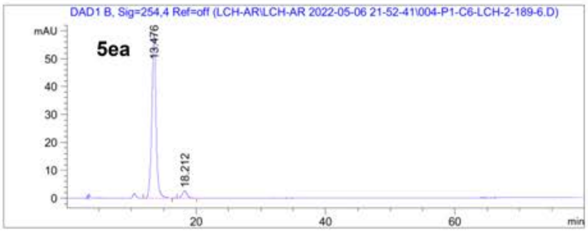

Peak #	RetTime [min]	Type	Width [min]	Area [mAU*s]	Height [mAU]	Area %
1	13.476	BB	0.6730	2679.22217	59.09135	95.1480
2	18.212	BB	0.7138	136.62396	2.63117	4.8520
Totals :				2815.84613	61.72252	

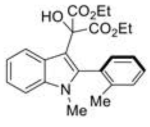

5fa

HPLC conditions:
Chiralpak AD-H, 20% ^{i}PrOH/n-Hexane eluent
1.0 mL/min, 254 nm

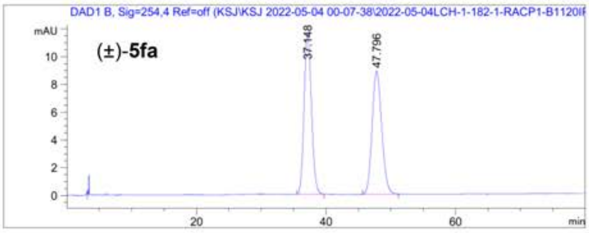

Peak #	RetTime [min]	Type	Width [min]	Area [mAU*s]	Height [mAU]	Area %
1	37.148	BB	1.0870	914.15308	11.60563	50.0121
2	47.796	BB	1.2469	913.71124	8.86212	49.9879
Totals :				1827.86432	20.46775	

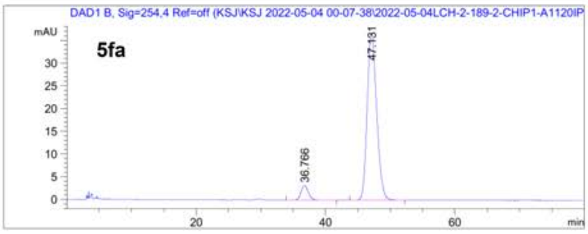

Peak #	RetTime [min]	Type	Width [min]	Area [mAU*s]	Height [mAU]	Area %
1	36.766	BB	1.3287	257.97864	3.18587	6.4693
2	47.131	BB	1.6372	3729.72925	36.59158	93.5307
Totals :				3987.70789	39.77746	

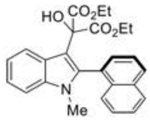

5ga

HPLC conditions:
Chiralpak AD-H, 20% ^{i}PrOH/n-Hexane eluent
1.0 mL/min, 254 nm

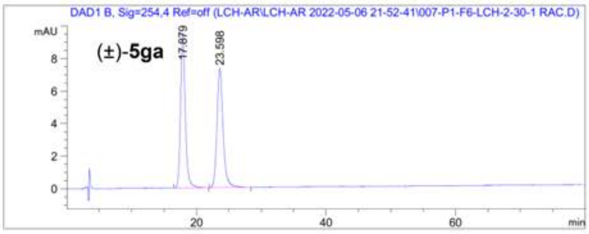

Peak #	RetTime [min]	Type	Width [min]	Area [mAU*s]	Height [mAU]	Area %
1	17.879	BB	0.7835	479.98581	9.46784	49.9398
2	23.598	BB	1.0078	481.14285	7.31114	50.0602
Totals :				961.12866	16.77898	

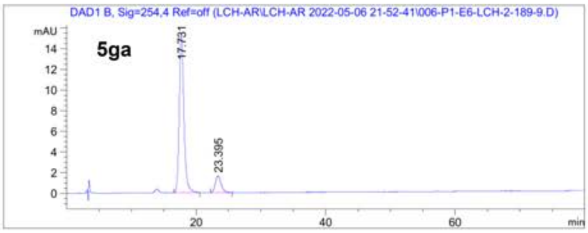

Peak #	RetTime [min]	Type	Width [min]	Area [mAU*s]	Height [mAU]	Area %
1	17.731	BB	0.7444	766.23547	15.40345	88.4505
2	23.395	BB	0.7370	100.05226	1.61454	11.5495
Totals :				866.28773	17.01800	

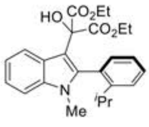

5ha

HPLC conditions:
Chiralpak AD-H, 10% ^{i}PrOH/n-Hexane eluent
1.0 mL/min, 254 nm

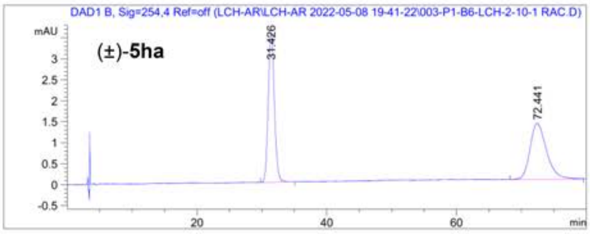

Peak #	RetTime [min]	Type	Width [min]	Area [mAU*s]	Height [mAU]	Area %
1	31.426	BB	1.0241	233.31718	3.54407	49.9128
2	72.441	BBA	2.6353	234.13286	1.33682	50.0872
Totals :				467.45004	4.88089	

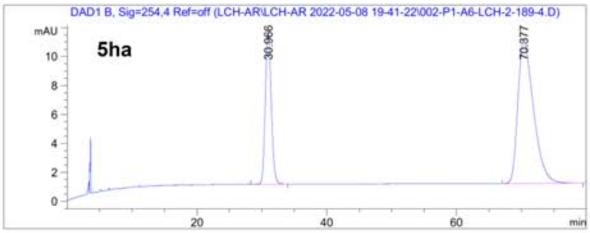

Peak #	RetTime [min]	Type	Width [min]	Area [mAU*s]	Height [mAU]	Area %
1	30.966	BB	0.9858	665.74457	10.41473	28.6755
2	70.377	BBA	2.5968	1655.90137	9.79241	71.3245
Totals :				2321.64594	20.20714	

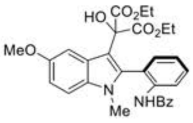

5ja

HPLC conditions:
Chiralpak AD-H, 20% ^{i}PrOH/n-Hexane eluent
1.0 mL/min, 254 nm

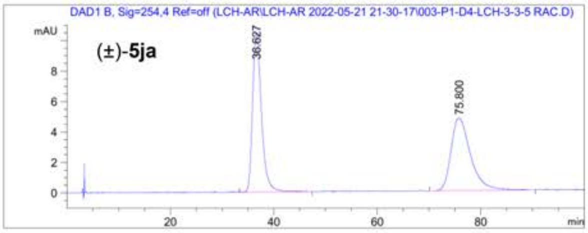

Peak #	RetTime [min]	Type	Width [min]	Area [mAU*s]	Height [mAU]	Area %
1	36.627	BB	1.8355	1254.68811	10.42997	50.1374
2	75.800	BB	3.8968	1247.81311	4.77492	49.8626

Totals : 2502.50122 15.20489

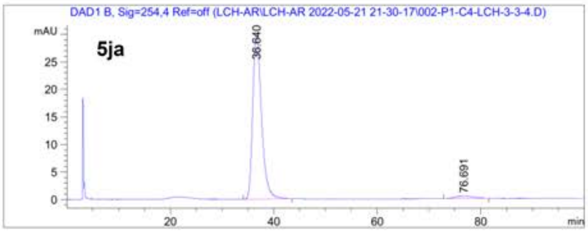

Peak #	RetTime [min]	Type	Width [min]	Area [mAU*s]	Height [mAU]	Area %
1	36.640	BV R	1.7712	3542.37866	30.13892	96.9842
2	76.691	BB	2.8445	110.15409	4.53072e-1	3.0158

Totals : 3652.53275 30.59199

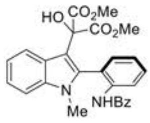

5ab

HPLC conditions:
Chiralpak IC, 40% ^{i}PrOH/n-Hexane eluent
1.0 mL/min, 254 nm

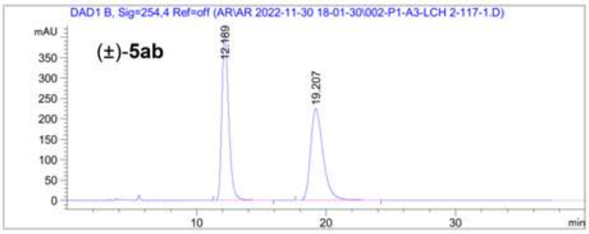

Peak #	RetTime [min]	Type	Width [min]	Area [mAU*s]	Height [mAU]	Area %
1	12.189	BB	0.5658	1.50236e4	407.82159	50.1298
2	19.207	BB	1.0187	1.49458e4	223.93236	49.8702
Totals :				2.99693e4	631.75395	

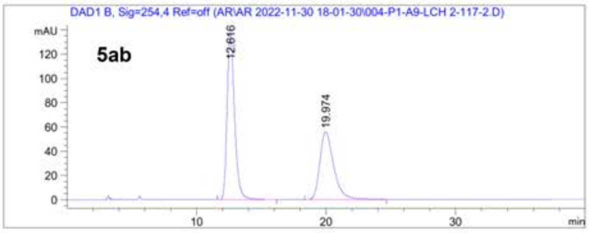

Peak #	RetTime [min]	Type	Width [min]	Area [mAU*s]	Height [mAU]	Area %
1	12.616	BB	0.6118	5501.88721	137.21687	57.0830
2	19.974	BB	1.1037	4136.50732	56.00420	42.9170
Totals :				9638.39453	193.22107	

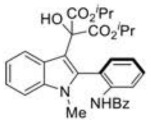

5ac

HPLC conditions:
Chiralpak AD, 20% ^{i}PrOH/*n*-Hexane eluent
1.0 mL/min, 254 nm

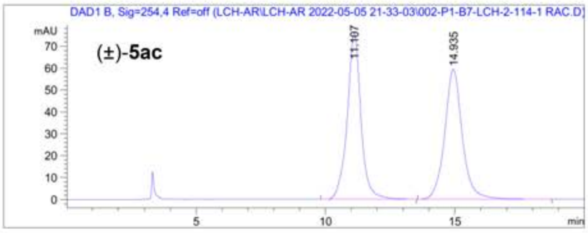

Peak #	RetTime [min]	Type	Width [min]	Area [mAU*s]	Height [mAU]	Area %
1	11.107	BB	0.5441	2814.02319	75.97202	50.0196
2	14.935	BB	0.7063	2811.81592	59.39902	49.9804
Totals :				5625.83911	135.37105	

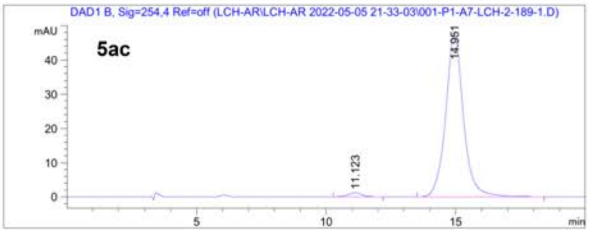

Peak #	RetTime [min]	Type	Width [min]	Area [mAU*s]	Height [mAU]	Area %
1	11.123	BB	0.4492	43.99681	1.23036	1.8991
2	14.951	BB	0.7070	2272.74731	47.77846	98.1009
Totals :				2316.74413	49.00882	

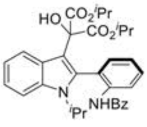

5kc

HPLC conditions:
Chiralpak IC, 20% ^{i}PrOH/n-Hexane eluent
1.0 mL/min, 254 nm

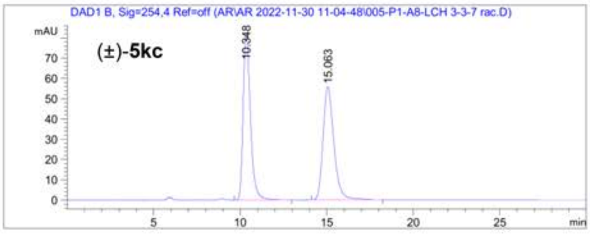

Peak #	RetTime [min]	Type	Width [min]	Area [mAU*s]	Height [mAU]	Area %
1	10.348	BB	0.4462	2412.00171	82.22081	49.8773
2	15.063	BB	0.6652	2423.86670	55.75922	50.1227
Totals :				4835.86841	137.98003	

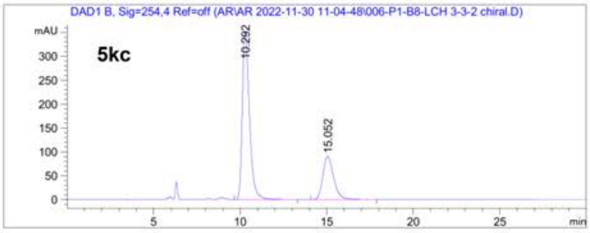

Peak #	RetTime [min]	Type	Width [min]	Area [mAU*s]	Height [mAU]	Area %
1	10.292	BB	0.4383	1.00618e4	349.06750	72.1052
2	15.052	BB	0.6649	3892.52222	90.30524	27.8948
Totals :				1.39543e4	439.37274	

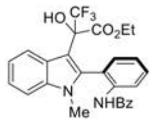

5ad

HPLC conditions:
Chiralpak OD-H, 10% ^{i}PrOH/n-Hexane eluent
1.0 mL/min, 254 nm

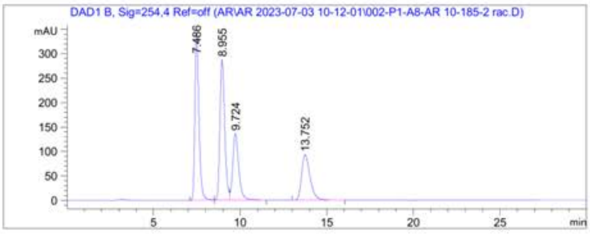

Peak #	RetTime [min]	Type	Width [min]	Area [mAU*s]	Height [mAU]	Area %
1	7.486	BV	0.2492	5600.48730	341.68011	31.8983
2	8.955	VV	0.2965	5528.38818	287.26691	31.4877
3	9.724	VB	0.3656	3274.28540	135.63324	18.6491
4	13.752	BB	0.5195	3154.13770	92.56293	17.9648
Totals :				1.75573e4	857.14319	

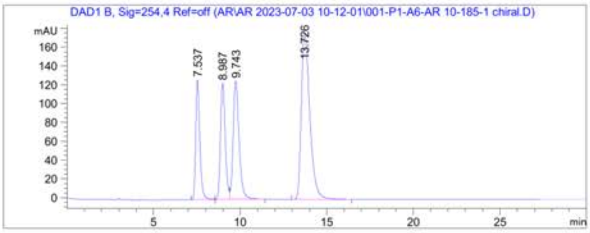

Peak #	RetTime [min]	Type	Width [min]	Area [mAU*s]	Height [mAU]	Area %
1	7.537	BB	0.2518	2089.48560	127.10181	15.4880
2	8.987	BV	0.2947	2346.90234	122.97232	17.3960
3	9.743	VB	0.3617	2986.56567	125.46176	22.1375
4	13.726	BB	0.5223	6068.05664	176.85013	44.9785
Totals :				1.34910e4	552.38602	

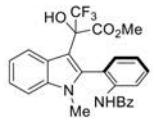

5ae

HPLC conditions:
Chiralpak OD-H, 10% ^{i}PrOH/n-Hexane eluent
1.0 mL/min, 254 nm

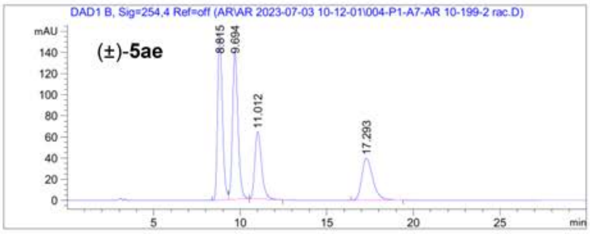

Peak #	RetTime [min]	Type	Width [min]	Area [mAU*s]	Height [mAU]	Area %
1	8.815	BV	0.2973	3045.91870	157.70018	31.7084
2	9.694	VB	0.3386	3087.71509	140.38359	32.1435
3	11.012	BB	0.4108	1702.53796	63.50776	17.7236
4	17.293	BB	0.6739	1769.86658	39.71887	18.4245
Totals :				9606.03833	401.31040	

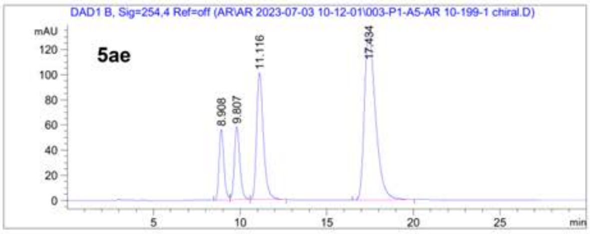

Peak #	RetTime [min]	Type	Width [min]	Area [mAU*s]	Height [mAU]	Area %
1	8.908	BV	0.2975	1075.50000	56.14376	9.7409
2	9.807	VB	0.3371	1263.92017	57.80004	11.4474
3	11.116	BB	0.4120	2724.64697	100.60279	24.6773
4	17.434	BB	0.6800	5977.04248	132.56885	54.1344
Totals :				1.10411e4	347.11544	

9 Characterization and Spectra of 7

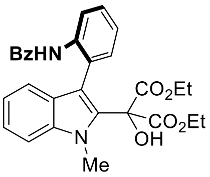

Diethyl 2-(3-(2-Benzamidophenyl)-1-methyl-1*H*-indol-2-yl)-2-hydroxymalonate (7aa) was synthesized by following Procedure 23 from **6a**. The crude material was purified by normal-phase column chromatography using an eluent of 5% EtOAc/CH_2Cl_2 to give **7aa** (65% yield).

^{1}H NMR (400 MHz, $CDCl_3$) δ 8.66 (d, *J* = 8.2 Hz, 1H), 8.06 (s, 1H), 7.50–7.30 (m, 6H), 7.26–7.20 (m, 3H), 7.20–7.13 (m, 2H), 7.07 (ddd, *J* = 7.9, 6.8, 1.0 Hz, 1H), 4.37 (brs, 1H), 3.93 (dqd, *J* = 10.7, 7.1, 5.5 Hz, 2H), 3.87–3.71 (m, 4H), 3.26 (dq, *J* = 10.7, 7.2 Hz, 1H), 1.07 (t, *J* = 7.1 Hz, 3H), 0.99 (t, *J* = 7.1 Hz, 3H).
^{13}C NMR (100 MHz, $CDCl_3$) δ 168.8, 168.6, 165.3, 138.0, 137.4, 135.2, 132.8, 131.7, 131.6, 129.1, 128.6 (2C), 127.0 (3C), 124.1, 123.5, 123.2, 120.8, 120.2, 119.8, 111.4, 109.5, 77.3, 63.8, 63.3, 31.7, 13.8, 13.6.
IR (FT-ATR, cm^{-1}, $CHCl_3$) ν_{max} 3984, 3899, 3865, 3842, 3799, 3780, 3730, 3699, 3626, 3599, 3460, 3394, 3163, 3059, 3012, 2981, 2939, 2607, 2422, 2360, 2337, 2295, 2268, 2137, 1963, 1925, 1809, 1739, 1666, 1608, 1581, 1516, 1446, 1365, 1308, 1250, 1207, 1103, 1022, 941, 895, 856, 744, 710, 667.
HRMS (EI) *m*/*z*: $[M]^+$ Calcd for $C_{29}H_{28}N_2O_6$ 500.1947; found 500.1943.
Optical: $[\alpha]^{20}_D$ = +20.3° (*c* = 1.62, $CHCl_3$, 89% e.e.)
HPLC (Chiralpak IC, ^{i}PrOH/*n*-hexane = 30/70, flow rate = 1.0 mL/min, λ = 254 nm) t_R = 8.8 min (major), 10.9 min (minor)

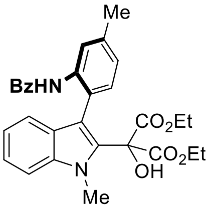

Diethyl 2-(3-(2-Benzamido-4-methylphenyl)-1-methyl-1*H*-indol-2-yl)-2-hydroxymalonate (7ba) was synthesized by following Procedure 23 from **6b**. The crude material was purified by normal-phase column chromatography using an eluent of 5% EtOAc/CH_2Cl_2 to give **7ba** (87% yield).

^{1}H NMR (400 MHz, $CDCl_3$) δ 8.51 (s, 1H), 8.02 (s, 1H), 7.41 (d, *J* = 8.3 Hz, 1H), 7.39–7.28 (m, 4H), 7.22 (t, *J* = 7.7 Hz, 2H), 7.17 (d, *J* = 7.9 Hz, 1H), 7.12 (d, *J* = 7.7 Hz, 1H), 7.06 (t, *J* = 7.5 Hz, 1H), 6.98 (dd, *J* = 7.7, 1.8 Hz, 1H), 4.37 (brs, 1H), 3.94 (dq, *J* = 10.7, 7.1 Hz, 2H), 3.88–3.72 (m, 4H), 3.29 (dq, *J* = 10.7, 7.2 Hz, 1H), 2.45 (s, 3H), 1.07 (t, *J* = 7.2 Hz, 3H), 0.99 (t, *J* = 7.1 Hz, 3H).
^{13}C NMR (100 MHz, $CDCl_3$) δ 168.9, 168.7, 165.2, 139.1, 137.7, 137.4, 135.2, 132.6, 131.8, 131.5, 128.6 (2C), 127.2, 127.0 (2C), 124.2, 124.0, 120.7, 120.3, 120.24, 120.19, 111.4, 109.4, 77.3, 63.8, 63.2, 31.7, 21.8, 13.7, 13.6.

IR (FT-ATR, cm^{-1}, $CHCl_3$) ν_{max} 3934, 3907, 3884, 3845, 3822, 3806, 3780, 3757, 3718, 3680, 3656, 3629, 3618, 3595, 3572, 3467, 3398, 3147, 3059, 2978, 2927, 2858, 2603, 2372, 2345, 2310, 2067, 1894, 1743, 1674, 1608, 1574, 1523, 1466, 1423, 1365, 1296, 1261, 1211, 1169, 1107, 1026, 887, 860, 822, 744, 717, 710.
HRMS (EI) *m*/*z*: $[M]^+$ Calcd for $C_{30}H_{30}N_2O_6$ 514.2104; found 514.2100.
Optical: $[a]^{20}_D$ = +25.3° (*c* = 0.36, $CHCl_3$, 87% e.e.)
HPLC (Chiralpak IC, ^{i}PrOH/*n*-hexane = 10/90, flow rate = 1.0 mL/min, λ = 254 nm) t_R = 28.1 min (major), 25.8 min (minor)

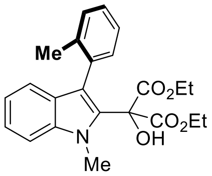

Diethyl 2-Hydroxy-2-(3-(*o*-tolyl)-1*H*-indol-2-yl)malonate (7ca) was synthesized by following Procedure 23 from **6c**. The crude material was purified by normal-phase column chromatography using an eluent of 5% EtOAc/CH_2Cl_2 to give **7ca** (37% yield).

^{1}H NMR (400 MHz, $CDCl_3$) δ 7.36 (dt, *J* = 8.3, 1.0 Hz, 1H), 7.30 (dd, *J* = 5.3, 2.9 Hz, 1H), 7.28–7.22 (m, 2H), 7.22–7.14 (m, 2H), 7.07–7.01 (m, 2H), 4.27 (brs, 1H), 4.05 (dq, *J* = 10.7, 7.1 Hz, 1H), 3.93 (dq, *J* = 10.8, 7.1 Hz, 1H), 3.82–3.72 (m, 4H), 3.30 (dq, *J* = 10.7, 7.2 Hz, 1H), 2.11 (s, 3H), 1.17 (t, *J* = 7.1 Hz, 3H), 1.11 (t, *J* = 7.1 Hz, 3H).
^{13}C NMR (100 MHz, $CDCl_3$) δ 169.1, 169.0, 139.4, 137.1, 133.3, 132.7, 129.8, 129.5, 127.8, 127.7, 125.1, 123.2, 120.5, 119.7, 116.6, 109.2, 77.3, 63.3, 63.0, 31.4, 20.5, 13.9, 13.7.
IR (FT-ATR, cm^{-1}, $CHCl_3$) ν_{max} 3961, 3899, 3845, 3799, 3730, 3699, 3626, 3595, 3475, 3055, 2981, 2931, 2854, 2735, 2607, 2553, 2480, 2407, 2360, 2337, 2295, 2268, 2133, 1886, 1809, 1736, 1608, 1577, 1554, 1466, 1365, 1323, 1265, 1207, 1146, 1095, 1022, 937, 860, 810, 741.
HRMS (EI) *m*/*z*: $[M]^+$ Calcd for $C_{23}H_{25}NO_5$ 395.1733; found 395.1729.
Optical: $[a]^{20}_D$ = –9.0° (*c* = 0.62, $CHCl_3$, 64% e.e.)
HPLC (Chiralpak IC, ^{i}PrOH/*n*-hexane = 5/95, flow rate = 1.0 mL/min, λ = 254 nm) t_R = 17.4 min (major), 25.4 min (minor)

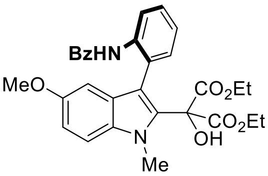

Diethyl 2-(3-(2-Benzamidophenyl)-5-methoxy-1-methyl-1*H*-indol-2-yl)-2-hydroxymalonate (7da) was synthesized by following Procedure 23 from **6d**. The crude material was purified by normal-phase column chromatography using an eluent of 5% EtOAc/CH_2Cl_2 to give **7da** (99% yield).

^{1}H NMR (400 MHz, $CDCl_3$) δ 8.64 (d, *J* = 8.0 Hz, 1H), 8.08 (s, 1H), 7.45 (ddd, *J* = 8.6, 7.4, 1.8 Hz, 1H), 7.37 (dt, *J* = 8.8, 2.0 Hz, 3H), 7.30 (d, *J* = 9.0 Hz, 1H), 7.28–7.22 (m, 4H), 7.17 (td, *J* = 7.4, 1.3 Hz, 1H), 6.97 (dd, *J* = 9.0, 2.5 Hz, 1H), 6.52 (d, *J* = 2.5

Hz, 1H), 4.32 (s, 1H), 3.92 (ddt, J = 10.1, 7.1, 3.5 Hz, 2H), 3.85–3.72 (m, 4H), 3.65 (s, 3H), 3.32–3.20 (m, 1H), 1.07 (t, J = 7.1 Hz, 3H), 1.00 (t, J = 7.1 Hz, 3H).
^{13}C NMR (100 MHz, $CDCl_3$) δ 168.9, 168.7, 165.3, 155.1, 137.9, 135.1, 132.9, 132.6, 131.9, 131.6, 129.2, 128.7 (2C), 127.2, 127.0 (2C), 123.6, 123.5, 120.0, 115.0, 111.0, 110.5, 100.8, 77.3, 63.8, 63.3, 56.0, 31.8, 13.8, 13.6.
IR (FT-ATR, cm^{-1}, $CHCl_3$) ν_{max} 3969, 3930, 3907, 3884, 3865, 3842, 3807, 3741, 3718, 3680, 3656, 3618, 3460, 3397, 3062, 2981, 2935, 2839, 2588, 2422, 2376, 2349, 2318, 2056, 1967, 1890, 1848, 1813, 1739, 1674, 1616, 1577, 1516, 1489, 1446, 1392, 1369, 1300, 1257, 1219, 1200, 1157, 1099, 1022, 891, 860, 798, 752, 710.
HRMS (EI) *m/z*: $[M]^+$ Calcd for $C_{30}H_{30}N_2O_7$ 530.2053; found 530.2053.
Optical: $[a]^{20}_D$ = +63.9° (c = 0.87, $CHCl_3$, 86% e.e.)
HPLC (Chiralpak IC, ^{i}PrOH/n-hexane = 30/70, flow rate = 1.0 mL/min, λ = 254 nm) t_R = 93.1 min (major), 6.9 min (minor)

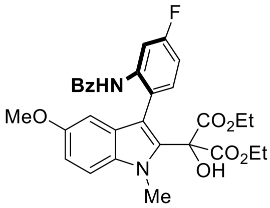

Diethyl 2-(3-(2-Benzamido-4-fluorophenyl)-5-methoxy-1-methyl-1*H*-indol-2-yl)-2-hydroxymalonate (7ea) was synthesized by following Procedure 23 from **6e**. The crude material was purified by normal-phase column chromatography using an eluent of 5% EtOAc/CH_2Cl_2 to give **7ea** (72% yield).

^{1}H NMR (400 MHz, $CDCl_3$) δ 8.51 (dd, J = 11.3, 2.7 Hz, 1H), 8.12 (s, 1H), 7.44–7.35 (m, 3H), 7.31 (d, J = 9.0 Hz, 1H), 7.29–7.23 (m, 2H), 7.20 (dd, J = 8.5, 6.4 Hz, 1H), 6.98 (dd, J = 9.0, 2.5 Hz, 1H), 6.88 (td, J = 8.2, 2.7 Hz, 1H), 6.49 (d, J = 2.4 Hz, 1H), 3.97 (ddq, J = 14.2, 10.7, 7.1 Hz, 2H), 3.87–3.73 (m, 4H), 3.66 (s, 3H), 3.40 (dq, J = 10.6, 7.1 Hz, 1H), 1.09 (t, J = 7.1 Hz, 3H), 1.01 (t, J = 7.1 Hz, 3H).
^{13}C NMR (100 MHz, $CDCl_3$) δ 168.8, 168.6, 165.4, 162.9 (d, J = 243.0 Hz, 1C), 155.2, 139.3 (d, J = 12.0 Hz, 1C), 134.6, 133.9 (d, J = 9.0 Hz, 1C), 132.6, 132.2, 131.9, 128.8 (2C), 127.3, 127.0 (2C), 118.8 (d, J = 3.0 Hz, 1C), 115.1, 110.6, 110.2 (d, J = 22.0 Hz, 1C), 109.9, 107.3 (d, J = 27.0 Hz, 1C), 100.6, 77.3, 63.9, 63.7, 63.3, 56.0, 31.9, 14.0, 13.8, 13.7.
IR (FT-ATR, cm^{-1}, $CHCl_3$) ν_{max} 3953, 3930, 3907, 3865, 3845, 3826, 3807, 3780, 3757, 3741, 3680, 3653, 3618, 3460, 3113, 3066, 2985, 2935, 2607, 2372, 2345, 2314, 2094, 1739, 1678, 1597, 1520, 1489, 1462, 1369, 1227, 1092, 1018, 860, 795, 717.
HRMS (EI) *m/z*: $[M]^+$ Calcd for $C_{30}H_{29}FN_2O_7$ 548.1959; found 548.1957.
Optical: $[a]^{20}_D$ = +110.3° (c = 1.89, $CHCl_3$, 79% e.e.)
HPLC (Chiralpak AD, ^{i}PrOH/n-hexane = 50/50, flow rate = 1.0 mL/min, λ = 254 nm) t_R = 5.8 min (major), 31.2 min (minor)

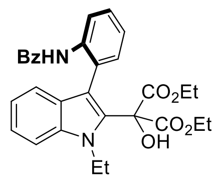

Diethyl 2-(3-(2-Benzamidophenyl)-1-ethyl-1*H*-indol-2-yl)-2-hydroxymalonate (7fa) was synthesized by following Procedure 23 from **6f**. The crude material was purified by normal-phase column chromatography using an eluent of 5% EtOAc/CH_2Cl_2 to give **7fa** (5% yield, –5 °C; 30% yield, 60 °C).

^{1}H NMR (400 MHz, $CDCl_3$) δ 8.65 (dd, *J* = 8.3, 1.2 Hz, 1H), 8.05 (s, 1H), 7.49–7.40 (m, 2H), 7.39–7.24 (m, 6H), 7.24–7.11 (m, 4H), 7.06 (ddd, *J* = 8.0, 6.9, 0.9 Hz, 1H), 4.39–4.14 (m, 3H), 4.01–3.82 (m, 2H), 3.63 (dq, *J* = 10.7, 7.1 Hz, 1H), 3.24 (dq, *J* = 10.7, 7.1 Hz, 1H), 1.47 (t, *J* = 7.0 Hz, 3H), 1.07 (t, *J* = 7.2 Hz, 3H), 0.94 (t, *J* = 7.1 Hz, 3H).
^{13}C NMR (100 MHz, $CDCl_3$) δ 169.0, 168.8, 165.2, 138.1, 136.2, 135.2, 132.8, 131.6, 131.0, 129.1, 128.6 (2C), 127.5, 127.0 (2C), 124.0, 123.4, 123.3, 120.7, 120.4, 119.7, 111.1, 110.0, 77.3, 63.8, 63.2, 40.4, 14.8, 13.70, 13.65.
IR (FT-ATR, cm^{-1}, $CHCl_3$) ν_{max} 3950, 3933, 3905, 3874, 3855, 3840, 3822, 3802, 3788, 3752, 3736, 3726, 3702, 3692, 3677, 3651, 3630, 3622, 3600, 3568, 3546, 3460, 3393, 3059, 3013, 2979, 2935, 2904, 2873, 2854, 2385, 2369, 2359, 2338, 2329, 2295, 1736, 1668, 1609, 1579, 1546, 1521, 1493, 1451, 1394, 1362, 1308, 1264, 1248, 1205, 1155, 1139, 1106, 1024, 932, 894, 858, 822, 795, 742, 708, 690, 668.
HRMS (EI) *m*/*z*: $[M]^+$ Calcd for $C_{30}H_{30}N_2O_6$ 514.2104; found 514.2106.
Optical: $[a]^{20}_D$ = +13.0° (*c* = 0.77, $CHCl_3$, 40% e.e.)
HPLC (Chiralpak IC, ^{i}PrOH/*n*-hexane = 30/70, flow rate = 1.0 mL/min, λ = 254 nm) t_R = 8.4 min (major), 11.3 min (minor)

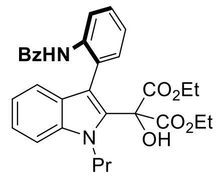

Diethyl 2-(3-(2-Benzamidophenyl)-1-propyl-1*H*-indol-2-yl)-2-hydroxymalonate (7ga) was synthesized by following Procedure 23 from **6g**. The crude material was purified by normal-phase column chromatography using an eluent of 5% EtOAc/CH_2Cl_2 to give **7ga** (18% yield, 60 °C).

^{1}H NMR (400 MHz, $CDCl_3$) δ 8.65 (dd, *J* = 8.3, 1.2 Hz, 1H), 8.05 (s, 1H), 7.47–7.39 (m, 2H), 7.39–7.33 (m, 1H), 7.33–7.24 (m, 4H), 7.24–7.11 (m, 4H), 7.05 (ddd, *J* = 7.9, 6.8, 1.0 Hz, 1H), 4.32 (s, 1H), 4.22–4.02 (m, 2H), 3.91 (ddq, *J* = 26.9, 10.8, 7.1 Hz, 2H), 3.61 (dq, *J* = 10.7, 7.1 Hz, 1H), 3.26 (dq, *J* = 10.7, 7.2 Hz, 1H), 2.05–1.81 (m, 2H), 1.07 (t, *J* = 7.2 Hz, 3H), 1.02 (t, *J* = 7.5 Hz, 3H), 0.94 (t, *J* = 7.1 Hz, 3H).
^{13}C NMR (100 MHz, $CDCl_3$) δ 169.0, 168.9, 165.2, 138.1, 136.6, 135.1, 132.8, 131.6, 131.1, 129.1, 128.6 (2C), 127.4, 127.0 (2C), 123.9, 123.4, 123.3, 120.7, 120.3, 119.7, 111.2, 110.2, 77.4, 63.8, 63.2, 47.4, 22.9, 13.67, 13.65, 11.6.
IR (FT-ATR, cm^{-1}, $CHCl_3$) ν_{max} 3950, 3933, 3925, 3905, 3874, 3855, 3822, 3792, 3736, 3727, 3702, 3692, 3677, 3630, 3596, 3569, 3546, 3504, 3455, 3393, 3059, 3014, 2966, 2933, 2875, 2401, 2385, 2369, 2359, 2348, 2338, 2295, 1898, 1736, 1668, 1610, 1579,

1546, 1522, 1494, 1451, 1395, 1354, 1306, 1269, 1249, 1214, 1155, 1139, 1105, 1080, 1021, 944, 894, 858, 795, 775, 741, 708, 691, 667.
HRMS (EI) *m/z*: $[M]^+$ Calcd for $C_{31}H_{32}N_2O_6$ 528.2260; found 528.2260.
Optical: $[a]^{20}_D$ = +17.5° (c = 0.44, $CHCl_3$, 22% e.e.)
HPLC (Chiralpak IC, ^{i}PrOH/n-hexane = 30/70, flow rate = 1.0 mL/min, λ = 254 nm) t_R = 6.6 min (major), 7.4 min (minor)

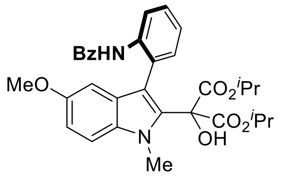

Diisopropyl 2-(3-(2-Benzamidophenyl)-5-methoxy-1-methyl-1*H*-indol-2-yl)-2-hydroxymalonate (7dc) was synthesized by following Procedure 23 from **6d**. The crude material was purified by normal-phase column chromatography using an eluent of 5% EtOAc/CH_2Cl_2 to give **7dc** (80% yield).

^{1}H NMR (400 MHz, $CDCl_3$) δ 8.59 (d, J = 8.0 Hz, 1H), 8.09 (s, 1H), 7.45 (ddd, J = 8.5, 7.5, 1.7 Hz, 1H), 7.41–7.32 (m, 3H), 7.32–7.20 (m, 4H), 7.16 (td, J = 7.5, 1.3 Hz, 1H), 6.95 (dd, J = 8.9, 2.4 Hz, 1H), 6.54 (d, J = 2.5 Hz, 1H), 4.72 (p, J = 6.2 Hz, 1H), 4.32 (qd, J = 9.3, 5.8 Hz, 2H), 3.77 (s, 3H), 3.63 (s, 3H), 1.15 (d, J = 6.1 Hz, 3H), 1.08–0.95 (m, 9H).
^{13}C NMR (100 MHz, $CDCl_3$) δ 168.44, 168.40, 165.4, 155.1, 137.8, 135.2, 133.0, 132.7, 132.1, 131.5, 129.0, 128.6 (2C), 127.1, 127.0 (2C), 123.7, 123.5, 120.3, 114.8, 111.2, 110.5, 100.8, 77.6, 72.7, 71.5, 56.0, 31.9, 21.4, 21.3 (2C), 21.2.
IR (FT-ATR, cm^{-1}, $CHCl_3$) ν_{max} 3946, 3926, 3899, 3869, 3842, 3803, 3780, 3730, 3699, 3626, 3599, 3456, 3394, 3163, 3062, 2981, 2939, 2839, 2360, 2333, 2295, 2268, 1967, 1847, 1732, 1670, 1616, 1577, 1515, 1489, 1450, 1373, 1304, 1269, 1223, 1157, 1095, 1022, 906, 829, 798, 748, 710, 667.
HRMS (EI) *m/z*: $[M]^+$ Calcd for $C_{32}H_{34}N_2O_7$ 558.2366; found 558.2368.
Optical: $[a]^{20}_D$ = +44.9° (c = 1.78, $CHCl_3$, 79% e.e.)
HPLC (Chiralpak IC, ^{i}PrOH/n-hexane = 20/80, flow rate = 1.0 mL/min, λ = 254 nm) t_R = 13.5 min (major), 16.1 min (minor)

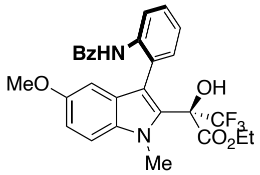

Ethyl 2-(3-(2-Benzamidophenyl)-5-methoxy-1-methyl-1*H*-indol-2-yl)-3,3,3-trifluoro-2-hydroxypropanoate (7dd) was synthesized by following Procedure 23 from **6d**. The crude material was purified by normal-phase column chromatography using an eluent of 5% EtOAc/CH_2Cl_2 to give **7dd** (99% yield, 20:1 d.r.).

^{1}H NMR (400 MHz, $CDCl_3$) δ 8.58 (dd, J = 8.4, 1.2 Hz, 1H), 7.87 (s, 1H), 7.46 (td, J = 7.9, 1.7 Hz, 1H), 7.43–7.14 (m, 7H), 7.09 (dd, J = 7.6, 1.7 Hz, 1H), 6.99 (dd, J = 9.0, 2.5 Hz, 1H), 6.40 (d, J = 2.5 Hz, 1H), 4.63 (s, 1H), 4.03 (s, 3H), 3.92 (dq, J = 10.6, 7.2 Hz, 1H), 3.64 (s, 3H), 3.05 (dtd, J = 14.4, 7.2, 3.8 Hz, 1H), 1.02 (t, J = 7.1 Hz, 3H).

^{13}**C NMR** (100 MHz, $CDCl_3$) δ 167.7, 165.6, 155.3, 137.6, 134.8, 133.6, 132.3, 131.7, 129.2, 128.7 (2C), 127.6, 126.8 (2C), 125.8 (q, J = 225.2 Hz, 1C), 124.7, 124.1, 120.9, 115.7, 113.2, 111.0, 100.5, 77.2 (q, J = 31.5 Hz, 1C), 64.3, 56.0, 33.3 (q, J = 4.3 Hz, 1C), 13.4.
IR (FT-ATR, cm^{-1}, $CHCl_3$) ν_{max} 3969, 3946, 3926, 3899, 3865, 3838, 3803, 3780, 3730, 3699, 3626, 3595, 3568, 3402, 3298, 3163, 3066, 3005, 2947, 2839, 2407, 2360, 2337, 2295, 2268, 2060, 1967, 1894, 1867, 1747, 1658, 1620, 1577, 1516, 1489, 1446, 1369, 1273, 1227, 1188, 1157, 1111, 1026, 984, 899, 837, 798, 752, 710, 683.
HRMS (EI) *m*/*z*: $[M]^+$ Calcd for $C_{28}H_{25}F_3N_2O_5$ 526.1716; found 526.1715.
Optical: $[a]^{20}_D$ = +32.3° (c = 2.55, $CHCl_3$, >20:1 d.r., 86% e.e.)
HPLC (Chiralpak AD-H, ^{i}PrOH/*n*-hexane = 30/70, flow rate = 1.0 mL/min, λ = 254 nm) t_R = 26.9 min (major diastereomer), 8.9 min (minor enantiomer)

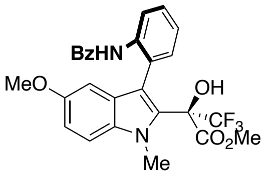

Methyl 2-(3-(2-Benzamidophenyl)-5-methoxy-1-methyl-1*H*-indol-2-yl)-3,3,3-trifluoro-2-hydroxypropanoate (7de) was synthesized by following Procedure 23 from **6d**. The crude material was purified by normal-phase column chromatography using an eluent of 5% EtOAc/CH_2Cl_2 to give **7de** (99% yield, 9:1 d.r.).

^{1}**H NMR** (400 MHz, $CDCl_3$) δ 8.53–8.47 (m, 1H), 7.82 (s, 1H), 7.44 (ddd, J = 8.7, 7.6, 1.6 Hz, 1H), 7.41–7.35 (m, 1H), 7.35–7.28 (m, 3H), 7.28–7.16 (m, 3H), 7.07 (dd, J = 7.5, 1.6 Hz, 1H), 6.98 (dd, J = 9.0, 2.5 Hz, 1H), 6.38 (d, J = 2.5 Hz, 1H), 4.69 (s, 1H), 4.00 (d, J = 1.3 Hz, 3H), 3.63 (s, 3H), 3.15 (s, 3H).
^{13}**C NMR** (100 MHz, $CDCl_3$) δ 168.2, 165.7, 155.2, 137.5, 134.6, 133.6, 132.2, 131.8, 129.2, 128.7 (2C), 127.6, 127.4, 126.8 (2C), 124.9, 124.2, 121.7 (q, J = 242.0 Hz, 1C), 121.3, 115.7, 113.2, 111.0, 100.5, 76.7 (q, J = 32.0 Hz, 1C), 55.9, 54.2, 33.3 (q, J = 4.0 Hz, 1C).
IR (FT-ATR, cm^{-1}, $CHCl_3$) ν_{max} 3950, 3925, 3905, 3874, 3855, 3840, 3822, 3785, 3752, 3737, 3727, 3712, 3703, 3691, 3677, 3651, 3630, 3620, 3596, 3569, 3553, 3504, 3401, 3285, 3066, 3015, 2954, 2851, 2360, 2339, 2328, 2295, 1752, 1654, 1619, 1578, 1578, 1537, 1512, 1489, 1449, 1438, 1359, 1291, 1271, 1250, 1224, 1187, 1159, 1140, 1113, 1078, 1028, 1001, 892, 960, 930, 907, 874, 831, 795, 776, 752, 708, 691.
HRMS (EI) *m*/*z*: $[M]^+$ Calcd for $C_{27}H_{23}F_3N_2O_5$ 512.1559; found 512.1558.
Optical: $[a]^{20}_D$ = +70.6° (c = 2.57, $CHCl_3$, >9:1 d.r., 77% e.e.)
HPLC (Chiralpak AD-H, ^{i}PrOH/*n*-hexane = 2/98, flow rate = 1.0 mL/min, λ = 254 nm) t_R = 41.3 min (major diastereomer), 46.9 min (minor enantiomer)

9.1 NMR Spectra of 7

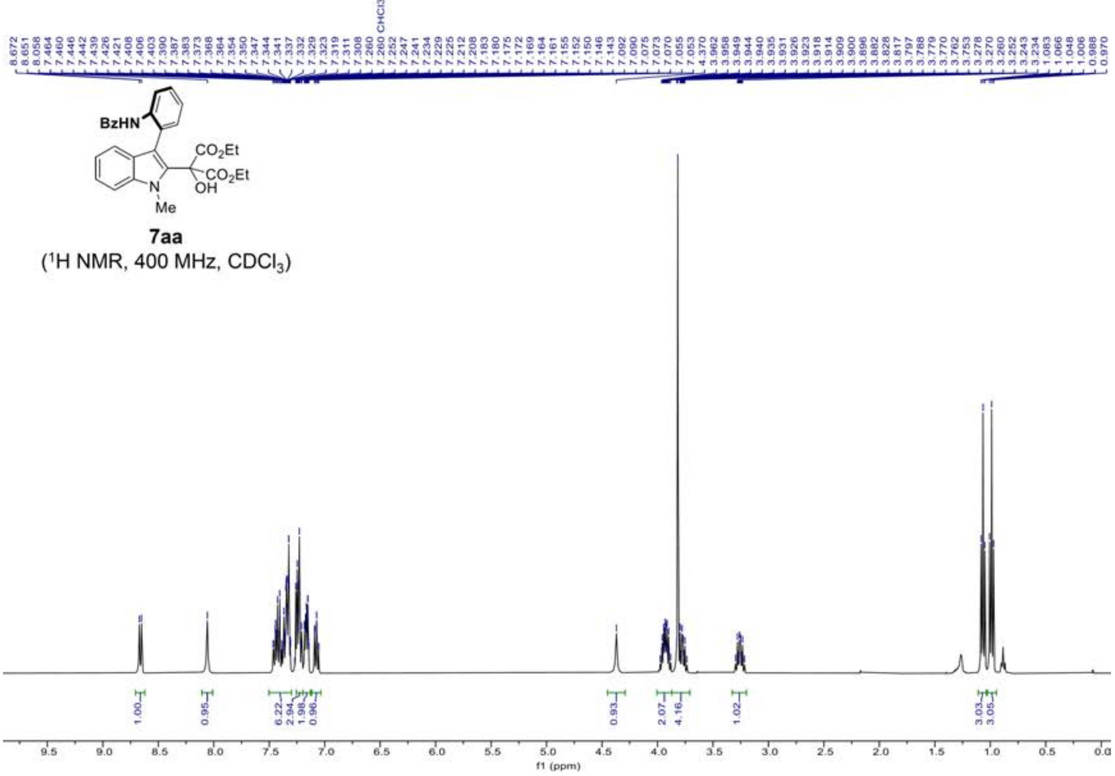

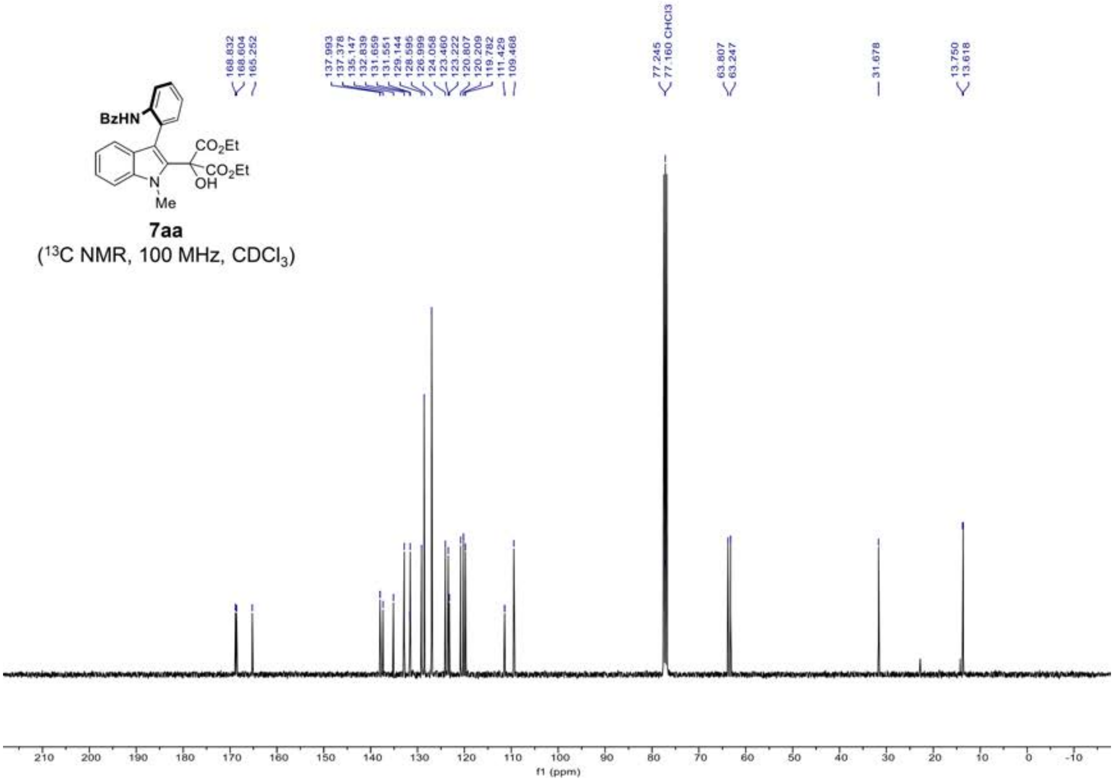

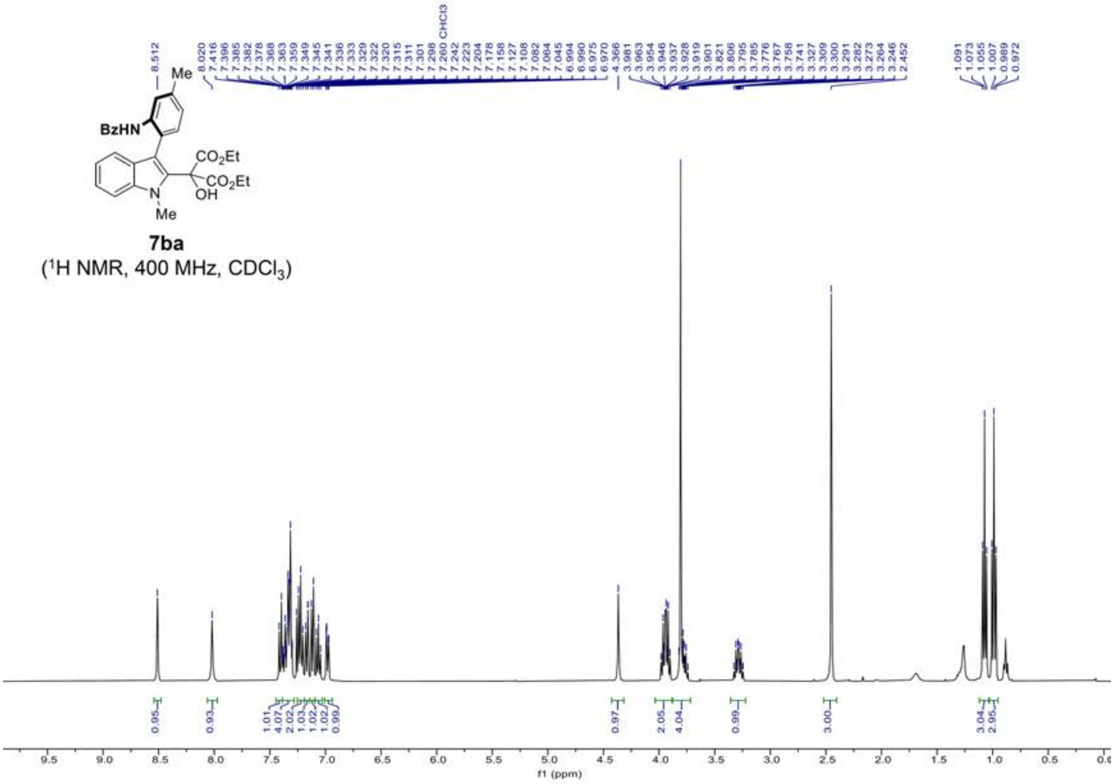
7ba
(1H NMR, 400 MHz, CDCl3)
f1 (ppm)

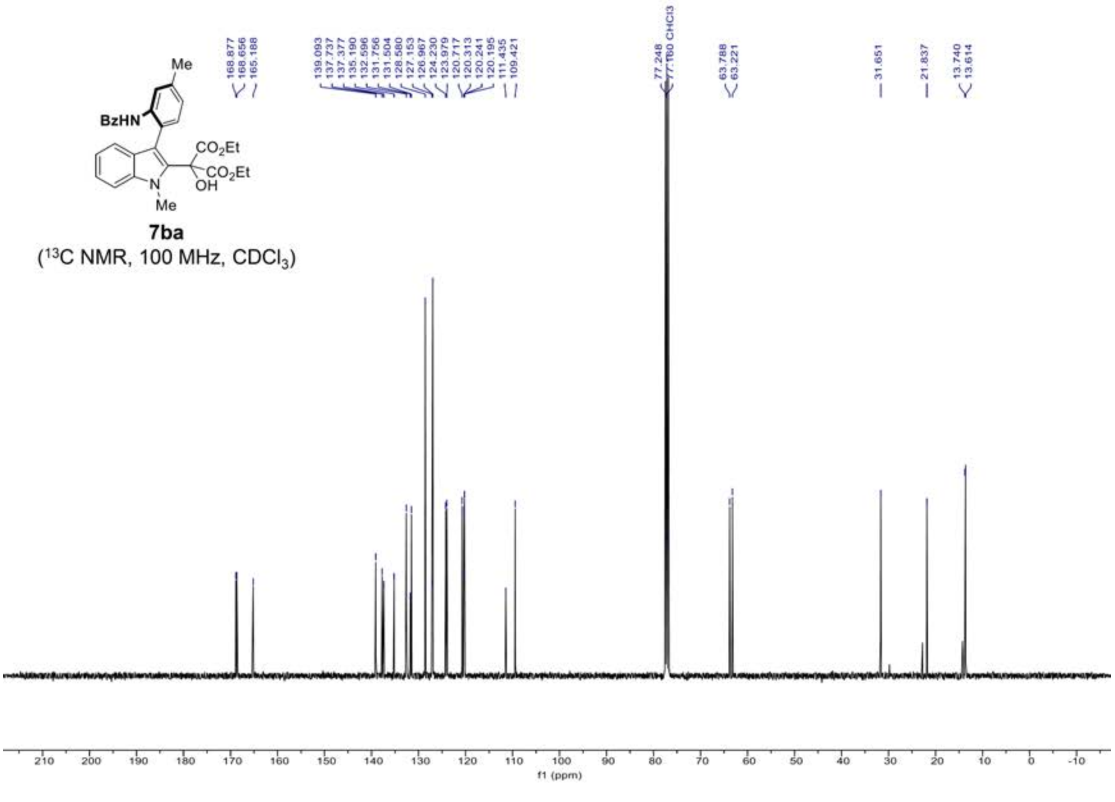
7ba
(13C NMR, 100 MHz, CDCl3)
f1 (ppm)

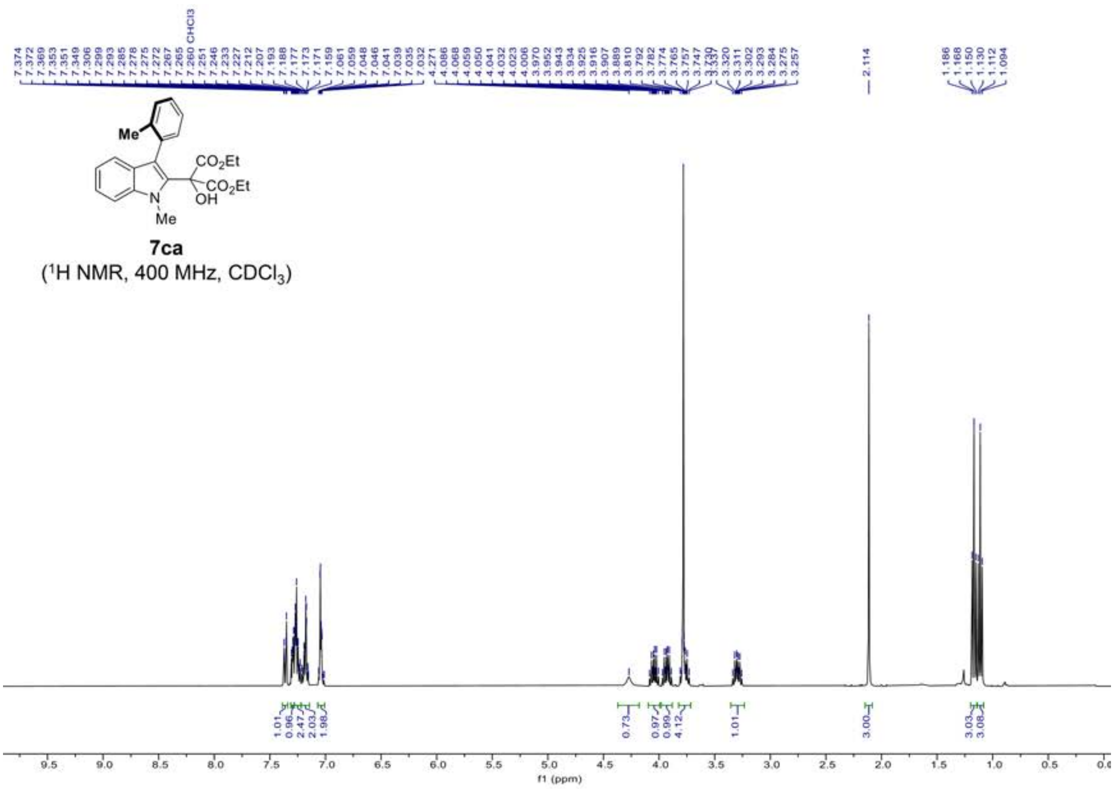
Me
CO2Et
CO2Et
OH
N
Me
7ca
(1H NMR, 400 MHz, CDCl3)
f1 (ppm)

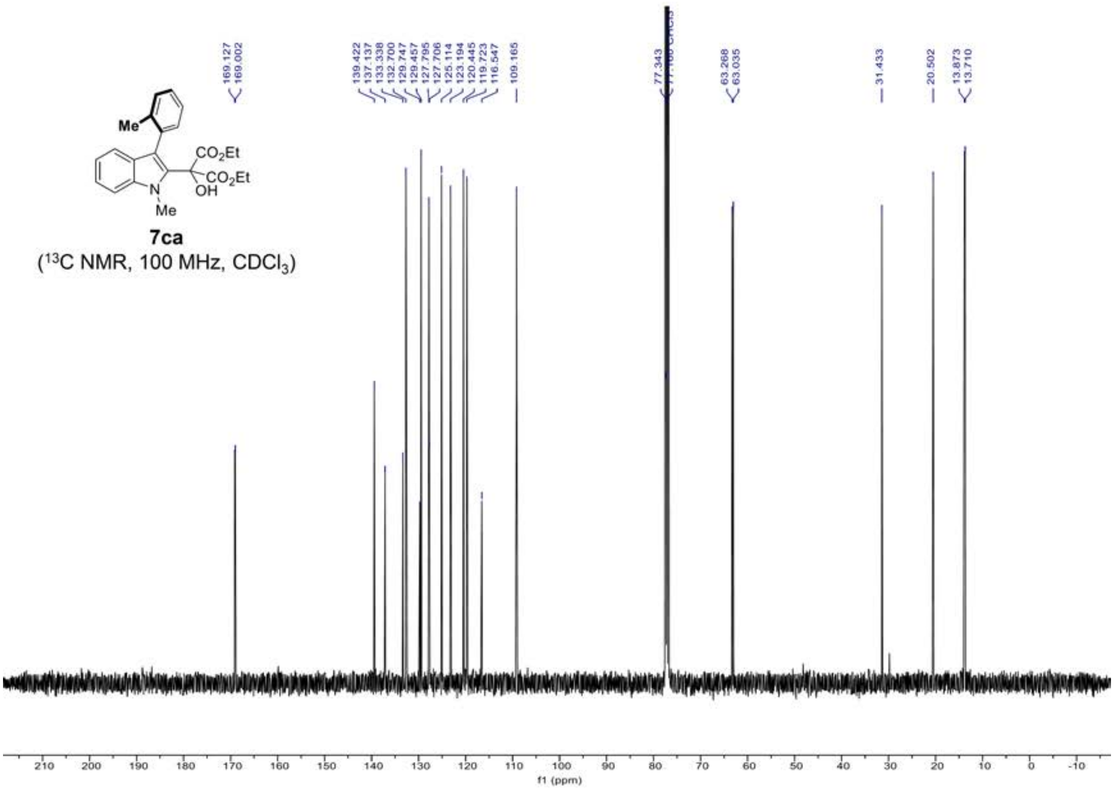
Me
CO2Et
CO2Et
OH
N
Me
7ca
(13C NMR, 100 MHz, CDCl3)
169.127
169.002
139.422
137.137
133.338
132.700
129.747
129.457
127.795
127.706
125.114
123.194
120.445
119.723
116.547
109.165
77.343
63.268
63.035
31.433
20.502
13.873
13.710
f1 (ppm)

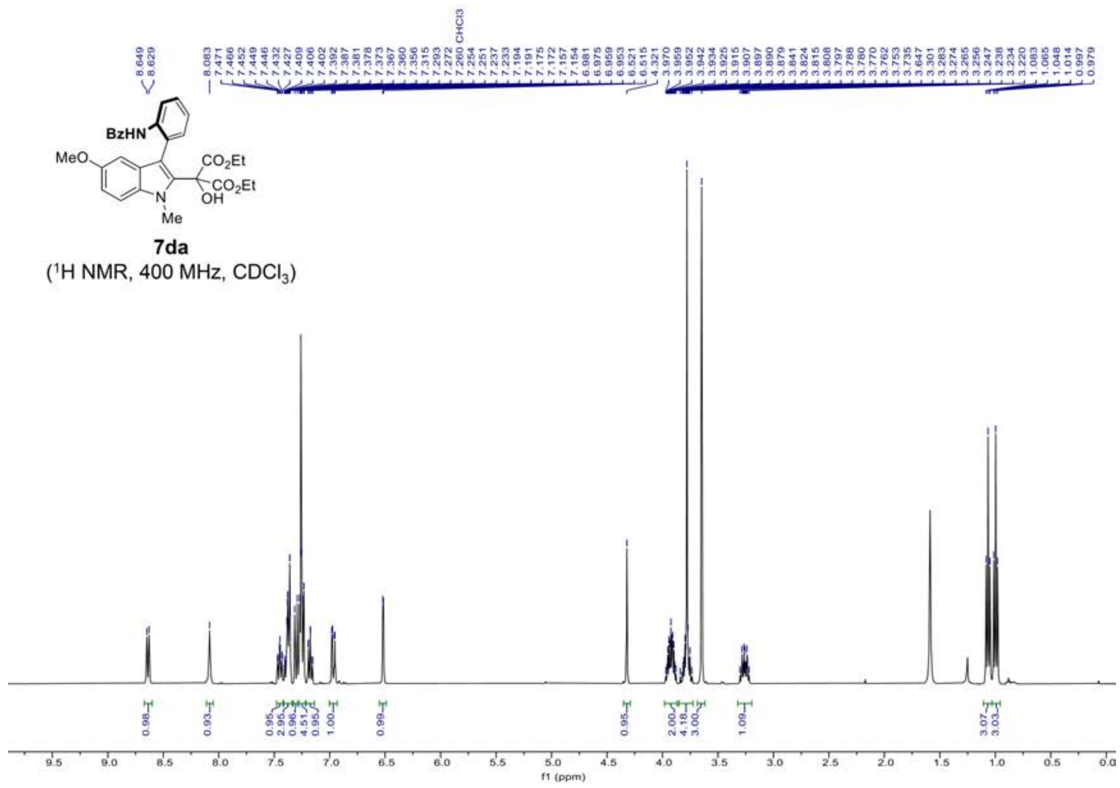

7da

(^{1}H NMR, 400 MHz, $CDCl_3$)

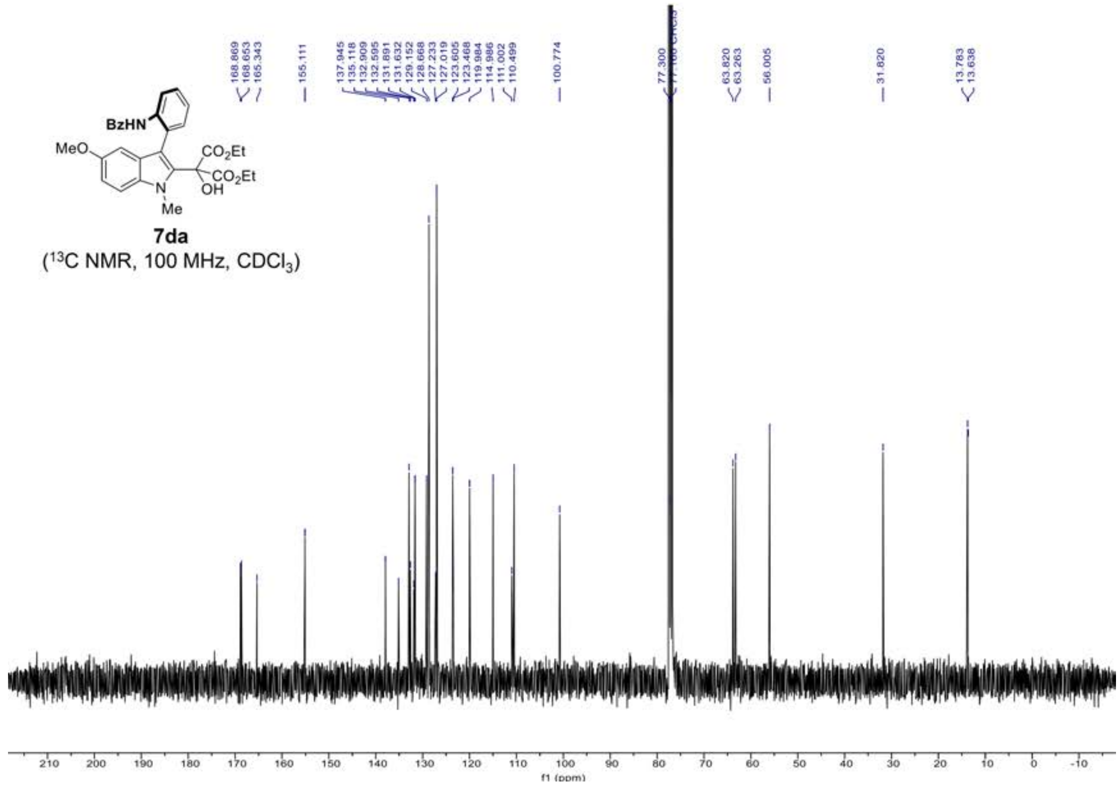

7da

(^{13}C NMR, 100 MHz, $CDCl_3$)

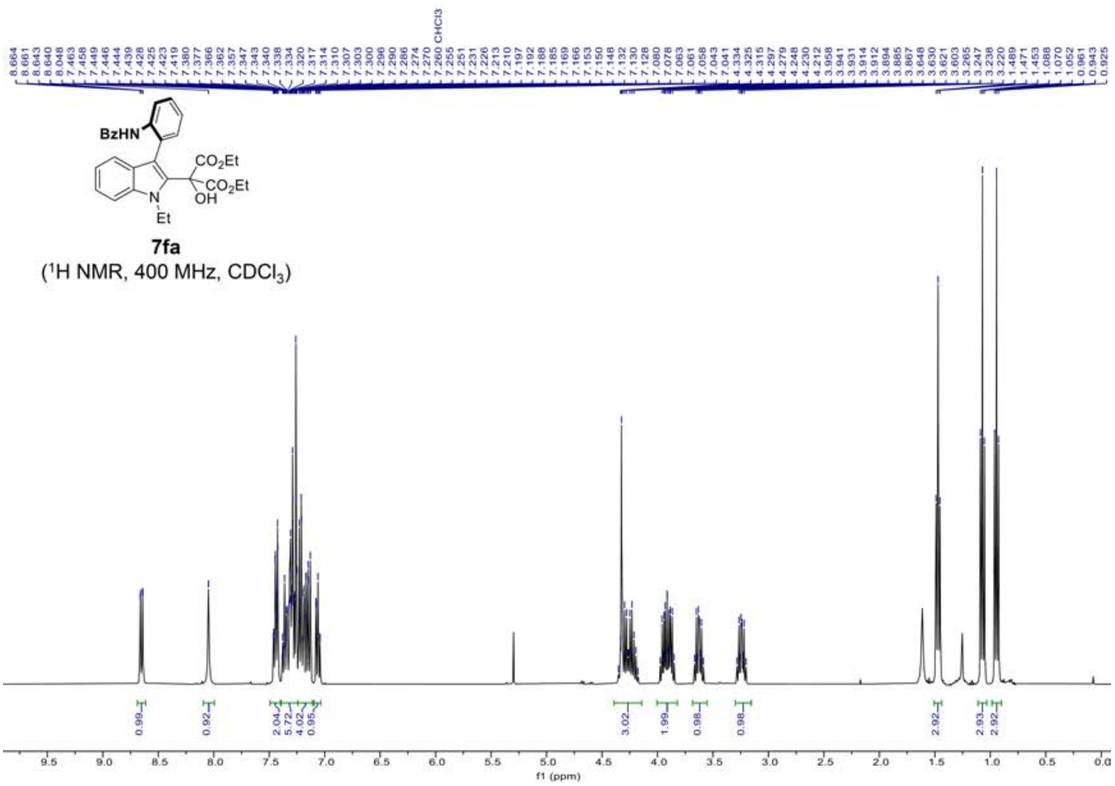
8.664 8.661 8.643 8.640 8.048 7.463 7.458 7.449 7.446 7.444 7.439 7.428 7.425 7.423 7.419 7.380 7.377 7.366 7.362 7.357 7.347 7.343 7.340 7.338 7.334 7.320 7.317 7.314 7.310 7.307 7.303 7.300 7.296 7.290 7.286 7.274 7.270 7.260 CHCl3 7.255 7.251 7.231 7.226 7.213 7.210 7.197 7.192 7.188 7.185 7.169 7.166 7.153 7.150 7.148 7.132 7.130 7.128 7.080 7.078 7.063 7.061 7.058 7.043 7.041 4.334 4.325 4.315 4.297 4.279 4.248 4.230 4.212 3.958 3.941 3.931 3.923 3.914 3.912 3.894 3.885 3.867 3.648 3.630 3.621 3.603 3.265 3.247 3.238 3.220 1.489 1.471 1.453 1.088 1.070 1.052 0.961 0.943 0.925
BzHN
CO2Et
CO2Et
OH
N
Et
7fa
(1H NMR, 400 MHz, CDCl3)
0.99 0.92 2.04 5.72 4.02 0.95 3.02 1.99 0.98 0.98 2.92 2.93 2.92
9.5 9.0 8.5 8.0 7.5 7.0 6.5 6.0 5.5 5.0 4.5 4.0 3.5 3.0 2.5 2.0 1.5 1.0 0.5 0.0
f1 (ppm)

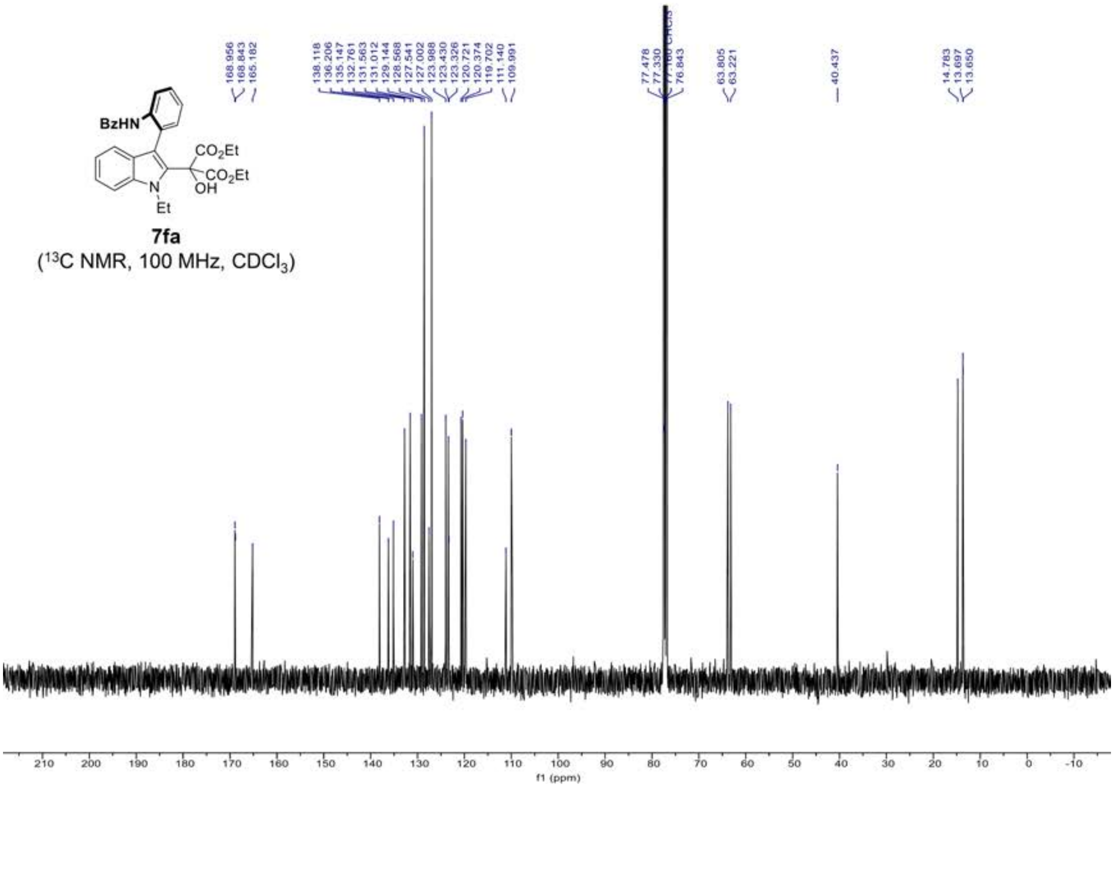
168.956 168.843 165.182
138.118 136.206 135.147 132.761 131.563 131.012 129.144 128.568 127.541 127.002 123.988 123.430 123.326 120.721 120.374 119.702 111.140 109.991
77.478 77.330 77.160 CHCl3 76.843
63.805 63.221
40.437
14.783 13.697 13.650
BzHN
CO2Et
CO2Et
OH
N
Et
7fa
(13C NMR, 100 MHz, CDCl3)
210 200 190 180 170 160 150 140 130 120 110 100 90 80 70 60 50 40 30 20 10 0 -10
f1 (ppm)

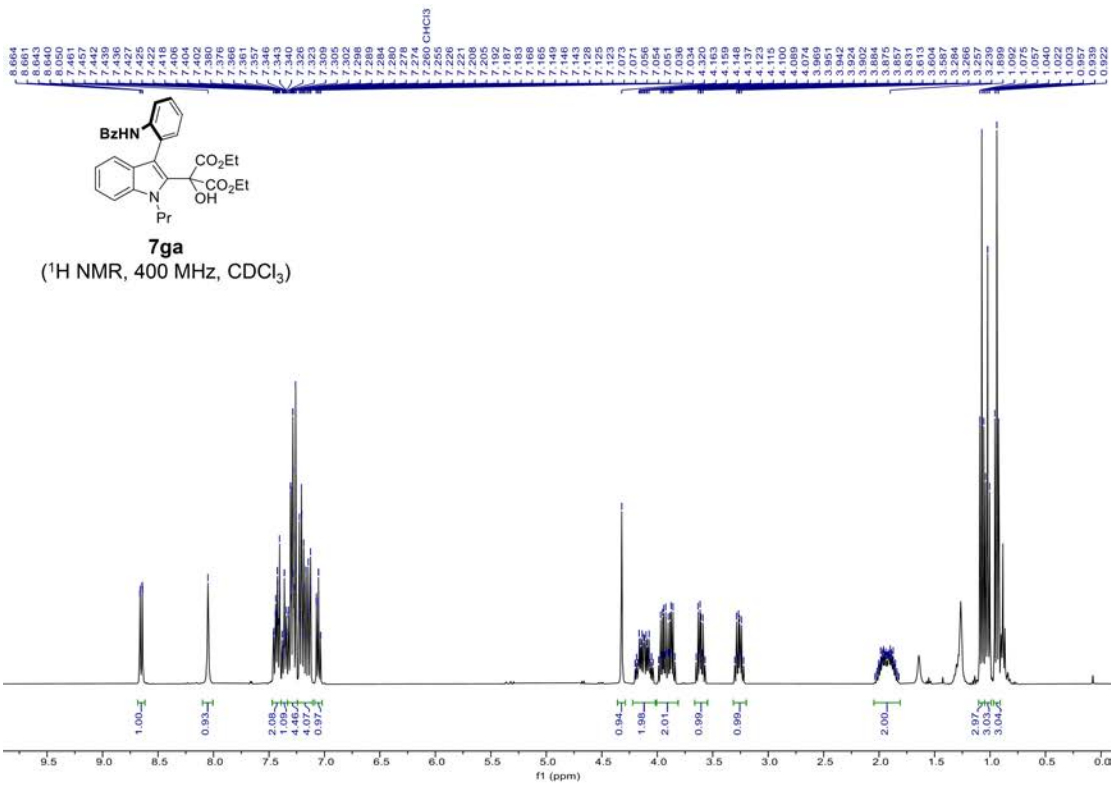
BzHN
CO2Et
CO2Et
OH
N
Pr
7ga
(1H NMR, 400 MHz, CDCl3)
f1 (ppm)

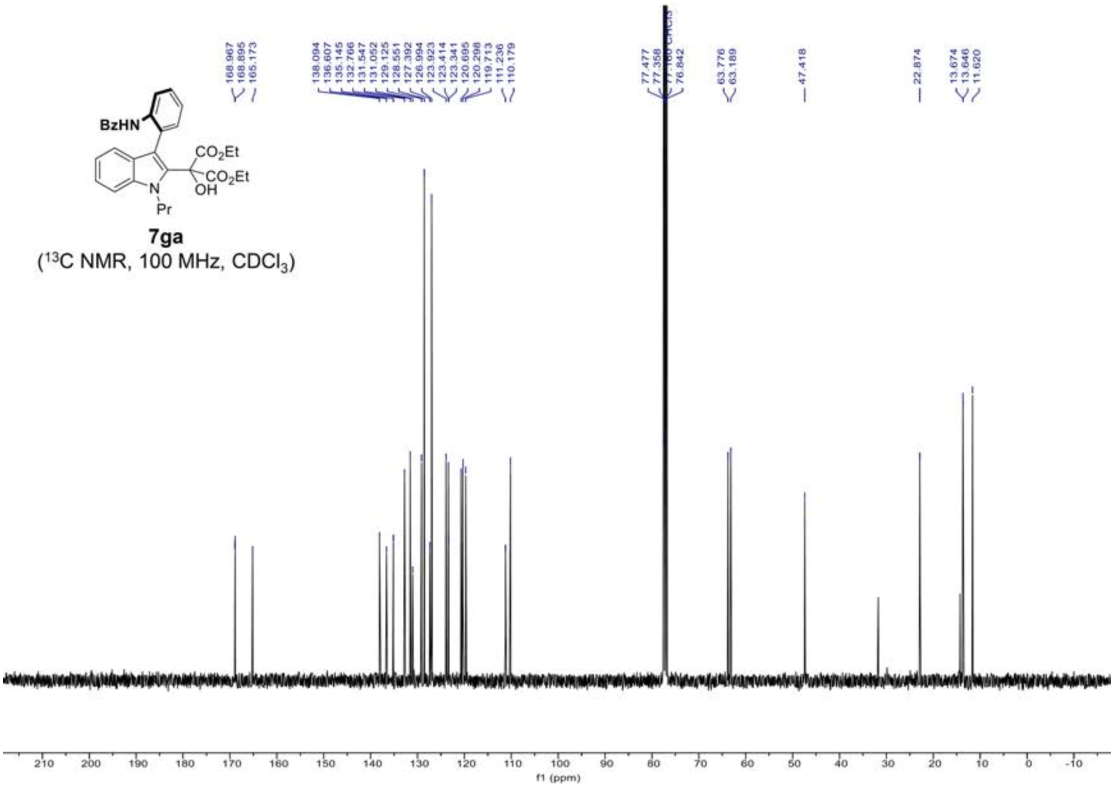
BzHN
CO2Et
CO2Et
OH
N
Pr
7ga
(13C NMR, 100 MHz, CDCl3)
f1 (ppm)

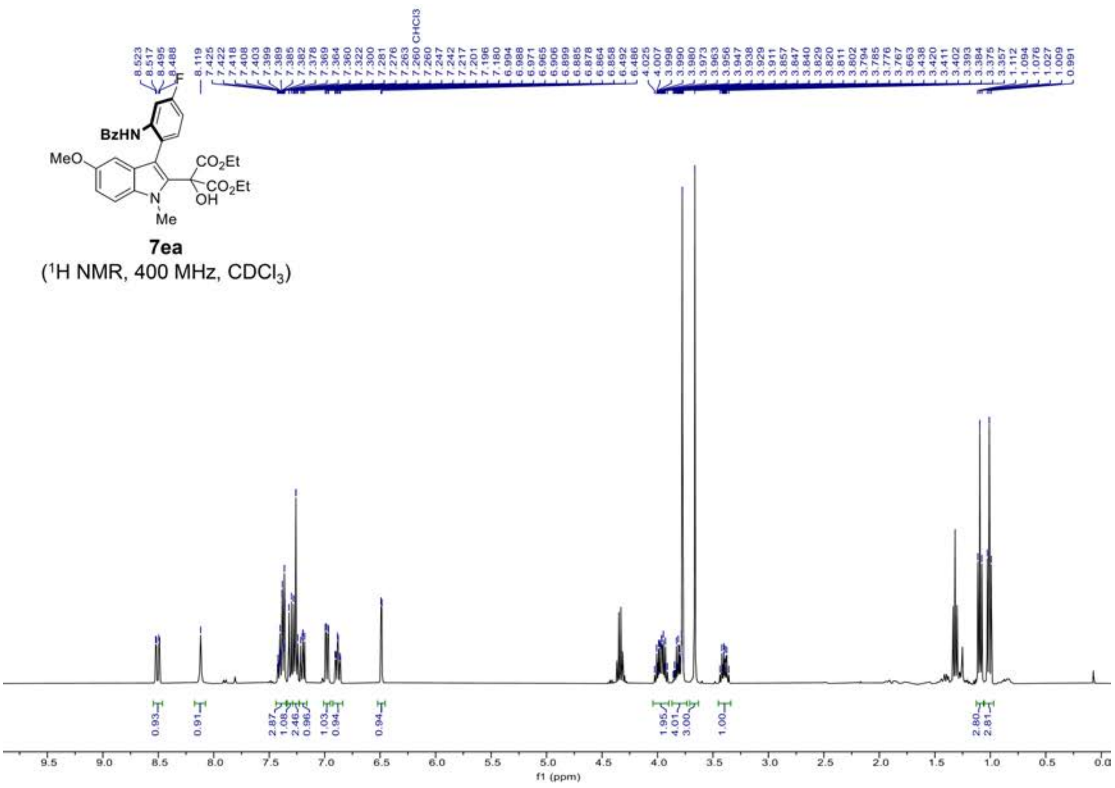

7ea

(^{1}H NMR, 400 MHz, $CDCl_3$)

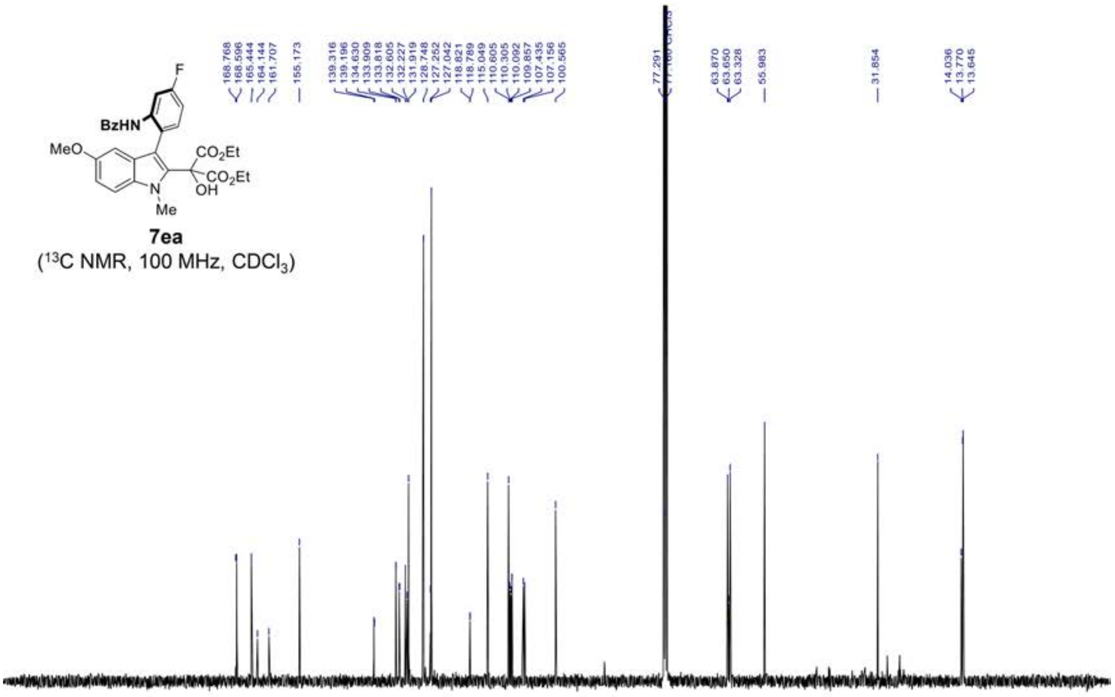

7ea

(^{13}C NMR, 100 MHz, $CDCl_3$)

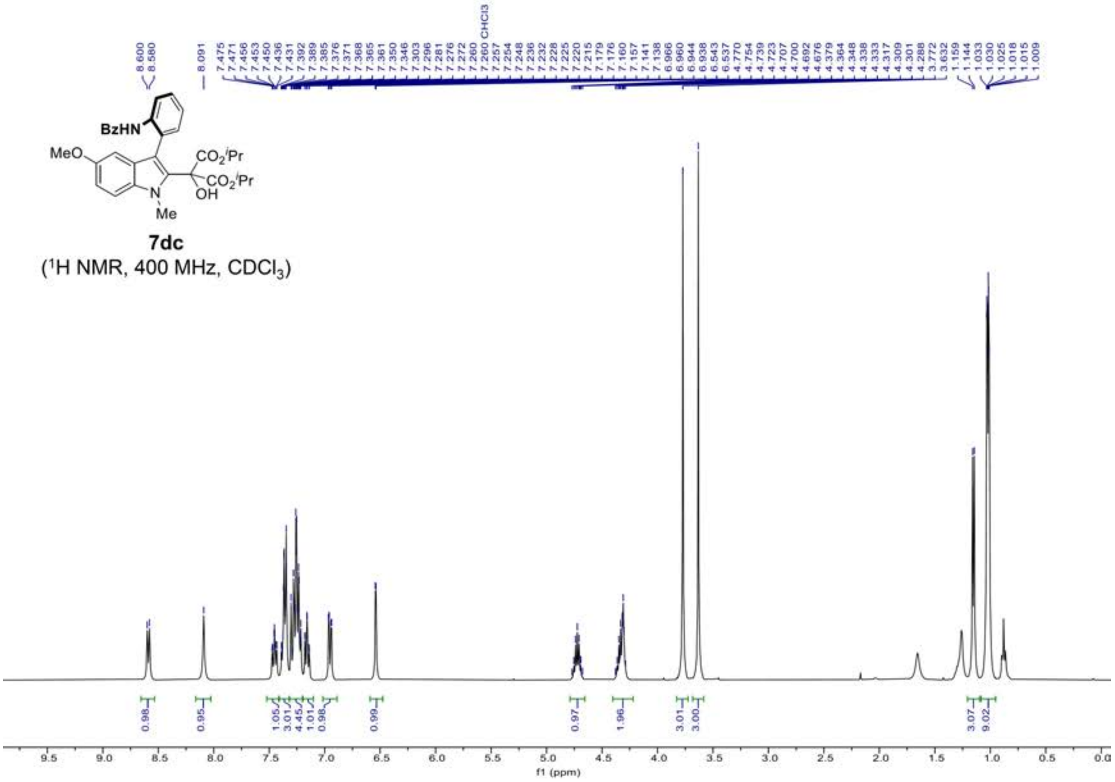

7dc

(^{1}H NMR, 400 MHz, $CDCl_3$)

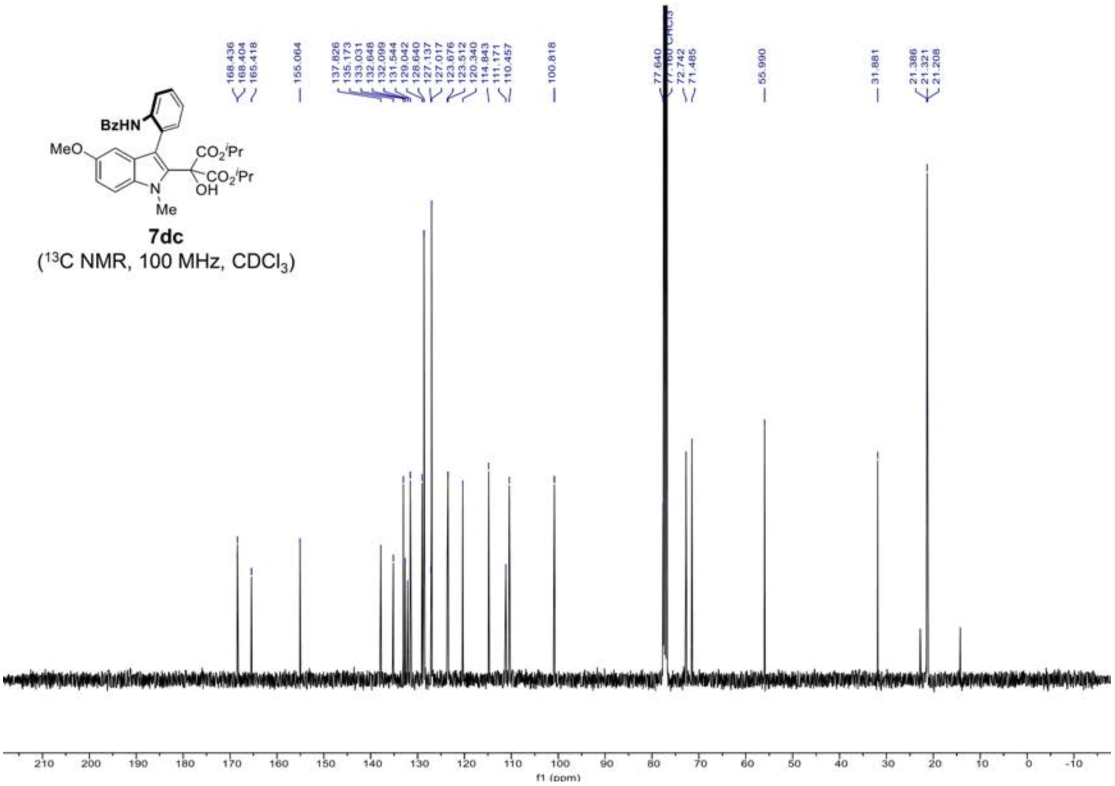

7dc

(^{13}C NMR, 100 MHz, $CDCl_3$)

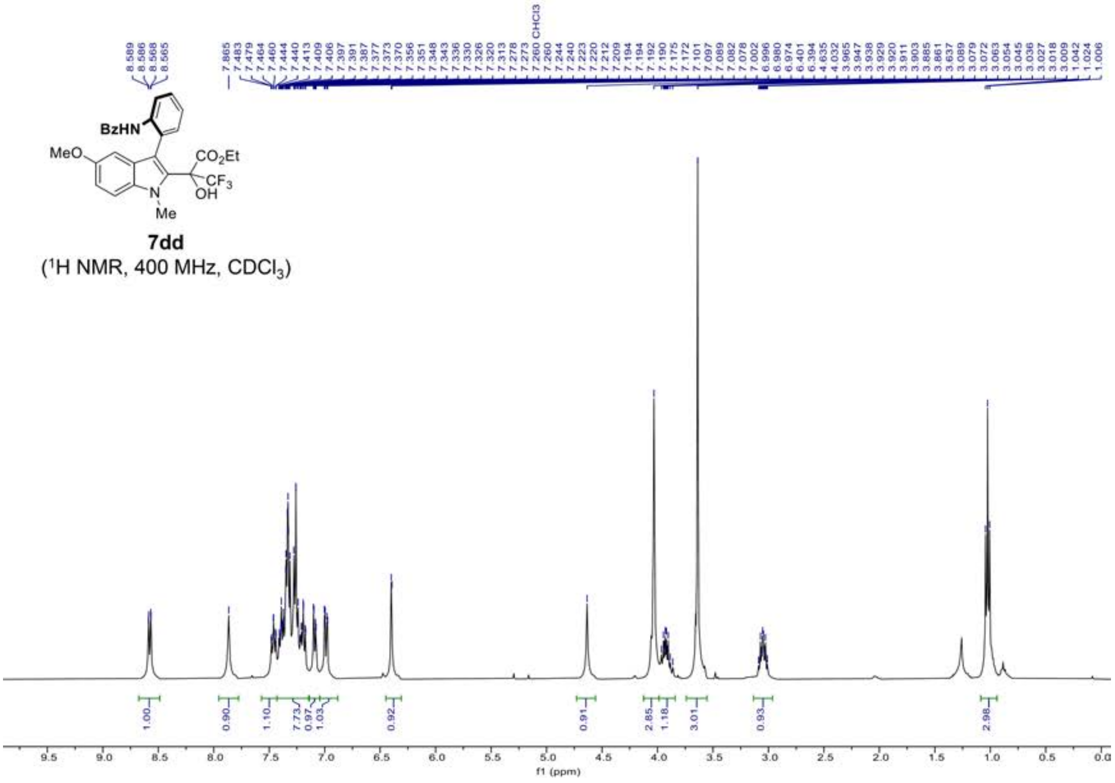

7dd
(^{1}H NMR, 400 MHz, $CDCl_3$)

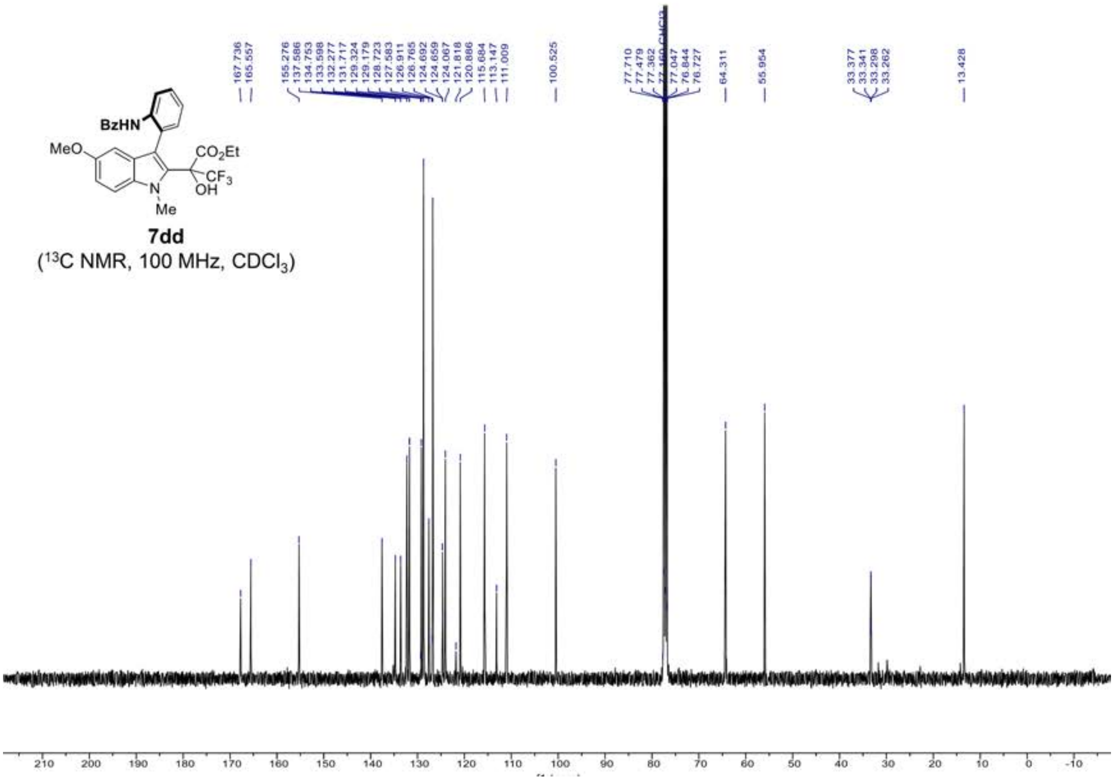

7dd
(^{13}C NMR, 100 MHz, $CDCl_3$)

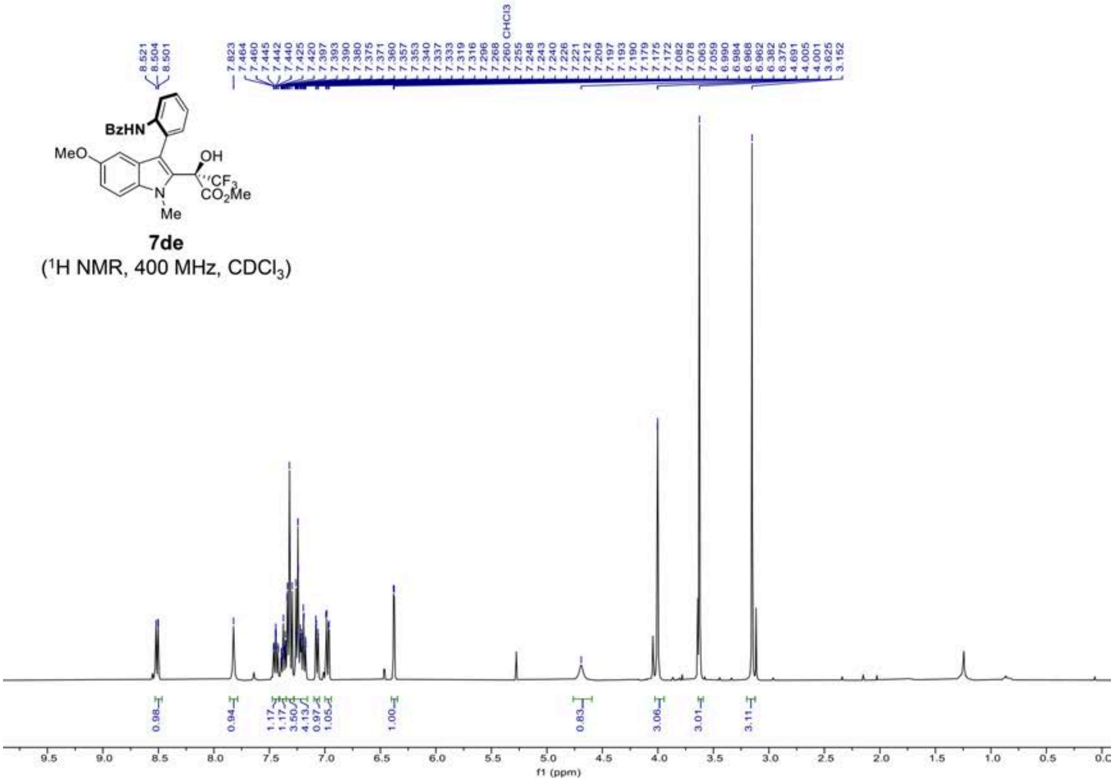
BzHN
MeO
OH
CF3
CO2Me
N
Me
7de
(1H NMR, 400 MHz, CDCl3)
f1 (ppm)

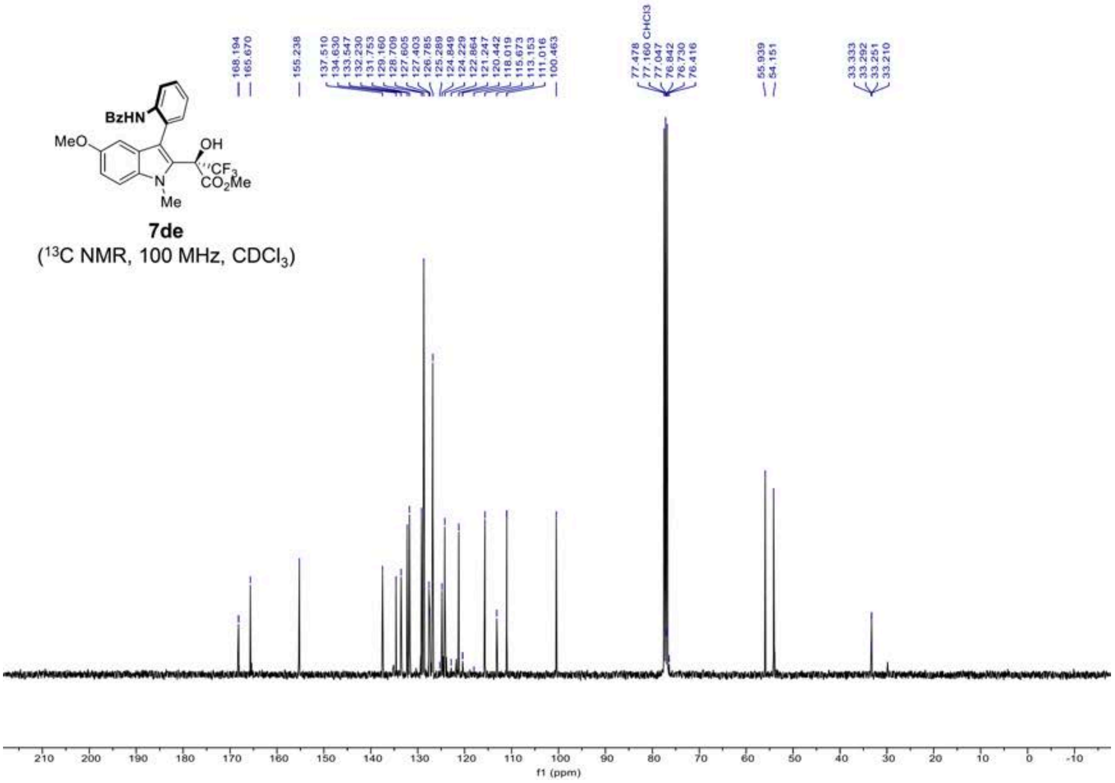
BzHN
MeO
OH
CF3
CO2Me
N
Me
7de
(13C NMR, 100 MHz, CDCl3)
f1 (ppm)

9.2 HPLC Traces of 7

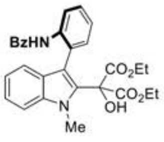

7aa

HPLC conditions:
Chiralpak IC, 30% ^{i}PrOH/n-Hexane eluent
1.0 mL/min, 254 nm

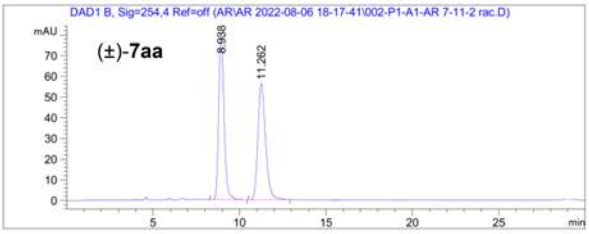

Peak #	RetTime [min]	Type	Width [min]	Area [mAU*s]	Height [mAU]	Area %
1	8.938	BB	0.3307	1727.07495	80.38293	49.9732
2	11.262	BB	0.4717	1728.92480	56.39359	50.0268

Totals : 3455.99976 136.77652

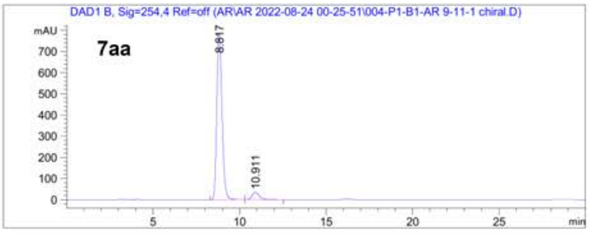

Peak #	RetTime [min]	Type	Width [min]	Area [mAU*s]	Height [mAU]	Area %
1	8.817	BB	0.3216	1.64491e4	787.66241	94.2909
2	10.911	BB	0.4551	995.95758	33.47574	5.7091

Totals : 1.74451e4 821.13815

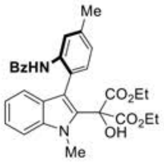

7ba

HPLC conditions:
Chiralpak IC, 10% ^{i}PrOH/n-Hexane eluent
1.0 mL/min, 254 nm

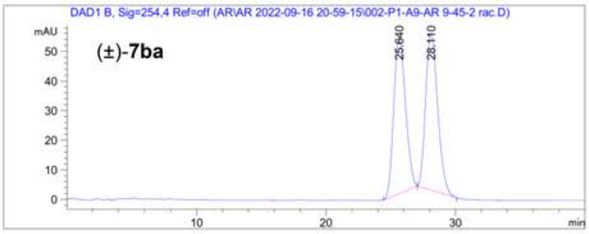

Peak #	RetTime [min]	Type	Width [min]	Area [mAU*s]	Height [mAU]	Area %
1	25.640	BB	0.9817	3326.65869	52.75542	49.2708
2	28.110	BB	1.0039	3425.13037	52.86456	50.7292
Totals :				6751.78906	105.61998	

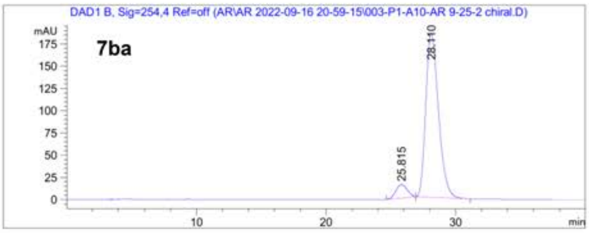

Peak #	RetTime [min]	Type	Width [min]	Area [mAU*s]	Height [mAU]	Area %
1	25.815	BB	0.8989	903.69391	15.04191	6.7239
2	28.110	BB	1.0409	1.25363e4	184.46826	93.2761
Totals :				1.34400e4	199.51017	

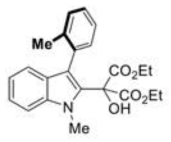

7ca

HPLC conditions:
Chiralpak IC, 5% ^{i}PrOH/*n*-Hexane eluent
1.0 mL/min, 254 nm

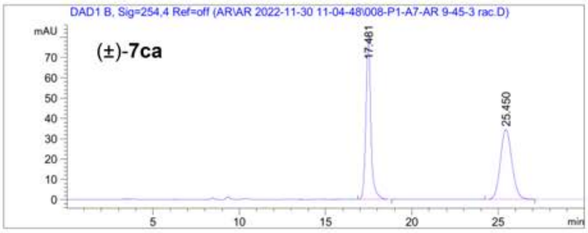

Peak #	RetTime [min]	Type	Width [min]	Area [mAU*s]	Height [mAU]	Area %
1	17.481	BB	0.2833	1579.70386	81.81332	49.8771
2	25.450	BB	0.7057	1587.48572	34.31441	50.1229
Totals :				3167.18958	116.12772	

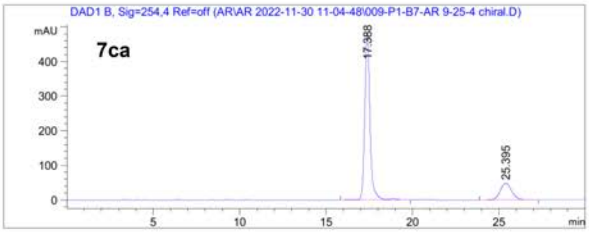

Peak #	RetTime [min]	Type	Width [min]	Area [mAU*s]	Height [mAU]	Area %
1	17.388	BV R	0.3415	1.01145e4	476.56073	82.1108
2	25.395	BB	0.7300	2203.60498	47.78347	17.8892
Totals :				1.23181e4	524.34420	

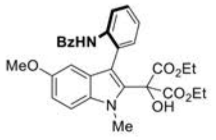

7da

HPLC conditions:
Chiralpak IC, 30% ^{i}PrOH/*n*-Hexane eluent
1.0 mL/min, 254 nm

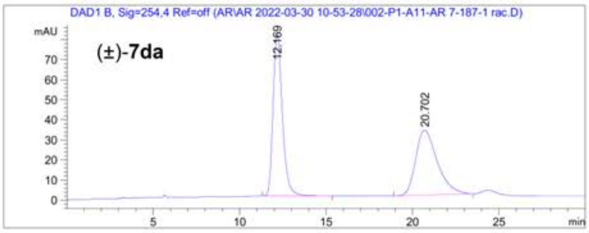

Peak #	RetTime [min]	Type	Width [min]	Area [mAU*s]	Height [mAU]	Area %
1	12.169	BB	0.5723	3021.67578	80.80828	51.7622
2	20.702	BB	1.2702	2815.93018	32.32957	48.2378
Totals :				5837.60596	113.13786	

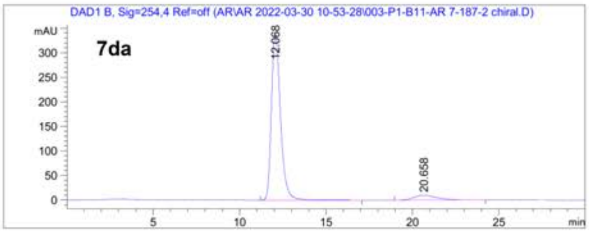

Peak #	RetTime [min]	Type	Width [min]	Area [mAU*s]	Height [mAU]	Area %
1	12.068	BB	0.5559	1.23578e4	340.13541	93.1442
2	20.658	BB	1.2328	909.58484	10.02220	6.8558
Totals :				1.32674e4	350.15760	

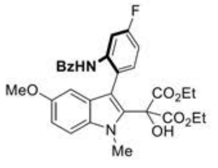

7ea

HPLC conditions:
Chiralpak AD, 50% ^{i}PrOH/n-Hexane eluent
1.0 mL/min, 254 nm

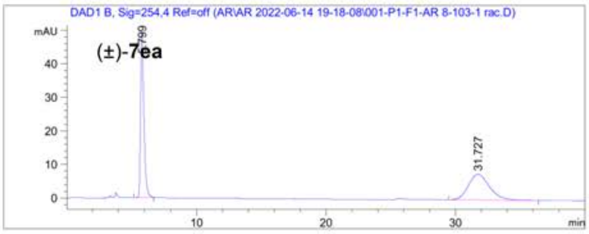

Peak #	RetTime [min]	Type	Width [min]	Area [mAU*s]	Height [mAU]	Area %
1	5.799	BB	0.2744	874.00476	48.45517	50.3776
2	31.727	BBA	1.7376	860.90375	7.64628	49.6224

Totals : 1734.90851 56.10144

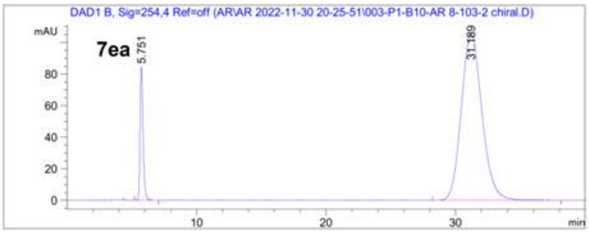

Peak #	RetTime [min]	Type	Width [min]	Area [mAU*s]	Height [mAU]	Area %
1	5.751	BB	0.2516	1357.39099	84.41911	10.4136
2	31.189	BB	1.7130	1.16774e4	105.69250	89.5864

Totals : 1.30348e4 190.11160

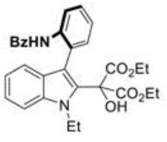

7fa

HPLC conditions:
Chiralpak IC, 30% ^{i}PrOH/n-Hexane eluent
1.0 mL/min, 254 nm

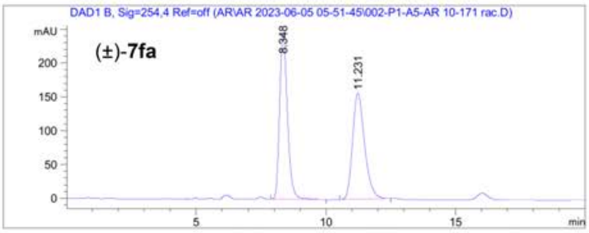

Peak #	RetTime [min]	Type	Width [min]	Area [mAU*s]	Height [mAU]	Area %
1	8.348	BB	0.3194	5112.18408	245.04631	50.2778
2	11.231	BB	0.4947	5055.68701	156.59436	49.7222
Totals :				1.01679e4	401.64067	

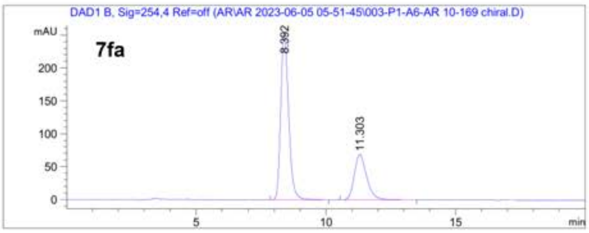

Peak #	RetTime [min]	Type	Width [min]	Area [mAU*s]	Height [mAU]	Area %
1	8.392	BB	0.3240	5334.75732	253.01669	69.9192
2	11.303	BB	0.5050	2295.12720	69.18166	30.0808
Totals :				7629.88452	322.19836	

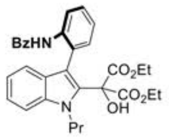

7ga

HPLC conditions:
Chiralpak IC, 30% ^{i}PrOH/*n*-Hexane eluent
1.0 mL/min, 254 nm

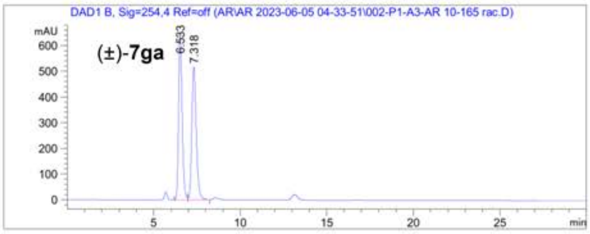

Peak #	RetTime [min]	Type	Width [min]	Area [mAU*s]	Height [mAU]	Area %
1	6.533	BV	0.2189	9275.78320	648.29730	50.2981
2	7.318	VB	0.2706	9165.82617	517.55005	49.7019
Totals :				1.84416e4	1165.84735	

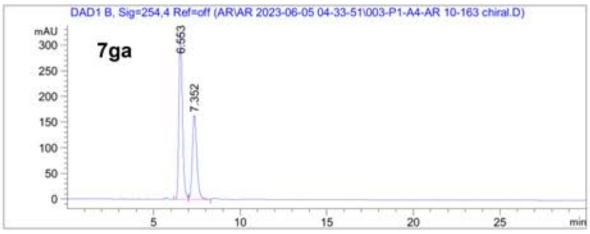

Peak #	RetTime [min]	Type	Width [min]	Area [mAU*s]	Height [mAU]	Area %
1	6.553	BV	0.2219	4650.38086	323.15753	61.3433
2	7.352	VB	0.2748	2930.52734	163.77950	38.6567
Totals :				7580.90820	486.93703	

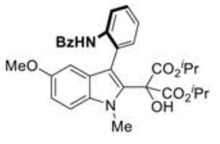

7dc

HPLC conditions:
Chiralpak IC, 20% ^{i}PrOH/n-Hexane eluent
1.0 mL/min, 254 nm

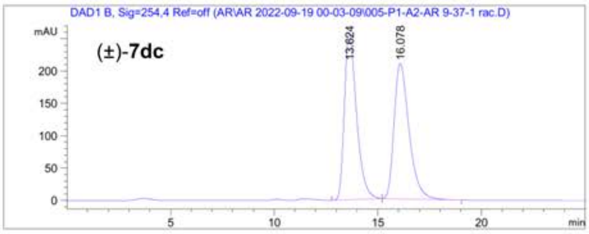

Peak #	RetTime [min]	Type	Width [min]	Area [mAU*s]	Height [mAU]	Area %
1	13.624	BB	0.6215	1.05092e4	256.77737	50.0674
2	16.078	BB	0.7636	1.04809e4	209.53806	49.9326
Totals :				2.09901e4	466.31543	

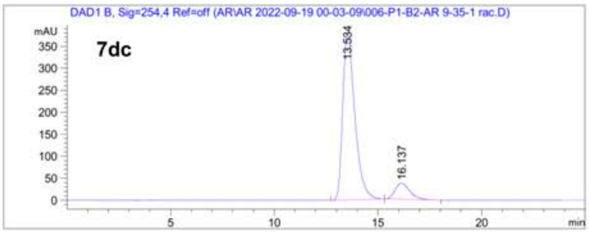

Peak #	RetTime [min]	Type	Width [min]	Area [mAU*s]	Height [mAU]	Area %
1	13.534	BB	0.6148	1.53187e4	377.95901	89.3491
2	16.137	BB	0.7558	1826.06958	36.87290	10.6509
Totals :				1.71447e4	414.83191	

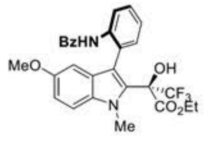

7dd

HPLC conditions:
Chiralpak AD-H, 30% ^{i}PrOH/n-Hexane eluent
1.0 mL/min, 254 nm

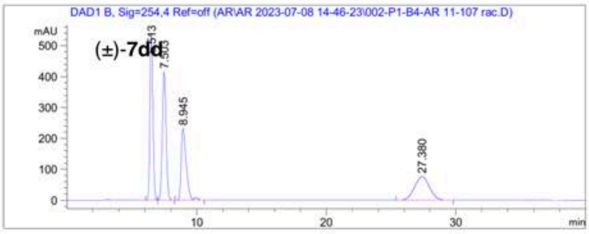

Peak #	RetTime [min]	Type	Width [min]	Area [mAU*s]	Height [mAU]	Area %
1	6.513	BB	0.2452	8465.54297	539.08398	28.7912
2	7.503	BB	0.3202	8461.58398	414.49445	28.7777
3	8.945	BV R	0.4062	6325.07764	231.07585	21.5115
4	27.380	BB	1.2097	6151.03369	76.36379	20.9196
Totals :				2.94032e4	1261.01807	

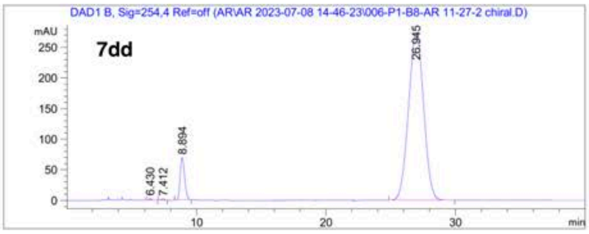

Peak #	RetTime [min]	Type	Width [min]	Area [mAU*s]	Height [mAU]	Area %
1	6.430	BB	0.2290	37.11243	2.53146	0.1552
2	7.412	BB	0.2771	33.55651	1.89059	0.1403
3	8.894	BB	0.3707	1668.34692	69.33955	6.9776
4	26.945	BB	1.2774	2.21709e4	272.45746	92.7268
Totals :				2.39099e4	346.21906	

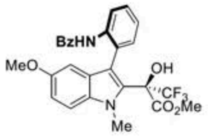

7de

HPLC conditions:
Chiralpak AD-H, 2% ^{i}PrOH/n-Hexane eluent
1.0 mL/min, 254 nm

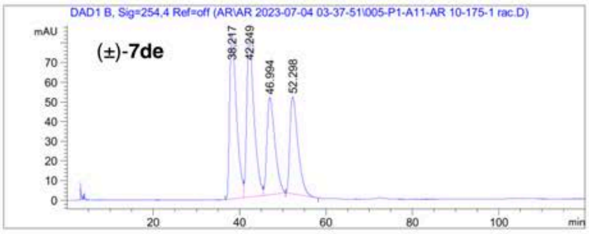

Peak #	RetTime [min]	Type	Width [min]	Area [mAU*s]	Height [mAU]	Area %
1	38.217	BV	1.6154	9285.78125	84.14955	29.3663
2	42.249	VV	1.7553	9550.36328	80.78009	30.2030
3	46.994	VB	1.9879	6345.33105	49.35508	20.0671
4	52.298	BB	2.0000	6439.08398	49.42263	20.3636

Totals : 3.16206e4 263.70735

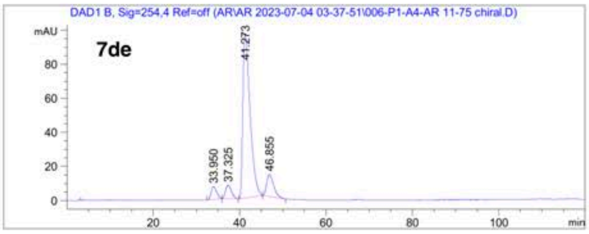

Peak #	RetTime [min]	Type	Width [min]	Area [mAU*s]	Height [mAU]	Area %
1	33.950	BB	1.0221	653.08459	7.57300	4.6693
2	37.325	BB	1.1540	755.31787	7.82589	5.4002
3	41.273	BB	1.6357	1.11457e4	96.62768	79.6867
4	46.855	BB	1.3708	1432.80200	12.87056	10.2439

Totals : 1.39869e4 124.89713

10 Chemical Modifications of 3aa

10.1 Reaction Procedures for Modifications of 3aa

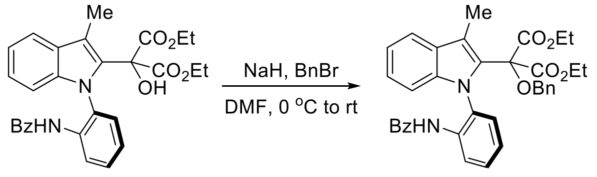

3aa **8**

Procedure 24: To a round-bottom flask equipped with a magnetic stirring bar, suspension of NaH (60% dispersion in mineral oil) (0.06 mmol, 1.2 equiv) in dry DMF (0.5 mL, 0.12 M) was cooled in an ice bath. Then a solution of **3aa** (0.05 mmol, 1 equiv) in dry DMF (0.5 mL, 0.1 M) was added slowly. After stirring the mixture for 15 min, benzyl bromide (0.055 mmol, 1.1 equiv) was added and the mixture was stirred at rt for 6 h. Then the reaction was quenched with saturated aqueous NH_4Cl and extracted with EtOAc, dried with anhydrous $MgSO_4$, and concentrated *in vacuo*. The crude material was then purified by flash chromatography to afford the desired material **8**.

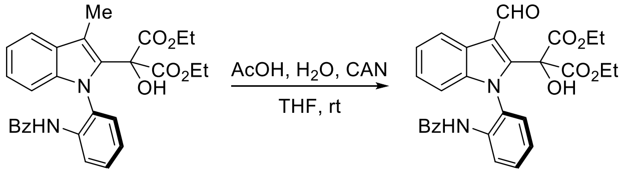

3aa **9**

Procedure 25: To a round-bottom flask equipped with a magnetic stirring bar, **3aa** (0.05 mmol, 1.0 equiv) was dissolved in THF (0.8 mL, 0.06 M), AcOH, and H2O. Then ceric ammonium nitrate was added to the mixture all in once. The reaction mixture was stirred at rt for 2 h. Then the mixture was poured into water and extracted with CH2Cl2 three times. The combined organic layers were then dried with anhydrous $MgSO_4$ and concentrated *in vacuo*. The crude material was then purified by flash chromatography to afford the desired material **9**.

10.2 Characterization and Spectra of 8 and 9

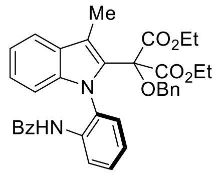

Diethyl 2-(1-(2-Benzamidophenyl)-3-methyl-1*H*-indol-2-yl)-2-(benzyloxy)malonate (8) was synthesized by following Procedure 24 from **3aa**. The crude material was purified by normal-phase column chromatography using an eluent of 20% EtOAc/Hx to give **8** (46% yield).

^{1}H NMR (400 MHz, $CDCl_3$) δ 8.36 (d, *J* = 7.7 Hz, 1H), 7.70 (s, 1H), 7.66–7.61 (m, 1H), 7.44 (ddd, *J* = 8.6, 5.3, 3.8 Hz, 1H), 7.41–7.35 (m, 1H), 7.30–7.21 (m, 4H), 7.21–7.08 (m, 9H), 6.73 (dt, *J* = 7.7, 0.9 Hz, 1H), 4.71 (d, *J* = 10.4 Hz, 1H), 4.64 (d, *J* = 10.4 Hz, 1H), 4.25 (dq, *J* = 10.8, 7.1 Hz, 1H), 4.19–3.99 (m, 3H), 2.37 (s, 3H), 1.24 (t, *J* = 7.1 Hz, 3H), 1.19 (t, *J* = 7.2 Hz, 3H).
^{13}C NMR (175 MHz, $CDCl_3$) δ 167.7, 167.2, 165.1, 138.6, 136.9, 136.8, 134.7, 131.6, 130.2, 130.1, 129.6, 128.9, 128.51 (2C), 128.49, 128.4 (2C), 128.3 (2C), 127.9, 127.1 (2C), 124.3, 124.1, 121.8, 120.8, 119.2, 114.4, 111.1, 83.4, 69.3, 62.8, 62.7, 14.01, 13.98, 9.8.
IR (FT-ATR, cm^{-1}, $CHCl_3$) ν_{max} 3950, 3933, 3925, 3905, 3874, 3855, 3840, 3822, 3802, 3752, 3736, 3728, 3712, 3702, 3692, 3677, 3630, 3622, 3596, 3569, 3553, 3531, 3504, 3489, 3423, 3061, 3033, 2980, 2929, 2872, 2384, 2369, 2359, 2338, 2329, 2295, 1737, 1682, 1593, 1562, 1523, 1496, 1455, 1360, 1307, 1243, 1157, 1135, 1095, 1028, 913, 892, 857, 795, 758, 738, 704, 668.
HRMS (EI) *m*/*z*: $[M]^+$ Calcd for $C_{36}H_{34}N_2O_6$ 590.2417; found 590.2418.
Optical: $[a]^{20}_{D}$ = +50.0° (*c* = 0.70, $CHCl_3$, 96% e.e.)
HPLC (Chiralpak AD-H, ^{i}PrOH/*n*-hexane = 5/95, flow rate = 1.0 mL/min, λ = 254 nm) t_R = 45.3 min (major), 30.4 min (minor)

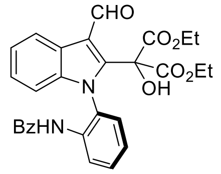

Diethyl 2-(1-(2-Benzamidophenyl)-3-formyl-1*H*-indol-2-yl)-2-hydroxymalonate (9) was synthesized by following Procedure 25 from **3aa**. The crude material was purified by normal-phase column chromatography using an eluent of 33% EtOAc/Hx to give **9** (60% yield).

^{1}H NMR (400 MHz, $CDCl_3$) δ 10.21 (s, 1H), 8.35 (dd, *J* = 8.3, 1.4 Hz, 1H), 8.08 (dt, *J* = 8.0, 1.0 Hz, 1H), 8.04 (s, 1H), 7.56 (ddd, *J* = 8.6, 7.5, 1.6 Hz, 1H), 7.41–7.31 (m, 4H), 7.31–7.13 (m, 5H), 6.79 (s, 1H), 6.75 (dt, *J* = 8.3, 0.9 Hz, 1H), 4.01 (ddq, *J* = 10.8, 10.0, 7.1 Hz, 2H), 3.92–3.76 (m, 2H), 1.12 (td, *J* = 7.2, 2.0 Hz, 6H).
^{13}C NMR (100 MHz, $CDCl_3$) δ 186.4, 168.6, 167.3, 166.0, 143.7, 138.1, 136.5, 134.0, 132.1, 131.3, 130.5, 128.7 (2C), 127.2 (2C), 126.43, 126.39, 125.6, 125.0, 124.5, 124.2, 119.4, 116.4, 112.0, 78.5, 63.9, 63.6, 13.83, 13.79.

IR (FT-ATR, cm^{-1}, $CHCl_3$) ν_{max} 3958, 3925, 3905, 3874, 3864, 3855, 3840, 3822, 3802, 3796, 3752, 3737, 3727, 3712, 3692, 3677, 3657, 3651, 3630, 3620, 3608, 3595, 3589, 3569, 3543, 3504, 3326, 3066, 2981, 2930, 2854, 2384, 2369, 2359, 2339, 2329, 2295, 1737, 1654, 1596, 1580, 1562, 1523, 1476, 1453, 1389, 1301, 1265, 1232, 1144, 1122, 1043, 955, 895, 858, 796, 754, 711, 689, 668.
HRMS (EI) *m*/*z*: $[M]^+$ Calcd for $C_{29}H_{26}N_2O_7$ 514.1740; found 514.1743.
Optical: $[a]^{20}_D$ = –12.8° (*c* = 1.45, $CHCl_3$, 96% e.e.)
HPLC (Chiralpak AD-H, ^{i}PrOH/*n*-hexane = 30/70, flow rate = 1.0 mL/min, λ = 254 nm) t_R = 17.0 min (major), 12.3 min (minor)

NMR Spectra of 8 and 9

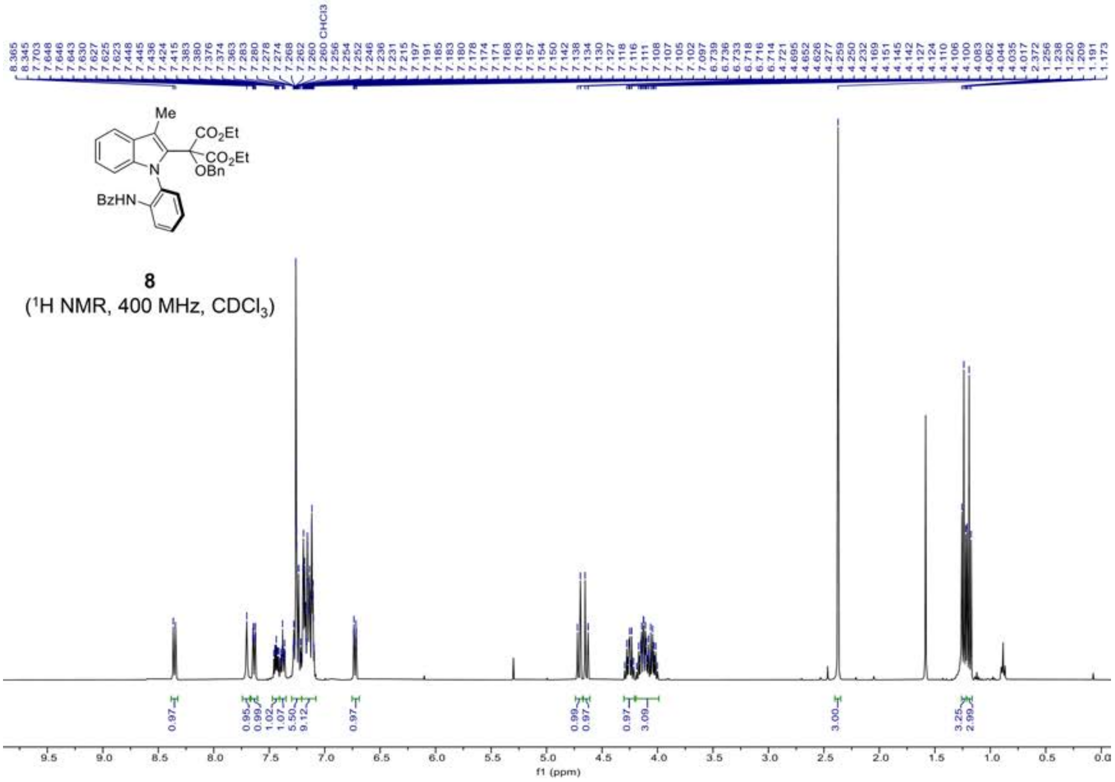

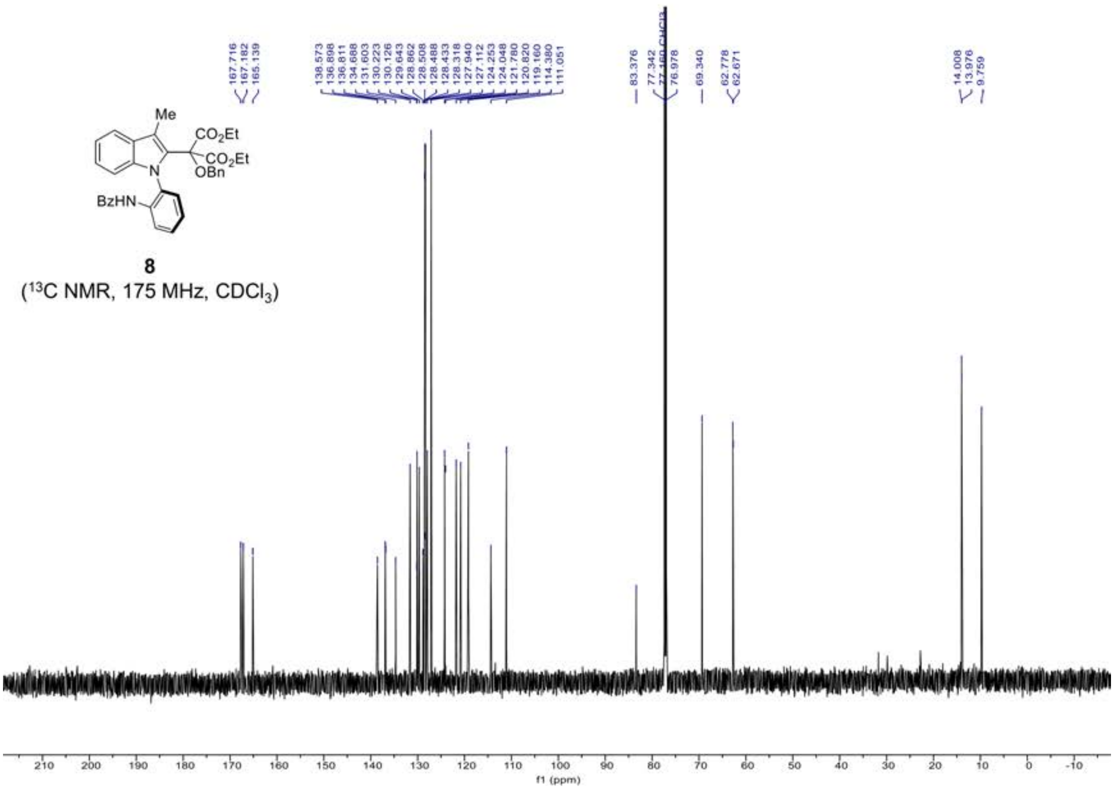

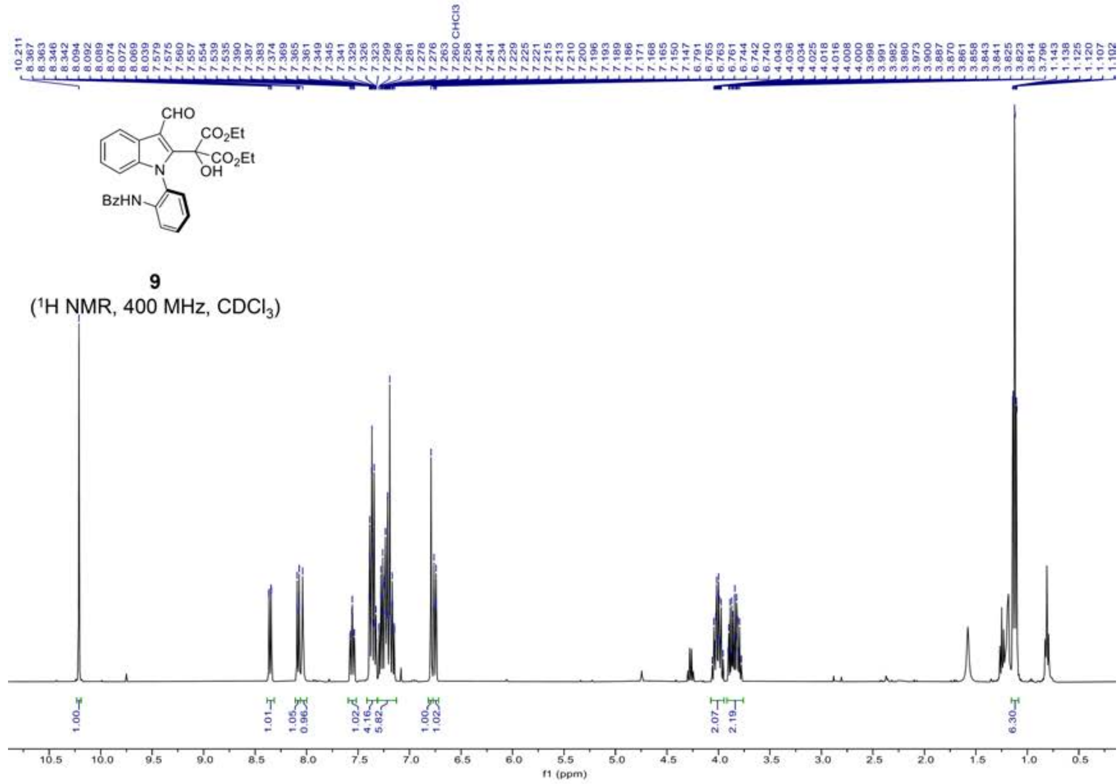
CHO
CO_2Et
CO_2Et
OH
N
BzHN
9
(^{1}H NMR, 400 MHz, $CDCl_3$)
f1 (ppm)

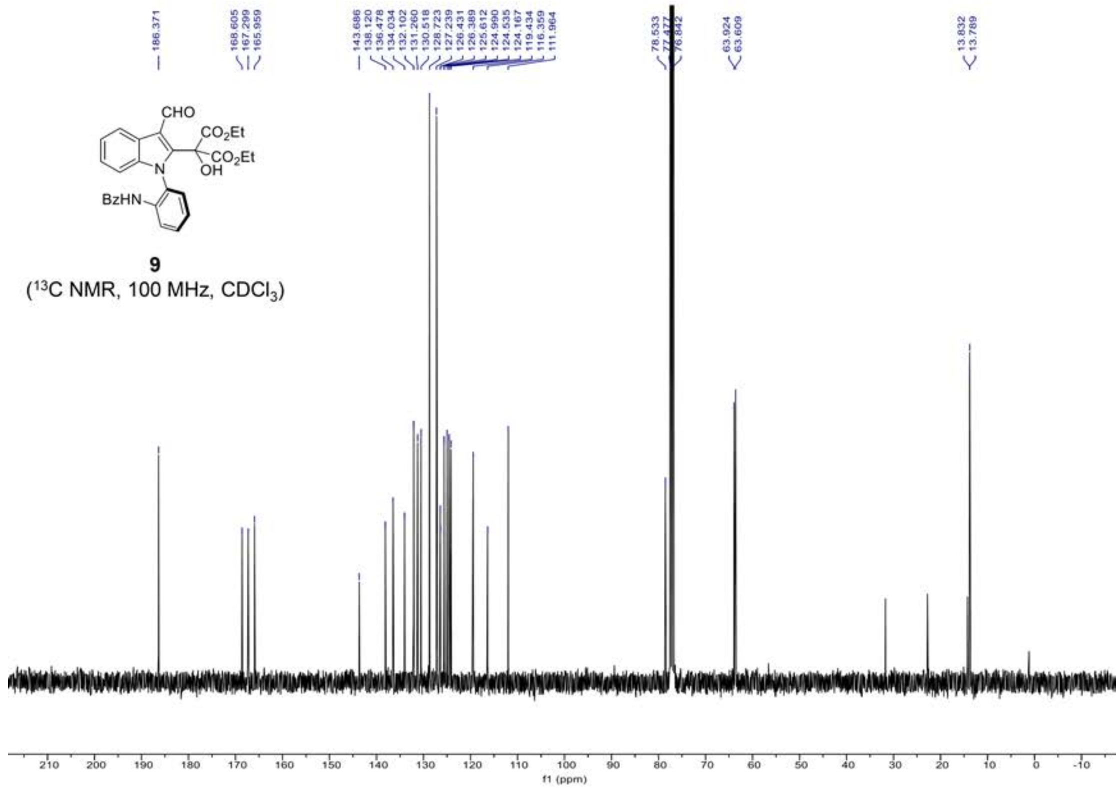
CHO
CO_2Et
CO_2Et
OH
N
BzHN
9
(^{13}C NMR, 100 MHz, $CDCl_3$)
186.371
168.605
167.299
165.959
143.686
138.120
136.478
134.034
132.102
131.260
130.518
128.723
127.239
126.431
126.389
125.612
124.990
124.535
124.167
119.434
116.359
111.964
78.533
77.477
76.842
63.924
63.609
13.832
13.789
f1 (ppm)

HPLC Traces of 8 and 9

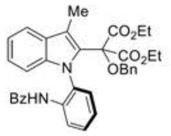

8

HPLC conditions:
Chiralpak AD-H, 5% ^{i}PrOH/*n*-Hexane eluent
1.0 mL/min, 254 nm

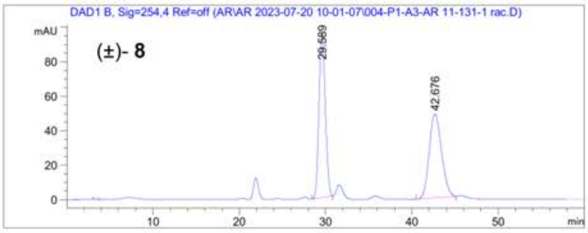

Peak #	RetTime [min]	Type	Width [min]	Area [mAU*s]	Height [mAU]	Area %
1	29.589	BB	0.8063	4908.57324	95.39689	50.4207
2	42.676	BB	1.3820	4826.66504	48.62609	49.5793
Totals :				9735.23828	144.02298	

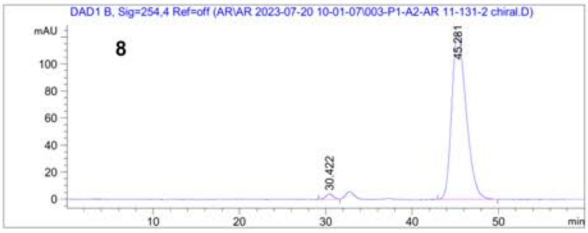

Peak #	RetTime [min]	Type	Width [min]	Area [mAU*s]	Height [mAU]	Area %
1	30.422	BB	0.7194	242.73383	4.03722	1.6663
2	45.281	BB	1.6604	1.43245e4	123.38906	98.3337
Totals :				1.45672e4	127.42628	

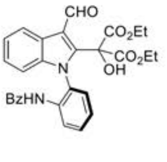

9

HPLC conditions:
Chiralpak AD-H, 30% ^{i}PrOH/n-Hexane eluent
1.0 mL/min, 254 nm

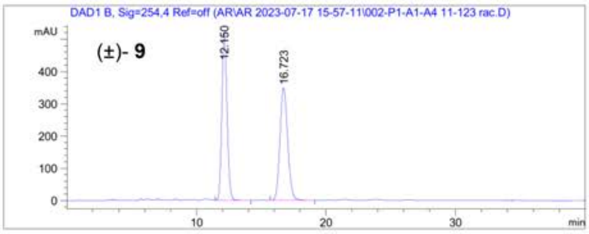

Peak #	RetTime [min]	Type	Width [min]	Area [mAU*s]	Height [mAU]	Area %
1	12.150	BB	0.4342	1.44420e4	516.57892	49.9581
2	16.723	BB	0.6442	1.44662e4	348.56467	50.0419

Totals : 2.89081e4 865.14359

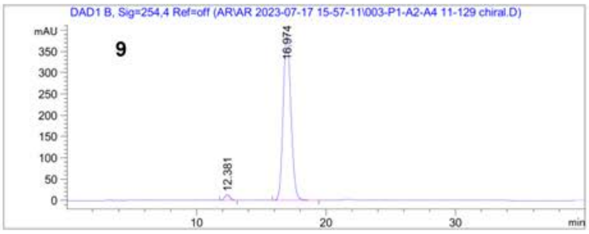

Peak #	RetTime [min]	Type	Width [min]	Area [mAU*s]	Height [mAU]	Area %
1	12.381	BB	0.4368	373.48151	13.33413	2.2301
2	16.974	BB	0.6493	1.63740e4	392.04129	97.7699

Totals : 1.67475e4 405.37542

11 Experiment for X-ray Crystallography

11.1 X-ray Crystallography of 3ba

Experimental details

A colorless rod-like specimen of $C_{30}H_{30}N_2O_6$, approximate dimensions 0.050 mm x 0.090 mm x 0.200 mm, was used for the X-ray crystallographic analysis. The X-ray intensity data were measured (λ = 0.71073 Å).

Supplementary Table 10. Data collection details for 3ba.

Axis	dx/mm	2θ/°	ω/°	φ/°	χ/°	Width/°	Frames	Time/s	Wavelength/Å	Voltage/kV	Current/mA	Temperature/K
Phi	60.627	0.00	0.00	0.00	54.74	1.00	180	1.20	0.71073	50	30.0	n/a
Phi	60.627	0.00	0.00	180.00	54.74	1.00	180	1.20	0.71073	50	30.0	n/a
Omega	60.627	18.54	-174.46	153.00	54.74	2.00	103	10.00	0.71073	50	30.0	n/a
Omega	60.627	27.81	-165.19	0.00	54.74	2.00	103	10.00	0.71073	50	30.0	n/a
Omega	60.627	18.54	-174.46	-105.00	54.74	2.00	103	10.00	0.71073	50	30.0	n/a
Omega	60.627	18.54	-174.46	51.00	54.74	2.00	103	10.00	0.71073	50	30.0	n/a
Omega	60.627	18.54	-174.46	-156.00	54.74	2.00	103	10.00	0.71073	50	30.0	n/a
Omega	60.627	18.54	-174.46	-54.00	54.74	2.00	103	10.00	0.71073	50	30.0	n/a
Omega	60.627	18.54	-174.46	102.00	54.74	2.00	103	10.00	0.71073	50	30.0	n/a
Phi	60.627	0.00	0.00	0.00	54.74	360.00	1	108.00	0.71073	50	30.0	n/a

A total of 1082 frames were collected. The total exposure time was 2.15 hours. The frames were integrated with the Bruker SAINT software package using a narrow-frame algorithm. The integration of the data using a monoclinic unit cell yielded a total of 50915 reflections to a maximum θ angle of 28.27° (0.75 Å resolution), of which 6737 were independent (average redundancy 7.558, completeness = 99.9%, R_{int} = 7.19%, R_{sig} = 4.92%) and 4943 (73.37%) were greater than $2\sigma(F^2)$. The final cell constants of a = 10.4488(8) Å, b = 11.0140(8) Å, c = 11.8477(9) Å, β = 93.723(3)°, volume = 1360.59(18) Å^3, are based upon the refinement of the XYZ-centroids of 9985 reflections above 20 σ(I) with 5.038° < 2θ < 45.89°. Data were corrected for absorption effects using the Multi-Scan method (SADABS). The ratio of minimum to maximum apparent transmission was 0.943. The calculated minimum and maximum transmission coefficients (based on crystal size) are 0.9830 and 0.9960.

The structure was solved and refined using the Bruker SHELXTL Software Package, using the space group P 1 21 1, with Z = 2 for the formula unit, $C_{30}H_{30}N_2O_6$. The final anisotropic full-matrix least-squares refinement on F^2 with 348 variables converged at R1 = 5.56%, for the observed data and wR2 = 12.23% for all data. The goodness-of-fit was 1.041. The largest peak in the final difference electron density synthesis was 0.248 e^-/Å^3 and the largest hole was −0.189 e^-/Å^3 with an RMS deviation of 0.043 e^-/Å^3. On the basis of the final model, the calculated density was 1.256 g/cm^3 and F(000), 544 e^-. CCDC number 2219791 (**3ba**) contains the supplementary crystallographic data for this paper. These data can be obtained free of charge from The Cambridge Crystallographic Data Center via www.ccdc.cam.ac.uk/data_request/cif.

The X-ray crystallography measurement was performed using the XRD at the National Research Facilities and Equipment Center (NanoBioEnergy Materials Center) at Ewha Womans University.

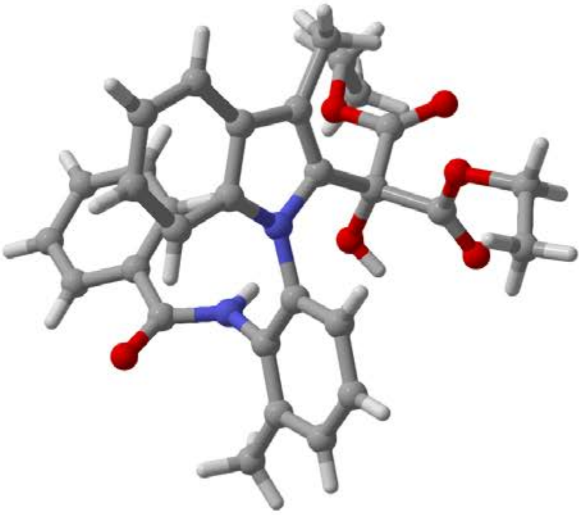

Supplementary Fig. 1 The single crystal X-ray structure of **3ba**; ellipsoid contour at the 50% probability level.

Supplementary Table 11. Sample and crystal data for 3ba.

**Chemical formula**	$C_{30}H_{30}N_2O_6$	
**Formula weight**	514.56 g/mol	
**Temperature**	296(2) K	
**Wavelength**	0.71073 Å	
**Crystal size**	0.050 x 0.090 x 0.200 mm	
**Crystal habit**	colorless rod	
**Crystal system**	monoclinic	
**Space group**	P 2_1	
**Unit cell dimensions**	a = 10.4488(8) Å	α = 90°
	b = 11.0140(8) Å	β = 93.723(3)°
	c = 11.8477(9) Å	γ = 90°
**Volume**	1360.59(18) $Å^3$	
**Z**	2	
**Density (calculated)**	1.256 g/cm^3	
**Absorption coefficient**	0.088 mm^{-1}	
**F(000)**	544	

Supplementary Table 12. Data collection and structure refinement for 3ba.

**Theta range for data collection**	1.72 to 28.27°
**Index ranges**	-13≤h≤13, -14≤k≤14, -15≤l≤15
**Reflections collected**	50915
**Independent reflections**	6737 [R(int) = 0.0719]
**Coverage of independent reflections**	99.9%
**Absorption correction**	Multi-Scan
**Max. and min. transmission**	0.9960 and 0.9830
**Structure solution technique**	direct methods

**Structure solution program**	SHELXT 2018/2 (Sheldrick, 2018)	
**Refinement method**	Full-matrix least-squares on F^2	
**Refinement program**	SHELXL-2018/3 (Sheldrick, 2018)	
**Function minimized**	$\Sigma\ w(F_o^2 - F_c^2)^2$	
**Data / restraints / parameters**	6737 / 1 / 348	
**Goodness-of-fit on F^2**	1.041	
**Final R indices**	4943 data; I>2σ(I)	R_1 = 0.0556, wR_2 = 0.1107
	all data	R_1 = 0.0838, wR_2 = 0.1223
**Weighting scheme**	$w=1/[\sigma^2(F_o^2)+(0.0491P)^2+0.2293P]$ where $P=(F_o^2+2F_c^2)/3$	
**Absolute structure parameter**	0.0(5)	
**Largest diff. peak and hole**	0.248 and -0.189 eÅ^{-3}	
**R.M.S. deviation from mean**	0.043 eÅ^{-3}	

Supplementary Table 13. Atomic coordinates and equivalent isotropic atomic displacement parameters (Å^2) for 3ba.

U(eq) is defined as one third of the trace of the orthogonalized U_{ij} tensor.

	x/a	y/b	z/c	U(eq)
C1	0.7842(3)	0.3581(3)	0.7208(3)	0.0371(7)
C2	0.7779(3)	0.2522(3)	0.6582(3)	0.0477(8)
C3	0.8069(4)	0.1456(3)	0.7149(4)	0.0603(11)
C4	0.8415(4)	0.1437(3)	0.8293(4)	0.0663(12)
C5	0.8491(4)	0.2489(4)	0.8916(4)	0.0608(10)
C6	0.8187(3)	0.3600(3)	0.8373(3)	0.0416(7)
C7	0.8155(3)	0.4834(3)	0.8742(3)	0.0423(7)
C8	0.7794(3)	0.5516(3)	0.7814(2)	0.0356(7)
C9	0.8515(4)	0.5235(4)	0.9931(3)	0.0663(11)
C10	0.7559(3)	0.6863(3)	0.7694(3)	0.0383(7)
C11	0.7214(4)	0.7462(3)	0.8819(3)	0.0491(8)
C12	0.5602(6)	0.7514(6)	0.0146(4)	0.0926(16)
C13	0.4719(7)	0.8460(9)	0.9778(7)	0.153(3)
C14	0.8713(3)	0.7550(3)	0.7287(3)	0.0434(7)
C15	0.0971(4)	0.7800(4)	0.7519(4)	0.0658(11)
C16	0.1431(4)	0.7399(5)	0.6436(4)	0.0726(12)
C17	0.7590(3)	0.5119(2)	0.5695(2)	0.0335(6)
C18	0.8748(3)	0.5287(3)	0.5217(3)	0.0435(7)
C19	0.8746(4)	0.5552(3)	0.4078(3)	0.0533(9)
C20	0.7607(4)	0.5650(3)	0.3438(3)	0.0557(10)
C21	0.6430(3)	0.5506(3)	0.3902(3)	0.0477(8)
C22	0.6438(3)	0.5236(3)	0.5050(2)	0.0356(6)
C23	0.5193(5)	0.5702(6)	0.3205(4)	0.0860(15)
C24	0.4397(3)	0.4309(3)	0.5560(3)	0.0450(8)
C25	0.3440(3)	0.4345(3)	0.6446(3)	0.0451(8)
C26	0.2283(3)	0.3740(4)	0.6240(4)	0.0574(10)
C27	0.1383(4)	0.3727(4)	0.7037(4)	0.0664(11)
C28	0.1621(4)	0.4313(5)	0.8050(4)	0.0709(12)
C29	0.2748(4)	0.4914(5)	0.8266(3)	0.0696(12)

C30	0.3669(4)	0.4941(4)	0.7471(3)	0.0570(10)
N1	0.7593(2)	0.4770(2)	0.6862(2)	0.0346(5)
N2	0.5292(2)	0.5181(3)	0.5635(2)	0.0434(6)
O1	0.6470(2)	0.70614(19)	0.69423(18)	0.0444(5)
O2	0.7784(3)	0.8290(3)	0.9252(2)	0.0758(9)
O3	0.6166(3)	0.6959(3)	0.9181(2)	0.0653(7)
O4	0.8598(3)	0.8376(2)	0.6625(2)	0.0613(7)
O5	0.9801(2)	0.7154(2)	0.7784(2)	0.0561(7)
O6	0.4376(3)	0.3521(3)	0.4829(2)	0.0726(9)

Supplementary Table 14. Bond lengths (Å) for 3ba.

C1-C2	1.382(5)	C1-N1	1.392(4)
C1-C6	1.403(5)	C2-C3	1.376(5)
C2-H2	0.93	C3-C4	1.380(6)
C3-H3	0.93	C4-C5	1.373(6)
C4-H4	0.93	C5-C6	1.409(5)
C5-H5	0.93	C6-C7	1.429(5)
C7-C8	1.365(4)	C7-C9	1.501(5)
C8-N1	1.400(4)	C8-C10	1.509(4)
C9-H9A	0.96	C9-H9B	0.96
C9-H9C	0.96	C10-O1	1.416(4)
C10-C14	1.528(4)	C10-C11	1.550(4)
C11-O2	1.187(4)	C11-O3	1.323(4)
C12-C13	1.440(9)	C12-O3	1.454(5)
C12-H12A	0.97	C12-H12B	0.97
C13-H13A	0.96	C13-H13B	0.96
C13-H13C	0.96	C14-O4	1.202(4)
C14-O5	1.320(4)	C15-O5	1.466(4)
C15-C16	1.468(6)	C15-H15A	0.97
C15-H15B	0.97	C16-H16A	0.96
C16-H16B	0.96	C16-H16C	0.96
C17-C18	1.381(4)	C17-C22	1.390(4)
C17-N1	1.434(4)	C18-C19	1.380(5)
C18-H18	0.93	C19-C20	1.374(5)
C19-H19	0.93	C20-C21	1.388(5)
C20-H20	0.93	C21-C22	1.391(4)
C21-C23	1.503(5)	C22-N2	1.423(4)
C23-H23A	0.96	C23-H23B	0.96
C23-H23C	0.96	C24-O6	1.225(4)
C24-N2	1.339(4)	C24-C25	1.497(5)
C25-C30	1.387(5)	C25-C26	1.388(5)
C26-C27	1.375(6)	C26-H26	0.93
C27-C28	1.371(6)	C27-H27	0.93
C28-C29	1.361(6)	C28-H28	0.93
C29-C30	1.390(6)	C29-H29	0.93
C30-H30	0.93	N2-H2A	0.86
O1-H1	0.82		

Supplementary Table 15. Bond angles (°) for 3ba.

C2-C1-N1	129.4(3)	C2-C1-C6	122.7(3)
N1-C1-C6	107.9(3)	C3-C2-C1	117.1(3)
C3-C2-H2	121.4	C1-C2-H2	121.4
C2-C3-C4	121.9(4)	C2-C3-H3	119.1
C4-C3-H3	119.1	C5-C4-C3	121.2(3)
C5-C4-H4	119.4	C3-C4-H4	119.4
C4-C5-C6	118.9(4)	C4-C5-H5	120.6
C6-C5-H5	120.6	C1-C6-C5	118.2(3)
C1-C6-C7	107.8(3)	C5-C6-C7	134.0(3)
C8-C7-C6	106.7(3)	C8-C7-C9	129.3(3)
C6-C7-C9	123.9(3)	C7-C8-N1	110.2(3)
C7-C8-C10	130.6(3)	N1-C8-C10	119.1(2)
C7-C9-H9A	109.5	C7-C9-H9B	109.5
H9A-C9-H9B	109.5	C7-C9-H9C	109.5
H9A-C9-H9C	109.5	H9B-C9-H9C	109.5
O1-C10-C8	109.3(2)	O1-C10-C14	110.1(2)
C8-C10-C14	112.9(3)	O1-C10-C11	104.7(3)
C8-C10-C11	112.6(3)	C14-C10-C11	107.0(3)
O2-C11-O3	125.7(3)	O2-C11-C10	124.1(3)
O3-C11-C10	110.1(3)	C13-C12-O3	110.5(5)
C13-C12-H12A	109.5	O3-C12-H12A	109.5
C13-C12-H12B	109.5	O3-C12-H12B	109.5
H12A-C12-H12B	108.1	C12-C13-H13A	109.5
C12-C13-H13B	109.5	H13A-C13-H13B	109.5
C12-C13-H13C	109.5	H13A-C13-H13C	109.5
H13B-C13-H13C	109.5	O4-C14-O5	126.0(3)
O4-C14-C10	122.2(3)	O5-C14-C10	111.7(3)
O5-C15-C16	111.4(3)	O5-C15-H15A	109.3
C16-C15-H15A	109.3	O5-C15-H15B	109.3
C16-C15-H15B	109.3	H15A-C15-H15B	108.0
C15-C16-H16A	109.5	C15-C16-H16B	109.5
H16A-C16-H16B	109.5	C15-C16-H16C	109.5
H16A-C16-H16C	109.5	H16B-C16-H16C	109.5
C18-C17-C22	120.8(3)	C18-C17-N1	119.0(3)
C22-C17-N1	120.2(3)	C19-C18-C17	119.0(3)
C19-C18-H18	120.5	C17-C18-H18	120.5
C20-C19-C18	120.2(3)	C20-C19-H19	119.9
C18-C19-H19	119.9	C19-C20-C21	122.0(3)
C19-C20-H20	119.0	C21-C20-H20	119.0
C20-C21-C22	117.6(3)	C20-C21-C23	121.2(3)
C22-C21-C23	121.2(3)	C17-C22-C21	120.5(3)
C17-C22-N2	117.0(3)	C21-C22-N2	122.3(3)
C21-C23-H23A	109.5	C21-C23-H23B	109.5
H23A-C23-H23B	109.5	C21-C23-H23C	109.5
H23A-C23-H23C	109.5	H23B-C23-H23C	109.5
O6-C24-N2	122.4(3)	O6-C24-C25	122.2(3)
N2-C24-C25	115.5(3)	C30-C25-C26	118.7(3)
C30-C25-C24	122.8(3)	C26-C25-C24	118.5(3)
C27-C26-C25	120.7(4)	C27-C26-H26	119.7

C25-C26-H26	119.7	C28-C27-C26	120.2(4)
C28-C27-H27	119.9	C26-C27-H27	119.9
C29-C28-C27	119.9(4)	C29-C28-H28	120.1
C27-C28-H28	120.1	C28-C29-C30	120.8(4)
C28-C29-H29	119.6	C30-C29-H29	119.6
C25-C30-C29	119.7(4)	C25-C30-H30	120.2
C29-C30-H30	120.2	C1-N1-C8	107.4(2)
C1-N1-C17	121.7(2)	C8-N1-C17	127.6(2)
C24-N2-C22	127.2(3)	C24-N2-H2A	116.4
C22-N2-H2A	116.4	C10-O1-H1	109.5
C11-O3-C12	117.7(3)	C14-O5-C15	116.7(3)

Supplementary Table 16. Anisotropic atomic displacement parameters (Å^2) for 3ba.

The anisotropic atomic displacement factor exponent takes the form: $-2\pi^2[h^2 a^{*2} U_{11} + ... + 2 h k a^* b^* U_{12}]$

	U_{11}	U_{22}	U_{33}	U_{23}	U_{13}	U_{12}
C1	0.0294(14)	0.0331(15)	0.0493(18)	0.0039(14)	0.0057(13)	-0.0017(13)
C2	0.0448(18)	0.0325(15)	0.066(2)	-0.0042(16)	0.0078(16)	0.0001(15)
C3	0.048(2)	0.0297(17)	0.104(3)	-0.0010(19)	0.013(2)	0.0025(15)
C4	0.062(3)	0.037(2)	0.099(4)	0.024(2)	0.004(2)	0.0066(18)
C5	0.061(2)	0.053(2)	0.068(2)	0.025(2)	0.0001(19)	0.0008(19)
C6	0.0373(16)	0.0372(17)	0.0505(19)	0.0112(15)	0.0051(14)	-0.0009(14)
C7	0.0455(18)	0.0440(18)	0.0370(17)	0.0038(15)	0.0008(14)	-0.0051(14)
C8	0.0421(17)	0.0319(15)	0.0327(15)	-0.0006(12)	0.0013(13)	-0.0047(13)
C9	0.089(3)	0.068(3)	0.0407(19)	0.0070(19)	-0.0084(19)	-0.005(2)
C10	0.0494(18)	0.0288(15)	0.0362(16)	-0.0007(13)	-0.0007(14)	-0.0014(13)
C11	0.063(2)	0.0413(18)	0.0430(19)	-0.0056(16)	0.0042(17)	0.0002(18)
C12	0.107(4)	0.112(4)	0.063(3)	-0.015(3)	0.037(3)	0.007(4)
C13	0.106(5)	0.198(8)	0.157(7)	-0.059(7)	0.016(4)	0.056(6)
C14	0.060(2)	0.0290(15)	0.0415(17)	-0.0033(14)	0.0077(15)	-0.0058(15)
C15	0.066(3)	0.068(3)	0.064(3)	0.006(2)	0.011(2)	-0.023(2)
C16	0.076(3)	0.068(3)	0.076(3)	-0.004(2)	0.019(2)	-0.011(2)
C17	0.0405(16)	0.0253(14)	0.0350(15)	-0.0030(12)	0.0050(12)	-0.0029(12)
C18	0.0388(17)	0.0400(17)	0.0523(19)	-0.0047(15)	0.0087(14)	-0.0003(14)
C19	0.054(2)	0.053(2)	0.057(2)	0.0003(18)	0.0265(18)	0.0004(17)
C20	0.076(3)	0.058(2)	0.0352(17)	-0.0014(17)	0.0135(18)	0.0058(19)

C21	0.054(2)	0.051(2)	0.0377(17)	-0.0033(16)	-0.0007(15)	0.0079(16)
C22	0.0375(16)	0.0346(15)	0.0351(15)	-0.0061(13)	0.0050(12)	0.0029(13)
C23	0.081(3)	0.126(4)	0.049(2)	0.006(3)	-0.014(2)	0.016(3)
C24	0.0349(16)	0.0509(19)	0.0484(19)	-0.0054(16)	-0.0022(14)	-0.0019(15)
C25	0.0348(17)	0.0452(18)	0.055(2)	0.0005(16)	-0.0005(15)	0.0006(14)
C26	0.0403(19)	0.057(2)	0.075(3)	-0.002(2)	0.0022(18)	-0.0079(17)
C27	0.038(2)	0.069(3)	0.093(3)	0.015(2)	0.007(2)	-0.0038(19)
C28	0.053(2)	0.088(3)	0.074(3)	0.021(3)	0.020(2)	0.005(2)
C29	0.066(3)	0.091(3)	0.053(2)	0.002(2)	0.0109(19)	-0.002(2)
C30	0.045(2)	0.075(3)	0.051(2)	0.0015(19)	0.0026(16)	-0.0105(18)
N1	0.0411(14)	0.0283(12)	0.0343(13)	0.0001(10)	0.0014(10)	-0.0016(10)
N2	0.0363(14)	0.0481(15)	0.0465(15)	-0.0127(12)	0.0079(11)	-0.0041(13)
O1	0.0550(13)	0.0318(11)	0.0456(13)	0.0007(10)	-0.0026(10)	0.0008(10)
O2	0.110(2)	0.0584(18)	0.0600(17)	-0.0268(14)	0.0128(16)	-0.0227(17)
O3	0.0679(17)	0.0745(18)	0.0557(15)	-0.0154(14)	0.0214(13)	-0.0083(15)
O4	0.0785(18)	0.0423(14)	0.0635(16)	0.0164(13)	0.0071(13)	-0.0060(12)
O5	0.0506(14)	0.0559(16)	0.0616(15)	0.0149(13)	0.0020(12)	-0.0142(12)
O6	0.0610(17)	0.0783(19)	0.0792(19)	-0.0403(17)	0.0103(14)	-0.0204(15)

Supplementary Table 17. Hydrogen atomic coordinates and isotropic atomic displacement parameters (Å^2) for 3ba.

	x/a	y/b	z/c	U(eq)
H2	0.7550	0.2529	0.5810	0.057
H3	0.8030	0.0729	0.6751	0.072
H4	0.8600	0.0699	0.8649	0.08
H5	0.8739	0.2468	0.9684	0.073
H9A	0.8793	0.6066	0.9927	0.099
H9B	0.9198	0.4734	1.0249	0.099
H9C	0.7785	0.5163	1.0378	0.099
H12A	0.5153	0.6900	1.0555	0.111
H12B	0.6275	0.7851	1.0654	0.111
H13A	0.5189	0.9143	0.9519	0.23
H13B	0.4231	0.8705	1.0398	0.23
H13C	0.4149	0.8163	0.9172	0.23
H15A	1.1633	0.7658	0.8117	0.079

H15B	1.0799	0.8665	0.7487	0.079
H16A	1.1583	0.6540	0.6460	0.109
H16B	1.2215	0.7814	0.6301	0.109
H16C	1.0796	0.7581	0.5837	0.109
H18	0.9516	0.5224	0.5655	0.052
H19	0.9518	0.5665	0.3744	0.064
H20	0.7624	0.5817	0.2669	0.067
H23A	0.5361	0.6136	0.2528	0.129
H23B	0.4615	0.6164	0.3634	0.129
H23C	0.4814	0.4930	0.3009	0.129
H26	0.2115	0.3339	0.5556	0.069
H27	0.0611	0.3320	0.6889	0.08
H28	0.1012	0.4299	0.8589	0.085
H29	0.2904	0.5312	0.8953	0.084
H30	0.4436	0.5356	0.7625	0.068
H2A	0.5160	0.5772	0.6086	0.052
H1	0.6645	0.7561	0.6463	0.067

11.2 X-ray Crystallography of 3ld

Experimental details

A colorless block-like specimen of $C_{28}H_{25}F_3N_2O_5$, approximate dimensions 0.050 mm x 0.050 mm x 0.120 mm, was used for the X-ray crystallographic analysis. The X-ray intensity data were measured (λ = 0.71073 Å).

Supplementary Table 18. Data collection details for 3ld.

Axis	dx/mm	2θ/°	ω/°	φ/°	χ/°	Width/°	Frames	Time/s	Wavelength/Å	Voltage/kV	Current/mA	Temperature/K
Omega	60.603	18.54	-174.46	-105.00	54.74	2.00	103	10.00	0.71073	50	30.0	n/a
Omega	60.603	18.54	-174.46	51.00	54.74	2.00	103	10.00	0.71073	50	30.0	n/a
Omega	60.603	18.54	-174.46	153.00	54.74	2.00	103	10.00	0.71073	50	30.0	n/a
Omega	60.603	18.54	-174.46	-54.00	54.74	2.00	103	10.00	0.71073	50	30.0	n/a
Omega	60.603	18.54	-174.46	102.00	54.74	2.00	103	10.00	0.71073	50	30.0	n/a
Omega	60.603	27.81	-165.19	0.00	54.74	2.00	103	10.00	0.71073	50	30.0	n/a
Omega	60.603	18.54	-174.46	-156.00	54.74	2.00	103	10.00	0.71073	50	30.0	n/a
Phi	60.603	18.54	-174.46	0.00	54.74	2.00	180	10.00	0.71073	50	30.0	n/a
Phi	60.603	0.00	0.00	0.00	54.74	360.00	1	108.00	0.71073	50	30.0	n/a

A total of 902 frames were collected. The total exposure time was 2.53 hours. The frames were integrated with the Bruker SAINT software package using a narrow-frame algorithm. The integration of the data using a monoclinic unit cell yielded a total of 49450 reflections to a maximum θ angle of 24.98° (0.84 Å resolution), of which 4634 were independent (average redundancy 10.671, completeness = 99.8%, R_{int} = 9.84%, R_{sig} = 5.17%) and 3120 (67.33%) were greater than $2\sigma(F^2)$. The final cell constants of a = 9.9777(9) Å, b = 12.3330(12) Å, c = 10.7825(10) Å, β = 96.725(3)°, volume = 1317.7(2) $Å^3$, are based upon the refinement of the XYZ-centroids of 6084 reflections above 20 σ(I) with 5.036° < 2θ < 39.67°. Data were corrected for absorption effects using the Multi-Scan method (SADABS). The ratio of minimum to maximum apparent transmission was 0.940. The calculated minimum and maximum transmission coefficients (based on crystal size) are 0.9870 and 0.9950.

The structure was solved and refined using the Bruker SHELXTL Software Package, using the space group P 1 21 1, with Z = 2 for the formula unit, $C_{28}H_{25}F_3N_2O_5$. The final anisotropic full-matrix least-squares refinement on F^2 with 359 variables converged at R1 = 5.65%, for the observed data and wR2 = 14.25% for all data. The goodness-of-fit was 1.028. The largest peak in the final difference electron density synthesis was 0.233 $e^-/Å^3$ and the largest hole was -0.218 $e^-/Å^3$ with an RMS deviation of 0.041 $e^-/Å^3$. On the basis of the final model, the calculated density was 1.327 g/cm^3 and F(000), 548 e^-.

The X-ray crystallography measurement was performed using the XRD at the National Research Facilities and Equipment Center (NanoBioEnergy Materials Center) at Ewha Womans University.

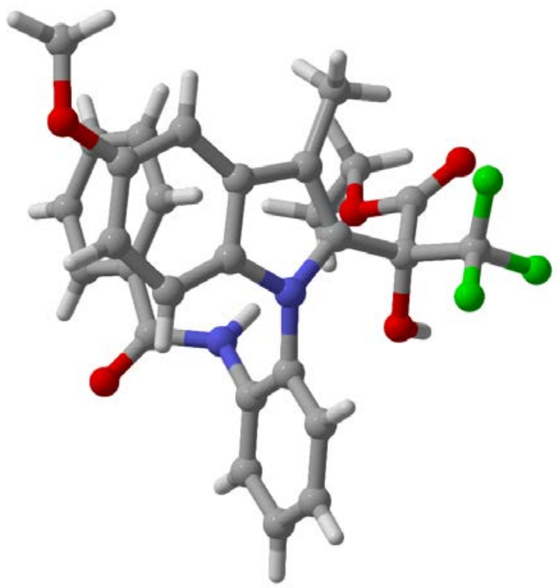

Supplementary Fig. 2 The single crystal X-ray structure of **3ld**; ellipsoid contour at the 50% probability level.

Supplementary Table 19. Sample and crystal data for 3ld.

**Chemical formula**	$C_{28}H_{25}F_3N_2O_5$	
**Formula weight**	526.50 g/mol	
**Temperature**	296(2) K	
**Wavelength**	0.71073 Å	
**Crystal size**	0.050 x 0.050 x 0.120 mm	
**Crystal habit**	colorless block	
**Crystal system**	monoclinic	
**Space group**	P 2_1	
**Unit cell dimensions**	a = 9.9777(9) Å	α = 90°
	b = 12.3330(12) Å	β = 96.725(3)°
	c = 10.7825(10) Å	γ = 90°
**Volume**	1317.7(2) $Å^3$	
**Z**	2	
**Density (calculated)**	1.327 g/cm^3	
**Absorption coefficient**	0.105 mm^{-1}	
**F(000)**	548	

Supplementary Table 20. Data collection and structure refinement for 3ld.

**Theta range for data collection**	1.90 to 24.98°
**Index ranges**	-11≤h≤11, -14≤k≤14, -12≤l≤12
**Reflections collected**	49450
**Independent reflections**	4634 [R(int) = 0.0984]
**Coverage of independent reflections**	99.8%
**Absorption correction**	Multi-Scan
**Max. and min. transmission**	0.9950 and 0.9870
**Structure solution technique**	direct methods
**Structure solution program**	SHELXT 2018/2 (Sheldrick, 2018)

**Refinement method**	Full-matrix least-squares on F^2	
**Refinement program**	SHELXL-2018/3 (Sheldrick, 2018)	
**Function minimized**	$\Sigma\, w(F_o^2 - F_c^2)^2$	
**Data / restraints / parameters**	4634 / 9 / 359	
**Goodness-of-fit on F^2**	1.028	
**Final R indices**	3120 data; I>2σ(I)	R_1 = 0.0565, wR_2 = 0.1238
	all data	R_1 = 0.0940, wR_2 = 0.1425
**Weighting scheme**	$w=1/[\sigma^2(F_o^2)+(0.0644P)^2+0.2787P]$ where $P=(F_o^2+2F_c^2)/3$	
**Absolute structure parameter**	0.0(5)	
**Extinction coefficient**	0.0160(30)	
**Largest diff. peak and hole**	0.233 and -0.218 eÅ^{-3}	

Supplementary Table 21. Atomic coordinates and equivalent isotropic atomic displacement parameters (Å^2) for 3ld.

U(eq) is defined as one third of the trace of the orthogonalized U_{ij} tensor.

	x/a	y/b	z/c	U(eq)
C1	0.7900(5)	0.4558(5)	0.6255(5)	0.0456(13)
C2	0.7871(6)	0.3693(5)	0.5422(6)	0.0611(16)
C3	0.7940(6)	0.2661(5)	0.5924(7)	0.0719(19)
C4	0.8035(6)	0.2498(5)	0.7206(7)	0.0634(17)
C5	0.8063(5)	0.3355(5)	0.8024(5)	0.0527(14)
C6	0.7989(5)	0.4405(5)	0.7543(5)	0.0470(13)
C7	0.8009(5)	0.5449(5)	0.8102(5)	0.0479(14)
C8	0.7913(5)	0.6202(5)	0.7152(5)	0.0444(13)
C9	0.8017(10)	0.1157(6)	0.8804(8)	0.101(3)
C10	0.8075(8)	0.5627(6)	0.9488(5)	0.0715(19)
C11	0.7553(5)	0.6039(5)	0.4750(5)	0.0453(13)
C12	0.8532(6)	0.6257(6)	0.4011(6)	0.0637(16)
C13	0.8184(7)	0.6574(7)	0.2786(6)	0.078(2)
C14	0.6850(8)	0.6700(7)	0.2327(6)	0.081(2)
C15	0.5849(6)	0.6466(6)	0.3081(5)	0.0663(18)
C16	0.6195(5)	0.6141(5)	0.4287(5)	0.0491(14)
C17	0.4256(5)	0.5166(5)	0.4952(5)	0.0504(13)
C18	0.3509(5)	0.4969(5)	0.6043(5)	0.0506(14)
C19	0.4172(6)	0.4932(6)	0.7249(6)	0.0707(19)
C20	0.3474(8)	0.4713(7)	0.8250(6)	0.085(2)
C21	0.2091(8)	0.4555(7)	0.8064(8)	0.085(2)
C22	0.1444(7)	0.4584(7)	0.6876(7)	0.087(2)
C23	0.2138(6)	0.4783(5)	0.5876(6)	0.0651(17)
C24	0.7865(6)	0.7421(5)	0.7222(5)	0.0511(14)
C25	0.9304(7)	0.7860(6)	0.7504(7)	0.0693(17)
C26	0.6985(7)	0.7799(5)	0.8218(6)	0.0599(15)
C27	0.4772(9)	0.7814(9)	0.8748(9)	0.139(4)
C28	0.3431(12)	0.7997(19)	0.7923(13)	0.124(8)
C28*	0.393(4)	0.877(3)	0.815(3)	0.137(15)
F1	0.0058(4)	0.7537(4)	0.6638(4)	0.0977(14)
F2	0.9912(4)	0.7553(4)	0.8602(4)	0.0924(13)

F3	0.9328(5)	0.8949(4)	0.7494(4)	0.0990(14)
N1	0.7886(4)	0.5651(4)	0.6007(4)	0.0447(11)
N2	0.5208(4)	0.5932(4)	0.5117(4)	0.0552(12)
O1	0.8077(5)	0.1413(4)	0.7548(5)	0.0887(16)
O2	0.4013(4)	0.4671(4)	0.3968(4)	0.0664(12)
O3	0.7335(5)	0.7825(4)	0.6059(4)	0.0678(12)
O4	0.7349(5)	0.8327(5)	0.9101(5)	0.0899(15)
O5	0.5721(5)	0.7478(4)	0.7897(4)	0.0854(15)

Supplementary Table 22. Bond lengths (Å) for 3ld.

C1-N1	1.375(7)	C1-C2	1.393(7)
C1-C6	1.395(7)	C2-C3	1.381(9)
C2-H2	0.93	C3-C4	1.389(9)
C3-H3	0.93	C4-C5	1.375(8)
C4-O1	1.387(8)	C5-C6	1.394(8)
C5-H5	0.93	C6-C7	1.421(8)
C7-C8	1.378(7)	C7-C10	1.504(8)
C8-N1	1.407(6)	C8-C24	1.506(8)
C9-O1	1.399(9)	C9-H9A	0.96
C9-H9B	0.96	C9-H9C	0.96
C10-H10A	0.96	C10-H10B	0.96
C10-H10C	0.96	C11-C12	1.358(7)
C11-C16	1.393(7)	C11-N1	1.439(7)
C12-C13	1.382(9)	C12-H12	0.93
C13-C14	1.374(9)	C13-H13	0.93
C14-C15	1.389(9)	C14-H14	0.93
C15-C16	1.366(7)	C15-H15	0.93
C16-N2	1.430(7)	C17-O2	1.224(6)
C17-N2	1.337(7)	C17-C18	1.484(8)
C18-C23	1.378(8)	C18-C19	1.388(8)
C19-C20	1.378(9)	C19-H19	0.93
C20-C21	1.385(10)	C20-H20	0.93
C21-C22	1.366(10)	C21-H21	0.93
C22-C23	1.370(9)	C22-H22	0.93
C23-H23	0.93	C24-O3	1.394(6)
C24-C25	1.531(9)	C24-C26	1.537(8)
C25-F2	1.320(7)	C25-F1	1.327(8)
C25-F3	1.344(7)	C26-O4	1.175(7)
C26-O5	1.328(8)	C27-O5	1.454(9)
C27-C28	1.534(3)	C27-C28*	1.538(3)
C27-H27A	0.97	C27-H27B	0.97
C28-H28A	0.96	C28-H28B	0.96
C28-H28C	0.96	C28*-H28D	0.96
C28*-H28E	0.96	C28*-H28F	0.96
N2-H2A	0.86	O3-H3A	0.82

Supplementary Table 23. Bond angles (°) for 3ld.

N1-C1-C2	128.9(5)	N1-C1-C6	108.9(4)
C2-C1-C6	122.2(5)	C3-C2-C1	117.2(6)
C3-C2-H2	121.4	C1-C2-H2	121.4
C2-C3-C4	121.2(6)	C2-C3-H3	119.4
C4-C3-H3	119.4	C5-C4-O1	125.0(6)
C5-C4-C3	121.4(6)	O1-C4-C3	113.6(6)
C4-C5-C6	118.6(5)	C4-C5-H5	120.7
C6-C5-H5	120.7	C5-C6-C1	119.4(5)
C5-C6-C7	133.4(5)	C1-C6-C7	107.2(5)
C8-C7-C6	107.4(5)	C8-C7-C10	129.2(5)
C6-C7-C10	123.4(5)	C7-C8-N1	108.6(5)
C7-C8-C24	129.5(5)	N1-C8-C24	121.9(5)
O1-C9-H9A	109.5	O1-C9-H9B	109.5
H9A-C9-H9B	109.5	O1-C9-H9C	109.5
H9A-C9-H9C	109.5	H9B-C9-H9C	109.5
C7-C10-H10A	109.5	C7-C10-H10B	109.5
H10A-C10-H10B	109.5	C7-C10-H10C	109.5
H10A-C10-H10C	109.5	H10B-C10-H10C	109.5
C12-C11-C16	120.5(5)	C12-C11-N1	121.0(5)
C16-C11-N1	118.4(4)	C11-C12-C13	119.9(6)
C11-C12-H12	120.0	C13-C12-H12	120.0
C14-C13-C12	120.1(6)	C14-C13-H13	120.0
C12-C13-H13	120.0	C13-C14-C15	119.9(6)
C13-C14-H14	120.1	C15-C14-H14	120.1
C16-C15-C14	119.9(6)	C16-C15-H15	120.0
C14-C15-H15	120.0	C15-C16-C11	119.6(5)
C15-C16-N2	122.2(5)	C11-C16-N2	118.1(4)
O2-C17-N2	122.2(5)	O2-C17-C18	122.6(5)
N2-C17-C18	115.1(5)	C23-C18-C19	118.3(6)
C23-C18-C17	120.4(5)	C19-C18-C17	121.3(5)
C20-C19-C18	120.7(6)	C20-C19-H19	119.6
C18-C19-H19	119.6	C19-C20-C21	120.1(7)
C19-C20-H20	120.0	C21-C20-H20	120.0
C22-C21-C20	119.0(7)	C22-C21-H21	120.5
C20-C21-H21	120.5	C21-C22-C23	121.1(6)
C21-C22-H22	119.5	C23-C22-H22	119.5
C22-C23-C18	120.8(6)	C22-C23-H23	119.6
C18-C23-H23	119.6	O3-C24-C8	108.8(4)
O3-C24-C25	107.5(5)	C8-C24-C25	109.1(5)
O3-C24-C26	109.4(5)	C8-C24-C26	111.1(5)
C25-C24-C26	110.7(5)	F2-C25-F1	107.8(6)
F2-C25-F3	106.6(6)	F1-C25-F3	106.4(6)
F2-C25-C24	113.5(5)	F1-C25-C24	110.5(5)
F3-C25-C24	111.6(6)	O4-C26-O5	124.8(6)
O4-C26-C24	125.9(6)	O5-C26-C24	109.3(5)
O5-C27-C28	105.3(8)	O5-C27-C28*	108.4(16)
O5-C27-H27A	110.7	C28-C27-H27A	110.7
O5-C27-H27B	110.7	C28-C27-H27B	110.7
H27A-C27-H27B	108.8	C27-C28-H28A	109.5

C27-C28-H28B	109.5	H28A-C28-H28B	109.5
C27-C28-H28C	109.5	H28A-C28-H28C	109.5
H28B-C28-H28C	109.5	C27-C28*-H28D	109.5
C27-C28*-H28E	109.5	H28D-C28*-H28E	109.5
C27-C28*-H28F	109.5	H28D-C28*-H28F	109.5
H28E-C28*-H28F	109.5	C1-N1-C8	107.7(4)
C1-N1-C11	120.3(4)	C8-N1-C11	130.2(4)
C17-N2-C16	125.1(5)	C17-N2-H2A	117.4
C16-N2-H2A	117.4	C4-O1-C9	118.1(5)
C24-O3-H3A	109.5	C26-O5-C27	114.7(6)

Supplementary Table 24. Anisotropic atomic displacement parameters (Å^2) for 3ld.

The anisotropic atomic displacement factor exponent takes the form: $-2\pi^2[h^2 a^{*2} U_{11} + ... + 2 h k a^* b^* U_{12}]$

	U_{11}	U_{22}	U_{33}	U_{23}	U_{13}	U_{12}
C1	0.040(3)	0.048(4)	0.048(3)	-0.004(3)	0.004(2)	0.004(2)
C2	0.068(4)	0.056(4)	0.059(4)	-0.012(3)	0.003(3)	0.002(3)
C3	0.070(4)	0.058(5)	0.088(5)	-0.027(4)	0.009(3)	0.000(3)
C4	0.060(4)	0.047(4)	0.082(5)	-0.003(3)	0.003(3)	0.001(3)
C5	0.052(3)	0.051(4)	0.054(4)	0.002(3)	0.005(3)	0.000(3)
C6	0.035(3)	0.050(4)	0.055(3)	-0.004(3)	0.004(2)	0.002(2)
C7	0.053(3)	0.048(4)	0.043(3)	-0.002(3)	0.007(3)	0.000(2)
C8	0.040(3)	0.049(3)	0.044(3)	-0.009(3)	0.005(2)	0.001(2)
C9	0.129(7)	0.054(4)	0.117(7)	0.015(5)	0.003(5)	-0.005(4)
C10	0.103(5)	0.066(4)	0.046(3)	0.000(3)	0.011(3)	-0.002(4)
C11	0.042(3)	0.054(3)	0.041(3)	-0.002(3)	0.007(2)	-0.003(2)
C12	0.047(3)	0.084(4)	0.061(4)	-0.005(3)	0.010(3)	-0.003(3)
C13	0.080(5)	0.111(6)	0.048(4)	0.006(4)	0.026(3)	-0.017(4)
C14	0.096(6)	0.110(6)	0.036(3)	0.000(4)	0.008(4)	-0.005(5)
C15	0.062(4)	0.087(5)	0.049(4)	0.001(3)	0.001(3)	0.006(3)
C16	0.050(3)	0.060(4)	0.037(3)	-0.007(3)	0.006(2)	-0.009(3)
C17	0.038(3)	0.059(4)	0.052(4)	-0.005(3)	-0.001(2)	0.000(3)
C18	0.045(3)	0.052(3)	0.054(3)	-0.001(3)	0.004(3)	0.001(3)
C19	0.057(4)	0.097(5)	0.058(4)	0.005(4)	0.008(3)	-0.007(4)
C20	0.084(5)	0.118(7)	0.053(4)	0.009(4)	0.009(3)	-0.014(5)
C21	0.074(5)	0.100(6)	0.088(5)	-0.010(5)	0.035(4)	-0.014(4)
C22	0.051(4)	0.122(6)	0.091(5)	-0.002(5)	0.021(4)	-0.011(4)
C23	0.045(3)	0.075(4)	0.074(4)	-0.003(3)	0.005(3)	-0.001(3)
C24	0.061(4)	0.049(3)	0.043(3)	-0.001(3)	0.003(3)	0.003(3)
C25	0.080(5)	0.057(5)	0.071(5)	0.001(4)	0.010(4)	-0.013(4)
C26	0.073(4)	0.057(4)	0.050(4)	-0.011(3)	0.007(3)	0.006(3)
C27	0.113(8)	0.186(12)	0.126(8)	-0.044(8)	0.048(6)	0.039(7)
C28	0.084(11)	0.141(18)	0.149(14)	0.003(11)	0.028(9)	0.034(10)
C28*	0.137(15)	0.138(15)	0.137(15)	0.000(3)	0.017(3)	0.000(3)
F1	0.079(3)	0.104(3)	0.117(3)	-0.010(3)	0.040(2)	-0.018(2)
F2	0.075(2)	0.100(3)	0.095(3)	0.007(2)	-0.022(2)	-0.020(2)

F3	0.118(3)	0.061(3)	0.118(4)	-0.006(2)	0.012(3)	-0.031(2)
N1	0.046(2)	0.050(3)	0.038(3)	-0.004(2)	0.0048(19)	0.002(2)
N2	0.045(3)	0.072(3)	0.050(3)	-0.013(2)	0.015(2)	-0.010(2)
O1	0.113(4)	0.047(3)	0.105(4)	-0.003(3)	0.007(3)	0.000(3)
O2	0.074(3)	0.068(3)	0.057(3)	-0.006(2)	0.007(2)	-0.017(2)
O3	0.100(3)	0.051(3)	0.051(2)	0.0007(19)	0.003(2)	0.018(2)
O4	0.110(4)	0.095(4)	0.065(3)	-0.035(3)	0.013(3)	-0.002(3)
O5	0.062(3)	0.108(4)	0.089(3)	-0.030(3)	0.020(2)	0.011(3)

Supplementary Table 25. Hydrogen atomic coordinates and isotropic atomic displacement parameters (Å^2) for 3ld.

	x/a	y/b	z/c	U(eq)
H2	0.7807	0.3805	0.4564	0.073
H3	0.7923	0.2065	0.5394	0.086
H5	0.8130	0.3236	0.8881	0.063
H9A	0.8788	0.1458	0.9303	0.152
H9B	0.8016	0.0383	0.8904	0.152
H9C	0.7207	0.1453	0.9068	0.152
H10A	0.8180	0.6387	0.9667	0.107
H10B	0.8831	0.5237	0.9905	0.107
H10C	0.7258	0.5370	0.9776	0.107
H12	0.9436	0.6194	0.4329	0.076
H13	0.8854	0.6702	0.2272	0.094
H14	0.6617	0.6941	0.1513	0.097
H15	0.4945	0.6530	0.2764	0.08
H19	0.5097	0.5056	0.7382	0.085
H20	0.3933	0.4671	0.9051	0.102
H21	0.1609	0.4431	0.8739	0.102
H22	0.0518	0.4466	0.6743	0.104
H23	0.1678	0.4794	0.5075	0.078
H27A	0.5074	0.8477	0.9177	0.167
H27B	0.4677	0.7255	0.9365	0.167
H28A	0.3608	0.8238	0.7111	0.185
H28B	0.2914	0.8537	0.8297	0.185
H28C	0.2933	0.7330	0.7844	0.185
H28D	0.3924	0.8739	0.7262	0.205
H28E	0.4327	0.9438	0.8462	0.205
H28F	0.3027	0.8715	0.8362	0.205
H2A	0.5228	0.6330	0.5774	0.066
H3A	0.6994	0.8421	0.6149	0.102

11.3 X-ray Crystallography of 7da

Experimental details

A colorless block-like specimen of $C_{30}H_{30}N_2O_7$, approximate dimensions 0.100 mm x 0.100 mm x 0.160 mm, was used for the X-ray crystallographic analysis. The X-ray intensity data were measured (λ = 0.71073 Å).

Supplementary Table 26. Data collection details for 7da.

Axis	dx/mm	2θ/°	ω/°	φ/°	χ/°	Width/°	Frames	Time/s	Wavelength/Å	Voltage/kV	Current/mA	Temperature/K
Phi	60.606	0.00	0.00	0.00	54.74	1.00	180	1.20	0.71073	50	30.0	n/a
Phi	60.606	0.00	0.00	180.00	54.74	1.00	180	1.20	0.71073	50	30.0	n/a
Phi	60.606	18.54	-174.46	0.00	54.74	1.00	360	10.00	0.71073	50	30.0	n/a
Phi	60.606	18.54	31.54	0.00	54.74	1.00	360	10.00	0.71073	50	30.0	n/a
Omega	60.606	18.54	-174.46	-156.00	54.74	1.00	206	10.00	0.71073	50	30.0	n/a
Omega	60.606	27.81	-165.19	0.00	54.74	1.00	206	10.00	0.71073	50	30.0	n/a
Omega	60.606	18.54	-174.46	102.00	54.74	1.00	206	10.00	0.71073	50	30.0	n/a
Omega	60.606	18.54	-174.46	-105.00	54.74	1.00	206	10.00	0.71073	50	30.0	n/a
Phi	60.606	0.00	0.00	0.00	54.74	360.00	1	108.00	0.71073	50	30.0	n/a

A total of 1905 frames were collected. The total exposure time was 4.44 hours. The frames were integrated with the Bruker SAINT software package using a narrow-frame algorithm. The integration of the data using an orthorhombic unit cell yielded a total of 116041 reflections to a maximum θ angle of 28.29° (0.75 Å resolution), of which 7043 were independent (average redundancy 16.476, completeness = 99.7%, R_{int} = 7.79%, R_{sig} = 3.47%) and 4914 (69.77%) were greater than 2σ(F2). The final cell constants of a = 13.5848(4) Å, b = 13.6768(5) Å, c = 15.2828(5) Å, volume = 2839.49(16) $Å^3$, are based upon the refinement of the XYZ-centroids of 9352 reflections above 20 σ(I) with 4.996° < 2θ < 42.91°. Data were corrected for absorption effects using the Multi-Scan method (SADABS). The ratio of minimum to maximum apparent transmission was 0.933. The calculated minimum and maximum transmission coefficients (based on crystal size) are 0.9860 and 0.9910.

The structure was solved and refined using the Bruker SHELXTL Software Package, using the space group P 21 21 21, with Z = 4 for the formula unit, $C_{30}H_{30}N_2O_7$. The final anisotropic full-matrix least-squares refinement on F2 with 378 variables converged at R1 = 5.72%, for the observed data and wR2 = 14.77% for all data. The goodness-of-fit was 0.961. The largest peak in the final difference electron density synthesis was 0.196 $e^-/Å^3$ and the largest hole was −0.187 $e^-/Å^3$ with an RMS deviation of 0.031 $e^-/Å^3$. On the basis of the final model, the calculated density was 1.241 g/cm^3 and F(000), 1120 e^-. CCDC number 2219782 (**7da**) contains the supplementary crystallographic data for this paper. These data can be obtained free of charge from The Cambridge Crystallographic Data Center via www.ccdc.cam.ac.uk/data_request/cif.

In CheckCif, one alert level B was detected, which is PLAT414_ALERT_2_B Short Intra D-H..H-X. The reason for this alert may be attributed to rapid amide-iminol tautomerism causing small dihedral angle between N-H and H-C.

The X-ray crystallography measurement was performed using the XRD at the National Research Facilities and Equipment Center (NanoBioEnergy Materials Center) at Ewha Womans University.

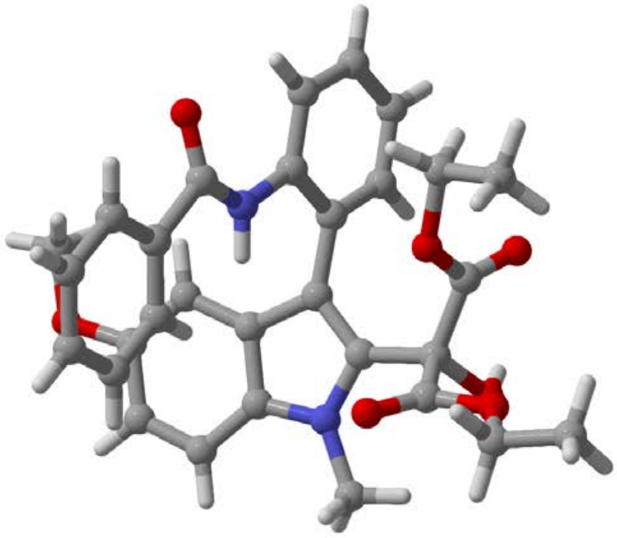

Supplementary Fig. 3 The single crystal X-ray structure of **7da**; ellipsoid contour at the 50% probability level.

Supplementary Table 27. Sample and crystal data for 7da.

**Chemical formula**	$C_{30}H_{30}N_2O_7$	
**Formula weight**	530.56 g/mol	
**Temperature**	296(2) K	
**Wavelength**	0.71073 Å	
**Crystal size**	0.100 x 0.100 x 0.160 mm	
**Crystal habit**	colorless block	
**Crystal system**	orthorhombic	
**Space group**	P 2_1 2_1 2_1	
**Unit cell dimensions**	a = 13.5848(4) Å	α = 90°
	b = 13.6768(5) Å	β = 90°
	c = 15.2828(5) Å	γ = 90°
**Volume**	2839.49(16) $Å^3$	
**Z**	4	
**Density (calculated)**	1.241 g/cm^3	
**Absorption coefficient**	0.089 mm^{-1}	
**F(000)**	1120	

Supplementary Table 28. Data collection and structure refinement for 7da.

**Theta range for data collection**	2.00 to 28.29°
**Index ranges**	-18≤h≤18, -18≤k≤18, -20≤l≤20
**Reflections collected**	116041
**Independent reflections**	7043 [R(int) = 0.0779]
**Coverage of independent reflections**	99.7%
**Absorption correction**	Multi-Scan
**Max. and min. transmission**	0.9910 and 0.9860
**Structure solution technique**	direct methods
**Structure solution program**	SHELXT 2018/2 (Sheldrick, 2018)

**Refinement method**	Full-matrix least-squares on F^2	
**Refinement program**	SHELXL-2018/3 (Sheldrick, 2018)	
**Function minimized**	$\Sigma w(F_o^2 - F_c^2)^2$	
**Data / restraints / parameters**	7043 / 28 / 378	
**Goodness-of-fit on F^2**	0.961	
**Final R indices**	4914 data; $I>2\sigma(I)$	$R_1 = 0.0572$, $wR_2 = 0.1306$
	all data	$R_1 = 0.0891$, $wR_2 = 0.1477$
**Weighting scheme**	$w=1/[\sigma^2(F_o^2)+(0.0721P)^2+0.6367P]$ where $P=(F_o^2+2F_c^2)/3$	
**Absolute structure parameter**	-0.4(4)	
**Largest diff. peak and hole**	0.196 and -0.187 eÅ^{-3}	
**R.M.S. deviation from mean**	0.031 eÅ^{-3}	

Supplementary Table 29. Atomic coordinates and equivalent isotropic atomic displacement parameters (Å^2) for 7da.

U(eq) is defined as one third of the trace of the orthogonalized U_{ij} tensor.

	x/a	y/b	z/c	U(eq)
C1	0.6368(2)	0.5714(2)	0.33820(19)	0.0502(7)
C2	0.7334(2)	0.5881(2)	0.36146(19)	0.0465(7)
C3	0.7569(2)	0.6855(2)	0.33203(19)	0.0474(7)
C4	0.8414(2)	0.7439(2)	0.3400(2)	0.0511(7)
C5	0.8374(3)	0.8369(2)	0.3057(2)	0.0585(8)
C6	0.7528(3)	0.8718(3)	0.2647(3)	0.0756(11)
C7	0.6702(3)	0.8163(3)	0.2558(3)	0.0704(10)
C8	0.6728(2)	0.7218(2)	0.2905(2)	0.0561(8)
C9	0.5033(3)	0.6662(3)	0.2553(3)	0.0748(11)
C10	0.5688(2)	0.4871(3)	0.3582(2)	0.0539(8)
C11	0.6170(3)	0.4075(3)	0.4146(3)	0.0636(9)
C12	0.6726(6)	0.3816(7)	0.5692(7)	0.074(3)
C13	0.5926(6)	0.3226(8)	0.6084(7)	0.113(4)
C12*	0.6577(14)	0.3449(12)	0.5440(8)	0.071(4)
C13*	0.6467(16)	0.3761(13)	0.6362(8)	0.108(7)
C14	0.4805(3)	0.5246(3)	0.4137(2)	0.0586(8)
C15	0.3311(3)	0.4779(4)	0.4803(4)	0.1043(17)
C16	0.2762(5)	0.3906(5)	0.4965(5)	0.145(3)
C17	0.8048(2)	0.5239(2)	0.4070(2)	0.0463(7)
C18	0.8344(2)	0.5462(2)	0.4924(2)	0.0505(7)
C19	0.9040(3)	0.4881(3)	0.5350(2)	0.0634(9)
C20	0.9437(3)	0.4092(3)	0.4919(3)	0.0724(10)
C21	0.9150(3)	0.3863(3)	0.4088(3)	0.0734(11)
C22	0.8460(3)	0.4436(2)	0.3664(2)	0.0616(9)
C23	0.7904(2)	0.6611(2)	0.61224(17)	0.0559(8)
C24	0.72540(16)	0.74990(14)	0.62745(14)	0.0551(8)
C25	0.66704(19)	0.79214(18)	0.56302(12)	0.0699(10)
C26	0.60639(19)	0.87073(18)	0.58349(18)	0.0867(13)
C27	0.60410(19)	0.90708(16)	0.6684(2)	0.0877(14)
C28	0.6625(2)	0.86484(19)	0.73284(15)	0.0865(13)
C29	0.72311(18)	0.78625(17)	0.71237(13)	0.0678(10)

C30	0.0050(3)	0.8691(3)	0.3396(3)	0.0724(10)
N1	0.6011(2)	0.6511(2)	0.29264(19)	0.0579(7)
N2	0.7880(3)	0.6269(2)	0.53042(17)	0.0695(9)
O1	0.52981(19)	0.4449(2)	0.28175(16)	0.0708(7)
O2	0.6387(2)	0.3293(2)	0.3878(2)	0.0950(10)
O3	0.62691(19)	0.4383(2)	0.49706(18)	0.0733(7)
O4	0.4757(2)	0.6037(2)	0.44611(18)	0.0804(8)
O5	0.41445(19)	0.4546(2)	0.42260(19)	0.0766(8)
O6	0.8566(16)	0.6417(15)	0.6613(9)	0.098(6)
O6*	0.8066(19)	0.6019(12)	0.6733(6)	0.063(5)
O7	0.9139(2)	0.90296(17)	0.3078(2)	0.0738(7)

Supplementary Table 30. Bond lengths (Å) for 7da.

C1-C2	1.379(4)	C1-N1	1.381(4)
C1-C10	1.509(5)	C2-C3	1.442(4)
C2-C17	1.482(4)	C3-C8	1.398(4)
C3-C4	1.404(5)	C4-C5	1.377(5)
C4-H4	0.93	C5-O7	1.377(4)
C5-C6	1.394(5)	C6-C7	1.361(6)
C6-H6	0.93	C7-C8	1.398(5)
C7-H7	0.93	C8-N1	1.373(4)
C9-N1	1.461(4)	C9-H9A	0.96
C9-H9B	0.96	C9-H9C	0.96
C10-O1	1.407(4)	C10-C11	1.534(5)
C10-C14	1.556(5)	C11-O2	1.182(4)
C11-O3	1.336(5)	C12-C13	1.480(3)
C12-O3	1.484(10)	C12-H12A	0.97
C12-H12B	0.97	C13-H13A	0.96
C13-H13B	0.96	C13-H13C	0.96
C12*-C13*	1.480(3)	C12*-O3	1.523(15)
C12*-H12C	0.97	C12*-H12D	0.97
C13*-H13D	0.96	C13*-H13E	0.96
C13*-H13F	0.96	C14-O4	1.191(4)
C14-O5	1.320(4)	C15-C16	1.430(8)
C15-O5	1.470(5)	C15-H15A	0.97
C15-H15B	0.97	C16-H16A	0.96
C16-H16B	0.96	C16-H16C	0.96
C17-C22	1.380(4)	C17-C18	1.400(4)
C18-C19	1.396(4)	C18-N2	1.398(4)
C19-C20	1.375(5)	C19-H19	0.93
C20-C21	1.365(6)	C20-H20	0.93
C21-C22	1.383(5)	C21-H21	0.93
C22-H22	0.93	C23-O6	1.201(7)
C23-O6*	1.255(8)	C23-N2	1.336(2)
C23-C24	1.519(2)	C24-C25	1.39
C24-C29	1.39	C25-C26	1.39
C25-H25	0.93	C26-C27	1.39
C26-H26	0.93	C27-C28	1.39
C27-H27	0.93	C28-C29	1.39
C28-H28	0.93	C29-H29	0.93

C30-O7	1.408(5)	C30-H30A	0.96
C30-H30B	0.96	C30-H30C	0.96
N2-H2	0.91(5)	O1-H1	0.82

Supplementary Table 31. Bond angles (°) for 7da.

C2-C1-N1	109.5(3)	C2-C1-C10	131.0(3)
N1-C1-C10	119.4(3)	C1-C2-C3	106.4(3)
C1-C2-C17	130.2(3)	C3-C2-C17	123.3(3)
C8-C3-C4	120.4(3)	C8-C3-C2	106.8(3)
C4-C3-C2	132.8(3)	C5-C4-C3	117.5(3)
C5-C4-H4	121.3	C3-C4-H4	121.3
C4-C5-O7	124.6(3)	C4-C5-C6	121.3(3)
O7-C5-C6	114.1(3)	C7-C6-C5	122.3(3)
C7-C6-H6	118.9	C5-C6-H6	118.9
C6-C7-C8	117.2(3)	C6-C7-H7	121.4
C8-C7-H7	121.4	N1-C8-C3	108.6(3)
N1-C8-C7	130.0(3)	C3-C8-C7	121.4(3)
N1-C9-H9A	109.5	N1-C9-H9B	109.5
H9A-C9-H9B	109.5	N1-C9-H9C	109.5
H9A-C9-H9C	109.5	H9B-C9-H9C	109.5
O1-C10-C1	112.1(3)	O1-C10-C11	109.6(3)
C1-C10-C11	113.3(3)	O1-C10-C14	107.3(3)
C1-C10-C14	109.2(3)	C11-C10-C14	104.9(3)
O2-C11-O3	125.9(4)	O2-C11-C10	123.6(4)
O3-C11-C10	110.4(3)	C13-C12-O3	106.1(7)
C13-C12-H12A	110.5	O3-C12-H12A	110.5
C13-C12-H12B	110.5	O3-C12-H12B	110.5
H12A-C12-H12B	108.7	C12-C13-H13A	109.5
C12-C13-H13B	109.5	H13A-C13-H13B	109.5
C12-C13-H13C	109.5	H13A-C13-H13C	109.5
H13B-C13-H13C	109.5	C13*-C12*-O3	100.3(11)
C13*-C12*-H12C	111.7	O3-C12*-H12C	111.7
C13*-C12*-H12D	111.7	O3-C12*-H12D	111.7
H12C-C12*-H12D	109.5	C12*-C13*-H13D	109.5
C12*-C13*-H13E	109.5	H13D-C13*-H13E	109.5
C12*-C13*-H13F	109.5	H13D-C13*-H13F	109.5
H13E-C13*-H13F	109.5	O4-C14-O5	125.3(3)
O4-C14-C10	124.7(3)	O5-C14-C10	109.9(3)
C16-C15-O5	109.0(5)	C16-C15-H15A	109.9
O5-C15-H15A	109.9	C16-C15-H15B	109.9
O5-C15-H15B	109.9	H15A-C15-H15B	108.3
C15-C16-H16A	109.5	C15-C16-H16B	109.5
H16A-C16-H16B	109.5	C15-C16-H16C	109.5
H16A-C16-H16C	109.5	H16B-C16-H16C	109.5
C22-C17-C18	118.4(3)	C22-C17-C2	121.8(3)
C18-C17-C2	119.8(3)	C19-C18-N2	124.1(3)
C19-C18-C17	120.4(3)	N2-C18-C17	115.5(3)
C20-C19-C18	119.3(3)	C20-C19-H19	120.4
C18-C19-H19	120.4	C21-C20-C19	120.9(3)
C21-C20-H20	119.5	C19-C20-H20	119.5

C20-C21-C22	119.9(3)	C20-C21-H21	120.0
C22-C21-H21	120.0	C17-C22-C21	121.1(3)
C17-C22-H22	119.5	C21-C22-H22	119.5
O6-C23-N2	121.7(5)	O6*-C23-N2	118.3(7)
O6-C23-C24	121.1(4)	O6*-C23-C24	120.3(5)
N2-C23-C24	114.1(2)	C25-C24-C29	120.0
C25-C24-C23	123.73(17)	C29-C24-C23	116.21(17)
C24-C25-C26	120.0	C24-C25-H25	120.0
C26-C25-H25	120.0	C25-C26-C27	120.0
C25-C26-H26	120.0	C27-C26-H26	120.0
C28-C27-C26	120.0	C28-C27-H27	120.0
C26-C27-H27	120.0	C27-C28-C29	120.0
C27-C28-H28	120.0	C29-C28-H28	120.0
C28-C29-C24	120.0	C28-C29-H29	120.0
C24-C29-H29	120.0	O7-C30-H30A	109.5
O7-C30-H30B	109.5	H30A-C30-H30B	109.5
O7-C30-H30C	109.5	H30A-C30-H30C	109.5
H30B-C30-H30C	109.5	C8-N1-C1	108.6(2)
C8-N1-C9	122.4(3)	C1-N1-C9	128.9(3)
C23-N2-C18	130.9(3)	C23-N2-H2	116.(3)
C18-N2-H2	113.(3)	C10-O1-H1	109.5
C11-O3-C12	125.4(5)	C11-O3-C12*	102.0(5)
C14-O5-C15	115.4(3)	C5-O7-C30	117.1(3)

Supplementary Table 32. Anisotropic atomic displacement parameters (Å^2) for 7da.

The anisotropic atomic displacement factor exponent takes the form: $-2\pi^2[h^2 a^{*2} U_{11} + ... + 2 h k a^* b^* U_{12}]$

	U_{11}	U_{22}	U_{33}	U_{23}	U_{13}	U_{12}
C1	0.0568(17)	0.0525(18)	0.0413(15)	0.0047(14)	0.0016(13)	0.0007(14)
C2	0.0551(17)	0.0462(16)	0.0381(14)	0.0043(12)	-0.0017(13)	0.0053(14)
C3	0.0542(17)	0.0437(16)	0.0443(15)	0.0067(13)	-0.0046(13)	0.0045(13)
C4	0.0539(17)	0.0478(17)	0.0517(17)	0.0056(14)	-0.0054(14)	0.0064(14)
C5	0.062(2)	0.0476(17)	0.066(2)	0.0086(16)	-0.0022(17)	-0.0013(15)
C6	0.081(3)	0.053(2)	0.093(3)	0.029(2)	-0.007(2)	0.0062(19)
C7	0.069(2)	0.063(2)	0.080(2)	0.027(2)	-0.015(2)	0.0117(19)
C8	0.0553(17)	0.0569(19)	0.0561(19)	0.0156(16)	-0.0050(15)	0.0049(15)

C9	0.061(2)	0.084(3)	0.079(2)	0.013(2)	-0.0229(18)	0.006(2)
C10	0.0581(18)	0.0546(18)	0.0491(17)	-0.0056(15)	0.0017(14)	-0.0068(15)
C11	0.065(2)	0.053(2)	0.073(2)	0.0100(18)	0.0172(18)	-0.0035(17)
C12	0.082(5)	0.069(6)	0.070(6)	0.015(4)	-0.006(4)	-0.009(4)
C13	0.123(7)	0.126(7)	0.090(6)	0.054(6)	-0.009(5)	-0.049(6)
C12*	0.084(8)	0.061(8)	0.068(7)	0.017(6)	-0.012(6)	-0.002(6)
C13*	0.142(15)	0.114(12)	0.069(9)	0.020(8)	0.008(9)	0.029(11)
C14	0.0607(19)	0.064(2)	0.0506(18)	-0.0001(17)	0.0041(15)	-0.0014(17)
C15	0.067(3)	0.119(4)	0.127(4)	-0.008(3)	0.033(3)	-0.006(3)
C16	0.121(5)	0.164(6)	0.149(6)	0.007(5)	0.047(4)	-0.043(5)
C17	0.0495(15)	0.0398(15)	0.0496(16)	0.0082(13)	0.0018(13)	0.0047(12)
C18	0.0547(16)	0.0470(16)	0.0497(17)	0.0046(13)	-0.0019(14)	0.0118(14)
C19	0.065(2)	0.064(2)	0.062(2)	0.0084(17)	-0.0080(17)	0.0205(18)
C20	0.074(2)	0.065(2)	0.078(3)	0.012(2)	0.001(2)	0.0327(19)
C21	0.090(3)	0.056(2)	0.074(3)	-0.0022(19)	0.017(2)	0.028(2)
C22	0.076(2)	0.0512(18)	0.0575(19)	0.0010(15)	0.0065(17)	0.0120(18)
C23	0.0594(19)	0.0568(19)	0.0513(18)	-0.0044(16)	-0.0092(16)	0.0080(16)
C24	0.0549(17)	0.0478(17)	0.0626(19)	-0.0073(15)	-0.0016(16)	-0.0008(15)
C25	0.072(2)	0.063(2)	0.074(2)	-0.0068(19)	-0.004(2)	0.0169(19)
C26	0.074(3)	0.067(2)	0.119(4)	0.000(3)	-0.004(3)	0.020(2)
C27	0.080(3)	0.056(2)	0.128(4)	-0.031(3)	0.016(3)	0.008(2)
C28	0.092(3)	0.066(2)	0.102(3)	-0.036(2)	0.018(3)	-0.006(2)
C29	0.073(2)	0.062(2)	0.069(2)	-0.0170(19)	0.0023(19)	-0.0117(19)

C30	0.068(2)	0.061(2)	0.089(3)	-0.002(2)	0.003(2)	-0.0102(19)
N1	0.0522(15)	0.0642(17)	0.0573(16)	0.0142(14)	-0.0116(13)	0.0024(13)
N2	0.090(2)	0.0675(19)	0.0508(16)	-0.0029(14)	-0.0164(16)	0.0384(17)
O1	0.0716(15)	0.0775(17)	0.0634(15)	-0.0240(13)	0.0064(13)	-0.0125(14)
O2	0.110(2)	0.0511(15)	0.123(3)	0.0030(17)	0.023(2)	0.0055(16)
O3	0.0769(16)	0.0805(17)	0.0626(16)	0.0269(14)	0.0121(13)	0.0131(14)
O4	0.097(2)	0.0686(17)	0.0754(18)	-0.0138(15)	0.0209(16)	-0.0010(15)
O5	0.0623(14)	0.0763(16)	0.0911(19)	-0.0087(15)	0.0184(14)	-0.0104(13)
O6	0.108(9)	0.103(9)	0.081(5)	-0.025(6)	-0.050(6)	0.042(8)
O6*	0.090(10)	0.060(6)	0.038(4)	0.008(3)	-0.011(4)	0.015(6)
O7	0.0719(15)	0.0495(13)	0.100(2)	0.0165(13)	-0.0061(15)	-0.0074(12)

Supplementary Table 33. Hydrogen atomic coordinates and isotropic atomic displacement parameters (Å^2) for 7da.

	x/a	y/b	z/c	U(eq)
H4	0.8978	0.7207	0.3674	0.061
H6	0.7527	0.9352	0.2427	0.091
H7	0.6145	0.8403	0.2278	0.084
H9A	0.4657	0.7081	0.2930	0.112
H9B	0.4705	0.6044	0.2495	0.112
H9C	0.5094	0.6962	0.1987	0.112
H12A	0.7243	0.3395	0.5468	0.089
H12B	0.7008	0.4253	0.6125	0.089
H13A	0.5486	0.3646	0.6399	0.17
H13B	0.6203	0.2752	0.6477	0.17
H13C	0.5571	0.2895	0.5628	0.17
H12C	0.6144	0.2907	0.5299	0.085
H12D	0.7251	0.3271	0.5305	0.085
H13D	0.6801	0.4373	0.6447	0.162
H13E	0.6749	0.3275	0.6740	0.162
H13F	0.5781	0.3838	0.6497	0.162
H15A	0.3552	0.5046	0.5351	0.125
H15B	0.2893	0.5265	0.4528	0.125
H16A	0.2490	0.3670	0.4425	0.217

H16B	0.2238	0.4045	0.5368	0.217
H16C	0.3188	0.3418	0.5211	0.217
H19	0.9234	0.5028	0.5919	0.076
H20	0.9907	0.3708	0.5198	0.087
H21	0.9419	0.3322	0.3807	0.088
H22	0.8271	0.4277	0.3097	0.074
H25	0.6686	0.7678	0.5062	0.084
H26	0.5673	0.8990	0.5404	0.104
H27	0.5635	0.9597	0.6821	0.105
H28	0.6609	0.8892	0.7897	0.104
H29	0.7622	0.7580	0.7555	0.081
H30A	0.9985	0.8518	0.4002	0.109
H30B	1.0536	0.9196	0.3336	0.109
H30C	1.0251	0.8127	0.3068	0.109
H2	0.745(3)	0.656(4)	0.494(3)	0.104
H1	0.5738	0.4170	0.2548	0.106

12 Computational Details

12.1 Computational Studies to Determine Rotational Barriers

12.1.1 General Comments

All calculations were carried out using Gaussian 16[22] and all visualization of input and output files was accomplished using Gauss View 6.0.[23] All geometry optimizations and transition state optimizations were performed with B3LYP[24-26] functional using 6-31G(d) basis set. The vibrational frequencies were computed at the same level of theory as for the geometry optimizations and to evaluate the zero-point vibrational energy (ZPVE) and thermal corrections at 298 K. The single-point energies were computed based on the gas-phase optimized structures, using M06-2X[27] using Def2TZVP basis set. Rendering of computational outputs was accomplished using CYLview 20.[28]

12.1.2 Calculation of Rotational Barrier for 3aa

Structure	G^{a} (Ha)	Thermal correction^{b}	G_{corr} (Ha)	$\Delta G_{corr}^{\ddagger}$(Ha)
**GS1**	–1682.81698	0.452460	–1682.36452000	
**TS1**	–1682.75458	0.457533	–1682.29704700	0.067473
**GS2**	–1682.81698	0.452460	–1682.36452000	
**TS2**	–1682.761253	0.455286	–1682.30596700	0.05855300

^{a}Computed at M06-2X/Def2TZVP level of theory. ^{b}Computed at 298 K and B3LYP/6-31G(d) level of theory.

$\Delta G_{corr}^{\ddagger}(1) = 42.340$ kcal/mol $\rightarrow k_1 = 6.325 \times 10^{-19}$ s^{-1}
$\Delta G_{corr}^{\ddagger}(2) = 36.743$ kcal/mol $\rightarrow k_2 = 7.910 \times 10^{-15}$ s^{-1}

$K_{obs} = k_1 + k_2 = 7.911 \times 10^{-15}$ s$^{-1} \rightarrow \Delta G_{obs}^{\ddagger} = 36.742$ kcal/mol

GS1 optimization

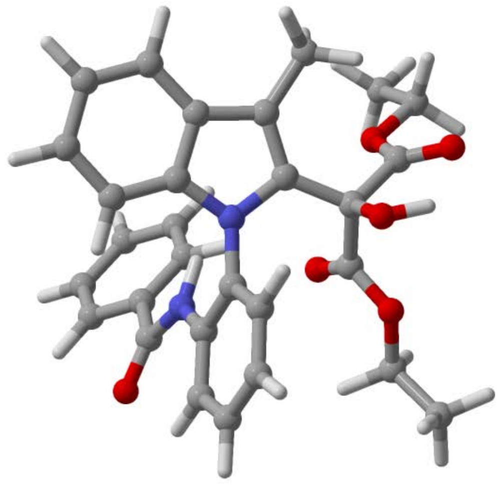

TS1 Optimization

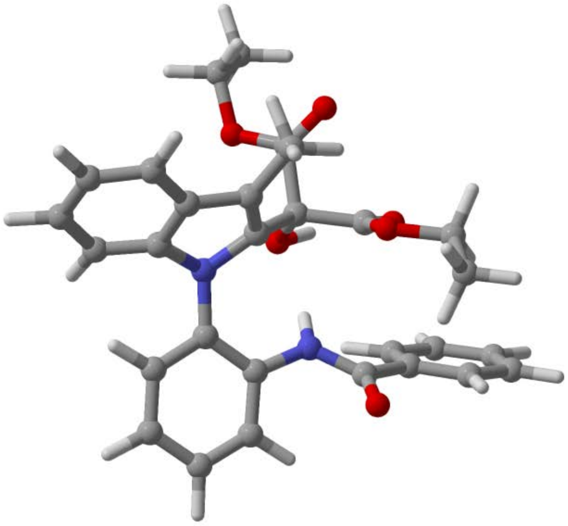

GS2 Optimization

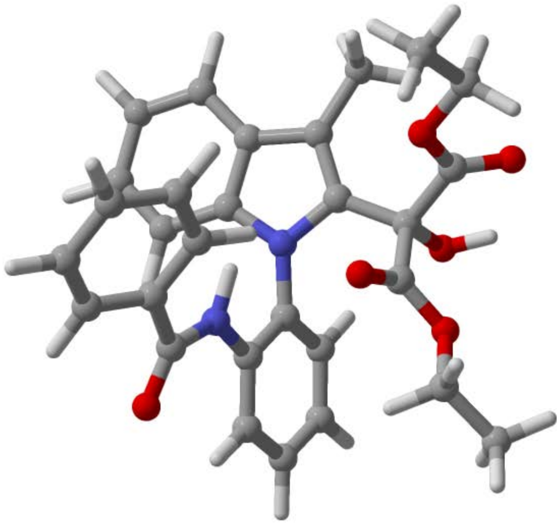

TS2 Optimization

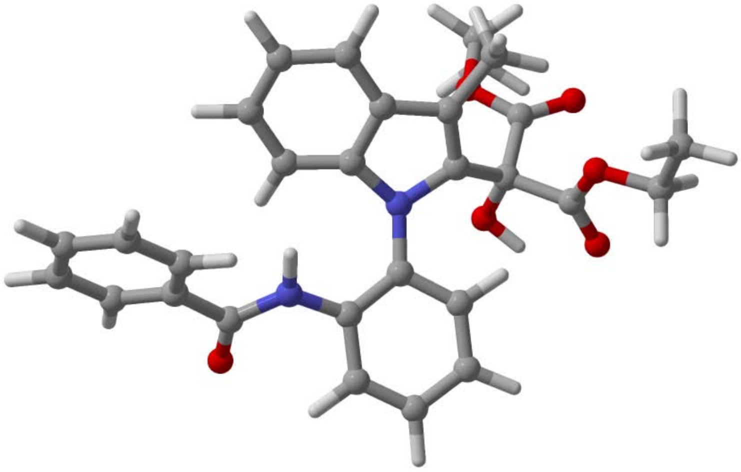

12.1.3 Calculation of Rotational Barrier for 5aa

Structure	G^a (Ha)	Thermal correction[b]	G_{corr} (Ha)	$\Delta G_{corr}^{‡}$(Ha)
**GS1**	–1682.820616	0.452464	–1682.36815200	
**TS1**	–1682.760805	0.457173	–1682.30363200	0.06452
**GS2**	–1682.820614	0.452462	–1682.36815200	
**TS2**	–1682.761104	0.455396	–1682.30570800	0.06244400

[a]Computed at M06-2X/Def2TZVP level of theory. [b]Computed at 298 K and B3LYP/6-31G(d) level of theory.

$\Delta G_{corr}^{‡}(1) = 40.487$ kcal/mol $\rightarrow k_1 = 1.437 \times 10^{-17}$ s^{-1}
$\Delta G_{corr}^{‡}(2) = 39.184$ kcal/mol $\rightarrow k_2 = 1.291 \times 10^{-16}$ s^{-1}

$K_{obs} = k_1 + k_2 = 1.435 \times 10^{-16}$ s$^{-1} \rightarrow \Delta G_{obs}^{‡} = 39.122$ kcal/mol

GS1 optimization

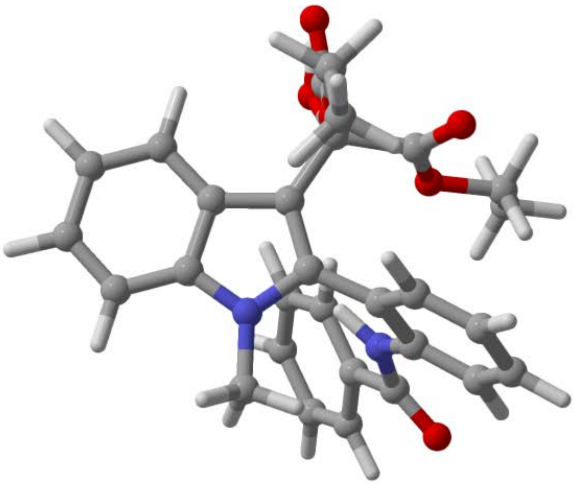

TS1 Optimization

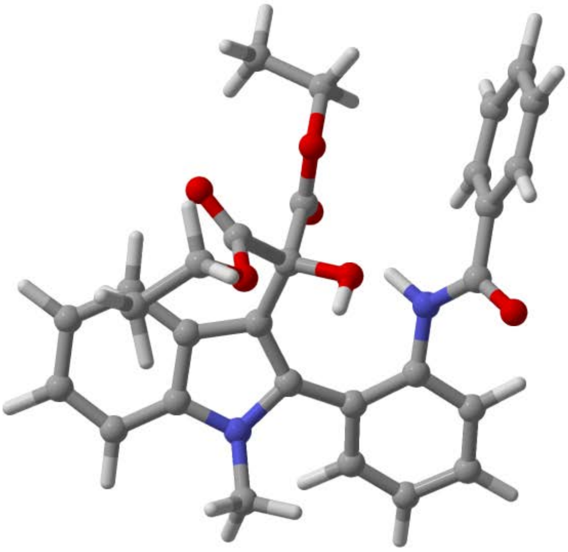

GS2 Optimization

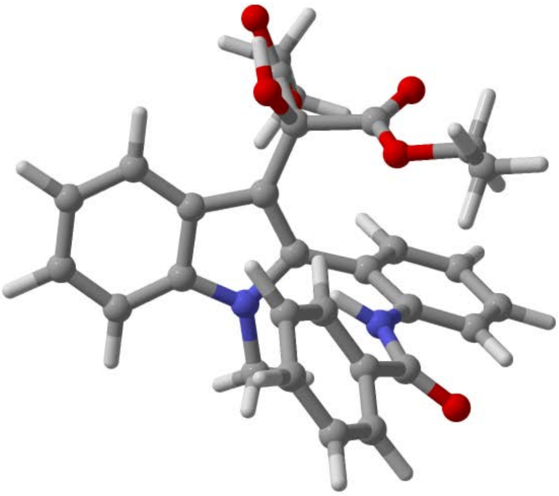

TS2 Optimization

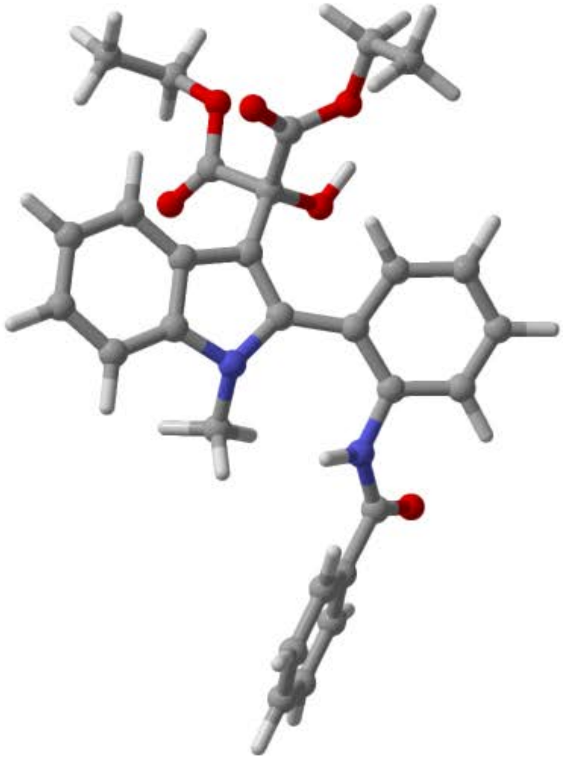

12.1.4 Calculation of Rotational Barrier for 7aa

Structure	G[a] (Ha)	Thermal correction[b]	G_{corr} (Ha)	$\Delta G_{corr}^{\ddagger}$(Ha)
**GS1**	–1682.818355	0.453281	–1682.36507400	
**TS1**	–1682.764557	0.457815	–1682.30674200	0.058332
**GS2**	–1682.818355	0.453281	–1682.36507400	
**TS2**	–1682.755626	0.456792	–1682.29883400	0.06624000

[a]Computed at M06-2X/Def2TZVP level of theory. [b]Computed at 298 K and B3LYP/6-31G(d) level of theory.

$\Delta G_{corr}^{\ddagger}(1) = 36.604$ kcal/mol $\rightarrow k_1 = 9.993 \times 10^{-15}$ s^{-1}
$\Delta G_{corr}^{\ddagger}(2) = 41.566$ kcal/mol $\rightarrow k_2 = 2.330 \times 10^{-18}$ s^{-1}

$K_{obs} = k_1 + k_2 = 9.995 \times 10^{-15}$ s$^{-1} \rightarrow \Delta G_{obs}^{\ddagger} = 36.604$ kcal/mol

GS1 optimization

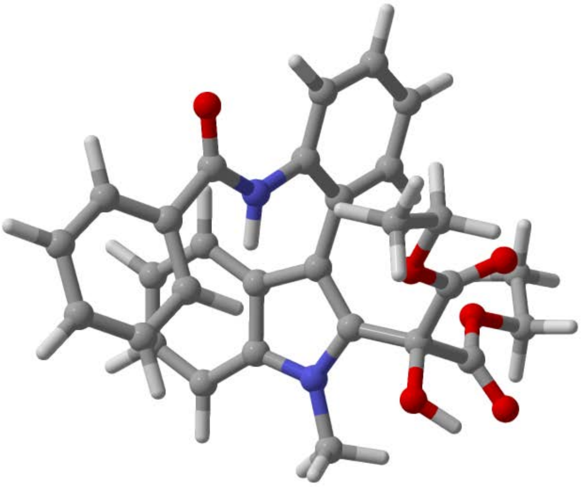

TS1 Optimization

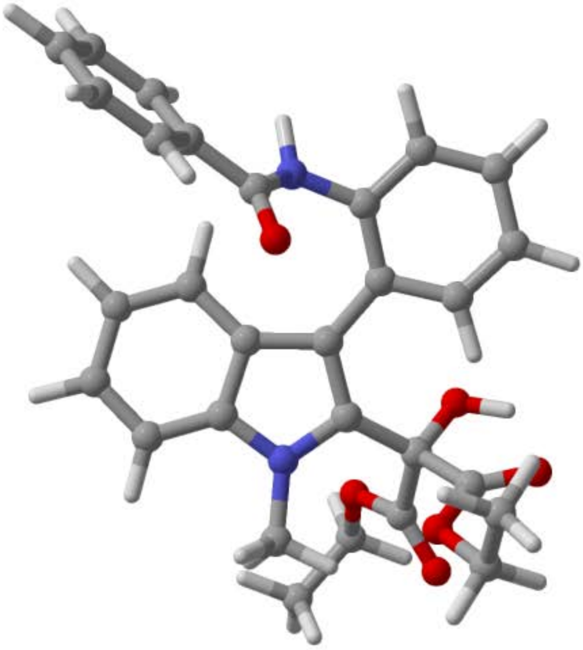

GS2 Optimization

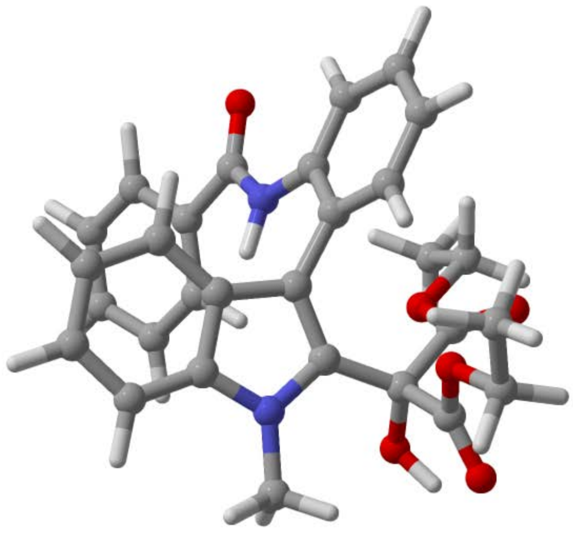

TS2 Optimization

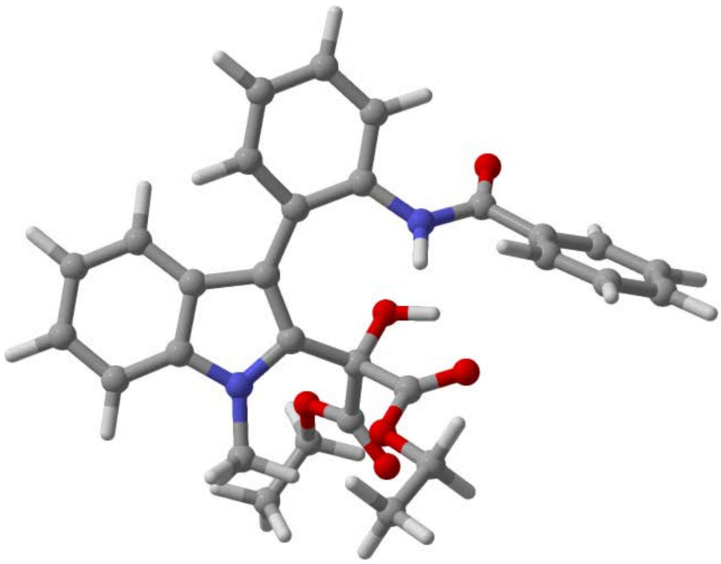

12.2 Computational Studies for ECD Spectra

12.2.1 General Comments

Conformational analyses were carried out via torsional sampling (MCMM) in Maestro 13.3 using the MMFF force filed with an energy cutoff of 2.5 kcal/mol. Based on the searched results, the conformers were re-optimized using Gaussian 16[22] with B3LYP[24-26] functional using 6-31G(d) basis set in methanol using the polarizable conductor calculation model (SMD). Then, single point calculations of the optimized conformers were performed through the time-dependent density functional theory electronic circular dichroism (TD-DFT/ECD) with B3LYP[24-26] using 6-31G(d) basis set in methanol using the polarizable conductor calculation model (SMD). The ECD spectra were simulated by the overlapping Gaussian function (half the bandwidth at 1/e peak height, sigma = 0.30 for all). Finally, the simulated spectra of the conformers were averaged according to the Boltzmann distribution theory and their relative Gibbs free energy.

12.2.2 ECD Spectra of 3aa

Calculated

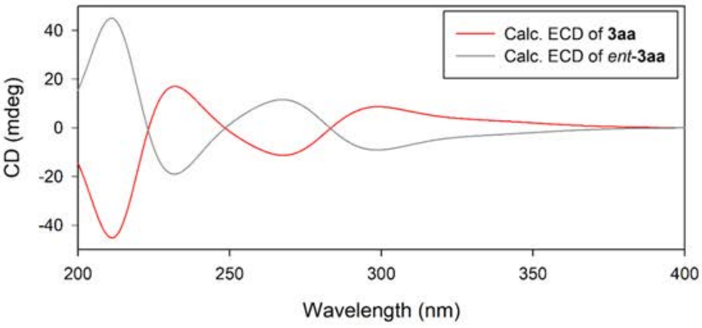

Measured

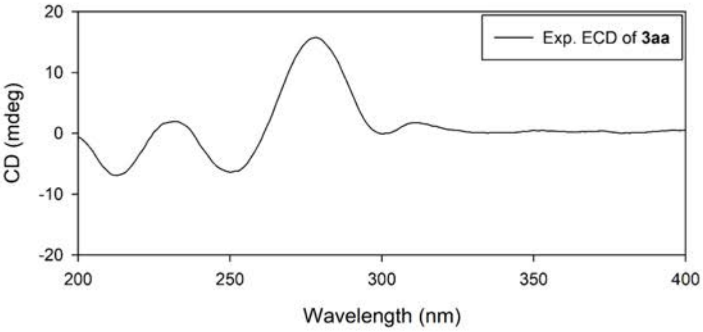

12.2.3 ECD Spectra of 5aa

Calculated

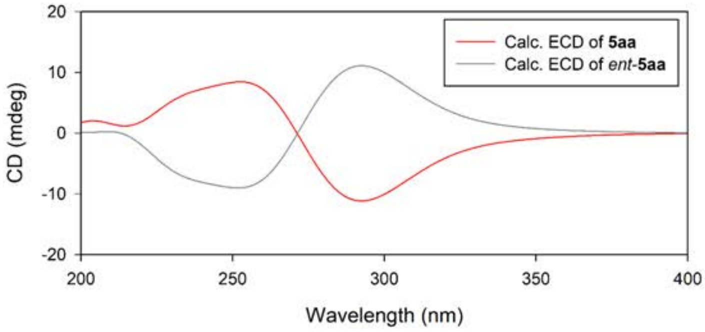

Measured

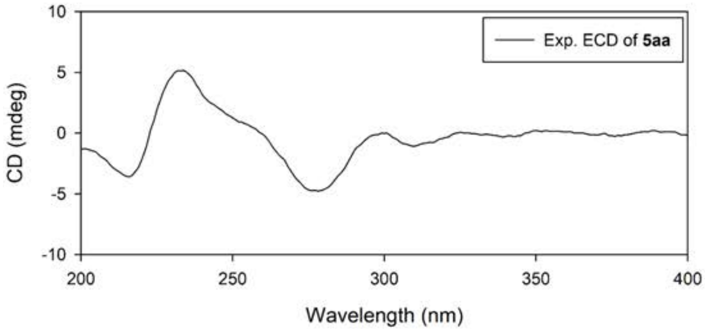

12.2.4 ECD Spectra of 7aa

Calculated

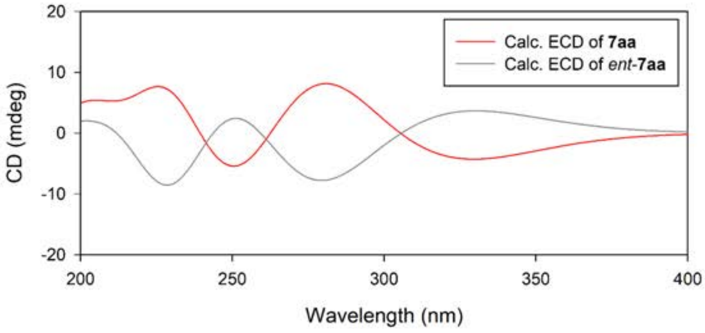

Measured

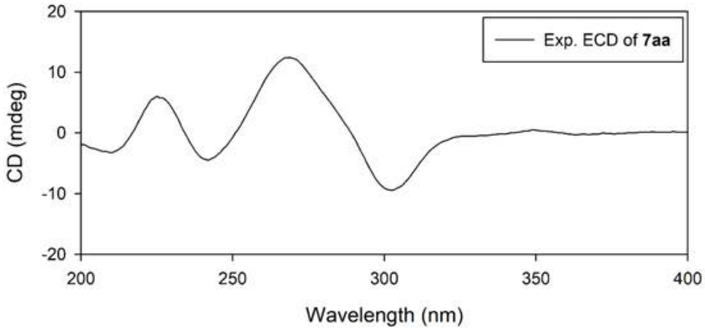

12.3 Computational Studies to Investigate the Origin of Enantioselectivity

12.3.1 General Comments

Conformational analyses were carried out via torsional sampling (MCMM) in Maestro 13.3 using the MMFF force filed with a maximum atom deviation cutoff of 2.0 Å. The constrained optimizations were performed with conformers generated from the conformational analyses using Gaussian 16.[22] Because of the bulky chiral catalyst, the two-layer quantum-mechanical (QM)/semiempirical (SE) ONIOM model[29-31] was applied to the catalytic complexes. The QM layer was treated with M06-2X[27]/6-31G(d) and the SE layer with PM6.[32] The phosphoric acid group [–O_2P(O)OH] in **P9** and substrates were assigned to the QM layer while the remaining atoms of **P9** were coated with the SE layer. Initial optimizations were performed with redundant coordinate freeze option by limiting the number of optimization steps at 30 (keyword 'maxcycles=30'). The conformers within 3 kcal/mol were selected and further optimized until the full convergence. With the two most stable conformers, the transition states were searched. The vibrational frequencies were computed at the same level of theory as for the geometry optimizations to evaluate the zero-point vibrational energy (ZPVE) and thermal corrections at 298 K. All optimized species were verified as either minima or transition structures by the presence of zero or a single imaginary vibrational frequency. The single-point energies of these optimized structures were calculated with M06-2X[27]/Def2TZVP for the QM layer and PM6[32] for the SE layer with the inclusion of solvation energy corrections using a self-consistent reaction field based on SMD implicit solvent model[33] with toluene as a solvent. Rendering of computational outputs was accomplished using CYLview 20.[28]

12.3.2 Conformational analyses for TS1-3aa

Conformer	G[a] (Initial 30 steps) (kcal/mol)	G[a] (Full convergence) (kcal/mol)	TS[b] (kcal/mol)
1	−1459669.928	−1459670.755	-
2	−1459669.130	-	-
3	−1459669.672	−1459670.731	-
4	−1459669.128	-	-
5	−1459669.865	−1459670.178	-
6	−1459668.921	-	-
7	−1459667.778	-	-
8	−1459669.760	−1459670.670	-
9	−1459668.230	-	-
10	−1459667.672	-	-
11	−1459667.624	-	-
12	−1459670.484	−1459671.049	−1459220.514 (**TS1-3aa**)
13	−1459668.858	-	-
14	−1459670.126	−1459672.293	−1459218.949
15	−1459667.196	-	-
16	−1459668.221	-	-
17	−1459665.699	-	-
18	−1459665.384	-	-
19	−1459670.069	−1459670.537	-
20	−1459663.058	-	-
21	−1459667.144	-	-
22	−1459664.115	-	-
23	−1459664.136	-	-
24	−1459668.977	-	-
25	−1459666.213	-	-
26	−1459665.798	-	-

[a]Computed using the QM/SE ONIOM model (M06-2X/6-31G(d)):PM6) level of theory. [b]Computed using the QM/SE ONIOM model (M06-2X/Def2TZVP):PM6) level of theory with the inclusion of solvation energy corrections (SMD, toluene) and thermal correction at 268.15 K.

12.3.3 Conformational analyses for TS2-3aa

Conformer	G[a] (Initial 30 steps) (kcal/mol)	G[a] (Full convergence) (kcal/mol)	TS[b] (kcal/mol)
1	−1459672.564	−1459674.623	-
2	−1459672.604	−1459674.324	-
3	−1459673.011	−1459674.860	−1459218.453 (**TS2-3aa**)
4	−1459657.850	-	-
5	−1459657.069	-	-
6	−1459673.485	−1459675.719	−1459217.643
7	−1459672.515	−1459674.430	-
8	−1459672.749	−1459674.589	-
9	−1459654.887	-	-
10	−1459672.680	−1459674.734	-
11	−1459667.659	-	-
12	−1459668.796	-	-
13	−1459672.701	−1459674.766	-
14	−1459659.333	-	-
15	−1459656.026	-	-
16	−1459669.027	-	-
17	−1459668.559	-	-
18	−1459669.787	-	-
19	−1459668.652	-	-
20	−1459659.403	-	-
21	−1459666.661	-	-
22	−1459668.364	-	-
23	−1459672.602	−1459674.805	-
24	−1459667.625	-	-
25	−1459669.335	-	-
26	−1459668.634	-	-
27	−1459668.840	-	-
28	−1459657.654	-	-
29	−1459669.077	-	-
30	−1459668.686	-	-
31	−1459667.281	-	-
32	−1459668.897	-	-
33	−1459656.546	-	-
34	−1459658.433	-	-
35	−1459671.739	−1459674.839	-
36	−1459669.392	-	-
37	−1459668.265	-	-
38	−1459668.464	-	-
39	−1459668.404	-	-
40	−1459668.590	-	-
41	−1459669.643	-	-
42	−1459667.258	-	-
43	−1459667.264	-	-

44	−1459667.005	-	-
45	−1459668.608	-	-
46	−1459669.096	-	-
47	−1459668.277	-	-
48	−1459668.543	-	-
49	−1459668.175	-	-
50	−1459668.437	-	-
51	−1459667.088	-	-
52	−1459667.392	-	-

[a]Computed using the QM/SE ONIOM model (M06-2X/6-31G(d)):PM6) level of theory. [b]Computed using the QM/SE ONIOM model (M06-2X/Def2TZVP):PM6) level of theory with the inclusion of solvation energy corrections (SMD, toluene) and thermal correction at 268.15 K.

12.3.4 Computed Structures, Energy and Imaginary Vibrational Frequency of Calculated Structures of 3aa

Structure	G^a (Ha)	Thermal correction[b]	G_{corr} (Ha)	Imaginary frequency[b]
**TS1-3aa**	−2327.09863669	1.666148	−2325.43248869	1 (−630.7651)
**TS2-3aa**	−2327.09918874	1.669984	−2325.42920474	1 (−397.4277)

[a]Computed using the QM/SE ONIOM model (M06-2X/Def2TZVP):PM6) level of theory with the inclusion of solvation energy corrections (SMD, toluene). [b]Computed at 268.15 K using the QM/SE ONIOM model (B3LYP/6-31G(d):PM6) level of theory.

TS1-3aa Optimization

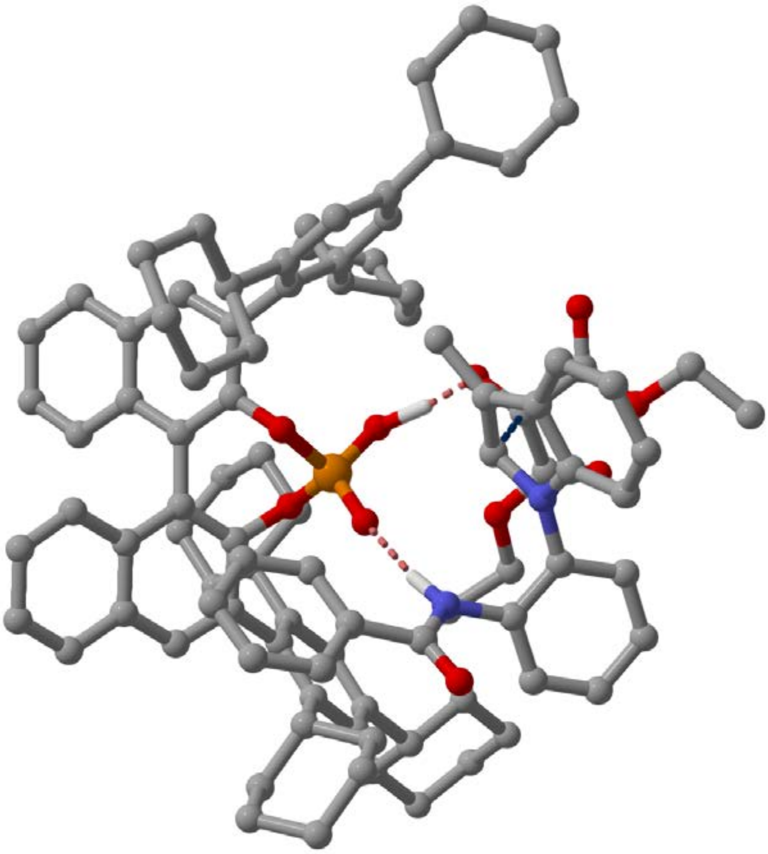

TS2-3aa Optimization

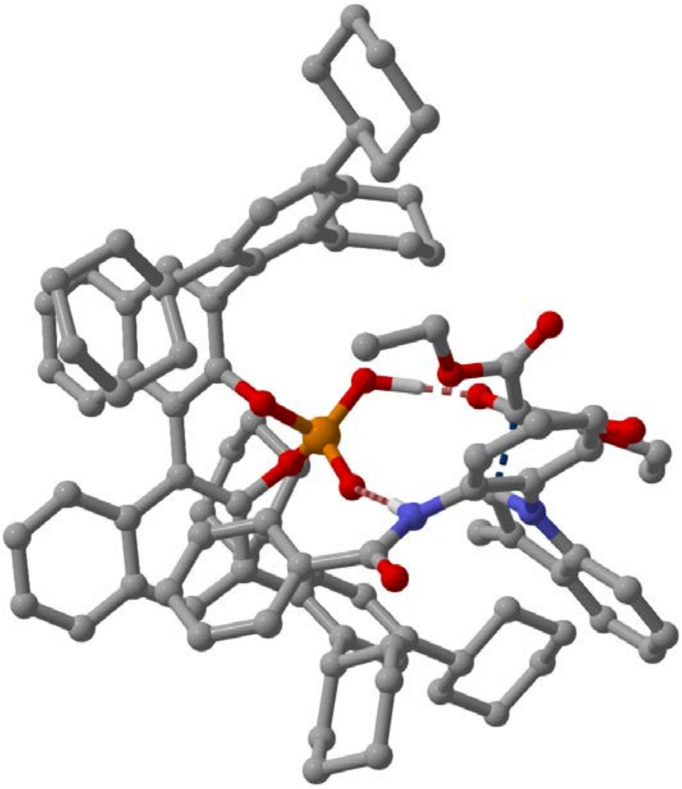

NCI analysis of **TS2-3aa**

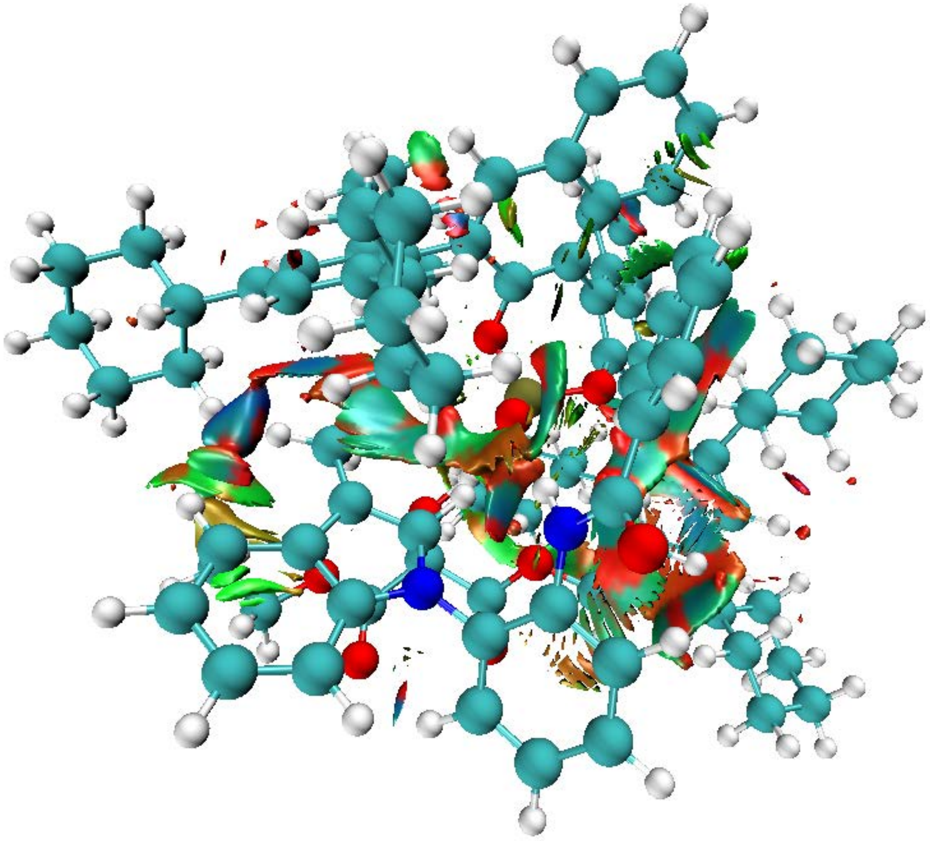

The NCI analysis of **TS2-3aa** revealed unfavorable interactions between the substituents of **P9** and the benzoyl group of **1a**.

12.3.5 Conformational analyss for TS1-5aa

Conformer	G^a (Initial 30 steps) (kcal/mol)	G^a (Full convergence) (kcal/mol)	TS^b (kcal/mol)
1	−1459675.495	−1459682.798	
2	−1459673.595	−1459681.126	
3	−1459675.362	−1459683.517	
4	−1459672.709	-	
5	−1459672.727	-	
6	−1459674.460	−1459683.639	−1459193.079 (**TS1-5aa**)
7	−1459672.585	-	
8	−1459674.036	−1459683.499	
9	−1459670.956	-	
10	−1459670.617	-	
11	−1459672.256	-	
12	−1459669.690	-	
13	−1459675.959	−1459683.514	
14	−1459676.000	−1459683.520	−1459192.443
15	−1459670.984	-	
16	−1459673.725	−1459681.550	
17	−1459674.457	−1459682.444	
18	−1459673.123	−1459681.384	
19	−1459670.824	-	
20	−1459674.982	−1459682.640	
21	−1459670.222	-	
22	−1459670.364	-	
23	−1459672.773	-	
24	−1459670.817	-	
25	−1459671.511	-	
26	−1459671.565	-	
27	−1459673.745	−1459681.754	
28	−1459669.996	-	
29	−1459673.626	−1459681.721	
30	−1459671.387	-	
31	−1459668.213	-	
32	−1459670.201	-	

[a]Computed using the QM/SE ONIOM model (M06-2X/6-31G(d)):PM6) level of theory. [b]Computed using the QM/SE ONIOM model (M06-2X/Def2TZVP):PM6) level of theory with the inclusion of solvation energy corrections (SMD, toluene) and thermal correction at 195.15 K.

12.3.6 Conformational analyses for TS2-5aa

Conformer	G[a] (Initial 30 steps) (kcal/mol)	G[a] (Full convergence) (kcal/mol)	TS[b] (kcal/mol)
1	−1459678.409	−1459681.672	−1459192.042 (**TS2-5aa**)
2	−1459678.032	−1459681.360	
3	−1459675.384	-	
4	−1459675.022	-	
5	−1459672.892	-	
6	−1459672.805	-	
7	−1459672.349	-	
8	−1459673.652	-	
9	−1459671.353	-	
10	−1459674.294	-	
11	−1459674.455	-	
12	−1459672.451	-	
13	−1459674.645	-	
14	−1459674.797	-	
15	−1459675.424	−1459679.353	
16	−1459671.921	-	
17	−1459673.230	-	
18	−1459674.252	-	
19	−1459673.155	-	
20	−1459670.883	-	
21	−1459674.385	-	
22	−1459671.006	-	
23	−1459675.763	−1459679.871	
24	−1459672.056	-	
25	−1459672.018	-	
26	−1459673.576	-	
27	−1459677.552	−1459682.967	−1459190.790
28	−1459672.050	-	
29	−1459674.913	-	
30	−1459675.440	−1459679.183	
31	−1459670.329	-	
32	−1459671.416	-	
33	−1459666.436	-	
34	−1459665.520	-	
35	−1459673.858	-	
36	−1459669.377	-	
37	−1459668.950	-	
38	−1459666.331	-	
39	−1459667.017	-	
40	−1459670.245	-	
41	−1459668.685	-	

[a]Computed using the QM/SE ONIOM model (M06-2X/6-31G(d)):PM6) level of theory. [b]Computed using the QM/SE ONIOM model (M06-2X/Def2TZVP):PM6)

level of theory with the inclusion of solvation energy corrections (SMD, toluene) and thermal correction at 195.15 K.

12.3.7 Computed Structures, Energy and Imaginary Vibrational Frequency of Calculated Structures of 5aa

Structure	G^a (Ha)	Thermal correction[b]	G_{corr} (Ha)	Imaginary frequency[b]
**TS1-5aa**	−2327.10954522	1.720777	−2325.38876822	1 (−307.2159)
**TS2-5aa**	−2327.10937444	1.722259	−2325.38512097	1 (−352.6505)

[a]Computed using the QM/SE ONIOM model (M06-2X/Def2TZVP):PM6) level of theory with the inclusion of solvation energy corrections (SMD, toluene). [b]Computed at 195.15 K using the QM/SE ONIOM model (M06-2X/6-31G(d):PM6) level of theory.

TS1-5aa Optimization

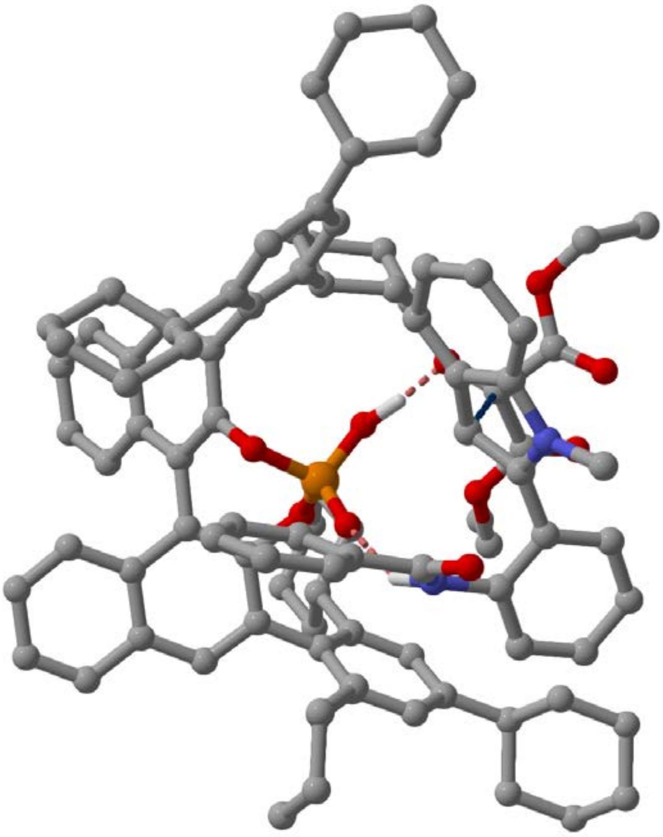

TS2-5aa Optimization

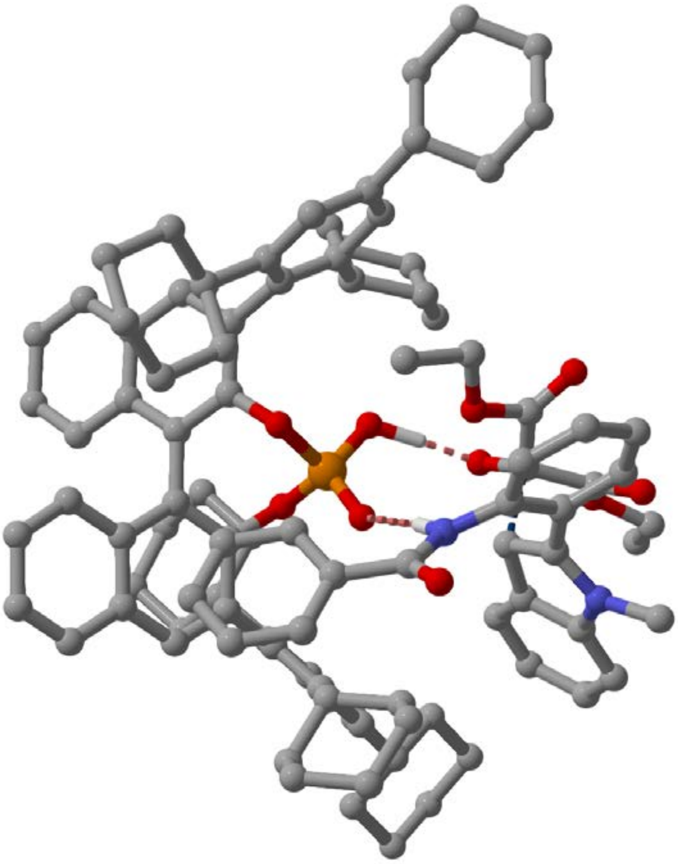

NCI analysis of **TS2-5aa**

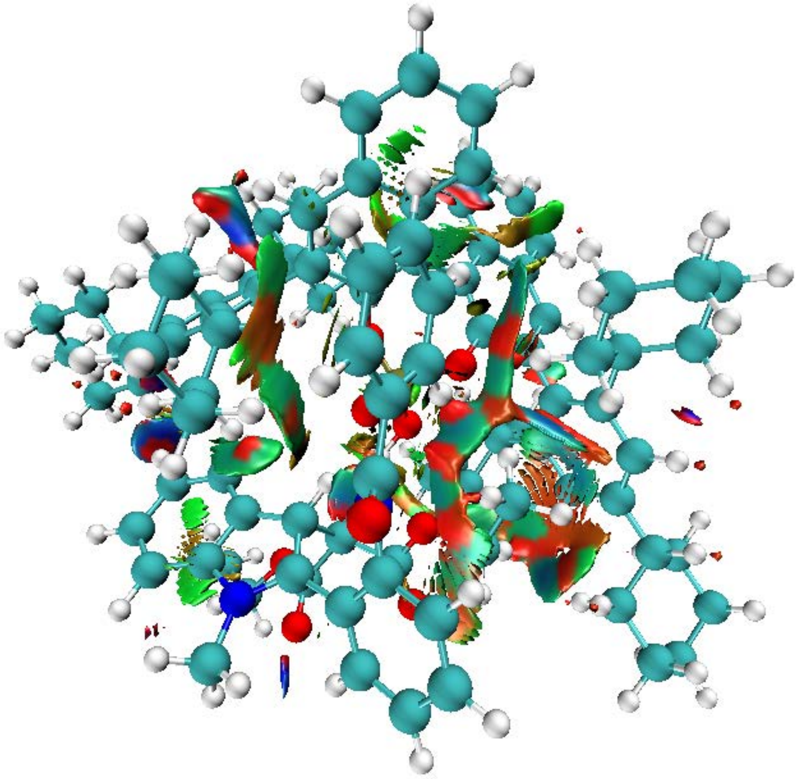

The NCI analysis of **TS2-5aa** revealed unfavorable steric interactions between the catalyst and the benzoyl group of **4a**.

12.3.8 Conformational analyses for TS1-7aa

Conformer	G^{a} (Initial 30 steps) (kcal/mol)	G^{a} (Full convergence) (kcal/mol)	TS^{b} (kcal/mol)
1	−1459668.107	−1459669.153	
2	−1459668.230	−1459669.324	−1459228.311 (**TS1-7aa**)
3	−1459666.885	−1459669.110	
4	−1459667.920	−1459667.693	
5	−1459668.063	−1459669.256	
6	−1459667.499	−1459668.482	
7	−1459667.154	−1459668.123	
8	−1459667.723	−1459667.918	
9	−1459667.432	−1459668.399	
10	−1459666.671	−1459667.904	
11	−1459667.145	−1459668.109	
12	−1459667.044	−1459667.808	
13	−1459668.341	−1459669.153	−1459227.476
14	−1459667.584	−1459669.109	
15	−1459667.639	−1459668.141	
16	−1459667.640	−1459668.130	
17	−1459666.685	−1459667.671	
18	−1459667.768	−1459668.694	
19	−1459666.626	−1459668.212	
20	−1459668.077	−1459668.694	
21	−1459668.057	−1459668.740	
22	−1459667.540	−1459668.164	
23	−1459667.255	−1459668.142	
24	−1459667.052	−1459668.105	
25	−1459667.002	−1459668.122	
26	−1459666.855	−1459667.646	
27	−1459663.570		
28	−1459663.637		
29	−1459664.173		
30	−1459663.901		
31	−1459663.281		
32	−1459665.096		

[a]Computed using the QM/SE ONIOM model (M06-2X/6-31G(d)):PM6) level of theory. [b]Computed using the QM/SE ONIOM model (M06-2X/Def2TZVP):PM6) level of theory with the inclusion of solvation energy corrections (SMD, dichloromethane) and thermal correction at 268.15 K.

12.3.6 Conformational analyses for TS2-7aa

Conformer	G[a] (Initial 30 steps) (kcal/mol)	G[a] (Full convergence) (kcal/mol)	TS[b] (kcal/mol)
1	−1459671.575	−1459672.021	−1459226.845 (**TS2-7aa**)
2	−1459671.405	−1459671.933	
3	−1459671.214	−1459671.730	
4	−1459671.160	−1459671.798	
5	−1459670.867	−1459671.607	
6	−1459671.352	−1459673.211	−1459226.752
7	−1459671.071	−1459671.633	
8	−1459666.860	-	
9	−1459667.960	-	
10	−1459666.978	-	
11	−1459666.867	-	
12	−1459666.689	-	
13	−1459668.933	−1459669.091	
14	−1459666.814	-	
15	−1459670.340	−1459670.782	
16	−1459670.103	−1459670.587	
17	−1459667.254	-	
18	−1459666.325	-	
19	−1459669.494	−1459670.093	
20	−1459669.223	−1459669.856	
21	−1459670.333	−1459670.670	
22	−1459666.203	-	
23	−1459669.901	−1459670.289	
24	−1459668.564	-	
25	−1459668.246	-	
26	−1459667.339	-	
27	−1459666.464	-	
28	−1459668.110	-	
29	−1459667.257	-	
30	−1459668.334	-	
31	−1459668.188	-	
32	−1459666.533	-	
33	−1459667.158	-	
34	−1459667.113	-	
35	−1459669.730	−1459670.015	
36	−1459666.523	-	
37	−1459667.532	-	
38	−1459666.108	-	
39	−1459667.194	-	

[a]Computed using the QM/SE ONIOM model (M06-2X/6-31G(d)):PM6) level of theory. [b]Computed using the QM/SE ONIOM model (M06-2X/Def2TZVP):PM6) level of theory with the inclusion of solvation energy corrections (SMD, dichloromethane) and thermal correction at 268.15 K.

12.3.4 Computed Structures, Energy and Imaginary Vibrational Frequency of Calculated Structures of 7aa

Structure	G^a (Ha)	Thermal correction[b]	G_{corr} (Ha)	Imaginary frequency[b]
**TS1-7aa**	−2327.11279385	1.667879	−2325.44491485	1 (−758.8975)
**TS2-7aa**	−2327.11328643	1.670709	−2325.44257743	1 (−777.5932)

[a]Computed using the QM/SE ONIOM model (M06-2X/Def2TZVP):PM6) level of theory with the inclusion of solvation energy corrections (SMD, dichloromethane). [b]Computed at 268.15 K using the QM/SE ONIOM model (B3LYP/6-31G(d):PM6) level of theory.

TS1-7aa Optimization

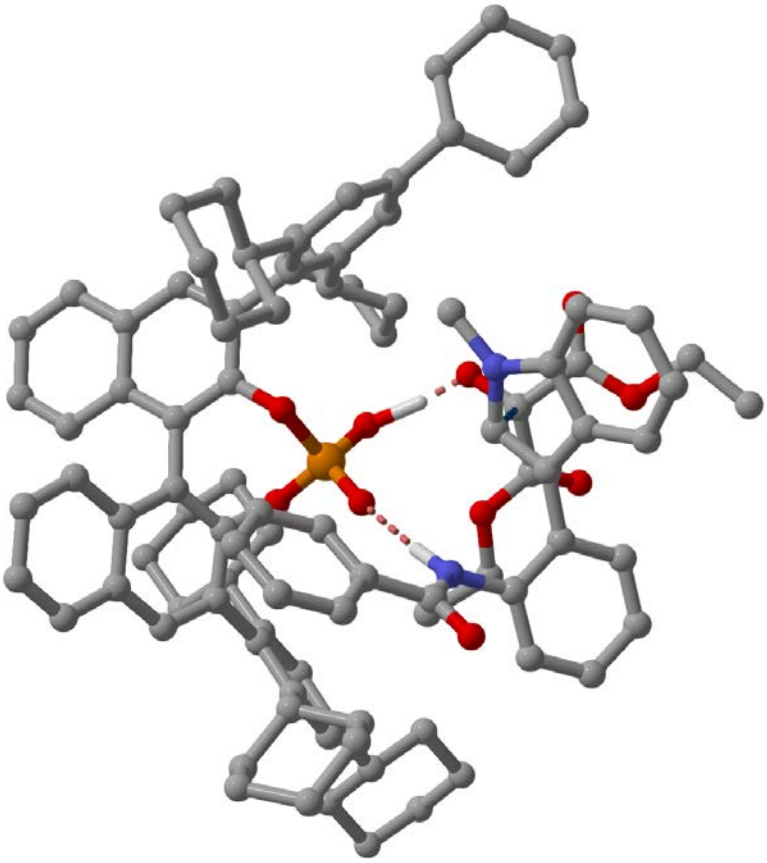

TS2-7aa Optimization

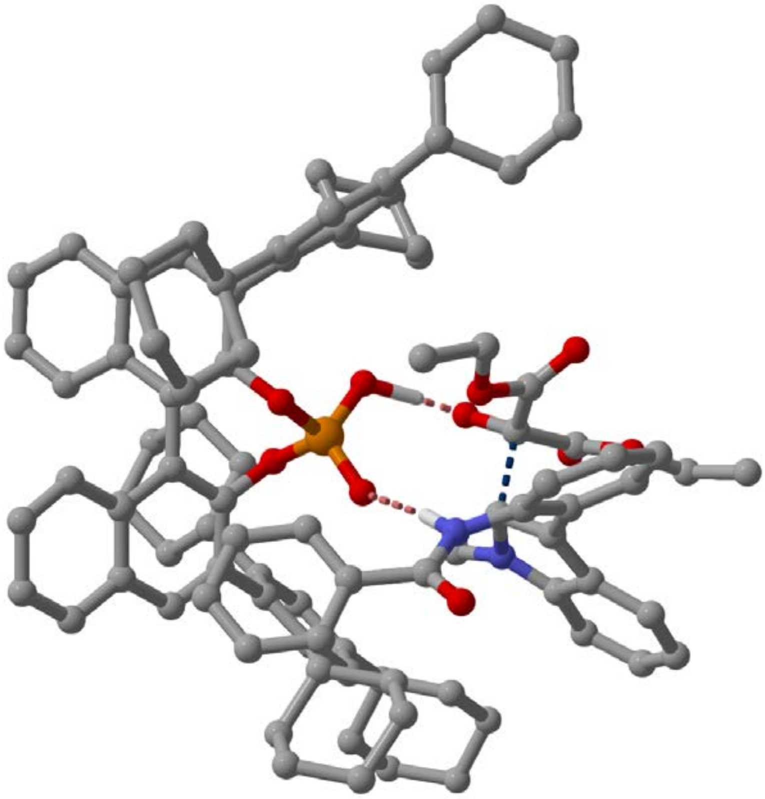

NCI analysis of **TS2-7aa**

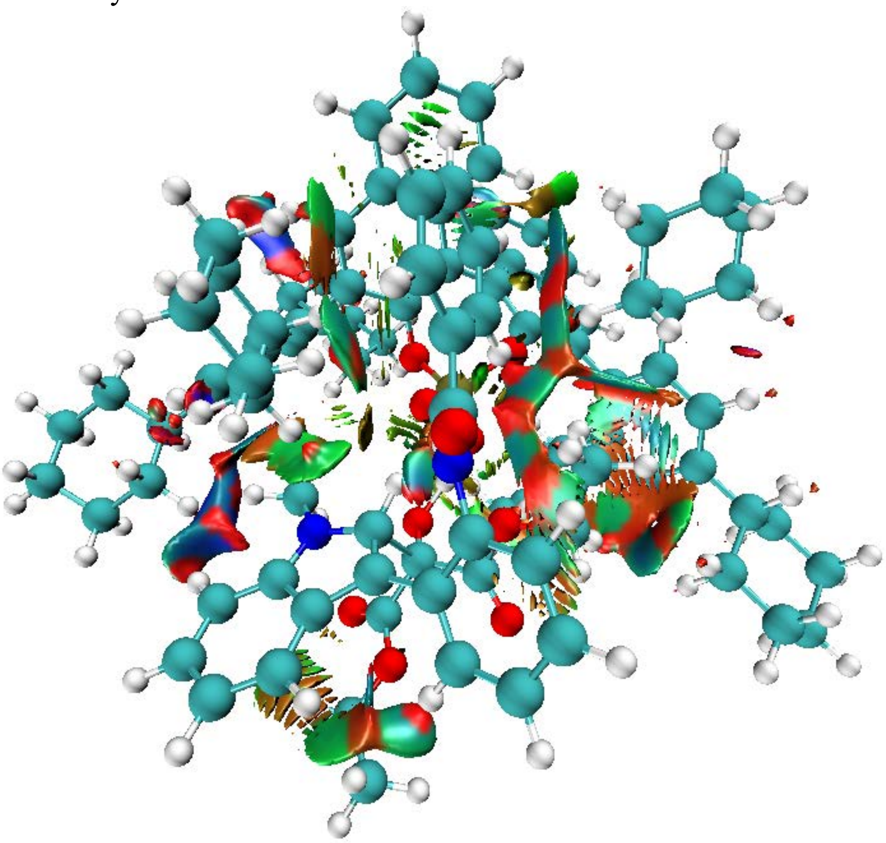

The NCI analysis of **TS2-7aa** revealed steric interactions between the catalyst and the benzoyl group of the substrate.

13 Biological Evaluation

Cell culture

A549: Human lung cancer cell line, HCT116: Human colon cancer cell line, MDA-MB-231: Human breast cancer cell line, SK-Hep-1: Human liver cancer cell line, and SNU-638: Human stomach cancer cell line were obtained from the Korean Cell Line Bank (Seoul, Korea). Cells were grown in medium (DMEM for SK-Hep-1 and MDA-MB-231 cells; RPMI 1640 medium for A549, HCT116, and SNU-638) supplemented with 10% fetal bovine serum and antibiotics-antimycotics (100 units/mL penicillin G sodium, 100 μg/mL streptomycin, and 250 ng/mL amphotericin B).

Cell proliferation assay

To evaluate the growth inhibitory activities of test compounds against various human cancer cells, sulforhodamine B (SRB) assay was performed. Cells were seeded in 96-well plates at 1×10^4 cells/well, and compounds that had been diluted to various concentrations in medium were added to the cells for 72 h. Cells were fixed with 10% trichloroacetic acid solution for 30 min at 4°C, washed five times with tap water, and air dried. The cells were stained with 0.4% SRB in 1% acetic acid solution for 30 min at room temperature. After washing unbound dye and drying, the stained cells were dissolved in 10 mM Tris (pH 10.0), and absorbance was measured at 515 nm. Cell viability was calculated by comparison with absorbance of the vehicle-treated control group. The concentration of 50% cell survival (IC_{50}) was determined by non-linear regression analysis using GraphPad Prism 6.

14 References

1. Fulmer GR, *et al.* NMR Chemical Shifts of Trace Impurities: Common Laboratory Solvents, Organics, and Gases in Deuterated Solvents Relevant to the Organometallic Chemist. *Organometallics* **29**, 2176−2179 (2010).

2. Xie C, *et al.* Dimethyl Sulfoxide Involved One-Pot Synthesis of Quinoxaline Derivatives. *J. Org. Chem.* **82**, 3491–3499 (2017).

3. Nan J, Ma Q, Yin J, Liang C, Tian L, Ma Y. RhIII-Catalyzed formal [5 + 1] cyclization of 2-pyrrolyl/indolylanilines using vinylene carbonate as a C1 synthon. *Org. Chem. Front.* **8**, 1764-1769 (2021).

4. Hartwig JF, Kawatsura M, Hauck SI, Shaughnessy KH, Alcazar-Roman LM. Room-Temperature Palladium-Catalyzed Amination of Aryl Bromides and Chlorides and Extended Scope of Aromatic C−N Bond Formation with a Commercial Ligand. *J. Org. Chem.* **64**, 5575–5580 (1999).

5. Chen X, Lin J, Wang B, Tian X. Nickel-Catalyzed Mizoroki–Heck/Amination Cascade Reactions of o-Dihaloarenes with Allylamines: Synthesis of Indoles. *Org. Lett.* **22**, 7704–7708 (2020).

6. Rubio-Presa R, Pedrosa MaR, Fernández-Rodríguez MA, Arnáiz FJ, Sanz R. Molybdenum-Catalyzed Synthesis of Nitrogenated Polyheterocycles from Nitroarenes and Glycols with Reuse of Waste Reduction Byproduct. *Org. Lett.* **19**, 5470–5473 (2017).

7. Zhao J, Zhang Y, Cheng K. Palladium-Catalyzed Direct C-2 Arylation of Indoles with Potassium Aryltrifluoroborate Salts. *J. Org. Chem.* **73**, 7428–7431 (2008).

8. Deprez NR, Kalyani D, Krause A, Sanford MS. Room Temperature Palladium-Catalyzed 2-Arylation of Indoles. *J. Am. Chem. Soc.* **128**, 4972–4973 (2006).

9. Bhunia SK, Polley A, Natarajan R, Jana R. Through-Space 1,4-Palladium Migration and 1,2-Aryl Shift: Direct Access to Dibenzo[a,c]carbazoles through a Triple C–H Functionalization Cascade. *Chem. Eur. J.* **21**, 16786–16791 (2015).

10. Agarwal PK, Sawant D, Sharma S, Kundu B. New Route to the Synthesis of the Isocryptolepine Alkaloid and Its Related Skeletons Using a Modified Pictet–Spengler Reaction. *Eur. J. Org. Chem.* **2009**, 292–303 (2009).

11. Ahmed J, *et al.* Direct C–H Arylation of Heteroarenes with Aryl Chlorides by Using an Abnormal N-Heterocyclic-Carbene–Palladium Catalyst. *Eur. J. Org. Chem.* **2017**, 1004–1011 (2017).

12. Bras JP, De Cointet P. A process for the preparation of 6-chloro-11H-indolo[3,2-c]quinolines useful as intermediates in the pharmaceutical and chemical industry Patent FR2590899 (1987).

13. Abe T, Yamashiro T, Shimizu K, Sawada D. Indole Editing Enabled by HFIP-Mediated Ring-Switch Reactions of 3-Amino-2-Hydroxyindolines. *Chem. Eur. J.* **28**, e202201113 (2022).

14. Kiang AK, Mann FG, Prior AF, Topham A. 268. The action of acyl cyanides on 2- and 1 : 2-substituted indoles. Part II. Derivatives of 2-o-aminophenylindole. *J. Chem. Soc.* 1319–1331 (1956).

15. Chowdhury R, Mendoza A. N-Hydroxyphthalimidyl diazoacetate (NHPI-DA): a modular methylene linchpin for the C–H alkylation of indoles. *Chem. Commun.* **57**, 4532–4535 (2021).

16. Liang Z, Yao B, Zhang Y. Pd(OAc)2-Catalyzed Regioselective Arylation of Indoles with Arylsiloxane in Acidic Medium. *Org. Lett.* **12**, 3185–3187 (2010).

17. Saito K, Chikkade PK, Kanai M, Kuninobu Y. Palladium-Catalyzed Construction of Heteroatom-Containing π-Conjugated Systems by Intramolecular Oxidative C–H/C–H Coupling Reaction. *Eur. J. Chem.* **21**, 8365–8368 (2015).

18. Badigenchala S, Rajeshkumar V, Sekar G. Iodine mediated intramolecular C2-amidative cyclization of indoles: a facile access to indole fused tetracycles. *Org. Biomol. Chem.* **14**, 2297–2305 (2016).

19. Choi I, Chung H, Park JW, Chung YK. Active and Recyclable Catalytic Synthesis of Indoles by Reductive Cyclization of 2-(2-Nitroaryl)acetonitriles in the Presence of Co–Rh Heterobimetallic Nanoparticles with Atmospheric Hydrogen under Mild Conditions. *Org. Lett.* **18**, 5508–5511 (2016).

20. Maddox SM, Nalbandian CJ, Smith DE, Gustafson JL. A Practical Lewis Base Catalyzed Electrophilic Chlorination of Arenes and Heterocycles. *Org. Lett.* **17**, 1042–1045 (2015).

21. Lee Y-J, Oh H-K, Ji H-S, Jeong W-J, Choi J-S, Choi D-H. Preparation of azacarbazole derivative for organic light emitting diode Patent WO2020067594 (2020).

22. Frisch MJ*, et al.* Gaussian 16 Rev. C.01. Gaussian, Inc. (2016).

23. Dennington R, Keith TA, Millam JM. GaussView, Version 6. Semichem Inc. (2016).

24. Lee C, Yang W, Parr RG. Development of the Colle-Salvetti correlation-energy formula into a functional of the electron density. *Phy. Rev. B* **37**, 785−789 (1988).

25. Becke AD. A new mixing of Hartree–Fock and local density-functional theories. *J. Chem. Phys.* **98**, 1372−1377 (1993).

26. Becke AD. Density-functional thermochemistry. III. The role of exact exchange. *J. Chem. Phys.* **98**, 5648−5652 (1993).

27. Zhao Y, Truhlar DG. The M06 suite of density functionals for main group thermochemistry, thermochemical kinetics, noncovalent interactions, excited states, and transition elements: two new functionals and systematic testing of four M06-class functionals and 12 other functionals. *Theor. Chem. Acc.* **120**, 215−241 (2008).

28. Legault CY. CYLview20.) (2020).

29. Li Z, Liu L, Fu Y, Guo Q-X. Assessing performance of diverse ONIOM methods in calculation of structures of organonickel and organopalladium compounds. *THEOCHEM* **757**, 69−76 (2005).

30. Ananikov VP, Musaev DG, Morokuma K. Real size of ligands, reactants and catalysts: Studies of structure, reactivity and selectivity by ONIOM and other hybrid computational approaches. *J. Mol. Catal. A: Chem.* **324**, 104−119 (2010).

31. Karami K, Abedanzadeh S, Farrokhpour H, Lipkowski J. Synthesis and characterization of the P,C-palladacycles with bridging and chelating dinitrogen ligands and ONIOM calculations on the pyrazine-bridged organometallic polymers (n = 1 to n = 10). *J. Organomet. Chem.* **805**, 68−76 (2016).

32. Stewart JJP. Optimization of parameters for semiempirical methods V: Modification of NDDO approximations and application to 70 elements. *J. Mol. Model.* **13**, 1173−1213 (2007).

33. Marenich AV, Cramer CJ, Truhlar DG. Universal Solvation Model Based on Solute Electron Density and on a Continuum Model of the Solvent Defined by the Bulk Dielectric Constant and Atomic Surface Tensions. *J. Phys. Chem. B* **113**, 6378−6396 (2009).
